# Bacille Calmette-Guérin vaccination to prevent febrile and respiratory illness in adults (BRACE): secondary outcomes of a randomised controlled phase 3 trial

**DOI:** 10.1016/j.eclinm.2024.102616

**Published:** 2024-05-13

**Authors:** Laure F. Pittet, Nicole L. Messina, Ellie McDonald, Francesca Orsini, Simone Barry, Marc Bonten, John Campbell, Julio Croda, Mariana G. Croda, Margareth Dalcolmo, Kaya Gardiner, Amanda Gwee, Bruno Jardim, Marcus V.G. Lacerda, Michaela Lucas, David J. Lynn, Laurens Manning, Kirsten P. Perrett, Jeffrey J. Post, Cristina Prat-Aymerich, Peter C. Richmond, Jorge L. Rocha, Jesus Rodriguez-Baño, Adilia Warris, Nicholas J. Wood, Andrew Davidson, Nigel Curtis, the BRACE Trial Consortium Group, Nigel Curtis, Andrew Davidson, Kaya Gardiner, Amanda Gwee, Tenaya Jamieson, Nicole Messina, Thilanka Morawakage, Susan Perlen, Kirsten Perrett, Laure Pittet, Amber Sastry, Jia Wei Teo, Francesca Orsini, Katherine Lee, Cecilia Moore, Suzanna Vidmar, Laure Pittet, Rashida Ali, Ross Dunn, Peta Edler, Grace Gell, Casey Goodall, Richard Hall, Ann Krastev, Nathan La, Ellie McDonald, Nick McPhate, Thao Nguyen, Jack Ren, Luke Stevens, Nicole Messina, Ahmed Alamrousi, Rhian Bonnici, Thanh Dang, Susie Germano, Jenny Hua, Rebecca McElroy, Monica Razmovska, Scott Reddiex, Xiaofang Wang, Jeremy Anderson, Kristy Azzopardi, Vicki Bennett-Wood, Anna Czajko, Nadia Mazarakis, Conor McCafferty, Frances Oppedisano, Belinda Ortika, Casey Pell, Leena Spry, Ryan Toh, Sunitha Velagapudi, Amanda Vlahos, Ashleigh Wee-Hee, Pedro Ramos, Karina De La Cruz, Dinusha Gamage, Anushka Karunanayake, Isabella Mezzetti, Benjamin Ong, Ronita Singh, Enoshini Sooriyarachchi, Suellen Nicholson, Natalie Cain, Rianne Brizuela, Han Huang, Veronica Abruzzo, Morgan Bealing, Patricia Bimboese, Kirsty Bowes, Emma Burrell, Joyce Chan, Jac Cushnahan, Hannah Elborough, Olivia Elkington, Kieran Fahey, Monique Fernandez, Catherine Flynn, Sarah Fowler, Marie Gentile Andrit, Bojana Gladanac, Catherine Hammond, Norine Ma, Sam Macalister, Emmah Milojevic, Jesutofunmi Mojeed, Jill Nguyen, Liz O'Donnell, Nadia Olivier, Isabelle Ooi, Stephanie Reynolds, Lisa Shen, Barb Sherry, Judith Spotswood, Jamie Wedderburn, Angela Younes, Donna Legge, Jason Bell, Jo Cheah, Annie Cobbledick, Kee Lim, Sonja Elia, Lynne Addlem, Anna Bourke, Clare Brophy, Nadine Henare, Narelle Jenkins, Francesca Machingaifa, Skye Miller, Kirsten Mitchell, Sigrid Pitkin, Kate Wall, Paola Villanueva, Nigel Crawford, Laure Pittet, Wendy Norton, Niki Tan, Thilakavathi Chengodu, Diane Dawson, Victoria Gordon, Tony Korman, Jess O'Bryan, Veronica Abruzzo, Sophie Agius, Samantha Bannister, Jess Bucholc, Alison Burns, Beatriz Camesella, John Carlin, Marianna Ciaverella, Maxwell Curtis, Stephanie Firth, Christina Guo, Matthew Hannan, Erin Hill, Sri Joshi, Katherine Lieschke, Megan Mathers, Sasha Odoi, Ashleigh Rak, Chris Richards, Leah Steve, Carolyn Stewart, Eva Sudbury, Helen Thomson, Emma Watts, Fiona Williams, Angela Young, Penny Glenn, Andrew Kaynes, Amandine Philippart De Floy, Sandy Buchanan, Thijs Sondag, Ivy Xie, Harriet Edmund, Bridie Byrne, Tom Keeble, Belle Ngien, Fran Noonan, Michelle Wearing-Smith, Alison Clarke, Pemma Davies, Oliver Eastwood, Alric Ellinghaus, Rachid Ghieh, Zahra Hilton, Emma Jennings, Athina Kakkos, Iris Liang, Katie Nicol, Sally O'Callaghan, Helen Osman, Gowri Rajaram, Sophia Ratcliffe, Victoria Rayner, Ashleigh Salmon, Angela Scheppokat, Aimee Stevens, Rebekah Street, Nicholas Toogood, Nicholas Wood, Twinkle Bahaduri, Therese Baulman, Jennifer Byrne, Candace Carter, Mary Corbett, Aiken Dao, Maria Desylva, Andrew Dunn, Evangeline Gardiner, Rosemary Joyce, Rama Kandasamy, Craig Munns, Lisa Pelayo, Ketaki Sharma, Katrina Sterling, Caitlin Uren, Clinton Colaco, Mark Douglas, Kate Hamilton, Adam Bartlett, Brendan McMullan, Pamela Palasanthiran, Phoebe Williams, Justin Beardsley, Nikki Bergant, Renier Lagunday, Kristen Overton, Jeffrey Post, Yasmeen Al-Hindawi, Sarah Barney, Anthony Byrne, Lee Mead, Marshall Plit, David Lynn, Saoirse Benson, Stephen Blake, Rochelle Botten, Tee Yee Chern, Georgina Eden, Liddy Griffith, Jane James, Miriam Lynn, Angela Markow, Domenic Sacca, Natalie Stevens, Steve Wesselingh, Catriona Doran, Simone Barry, Alice Sawka, Sue Evans, Louise Goodchild, Christine Heath, Meredith Krieg, Helen Marshall, Mark McMillan, Mary Walker, Peter Richmond, Nelly Amenyogbe, Christina Anthony, Annabelle Arnold, Beth Arrowsmith, Rym Ben-Othman, Sharon Clark, Jemma Dunnill, Nat Eiffler, Krist Ewe, Carolyn Finucane, Lorraine Flynn, Camille Gibson, Lucy Hartnell, Elysia Hollams, Heidi Hutton, Lance Jarvis, Jane Jones, Jan Jones, Karen Jones, Jennifer Kent, Tobias Kollmann, Debbie Lalich, Wenna Lee, Rachel Lim, Sonia McAlister, Fiona McDonald, Andrea Meehan, Asma Minhaj, Lisa Montgomery, Melissa O'Donnell, Jaslyn Ong, Joanne Ong, Kimberley Parkin, Glady Perez, Catherine Power, Shadie Rezazadeh, Holly Richmond, Sally Rogers, Nikki Schultz, Margaret Shave, Patrycja Skut, Lisa Stiglmayer, Alexandra Truelove, Ushma Wadia, Rachael Wallace, Justin Waring, Michelle England, Erin Latkovic, Laurens Manning, Susan Herrmann, Michaela Lucas, Marcus Lacerda, Paulo Henrique Andrade, Fabiane Bianca Barbosa, Dayanne Barros, Larissa Brasil, Ana Greyce Capella, Ramon Castro, Erlane Costa, Dilcimar de Souza, Maianne Dias, José Dias, Klenilson Ferreira, Paula Figueiredo, Thamires Freitas, Ana Carolina Furtado, Larissa Gama, Vanessa Godinho, Cintia Gouy, Daniele Hinojosa, Bruno Jardim, Tyane Jardim, Joel Junior, Augustto Lima, Bernardo Maia, Adriana Marins, Kelry Mazurega, Tercilene Medeiros, Rosangela Melo, Marinete Moraes, Elizandra Nascimento, Juliana Neves, Maria Gabriela Oliveira, Thais Oliveira, Ingrid Oliveira, Arthur Otsuka, Rayssa Paes, Handerson Pereira, Gabrielle Pereira, Christiane Prado, Evelyn Queiroz, Laleyska Rodrigues, Bebeto Rodrigues, Vanderson Sampaio, Anna Gabriela Santos, Daniel Santos, Tilza Santos, Evelyn Santos, Ariandra Sartim, Ana Beatriz Silva, Juliana Silva, Emanuelle Silva, Mariana Simão, Caroline Soares, Antonny Sousa, Alexandre Trindade, Fernando Val, Adria Vasconcelos, Heline Vasconcelos, Julio Croda, Carolinne Abreu, Katya Martinez Almeida, Camila Bitencourt de Andrade, Jhenyfer Thalyta Campos Angelo, Ghislaine Gonçalvez de Araújo Arcanjo, Bianca Maria Silva Menezes Arruda, Wellyngthon Espindola Ayala, Adelita Agripina Refosco Barbosa, Felipe Zampieri Vieira Batista, Fabiani de Morais Batista, Miriam de Jesus Costa, Mariana Garcia Croda, Lais Alves da Cruz, Roberta Carolina Pereira Diogo, Rodrigo Cezar Dutra Escobar, Iara Rodrigues Fernandes, Leticia Ramires Figueiredo, Leandro Galdino Cavalcanti Gonçalves, Sarita Lahdo, Joyce dos Santos Lencina, Guilherme Teodoro de Lima, Bruna Tayara LEOPOLDINA MEIRELES, Debora Quadros Moreira, Lilian Batista Silva Muranaka, Adriely de Oliveira, Karla Regina Warszawski de Oliveira, Matheus Vieira de Oliveira, Roberto Dias de Oliveira, Andrea Antonia Souza de Almeida dos Reis Pereira, Marco Puga, Caroliny Veron Ramos, Thaynara Haynara Souza da Rosa, Karla Lopes dos Santos, Claudinalva Ribeiro dos Santos, Dyenyffer Stéffany Leopoldina dos Santos, Karina Marques Santos, Paulo César Pereira da Silva, Paulo Victor Rocha da Silva, Débora dos Santos Silva, Patricia Vieira da Silva, Bruno Freitas da Rosa Soares, Mariana Gazzoni Sperotto, Mariana Mayumi Tadokoro, Daniel Tsuha, Hugo Miguel Ramos Vieira, Margareth Maria Pretti Dalcolmo, Cíntia Maria Lopes Alves da Paixão, Gabriela Corrêa E Castro, Simone Silva Collopy, Renato da Costa Silva, Samyra Almeida da Silveira, Alda Maria Da-Cruz, Alessandra Maria da Silva Passos de Carvalho, Rita de Cássia Batista, Maria Luciana Silva De Freitas, Aline Gerhardt de Oliveira Ferreira, Ana Paula Conceição de Souza, Paola Cerbino Doblas, Ayla Alcoforado da Silva dos Santos, Vanessa Cristine de Moraes dos Santos, Dayane Alves dos Santos Gomes, Anderson Lage Fortunato, Adriano Gomes-Silva, Monique Pinto Gonçalves, Paulo Leandro Garcia Meireless Junior, Estela Martins da Costa Carvalho, Fernando do Couto Motta, Ligia Maria Olivo de Mendonça, Girlene dos Santos Pandine, Rosa Maria Plácido Pereira, Ivan Ramos Maia, Jorge Luiz da Rocha, João Victor Paiva Romano, Glauce dos Santos, Erica Fernandes da Silva, Marilda Agudo Mendonça Teixeira de Siqueira, Ágatha Cristinne Prudêncio Soares, Marc Bonten, Sandra Franch Arroyo, Henny Ophorst-den Besten, Anna Boon, Karin M. Brakke, Axel Janssen, Marijke A.H. Koopmans, Toos Lemmens, Titia Leurink, Cristina Prat-Aymerich, Engelien Septer-Bijleveld, Kimberly Stadhouders, Darren Troeman, Marije van der Waal, Marjoleine van Opdorp, Nicolette van Sluis, Beatrijs Wolters, Jan Kluytmans, Jannie Romme, Wouter van den Bijllaardt, Linda van Mook, M.M.L (Miranda) van Rijen, P.M.G. Filius, Jet Gisolf, Frances Greven, Danique Huijbens, Robert Jan Hassing, R.C. Pon, Lieke Preijers, J.H. van Leusen, Harald Verheij, Wim Boersma, Evelien Brans, Paul Kloeg, Kitty Molenaar-Groot, Nhat Khanh Nguyen, Nienke Paternotte, Anke Rol, Lida Stooper, Helga Dijkstra, Esther Eggenhuizen, Lucas Huijs, Simone Moorlag, Mihai Netea, Eva Pranger, Esther Taks, Jaap ten Oever, Rob ter Heine, Kitty Blauwendraat, Bob Meek, Isil Erkaya, Houda Harbech, Nienke Roescher, Rifka Peeters, Menno te Riele, Carmen Zhou, Esther Calbo, Cristina Badia Marti, Emma Triviño Palomares, Tomás Perez Porcuna, Anabel Barriocanal, Ana Maria Barriocanal, Irma Casas, Jose Dominguez, Maria Esteve, Alicia Lacoma, Irene Latorre, Gemma Molina, Barbara Molina, Antoni Rosell, Sandra Vidal, Lydia Barrera, Natalia Bustos, Ines Portillo Calderón, David Gutierrez Campos, Jose Manuel Carretero, Angel Dominguez Castellano, Renato Compagnone, Encarnacion Ramirez de Arellano, Almudena de la Serna, Maria Dolores del Toro Lopez, Marie-Alix Clement Espindola, Ana Belen Martin Gutierrez, Alvaro Pascual Hernandez, Virginia Palomo Jiménez, Elisa Moreno, Nicolas Navarrete, Teresa Rodriguez Paño, Jesús Rodríguez-Baño, Enriqueta Tristán, Maria Jose Rios Villegas, Atsegiñe Canga Garces, Erika Castro Amo, Raquel Coya Guerrero, Josune Goikoetxea, Leticia Jorge, Cristina Perez, María Carmen Fariñas Álvarez, Manuel Gutierrez Cuadra, Francisco Arnaiz de las Revillas Almajano, Pilar Bohedo Garcia, Teresa Giménez Poderos, Claudia González Rico, Blanca Sanchez, Olga Valero, Noelia Vega, John Campbell, Anna Barnes, Helen Catterick, Tim Cranston, Phoebe Dawe, Emily Fletcher, Liam Fouracre, Alison Gifford, Neil Gow, John Kirkwood, Christopher Martin, Amy McAnew, Marcus Mitchell, Georgina Newman, Abby O'Connell, Jakob Onysk, Lynne Quinn, Shelley Rhodes, Samuel Stone, Lorrie Symons, Harry Tripp, Darcy Watkins, Bethany Whale, Alex Harding, Gemma Lockhart, Kate Sidaway-Lee, John Campbell, Sam Hilton, Sarah Manton, Daniel Webber-Rookes, Rachel Winder, James Moore, Freya Bateman, Michael Gibbons, Bridget Knight, Julie Moss, Sarah Statton, Josephine Studham, Lydia Hall, Will Moyle, Tamsin Venton

**Affiliations:** a Infectious Diseases Group, Murdoch Children's Research Institute, Parkville, Victoria, Australia; b Department of Paediatrics, The University of Melbourne, Parkville, Victoria, Australia; c Infectious Diseases, The Royal Children's Hospital Melbourne, Parkville, Victoria, Australia; d Immunology, Vaccinology, Rheumatology, and Infectious Diseases Unit, Department of Paediatrics, Gynaecology and Obsterics, Faculty of Medicine, University of Geneva and University Hospitals of Geneva, Geneva, Switzerland; e Clinical Epidemiology and Biostatistics Unit, Murdoch Children's Research Institute, Parkville, Victoria, Australia; f Melbourne Children's Trial Centre, Murdoch Children's Research Institute, Parkville, Victoria, Australia; g Precision Medicine Theme, South Australian Health and Medical Research Institute, Adelaide, South Australia, Australia; h Department of Thoracic Medicine, Royal Adelaide Hospital, Adelaide, South Australia, Australia; i Julius Center for Health Sciences and Primary Care, University Medical Centre Utrecht, Utrecht University, the Netherlands; j Exeter Collaboration for Academic Primary Care, University of Exeter Medical School, Exeter, United Kingdom; k Fiocruz Mato Grosso do Sul, Fundação Oswaldo Cruz, Campo Grande, Brazil; l Department of Epidemiology of Microbial Diseases, Yale School of Public Health, New Haven, CT, USA; m Universidade Federal de Mato Grosso do Sul, Campo Grande, Brazil; n Helio Fraga Reference Center, Oswaldo Cruz Foundation Ministry of Health, Curicica, Brazil; o Catholic University, Rio de Janeiro, Brazil; p Research Operations, The Royal Children's Hospital Melbourne, Parkville, Victoria, Australia; q Institute of Clinical Research Carlos Borborema, Doctor Heitor Vieira Dourado Tropical Medicine Foundation, Manaus, Brazil; r Instituto Leônidas & Maria Deane, Oswaldo Cruz Foundation Ministry of Health, Manaus, Brazil; s University of Texas Medical Branch, Galveston, TX, USA; t Department of Immunology, Pathwest, Queen Elizabeth II Medical Centre, Nedlands, Western Australia, Australia; u Department of Immunology, Sir Charles Gairdner Hospital, Nedlands, Western Australia, Australia; v Department of Immunology and General Paediatrics, Perth Children's Hospital, Nedlands, Western Australia, Australia; w School of Medicine, University of Western Australia, Perth, Western Australia, Australia; x Flinders Health and Medical Research Institute, Flinders University, Bedford Park, South Australia, Australia; y Wesfarmers Centre for Vaccines and Infectious Diseases, Telethon Kids Institute, Nedlands, Western Australia, Australia; z Department of Infectious Diseases, Fiona Stanley Hospital, Murdoch, Western Australia, Australia; aa Department of Allergy and Immunology, Royal Children's Hospital Melbourne, Parkville, Victoria, Australia; ab Department of Infectious Diseases, Prince of Wales Hospital, Randwick, New South Wales, Australia; ac School of Clinical Medicine, University of New South Wales, Sydney, New South Wales, Australia; ad Institut d'Investigació Germans Trias i Pujol, Departament de Genètica i Microbiologia, Universitat Autònoma de Barcelona, Centro de Investigación Biomédica en Red (CIBER) de Enfermedades Respiratorias, Instituto de Salud Carlos III, Barcelona, Spain; ae Division of Infectious Diseases and Microbiology, Department of Medicine, Hospital Universitario Virgen Macarena, University of Seville, Biomedicines Institute of Seville-Consejo Superior de Investigaciones Científicas, Seville, Spain; af CIBER de Enfermedades Infecciosas, Instituto de Salud Carloss III, Madrid, Spain; ag Medical Research Council Centre for Medical Mycology, University of Exeter, Exeter, United Kingdom; ah Faculty of Medicine and Health, University of Sydney, Sydney, New South Wales, Australia; ai Sydney Children's Hospital Network, Westmead, New South Wales, Australia; aj National Centre for Immunisation Research and Surveillance of Vaccine Preventable Disease, Westmead, New South Wales, Australia

**Keywords:** Bacille Calmette-Guérin (BCG) vaccine, Immunity, Heterologous, Health personnel, Randomised controlled trial, Primary prevention, Placebo

## Abstract

**Background:**

Bacille Calmette-Guérin (BCG) vaccination has off-target (non-specific) effects that are associated with protection against unrelated infections and decreased all-cause mortality in infants. We aimed to determine whether BCG vaccination prevents febrile and respiratory infections in adults.

**Methods:**

This randomised controlled phase 3 trial was done in 36 healthcare centres in Australia, Brazil, the Netherlands, Spain, and the United Kingdom. Healthcare workers were randomised to receive BCG-Denmark (single 0.1 ml intradermal injection) or no BCG in a 1:1 ratio using a web-based procedure, stratified by stage, site, age, and presence of co-morbidity. The difference in occurrence of febrile or respiratory illness were measured over 12 months (prespecified secondary outcome) using the intention-to-treat (ITT) population. This trial is registered with ClinicalTrials.gov, NCT04327206.

**Findings:**

Between March 30, 2020, and April 1, 2021, 6828 healthcare workers were randomised to BCG-Denmark (n = 3417) or control (n = 3411; no intervention or placebo) groups. The 12-month adjusted estimated risk of ≥1 episode of febrile or respiratory illness was 66.8% in the BCG group (95% CI 65.3%–68.2%), compared with 63.4% in the control group (95% CI 61.8%–65.0%), a difference of +3.4 percentage points (95% CI +1.3% to +5.5%; p 0.002). The adjusted estimated risk of a severe episode (defined as being incapacitated for ≥3 consecutive days or hospitalised) was 19.4% in the BCG group (95% CI 18.0%–20.7%), compared with 18.8% in the control group (95% CI 17.4%–20.2%) a difference of +0.6 percentage points (95% CI −1.3% to +2.5%; p 0.6). Both groups had a similar number of episodes of illness, pneumonia, and hospitalisation. There were three deaths, all in the control group. There were no safety concerns following BCG vaccination.

**Interpretation:**

In contrast to the beneficial off-target effects reported following neonatal BCG in infants, a small increased risk of symptomatic febrile or respiratory illness was observed in the 12 months following BCG vaccination in adults. There was no evidence of a difference in the risk of severe disease.

**Funding:**

10.13039/100000865Bill & Melinda Gates Foundation, 10.13039/501100016056Minderoo Foundation, Sarah and Lachlan Murdoch, the 10.13039/100014607Royal Children's Hospital Foundation, Health Services Union NSW, the 10.13039/501100014093Peter Sowerby Foundation, SA Health, the Insurance Advisernet Foundation, the NAB Foundation, the Calvert-Jones Foundation, the Modara Pines Charitable Foundation, the UHG Foundation Pty Ltd, Epworth Healthcare, the 10.13039/501100000925National Health and Medical Research Council, the 10.13039/100000001Swiss National Science Foundation and individual donors.

**Research in context**

**Evidence before this study**

A previous systematic review of randomised controlled trials (RCT) showed that neonatal bacille Calmette-Guérin (BCG) protects against unrelated infections and reduces all-cause mortality in infants living in high-mortality settings; preliminary results from several RCTs in adults suggest that BCG vaccination offers some protection against unrelated pathogens, including respiratory infections. Early in the COVID-19 pandemic, it was proposed that the immunomodulatory off-target effects of BCG vaccine could be exploited to reduce the impact of COVID-19; it was hoped they could provide partial protection against SARS-CoV-2 until effective COVID-19-specific vaccines became available. However, in recent RCTs, BCG vaccine did not protect adults against COVID-19 over 3–15 months of follow up.

**Added value of this study**

BCG vaccination increased, rather than decreased, the risk of subsequent non-severe symptomatic febrile or respiratory illness in adults in the 12 months post randomisation. This increase was relatively small (5% increase, +3.4 percentage points) and was less evident for severe disease. There was no increase in all-cause mortality, as the three deaths were all in the control group.

**Implications of all the available evidence**

BCG vaccination did not prevent symptomatic febrile or respiratory infection in adults in the 12 months following randomisation. Future studies should focus on the explanation for the observed setting-related and possibly age-related discrepancy in the off-target effects of BCG vaccination, as our results contrast with the considerable beneficial off-target effects observed on unrelated infections and all-cause mortality following neonatal BCG vaccination in infants in high-mortality settings.

## Introduction

The COVID-19 pandemic has focused attention on bacille Calmette-Guérin (BCG) vaccine and its potential to prevent respiratory infection through off-target (non-specific) effects. This 100-years-old vaccine, used to prevent tuberculosis, is associated with a decrease in all-cause mortality in infants in high-mortality settings,^1^ and possible protection against respiratory infections other than those caused by *Mycobacterium tuberculosis*.^2^, ^3^, ^4^ It is postulated that BCG vaccination induces changes in immune cells that lead to more efficient responses to subsequent infections (‘trained immunity’), irrespective of the pathogen.^5^^,^^6^

We previously reported that, in the BRACE (*BCG vaccination to reduce the impact of COVID-19 in healthcare workers*) randomised controlled trial (RCT),^7^ BCG vaccination, compared with placebo vaccination, did not reduce the incidence of COVID-19 in the 6 months following randomisation.^8^ Here we report on the prespecified secondary outcome of protection against any febrile or respiratory illnesses in the 12 months following randomisation.

## Methods

### Study design

BRACE is a phase III multicentre RCT involving healthcare workers from 36 sites in Australia, Brazil, the Netherlands, Spain, and the United Kingdom, who were randomised between March 2020 and April 2021 in a 1:1 ratio to the BCG group or the control group, and who were followed up for 12 months. The trial protocol and primary outcomes have been published,^7^^,^^8^ and is registered on ClinicalTrials.gov (NCT04327206; registered in March 2020 before enrolling the first participant). The trial comprised two stages: stage 1 (recruitment in Australia between March and May 2020; open label) and stage 2 (recruitment in Australia, Brazil and Europe between May 2020 and April 2021; triple blind). The transition from stage 1 to stage 2, made possible by further funding, aimed to enhance the trial's robustness and adapt to the dynamic circulation of SARS-CoV-2 during the pandemic. As SARS-CoV-2 exposure was virtually negligible during stage 1 in Australia, these participants were excluded from the previously published COVID-19-related outcomes at 6 months.^7^^,^^8^

The study was approved by the Royal Children's Hospital Melbourne Human Research Ethics Committee (No. 62586); the protocol was approved by the ethics committee at each site and all participants provided informed consent. The trial was overseen by a steering committee and a data safety and monitoring board. This report follows CONSORT reporting guidelines.

### Participants

All healthcare workers from participating institutions were invited to have their eligibility ascertained during a baseline visit. Exclusion criteria included: involvement in another COVID-19 prevention trial, previous positive test for SARS-CoV-2 or receipt of any COVID-specific vaccine, contraindication to BCG vaccination, receipt of BCG vaccine within the last year or of a live-attenuated vaccine within the last month. All participants provided written informed consent and answered a baseline questionnaire collecting demographic and health data, including self-reported sex data.

### Randomisation and masking

Randomisation was done using a web-based procedure (REDCap®),^9^ in randomly permuted blocks of variable length (2, 4, or 6), stratified by stage, site, age and presence of comorbidity. Investigators, statisticians, and trial staff were blinded to the randomisation group throughout the trial.

### Procedure

BCG-Denmark (AJ Vaccines, Copenhagen) was given to participants randomised to the BCG group as a single 0.1 ml intradermal injection in the region of the deltoid muscle at the inclusion visit, corresponding to 2–8 x 10^5^ colony forming units of *Mycobacterium bovis* Danish strain 1331.

Participants randomised to the control group received no intervention in stage 1, and a placebo saline 0.1 ml intradermal injection in stage 2.

Stage 1 participants were required to receive influenza vaccination on the day of randomisation, regardless of group allocation, as they were recruited during the Australian influenza season.

Participants were asked weekly if they had been unwell using a smartphone application designed for the trial (Trial Symptom Tracker, WeGuide) and/or by direct contact. Symptom reports were collected daily during each episode of illness and included the following: fever, cough, shortness of breath/difficulty breathing, sore throat, runny/blocked nose, fatigue, muscle or joint pain, headache, nausea or vomiting, diarrhoea, and loss of smell or taste. To evaluate the severity of the episode, participants reported daily whether they felt too unwell to work, were confined to bed and/or were hospitalised. Accuracy and completeness of the data collected was ascertained 3-monthly by participants using customised web-based questionnaires that summarised individual data collected. Additional information on hospitalisation was obtained from medical records.

### Outcomes

A febrile or respiratory illness was defined as an episode lasting at least one day with at least one of the following symptoms: fever, cough, shortness of breath, difficulty breathing and/or sore throat. The illness was categorised as severe if the participant was unable to work or confined to bed for three or more consecutive days, was hospitalised, or died, as a result of the febrile or respiratory illness.

Additional prespecified secondary outcomes over 12 months included: number of episodes of fever or respiratory illness, number of days with symptoms due to fever or respiratory illness, number of days unable to work due to fever or respiratory illness, number of days confined to bed due to fever or respiratory illness, occurrence of pneumonia as a consequence to fever or respiratory illness, occurrence of hospitalisation as a consequence to fever or respiratory illness, need for oxygen therapy as a consequence to fever or respiratory illness, admission to critical care as a consequence to fever or respiratory illness, need for mechanical ventilation as a consequence to fever or respiratory illness, occurrence of death as a consequence to fever or respiratory illness and number of days of unplanned absenteeism for an acute illness or hospitalisation.

### Statistical analysis

The statistical analysis plan was published before unblinding.^10^ Analyses were done using Stata v17 (StataCorp, College Station, TX). Occurrence of febrile or respiratory illness (any and severe) were compared between the two groups using difference in proportions, estimated using a time-to-event analysis, and adjusted for stratification factors used for randomisation, namely stage/site (Australia stage 1; Australia stage 2; Europe; South America), age (<40; 40–59; ≥60 years-old), and comorbidity (presence; absence). To do this analysis, the survival curve for each combination of strata and randomised group was calculated using a flexible parametric survival model (Royston-Parmar model) using 3 degrees of freedom, which represented a good balance between capturing the complexity of the baseline hazard function and avoiding overfitting. The coefficients included in the model were data collected at baseline and did not vary over time. As modelling was used to estimate proportions as well as the difference in proportion, the results from this analysis do not correspond directly to the raw summaries presented. Participants were censored at 12 months or at the first instance at which it could not be ascertained whether an episode had occurred (defined as three or more consecutive days of missing data). Sensitivity analyses were done using a hypothetical strategy censoring participants at the intercurrent event of receiving any subsequent vaccine. Hazard ratios (HR) were calculated for rarer outcomes (admission to critical care, mechanical ventilation). Incidence rate ratios (IRR) were used to compare the number of episodes, the number of days with symptoms, the number of days unable to work, the number of days confined to bed, and the number of days of unplanned absenteeism between the two groups. Analyses were done using the intention-to-treat (ITT) population, including all eligible participants who undertook randomisation.

Predefined subgroup analyses were done when there was a significant interaction between the treatment arm and one of the stratification factors.

### Role of the funding source

The funders had no role in study design, data collection, data analysis, data interpretation, or writing of the report. LFP and FO have directly accessed and verified the underlying data reported in the manuscript. All authors had final responsibility for the decision to submit for publication.

## Results

**Fig. 1:**
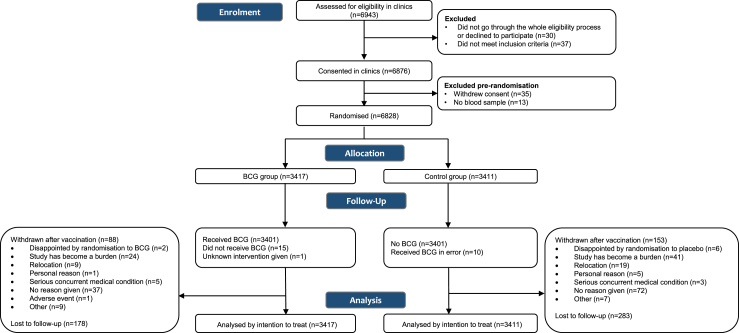
**Consort diagram**. Consort diagram of screening, randomisation and follow-up. BCG denotes bacille Calmette-Guérin.

Of 6943 individuals screened, 6828 were randomised (3417 to BCG group, 3411 to control) between March 30, 2020 and April 1, 2021 (Fig. 1). Baseline participant characteristics were similar between treatment groups (Table 1). Participants had a mean age of 42.0 years (SD 12.0) at inclusion and 18% reported a comorbidity.

**Table 1:** Participants characteristics at baseline, intention-to-treat population.

	BCG	Control
Participants	3417	3411
Sex, female	2511/3417 (73.5%)	2593/3411 (76.0%)
Age, years (mean, SD, range)	42.0 (12.1), 18.4 to 78.7	42.0 (12.0), 18.1 to 83.5
<40 years old	1592/3417 (46.6%)	1594/3411 (46.7%)
40–59 years old	1566/3417 (45.8%)	1557/3411 (45.7%)
≥60 years old	259/3417 (7.6%)	260/3411 (7.6%)
Presence of comorbidities	613/3417 (17.9%)	625/3411 (18.3%)
Participant with 1 comorbidity	552/613 (90.0%)	577/625 (92.3%)
Participant with 2 comorbidities	60/613 (9.8%)	45/625 (7.2%)
Participant with 3 comorbidities	1/613 (0.2%)	3/625 (0.5%)
Cardiovascular disease	354/3417 (10.4%)	364/3411 (10.7%)
Chronic respiratory disease	230/3417 (6.7%)	208/3411 (6.1%)
Diabetes	89/3417 (2.6%)	104/3411 (3.0%)
BCG vaccination in the past	2264/3417 (66.3%)	2246/3410 (66.0%)
Last BCG 1–5 years prior to inclusion	53/2264 (2.3%)	49/2246 (2.2%)
Last BCG >5 years prior to inclusion	2211/2264 (97.7%)	2197/2246 (97.8%)
Previous known tuberculosis exposure	29/3417 (0.9%)	24/3410 (0.7%)
Previous positive tuberculin skin test (>5 mm)	205/3417 (6.0%)	224/3410 (6.6%)
Geographical location		
Australia	1634/3417 (47.8%)	1628/3411 (47.7%)
Europe	498/3417 (14.6%)	500/3411 (14.7%)
South America	1285/3417 (37.6%)	1283/3411 (37.6%)
Study stage		
Stage 1	1418/3417 (41.50%)	1422/3411 (41.69%)
Stage 2	1999/3417 (58.50%)	1989/3411 (58.31%)

SD, standard deviation.

**Fig. 2:**
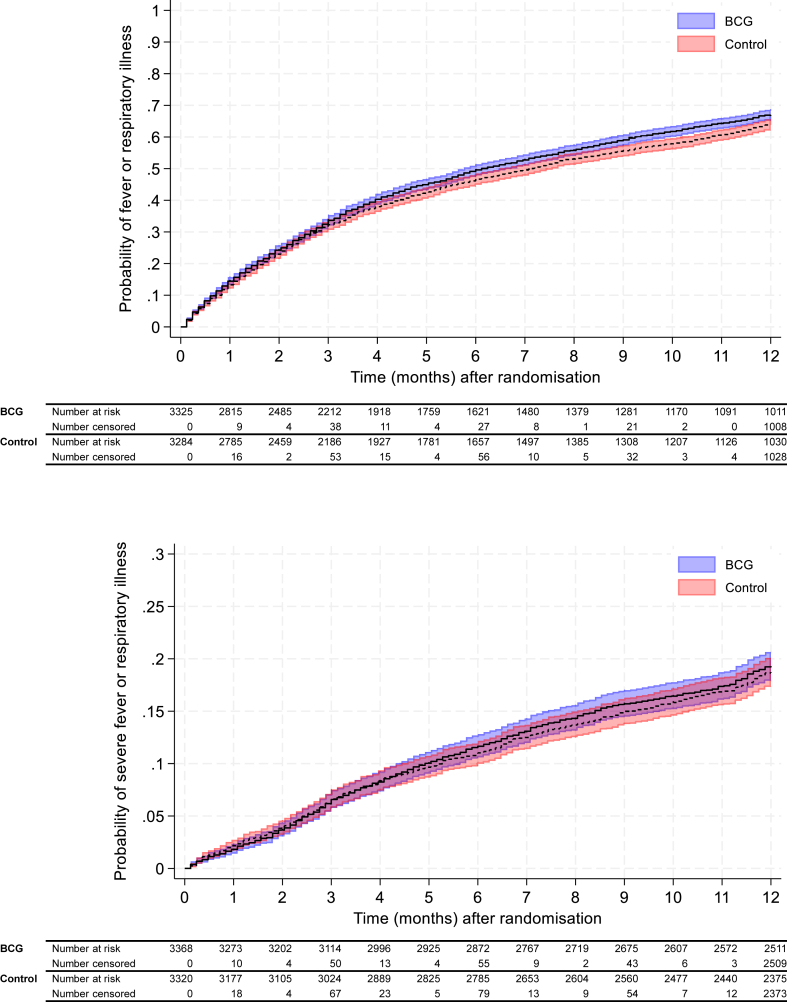
**Fever and respiratory illness by 12 months following randomisation, by treatment arm**. Time to first symptomatic febrile or respiratory illness (top panel) or first severe symptomatic febrile or respiratory illness (bottom panel) are shown with Kaplan–Meier curves and 95% confidence intervals. BCG denotes Bacille Calmette-Guérin. BCG group is shown in blue and control group in red.

During the 12 months of follow up, 4243 participants reported one or more episodes of febrile or respiratory illness, 2214 in the BCG group (adjusted estimated risk 66.8%; 95% CI 65.3%–68.2%), compared with 2029 in the control group (adjusted estimated risk 63.4%; 95% CI 61.8%–65.0%), a difference of +3.4 percentage points (95% CI +1.3% to +5.5%; p 0.002; Fig. 2). Severe episodes of febrile or respiratory illness, as defined by the trial, occurred in 637 participants in the BCG group (adjusted estimated risk 19.4%; 95% CI 18.0%–20.7%), compared with 588 in the control group (adjusted estimated risk 18.8%; 95% CI 17.4%–20.2%), a difference of +0.6 percentage point (95% CI −1.3% to +2.5%; p 0.6; Fig. 2).

When the number of days with symptoms was compared between the two groups, there was evidence of an interaction between the trial group and two randomisation factors (comorbidity and geographical location), which rendered an overall comparison between randomisation groups noninterpretable. Subsequent subgroup analyses showed that participants without a comorbidity in the BCG group reported being ill for fewer days, compared to those in the control group (IRR 0.91, 95% CI 0.86–0.96; p 0.001), whilst no effect was shown in participants with comorbidities (IRR 1.1, 95% CI 0.96–1.30; p 0.2). Regarding the interaction with study stage and geographical region, participants in Australia during stage 1 in the BCG group reported being ill for fewer days, compared to those in the control group (IRR 0.85, 95% CI 0.78–0.94; p 0.001), whereas no substantial intervention effect was seen between trial groups amongst participants in stage 2 and the other geographical strata (Table 2).

**Table 2:** Fever or respiratory illness by 12 months following randomisation.

	BCG	Control	Difference	p-value
	N = 3417	N = 3411	BCG/control	
**Febrile or respiratory illness by 12 months**	**2214**	**2029**		
Person years	1776	1783		
Event rate (per 100 person years)	122 (116; 127)	112 (107; 117)		
Adjusted estimated percent, with 95% CI^a^	66.8% (65.3; 68.2)	63.4% (61.8; 65.0)	+3.4% (+1.3; +5.5)	0.002
**Severe febrile or respiratory illness by 12 months**	**637**	**588**		
Person years	2885	2778		
Event rate (per 100 person years)	22 (20; 23)	21 (19; 23)		
Adjusted estimated percent, with 95% CI^a^	19.4% (18.0; 20.7)	18.8% (17.4; 20.2)	+0.6% (−1.3; +2.5)	0.6
**Secondary outcomes by 12 months**				
Pneumonia^a^^,^^b^	22 (0.7%)	21 (0.6%)	+0.0% (−0.4; +0.4)	0.9
Hospitalisation^a^^,^^b^	27 (0.8%)	20 (0.6%)	+0.2% (−0.2; +0.6)	0.4
Oxygen therapy^b^	12 (0.4%)	6 (0.2%)	+0.2% (−0.1; +0.4)	0.2
Admission to critical care^b^	5 (0.2%)	3 (0.1%)	aHR: 1.66 (0.40; 6.98)	0.5
Mechanical ventilation^b^	2 (0.1%)	1 (<0.1%)	aHR: 1.98 (0.18; 21.85)	0.6
Death^b^	0	3 (0.1%)	–	–
Number of days confined to bed^a^^,^^c^	0 (0–0), min 0 max 116	0 (0–0), min 0 max 20	aIRR: 0.95 (0.79; 1.14)	0.6
Number of episodes^a^^,^^c^	2.0 (1.0–3.0)	2.0 (1.0–3.0)	aIRR: 0.98 (0.94; 1.03)	0.4
**Post-hoc subgroup analysis**				
**Number of days with symptoms**^e^				
Comorbidity randomisation strata				0.004^f^
Presence of any comorbidity^a^^,^^c^	13.0 (6.0–25.0)	11.0 (5.0–22.0)	aIRR: 1.10 (0.96–1.26)	
Absence of any comorbidity^a^^,^^c^	11.0 (5.0–20.0)	11.0 (6.0–22.0)	aIRR: 0.91 (0.86–0.96)	
Region strata				0.009^f^
Australia stage 1^a^^,^^c^	8.0 (4.0–15.0)	9.0 (4.0–16.0)	aIRR: 0.85 (0.78–0.94)	
Australia stage 2^a^^,^^c^	8.0 (4.0–16.0)	7.5 (4.0–18.0)	aIRR: 0.92 (0.74–1.15)	
Europe^a^^,^^c^	11.0 (5.0–21.0)	10.0 (5.0–20.0)	aIRR: 1.11 (0.95–1.30)	
South America^a^^,^^c^	15.0 (8.0–27.0)	14.0 (8.0–28.0)	aIRR: 0.96 (0.89–1.04)	
**Number of days unable to work**^e^				
Age group randomisation strata				0.05^f^
<40 years-old^a^^,^^c^	1.0 (0.0–3.0)	1.0 (0.0–4.0)	aIRR: 0.99 (0.84–1.17)	
40–59 years-old^a^^,^^c^	1.0 (0.0–4.0)	0.0 (0.0–4.0)	aIRR: 1.14 (0.93–1.39)	
≥60 years-old^a^^,^^c^	0.0 (0.0–3.0)	1.0 (0.0–3.0)	aIRR: 0.59 (0.35–1.01)	
Comorbidity randomisation strata				0.05^f^
Presence of any comorbidity^a^^,^^c^	1.0 (0.0–4.0)	0.0 (0.0–4.0)	aIRR: 1.25 (0.93–1.69)	
Absence of any comorbidity^c^	1.0 (0.0–4.0)	1.0 (0.0–4.0)	aIRR: 0.94 (0.82–1.08)	
**Number of days of unplanned absenteeism**^e^				
Comorbidity randomisation strata				0.004^f^
Presence of any comorbidity^a^^,^^c^	6.0 (2.0–14.0)	6.0 (3.0–11.0)	aIRR: 1.15 (0.96–1.38)	
Absence of any comorbidity^a^^,^^c^	5.0 (2.0–10.0)	5.0 (2.0–10.0)	aIRR: 0.91 (0.84–0.99)	
Region strata				0.01^f^
Australia stage 1^a^^,^^c^	3.0 (2.0–6.0)	4.0 (2.0–7.5)	aIRR: 0.84 (0.75–0.94)	
Australia stage 2^a^^,^^c^	3.0 (2.0–7.0)	3.0 (2.0–6.0)	aIRR: 1.03 (0.78–1.37)	
Europe^a^^,^^c^	4.5 (2.0–12.0)	3.0 (2.0–10.0)	aIRR: 1.17 (0.87–1.56)	
South America^a^^,^^c^	10.0 (5.0–14.0)	10.0 (5.0–14.0)	aIRR: 1.02 (0.93–1.12)	

aHR, adjusted hazard ratio^a^; aIRR, adjusted incidence rate ratio.^a^

a Adjusted for stratification factors.

b Reported as n (%).

c Within participant with one or more episode of febrile or respiratory illness, reported as median (interquartile range).

e The number of days with symptoms, the number of days unable to work, and the number of unplanned absenteeism are only presented by subgroups, due to a significant interaction between treatment arm and stratification factors, which rendered the main arm comparison non-interpretable. Denominators available in Supplementary Appendix (Table S1).

f P-value for interaction between arm and subgroup.

Both groups had a similar number of episodes of illness (median 2, IQR 1–3 episodes) and number of days confined to bed (median 0, IQR 0–0 days). Regarding the number of days unable to work, there was evidence of an interaction between trial group and two randomisation strata (age group, and comorbidity) as detailed in Table 2. Similarly, for the number of days of unplanned absenteeism, there was evidence of an interaction between the trial group and two randomisation strata (comorbidity and geographical localisation). Subgroup analyses showed that participants without a comorbidity in the BCG group reported being absent for fewer days, compared to those in the control group (IRR 0.91, 95% CI 0.84–0.99; p 0.02), which was not the case in participants with comorbidities (IRR 1.15, 95% CI 0.96–1.38; p 0.1). Regarding the interaction with study stage and geographical region, the difference was observed among stage 1 participants living in Australia, with participants in the BCG group reporting being absent for fewer days, compared to those in the control group (IRR 0.84, 95% CI 0.75–0.94; p 0.003). No substantial difference between trial groups was seen among participants in stage 2 and the other geographical strata (Table 2).

There was no evidence of a difference in the number of participants with pneumonia (22/3417 in the BCG group vs 21/3411 in the control group), who were hospitalised (27/3417 vs 20/3411), required supplemental oxygen therapy (12/3417 vs 6/3411), were admitted to critical care unit (5/3417 vs 3/3411), required mechanical ventilation (2/3417 vs 1/3411), or who died (0/3417 vs 3/3411). Causes of death were COVID-19 (2 participants) and unspecified pneumonia (1 participant).

Sensitivity analyses using the hypothetical strategy showed similar results (see statistical report in the Supplementary material). As previously reported, there were no safety concerns following BCG vaccination in the BRACE trial.^8^^,^^11^

## Discussion

In this large randomised controlled trial of nearly 7000 healthcare workers, we found that BCG vaccination was associated with an increased risk of febrile or respiratory illness in the 12 months following randomisation. This increase was relatively small (5.4% increase, +3.4 percentage points) and not observed for severe disease, and notably, the three deaths were all in the control group.

These findings contrast with previous trials in infants in high-mortality settings which suggest that prevention of unrelated respiratory infections and sepsis underlies the reduction in all-cause mortality observed following neonatal BCG vaccination.^1^^,^^12^^,^^13^ Although two subsequent RCTs in low-mortality settings (Denmark and Australia) did not find a significant overall beneficial effect of neonatal BCG vaccination on the prevention of unrelated infections,^14^^,^^15^ the trial done in Denmark reported a reduction in hospital admissions in BCG-vaccinated infants born to BCG-vaccinated mothers compared with controls.^14^ BCG-induced protection against unrelated respiratory infections has also been observed in other age groups. In a sub-analysis of adverse events in an RCT involving 990 adolescents in South Africa, the rate of upper respiratory tract infections was reduced in BCG-Denmark-vaccinated adolescents compared with placebo vaccination (2.1% vs 7.9%; p < 0.001), although the follow-up period was relatively short.^4^ In another RCT, involving 198 elderly patients receiving BCG-Denmark or placebo vaccine at hospital discharge in Greece, the incidence of respiratory tract infections was also lower in the BCG group compared with placebo (4.2%; vs 17.9%; p 0.01).^3^ In an RCT in 34 elderly patients in Indonesia, three monthly doses of BCG-Pasteur was reported to reduce respiratory infections.^2^

During the COVID-19 pandemic, a number of trials worldwide evaluated whether BCG vaccine's off-target effects could be exploited to protect high-risk individuals before COVID-19-specific vaccines were developed (recently summarised in Noble et al.^16^). In brief, among the 11 trials published to date, most failed to find a protective effect of BCG vaccination against COVID-19, except for one, in an ongoing trial investigating the influence of repeated doses of BCG-Japan on glycaemic control in type 1 diabetic patients (1/96 case of COVID-19 in the BCG group vs 6/48 cases in the control group; p 0.006).^17^ Although the results from the 11 trials were inconsistent and mostly underpowered, three of the largest trials, including the BRACE trial,^8^ suggest that BCG vaccination increases the risk of COVID-19.^16^^,^^18^

The increased risk of febrile or respiratory illness observed in the BCG group could be explained by an enhancement of the immune response following vaccination. Studies suggest that BCG vaccine trains the innate immune system by inducing epigenetic and functional reprogramming, making it more effective at clearing a pathogen at subsequent encounters.^5^^,^^6^ A previous study using yellow fever vaccine in a human challenge model reported reduced yellow fever virus viraemia in the BCG group compared with the placebo group, together with evidence of trained immunity.^19^ In a malaria human challenge model, evaluating whether BCG-Bulgaria alters the clinical and immunological responses, BCG-vaccinated volunteers had earlier expression of NK-cell activation markers, which was associated with lower parasitaemia, but with an earlier and more severe clinical response, compared with controls.^20^

In participants from the BRACE trial, we reported a stronger T-cell response to *in vitro* SARS-CoV-2 stimulation in BCG-vaccinated participants compared with controls.^21^ The increased risk of illness in the BCG group in the present study might also be explained by fewer asymptomatic infections. The enhanced immune response could be associated with more rapid clearance of pathogens, leading to shorter illnesses. There was some evidence for this in our trial, with some subgroup analyses suggesting milder illness in the BCG group compared with the control group, including in the elderly. The latter finding is in line with some previous reports which suggest a beneficial effect of BCG in the elderly,^3^^,^^22^ but in contrast with a large recent study that failed to demonstrate a beneficial effect of BCG to prevent COVID-19 in elderly individuals with or without comorbidities.^23^ In addition, the absence of effect of BCG in the subgroup of participants with comorbidities in the present study is in contrast with previous findings suggesting a beneficial effect of BCG vaccination in patients with comorbidities.^17^^,^^24^

The discrepancies observed amongst BCG trials might be attributable to differences in setting (mortality rates, mycobacterial exposure, (epi)genetic differences in population), age group (adults vs infants, adolescent, or elderly), BCG dose regime (1 vs 3 doses in some trials) or strain, definition of respiratory infection (any vs upper or lower), pathogen (SARS-CoV-2 vs undetermined) and/or duration of follow-up (12 months in BRACE vs 7 days to 12 months in other studies).^16^ Nevertheless, reported all-cause mortality seems to be lower in the BCG group, in both adults^25^ and in infants.^1^

The strength of our trial is its size, being among the largest to report the effect of BCG vaccination on febrile or respiratory illness in adults. The main limitation of our trial is the self-reporting of illness without microbiological confirmation, precluding sub-analysis by pathogen. Another is the inevitable inability to ensure complete blinding in trials using BCG due to formation of injection site scars in most BCG recipients.^26^ The retention rate was higher in the BCG group compared with the control group, but the person years used for the analyses were comparable between groups. Participants were exclusively healthcare workers, who may differ from the general population in terms of microbial (and mycobacterial) exposure. Finally, lock downs and the use of masks, social distancing and hand hygiene during the COVID-19 pandemic had an impact on the circulation of common respiratory pathogens. Our results are therefore not necessarily generalisable to periods with higher circulation of RSV, influenza, and rhinovirus.

In conclusion, our study is the first to suggest that BCG vaccination slightly increases rather than decreases the risk of subsequent non-severe symptomatic febrile or respiratory illness in adults. Future studies should focus on determining the factors that explain the difference in the effect to BCG in this setting compared with the beneficial effects on infections and all-cause mortality observed in neonates living in high-mortality settings.^1^^,^^16^^,^^27^

## Contributors

All authors contributed substantially to the BRACE trial. NC is the chief principal investigator of the BRACE trial. FO wrote the statistical analysis plan with input from LFP, EMD, NLM, NC, and did all the statistical analysis. LFP wrote the first draft of the report with input from NC, and all authors critically reviewed it. All authors had final responsibility for the decision to submit for publication. LFP and FO have directly accessed and verified the underlying data reported in the manuscript. NC attests that all listed authors meet authorship criteria and that no others meeting the criteria have been omitted.

## Data sharing statement

Deidentified participant data and data dictionary are available to others on request and on completion of a signed data access agreement. Requests can be made in writing to braceresearch@mcri.edu.au.

## Declaration of interests

All authors have completed the ICMJE uniform disclosure form at http://www.icmje.org/disclosure-of-interest/and declare: the trial is financially supported by the Foundations listed in the Funding section. Authors disclose funding support over the past 36 months: National Health and Medical Research Council (NHMRC) Ideas Grant (NM), Investigator Grant (NC); Melbourne Children's Clinician-Scientist Fellowship Grant (KPP); Institutional support for research grants, presentations, meeting attendance and participation on Scientific Advisory Boards related to pertussis, RSV, pneumococcal, meningococcal and COVID-19 diseases from GlaxoSmithKline, Merck Sharpe & Dohme, Pfizer, Clover Biopharmaceuticals and Resvinet Foundation (PCR); grant support from Sanofi, MSD & CEPI for COVID-19 vaccine/drug research (JC).

PCR has participated on Astra Zeneca COVID-19 Scientific Advisory Board and has a bacteriotherapy patent pending. JC participates on Latin American data safety monitoring/advisory boards for mRNA-1273 (Modern/Zodiac), RSV maternal vaccine (Pfizer), Qdenga vaccine (Takeda), Nirmatrelvir/Ritonavir-Paxlovid (Pfizer) and recently presented to Foro Latinoamericano para Asesores Médicos en Vacunas (Pfizer). NW has participated in the Covalia COVID-19 DNA vaccine trial in an advisory/data safety monitoring board capacity. KG is a member of the Royal Children's Hospital (RCH) Human Research Ethics Committee (the primary ethics committee providing approval for the BRACE trial) and Director of Research Operations at RCH; she abstained from all discussion, voting, approval and review related to the BRACE trial.

## Appendix A Supplementary data

Supplement.pdf

BRACE Consortium eClinicalMedicine
