# Supplementary material for: Bacille Calmette-Guérin vaccination to prevent febrile and respiratory illness in adults (BRACE): secondary outcomes of a randomised controlled phase 3 trial
Source: eClinicalMedicine. 2024 May 13;72:102616. doi: 10.1016/j.eclinm.2024.102616 (PMC11106519; doi:10.1016/j.eclinm.2024.102616)
Supplement: Supplement.pdf [file mmc1.pdf]

Supplement

This supplement contains the following items:

1. (a) Original protocol,
   (b) Final protocol,
   (c) Summary of changes.

2. (a) Original statistical analysis plan,
   (b) Final statistical analysis plan, including (c) Summary of changes

PROTOCOL

HREC/protocol no: 62586

Exploiting the Beneficial Off-Target Effects of BCG Vaccination to Protect Health Care Workers Against COVID-19

Version 1, 15th March 2020

Document history:

Version Number and Date	Summary of changes
v1 15th March 2020	Initial release

CONFIDENTIAL

This protocol is confidential and is the property of Murdoch Children's Research Institute. No part of it may be transmitted, reproduced, published, or used without prior written authorisation from the institution.

Statement of Compliance

This clinical trial will be conducted in compliance with all stipulation of this protocol, the conditions of the ethics committee approval, the NHMRC National Statement on ethical Conduct in Human Research (2007 and all updates), the Integrated Addendum to ICH E6 (R1): Guideline for Good Clinical Practice E6 (R2), dated 9 November 2016 annotated with TGA comments and the NHMRC guidance Safety monitoring and reporting in clinical trials involving therapeutic goods (EH59, 2016).

This clinical trial is not sponsored by any pharmaceutical company or other commercial entity.

CONTENTS

PROTOCOL SYNOPSIS 6
GLOSSARY OF ABBREVIATIONS 9
INVESTIGATOR AGREEMENT 10
1. ADMINISTRATIVE INFORMATION 11
1.1. Trial registration 11
1.1.1. Trial registry .... Error! Bookmark not defined.
1.2. Sponsor 11
1.3. Expected duration of study 11
1.4. Contributorship 11
1.5. Stakeholder involvement 12
2. INTRODUCTION AND BACKGROUND 13
2.1. Trial rationale and aim 13
2.2. Background 13
2.3. Risk/Benefit assessment 15
2.3.1. Known potential risks 15
2.3.2. Known potential benefits 19
2.3.3. Assessment of potential risks and benefits 20
3 TRIAL OBJECTIVES AND OUTCOMES 20
3.1 Objectives .... Error! Bookmark not defined.
3.1.1 Primary objective .... Error! Bookmark not defined.
3.1.2 Secondary objectives .... Error! Bookmark not defined.
3.1.3 Exploratory objectives .... Error! Bookmark not defined.
3.2 Outcomes .... Error! Bookmark not defined.
4 TRIAL DESIGN 24
4.1 Overall design 24
4.2 Justification for dose 25
4.3 Trial population 25
4.4 Eligibility criteria 25
4.4.1 Inclusion criteria 25
4.4.2 Exclusion criteria 26
4.5 Lifestyle considerations 27
4.6 Screen failures 27
4.7 Recruitment and identification of potential participants 27

4.8 Consent .... **Error! Bookmark not defined.**
5 INTERVENTION 28
5.1 Treatment arms 28
5.2 Trial Intervention(s) 29
5.2.1 Description of trial investigational products 29
5.2.1.1 <Trial Product insert> .... **Error! Bookmark not defined.**
5.2.2 Dosage 29
5.2.3 Dose modification 29
5.2.4 Storage, preparation, dispensing and administration of trial drug 29
5.2.5 Product accountability 32
5.2.6 Measurement of participant compliance .... **Error! Bookmark not defined.**
5.2.7 Excluded medications and treatments 32
5.2.8 Concomitant therapy .... **Error! Bookmark not defined.**
5.2.9 Discontinuation from trial intervention 33
6 RANDOMISATION AND BLINDING 33
6.1 Concealment mechanism 33
6.2 Breaking of the trial blind .... **Error! Bookmark not defined.**
6.2.1 On trial .... **Error! Bookmark not defined.**
6.2.2 On completion of the trial .... **Error! Bookmark not defined.**
7 TRIAL VISITS AND PROCEDURES 34
7.1 Trial timeline 34
7.2 Schedule of assessments 35
7.3 Description of procedures 35
7.4 Notes on specific trial visits 37
7.4.1 Screening .... **Error! Bookmark not defined.**
7.4.2 Final trial visit .... **Error! Bookmark not defined.**
7.4.3 Unscheduled visit 37
7.5 Treatment discontinuation, participant withdrawals and losses to follow up 37
7.5.1 Discontinuation of treatment - participant remains in trial for follow up 37
7.5.2 Withdrawal of consent - participant withdraws from all trial participation 38
7.5.3 Losses to follow-up 38
7.5.4 Replacements 38
7.5.5 Trial Closure 38
7.5.6 Continuation of therapy 39

8 SAFETY MONITORING AND REPORTING ... 39
8.1 Definitions ... 39
8.1.1 Definitions for use in trials involving investigational medicinal products ... 39
8.1.2 Definitions for use in trials involving investigational medical devices **Error! Bookmark not defined.**
8.2 Capturing and eliciting adverse event/reaction information ... 39
8.3 Documentation of AEs ... 40
8.4 Assessing the seriousness of a participant's AE ... 41
8.5 Assessing the relatedness (causality) of a participant's AE ... 41
8.6 Assessing the expectedness of a participant's AE ... 41
8.7 Reporting of safety events ... 42
9 DATA AND INFORMATION MANAGEMENT ... 42
9.1 Overview ... 43
9.2 Data management ... 43
9.2.1 Data generation (source data) ... **Error! Bookmark not defined.**
9.2.2 Data capture methods and data use, storage, access and disclosure during the trial **Error! Bookmark not defined.**
9.2.3 Data confidentiality ... **Error! Bookmark not defined.**
9.2.4 Quality assurance ... **Error! Bookmark not defined.**
9.2.5 Archiving - Data and document retention ... **Error! Bookmark not defined.**
9.2.6 Data sharing ... **Error! Bookmark not defined.**
10 TRIAL OVERSIGHT ... 48
10.1 Governance structure ... 48
10.1.1 Trial Management Group (TMG) ... 48
10.1.2 Trial Steering Committee (TSC) ... 48
10.1.3 Safety Monitoring ... **Error! Bookmark not defined.**
10.2 Site Monitoring ... **Error! Bookmark not defined.**
10.3 Quality Control and Quality Assurance ... 48
11 STATISTICAL METHODS ... 48
11.1 Sample Size Estimation ... 49
11.2 Population to be analysed ... 49
11.2.1 Handling of missing data ... 50
11.3 Methods of analysis ... 50
11.4 Interim Analyses ... 50

12 ETHICS AND DISSEMINATION 51
12.1 Research Ethics Approval & Local Governance Authorisation 51
12.2 Amendments to the protocol 51
12.3 Protocol Deviations and Serious Breaches 52
13 CONFIDENTIALITY 52
14 PARTICIPANT REIMBURSEMENT 52
15 FINANCIAL DISCLOSURE AND CONFLICTS OF INTEREST 52
16 DISSEMINATION AND TRANSLATION PLAN 53
17 ADDITIONAL CONSIDERATIONS 53
18 REFERENCES 53
19 APPENDICES 55
19.1 Appendix 1: Division of sponsor responsibilities between sponsor and sponsor-investigator 55
19.2 APPENDIX 2: Significant Safety Issues (SSI) - some examples 58
19.3 APPENDIX 3: Expedited Safety Report Form ..... **Error! Bookmark not defined.**
19.4 APPENDIX 4: Specimens for biobanking - completed biobank registration form 59

PROTOCOL SYNOPSIS

TITLE	*Exploiting the Beneficial Off-Target Effects of BCG Vaccination to Protect Health Care Workers Against COVID-19*
TRIAL DESCRIPTION	Phase III, two group, multicentre, open label randomised controlled trial in up to 4170 health care workers to determine if BCG vaccine reduces prevalence and the severity of COVID-19 disease during the 2020 SARS-CoV-2 pandemic.<br><br>Randomisation and immunisation will occur with the annual staff influenza immunisation roll out at each site. Participants will be randomised to receive Influenza vaccine alone, or, both the BCG vaccine and Influenza vaccine. Participants will be followed-up for 12months with regular text messages (up to weekly) and surveys to identify and detail suspected COVID-19 infection. Additional information on severe disease will be obtained from hospital medical records and government databases. Blood samples will be collected at prior to randomisation and at 12 months to determine SARS-CoV-2 exposure. Where required swab/blood samples will be taken at illness episodes to assess SARS-CoV-2 infection.
OBJECTIVES	**Primary objectives**<br>1. To determine if BCG vaccination (Intervention) compared with no BCG vaccination (Comparator) reduce the prevalence of COVID-19 disease (Outcome) measured over the 6 months following randomisation (Time) in healthcare workers in Australia exposed to SARS-CoV-2 (Participants).<br>2. To determine if BCG vaccination (Intervention) compared with no BCG vaccination (Comparator) reduce the prevalence of severe of COVID-19 disease (with hospitalisation or death) (Outcome) measured over the 6 months following randomisation (Time) in healthcare workers in Australia exposed to SARS-CoV-2 (Participants).<br>**Secondary objectives**<br>3. To determine if BCG vaccination (Intervention) compared with no BCG vaccination (Comparator) reduce the prevalence of COVID-19 disease (Outcome) measured over the 12 months following randomisation (Time) in healthcare workers in Australia exposed to SARS-CoV-2 (Participants).<br>4. To determine if BCG vaccination (Intervention) compared with no BCG vaccination (Comparator) reduce the prevalence of severe of COVID-19 disease (hospitalisation or death) (Outcome) measured over the 12 months following randomisation (Time) in healthcare workers in Australia exposed to SARS-CoV-2 (Participants).

	5. To determine if BCG vaccination (Intervention) compared with no BCG vaccination (Comparator) prolong the time to first SARS-CoV-2-proven respiratory illness (Outcome) measured over the 12 months following randomisation (Time) in healthcare workers in Australia exposed to SARS-CoV-2 (Participants).<br>6. To determine if BCG vaccination (Intervention) compared with no BCG vaccination (Comparator) reduce the severity of COVID-19 disease (Outcome) measured over the 12 months following randomisation (Time) in healthcare workers in Australia exposed to SARS-CoV-2 (Participants).<br>7. To determine if BCG vaccination (Intervention) compared with no BCG vaccination (Comparator) reduce the rate and severity of febrile respiratory illness (fever with at least one sign or symptom of respiratory disease) measured over the 12 months following randomisation (Time) in healthcare workers in Australia (Participants).<br>8. To determine if BCG vaccination (Intervention) compared with no BCG vaccination (Comparator) reduce absenteeism (days off work) in healthcare workers in Australia (Participants).<br>9. To determine if BCG vaccination (Intervention) compared with no BCG vaccination (Comparator) reduce hospital-related health costs (days off work) in healthcare workers in Australia (Participants).<br>10. To evaluate the safety of BCG vaccination in adult healthcare workers in Australia.
OUTCOMES AND OUTCOME MEASURES	Primary outcomes:<br>1. Number of participants with COVID-19 disease defined as fever plus at least one sign or symptom of respiratory disease including cough, shortness of breath, respiratory distress/failure, runny/blocked nose (using self-reported questionnaire), plus a positive SARS-Cov-2 test (PCR or serology)over the 6 months following randomisation.<br>2. Number of participants who were admitted to hospital or died (using self-reported questionnaire and/or medical/hospital records) in the context of a positive SARS-CoV-2 test, over the 6 months following randomisation<br><br>Secondary outcomes: All assessed over the 12 months following randomisation unless otherwise indicated.<br>- The following outcomes are for both COVID-19 disease and febrile respiratory illness: Number of participants with: COVID-19 disease, days unable to work, days confined to bed, of days with symptoms, pneumonia, need for oxygen therapy, admission to critical care, need for mechanical ventilation

	- Number of episodes of COVID-19 disease / febrile respiratory illness<br>- Time to first symptom of COVID-19 / febrile respiratory illness<br>- Number of deaths<br>- Number of days of unplanned absenteeism<br>- Hospital-related health costs associated with participant hospitalisation<br>- Type and severity of local and systemic adverse event over the 3 months following randomisation<br>- Exploratory outcomes: Number of participants with, episodes of and time to first recurrence of herpes simplex recurrence.
TRIAL POPULATION	4170 adult employees of hospitals in Victoria involved in the study who intend *to* have the annual influenza vaccine as part of their hospital's annual influenza vaccine roll-out. Exclusion criteria are having BCG vaccine or influenza vaccine contraindications, previously had a SARS-CoV-2 positive test result and prior involvement this trial at an alternate study site. Participants will be randomised at 1:1 ratio giving 2085 per group.
DESCRIPTION OF SITES ENROLLING PARTICIPANTS	Participating sites are hospitals within Victoria.
DESCRIPTION OF INTERVENTIONS	BCG + influenza vaccination group and Influenza only vaccination (control) group: 0.5mL quadrivalent/trivalent (depending of participant age) inactivated influenza vaccine injected via intradermal / subcutaneous injection<br>BCG + influenza vaccination group: BCG Denmark, 0.1 mL injected intradermally over the distal insertion of the deltoid muscle onto the humerus
TRIAL DURATION	*5 years*
PARTICIPANT DURATION	*13 months from randomisation to final follow-ups*

GLOSSARY OF ABBREVIATIONS

ABBREVIATION	TERM
AE	Adverse Event
ANOVA	Analysis of Variance
AR	Adverse Reaction
BCG	Bacillus Calmette–Guérin vaccine (used to prevent tuberculosis)
BRF	Biobank Registration Form (MCRI)
COVID-19	coronavirus disease 19
CRF / eCRF	Case Report Form / electronic Case Report Form
DMC SMC	Data Monitoring Committee / Safety Monitoring Committee
DSMB	Data Safety Monitoring Board
ED	Emergency Department
GCP	Good Clinical Practice
HREC	Human Research Ethics Committee
ICH	International Conference on Harmonisation
ITT	Intention To Treat
MERS	Middle East respiratory syndrome
MCRI	Murdoch Children's Research Institute
NHMRC	National Health and Medical Research Council
NSE	Non-specific effects
PI / CPI	Principal Investigator / Coordinating or Chief Principal Investigator
PI	Product Information (available for an approved drug or device)
QA	Quality Assurance
QC	Quality Control
RGO	Research Governance Office
RCH	Royal Children's Hospital (Melbourne)
SAE	Serious Adverse Event
SAP	Statistical Analysis Plan
SAR	Serious Adverse Reaction
SARS-CoV-2	Severe Acute Respiratory Syndrome Coronavirus 2
SMC	Safety Monitoring Committee
SOP	Standard Operating Procedure
SSI	Significant Safety Issue
SUSAR	Suspected Unexpected Serious Adverse Reaction
TB	Tuberculosis
TGA	Therapeutic Goods Administration
UAR	Unexpected Adverse Reaction
USM	Urgent Safety Measure

Abbreviations specific to Investigational Medical Device trials:

ADE	Adverse Device Effect
IMD	Investigational Medical Device
SADE	Serious Adverse Device Effect
USADE	Unanticipated Serious Adverse Device Effect

We use the following terminology with regards to the term 'investigators':

- ***Sponsor-Investigator** – is used to describe the **overall trial level** Investigator for both multi-site (i.e. replaces the term Coordinating Principal Investigator) and single-site (i.e. replaces the term Principal Investigator) trials. This person has the role of both Sponsor and Investigator as defined in ICH GCP.*
- ***Participating Site Principal Investigator** – is used to describe **the site-level** Investigator (i.e. the site Principal Investigator) at a participating site (i.e. not the lead site) in a multi-site trial.*

INVESTIGATOR AGREEMENT

I have read the protocol entitled "*Exploiting the Beneficial Off-Target Effects of BCG Vaccination to Protect Health Care Workers Against COVID-19*".
By signing this protocol, I agree to conduct the clinical trial, after approval by a Human Research Ethics Committee or Institutional Review Board (as appropriate), in accordance with the protocol, the principles of the Declaration of Helsinki and the good clinical practice guidelines adopted by the TGA [Integrated Addendum to ICH E6 (R1): Guideline for Good Clinical Practice E6 (R2), dated 9 November 2016 annotated with TGA comments].
Changes to the protocol will only be implemented after written approval is received from the Human Research Ethics Committee or Institutional Review Board (as appropriate), with the exception of medical emergencies.
I will ensure that trial staff fully understand and follow the protocol and evidence of their training is documented on the trial training log.

Name	Role	Signature and date
Prof Nigel Curtis	Sponsor Investigator	
Prof Andrew Davidson	Co-investigator	
Prof Kanta Subbarao	Co-investigator	

1. ADMINISTRATIVE INFORMATION

1.1. Trial registration

This trial will be registered prior to commencement of recruitment on ClinicalTrials.gov

1.2. Sponsor

**Trial Sponsor**	MCRI
**Contact name**	Nigel Curtis
**Address**	Royal Children's Hospital, 50 Flemington Road
**Sponsor-Investigator**	Nigel Curtis

On behalf of the Sponsor, MCRI, the Sponsor-Investigator leading the trial will undertake and/or oversee those Sponsor responsibilities delegated by the Sponsor. The delegated Sponsor responsibilities are documented in Appendix 1 of the protocol 62586.

1.3. Expected duration of study

The recruitment period is expected to take 3 weeks in late April-early May 2020 as it will coincide with the staff influenza vaccination program at each site.

The intervention (BCG vaccine) administered once, so the treatment period is approximately 2mins. The individual's follow-up will be 1-year.

1.4. Contributorship

Name	Summary of contribution
Nigel Curtis:<br>- Leader of Infectious Diseases Group, MCRI<br>- Head of Infectious Diseases, RCH Melbourne<br>- Professor of Paediatric Infectious Diseases, The University of Melbourne	Study design, study lead
Prof Andrew Davidson<br>- Medical Director, Melbourne Children's Trial Centre<br>- Honorary Professor, The University of Melbourne<br>- Staff Anesthetist, RCH Melbourne<br>- Chair of MACH Clinical Trials Committee	Study design
Prof Kanta Subbarao<br>- Director of the WHO Collaborating Centre for Reference and Research on Influenza<br>- The Peter Doherty Institute for Infection and Immunity	Study design

Name	Summary of contribution
<u>MCRI Infectious Diseases Group</u><br>Kaya Gardiner (Group Research Co-ordinator and Chief Trial Co-ordinator)<br>Casey Goodall (MIS BAIR Co-ordinator)<br>Chris Richards (Infection Flagship Co-ordinator)<br>Amanda Gwee (Team Leader)<br>Laure Pittet (Post-doctoral researcher)<br>Nicole Messina (Post-doctoral researcher)<br>Susie Germano (Research assistant)<br>Veronica Abruzzo (Data manager)<br>Samantha Bannister (PhD student)<br>Eva Sudbury (PhD student)<br>Paola Villanueva (PhD student)<br>Joyce Chan (MDRP student)<br>Patricia Bimboese (Paeds trainee)<br><br><u>Melbourne Children's Trial Centre (MCTC)</u><br>Kirsten Perrett (Team Leader)<br>Katherine Lieschke (RCH Research Ethics and Governance)<br><br><u>Clinical Epidemiology & Biostatistics (CEBU)</u><br>Francesca Orsini (Biostatistician, Snr Research Officer)<br>Katherine Lee (Senior Biostatistician)<br>Ellie McDonald (Research Officer)<br><br><u>RCH Immunisation Centre</u><br>Sonja Elia (Manager, Immunisation Centre)	Study collaborators

1.5. Stakeholder involvement

Stakeholder	Engagement
Melbourne Children's Trials Centre (MCTC)	
Royal Children's Hospital (RCH)	
Emergency Department (ED) directors and staff where participants (staff) will be recruited	
Hospitals whose emergency department staff will be included as sites	

Department of Health (for each state)	
Melbourne Academic Centre for Health (MACH)	
Royal Children’s Hospital Immunisation Service	
Australian Health Research Alliance (AHRA)	

2. INTRODUCTION AND BACKGROUND

2.1. Trial rationale and aim

In recent months severe acute respiratory syndrome-coronavirus 2 (SARS-CoV-2) has emerged as a novel human pathogen. With no pre-existing immunity against this virus, susceptibly among humans is presumed to be universal. Healthcare workers are at the frontline of novel infectious disease outbreaks such as this. Due to their contact with patients and production of aerosols during some medical procedures they have greater exposure and potentially risk of contracting newly emerged human pathogens. Current strategies to protect healthcare workers rely on the use (and sustained supply) of personal protective equipment. Health care worker absenteeism due to infection with the outbreak pathogen or illness cause by another disease with similar symptoms, compounds the pressure already placed on the healthcare system.

Prophylactic interventions to protect against emerging pathogens are needed, particularly for healthcare workers. The tuberculosis (TB) vaccine, Bacillus Calmette-Guérin (BCG) has beneficial off-target effects and has been shown to protect against non-TB infections[1]. This is proposed to result from BCG mediated boosting of early immune responses. As such, BCG vaccination represents a potential prophylactic intervention to provide protection against emerging pathogens such as SARS-CoV-2.

The aim of this trial is to determine whether in healthcare workers, BCG can reduce the prevalence and severity of illness caused by the novel coronavirus, SARS-CoV-2.

2.2. Background

Since the emergence of coronavirus disease 19 (COVID-19) in China in December 2019, there have been over 150,00 cases disease and greater than 5000 deaths caused by the disease globally[2]. The causative agent of COVID-19 a novel coronavirus, severe acute respiratory syndrome-coronavirus 2 (SARS-CoV-2), has already spread to 108 countries (including over 200 cases in Australia) and it is predicted that up to 60% of the global population could become infected[3]. Following from SARS in 2002[4] and Middle East respiratory syndrome (MERS) in 2012[5], SARS-CoV-2 is the third coronavirus to make the jump from animals to humans and emerge as a serious human pathogen in less than 20 years.

In approximately 80% of cases COVID-19 results in mild to moderate disease with symptoms similar to common respiratory diseases such as influenza-like illnesses, with fever in the majority (87.9%) of cases, followed by dry cough (67.7%), fatigue (38.1%), sputum production (33.4%)[6]. In 14% of cases, SARS-CoV-2 causes severe disease requiring oxygen supplementation and/or mechanical ventilation, with a further 6% being critical cases that have respiratory failure, septic shock and/or organ failure.

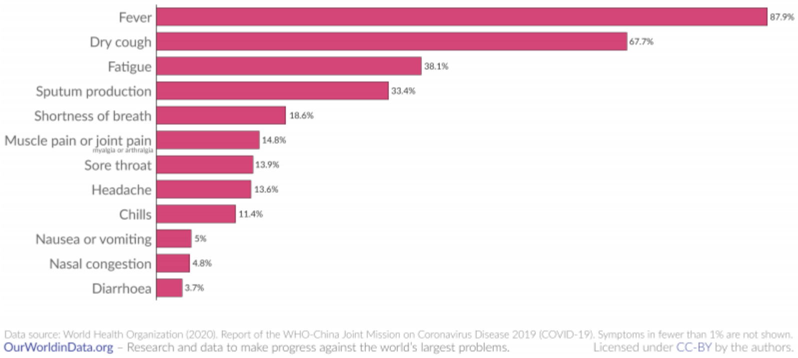

There are worldwide efforts to reduce the peak of SARS-CoV-2 infection, in order to have enough hospital resources. However, with no vaccines or preventative interventions available to protect against COVID-19 disease, current strategies rely on conventional control measures including travel restrictions, quarantines and increased hygiene practices. The overlap of COVID-19 symptoms with common respiratory diseases makes screening for SARS-CoV-2 infection difficult with diagnosis relying on microbiological confirmation of SARS-CoV-2 infection. Moreover, healthcare workers with these common respiratory symptoms are advised to be tested for SARS-CoV-2 infection prior to return to work. The loss of these healthcare workers with non-COVID-19 respiratory infections due to quarantine requirements places further pressure on the healthcare system during this critical time.

BCG, a vaccine given to over 120 million infants annually to protect against TB, represents a potential prophylactic intervention for the prevention of COVID-19 disease. In addition to protecting against TB, BCG has beneficial off target (also termed 'heterologous' or 'non-specific') effects that protect against unrelated infections in children and adults[7-11].

The beneficial off-target effects of BCG vaccination have been most extensively studied in children. A world health organisation (WHO)-commissioned meta-analysis of 12 studies in high mortality settings concluded that BCG vaccination reduces all-cause mortality in children under 5-years of age by 30-53%[8]. This protection is evident within days of vaccination is proposed to be attributable to reduced deaths from infections other than TB, particularly respiratory tract infections and sepsis. Two large cohort studies in children similarly found that BCG reduces non-TB infections. The first, a 25-year retrospective study of over 150,000 children from 33 countries reported that BCG-vaccinated children had an up to 37% lower risk of acute lower respiratory tract infections[12]. The second, a study of paediatric hospitalisations in Spain, found that BCG-vaccinated children had a 41% lower risk of serious respiratory infection and 53% lower risk of sepsis not related to TB[13].

In adults, in a human challenge model, prior BCG vaccination reduced viraemia by over 70% and improved anti-viral immune responses to yellow fever vaccine virus[14]. Notably, yellow fever virus is a single-stranded, positive-sense RNA virus like SARS-CoV-2. Consistent with BCG mediated protection against infections, in two randomised control trials in adults, BCG vaccination reduced incidence of acute upper tract respiratory infections by 70-80%[15,16].

Several studies have also shown that BCG can reduce symptoms in human papilloma virus infection and herpes simplex virus infection adults[17].

A plethora of studies in animal models, have also shown that BCG protects against disease and mortality caused by a wide range of bacterial, fungal, protozoan and viral infections including infections with single-stranded, positive-sense RNA viruses[18-20].

The beneficial off-target effects of BCG are proposed to result from BCG induced changes in immune responses[1,14,19]. In adults, BCG vaccination increases immune responses to unrelated pathogens, an effect that is sustained for at least a year after vaccination[21]. BCG vaccination also boosts antibody responses to several vaccines including influenza vaccine[22-24]. Thus, in addition to protecting against viral infections, BCG provides further protection by increasing the efficacy of other vaccinations.

Therefore, by boosting the immune system, BCG vaccination may provide early protection against new human pathogen thus reducing their spread and severity. This will be of particular benefit among healthcare workers and high-risk groups for whom contraction of the disease would have the greatest impact.

This trial will determine whether BCG vaccination (given at the time of routine annual influenza vaccination), compared to influenza vaccination alone reduces the prevalence and severity of COVID-19 but also whether BCG vaccination reduces other respiratory illnesses in healthcare workers. In this case of COVID-19, where symptoms overlap with common respiratory diseases and diagnostic tests currently take several days, the prevention of non-CODVID-19 respiratory illnesses will also reduce the strain on the healthcare system caused by the outbreak. This is particularly important in Australia and other countries in the southern hemisphere as the outbreak peak is expected to occur during the winter influenza season.

The results of this trial will establish whether, in future novel disease outbreaks, BCG vaccination could be implemented as an early intervention to protect healthcare workers and high-risk groups.

2.3. Risk/Benefit assessment

2.3.1. Known potential risks

This study involves minimal risk to participants.

All health care workers in the study will receive influenza vaccine as per standard of care.

HCWs randomised to receive BCG vaccine will have known potential risks associated with BCG vaccination. These risks are slightly increased for HCWs who have previously had BCG vaccine (revaccination), compared to HCWs receiving BCG vaccine for the first time (vaccine naïve).

There are additional known minimal risks for all HCWs re: blood tests and respiratory swabs.

<u>BCG vaccination</u>

Expected (common) reactions to BCG vaccination[25]:

- A small swelling, redness and tenderness (measuring 0.5-1.5 cm in diameter) at the injection site appears within 1-2 weeks at the injection site. The local lesion evolves into a small ulcer. The ulcer heals over several weeks to months, usually healing into a small flat scar.
- Slightly swollen lymph nodes in the axilla in up to 10% of recipients, and usually resolve spontaneously.

** *Revaccination is associated with an earlier, accelerated reaction which begins within 24–48 hours of vaccination with induration followed by pustule formation in 5–7 days and healing within 10–15 days*[26] (https://www1.health.gov.au/internet/main/publishing.nsf/Content/cda-cdi3701h.htm)
** *Tuberculous skin lesions are more common in people over 15 years or with revaccination)*[27,28].

Uncommon side effects of BCG vaccination[25,29-31]:

- large ulcer, abscess at the injection site (2.5% of vaccinated recipients)
- Keloid scar at injection site.
- Headache
- Fever
- Swelling of lymph nodes in the armpit larger than 1 cm across

Rare side effects (up to 1 in 100)

- Significant inflammation of lymph nodes in the axilla, sometimes with oozing ulcers, possibly abscess (up to 1% of BCG vaccine recipients)
- Infection with the bacteria from the vaccine can occur. The infection can spread throughout the body, including the bones (osteomyelitis)
- Allergic reaction or anaphylaxis (e.g.: redness of the face and neck, swelling of the face, throat or neck, skin rash, breathing difficulties and collapse)
- Fainting, seizures and convulsions (rare among patients receiving injections)

Very rare side effects (1–4 cases per million vaccinated people[25]):

Disseminated BCG infection has been reported rarely after BCG vaccination, mainly in immunocompromised individuals (who are excluded from the trial).

Co-administration of vaccines:

As indicated in the Australian immunisation handbook, BCG vaccine can be given at the same time as, or at any time after, other inactivated vaccines thus there is no additional risk for co-administration of influenza and BCG vaccines[25].

BCG vaccination in Australia (current recommendations)

BCG vaccination in Australia is limited to selected high risk groups and is not routinely recommended for most health care workers (HCW)[26]. BCG vaccination is recommended for Aboriginal and Torres Strait Islander neonates in communities with a high incidence of TB; neonates and children 5 years of age and under who will be travelling or living in areas with a

high prevalence of TB for extended periods; and neonates born to parents with leprosy. BCG should be considered in HCWs who may be at high risk of exposure to drug resistant cases. It is recommended that all individuals have a tuberculin skin test (TST) prior to BCG vaccination, except infants less than 6 months of age with no history of tuberculosis (TB) contact, and that BCG should not be given to an individual with a tuberculin reading of 5mm or more. Additionally, BCG revaccination is not recommended, regardless of TST reaction size[26].

BCG vaccination in Australia (current contraindications)

- BCG is contraindicated in immunocompromised individuals due to the risk of disseminated BCG infection[26]. This includes individuals immunocompromised by HIV infection, primary immunodeficiencies, corticosteroids or other immunosuppressive agents, and malignancies involving bone marrow of lymphoid systems.
- BCG is also contraindicated in individuals with any serious illness and those with generalised septic skin diseases and active skin conditions such as eczema, dermatitis and psoriasis near the site of vaccination[25].
- While BCG has not been shown to cause foetal damage the use of live vaccines is contraindicated in pregnancy [26].
- Individuals who have previously had tuberculosis or a large tuberculin (TST) reaction

In this study, HCWs will be excluded from the study if they are immunocompromised, have serious illness, skin disease at site of vaccination or are pregnant (pregnancy test will be performed on all women of child-bearing age prior randomised to BCG vaccination group).

Global BCG recommendations and practices

The current World Health Organization (WHO) position is that BCG revaccination is not recommended for any person, as there is no evidence to support the role of BCG revaccination in protection against tuberculosis[32]. A number of countries have previously included BCG revaccination as part of their national immunisation policies[33]. In 1999, 30 countries in Europe and an additional 18 countries in the Middle East, South East Asia and the Western Pacific region reported using BCG revaccination. In several countries the national policy included BCG in infancy and again at school entry or leaving. In other countries, particularly in Eastern Europe, revaccination with BCG up to age five has been recommended. Some countries, such as Poland, recommended universal revaccination while others restrict revaccination to individuals without a BCG scar or those with a 'negative' TST. Criteria for TST negativity differs between countries [34,35]. ***In countries where BCG revaccination has been part of national immunisation practice, passive surveillance has not reported any cases of disseminated BCG in immunocompetent individuals.***

Pre-vaccination screening

TST and interferon gamma release assay (IGRA) screening aims to identify individuals with latent tuberculosis infection (LTBI)[36]. The diameter of induration following TST gives an indication of the likelihood of LTBI, however, positive results can also arise from previous BCG vaccination and exposure to environmental mycobacteria. This is in contrast to IGRA which are unaffected by previous BCG vaccination. A positive IGRA indicates either current or past infection with TB[36]. Screening of individuals using TST prior to BCG vaccination is

recommended in Australia and other countries on the grounds that it may prevent complications due to pre-existing immunity due to previous exposure to mycobacterial antigens [28]. ***However, a large review of adverse effects of over 1.5 billion doses of BCG vaccine in adults and children showed that a positive TST did not increase the likelihood of complications from the BCG vaccine and did not predict the development of local skin reactions, abscesses or axillary lymphadenitis[27].***

Trials of BCG revaccination

Three large randomised controlled trials of BCG revaccination in children and adults in Malawi (n=54865), children in Guinea Bissau (n=2871) and adolescents in South Africa (n=990) did not show increased rates of serious adverse events among BCG revaccinated participants[15,37,38]. Participants in the Malawi study did not undergo any pre-randomisation screening with tuberculin skin test (TST) or interferon gamma release assay (IGRA)[37]. This study found a lower rate of leprosy amongst revaccinated participants but no difference in the rates of tuberculosis or death between the groups. Of the children in the Guinea Bissau study, 3 of 6 children with a measurable TST (1-14mm) had increased rates of large local reaction compared to controls (18/388). Two months after revaccination all had healed vaccination scars with no axillary node enlargement, fever or suppurative lymphadenitis[38]. Participants in the South African study all had a negative IGRA at enrolment[15]. Among BCG revaccinated adolescents 93% reported mild local injection site reactions including swelling, induration, discharge, erythema, scab and ulceration. This was compared to 25% in the placebo group. The rates of moderate injection site reactions were similar between the BCG (5%) and placebo (6%) groups. There was 1 severe and 7 serious adverse events in each of the BCG and control groups. The serious adverse events reported in the BCG arm were not attributed to BCG revaccination and included gastroenteritis, chest injury, thermal burn, intentional self-injury, suicide attempt and small intestinal obstruction. The rate of upper respiratory tract infections was also lower in the BCG revaccinated group compared to placebo (2.1% compared to 7.9%, $p<0.001$).

Further studies looking at BCG revaccination in individuals with positive TST or IGRA do not show increased risk of significant adverse effects. A case-control study of 200 healthy nursing students in India included 28 participants with a positive IGRA who received BCG revaccination[39]. There were no serious side effects reported and no participants developed active tuberculosis during the follow-up study period. A randomised controlled trial of BCG revaccination in healthy adults with a positive TST (>15mm) with or without isoniazid pre-treatment (n=82) showed no difference in the rate of reactions between groups with only local injection site reactions (35-76%) and mild systemic adverse effects (19%) including headache, fever and nausea [40]. Among the 76% of participants who developed ulceration the median ulcer size was 5mm (IQR 4.0-6.0). Maximum ulcer diameter did not correlate with IGRA result prior to BCG vaccination in either group. There were no reports of regional lymphadenitis or serious morbidity.

Enhanced routine passive surveillance of BCG revaccinated school children in the BCG-REVAC trial in Brazil is available for 71718 individuals [41]. There are only 33 reported adverse events of which 60% were local cutaneous reactions and 28% axillary lymphadenopathy without suppuration. There were no deaths, permanent injuries or disseminated infections reported.

In a case series of 13 children who experienced adverse events following BCG revaccination in Brazil all developed local ulceration or abscess formation with complete recovery following antimycobacterial therapy[42]. There were no cases of suppurative lymphadenitis or disseminated BCG. Further, an ongoing randomised trial in 150 participants in the US is giving repeat BCG (two vaccinations in the first year, then annually for 4 years) to adults aged 18-65 with type 1 diabetes to test if multiple BCG vaccinations can improve diabetic control and prevent complications[43]. They have reported variable local reactions but no increased risk of lymphadenopathy or disseminated BCG (Denise Faustman, personal communication).

The data presented above supports the WHO position that while BCG revaccination is not recommended due to a lack of evidence of efficacy against tuberculosis the risk of administering BCG vaccine to persons with positive tuberculin reactions due to either prior BCG vaccination or to natural infection is minimal.

One aim of the BCG-19 study is to document the safety of BCG vaccination (and revaccination) in healthcare workers in Australia. The decision not to perform pre-vaccination TST screening in the study is pragmatic in order to reduce barriers to participation for already busy and stretched healthcare workers during the current COVID-19 outbreak. While it does not align with current Australian vaccination guidelines it has been carefully considered upon systematic review of the literature presented above.

Risks related to blood sample collection

Having a blood test can sometimes cause some pain from the needle or be uncomfortable. Occasionally a small amount of bruising can occur on the skin where the blood was taken. Trained members of the research team will collect the blood samples from participants.

Risks related to respiratory swab collection

Having a respiratory swab can sometimes be uncomfortable. Trained members of the research team will collect the respiratory swabs from participants.

2.3.2. Known potential benefits

In Australia, BCG is given to infants and children under 5-years of age travelling to TB endemic areas. In adults its efficacy is variable and likely to have little effect in adults in Victoria as their risk of TB is very low. BCG also protects against non-TB mycobacterial infections (e.g. leprosy) but these are also rare in Australia.

However, due to the beneficial off target effects of BCG, BCG vaccination may reduce COVID-19 illness and other respiratory infections in study participants. In addition to the direct benefit this would give the participants by reducing disease, this would also benefit the healthcare facilities that they work at by reducing their need to be absent (symptom related quarantine or illness) and thus enabling them to continue working and supporting the healthcare system during this period of intense demand.

2.3.3. Assessment of potential risks and benefits

BCG vaccination has a well-established safety profile in healthy individuals. While there are known adverse reactions to BCG, serious adverse reactions are rare. BCG vaccination does also cause a scar in over 80% participants. Participants will be screened prior to BCG vaccination to ensure they have no known contraindications for BCG vaccination. Vaccination will be done by staff trained in intradermal injection to reduce the potential subcutaneous injection which can increase scarring. Blood tests and respiratory swabs will be done by trained staff.

Given the minor risks of BCG vaccination, the potential benefits of BCG vaccination for the participants (by reducing COVID and other respiratory infections), the healthcare system (by reducing absenteeism) during this current COVID-10 outbreak far outweigh them. In addition to this current outbreak, the findings of this study have major implications for future outbreak responses globally. If BCG vaccination is found to be effective at reducing COVID-19, BCG vaccination could be implemented as an early preventative intervention in future outbreaks to protect healthcare workers globally. BCG vaccine is cheap and already administered to infants in over 80% of countries worldwide, therefore implementation of BCG vaccination campaigns during outbreaks is a feasible intervention to complement other preventative strategies.

We will be using BCG vaccine outside of its standard/recommended use in Australia, therefore, as per use of any intervention outside of standard regulations we will be assessing the reactogenicity and safety of BCG vaccination in vaccine naïve and previously vaccinated health care workers.

3 TRIAL OBJECTIVES AND OUTCOMES

3.1 Objectives

Two primary outcomes have been chosen for this study: occurrence of COVID-19 disease and occurrence of severe COVID-19 disease. Considering the number of unknown factors and the little knowledge of this new virus, we deemed it of clinical importance to have sufficient power to detect the potential effect of BCG vaccine compared to control for both outcomes (occurrence of any COVID-19 disease, as well as occurrence of severe COVID-19). Our hypothesis is that, compared to control, the BCG vaccine will be able to shift the “severity of COVID-19” curve down, i.e. to generally reduce the severity of the symptoms in health care workers. In other words, we have the hypothesis that BCG will reduce both the number of cases of COVID-19 (increase the number of asymptomatic SARS-CoV-2 infections) and the number of severe cases of COVID-19.

Because of the potential for multiplicity testing, the method of controlling type I error is explained in the sample size section (4.1).

3.1.1 Primary objective

1. To determine if BCG vaccination (Intervention) compared with no BCG vaccination (Comparator) reduce the prevalence of COVID-19 disease (Outcome) measured over the

6 months following randomisation (Time) in healthcare workers in Australia exposed to SARS-CoV-2 (Participants).

2. To determine if BCG vaccination (Intervention) compared with no BCG vaccination (Comparator) reduce the prevalence of severe of COVID-19 disease (with hospitalisation or death) (Outcome) measured over the 6 months following randomisation (Time) in healthcare workers in Australia exposed to SARS-CoV-2 (Participants).

3.1.1 Secondary objectives

3. To determine if BCG vaccination (Intervention) compared with no BCG vaccination (Comparator) reduce the prevalence of COVID-19 disease (Outcome) measured over the 12 months following randomisation (Time) in healthcare workers in Australia exposed to SARS-CoV-2 (Participants).
4. To determine if BCG vaccination (Intervention) compared with no BCG vaccination (Comparator) reduce the prevalence of severe of COVID-19 disease (hospitalisation or death) (Outcome) measured over the 12 months following randomisation (Time) in healthcare workers in Australia exposed to SARS-CoV-2 (Participants).
5. To determine if BCG vaccination (Intervention) compared with no BCG vaccination (Comparator) prolong the time to first SARS-CoV-2-proven respiratory illness (Outcome) measured over the 12 months following randomisation (Time) in healthcare workers in Australia exposed to SARS-CoV-2 (Participants).
6. To determine if BCG vaccination (Intervention) compared with no BCG vaccination (Comparator) reduce the severity of COVID-19 disease (Outcome) measured over the 12 months following randomisation (Time) in healthcare workers in Australia exposed to SARS-CoV-2 (Participants).
7. To determine if BCG vaccination (Intervention) compared with no BCG vaccination (Comparator) reduce the rate and severity of febrile respiratory illness (fever with at least one sign or symptom of respiratory disease) measured over the 12 months following randomisation (Time) in healthcare workers in Australia (Participants).
8. To determine if BCG vaccination (Intervention) compared with no BCG vaccination (Comparator) reduce absenteeism (days off work) in healthcare workers in Australia (Participants).
9. To determine if BCG vaccination (Intervention) compared with no BCG vaccination (Comparator) reduce hospital-related health costs (days off work) in healthcare workers in Australia (Participants).
10. To evaluate the safety of BCG vaccination in adult healthcare workers in Australia.

3.1.2 Exploratory objectives

11. To determine in a subgroup of adults with recurrent cold sores whether BCG vaccination compared with no BCG vaccination reduces herpes simplex recurrences (such as cold sores).

3.2 Outcomes

Table listing objectives and outcomes

OBJECTIVE	OUTCOME & OUTCOME MEASURE
**Primary**	
1. To determine if BCG vaccination (Intervention) compared with no BCG vaccination (Comparator) reduce the prevalence of COVID-19 disease (Outcome) measured over the 6 months following randomisation (Time) in healthcare workers in Australia exposed to SARS-CoV-2 (Participants).	Number of participants with COVID-19 disease defined as<br>- fever (using self-reported questionnaire), plus<br>- at least one sign or symptom of respiratory disease including cough, shortness of breath, respiratory distress/failure, runny/blocked nose (using self-reported questionnaire), plus<br>- positive SARS-Cov-2 test (PCR or serology)<br>over the 6 months following randomisation
2. To determine if BCG vaccination (Intervention) compared with no BCG vaccination (Comparator) reduce the prevalence of severe of COVID-19 disease (with hospitalisation or death) (Outcome) measured over the 6 months following randomisation (Time) in healthcare workers in Australia exposed to SARS-CoV-2 (Participants).	Number of participants who were admitted to hospital or died (using self-reported questionnaire and/or medical/hospital records) in the context of a positive SARS-CoV-2 test, over the 6 months following randomisation
**Secondary**	
3. To determine if BCG vaccination (Intervention) compared with no BCG vaccination (Comparator) reduce the prevalence of COVID-19 disease (Outcome) measured over the 12 months following randomisation (Time) in healthcare workers in Australia exposed to SARS-CoV-2 (Participants).	Number of participants with COVID-19 disease as defined above over the 12 months following randomisation
4. To determine if BCG vaccination (Intervention) compared with no BCG vaccination (Comparator) reduce the prevalence of severe of COVID-19 disease (hospitalisation or death) (Outcome) measured over the 12 months following randomisation (Time) in healthcare workers in Australia exposed to SARS-CoV-2 (Participants).	Number of participants with severe COVID-19 disease as defined above over the 12 months following randomisation
5. To determine if BCG vaccination (Intervention) compared with no BCG vaccination (Comparator) prolong the time to first SARS-CoV-2-proven respiratory illness (Outcome) measured over the 12 months following randomisation (Time) in healthcare workers in Australia exposed to SARS-CoV-2 (Participants).	Time to first symptom of COVID-19 in a participant who subsequently meets the case definition over the 12 months following randomisation.
6. To determine if BCG vaccination (Intervention) compared with no BCG vaccination (Comparator) reduce the severity of COVID-19 disease (Outcome) measured over the 12 months following randomisation (Time) in healthcare workers in Australia exposed to SARS-CoV-2 (Participants).	All the following measures will be assessed, over the 12 months following randomisation<br>Number of participants with COVID-19 disease as defined above<br>Number of episodes of COVID-19 disease as defined above<br>Number of participants with asymptomatic SARS-CoV-2 infection defined as<br>- Evidence of SARS-CoV-2 infection (by PCR or seroconversion)

OBJECTIVE	OUTCOME & OUTCOME MEASURE
	- Absence of respiratory illness (using self-reported questionnaire)<br>- No evidence of exposure prior to randomisation (inclusion serology negative)<br><br>Number of days unable to work (using self-reported questionnaire) due to COVID-19 disease as defined above (excludes quarantine/workplace restrictions)<br><br>Number of days confined to bed (using self-reported questionnaire) due to COVID-19 disease as defined above<br><br>Number of days with symptoms in any episode of illness that meets the above the case definition for COVID-19 disease<br><br>Number of pneumonia cases (abnormal chest X-ray) (using self-reported questionnaire and/or medical/hospital records) associated with a positive SARS-CoV-2 test<br><br>Need for oxygen therapy (using self-reported questionnaire and/or medical/hospital records) associated with a positive SARS-CoV-2 test<br><br>Number of admission to critical care (using self-reported questionnaire and/or medical/hospital records) associated with a positive SARS-CoV-2 test<br><br>Need of mechanical ventilation (using self-reported questionnaire and/or medical/hospital records) and a positive SARS-CoV-2 test<br><br>Number of deaths (from death registry) associated with a positive SARS-CoV-2 test<br><br>Data will be collected in self-reported participant questionaries, medical/hospital records and/or government registries
7. To determine if BCG vaccination (Intervention) compared with no BCG vaccination (Comparator) reduce the rate and severity of febrile respiratory illness (fever with at least one sign or symptom of respiratory disease) measured over the 12 months following randomisation (Time) in healthcare workers in Australia (Participants).	All the following measures will be assessed, over the 12 months following randomisation<br><br>For the following outcomes, febrile respiratory illness will be defined as:<br>- fever (using self-reported questionnaire), plus<br>- at least one sign or symptom of respiratory disease including cough, shortness of breath, respiratory distress/failure, runny/blocked nose (using self-reported questionnaire)<br><br>Number of participants with febrile respiratory illness, as defined above<br><br>Number of episodes of febrile respiratory illness, as defined above<br><br>Number of days unable to work (using self-reported questionnaire) due to febrile respiratory illness, as defined above (excludes quarantine/workplace restrictions)<br><br>Number of days confined to bed (using self-reported questionnaire) due to febrile respiratory illness, as defined above

OBJECTIVE	OUTCOME & OUTCOME MEASURE
	Number of days with symptoms in any episode of illness that meets the above the case definition febrile respiratory illness<br>Number of pneumonia cases (abnormal chest X-ray) (using self-reported questionnaire and/or medical/hospital records)<br>Need for oxygen therapy (using self-reported questionnaire and/or medical/hospital records)<br>Number of admission to critical care (using self-reported questionnaire and/or medical/hospital records)<br>Need of mechanical ventilation (using self-reported questionnaire and/or medical/hospital records)<br>Number of deaths (from death registry)<br>This data will be collected in self-reported participant questionaries, medical/hospital records and/or government registries
8. To determine if BCG vaccination (Intervention) compared with no BCG vaccination (Comparator) reduce absenteeism (days off work) in healthcare workers in Australia (Participants).	Number of days of unplanned absenteeism for any reason (using self-reported questionnaire) over the 12 months following randomisation
9. To determine if BCG vaccination (Intervention) compared with no BCG vaccination (Comparator) reduce hospital-related health costs (days off work) in healthcare workers in Australia (Participants).	Hospital-related health costs associated with participant hospitalisation (HIS records) over the 12 months following randomisation
10. To evaluate the safety of BCG vaccination in health care workers.	Type and severity of local and systemic adverse event over the 3 months following randomisation will be collected in self-reported questionnaire and graded using toxicity grading scale.
Exploratory	
11. To determine in a subgroup of participants with recurrent cold sores whether BCG vaccination compared with no BCG vaccination reduces herpes simplex recurrence (such as cold sores).	Number of participants with herpes simplex recurrence (using self-reported questionnaire) over the 12 months following randomisation<br>Number of episodes of herpes simplex recurrence (using self-reported questionnaire) over the 12 months following randomisation<br>Time: to first of herpes simplex recurrence (using self-reported questionnaire) over the 12 months following randomisation

4 TRIAL DESIGN

4.1 Overall design

This is a phase III, two group, multicentre, open label randomised controlled trial (RCT) in up to 4170 frontline health care workers to determine if BCG vaccine reduces prevalence and the severity of COVID-19 disease during the 2020 SARS-CoV-2 pandemic.

Randomisation and immunisation will coincide with the annual staff influenza immunisation roll out at each hospital. Participants will be randomised to receive Influenza vaccine alone, or, both the BCG vaccine and Influenza vaccine.

Each hospital, independent of the research team will decide when to roll-out the annual staff influenza immunisation program. This decision is based on the availability of Influenza vaccine and the current rates of influenza in the community. Based on the information available, we predict that hospitals will likely to start rolling out the influenza vaccine around the middle of April. For most hospitals in Melbourne, the majority of staff (up to 6000 staff/hospital) are immunised over the first two weeks of the roll-out.

The control group will receive no placebo. Most people vaccinated with the BCG vaccine develop a papule/blister at the injection site around two-weeks after vaccination. Due to this, even using a placebo, it is not possible to blind participants to their treatment group allocation. The outcomes (prevalence of COVID-19 disease or admission to hospital for COVID-19 disease) are objective measures, it is not expected that participant's awareness of their group allocation would bias the study results significantly. Members of the research team doing the analysis will be blinded to the group allocation (by the removal of this variable and all other variables related to BCG from the dataset) until the formal detailed statistical analysis plan is confirmed and signed by all investigators and all data cleaning/preparation is complete.

Randomisation will be stratified for all factors that might influence the effectiveness of the intervention. For more details see section 6.

For participants randomised to the intervention (BCG vaccine), vaccination will occur within 24-hours.

Follow-up for all participants will last 1-year. For each episode of fever with a respiratory symptom during the follow-up period, all participants complete an online survey and will have a home visit by members of the research team for sample collection (respiratory swab preferred, however blood sample will be taken if no swab testing kits are available).

4.2 Justification for dose

The dose and route of BCG administration are the standard accepted dosage for BCG vaccine when used to prevent TB. There is no justification to vary from this.

4.3 Trial population

4.4 Eligibility criteria

Participants will be assigned to a randomised trial treatment only if they meet all of the inclusion criteria and none of the exclusion criteria.

4.4.1 Inclusion criteria

- Over 18 years of age
- Employed by one of the hospital's involved in the study
- Provide a signed and dated informed consent form

- Is having the influenza vaccine as part of their hospital's annual influenza vaccine roll-out
- Pre-randomisation blood collected

4.4.2 Exclusion criteria

- Has any BCG vaccine contraindication (combined from BCG SSI product information and Australian Immunisation Handbook)
    - Fever or generalised skin infection (where feasible, randomisation can be delayed until cleared)
    - Weakened resistance toward infections due to a disease in/of the immune system
        - Receiving medical treatment that affects the immune response or other immunosuppressive therapy in the last year.
            - These therapies include systemic corticosteroids (≥20 mg for ≥2 weeks), non-biological immunosuppressant (also known as 'DMARDS'), biological agents (such as monoclonal antibodies against tumour necrosis factor (TNF)-alpha).
        - People with congenital cellular immunodeficiencies, including specific deficiencies of the interferon-gamma pathway
        - People with malignancies involving bone marrow or lymphoid systems
    - People with any serious underlying illness (such as malignancy)
        - People with cardiovascular disease, hypertension, diabetes, and/or chronic respiratory disease are eligible if not immunocompromised
    - Known or suspected HIV infection,[11] even if they are asymptomatic or have normal immune function.
        - This is because of the risk of disseminated BCG infection[12,13]
    - People with active skin disease such as eczema, dermatitis or psoriasis at or near the site of vaccination
        - A different site (other than left arm) can be chosen if necessary
    - Pregnant or breastfeeding
        - Although there is no evidence that BCG vaccination is harmful during pregnancy or breastfeeding, it is a contra-indication to BCG vaccination. Therefore, we will exclude women who think they could be pregnant.
    - Another live vaccine administered in the month prior to randomisation
    - Require another live vaccine to be administered within the month following BCG randomisation
        - If the other live vaccine can be given on the same day, this exclusion criteria does not apply
    - Known anaphylactic reaction to any of the ingredient present in the BCG or influenza vaccine

- BCG vaccine given within the last year
- Have previously had a SARS-CoV-2 positive test result
- Already part of this trial, recruited at a different hospital.

4.5 Lifestyle considerations

Not applicable

4.6 Screen failures

Screen failures are defined as participants who consent to participate in the trial but who are found, during the screening procedures, to be ineligible to continue in the trial. They therefore do not receive the intervention / are not randomised.

4.7 Recruitment and Consent

In the period before staff influenza vaccinations are rolled out at the hospital, all staff (potential participants) at the participating sites will receive information (via email and/or hospital notice board) about the trial from the hospital. This will include a short blurb about the study and a link to a website where they can read further information including the participant information and consent form (PICF).

Interested staff will be able to contact the research team by phone or video conferencing if they have any questions (social distancing practices will still be applied wherever possible).

If they are interested in participating, they will click a link on the website which will take them to a REDCap form where they will need to answer questions designed to determine their eligibility. After submitting the form, if eligible, they will be taken to the electronic PICF (also in their REDCap record) where they will sign their signature electronically. Consent will be voluntary and free from coercion. A copy of the signed consent form will be emailed direct from REDCap once the participant completes the form.

If they are ineligible, the next page of the form will thank them for their interest but inform them that they are ineligible. No identifying information will be collected on these participants.

(N.B. this webpage text, electronic PICF and eligibility questionnaire will be submitted to the HREC for review and no participants will see it until approval is granted).

For those who are eligible and provide informed consent, they will be asked to fill in their contact details (including date of birth, Medicare card number, name and other identifying details) and a baseline questionnaire (demographics and environmental information required for table 1 of the final results paper) (N.B. these questionnaires will be submitted to the HREC for review and no participants will see it until approval is granted). It will be possible for participants to save and return to the REDCap form to complete/update this at a later time, prior to randomisation.

We have decided to do pre-consent online due to the time constraints of the pending pandemic and the logistics of the staff Influenza vaccine rollout:

1. The hospital aims for all staff to get the Influenza vaccine in a short period of time (~2-weeks). Due to this, there is already significant work done to ensure that there is

no impact to patient care during the staff influenza vaccine roll out. By ensuring all participants are consented and eligibility confirmed prior to coming to the clinic (and providing additional nursing support for the hospitals' influenza program), we hope to limit the impact on the hospitals' functioning.

2. For BCG vaccination to be effective to prevent COVID-19 disease prevalence and severity, it needs to be given before the peak of the pandemic in Australia, which we expect to be in May 2020.
3. There will likely only be approximately 1-week between governance approval at each site and the start of their staff influenza roll out, so there will be insufficient time or opportunity to face-to-face consent all staff without impacting patient care.

Participants will be told (in the PICF) and on REDCap after submitting the consent form, to identify themselves to the researchers at the staff influenza clinic PRIOR to getting their influenza vaccination. Their eligibility and that their baseline forms are completed will be rechecked prior to randomisation.

Because only 4170 participants are to be recruited over eight hospitals (total ~50,000 employees), it is possible that more staff will be interested in participating than can be included in the trial. Given there will likely be staff who consent but are not randomised (become sick, get their influenza vaccine when study staff are not available, become ineligible, change their mind) we cannot stop recruitment as soon as 4170 participants consent to the trial or we will not reach the required number of participants randomised. Randomisation will need to cease once 4170 participants are randomised. On the consent form and other pre-information, staff will be informed that due to the limited numbers who can be included in the trial, despite consenting, we cannot guarantee they will be randomised.

Given the importance of finding an intervention that can be used early in future pandemics (before a disease-specific vaccine is available), we expect there will be significant interest from researchers to try and understand how BCG works to boost the immune system. To this end, we will include an optional consent for participants to indicate whether they are interested in being approached for other projects.

No identifying information will be provided to the hospital regarding which of their staff consented to be part of the trial.

5 INTERVENTION

5.1 Treatment arms

Intervention group 1: Influenza vaccine ('standard care')
Intervention group 2: Influenza vaccine + BCG vaccine

5.2 Trial Intervention(s)

5.2.1 Description of trial investigational products

5.2.1.1 BCG vaccine SSI

Active substance and excipients	Freeze-dried powder:<br>Live attenuated bacteria of the type *Mycobacterium bovis* BCG (Bacillus Calmette-Guerin), Danish strain 1331<br>0.1 ml vaccine contains between 2 to 8 x 10^5 colony forming units.<br><br>Powder Excipient: Sodium glutamate<br><br>Solvent for resuspension:<br>magnesium sulphate heptahydrate, dipotassium phosphate, citric acid monohydrate, l-asparagine monohydrate, ferric ammonium citrate, glycerol 85%, and water for injections.
Trade or Generic name	BCG Vaccine SSI
Dosage form	Powder for Injection with solvent for resuspension
Route of administration	Intradermal

5.2.2 Dosage

A single dose of BCG vaccine SSI will be given to all participants who are randomised to the intervention group 2. The adult dose is 0.1 mL injected intradermally over the distal insertion of the deltoid muscle onto the humerus (approximately one third down the upper arm).

5.2.3 Dose modification

There are no allowable dose modifications

5.2.4 Storage and dispensing of BCG vaccine SSI

- Store between 2ºC - 8ºC
- Store in the original package in order to protect from light
- Do not freeze
- Do not use the vaccine after the expiry date which is stated on the carton as “EXP” and refers to the last day of the month listed
- Any unused vaccine at the end of the study, meaning vaccines unused after the last dosing of the last participant will be disposed of according to local regulations

5.2.5 Preparation

BCG Vaccine SSI consists of a powder and solvent for suspension for injection (2-8 x 10^5 CFU/0.1 mL dose).

Prior to reconstitution, the LogTag or similar temperature monitoring device in each esky used to transport to and store the vaccines at sites will be checked to ensure the LogTag is flashing green; the flashing green light indicates that the cold chain storage has been maintained.

The rubber stopper must not be wiped with any antiseptic or detergent. In the eventuality of alcohol being used to swab the rubber stopper, it must be allowed to evaporate before the stopper is penetrated with the syringe needle. The BCG is re-suspended using the solvent provided according to the product directions then carefully inverted a few times to produce uniform resuspension of the lyophilised BCG. Do not shake the vial. The investigator who re-suspends the BCG will label the vial with the date, time of reconstitution and their initials.

To ensure a uniform suspension, and therefore dose, the vial will be gently swirled before drawing up each dose. When drawn up into the syringe the reconstituted vaccine should appear homogeneous, slightly opaque and colourless.

Each vial of BCG contains up to 10 adult doses. NEVER administer the whole vial. Each vial can be kept for up to 6 hours after resuspension. During this time the vial is kept at room temperature. Each vial is discarded after 6 hours, or when the vial is empty, whichever occurs first.

5.2.6 Administration of trial drug

The vaccine will only be administered by clinician members of the research team trained in the intradermal vaccination technique.

Both the vaccinator and the participant receiving the vaccine should wear protective eyewear. Eye splashes can ulcerate. If eyes are splashed, wash the eyes with saline or water immediately and contact the the sponsor-investigator (or delegate) to decide on management and follow-up.

Administration of the BCG vaccine will take place at the hospital site so facilities for the treatment of any potential anaphylactic reaction (extremely rare) are available. There will be space to allow for privacy for the staff member if required (e.g. upper left arm not accessible due to clothing).

As per standard practice, participants will be required to remain in the hospital for 20 minutes after vaccination, in case an allergic reaction should occur, wearing a sticker “I have received the BCG vaccine at [time of vaccination]”.

The time and date of resuspension of the vial, batch identifier, immunisation date/time, any issues with immunisation will be entered in the participants’ study record.

Route/method of administration

The injection site should be clean and dry using non-alcohol based antiseptic. Alcohol antiseptics should not be used prior to administration. If alcohol is used to swab the skin, it must be allowed to evaporate before the vaccine is injected. The vaccine must be given

strictly intradermally, approximately one third down the upper arm corresponding to the area of the distal insertion of the deltoid muscle, as follows:

- The skin is stretched between thumb and forefinger
- The needle should be almost parallel with the skin surface and slowly inserted (bevel upwards), approximately 2 mm into the superficial layers of the dermis. The needle should be visible through the epidermis during insertion
- The vaccine should be given slowly

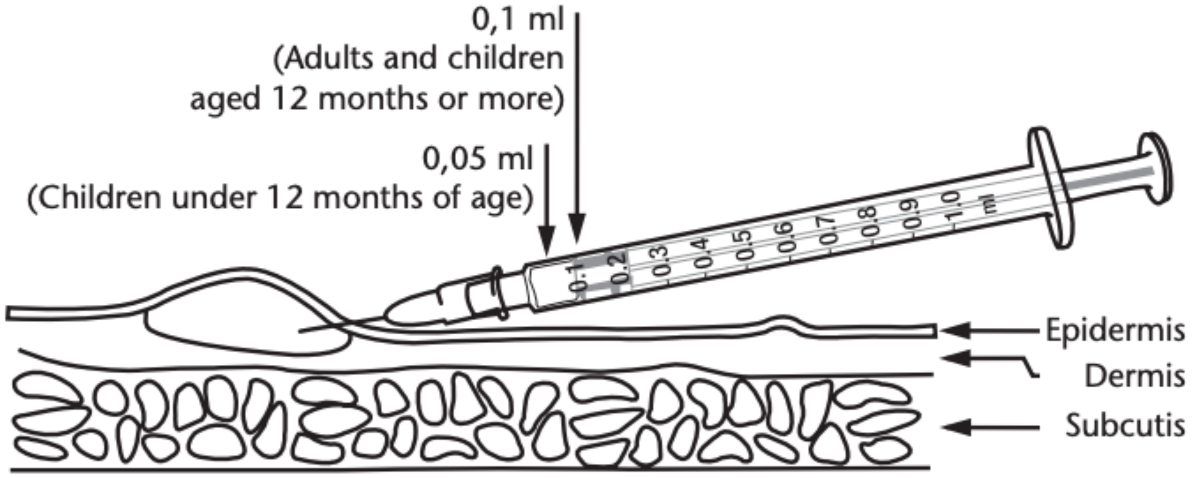

- The mixed vaccine should be administered with a syringe of 1 ml graduated into hundredths of millilitre (1/100) fitted with a short bevel syringe needle (25G or 26G).
- You should feel considerable resistance as you give the injection. If there is no resistance, the needle may be in the subcutaneous tissues.
- If the injection is not intradermal, withdraw the needle and repeat at a new site.
- A raised, blanched papule/bleb of about 7 mm diameter (looks like orange peel) at the needle point is a sign of correct injection
- The injection site is best left uncovered to facilitate healing
- Jet injectors or multiple puncture devices should not be used to administer the vaccine.

Over/under dosage or incorrect administration

Overdose increases the risk of suppurative lymphadenitis and may lead to excessive scar formation. Gross over dosage increases the risk of undesirable BCG complications. Deep injections increase the risk of lymphadenitis and abscess formation.

The clinician members of the research team who administers BCG as part of this trial will be required to document whether the BCG vaccination was given ‘perfectly’ with appropriate bleb. Any variations will be documented, and standard procedures followed regarding the need for re-administration, notification to Sponsor-Investigator (or delegate).

Complications

All BCG-related complications will be referred to the sponsor-investigator for advice regarding management. In the very unlikely event a participant has a systemic infection of

Mycobacterium bovis or persistent local infection following vaccination Prof Nigel Curtis (or delegate) will provide advice to the local treating team regarding management, including antibiotic treatment choice.

5.2.7 Product accountability

RCH pharmacy will act as the study central pharmacy and co-ordinate the storage, distribution and maintain accountability records of the BCG vaccine supply. Trial documentation of storage, dispensation and destruction if required will be maintained in the pharmacy file.

BCG will be delivered to the RCH pharmacy and stored appropriately for the duration of the study before kits are dispensed to hospital sites for vaccination sessions. Study supplied vaccines will be dispensed as packs of 10 vials of BCG with solvent for resuspension to each site per day of recruitment and randomisation. Each pack will be labelled with a simple batch identifier if needed (developed by RCH pharmacy).

Each morning during the randomisation period, the following is dispensed to the study team for each site:

- 1 study labelled esky with icepacks
- 1 pack of 10 BCG vials and solvent for resuspension
- LogTag temperature monitoring device
- Bubble wrap as required.
- A cold chain verification documentation form to log the study drug transfers from the central pharmacy to each site and to track unused study vaccines and solvent back to the central pharmacy after the completion of randomisation sessions on each day.

In the event that the return of the unused vials and solvent occurs after the usual operational hours of the central pharmacy the packs will be transferred for overnight storage to the Infectious Disease monitored fridge in MCRI (level 5 laboratories), LogTags will remain operational and the packs will be returned as soon as possible to the central pharmacy. The MCRI infectious diseases fridge is in a locked area. The Sponsor-Investigator will delegate access to a restricted number of investigators.

Unused Vials may be used for multiple sessions if the cold chain storage has been maintained.

Any reason for departure from the expected dispensing regimen will be recorded. At the end of the trial, there will be final reconciliation of trial drug received, dispensed, used and returned. Any discrepancies will be investigated, resolved and documented by the trial team.

5.2.8 Excluded medications and treatments

BCG vaccination may be given concurrently with inactivated or live vaccines. If not given concurrently a theoretical period of not less than 4 weeks must pass before giving another live vaccine (although there is no real data supporting this precaution). There must be an interval of at least 3 months before a vaccination in the same arm can take place.

5.2.9 Discontinuation from trial intervention

The trial intervention is a once-off vaccination. Due to this there is no possibility to 'discontinue the trial intervention'. If a participant changes their mind between randomisation and vaccination, deciding that they do not want to have the vaccination (but are happy to continue in the study for the follow-up period) they will be included in the analysis as intention to treat.

6 RANDOMISATION AND BLINDING

Once consent has been obtained, and following baseline assessment, participants will be enrolled and randomised on the day of the vaccination via Redcap. Randomisation will be to Intervention group 1 and intervention group 2 with an allocation ratio of 1:1, using a web-based randomisation procedure. The randomisation schedule and web-based service will be provided by an independent statistician from the Clinical Epidemiology and Biostatistics Unit (CEBU) at the Murdoch Children's Research Institute. Randomisation will be in randomly permuted blocks of variable length. Randomisation will be stratified by study site, by age (<40 years; 40 to 59 years; >=60 years) and by presence of comorbidity (any of diabetes, chronic condition, respiratory disease, cardiac condition, hypertension). Stratification by age is necessary for data analysis because older ages are associated with a greater likelihood of developing severe COVID-19. Likewise, presence of comorbidity is associated with a greater risk of developing severe COVID-19. Each study site will have their own randomisation list stratified by age and presence of comorbidity.

6.1 Concealment mechanism

The control group (intervention group 1) will have no placebo. Most people vaccinated with the BCG vaccine develop a papule/blister at the injection site around two-weeks after vaccination. Due to this, even with a placebo, it is not possible to blind participants to their treatment group allocation. The outcomes (prevalence of COVID-19 disease or admission to hospital for COVID-19 disease) are objective, so we do not expect participants awareness of their group allocation to bias the study results significantly. Members of the research team doing the analysis will be blinded to group allocation (by the removal of this variable and all other variables related to BCG from the dataset) until the formal detailed statistical analysis plan is confirmed and signed by all investigators and all data cleaning/preparation is complete.

7 TRIAL VISITS AND PROCEDURES

7.1 Trial timeline

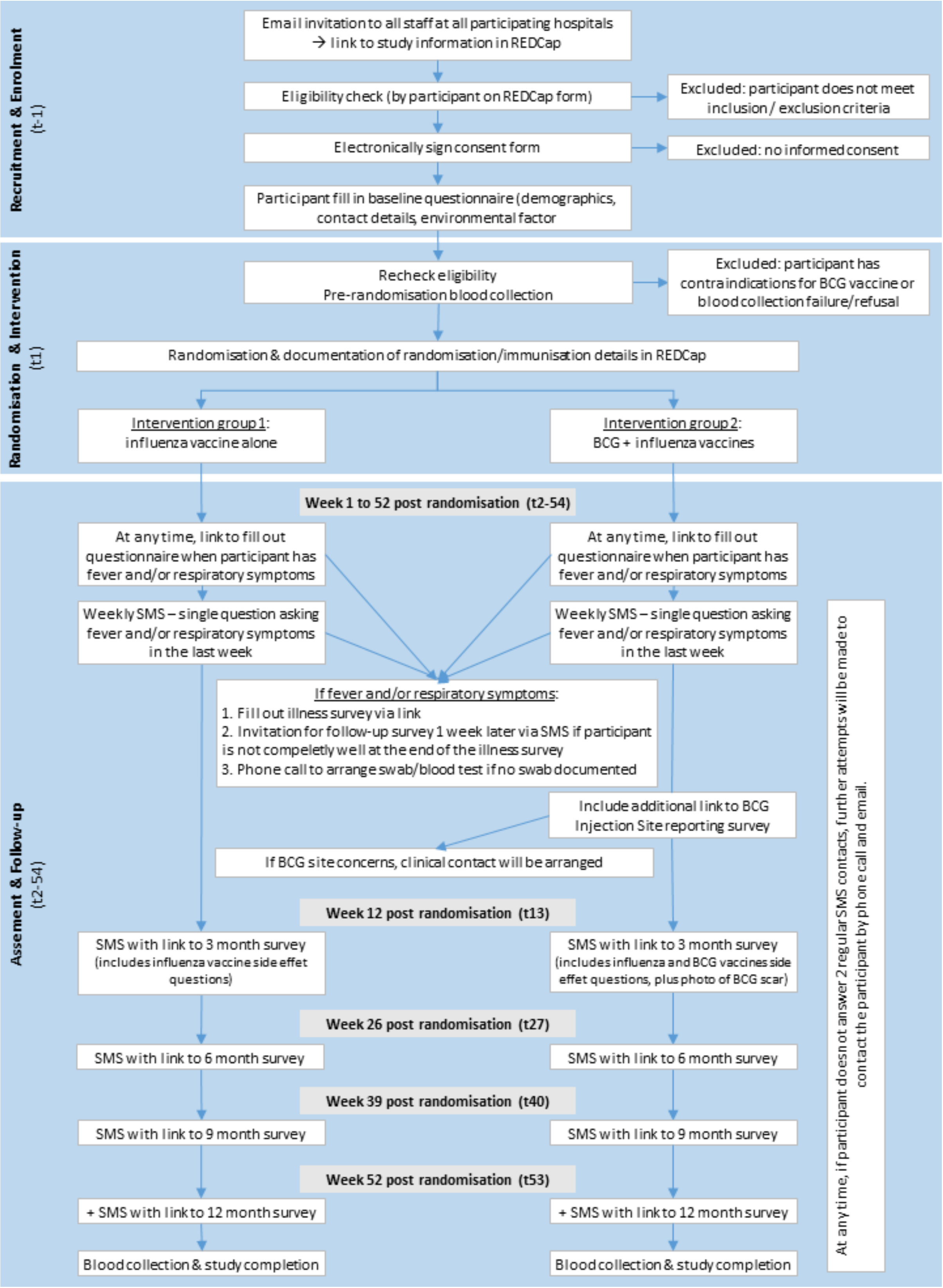

7.2 Schedule of assessments

	TRIAL PERIOD										
	Enrolment	Inclusion & randomisation	Post-randomisation								Close-out
TIME POINT	t_{-1}	t_1	t_{2-12}	t_{13}	t_{14-26}	t_{27}	t_{28-39}	t_{40}	t_{41-52}	t_{53}	t_{53}
ENROLMENT:											
Eligibility screen	X										
Informed consent	X										
Contact details	X										
Allocation to intervention		X									
INTERVENTIONS:											
Influenza vaccine		X (both groups)									
BCG vaccine		X (group 2)									
ASSESSMENTS:											
Baseline questionnaire	X	X									
Weekly survey			X	X	X	X	X	X	X	X	
Swab *(if indicated by weekly survey)*			X	X	X	X	X	X	X	X	
3-month survey				X							
6-month survey						X					
9-month survey								X			
12-month survey										X	
Clinical advice on BCG injection site *(if indicated)***			X	X							
*Blood collection**		X									X

*Study nurse
** Infectious Diseases clinician

7.3 Description of procedures

The procedures related to recruitment, consent, eligibility confirmation and randomisation are described in section 4 of this protocol.

After randomisation there are 2 key aspects of the 1-year follow-up period; questionnaires and sample collections for SARS-CoV-2 identification (respiratory swabs or blood samples). Participants will be asked to complete a questionnaire and self-collect a respiratory swab each time they have a febrile illness with a respiratory symptom.

<u>Questionnaires</u>

Baseline

- Comorbidities: diabetes, cardiovascular disease, chronic respiratory disease, hypertension
- Risk factors: smoking
- BCG/TB history: Prior BCG vaccination, ever positive TST
- Other: Recurrent herpes infection (such as cold sores)

Up to Weekly and/or Ad-hoc, for each episode of illness;

- Any symptoms of COVID/19: fever with at least one sign or symptom of respiratory disease such as cough, shortness of breath, respiratory distress/failure, runny/blocked nose (y/n)
- Has a COVID-19 swab been taken? (if so what was the result)
- Date of/days since onset and cessation of symptoms
- Which Symptoms: fever, cough, shortness of breath, respiratory distress/failure, runny/blocked nose
- Days absent from work (total number and number due to illness)
- ED presentations
- Hospital admission (oxygen, ICU admission, mechanical ventilation)
- Known test results
- If a swab has been taken for clinical purposes, who ordered it
- Impact on daily activities (using scores yet to define)
- Days in bed
- Chest x-ray results

Periodic questionnaires (once every 3 months)

- Exposure (average days working in hospital)
- Cold Sore recurrence

Additional questions for 3rd month questionnaire only:

- Safety and BCG side effects (photo of BCG injection site)
- Influenza vaccine side effects

Swabs

- Where a participant has had a swab sample assessed outside of the study (e.g. at VIDRL) results from centralised SARS-CoV-2 testing sites will be obtained.
- Where a participant has symptoms of febrile respiratory illness (cough, shortness of breath, respiratory distress/failure, runny/blocked nose (y/n)) and swab sample is not assessed for SARS-CoV-2 through standard pathway (per example due to swab shortage, or government decision to restrict screening to high-risk patients), a sample collection study visit will be done within 7 days of the onset of symptoms. A member of the study team will visit the participant’s home and collect a respiratory swab sample (nasal plus throat). The sample will be labelled with participant ID, date/time collected (no identifying information), transported to the MCRI laboratory (or associated study site) and stored at -80˚C for future SARS-CoV-2 testing. As testing will be done weeks-months after sample collection, the results will not inform clinical care.

Blood samples

- At randomisation, will be taken for later assessment of seroconversion (production of specific anti-SARS-CoV-2 antibodies). This will identify participants who had SARS-CoV-2 exposure and immunity prior to commencement of the study.
- At 12 months (+/- 2 months) post randomisation, the study team will go to each hospital to collect end of study blood samples. This will identify participants who had

an immune response to SARS-CoV-2 (surrogate marker of infection) during the study. This is needed to determine asymptomatic SARS-CoV-2 infections.

- After febrile respiratory illness (if SARS-CoV-2 swab samples cannot be taken). In the eventuality that it is unfeasible to collect swab samples to confirm SARS-CoV-2 infection at the time of febrile respiratory illness episodes, seroconversion may be used to associate episodes of febrile respiratory illness with SARS-CoV-2 infection. Therefore, for every episodes of febrile respiratory illness where a swab sample cannot be taken, a member of the study team will visit the participants home 1 month after the onset of symptoms (expected peak post-infection antibody production) to collect a blood sample.

For all blood sample collection, a venous blood sample (up to 10 ml) will be taken by a trained member of the study team and labelled with participant ID, date/time collected (no identifying information). Samples will be transported to the laboratories at the MCRI (or laboratories at a study site). Samples will be processed for serum separation and stored at -80˚C for later assessment.

Data Retrieval

Data retrieval and linkage is further described in Section 9 of this Protocol.

The present study expects that it will acquire some research data from existing administrative and service data sources. This would include obtaining details from the Department of Human Services (DHS) who hold Medicare Benefits Scheme (MBS) and Pharmaceutical Benefits Scheme (PBS) and the Victorian Department of Health and Human Services (VDHHS) who collects information about presentations to hospitals and emergency departments for medical care in Victoria.

Collecting this information will help us to determine the cost effectiveness of the intervention and help us measure the outcomes at the end of the study.

7.4 Notes on specific trial visits

7.4.1 Unscheduled visit

If participants have any concerns related to side effects or the BCG scar, they can call the study team for advice and if necessary, they will be seen at the Royal Children's Hospital by the sponsor-investigator or his delegate.

7.5 Treatment discontinuation, participant withdrawals and losses to follow up

7.5.1 Discontinuation of treatment - participant remains in trial for follow up

The trial intervention is a once-off vaccination. Due to this there is no possibility to 'discontinue the treatment'. If a participant changes their mind between randomisation and vaccination, deciding that they do not want to have the vaccination (but are happy to continue in the study for the follow-up period) they will be included in the analysis as intention to treat.

7.5.2 Withdrawal of consent - participant withdraws from all trial participation

Participants are free to withdraw from the trial at any time upon their request. Withdrawing from the trial will not affect their access to standard treatment or their employment as their participation will not be shared with their employer.

For the safety of all participants ceasing trial treatment, reasonable efforts should be made to undertake protocol-specified safety evaluations to capture new safety events and to assess existing, unresolved safety events following withdrawal.

A dedicated Case Report Form (CRF) page will be used to capture the date of participant withdrawal of consent.

7.5.3 Losses to follow-up

Due to the study taking place in health care workers during a pandemic, we expect that there may be periods that participants will ignore our SMS contacts. This includes the eventuality that a participant has been admitted to hospital. The weekly SMS contacts will only ask whether the participant has had a fever or respiratory symptom since the last time they answered the SMS (date provided). We deem this very unlikely to annoy participants excessively as they can ignore the text message if they are too busy (or withdraw by replying to the SMS). This will give the project the best chance of having a complete dataset to analyse as they can answer 'Yes' when they get the opportunity and fill in the associated questionnaire. Therefore, we will continue to send out weekly SMS for the entire study regardless of whether they respond.

If a participant does not answer 2 regular SMS contacts (2 consecutive weeks), further attempts will be made to contact them by phone call (maximal 5 attempts) and email (maximal 2 attempts). If there is still no response, and the participant is not found to have died on medical records, we will continue.

7.5.4 Replacements

Participants who have been randomised may NOT be replaced.

7.5.5 Trial Closure

A participant is considered to have completed the trial if he or she has completed all phases of the trial including the last visit or the last scheduled procedure shown in the Schedule of Assessments.

The end of the trial is defined as completion of the last visit or procedure shown in the Schedule of Assessments in the trial at all sites. At this stage, the Sponsor-Investigator will ensure that all HRECs and RGOs as well as all regulatory and funding bodies have been notified.

This trial may be temporarily suspended or prematurely terminated if there is sufficient reasonable cause. If the trial is prematurely terminated or suspended, the Sponsor-Investigator will promptly inform trial participants, HREC and RGO, the funding (where applicable) and regulatory bodies, providing the reason(s) for the termination or suspension.

Circumstances that may warrant termination or suspension include, but are not limited to:

- Determination of an unexpected, significant, or unacceptable risk to participants that meets the definition of a Significant Safety Issue (SSI) (for the definition refer to Section 8.1).
- Insufficient compliance to protocol requirements
- Data that are not sufficiently complete and/or evaluable
- Demonstration of efficacy that would warrant stopping
- Determination that the primary endpoint has been met
- Determination of futility

In the case of concerns about safety, protocol compliance or data quality, the trial may resume once the concerns have been addressed to the satisfaction of the sponsor, HREC, RGO, funding and/or regulatory bodies.

7.5.6 Continuation of therapy

As the treatment is 'once-off' there is no provision for continuation of therapy.

8 SAFETY MONITORING AND REPORTING

8.1 Definitions for use in trials involving investigational medicinal products

The text below uses the definitions listed in NHMRC Guidance: Safety monitoring and reporting in clinical trials involving therapeutic goods (dated November 2016) https://www.nhmrc.gov.au/guidelines-publications/eh59

Note that adverse events and adverse reactions to investigational medical products are classified as non-serious (AE and AR) or serious (SAE or SAR). See full terms and definitions below.

8.2 Definitions

Participant-specific adverse events

Adverse Event (AE):
Any untoward medical occurrence in a participant administered a medicinal product and does not necessarily have a causal relationship with the study treatment.

Adverse Reaction (AR):
Any untoward and unintended response to an investigational medicinal product related to any dose administered.

Comment: All adverse events judged by either the reporting investigator or the sponsor as having a reasonable possibility of a causal relationship to a study vaccine would qualify as adverse reactions. The expression 'reasonable causal relationship' means to convey, in general, that there is evidence or argument to suggest a causal relationship.

Serious Adverse Event (SAE) / Serious Adverse Reaction (SAR):
Any adverse event/adverse reaction that:

- Results in death; or
- Is life-threatening; or
- Requires hospitalisation or prolongation of existing hospitalisation; or
- Results in persistent or significant disability/incapacity

Note: Life-threatening refers to an event in which the participant was at risk of death at the time of the event. It does not refer to an event that hypothetically might have caused death if it were more severe.

Medical and scientific judgement should be exercised in deciding whether an adverse event/reaction should be classified as serious in other situations. **Important medical events** that are not immediately life-threatening or do not result in death or hospitalisation but may jeopardise the participant or may require intervention to prevent one of the other outcomes listed in this definition should also be considered serious.

Suspected Unexpected Serious Adverse Reaction (SUSAR):
An adverse reaction that is both serious and unexpected.

Safety issues (requiring expedited reporting)
The following definitions describe additional safety events that require expedited reporting to stakeholders including the Sponsor, Investigators, HREC, local governance office and TGA.

Significant Safety Issue (SSI):
A safety issue that could adversely affect the safety of participants or materially impact on the continued ethical acceptability or conduct of the trial.
Comment: A SSI is a new safety issue or validated signal considered by the Sponsor in relation to the study vaccines that requires urgent attention of stakeholders. This may be because of the seriousness and potential impact on the benefit-risk balance of the study vaccines, which could prompt regulatory action and/or changes to the overall conduct of the clinical trial, including the monitoring of safety and/or the administration of the study vaccines.

Urgent Safety Measure (USM):
A measure required to be taken in order to eliminate an immediate hazard to a participant's health or safety. Note: This is a type of SSI that can be instigated by either the investigator or sponsor and can be implemented before seeking approval from HRECs or institutions.

8.3 Capturing and eliciting adverse event/reaction information

Adverse events and adverse reactions (non-serious and serious) will be captured from the period participant is vaccinated until the 3-month follow-up questionnaire. The participant will have access to a telephone number and email address to contact a member of the research team to inform of a suspected adverse event until 3 months post study vaccination and will be followed until resolution or stabilisation.

At the three-month questionnaire we will ask participant if they have concern about their vaccination site. They will also be asked if they have been hospitalised, had any accidents, used any new medication. In addition, AEs will be documented from physical examination findings, clinically significant laboratory results or other documents (including correspondence from their primary care physician) that are relevant to participant safety.

8.4 Documentation of AEs

For the purposes of this study the investigator or delegate is responsible for recording all Adverse Events, regardless of their relationship to study vaccines, with the following exceptions:

- Conditions that are pre-existing at enrolment and do not deteriorate will not be considered adverse events.
- Abnormal laboratory values will not be considered adverse events unless deemed clinically significant by the investigator and documented as such.

The documentation of each AE on the CRF will include:

- A description of the AE
- The onset date, duration, date of resolution
- Severity (mild, moderate or severe – what is the impact on the participant's daily life?)
- Seriousness (SAE or not)
- Any action taken (e.g. treatment, follow-up tests)
- The outcome (recovery, death, continuing, worsening)
- The likelihood of the relationship of the AE to the trial treatment (Unrelated, Possible, Probable, Definite)

Changes in the severity of an AE will be reported. AEs characterised as intermittent will be documented for each episode. All AEs will be followed to adequate resolution, where possible.

8.5 Assessing the seriousness of a participant's AE

The seriousness of an AE will be assessed by an investigator according to the definition in in the preceding section on definitions with the following exception:

- Elective surgery planned at the time of enrolment.
- Hospitalisation due to progression of disease will not be considered an SAE for the purposes of this trial.

8.6 Assessing the relatedness (causality) of a participant's AE

All adverse events/serious adverse events must have their relationship to the trial intervention assessed by the Investigator who evaluates the adverse event based on temporal relationship and his/her clinical judgment. The degree of certainty about causality will be graded using the categories below. In a clinical trial, the intervention should always be suspected.

The relationship of the event to the trial intervention will be assessed as follows:

Code	Causal Relationship		Description
1	Unrelated	Unrelated	The AE is clearly NOT related to the intervention
2	Unlikely		The AE is doubtfully related to the intervention
3	Possible	Related	The AE may be related to the intervention

4	Probable		The AE is likely related to the intervention
5	Definite		The AE is clearly related to the intervention

8.7 Assessing the severity of a participant's AE

The Site Investigator will be responsible for assessing the severity of an adverse event (AE). The determination of severity for all adverse events should be made by the investigator based upon medical judgment and the severity categories of Grade 1 to 5 as defined below, with the following exception:

- Hospitalisation due to progression of COVID-19 disease will not be considered an SAE for the purposes of this study.
- Hospitalisation due to progression of febrile, respiratory illness will not be considered an SAE for the purposes of this study.

Grade	Severity	Description
Grade 1	Mild	Asymptomatic or mild symptoms; clinical or diagnostic observations only; intervention not indicated
Grade 2	Moderate	Moderate; minimal, local or non-invasive intervention indicated; limiting age appropriate instrumental activities of daily living (ADL)
Grade 3	Severe	Severe or medically significant but not immediately life-threatening; hospitalisation or prolongation of hospitalisation indicated; disabling; limiting self-care ADL
Grade 4	Life Threatening	Life-threatening consequences; urgent intervention indicated
Grade 5	Fatal	Death related to AE

8.8 Reporting of safety events

<u>Site Principal Investigator Reporting Procedures</u>

The Site Principal Investigator or delegate is responsible for recording all safety events in the source document.

The Investigator is responsible for expedited reporting (within 24 hours of becoming aware of the event) to the Sponsor-Investigator the following local safety events:

1. USMs
2. SUSARs
3. All SAEs /SAR

The Site Principal Investigator is responsible for reporting SAEs (including SUSARs) to the Sponsor-Investigator as soon as possible but within 24 hours of the first knowledge of the event. These reports should be submitted using the trial Expedited Safety Report Form.

The Site Principal Investigator is also responsible for reporting SSIs, local USMs and local SUSARs to their research governance office within 72 hours of becoming aware of the event and in accordance with their local governance authorisation.

Sponsor-Investigator Reporting Procedures
The Sponsor-Investigator must assess and categorise the Expedited Safety Reports received from Investigators and report these to all Site Principal Investigators, the RCH HREC and TGA in accordance with the NHMRC's 'Safety monitoring and reporting in clinical trials involving therapeutic goods' (November 2016) and any additional requirements of the RCH HREC. All safety reports must clarify the impact of the safety event on participant safety, trial conduct and trial documentation.

The Sponsor-Investigator is responsible for the following reporting to PIs, the RCH HREC and TGA:

1. All SSIs that meet the definition of a USM within 72 hours of becoming aware of the issue.
2. All other SSIs within 15 calendar days of instigating or becoming aware of the issue
3. For SSIs leading to an amendment of trial documentation:
    a. Submit details of the SSI without undue delay and no later than 15 calendar days of becoming aware of the issue.
    b. Submit amendment to the HREC without undue delay.
4. For SSIs leading to temporary halt or early termination of a trial for safety reasons:
    a. Communicate reasons, scope of halt, measures taken, further actions planned without undue delay and no later than 15 calendar days of decision to halt.
    b. For a temporary halt, notify the PIs, HREC and TGA when the trial restarts, including evidence that it is safe to do so.

The Sponsor will also report SUSARs to the TGA as follows:

1. Fatal or life-threatening SUSARs immediately, but no later than 7 calendar days after being made aware of the issue (follow up info within a further 8 calendar days)
2. All other SUSARs no later than 15 calendar days of being made aware of the issue

The Sponsor-Investigator is responsible for providing the additional safety information to the RCH HREC:

Provide any updated Product Information/Investigator's Brochure for the study vaccines

9 DATA AND INFORMATION MANAGEMENT

9.1 Overview

The Principal Investigator is responsible for storing essential trial documents relevant to data management and maintaining a site-specific record of the location(s) of the site's data management-related Essential Documents.

The Principal Investigator is responsible for maintaining adequate and accurate source documents that include all key observations on all participants at their site. Source data will

be attributable, legible (including any changes or corrections), contemporaneous, original, accurate, complete, consistent, enduring and available. Changes to source data (hardcopy and electronic) must be traceable, must not obscure the original entry, and must be explained where this is necessary. A site-specific Source Document Plan will be maintained to indicate the location(s) of source documents.

The Principal Investigator will also maintain accurate case report forms (CRFs) (i.e. the data collection forms) and be responsible for ensuring that the collected and reported data is accurate, legible, complete, entered in a timely manner and enduring. To maintain the integrity of the data, any changes to data (hardcopy and electronic) must be traceable, must not obscure the original entry, and must be explained where this is necessary.

Any person delegated to collect data, perform data entry or sign for data completeness will be recorded on the delegation log and will be trained to perform these trial-related duties and functions.

9.2 Data management

9.2.1 Data generation (source data)

In this study, the following types of data will be collected:

- personal identifying information (names, dates of birth, contact details)
- sensitive information including health data (medical history, participant eligibility, adverse reactions and other notes as appropriate)
- participant completed electronic questionnaires
- de-identified data from laboratory assays

Source document plan

The source documents for this study will include, questionnaires completed by the participant's or researcher; recorded data from automated instruments, laboratory reports and the signed information and consent forms. Each site participating in the trial will maintain a site-specific Source Document Plan that will document the source, i.e. original recording, for each data discrete item/ category of items collected for the study. This Source Document Plan, signed and dated by the Site Principal Investigator, will be prepared prior to recruitment of the first participant and will be filed in the site's Investigator Site File.

9.2.2 Data capture methods and data use, storage, access and disclosure during the trial

Data collection methods
Data for this trial will be collected and entered using electronic database REDCap. REDCap is a secure, web-based application for building and managing online surveys and databases.

Use of the data
The data will be used for the analyses specified in the protocol and Statistical Analysis Plan.

Following the completion and analysis of the trial, the data will be retained long-term following the mandatory archive period for use in future research projects.

<u>Storage and access</u>
Hard copy data will be stored by at MCRI Infectious diseases laboratories in a locked cabinet in a secure location, accessible to the research team only.

Electronic data will be securely stored in MCRI's REDCap database system and in files stored in MCRI's network file servers, which are backed up nightly. Files containing private or confidential data will be stored only in locations accessible only by appropriate designated members of the research team.

REDCap is hosted on MCRI infrastructure and is subject to the same security and backup regimen as other systems (e.g. the network file servers). Data is backed up nightly to a local backup server, with a monthly backup taken to tape and stored offsite. REDCap maintains an audit trail of data create/update/delete events that is accessible to project users who are granted permission to view it. Access to REDCap will be provided via an MCRI user account or (for external collaborators) via a REDCap user account created by the MCRI system administrator. The permissions granted to each user within each REDCap project will be controlled by, and will be the responsibility of, the trial team delegated this task by the Principal Investigator. REDCap has functionality that makes adding and removing users and managing user permissions straightforward. All data transmissions between users and the REDCap server are encrypted. The instructions for data entry to REDCap must be read and the training log signed prior to personnel commencing data entry on REDCap.

Authorised representatives of the sponsoring institution as well as representatives from the HREC, Research Governance Office and regulatory agencies may inspect all documents and records required to be maintained by the Investigator for the participants in this trial. The trial site will permit access to such records.

<u>Disclosure</u>

The trial protocol, documentation, data and all other information generated will be held in strict confidence. No information concerning the study or the data will be released to any unauthorised third party, without prior written approval of MCRI. Clinical information will not be released without written permission of the participant, except as necessary for monitoring by the HREC, Research Governance Office or regulatory agencies.

9.2.3 Data confidentiality

<u>Data confidentiality</u>
Participant confidentiality is strictly held in trust by the Site Principal Investigator, participating investigators, research staff, and the Murdoch Children's Research Institute (MCRI) and their agents. This confidentiality is extended to cover testing of biological samples in addition to the clinical information relating to participating participants.

To preserve confidentiality and reduce the risk of identification during collection, analysis and storage of data and information, the following will be undertaken:

(1) The number of private/confidential variables collected for each individual has been minimised. The data collected will be limited to that required to address the primary and secondary objectives

(2) Participant data and samples will be identified through use of a unique participant study number assigned to the study participant ("re-identifiable").

The Site Principal Investigator is responsible for the storage of a master-file of names and other identifiable data with the participant ID; access to this document will be restricted to members of the research team and authorised persons as listed previously. The master file will be stored securely, and separately, from study data in locked/ password-protected databases with passwords kept separately.

(3) Separation of the roles responsible for management of identifiers and those responsible for analysing content. The data will be analysed by members of the research team, who will be provided with anonymised data identified only by the unique participant study ID.

9.2.4 Quality assurance

A REDCap data dictionary with range checks will be used to minimise data entry errors, such as out-of-range values. Data quality control checks (e.g. checking for invalid characters, invalid dates, data that is not consistent with data in other data fields) and data cleaning will be done by trained members of the research team on a regular basis. Any discrepancies will be reported to the CPI or delegate and addressed in a timely manner.

Quality control checks will be run by the data clerk, on a weekly basis, who will highlight any queries to the principal investigator. Data will only be uploaded onto the master database once any queries highlighted by quality control checks have been resolved.

9.2.5 Archiving - Data and document retention

Data will be stored securely on MCRI server (restricted access) and/or locked in secure cabinet in MCRI laboratories (for hardcopy data) for 15 years after study completion, in accordance with the requirements of the Therapeutic Goods Administration and Health Privacy Principles.

Prof Nigel Curtis (CPI) will be the custodian during the archive period, and members of the research team will have access to the stored data. At the end of the archival period, long-term retention of the data may occur, as per section 10.2.6

Records should not be destroyed without the written consent of the Sponsor Investigator. The Sponsor-Investigator will inform Site Principal Investigators when these documents no longer need to be retained.

9.2.6 Data sharing

Data sharing

Beginning 6 months following analysis and article publications, the following may be made available long-term for use by future researchers from a recognised research institution whose proposed use of the data has been ethically reviewed and approved by an independent committee and who accept MCRI's conditions, under a collaborator agreement, for accessing:

- Individual participant data that underlie the results reported in our articles after de-identification (text, tables, figures and appendices)
- Study protocol, Statistical Analysis Plan, PICF

Long-term custodianship (after archive period finished)

Prof Nigel Curtis will be the long-term custodian following the archive period.

9.2.7 Data retrieval and linkage

The present study expects that it will acquire some research data from existing administrative and service data sources. In some instances, participant consent may allow retrieval of datasets without the need for linkage keys, as has usually been the case of other MCRI studies.

For datasets from government sources requiring linkage keys, the study will primarily work with the Centre for Victorian Data Linkage and Australian Institute of Health and Welfare. For private sources such as pathologists, the study will establish appropriate initiatives.

We anticipate data linkage and access will occur after the study recruitment period is complete, but the exact timing is yet to be determined. Working with both the capabilities of the data linkage services and through consultation with research studies that have extensive data linkage experience, this study will establish IT systems and SOPs to support data linkages processes that are efficient and minimise the risk of disclosure. These processes will use data linkage keys to separate the personally identifiable information needed for data linkage from the administrative and clinical data being sourced.

9.2.8 Sample management: Specimen & Biobanking

All samples that are not used immediately for the laboratory assessments described in previous sections, may be cryopreserved for an indefinite period of time to enhance the possible benefit from this study, by providing a sample biobank that may be used for research related to immunology or infectious diseases, in the future. The biobank will be at MCRI laboratories (Infectious Diseases Group) in Melbourne, (please see Appendix 3 for Biobank Registration Form). The biobank will be registered with the Melbourne Children's Bioresource Centre (MCBC). Written informed permission (extended consent) for banking of specimens and future use for study objectives without further consent will be obtained from the participant. These samples may be used for additional research studies related to immunology or infectious diseases. For tests that require equipment or technical expertise not available in Melbourne, select specimens may be sent to collaborating laboratories outside of Melbourne (interstate and/or overseas) for further testing.

Databank is defined as: "A systematic collection of data, whether individually identifiable, re-identifiable or non-identifiable" (NHMRC National Statement on Ethical Conduct in Human Research)

Biobank is defined as: ".... collections of human biological materials (biospecimens) linked to relevant personal and health information (which may include health records, family history, lifestyle and genetic information) and held specifically for use in health and medical research." (NHMRC Biobanks Information Paper 2010)

10 TRIAL OVERSIGHT

10.1 Governance structure

10.1.1 Trial Management Group (TMG)

The Chief Principal Investigator is responsible for supervising any individual or party to whom they have delegated tasks at the study site. They must provide continuous supervision and documentation of their oversight. To meet this Good Clinical Practice requirement, a group will be responsible for the day-to-day management of the study and will include at a minimum the Site PI, study coordinator and research nurse. The group will closely review all aspects of the conduct and progress of the study, ensuring that there is a forum for identifying and addressing issues. Meetings will be minuted with attendees listed, pertinent emails retained and phone calls documented.

10.1.2 Trial Steering Committee (TSC)

The trial steering committee will be made up of the investigators of the project. They will meet at a minimum; weekly until the end of the randomisation period, monthly until the end of participant follow-up and then as required until the study is complete.

10.1.3 Independent Data and Safety Monitoring Board (DSMB)

In this trial the intervention will occur over approximately 1 month and primary outcome data will be collected over 6-12 months. This reduces the capacity for a formal data & safety monitoring committee to advise in recruitment matters based on outcome data. Nevertheless an independent Data and Safety Monitoring Board (DSMB) will be convened to review the outcome data and safety at 3 and 6 months post initial recruitment. It will be composed of individuals with the appropriate expertise, including at least three independent clinicians and/or biostatisticians who, collectively, have experience in the management of biostatistics and the conduct and monitoring of randomised controlled trials. Members of the DSMB will be independent of trial conduct. The DSMB will review data from each intervention group of the trial. At each meeting, a descriptive summary of withdrawals, serious adverse events and non-serious adverse events thought to be related to the study drugs will be provided, along with outcome data for the primary outcomes. The DSMB will provide its input to the trial investigator.

10.1.4 Independent Safety Monitor

During the recruitment phase an independent safety monitor will review all adverse events on a weekly basis and report any concerns to the Sponsor-Investigator. This role will cease once recruitment is complete.

10.1.5 Quality Control and Quality Assurance

Both the Sponsor-Investigator and Site Investigator have responsibilities in relation to quality management.

The Sponsor-Investigator will develop SOPs that identify, evaluate and control risk for all aspects of the study, e.g. study design, source data management, training, eligibility, informed consent and adverse event reporting. The Sponsor-Investigator will also implement quality control (QC) procedures, which will include the data entry system and data QC checks. Any missing data or data anomalies will be communicated to the site(s) for clarification/resolution.

As outlined in the previous section (Site Monitoring), the study monitor will verify that the clinical trial is conducted and data are generated and biological specimens are collected, documented (recorded), and reported in compliance with the protocol, good clinical practice and applicable regulatory requirements.

In the event of non-compliance that significantly affects human participant protection or reliability of results, the Sponsor-Investigator will perform a root cause analysis and corrective and preventative action plan (CAPA).

In addition, each clinical site will perform internal quality management of study conduct, data and biological specimen collection, documentation and completion. An individualised quality management plan will be developed to describe a site's quality management.

11 STATISTICAL METHODS

11.1 Sample Size Estimation

4170 health care workers will be enrolled and randomly allocated in a 1:1 ratio to BCG vaccine group (n=2085), and to control (n=2085). Sample size was calculated based on the two primary outcomes of (1) number of participant COVID-19 disease and (2) number of participant with severe COVID-19 disease. Since the study aims to assess two primary outcomes, an adjustment for multiplicity will be applied to maintain a global Type I error rate of 5%.

For the primary outcome (1) number of participants with COVID-19 disease: it is conservatively estimated that a proportion of 55% of subjects will be infected by COVID-19 disease in the control group; applying a 1:1 ratio for randomisation, a total sample size of n=2016 (1008 per group) will provide 95% power with 2-tailed 0.005 significance level (10% of the global significance level) for the Pearson chi-square test to detect an absolute difference of 10% between a prevalence of COVID-19 disease of 45% in the BCG vaccine group and 55% in the control group.

For the primary outcome (2) number of participants with severe COVID-19 disease: it is estimated that a proportion of 4% of subjects will be infected by severe COVID-19 disease in the control group; applying a 1:1 ratio for randomisation, a total sample size of n=3336 (1668 per group) will provide 90% power with 2-tailed 0.045 significance level (90% of the global significance level) for the Pearson chi-square test to detect an absolute difference of 2% between a prevalence of severe COVID-19 disease of 2% in the BCG vaccine group and 4% in the control group.

Allowing for a 20% loss to follow up, it is planned the study will recruit **4170** health care workers, resulting in approximately 2085 participants to each group.

11.2 Population to be analysed

The primary analysis of all outcome data will be an intention-to-treat (ITT) analysis including all randomised participants, regardless of whether they received trial drug.

11.2.1 Handling of missing data

For the primary analysis the imputation of missing data will only be considered if 10-20% of the primary outcome is missing and will be undertaken throughout multiple imputation (MI) models. Multiple imputation analysis will be performed on the ITT population. The frequency and patterns of missing data will be examined and multiple imputation of missing data performed. Multiple imputation models will be conducted separately in the two treatment groups using chained equations applied to all outcomes, including baseline measures, as auxiliary variables. Fifty imputed datasets will be generated including all randomised subjects.

11.3 Methods of analysis

Data analysis for the study will be performed by CEBU at MCRI. Ms Francesca Orsini has been appointed for the trial.
Statistical analysis will follow standard methods for randomised trials and the primary analysis will be by intention to treat (ITT), including all randomised participants.
Categorical variables will be presented as the number and proportion in each category.
Continuous variables will be presented as means and standard deviations (SDs), or medians and interquartile ranges for skewed data, and the range.

PRIMARY ANALYSIS. Comparison between the two groups in the proportions of participants with COVID-19 disease (primary outcome 1), as well as in the proportions of participants with severe COVID-19 (primary outcome 2), will be presented as the absolute risk difference (RD) at 6 months and its 95% confidence interval (CI), obtained using a generalised linear model, with adjustment for the strata (defined by site, age and presence of comorbidity) used in the randomisation. The same analysis will be repeated on the same outcomes at 12 months. As secondary analyses the same models will be run to include also the following covariates: gender, number and type of comorbidities, whether already vaccinated for BCG in the past, and any other factor that may show imbalance between the groups at baseline.

SECONDARY OUTOMES. According to the nature of the secondary outcomes to be analysed (binary, continuous or categorical) the appropriate generalized linear model (GLM) will be

used to estimate the effect of the BCG vaccine on the outcome of interest compared to the control group. All analyses will be adjusted for the stratification factors used in the randomisation (site, age and presence of comorbidity). As secondary analyses the same models will be run to include also the following covariates: gender, number and type of comorbidities, whether already vaccinated for BCG in the past, and any other factor that may show imbalance between the groups at baseline.

Survival analysis techniques will be adopted to analyse to time to event data.

SUBGROUP ANALYSIS
Analyses will be undertaken on outcomes of those who:
- Have a history of recurrent herpes simplex infection
- Had previous BCG vaccine before enrolling into the trial

The full details for each variable will be included in the Statistical Analysis Plan (SAP).

11.4 Interim Analyses

No interim analysis will be undertaken.

12 ETHICS AND DISSEMINATION

12.1 Research Ethics Approval & Local Governance Authorisation

This protocol and the informed consent document and any subsequent amendments will be reviewed and approved by the human research ethics committee (HREC) prior to commencing the research. A letter of protocol approval by HREC will be obtained prior to the commencement of the trial, as well as approval for other trial documents requiring HREC review.

Each participating institution will also obtain institutional governance authorisation for the research and associated HREC-approved documents. A letter of authorisation will be obtained from the RGO prior to the commencement of the research at that institution. Institutional governance authorisation for any subsequent HREC-approved amendments will be obtained prior to implementation at each site.

12.2 Amendments to the protocol

This trial will be conducted in compliance with the current version of the protocol. Any change to the protocol document or Informed Consent Form that affects the scientific intent, trial design, participant safety, or may affect a participants willingness to continue participation in the trial is considered an amendment, and therefore will be written and filed as an amendment to this protocol and/or informed consent form. All such amendments will be submitted to the HREC, for approval prior to being implemented.

12.3 Protocol Deviations and Serious Breaches

All protocol deviations will be recorded in the participant record (source document) and on the CRF and must be reported to the Sponsor-Investigator or delegate, who will assess for seriousness.

Those deviations deemed to affect to a significant degree rights of a trial participant or the reliability and robustness of the data generated in the clinical trial will be reported as serious breaches. Reporting will be done in a timely manner (the sponsor-Investigator to review and submit to the approving HREC within 7 days).

Where non-compliance significantly affects human participant protection or reliability of results, a root cause analysis will be undertaken and a corrective and preventative action plan prepared.

Where protocol deviations or serious breaches identify protocol-related issues, the protocol will be reviewed and, where indicated, amended.

13 CONFIDENTIALITY

Participant confidentiality is strictly held in trust by the participating investigators, research staff, and the sponsoring institution and their agents. This confidentiality is extended to cover testing of biological samples in addition to the clinical information relating to participating participants.

The trial protocol, documentation, data and all other information generated will be held in strict confidence. No information concerning the trial or the data will be released to any unauthorised third party, without prior written approval of the sponsoring institution. Authorised representatives of the sponsoring institution may inspect all documents and records required to be maintained by the Investigator. The clinical trial site will permit access to such records.

All laboratory specimens, evaluation forms, reports and other records that leave the site will be identified only by the Participant Identification Number (SID) to maintain participant confidentiality.

Clinical information will not be released without written permission of the participant, except as necessary for monitoring by HREC or regulatory agencies.

14 PARTICIPANT REIMBURSEMENT

Participants will not be reimbursed for their involvement

15 FINANCIAL DISCLOSURE AND CONFLICTS OF INTEREST

This is an investigator-initiated study, and the funders will have no role in the study design, data collection and analysis, decision to publish, or preparation of the manuscript. All investigators have no financial or competing interest to declare.

16 DISSEMINATION AND TRANSLATION PLAN

The results of the trial will be reported to the participants after analysis is complete. The results of this trial will be submitted to peer reviewed journals, presented at conferences and may form part of student theses.

The sponsor-investigator holds primary responsibility for publication of the results of the trial.

17 ADDITIONAL CONSIDERATIONS

No further considerations.

18 REFERENCES

1. Novakovic B, et al. Chapter 6 - The Heterologous Effects of Bacillus Calmette-Guérin (BCG) Vaccine and Trained Innate Immunity. In: Faustman DL, ed. The Value of BCG and TNF in Autoimmunity (Second Edition). Second edition. ed: Academic Press; 2018: 71-90
2. World Health Organisation. Situation report - 50: World Health Organisation, 2020
3. Anderson RM, et al. How will country-based mitigation measures influence the course of the COVID-19 epidemic? *The Lancet* 2020;
4. World Health Organisation. SARS (Severe Acute Respiratory Syndrome). 2020. https://www.who.int/ith/diseases/sars/en/ (accessed 11/03/2020 2020)
5. World Health Organisation. Middle East respiratory syndrome coronavirus (MERS-CoV). 11 March 2019 2020. https://www.who.int/en/news-room/fact-sheets/detail/middle-east-respiratory-syndrome-coronavirus-(mers-cov) (accessed 11/03/2020 2020)
6. World Health Organisation. Report of the WHO-China Joint Mission on Coronavirus Disease 2019 (COVID-19) World Health Organisation, 2020
7. Elguero E, et al. Non-specific effects of vaccination on child survival? A prospective study in Senegal. *Tropical medicine & international health : TM & IH* 2005; **10**(10):956-60
8. Higgins JP, et al. Association of BCG, DTP, and measles containing vaccines with childhood mortality: systematic review. *BMJ (Clinical research ed)* 2016; **355**i5170
9. Kristensen I, et al. Routine vaccinations and child survival: follow up study in Guinea-Bissau, West Africa. *BMJ (Clinical research ed)* 2000; **321**(7274):1435-8
10. Nankabirwa V, et al. Child survival and BCG vaccination: a community based prospective cohort study in Uganda. *BMC public health* 2015; **15**175
11. Vaugelade J, et al. Non-specific effects of vaccination on child survival: prospective cohort study in Burkina Faso. *BMJ (Clinical research ed)* 2004; **329**(7478):1309
12. Hollm-Delgado MG, et al. Acute lower respiratory infection among Bacille Calmette-Guerin (BCG)-vaccinated children. *Pediatrics* 2014; **133**(1):e73-81
13. de Castro MJ, et al. Nonspecific (Heterologous) Protection of Neonatal BCG Vaccination Against Hospitalization Due to Respiratory Infection and Sepsis. *Clin Infect Dis* 2015; **60**(11):1611-9
14. Arts RJW, et al. BCG Vaccination Protects against Experimental Viral Infection in Humans through the Induction of Cytokines Associated with Trained Immunity. *Cell Host Microbe* 2018; **23**(1):89-100 e5
15. Nemes E, et al. Prevention of M. tuberculosis Infection with H4:IC31 Vaccine or BCG Revaccination. *N Engl J Med* 2018; **379**(2):138-49
16. Wardhana, et al. The efficacy of Bacillus Calmette-Guerin vaccinations for the prevention of acute upper respiratory tract infection in the elderly. *Acta Med Indones* 2011; **43**(3):185-90
17. Moorlag S, et al. Non-specific effects of BCG vaccine on viral infections. *Clin Microbiol Infect* 2019; **25**(12):1473-8

18. Freyne B, et al. BCG-associated heterologous immunity, a historical perspective: intervention studies in animal models of infectious diseases. *Trans R Soc Trop Med Hyg* 2015; **109**(4):287
19. Kleinnijenhuis J, et al. Bacille Calmette-Guerin induces NOD2-dependent nonspecific protection from reinfection via epigenetic reprogramming of monocytes. *Proc Natl Acad Sci U S A* 2012; **109**(43):17537-42
20. Kleinnijenhuis J, et al. BCG-induced trained immunity in NK cells: Role for non-specific protection to infection. *Clin Immunol* 2014; **155**(2):213-9
21. Kleinnijenhuis J, et al. Long-lasting effects of BCG vaccination on both heterologous Th1/Th17 responses and innate trained immunity. *J Innate Immun* 2014; **6**(2):152-8
22. Messina NL, et al. The impact of vaccines on heterologous adaptive immunity. *Clin Microbiol Infect* 2019; **25**(12):1484-93
23. Zimmermann P, et al. The influence of neonatal Bacille Calmette-Guerin (BCG) immunisation on heterologous vaccine responses in infants. *Vaccine* 2019; **37**(28):3735-44
24. Zykov MP, Subbotina TI. Modulation of humoral immune response to influenza vaccines by BCG. *Acta Virol* 1985; **29**(5):403-9
25. Australian Technical Advisory Group on Immunisation (ATAGI). Australian Immunisation Handbook. Canberra: Australian Government Department of Health,; 2018
26. Australian Government Department of Health. The BCG vaccine: information and recommendations for use in Australia
27. Lotte A, et al. BCG complications. Estimates of the risks among vaccinated subjects and statistical analysis of their main characteristics. *Adv Tuberc Res* 1984; **21**107-93
28. Bothamley GH, et al. Tuberculin testing before BCG vaccination. *BMJ (Clinical research ed)* 2003; **327**(7409):243-4
29. Hendry AJ, et al. Adverse events following immunisation with bacille Calmette-Guerin vaccination: baseline data to inform monitoring in Australia following introduction of new unregistered BCG vaccine. *Communicable diseases intelligence quarterly report* 2016; **40**(4):E470-e4
30. Ltd SN. BCG Vaccine SSI data sheet 14 March 2018. https://www.medsafe.govt.nz/profs/datasheet/b/BCGCSLinj.pdf (accessed 24 Dec 2019
31. Pilgrim S. Administration of the Bacillus Calmette Guerin (BCG) Vaccination in neonates2019. https://www.meht.nhs.uk/EasysiteWeb/getresource.axd?AssetID=14766&type=full&servicetype=Attachment (accessed 24 Dec 2019)
32. World Health Organization. Bulletin of the World Health Organization 1995: World Health Organization, 1995
33. Fine P, et al. Issues relating to the use of BCG in immunization programmes: a discussion document. Geneva: Department of Vaccines and Biologicals, World Health Organization, 1999
34. Paul Fine IC, Julie Milstien, C. John Clements. Issues relating to the use of BCG in immunization programmes: a discussion document. Geneva: Department of Vaccines and Biologicals, World Health Organization, 1999
35. Immunisation schedules in the WHO European Region: World Health Organization, 1995
36. Coulter C. Tuberculosis testing. *Aust Fam Physician* 2012; **41**(7):489-92
37. Randomised controlled trial of single BCG, repeated BCG, or combined BCG and killed Mycobacterium leprae vaccine for prevention of leprosy and tuberculosis in Malawi. Karonga Prevention Trial Group. *Lancet* 1996; **348**(9019):17-24
38. Roth AE, et al. Effect of revaccination with BCG in early childhood on mortality: randomised trial in Guinea-Bissau. *BMJ (Clinical research ed)* 2010; **340**c671
39. Rakshit S, et al. BCG revaccination boosts adaptive polyfunctional Th1/Th17 and innate effectors in IGRA+ and IGRA- Indian adults. *JCI Insight* 2019; **4**(24):

40. Hatherill M, et al. Safety and reactogenicity of BCG revaccination with isoniazid pretreatment in TST positive adults. *Vaccine* 2014; **32**(31):3982-8
41. Rodrigues LC, et al. Effect of BCG revaccination on incidence of tuberculosis in school-aged children in Brazil: the BCG-REVAC cluster-randomised trial. *Lancet* 2005; **366**(9493):1290-5
42. Cunha AJ, et al. Adverse effects of BCG revaccination: a report on 13 cases from Rio de Janeiro, Brazil. *Int J Tuberc Lung Dis* 2002; **6**(12):1110-3
43. Faustman DL. Type 1 Diabetes Reversal Trials at Massachusetts General Hospital. In: Hospital MG, editor. Massachusetts General Hospital: Massachusetts General Hospital; 2018

19 APPENDICES

19.1 Appendix 1: Division of sponsor responsibilities between sponsor and sponsor-investigator

For non-commercial trials, the overall responsibility for initiating and managing the trial, lies with the sponsor. However, as the person responsible for leading the team of researchers undertaking the design, conduct and reporting of the trial, the Sponsor-Investigator should have oversight of all activities, even when they are delegated to third parties such as clinical trial units or coordinating centres.

Before taking on the role of Sponsor-Investigator, it is important to understand:
- *What you need to have in place before you start a trial*
- *What aspects of the trial you need to review as the trial progresses*

This document sets out the typical allocation of clinical trial functions between the Sponsor-Investigator and the sponsor for investigator-led or collaborative group clinical trials; note that this is an example and may be amended on a case by case basis).

**Sponsor-Investigator:**	Prof Nigel Curtis	
**Responsibility**	Sponsor	Sponsor-Investigator
Ensure a peer review/independent expert review has demonstrated that the trial proposal is worthwhile and is of high scientific quality.	X	
Ensure the Sponsor-Investigator has adequate procedures in place for all key trial management activities	X	
Assign an overall risk category based on type of intervention	X	
Ensure that the Sponsor-Investigator has the necessary expertise and experience to conduct the trial	X	
Ensure that the Sponsor-Investigator has the resources needed to complete the trial successfully or that plans are in place to raise additional funds.	X	
Confirm provision of insurance and indemnity for the trial and trial related staff as well as measures for participant compensation for trial related injury	X	
Ensure all the roles and responsibilities for the clinical trial are delegated, agreed and documented appropriately	X	
Oversee/sign-off all contract negotiations with external providers (e.g. external lab facilities; pharmaceutical companies for supply of investigational product,)	X	
Ensure the protocol (or other document) details appropriate monitoring and management plans commensurate to the risk and complexity of the trial	X	

Maintain oversight to include audit, where applicable	X	
Ensure that the trial is based on a thorough review of scientific literature including whether any relevant systematic review exists.		X
Secure funding and/or confirm sufficient resources are available to conduct the trial (e.g. trial subjects, time, staff, facilities, finances) or put in place plans to raise additional funds.		X
Ensure that trials are registered on clinical.trials.gov, ANZCTR or other appropriate registry before first patient is enrolled and that appropriate plans for the dissemination of trial findings are in place		X
Unless delegated to a third party, undertake/oversee the design, conduct and reporting of the trial with support from all relevant specialist staff (e.g. statistician, research methodologist) including the development of a protocol that is compliant with international standards including the SPIRIT Statement. Where appropriate, prepare the regulatory dossier for trials aiming to commercialise a new investigational medicinal product/investigational medical device.		X
Ensure a trial risk assessment has been carried out and proportionate trial management and monitoring plans are in place		X
For investigational medicinal product trials, ensure (through the appointed pharmacist, medical engineering) that all requirements for IMP/IMD supplies are met (e.g. manufacture/ packaging/labelling).		X
Develop/endorse an appropriate strategy for independent trial oversight (e.g. Trial Management Group, Trial Steering Committee, Data Safety Monitoring Board)<br>If a Data Safety Monitoring Board is not warranted, ensure alternative mechanisms for ongoing safety monitoring are in place		X
Document trial specific delegation of duty on a Staff Signature and Delegation Log		X
Confirm each member of the trial team are aware of their trial-related duties		X
Ensure the development of all relevant trial documentation (e.g. protocol, Participant Information and Consent Form and Case Report Form)		X
Develop/obtain the investigator's brochure or where appropriate, the Product Information to be used for the trial and ensure that the reference safety information for identifying expectedness of adverse events is clearly identified		X
Oversee the set-up of a clinical trial database		X
Ensure all trial approvals and notification are in place before the trial commences (e.g. HREC, SSA, TGA)		X
Ensure relevant agreements/signatories from service departments supporting the trial (e.g. pharmacy, laboratories, radiology) are obtained		X
Ensure arrangements are in place for the effective financial management of the trial		x
Prepare and submit amendments to the trial		X
Implement procedures to ensure the collection of high quality and accurate data		X
Oversee the set-up and maintenance of a Trial Master File		X
Ensure safety reporting and monitoring for the trial complies with the requirements of the NHMRC Guidance for Safety Monitoring and confirm and execute any sponsor reporting responsibilities that are delegated		X
Submit annual report(s) to the HREC and Research Office in accordance with Australian Guidance and local requirements		X
Report suspected serious breaches of GCP/protocol to the HREC and Research Office in accordance with the NHMRC Guidance		X
Notify HREC, Research Office, TGA and other relevant bodies of the completion of the trial		X
Produce all necessary reports to funders and others		X
Disseminate trial findings through publication/dissemination of trial results where applicable, following the CONSORT Statement		X

Fulfil commitments to trial participants, such as providing information about the outcome(s) of the trial, re-obtaining consent if required due to change in risk-benefit ratio (of investigational medicinal product) or change in protocol procedures		X
Ensure all trial data (including the Trial Master File) and materials, are archived appropriately and retrievable for audit purposes		X
Maintain trial registration record in accordance with the registry's requirements		X

19.2 APPENDIX 2: Significant Safety Issues (SSI) - some examples

Examples below have been extracted from the NHMRC's "Safety monitoring and reporting in clinical trials involving therapeutic goods" (November 2016)

- a serious adverse event that could be associated with the trial procedures and that requires modification of the conduct of the trial
- a hazard to the patient population, such as lack of efficacy of an IMP used for the treatment of a life-threatening disease
- a major safety finding from a newly completed animal study (such as carcinogenicity)
- a temporary halt/termination of a trial for safety reasons
- recommendations of the Data Safety Monitoring Board, where relevant for the safety of participants, such as an increase in frequency or severity of an expected adverse reaction
- single case events (e.g. toxic epidermal necrolysis, agranulocytosis, hepatic failure) that lead to an urgent safety measure

Examples below have been extracted from the TGA's "Pharmacovigilance responsibilities of medicine sponsors: Australian recommendations and requirements" Version 2.0, September 2017

- safety-related actions by comparable international regulatory agencies such as the:
  - withdrawal or suspension of the medicine's availability
  - addition or modification, for safety reasons, of a contraindication, warning or precaution statement to the product information or label
  - modification or removal, for safety reasons, of an indication.
- changes in the nature, severity or frequency of known serious adverse reactions which are medically significant
- detection of new risk factors for the development of a known adverse reaction or a new serious adverse reaction that may impact on the safety or benefit-risk balance of the medicine
- series of reports of similar or linked adverse reactions reported at the same time (that is, a cluster) assessed to suggest a quality defect issue that may have implications for public health
- an unusual and significant lack of efficacy occurring in or outside Australia that may have implications for public health
- major safety findings from a newly completed non-clinical study, post-registration study or clinical trial that may impact the benefit-risk balance of the medicine on the ARTG
- a signal of a possible teratogenic effect or of significant hazard to public health
- safety issues related to any raw materials used in the medicine that may impact the safety of the medicine and/or have implications for public health
- safety issues due to misinformation in the product information or label that may impact the safety of the medicine
- safety issues related to use outside the approved indication or intended use that may impact the safety or benefit-risk balance of the medicine

19.3 APPENDIX 3: Specimens for biobanking - completed biobank registration form

**Document version & date**	Version 1 13th March 2020
**Name of the bank**	BCG vaccine to prevent severe COVID-19 disease in healthcare workers: an RCT (BCG-19)
**Custodian of the bank**	Name: Prof Nigel Curtis
**Purpose of the bank**	To store data and samples collected in the 'BCG vaccine to prevent severe COVID-19 disease in healthcare workers: an RCT (BCG-19)' so they can be used in future research related to infectious diseases and immunity.
**Sample/data type(s) and where these will be accessed from and over what time period**	Data will be collected from<br>Questionnaires, Medicare records and test results obtained as part of the research project 'BCG vaccine to prevent severe COVID-19 disease in healthcare workers: an RCT (BCG-19)', by members of the research team.<br>Blood and/or swab samples will be obtained via this research project also, and will be stored for an indefinite period of time.<br>The samples/data may be sent overseas for future research related to infectious diseases, immunology, or vaccines.<br>Data stored includes:<br>- Demographics (e.g. age, gender, date)<br>- Environment (e.g. household members, exposure to SARS-CoV-2 positive people, role in the hospital, TB exposure, previous vaccinations)<br>- Study outcome related data (e.g. SARS-CoV-2 test results, BCG and flu vaccine reactions, illnesses during study period, data generated from the laboratory analysis of samples collected)<br>Sample types stored:<br>- Swabs<br>- Plasma<br>- Serum<br>- Peripheral blood samples<br>- Granular sites and whole blood<br>- Nucleic acid<br>After data ceases to be collected directly from participants, data may be obtained/generated via access to their medical records, government data sets or as samples are analysed and the data is added back into the data/biobank.
**Sample/data identifiability**	Clinical data in 'BCG vaccine to prevent severe COVID-19 disease in healthcare workers: an RCT (BCG-19)'will be collected

	and stored in a REDCap database; a secure password-encrypted online database, or similar electronic database hosted by MCRI.<br>Data will be stored in re-identifiable format with the key held by the custodian or delegate. The REDCap database or comparable database will be hosted on the secure Murdoch Children's Research Institute (MCRI) server and backed up regularly by MCRI Information Technology.<br>Only members of the research team involved in data collection or data management will have access to the project's REDCap database or similar electronic database.<br>Samples will be stored (frozen) in re-identifiable format by using study ID number.<br>All data associated with sample storage location and tracking will be stored in a separate REDCap database or similar electronic database 'BCG-19 samples'. Access to this database is limited to members of the research team working in data/sample management or sample processing.<br>Laboratory generated data, any data collected outside of REDCap and data exported from REDCap or similar electronic database, will be stored in re-identifiable format by study ID. The data will be stored on the MCRI server in restricted folders on the Infectious Diseases group drive, as per MCRI policy.<br>Samples/data stored in re-identifiable format can be linked by the custodian or delegate to participants' identifiable information if it is ethically appropriate and required.
Criteria for Bank participants	Consenting to the project includes allowing the participants' data and samples to be used as defined in the protocol.<br>In addition there is an optional consent in the PICF for the storage of participants' biospecimens and participants' re-identifiable data for use in future research related to infectious diseases and immunity.<br>Inclusion criteria for Bank participants<br>- Recruited participant in the research project 'BCG vaccine to prevent severe COVID-19 disease in healthcare workers: an RCT (BCG-19)'<br>- Provided informed consent for their data and samples to be stored for future ethically approved research (extended consent) related to infectious diseases and immunity.
Access process for obtaining the samples/data	Researchers must discuss their research plan with a member of the research team of the project 'BCG vaccine to prevent severe COVID-19 disease in healthcare workers: an RCT (BCG-19)'. The following will be taking into consideration:

	- Scientifically justifiable hypothesis and aims<br>- Study design is appropriate to achieve study aims<br>- Inclusion/exclusion criteria for participants appropriate to answer question<br>- If the research proposal is deemed to have merit, the researcher will complete a REDCap (or similar electronic database) access form detailing the proposed design, participants, data +/- samples that they would like access to.<br>This will be reviewed by the custodian (or delegate) of the data who will need to take into account the following, before approval is granted:<br>- Does the research plan involve research in the area of immunology or infectious diseases? If not, it is outside the scope of the data/biobank. To use the data one of the following will be required:<br>o a new project approved by the RCH HREC and participants contacted for their consent<br>o a new project approved by the RCH HREC and a waiver of consent granted<br>- Is the planned analysis feasible with the data/samples available in the data/biobank?<br>- Are there competing interests for the sample/data type in question?<br>- Is another researcher already analysing the data in a similar way and would collaboration on the existing project be more appropriate?<br>The access form for access to the data/biobank will be kept on the REDCap database or similar electronic database.
Sample and data input	Members of the research team working in data/sample management will input the data and samples to the data/biobank.
Location of the Bank	Samples will be stored in the MCRI freezer farm or in the Infectious Disease Group's freezers, and may be distributed to other collaborating laboratories where they may also be stored.<br>Data will be stored in a REDCap online database or similar electronic database, hosted on the secure Murdoch Children's Research Institute (MCRI) server, as well as in restricted electronic folders on the MCRI Infection and Immunity group drive.

**Confidentiality/security of samples/data**	Members of the research team of the project 'BCG vaccine to prevent severe COVID-19 disease in healthcare workers: an RCT (BCG-19)' involved in data/sample collection or management will have open access to the bank data/samples.<br>No identifying data will be provided to researchers using data/samples from the biobank. To re-identify data/samples, the custodian (or delegate) will have access to the key, but will not pass this information onto researchers unless approved by ethics, or as required by law.<br>Data stored on REDCap database or similar electronic database will be password protected, and hosted on the secure MCRI server. This is backed up regularly by MCRI Information Technology.<br>The Bank will be secure against unauthorised access and passwords will be changed at regular intervals (as per MCRI policy).<br>The custodian (or delegate) will ensure removal of access to data once a project is finished or a researcher leaves the project.
**Destruction of samples/data**	Destruction of samples/data will occur upon participant request. This will be managed by the custodian (or delegate).
**Modifications to Bank Protocol**	If a change of purpose/data type/type of samples is to be considered, the custodian (or delegate) is required to submit to the HREC for approval and either contact the participants to obtain consent, or a waiver must have been granted.

PROTOCOL

RCH HREC/protocol no: 62586

NCT04327206

BCG vaccination to Reduce the impact of COVID-19 in healthcare workers (BRACE) Trial

Version 12.0, 17May2022

CONFIDENTIAL
This protocol is confidential and is the property of Murdoch Children's Research Institute. No part of it may be transmitted, reproduced, published, or used without prior written authorisation from the institution.

Statement of Compliance

This clinical trial will be conducted in compliance with all stipulation of this protocol, the conditions of the ethics committees' approvals, and the Integrated Addendum to ICH E6 (R1): Guideline for Good Clinical Practice E6 (R2), dated 9 November 2016.

In Australia, this trial will also be conducted in compliance with with the NHMRC National Statement on Ethical Conduct in Human Research (2007 and all updates), the Integrated Addendum to ICH E6 (R1): Guideline for Good Clinical Practice E6 (R2), dated 9 November 2016 annotated with TGA comments and the NHMRC guidance Safety monitoring and reporting in clinical trials involving therapeutic goods (EH59, 2016).

This clinical trial is not sponsored by any pharmaceutical company or other commercial entity.

CONTENTS

PROTOCOL SYNOPSIS 6
Primary objectives 6
Secondary objectives 6
GLOSSARY OF ABBREVIATIONS 10
INVESTIGATOR AGREEMENT 11
1. ADMINISTRATIVE INFORMATION 12
1.1. Trial registration 12
1.2. Overall Sponsor 12
1.3. Expected duration of study 12
1.4. Stakeholder involvement 12
2. INTRODUCTION AND BACKGROUND 12
2.1. Trial rationale and aim 12
2.2. Background 13
2.3. Risk/Benefit assessment 15
2.3.1. Known potential risks 15
2.3.2. Known potential benefits 19
2.3.3. Assessment of potential risks and benefits 19
3 TRIAL OBJECTIVES AND OUTCOMES 20
3.1 Objectives 20
3.1.1 Primary objective 20
3.1.2 Secondary objectives 20
3.1.3 Planned exploratory analyses 21
3.2 Outcomes 22
4 TRIAL DESIGN 25
4.1 Overall design 25
4.2 Justification for dose 26
4.3 Trial population 26
4.3.1 Eligibility criteria 26
4.3.2 Inclusion criteria 27
4.3.3 Exclusion criteria 27
4.4 Lifestyle considerations 29
4.5 Screen failures 29
4.6 Recruitment and Consent 29

4.7 Pre-randomisation blood sample 30
4.8 Re-consent 30
5 INTERVENTION 31
5.1 Treatment arms 31
5.2 Trial Intervention(s) 31
5.2.1 Description of trial investigational products 31
5.2.1.1 BCG vaccine SSI 31
5.2.1.2 Placebo to match BCG vaccine SSI 31
5.2.2 Dosage 32
5.2.3 Dose modification 32
5.2.4 Storage and dispensing of BCG vaccine SSI 32
5.2.5 Preparation 32
5.2.6 Administration of trial drug 33
5.2.7 Product accountability 35
5.2.8 Excluded medications and treatments 35
5.2.9 Discontinuation from trial intervention 35
6 RANDOMISATION AND BLINDING 35
6.1 Concealment mechanism 36
7 TRIAL VISITS AND PROCEDURES 37
7.1 Trial timeline 37
7.2 Schedule of assessments 38
7.3 Description of procedures 38
7.4 Notes on specific trial visits 42
7.4.1 Unscheduled visit 42
7.5 Procedure discontinuation, participant withdrawals and losses to follow up 42
7.5.1 Discontinuation of blood collection - participant remains in trial for follow up 42
7.5.2 Withdrawal of consent - participant withdraws from all trial participation 42
7.5.3 Losses to follow-up 42
7.5.4 Replacements 43
7.5.5 Trial Completion 43
7.5.6 Continuation of therapy 43
8 SAFETY MONITORING AND REPORTING 44
8.1 Definitions 44
8.2 Capturing and eliciting adverse event information 45

8.2.1 SAE capture 45
8.2.2 Non-Serious AE Capture..46
8.3 Documentation of AEs..46
8.4 Assessing the relatedness (causality) of a participant's AE 46
8.5 Assessing the severity of a participant's AE 47
8.6 Reporting of safety events 48
9 DATA AND INFORMATION MANAGEMENT 49
9.1 Overview 49
9.2 Data management...49
9.2.1 Data generation (source data) 49
9.2.2 Data capture methods and data use, storage, access and disclosure during the trial 50
9.2.3 Data confidentiality 51
9.2.4 Quality assurance 51
9.2.5 Archiving - Data and document retention...51
9.2.6 Data sharing 52
9.2.7 Long-term custodianship (after archive period finished) 52
9.2.8 Data retrieval 53
9.2.9 Sample management: Additional data management considerations 53
9.2.10 Sample management: Specimen collection & storage. 53
9.2.11 Sample management: Specimen & Biobanking 53
10 TRIAL OVERSIGHT 54
10.1 Governance structure 54
10.1.1 Trial Steering Committee (TSC)...54
10.1.2 Independent Data and Safety Monitoring Board (DSMB) 54
10.1.3 Independent Safety Monitor 55
10.1.4 Quality control and quality assurance. 55
11 STATISTICAL METHODS 55
11.1 Sample Size Estimation 55
11.2 Population to be analysed...57
11.2.1 Handling of missing data 57
11.3 Methods of analysis 57
11.4 Interim Analyses..58
12 ETHICS AND DISSEMINATION 59
12.1 Research Ethics Approval & Local Governance Authorisation 59

12.2 Amendments to the protocol 59
12.3 Protocol Deviations and Serious Breaches 59
13 CONFIDENTIALITY 60
14 PARTICIPANT REIMBURSEMENT 60
15 FINANCIAL DISCLOSURE AND CONFLICTS OF INTEREST 60
16 DISSEMINATION AND TRANSLATION PLAN 60
17 REFERENCES 61
17.1 Appendix 1: Specimens for biobanking - completed biobank registration form 64
17.2 Appendix 2. Collection of stool samples from a subset of BRACE participants 68
17.3 Appendix 3 UK Specific Requirements 72
17.4 Appendix 4 Brazil Specific Requirements 73
17.5 Appendix 5 The Netherlands Specific Requirements 75
17.6 Appendix 6 Spain Specific Requirements 76
17.7 Appendix 7 Optional Biological sample collection during episodes of illness 77
17.8 Appendix 8 Optional Sub-study: collection of blood samples to measure immune responses to COVID-19 specific vaccines. 80
17.9 Appendix 9 Optional Sub-study: analysis of swab samples to determine the impact of SARS-CoV-2 variants 86

PROTOCOL SYNOPSIS

TITLE	*BCG vaccination to Reduce the impact of COVID-19 in healthcare workers (BRACE) Trial*
TRIAL DESCRIPTION	Phase III, two group, multicentre, randomised placebo-controlled trial in up to 7244 healthcare workers to determine if BCG vaccine reduces incidence and the severity of COVID-19 during the 2020 SARS-CoV-2 pandemic. The trial includes a pre-planned meta-analysis with data from the 2834 participants recruited in first stage of this study which followed the same protocol but where participants were randomised between BCG and no BCG at the time of receiving an influenza vaccination, with a total sample size of 10078.<br><br>Randomisation and immunisation will occur at each participating site. Participants will be randomised to receive BCG vaccine or 0.9% NaCl placebo. Participants will be followed-up for 12 months with notification from a smartphone application (up to daily when ill) or via phone calls, electronic messages, home visits and surveys to identify and detail suspected COVID-19 infection. Additional information on severe disease will be obtained from hospital medical records and/or government databases. Blood samples will be collected prior to randomisation, at 3 and 6 months, and in a sub-set of participants at 9 and 12 months to determine SARS-CoV-2 exposure. Where required swab/blood samples will be taken at illness episodes to assess SARS-CoV-2 infection.
OBJECTIVES	Primary objectives<br>1. To determine if BCG vaccination (Intervention) compared with placebo (Comparator) reduces the incidence of symptomatic COVID-19 (Outcome) measured over the 6 months following randomisation (Time) in healthcare workers (Participants).<br>2. To determine if BCG vaccination (Intervention) compared with placebo (Comparator) reduces the incidence of severe COVID-19 (COVID-19-related death, hospitalisation, or non-hospitalised severe disease, defined as 'non-ambulant' for ≥ 3 consecutive days OR Unable to work for ≥ 3 consecutive days) (Outcome) measured over the 6 months following randomisation (Time) in healthcare workers (Participants).<br><br>SECONDARY OBJECTIVES<br>3. To determine if BCG vaccination (Intervention) compared with placebo (Comparator) reduces the incidence of symptomatic COVID-19 (Outcome) measured over the 12 months following randomisation (Time) in healthcare workers (Participants).<br>4. To determine if BCG vaccination (Intervention) compared with placebo (Comparator) reduces the incidence of severe COVID-19

	(non-hospitalised severe disease, hospitalisation or death) (Outcome) measured over the 12 months following randomisation (Time) in healthcare workers (Participants).<br>5. To determine if BCG vaccination (Intervention) compared with placebo (Comparator) prolongs the time to first COVID-19 episode (Outcome) measured over 6 and 12 months following randomisation (Time) in healthcare workers (Participants).<br>6. To determine if BCG vaccination (Intervention) compared with placebo (Comparator) reduces the severity of COVID-19 (Outcome) measured over 6 and 12 months following randomisation (Time) in healthcare workers (Participants).<br>7. To determine if BCG vaccination (Intervention) compared with placebo (Comparator) reduces the rate and severity of illness (fever or at least one sign or symptom of respiratory disease) measured over 12 months following randomisation (Time) in healthcare workers (Participants).<br>8. To determine if BCG vaccination (Intervention) compared with placebo (Comparator) reduces absenteeism (days off work) measured over 6 and 12 months following randomisation (Time) in healthcare workers (Participants).<br>9. To evaluate the safety of BCG vaccination in healthcare workers.<br>Planned exploratory analyses<br>10. To determine in a subgroup of adults with recurrent cold sores whether BCG vaccination compared with placebo reduces herpes simplex recurrences (such as cold sores).<br>11. To determine the BCG vaccination induces changes in the immune system that are associated with protection of healthcare workers from non-tuberculous infectious diseases including COVID-19.<br>12. To determine and compare changes in the immune system induced by vaccination of healthcare workers.<br>13. To identify factors (e.g. age, sex, chronic conditions such as diabetes and cardiovascular disease, smoking, asthma, prior BCG vaccination, genetics, other vaccinations including COVID-19-specific vaccines, latent TB, immunological/molecular factors) that influence immune responses, infection and COVID-19 risk.<br>14. *(Brazil specific) To identify biomarkers for diagnosing TB infection*

OUTCOMES AND OUTCOME MEASURES	Primary outcomes:<br>1. Symptomatic COVID-19 defined as:<br>• a positive SARS-CoV-2 test (PCR, RAT or serology) over the 6 months following randomisation PLUS<br>• fever (using self-reported questionnaire)OR<br>• at least one sign or symptom of respiratory disease, including cough, sore throat, shortness of breath, respiratory distress/failure (using self-reported questionnaire)<br>2. Severe COVID-19 defined as:<br>• a positive SARS-CoV-2 test (PCR, RAT or serology) over the 6 months following randomisation PLUS<br>• Death (as a consequence of COVID-19)<br>• Hospitalised (including mechanical ventilation and death), OR<br>• Non-hospitalised severe disease, defined as Non-ambulant[1] for ≥ 3 consecutive days OR Unable to work[2] for ≥ 3 consecutive days<br>*[1] "pretty much confined to bed (meaning finding it very difficult to do any normal daily activities"*<br>*[2] "I do not feel physically well enough to go to work"*<br>Secondary outcomes: All assessed at 6 and 12 months following randomisation unless otherwise indicated.<br>- The following outcomes are for both COVID-19 and fever or respiratory illness: presence of disease, days unable to work, days confined to bed, of days with symptoms, pneumonia, need for oxygen therapy, admission to critical care, need for mechanical ventilation<br>- Symptomatic or severe COVID-19, fever or respiratory illness<br>- Time to first symptom of COVID-19, fever or respiratory illness<br>- Deaths<br>- Number of days of unplanned absenteeism<br>- Type and severity of local and systemic adverse event over the 3 months following randomisation<br>- Planned exploratory analyses: episodes of and time to first recurrence of herpes simplex recurrence, immunological studies
TRIAL POPULATION	7244 adult healthcare workers from Brazil, Europe and Australia (Victoria, Western Australia, South Australia and New South Wales) will be involved in the study, plus 2834 recruited in the earlier stage of this study. Key exclusion criteria are having BCG vaccine contraindication, previously had a SARS-CoV-2 positive test result and prior involvement in this trial at an alternate study site. Participants will be randomised at 1:1 ratio giving approximately 5039 per group.
DESCRIPTION OF SITES	Multiple sites will enrol healthcare workers in Brazil, Europe and Australia.

ENROLLING PARTICIPANTS	Australian sites involved in this study include the Royal Children's Hospital (RCH) VIC, Monash Health VIC, Epworth Healthcare VIC, Perth Children's Hospital WA, Fiona Stanley Hospital WA, Sir Charles Gairdner Hospital WA, the Royal Adelaide Hospital SA, Women's and Children's Hospital Adelaide SA, The Children's Hospital at Westmead NSW, Westmead Hospital NSW, Prince of Wales Hospital NSW, St Vincent's Hospital NSW and Sydney Children's Hospital, Randwick NSW. Recruitment and follow-up may occur on site or at centrally identified locations overseen by Regional and/or Site Investigators.<br><br>In Brazil, the study will be carried out in three cities, Campo Grande-MS, Rio de Janeiro-RJ and Manaus-AM. In Campo Grande, the Faculty of Medicine of UFMS, State Regional Hospital of Mato Grosso do Sul, Municipal Health Units, CASSEMS Hospital, Santa Casa Hospital and Eyes Hospital of the Pantanal will participate. In Rio de Janeiro the Centro de Referência Professor Hélio Fraga (CRPHF) da Escola Nacional de Saúde Pública Sergio Arouca (ENSP), FIOCRUZ and Municipal Health Office of Rio de Janeiro. In Manaus, the Tropical Medicine Foundation and the State Health Department of Amazonas will participate.<br><br>In the Netherlands the study will be carried out in Noordest Ziekenhuis Alkmaar, Rijnstate Hospital Arnhem, Amphia Hospital Breda, St Antonius Hospital Nieuwegein, Radboud UMC Nijmegen and Universitair Medisch Centrum Utrecht.<br><br>In Spain the study will be carried out in University Hospital German Trias I Pujol Barcelona, Mutua Terrassa University Hospital Barcelona, University Hospital Cruces Bizkaia, Marqués de Valdecilla University Hospital Santander and University Hospital Virgen Macarena Sevilla.<br><br>In the United Kingdom (UK) the study will be carried out in Teign Estuary Medical Group Devon, Ide Lane Surgery Exeter, Travel Clinic Exeter, St Leonard's Practice Exeter and Royal Devon and Exeter NHS Foundation Trust Exeter.
DESCRIPTION OF INTERVENTIONS	BCG vaccination group: BCG Denmark, 0.1 mL injected intradermal over the distal insertion of the deltoid muscle onto the humerus.<br>Control group: 0.1 ml of 0.9% NaCl injected intradermal over the distal insertion of the deltoid muscle onto the humerus.
TRIAL DURATION	2.5 years
PARTICIPANT DURATION	13.5 months from randomisation to final follow-ups

GLOSSARY OF ABBREVIATIONS

ABBREVIATION	TERM
AE	Adverse Event
AR	Adverse Reaction
BCG	Bacillus Calmette–Guérin Vaccine
BRF	Biobank Registration Form (MCRI)
COVID-19	Coronavirus Disease 19
CPI	Chief Principal Investigator
CRF / eCRF	Case Report Form / Electronic Case Report Form
DSMB	Data Safety Monitoring Board
ED	Emergency Department
GCP	Good Clinical Practice
HCW	Healthcare Worker/s
HREC	Human Research Ethics Committee
ICH	International Conference on Harmonisation
ITT	Intention To Treat
MCRI	Murdoch Children's Research Institute
MERS	Middle East respiratory syndrome
NHMRC	National Health and Medical Research Council
NSE	Non-Specific Effects
NSW	New South Wales
PI	Principal Investigator
PPE	Personal Protective Equipment
QC	Quality Control
RAT	Rapid Antigen Test
RCH	Royal Children's Hospital (Melbourne)
RGO	Research Governance Office
RPI	Region Principal Investigator
SAE	Serious Adverse Event
SAP	Statistical Analysis Plan
SAR	Serious Adverse Reaction
SARS-CoV-2	Severe Acute Respiratory Syndrome Coronavirus 2
SOP	Standard Operating Procedure
SSI	Significant Safety Issue
SPI	Site Principal Investigator
SUSAR	Suspected Unexpected Serious Adverse Reaction
TB	Tuberculosis
TGA	Therapeutic Goods Administration
UAR	Unexpected Adverse Reaction
USM	Urgent Safety Measure

We use the following terminology with regards to the term 'investigators':

- ***Chief Principal-Investigator** – is used to describe the **overall trial level** Investigator for this multi-site trial: Prof Nigel Curtis of MCRI in Australia (Overall Sponsor)*
- ***Region Principal Investigator** – is used to describe **the region-level** Investigator (i.e. the Region Principal Investigator) responsible for an area including multiple sites in this multi-site trial.*
- ***Site Principal Investigator** – is used to describe **the site-level** Investigator at a participating site in a multi-site trial.*

For some trial sites, one investigator fulfils the role of both Region Principal Investigator and Site Principal Investigator.

INVESTIGATOR AGREEMENT

I have read the protocol entitled "*BCG vaccination to Reduce the impact of COVID-19 in healthcare workers BRACE) Trial*".

By signing this protocol, I agree to conduct the clinical trial, after approval by a Human Research Ethics Committee or Institutional Review Board (as appropriate), in accordance with the protocol, the principles of the Declaration of Helsinki and the good clinical practice guidelines [Integrated Addendum to ICH E6 (R1): Guideline for Good Clinical Practice E6 (R2), dated 9 November 2016].

Changes to the protocol will only be implemented after written approval is received from the applicable Human Research Ethics Committee or Institutional Review Board (as appropriate), with the exception of medical emergencies.

I will ensure that study staff fully understand and follow the protocol and evidence of their training is documented.

Name	Role	Signature and date
Prof Nigel Curtis	Chief Principal Investigator	
Prof Marc Bonten	Region Principal Investigator for the Netherlands and Spain	
Prof Peter Richmond	Region Principal Investigator for Western Australia	
Prof David Lynn	Region Principal Investigator for South Australia	
A/Prof Nicholas Wood	Region Principal Investigator for New South Wales, Australia	
Prof John Campbell	Region Principal Investigator for United Kingdom	
Prof Julio Croda	Region Principal Investigator for Mato Grosso do Sul, Brazil	
Prof Margareth Dalcolmo	Region Principal Investigator for Rio de Janeiro, Brazil	
Prof Marcus Vinicius Guimaraes de Lacerda	Region Principal Investigator for Manaus, Amazonas, Brazil	

1. ADMINISTRATIVE INFORMATION

1.1. Trial registration

This trial is registered on ClinicalTrials.gov, NCT04327206.

1.2. Overall Sponsor

Trial Sponsor	MCRI
Chief Principal Investigator Contact name	Nigel Curtis
Address	Royal Children's Hospital, 50 Flemington Road

On behalf of the Sponsor, MCRI, the Chief Principal Investigator leading the trial will undertake and/or oversee those Sponsor responsibilities delegated by the Sponsor.

1.3. Expected duration of study

The recruitment and IP administration period is expected to take place from March 2020 to March 2021. The individual's follow-up will be 13.5 months from randomisation.

1.4. Stakeholder involvement

Stakeholder
Murdoch Children's Research Institute (MCRI)
Melbourne Children's Trials Centre (MCTC)
Royal Children's Hospital (RCH)
Royal Children's Hospital Immunisation Service
Hospital directors and staff where participants (staff) will be recruited
Hospitals whose staff will be included as sites
Department of Health (for each state)
Australian Health Research Alliance (AHRA)

2. INTRODUCTION AND BACKGROUND

2.1. Trial rationale and aim

In recent months severe acute respiratory syndrome-coronavirus 2 (SARS-CoV-2) has emerged as a novel human pathogen. With no pre-existing immunity against this virus, susceptibly among humans is presumed to be universal. Healthcare workers are at the frontline of novel infectious disease outbreaks such as this. Due to their contact with patients and production of aerosols during some medical procedures they have greater exposure and potentially risk of contracting newly emerged human pathogens. Current strategies to protect healthcare workers rely on the use (and sustained supply) of personal protective

equipment. Healthcare worker absenteeism due to infection with the outbreak pathogen or illness cause by another disease with similar symptoms, compounds the pressure already placed on the healthcare system.

Prophylactic interventions to protect against emerging pathogens are needed, particularly for healthcare workers. The tuberculosis (TB) vaccine, Bacillus Calmette-Guérin (BCG) has beneficial off-target effects and has been shown to protect against non-TB infections[1]. This is proposed to result from BCG mediated boosting of early immune responses. As such, BCG vaccination represents a potential prophylactic intervention to provide protection against emerging pathogens such as SARS-CoV-2.

The aim of this trial is to determine whether in healthcare workers, BCG can reduce the incidence and severity of illness caused by the novel coronavirus, SARS-CoV-2.

2.2. Background

Since the emergence of coronavirus disease 19 (COVID-19) in China in December 2019, there have been over 18,000,000 cases disease and greater than 690,000 deaths caused by the disease globally[2] (as of August 2020). The causative agent of COVID-19 a novel coronavirus, severe acute respiratory syndrome-coronavirus 2 (SARS-CoV-2), has already spread to 108 countries (including over 200 cases in Australia) and it is predicted that up to 60% of the global population could become infected[3]. Following from SARS in 2002[4] and Middle East respiratory syndrome (MERS) in 2012[5], SARS-CoV-2 is the third coronavirus to make the jump from animals to humans and emerge as a serious human pathogen in less than 20 years.

In approximately 80% of cases COVID-19 results in mild to moderate disease with symptoms similar to common respiratory diseases such as influenza-like illnesses, with fever in the majority (87.9%) of cases, followed by dry cough (67.7%), fatigue (38.1%), sputum production (33.4%)[6]. In 14% of cases, SARS-CoV-2 causes severe disease requiring oxygen supplementation and/or mechanical ventilation, with a further 6% being critical cases that have respiratory failure, septic shock and/or organ failure.

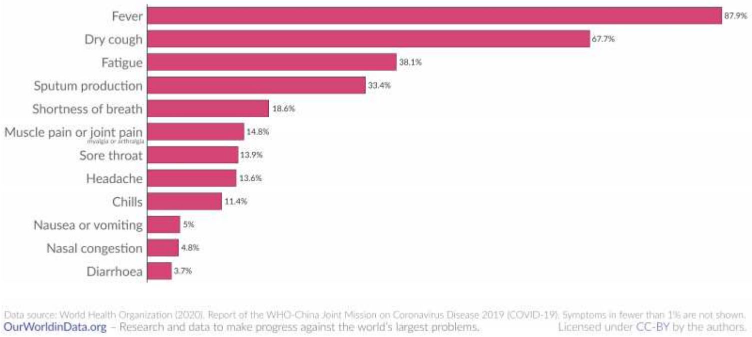

There are worldwide efforts to reduce the peak of SARS-CoV-2 infection, in order to have enough hospital resources. However, with no vaccines or preventative interventions available to protect against COVID-19, current strategies rely on conventional control measures including travel restrictions, quarantines and increased hygiene practices. The overlap of COVID-19 symptoms with common respiratory diseases makes screening for SARS-CoV-2 infection difficult with diagnosis relying on microbiological confirmation of SARS-CoV-2 infection. Moreover, healthcare workers with these common respiratory symptoms are advised to be tested for SARS-CoV-2 infection prior to return to work. The loss of these healthcare workers with non-COVID-19 respiratory infections due to quarantine requirements places further pressure on the healthcare system during this critical time.

BCG, a vaccine given to over 120 million infants annually to protect against TB, represents a potential prophylactic intervention for the prevention of COVID-19. In addition to protecting against TB, BCG has beneficial off target (also termed 'heterologous' or 'non-specific') effects that protect against unrelated infections in children and adults[7-11].

The beneficial off-target effects of BCG vaccination have been most extensively studied in children. A world health organisation (WHO)-commissioned meta-analysis of 12 studies in high mortality settings concluded that BCG vaccination reduces all-cause mortality in children under 5-years of age by 30-53%[8]. This protection is evident within days of vaccination is proposed to be attributable to reduced deaths from infections other than TB, particularly respiratory tract infections and sepsis. Two large cohort studies in children similarly found that BCG reduces non-TB infections. The first, a 25-year retrospective study of over 150,000 children from 33 countries reported that BCG-vaccinated children had an up to 37% lower risk of acute lower respiratory tract infections[12]. The second, a study of paediatric hospitalisations in Spain, found that BCG-vaccinated children had a 41% lower risk of serious respiratory infection and 53% lower risk of sepsis not related to TB[13].

In adults, in a human challenge model, prior BCG vaccination reduced viraemia by over 70% and improved anti-viral immune responses to yellow fever vaccine virus[14]. Notably, yellow fever virus is a single-stranded, positive-sense RNA virus like SARS-CoV-2. Consistent with BCG mediated protection against infections, in two randomised control trials in adults, BCG vaccination reduced incidence of acute upper tract respiratory infections by 70-80%[15,16]. Several studies have also shown that BCG can reduce symptoms in human papilloma virus infection and herpes simplex virus infection adults[17].

A plethora of studies in animal models, have also shown that BCG protects against disease and mortality caused by a wide range of bacterial, fungal, protozoan and viral infections including infections with single-stranded, positive-sense RNA viruses[18-20].

The beneficial off-target effects of BCG are proposed to result from BCG induced changes in immune responses[1,14,19]. In adults, BCG vaccination increases immune responses to unrelated pathogens, an effect that is sustained for at least a year after vaccination[21]. BCG vaccination also boosts antibody responses to several vaccines including influenza vaccine[22-24]. Thus, in addition to protecting against viral infections, BCG provides further protection by increasing the efficacy of other vaccinations.

Therefore, by boosting the immune system, BCG vaccination may provide early protection against new human pathogens thus reducing their spread and severity. This will be of particular benefit among healthcare workers and high-risk groups for whom contraction of the disease would have the greatest impact.

This trial will determine whether BCG vaccination reduces the incidence and severity of COVID-19 but also whether BCG vaccination reduces other respiratory illnesses in healthcare workers. In this case of COVID-19, where symptoms overlap with common respiratory diseases and diagnostic tests currently take several days, the prevention of non-CODVID-19 respiratory illnesses will also reduce the strain on the healthcare system caused by the outbreak. This is particularly important in Australia and other countries in the southern hemisphere as the outbreak peak is expected to occur during the winter influenza season.

The results of this trial will establish whether, in future novel disease outbreaks, BCG vaccination could be implemented as an early intervention to protect healthcare workers and high-risk groups.

2.3. Risk/Benefit assessment

2.3.1. Known potential risks

This study involves minimal risk to participants.

HCWs randomised to receive BCG vaccine will have known potential risks associated with BCG vaccination. These risks are slightly increased for HCWs who have previously had BCG vaccine (revaccination), compared to HCWs receiving BCG vaccine for the first time (vaccine naïve).

There are additional known minimal risks for all HCWs re: blood tests and respiratory swabs.

<u>BCG vaccination</u>
Expected (common) reactions to BCG vaccination[25]:

- A small swelling, redness and tenderness (measuring 0.5-1.5 cm in diameter) at the injection site appears within 1-2 weeks at the injection site. The local lesion evolves into a small ulcer. The ulcer heals over several weeks to months, usually healing into a small flat scar.
- Slightly swollen lymph nodes in the axilla in up to 10% of recipients, and usually resolve spontaneously.

Revaccination is associated with an earlier, accelerated reaction which begins within 24–48 hours of vaccination with induration followed by pustule formation in 5–7 days and healing within 10–15 days[26] *(*https://www1.health.gov.au/internet/main/publishing.nsf/Content/cda-cdi3701h.htm)
Tuberculous skin lesions are more common in people over 15 years or with revaccination)[27,28].

Uncommon side effects of BCG vaccination (up to 1 in 100)[25,29-31]:

- Large ulcer, abscess at the injection site
- Keloid scar at injection site
- Swelling of lymph nodes in the armpit larger than 1 cm across

Rare side effects (up to 1 in 1000)

- Significant inflammation of lymph nodes in the axilla, sometimes with oozing ulcers, possibly abscess
- Infection with the bacteria from the vaccine can occur. The infection can spread throughout the body, including the bones (osteomyelitis)
- Allergic reaction or anaphylaxis (e.g.: redness of the face and neck, swelling of the face, throat or neck, skin rash, breathing difficulties and collapse)
- Fainting, seizures and convulsions (rare among patients receiving injections)

Very rare side effects (1–4 cases per million vaccinated people[25]):

- Disseminated BCG infection has been reported rarely after BCG vaccination, mainly in immunocompromised individuals (who are excluded from the trial).

Co-administration of vaccines:

- As indicated in the Australian immunisation handbook, BCG vaccine can be given at the same time as, or at any time after, other inactivated vaccines thus there is no additional risk for co-administration of influenza and BCG vaccines[25].

BCG vaccination in Europe (current recommendations) [32]

In Europe, recommendation for BCG vaccination varies among countries. In some, BCG is no longer recommended (e.g. Spain), whereas in others it is given routinely to all neonates (mainly Eastern Europe).[32] In the Netherlands it is limited to the children of parents from countries with a high incidence of tuberculosis (>50/100,000 people) and is not routinely recommended for healthcare workers.[33] In the UK, routine BCG vaccination of adolescents was stopped in 2005, with subsequent efforts focusing on high-risk groups for tuberculosis (UK 'Green Book' chapter 32).

BCG vaccination in Australia (current recommendations)

BCG vaccination in Australia is limited to selected high risk groups and is not routinely recommended for most healthcare workers (HCW)[26]. BCG vaccination is recommended for Aboriginal and Torres Strait Islander neonates in communities with a high incidence of TB; neonates and children 5 years of age and under who will be travelling or living in areas with a high prevalence of TB for extended periods; and neonates born to parents with leprosy. BCG should be considered in HCWs who may be at high risk of exposure to drug resistant cases. It is usually recommended that all individuals have a tuberculin skin test (TST) prior to BCG vaccination, except infants less than 6 months of age with no history of tuberculosis (TB) contact, and that BCG should not be given to an individual with a tuberculin reading of 5mm or more. Additionally, BCG revaccination is not recommended, regardless of TST reaction size[26].

BCG vaccination in Brazil (current recommendations)

In Brazil, BCG vaccination has been mandatory since 1976 in newborns. Revaccination in school-aged children (<6 years) was suspended in 2019. The REVAC trial evaluated adverse reactions resulting from BCG vaccination and revaccination in 71,347 Brazilian school-aged children. The authors concluded that the rate of adverse reactions associated with BCG revaccination is approximately twice the rate associated with vaccination, but this difference was not statistically significant. Similar results have been observed in previous studies that concluded that BCG revaccination is not associated with a higher rate of serious adverse events than primary BCG vaccination.[43,44]

Current contraindications of BCG vaccination

- BCG is contraindicated in immunocompromised individuals due to the risk of disseminated BCG infection[26]. This includes individuals immunocompromised by HIV infection, primary immunodeficiencies, corticosteroids or other immunosuppressive agents, and malignancies involving bone marrow of lymphoid systems.
- BCG is also contraindicated in individuals with any serious illness and those with generalised septic skin diseases and active skin conditions such as eczema, dermatitis and psoriasis near the site of vaccination[25].

- While BCG has not been shown to cause foetal damage the use of live vaccines is contraindicated in pregnancy [26].
- Individuals who have previously had tuberculosis or a large tuberculin (TST) reaction

In this study, HCWs will be excluded from the study if they are immunocompromised, have serious illness, skin disease at site of vaccination or are pregnant.

Global BCG recommendations and practices

The current World Health Organization (WHO) position is that BCG revaccination is not recommended for any person, as there is no evidence to support the role of BCG revaccination in protection against tuberculosis[34]. A number of countries have previously included BCG revaccination as part of their national immunisation policies[35]. In 1999, 30 countries in Europe and an additional 18 countries in the Middle East, South East Asia and the Western Pacific region reported using BCG revaccination. In several countries the national policy included BCG in infancy and again at school entry or leaving. In other countries, particularly in Eastern Europe, revaccination with BCG up to age five has been recommended. Some countries, such as Poland, recommended universal revaccination while others restrict revaccination to individuals without a BCG scar or those with a 'negative' TST. Criteria for TST negativity differs between countries [36,37]. ***In countries where BCG revaccination has been part of national immunisation practice, passive surveillance has not reported any particular issues, nor any cases of disseminated BCG in immunocompetent individuals.***

Pre-vaccination screening

TST and interferon gamma release assay (IGRA) screening aims to identify individuals with latent tuberculosis infection (LTBI)[38]. The diameter of induration following TST gives an indication of the likelihood of LTBI, however, positive results can also arise from previous BCG vaccination and exposure to environmental mycobacteria. This is in contrast to IGRA which are unaffected by previous BCG vaccination. A positive IGRA indicates either current or past infection with TB[38]. Screening of individuals using TST prior to BCG vaccination is recommended in Australia and other countries on the grounds that it may prevent complications due to pre-existing immunity due to previous exposure to mycobacterial antigens [28]. ***However, a large review of adverse effects of over 1.5 billion doses of BCG vaccine in adults and children showed that a positive TST did not increase the likelihood of complications from the BCG vaccine and did not predict the development of local skin reactions, abscesses or axillary lymphadenitis[27].***

Trials of BCG revaccination

Three large randomised controlled trials of BCG revaccination in children and adults in Malawi (n=54865), children in Guinea Bissau (n=2871) and adolescents in South Africa (n=990) did not show increased rates of serious adverse events among BCG revaccinated participants[15,39,40]. Participants in the Malawi study did not undergo any pre-randomisation screening with tuberculin skin test (TST) or interferon gamma release assay (IGRA)[39]. This study found a lower rate of leprosy amongst revaccinated participants but no difference in the rates of tuberculosis or death between the groups. Of the children in the Guinea Bissau

study, 3 of 6 children with a measurable TST (1-14mm) had increased rates of large local reaction compared to controls (18/388). Two months after revaccination all had healed vaccination scars with no axillary node enlargement, fever or suppurative lymphadenitis[40]. Participants in the South African study all had a negative IGRA at enrolment[15]. Among BCG revaccinated adolescents 93% reported mild local injection site reactions including swelling, induration, discharge, erythema, scab and ulceration. This was compared to 25% in the placebo group. The rates of moderate injection site reactions were similar between the BCG (5%) and placebo (6%) groups. There was 1 severe and 7 serious adverse events in each of the BCG and control groups. The serious adverse events reported in the BCG arm were not attributed to BCG revaccination and included gastroenteritis, chest injury, thermal burn, intentional self-injury, suicide attempt and small intestinal obstruction. The rate of upper respiratory tract infections was also lower in the BCG revaccinated group compared to placebo (2.1% compared to 7.9%, $p<0.001$).

Further studies looking at BCG revaccination in individuals with positive TST or IGRA do not show increased risk of significant adverse effects. A case-control study of 200 healthy nursing students in India included 28 participants with a positive IGRA who received BCG revaccination[41]. There were no serious side effects reported and no participants developed active tuberculosis during the follow-up study period. A randomised controlled trial of BCG revaccination in healthy adults with a positive TST (>15mm) with or without isoniazid pre-treatment (n=82) showed no difference in the rate of reactions between groups with only local injection site reactions (35-76%) and mild systemic adverse effects (19%) including headache, fever and nausea[42]. Among the 76% of participants who developed ulceration the median ulcer size was 5mm (IQR 4.0-6.0). Maximum ulcer diameter did not correlate with IGRA result prior to BCG vaccination in either group. There were no reports of regional lymphadenitis or serious morbidity.

Enhanced routine passive surveillance of BCG revaccinated school children in the BCG-REVAC trial in Brazil is available for 71718 individuals[43]. There are only 33 reported adverse events of which 60% were local cutaneous reactions and 28% axillary lymphadenopathy without suppuration. There were no deaths, permanent injuries or disseminated infections reported. In a case series of 13 children who experienced adverse events following BCG revaccination in Brazil all developed local ulceration or abscess formation with complete recovery following antimycobacterial therapy[44]. There were no cases of suppurative lymphadenitis or disseminated BCG. Further, an ongoing randomised trial in 150 participants in the US is giving repeat BCG (two vaccinations in the first year, then annually for 4 years) to adults aged 18-65 with type 1 diabetes to test if multiple BCG vaccinations can improve diabetic control and prevent complications[45]. They have reported variable local reactions but no increased risk of lymphadenopathy or disseminated BCG (Denise Faustman, personal communication).

The data presented above supports the WHO position that while BCG revaccination is not recommended due to a lack of evidence of efficacy against tuberculosis the risk of administering BCG vaccine to persons with positive tuberculin reactions due to either prior BCG vaccination or to natural infection is minimal.

One aim of the present study is to document the safety of BCG vaccination (and revaccination) in healthcare workers. The decision not to perform pre-vaccination TST screening in the study is pragmatic in order to reduce barriers to participation for already busy and stretched

healthcare workers during the current COVID-19 outbreak. While it does not align with current Australian vaccination guidelines it has been carefully considered upon systematic review of the literature presented above.

Risks related to Placebo injection

Having an injection can sometimes cause very minor pain from the needle or be uncomfortable. The 0.9% NaCl is an inert salt solution that will not cause any degree of local reaction. The placebo injection will be administered by a trained immunization nurse.

Risks related to blood sample collection

Having a blood test can sometimes cause some pain from the needle or be uncomfortable. Occasionally a small amount of bruising can occur on the skin where the blood was taken. Trained members of the study team will collect the blood samples from participants.

Risks related to respiratory swab collection

Having a respiratory swab can sometimes be uncomfortable. Trained members of the study team will collect the respiratory swabs from participants. Self-testing swab kits may be provided as required, with clear instructions to participants on safe self-swabbing technique.

2.3.2. Known potential benefits

In most places in the world, BCG is given to infants and children living in or travelling to TB endemic areas. In adults its efficacy is variable, and likely to have little effect in adults living in low prevalence settings (such as Australia, UK, Spain or the Netherlands) as their risk of TB is very low. BCG also protects against non-TB mycobacterial infections (e.g. leprosy, Buruli ulcer) but these are also rare in Australia and in Europe.

However, BCG also induce beneficial off target effects, and therefore BCG vaccination may reduce COVID-19 illness and other respiratory infections in study participants. In addition to the direct benefit this would give the participants by reducing disease, this would also benefit the healthcare facilities that they work at by reducing their need to be absent (symptom related quarantine or illness) and thus enabling them to continue working and supporting the healthcare system during this period of intense demand.

2.3.3. Assessment of potential risks and benefits

BCG vaccination has a well-established safety profile in healthy individuals. While there are known adverse reactions to BCG, serious adverse reactions are rare. BCG vaccination does also cause a scar in over 80% participants. Participants will be screened prior to BCG vaccination to ensure they have no known contraindications for BCG vaccination. Vaccination will be done by staff trained in intradermal injection to reduce the potential subcutaneous injection which can increase scarring. Blood tests and respiratory swabs will be done by trained staff. If necessary (e.g. insufficient testing capacity or personal protective equipment) participants may be asked to self-collect throat/nose swabs for later collection by study staff.

Given the minor risks of BCG vaccination, the potential benefits of BCG vaccination for the participants (by reducing COVID-19 and other respiratory infections), the healthcare system (by reducing absenteeism) during this current COVID-19 outbreak far outweigh them. In addition to this current outbreak, the findings of this study have major implications for future outbreak responses globally. If BCG vaccination is found to be effective at reducing COVID-19,

BCG vaccination could be implemented as an early preventative intervention in future outbreaks to protect healthcare workers globally. BCG vaccine is cheap and already administered to infants in over 80% of countries worldwide, therefore implementation of BCG vaccination campaigns during outbreaks is a feasible intervention to complement other preventative strategies.

We will be using BCG vaccine outside of its standard/recommended use, therefore, as per use of any intervention outside of standard regulations we will be assessing the reactogenicity and safety of BCG vaccination in vaccine naïve and previously vaccinated healthcare workers.

Risks will be continuously reviewed by continuously checking the literature and communicating with the other research group doing similar BCG trials. We have planned an interim analysis as well within our own cohort.

3 TRIAL OBJECTIVES AND OUTCOMES

3.1 Objectives

Two primary outcomes have been chosen for this study: occurrence of symptomatic COVID-19 and occurrence of severe COVID-19. Considering the number of unknown factors and the little knowledge of this new virus, we deemed it of clinical importance to have sufficient power to detect the potential effect of BCG vaccine compared to control for both outcomes (occurrence of symptomatic COVID-19, as well as occurrence of severe COVID-19). Our hypothesis is that, compared to control, the BCG vaccine will reduce both the number of cases of COVID-19 (increase the number of asymptomatic SARS-CoV-2 infections) and the number of severe cases of COVID-19. In other words, we have the hypothesis that BCG vaccine would be able to shift the “severity of COVID-19” curve down, i.e. to generally reduce the severity of the symptoms in healthcare workers. Because of the potential for multiplicity testing, the method of controlling type I error is explained in the sample size section (11.1).

3.1.1 Primary objective

1. To determine if BCG vaccination (Intervention) compared with placebo (Comparator) reduces the incidence of symptomatic COVID-19 (Outcome) measured over the 6 months following randomisation (Time) in healthcare workers (Participants).
2. To determine if BCG vaccination (Intervention) compared with placebo (Comparator) reduces the incidence of severe COVID-19 (COVID 19 related death, hospitalisation, or non-hospitalised severe disease (defined as Non-ambulant[1] for ≥ 3 consecutive days OR Unable to work[2] for ≥ 3 consecutive days) (Outcome) measured over the 6 months following randomisation (Time) in healthcare workers (Participants).

[1] “pretty much confined to bed (meaning finding it very difficult to do any normal daily activities”

[2]“I do not feel physically well enough to go to work”

3.1.2 Secondary objectives

3. To determine if BCG vaccination (Intervention) compared with placebo (Comparator) reduces the incidence of symptomatic COVID-19 (Outcome) measured over the 12 months following randomisation (Time) in healthcare workers (Participants).

4. To determine if BCG vaccination (Intervention) compared with placebo (Comparator) reduces the incidence of severe COVID-19 (non-hospitalised severe disease, hospitalisation or death) (Outcome) measured over the 12 months following randomisation (Time) in healthcare workers (Participants).
5. To determine if BCG vaccination (Intervention) compared with placebo (Comparator) prolongs the time to first COVID-19 episode (Outcome) measured over 6 and 12 months following randomisation (Time) in healthcare (Participants).
6. To determine if BCG vaccination (Intervention) compared with placebo (Comparator) reduces the severity of COVID-19 (Outcome) measured over 6 and 12 months following randomisation (Time) in healthcare workers (Participants).
7. To determine if BCG vaccination (Intervention) compared with placebo (Comparator) reduces the rate and severity of illness (fever or at least one sign or symptom of respiratory disease) measured over 12 months following randomisation (Time) in healthcare workers (Participants).
8. To determine if BCG vaccination (Intervention) compared with placebo (Comparator) reduces absenteeism (days off work) over 6 and 12 months following randomisation (Time) in healthcare workers (Participants).
9. To evaluate the safety of BCG vaccination in healthcare workers.

3.1.3 Planned exploratory analyses

10. To determine in a subgroup of adults with recurrent cold sores whether BCG vaccination compared with placebo reduces herpes simplex recurrences (such as cold sores).
11. To determine the BCG vaccination induces changes in the immune system that are associated with protection of healthcare workers from non-tuberculous infectious diseases including COVID-19.
12. To determine and compare changes in the immune system induced by vaccination of healthcare workers.
13. To identify factors (e.g. age, sex, chronic conditions such as diabetes and cardiovascular disease, smoking, asthma, prior BCG vaccination, genetics, other vaccinations including COVID-19-specific vaccines, latent TB, immunological/molecular factors) that influence immune responses, infection and COVID-19 risk.
14. *(Brazil specific) To identify biomarkers for diagnosing TB infection*

3.2 Outcomes

OBJECTIVE	OUTCOME & OUTCOME MEASURE
Primary	
1. To determine if BCG vaccination (Intervention) compared with placebo (Comparator) reduces the incidence of symptomatic COVID-19 (Outcome) measured over the 6 months following randomisation (Time) in healthcare workers (Participants).	Symptomatic COVID-19 by 6 months following randomisation defined as:<br><br>Case definition<br>• Positive SARS-CoV-2 test (PCR, RAT or serology), plus<br>• Fever (using self-reported questionnaire), OR<br>• At least one sign or symptom of respiratory disease including cough, sore throat, shortness of breath, respiratory distress/failure (using self-reported questionnaire)"
2. To determine if BCG vaccination (Intervention) compared with placebo (Comparator) reduces the incidence of severe COVID-19 (with COVID related hospitalisation, death, or non-hospitalised severe disease) (Outcome) measured over the 6 months following randomisation (Time) in healthcare workers (Participants).	Severe COVID-19 by 6 months following randomisation defined as<br><br>Severe COVID-19 by 6 months following randomisation defined as:<br><br>Case definition<br>• Positive SARS-CoV-2 test (PCR, RAT or serology), PLUS<br>• Death as a consequence of COVID-19, OR<br>• Hospitalised as a consequence of COVID-19, OR<br>• Non-hospitalised severe disease as a consequence of COVID-19, defined as non-ambulant[1] for ≥ 3 consecutive days or unable to work[2] for ≥ 3 consecutive days<br><br>[1] *"pretty much confined to bed (meaning finding it very difficult to do any normal daily activities"*<br><br>[2] *"I do not feel physically well enough to go to work" (excludes stay at home exclusively for quarantine/workplace restrictions)*
Secondary	
3. To determine if BCG vaccination (Intervention) compared with placebo (Comparator) reduces the incidence of symptomatic COVID-19 (Outcome) measured over the 12 months following randomisation (Time) in healthcare workers (Participants).	Symptomatic COVID-19 as defined above over the 12 months following randomisation
4. To determine if BCG vaccination (Intervention) compared with placebo (Comparator) reduces the incidence of severe COVID-19 (COVID related hospitalisation, death, or non-hospitalised severe disease) (Outcome) measured over 12 months following randomisation (Time) in healthcare workers (Participants).	Severe COVID-19 as defined above over the 12 months following randomisation
5. To determine if BCG vaccination (Intervention) compared with placebo (Comparator) prolongs the time to first COVID-19 episode (Outcome) measured over 6 and 12 months following randomisation (Time) in healthcare workers (Participants).	Time to first symptom of COVID-19 over the 6 and 12 months following randomisation.
6. To determine if BCG vaccination (Intervention) compared with placebo (Comparator) reduces the severity of COVID-19 (Outcome) measured over 6 and 12 months following randomisation (Time) in healthcare workers (Participants).	All the following measures will be assessed, over the 6 and 12 months following randomisation.<br><br>• Number of episodes of COVID-19 disease as (defined above)<br>• Asymptomatic SARS-CoV-2 infection (defined as:

	- Evidence of SARS-CoV-2 infection (by seroconversion) and<br>- Absence of any episodes of illness (using self-reported questionnaire) and<br>- No evidence of exposure prior to randomisation<br>• Number of days unable to work (using self-reported questionnaire) due to COVID-19 as defined above (excludes quarantine/workplace restrictions)<br>• Number of days confined to bed (using self-reported questionnaire) due to COVID-19 as defined above<br>• Number of days with symptoms due to for COVID-19<br>• Pneumonia cases (using self-reported questionnaire and/or medical/hospital records) due to COVID-19.<br>• Need for oxygen therapy (using self-reported questionnaire and/or medical/hospital records) due to COVID-19<br>• Admission to critical care and duration of stay (using self-reported questionnaire and/or medical/hospital records) due to COVID-19.<br>• Need of mechanical ventilation and duration (using self-reported questionnaire and/or medical/hospital records)<br>• Hospitalisation and duration due to COVID-19 (using self-reported questionnaire and/or medical/hospital records)<br>• Death due to COVID 19
7. To determine if BCG vaccination (Intervention) compared with placebo (Comparator) reduces the rate and severity of illness (fever or at least one sign or symptom of respiratory disease) measured over the 12 months following randomisation (Time) in healthcare workers (Participants).	All the following measures will be assessed, over the 12 months following randomisation<br><br>For the following outcomes, fever or respiratory illness will be defined as:<br>- fever (using self-reported questionnaire), or<br>- at least one sign or symptom of respiratory disease including cough, sore throat, shortness of breath, respiratory distress/failure, or runny/blocked nose (in combination with another respiratory symptom or fever)<br>fever or respiratory illness, as defined above<br>• Severe fever or respiratory illness defined as:<br>- Death, OR<br>- Hospitalised, OR<br>- Non-hospitalised severe disease, defined as non-ambulant[1] for ≥ 3 consecutive days or unable to work[2] for ≥ 3 consecutive days<br><br>as a consequence of fever or respiratory illness, as defined above<br><br>[1] *"pretty much confined to bed (meaning finding it very difficult to do any normal daily activities"*<br><br>[2] *"I do not feel physically well enough to go to work" (excludes stay at home exclusively for quarantine/workplace restrictions)*<br><br>• Number of episodes of fever or respiratory illness, as defined above<br>• Number of days unable to work (using self-reported questionnaire) due to fever or respiratory illness, as defined above (excludes quarantine/workplace restrictions)

	• Number of days confined to bed (using self-reported questionnaire) due to fever or respiratory illness, as defined above<br>• Number of days with symptoms due to fever or respiratory illness<br>• Pneumonia within a febrile or respiratory illness (using self-reported questionnaire and/or medical/hospital records)<br>• Need for oxygen therapy for a febrile or respiratory illness (using self-reported questionnaire and/or hospitalisation records)<br>• Admission to critical care for a febrile or respiratory illness (using self-reported questionnaire and/or medical/hospital records)<br>• Need of mechanical ventilation for a febrile or respiratory illness (using self-reported questionnaire and/or medical/hospital records)<br>• Deaths as a consequence of an episode of fever or respiratory illness<br>• Duration of hospitalisation for a febrile or respiratory illness (using self-reported questionnaire and/or medical/hospital records)
8. To determine if BCG vaccination (Intervention) compared with placebo (Comparator) reduces absenteeism (days off work) over 6 and 12 months in healthcare workers (Participants).	Number of days of unplanned absenteeism for an acute illness or hospitalisation (using self-reported questionnaire) within 6 and 12 months following randomisation
9. To evaluate the safety of BCG vaccination in healthcare workers.	Adverse events (AEs), over the 3 months following randomisation, by type, severity (graded using toxicity grading scale), relationship to intervention of adverse events (AEs) of interest.<br><br>Serious Adverse Events, over the 3 months following randomisation
Exploratory analyses	
10. To determine in a subgroup of participants with recurrent cold sores whether BCG vaccination compared with placebo reduces herpes simplex recurrence (such as cold sores).	Herpes simplex recurrences (self-reported) over the 12 months following randomisation<br><br>Number of episodes of herpes simplex recurrence (self-reported) over the 12 months following randomisation<br><br>Time: to first of herpes simplex recurrence (self-reported) over the 12 months following randomisation
11. To determine the BCG vaccination-induced changes in the immune system that are associated with protection against non-tuberculous infectious diseases including COVID-19.<br><br>12. To determine and compare changes in the immune system induced by vaccination.	The immune system will be assessed by several methods including:<br>- Cytokine levels in supernatants from whole blood stimulated with off-target pathogens (including BCG, *Staphylococcus aureus*, *Escherichia coli*) and Toll-like receptor (TLR) agonists, measured by multiplex<br>- Cytokine production, activation and differentiation of immune cells (measured by flow cytometry)<br>- Epigenetic modifications (e.g. histone methylation/acetylation and CpG methylation) measured by ChIP-Seq and/or microarray<br>- Anti-vaccine and anti-pathogen (including SARS-CoV2) antibody levels measured by ELISA, multiplex or VirScan<br>- RNA expression measured by qRT-PCR or RNA-Seq

13. To identify factors (e.g. age, sex, chronic conditions such as diabetes and cardiovascular disease, smoking, asthma, prior BCG vaccination, genetics, other vaccinations including COVID-19-specific vaccines, latent TB, immunological/molecular factors) that influence immune responses, infection and COVID-19 responses.	Association of demographic factors, exposure, genetic factors (e.g. single nucleotide polymorphisms) and immune factors (e.g. cell numbers, circulating cytokines, anti-vaccine/anti-pathogen antibodies) with the function of the immune system as described above and infection (including COVID-19) prevalence or severity as defined above
14. (Brazil specific) To identify biomarkers for diagnosing TB infection	Association of biomarkers (e.g. cytokines measured from QFT-Plus supernatants, whole blood transcriptional signature) with TB infection

4 TRIAL DESIGN

4.1 Overall design

This is a phase III, two group, multicentre, randomised placebo-controlled trial in up to 7244 frontline healthcare workers to determine if BCG vaccine reduces prevalence and the severity of COVID-19 during the 2020, SARS-CoV-2 pandemic. As part of this study, we plan to combine the data from this study in a pre-planned meta-analysis with data from the 2834 participants recruited in the first stage of this study which followed the same protocol but where participants were randomised between BCG and no BCG at the time of receiving an influenza vaccination, for a total sample size of 10078. Although we recognise that the first stage of this study was addressing a slightly different research question, we feel that it is important to combine data from the first stage of study as they both provide estimates of the efficacy of the BCG vaccination, which is critical to provide adequate power to determine the efficacy of the BCG vaccination. In Europe, there will be participating sites in the Netherlands, Spain and UK. The sites participating in the Netherlands are Noordest Ziekenhuis Alkmaar, Rijnstate Hospital Arnhem, Amphia Hospital Breda, St Antonius Hospital Nieuwegein, Radboud UMC Nijmegen and Universitair Medisch Centrum Utrecht. In Spain, University Hospital German Trias I Pujol Barcelona, Mutua Terrassa Univeristy Hospital Barcelona, University Hospital Cruces Bizkaia, Marqués de Valdecilla University Hospital Santander and University Hospital Virgen Macarena Sevilla. In United Kingdom, Teign Estuary Medical Group Devon, Ide Lane Surgery Exeter, St Leonard's Practice Exeter, Travel Clinic Exeter and Royal Devon and Exeter NHS Foundation Trust Exeter.

In Australia, participating sites are hospitals within Victoria, Western Australia, South Australia and New South Wales. Australian sites involved in this study include the RCH VIC, Monash Health VIC, Epworth Healthcare VIC, Perth Children’s Hospital WA, Fiona Stanley Hospital WA , Sir Charles Gairdner Hospital WA, Royal Adelaide Hospital SA, Women’s and Children’s Hospital Adelaide SA, The Children’s Hospital at Westmead NSW, Westmead Hospital NSW, Prince of Wales Hospital NSW, St Vincent’s Hospital Sydney NSW and Sydney Children’s Hospital, Randwick NSW.

In Brazil, there will be participating sites in Mato Grosso do Sul State, Rio de Janeiro and Amazonas. In Mato Grosso do Sul the principal site will the Faculty of Medicine of the Federal University of Mato Gross do Sul (UFMS) with additional locations: Regional Hospital of Mato Grosso do Sul, Hospital CASSEMS, Hospital Santa Casa and Municipal Health Units. In Rio de Janeiro the principal site will the Centro de Referência Prof Hélio Fraga (CRPFH) da Escola Nacional de Saúde Pública Sérgio Arouca (ENSP), FIOCRUZ and Municipal Health Office of Rio de Janeiro. Other sites in Rio de Janeiro and Mato Grosso do Sul will be identified. In

Amazonas the principal site will be Fundação de Medicina Tropical and Health State Office. Other sites in Brazil may be identified.

Recruitment may be held at participating sites or centrally identified locations with appropriate safety and privacy infrastructure.

Participants will be randomised to receive BCG or placebo.

During the first stage of the study in Australia, randomisation and immunisation coincided with the annual staff influenza immunisation roll out at each hospital. During the first stage of the study, influenza vaccine occurred at the same time as randomisation and BCG vaccination or no BCG for 2834 healthcare workers. During the second stage in locations where the annual influenza vaccine is available, participants are asked to confirm they have received the influenza vaccination a minimum of 72 hours prior to randomisation.

The control group will receive a placebo injection of 0.9% NaCl. Most people vaccinated with the BCG vaccine develop a papule/blister at the injection site around two-weeks after vaccination. Due to this, even using a placebo, it is not possible to completely blind participants to their treatment group allocation. The outcomes (symptomatic and severe COVID-19 or admission to hospital for COVID-19) are objective measures, it is however still plausible that participant's suspicion of their group allocation might bias the study results. This risk will be mitigated by using a placebo where an element of doubt over treatment allocation may persist even in the absence of scar formation. Members of the research team doing follow-up, data cleaning and analysis will be blinded to the group allocation (by the hiding of this variable and all other variables related to BCG from the dataset) until the formal detailed statistical analysis plan is confirmed and signed by all investigators and all data cleaning/preparation is complete.

Randomisation will be stratified for all factors that might influence the effectiveness of the intervention. For more details see section 6.

Follow-up for all participants will last 1-year. For each episode of fever with a respiratory symptom during the follow-up period, all participants complete a survey in a smartphone app, electronic message or by phone, and may have a home visit by members of the research team for sample collection (e.g. if the government ceases or limits COVID-19 testing; respiratory swab preferred, however blood sample will be taken if no swab testing kits are available). If necessary (e.g. insufficient testing capacity or personal protective equipment) participants may be asked to self-collect throat/nose swabs for later collection by study staff, or self-test a finger-prick blood sample and send a photo of the results to the study team.

4.2 Justification for dose

The dose and route of BCG administration are the standard accepted dosage for BCG vaccine when used to prevent TB. There is no justification to vary from this.

4.3 Trial population

4.3.1 Eligibility criteria

Participants will be assigned to a randomised trial treatment only if they meet all inclusion criteria and no exclusion criteria.

As soon as COVID-19-specific vaccine becomes available, for sites still recruiting participants into the BRACE trial, the site's study team will have to inform the participant before they provide consent, that there will be a delay in receiving their COVID-19-specific-vaccine by either (1) at least 7 days following BCG/placebo injection OR (2) in accordance with their relevant vaccine national guidelines whichever is the longest.

4.3.2 Inclusion criteria

- Over 18 years of age
- Healthcare worker
  - This is defined as anyone who works in a healthcare setting or has face to face contact with patients.
- Provide a signed and dated informed consent form
- Australian sites only: If annual influenza vaccination is available, receiving the influenza vaccine is an eligibility requirement. The influenza vaccine will be required a minimum of 3 days in advance of randomisation in the BRACE trial.
- Pre-randomisation blood collected

4.3.3 Exclusion criteria

- Has any BCG vaccine contraindication
  - o Fever or generalised skin infection (where feasible, randomisation can be delayed until cleared)
  - o Weakened resistance toward infections due to a disease in/of the immune system
  - Receiving medical treatment that affects the immune response or other <u>immunosuppressive therapy</u> in the last year.
      - These therapies include systemic corticosteroids (≥20 mg for ≥2 weeks), non-biological immunosuppressant (also known as 'DMARDS'), biological agents (such as monoclonal antibodies against tumour necrosis factor (TNF)-alpha).
    - People with congenital cellular immunodeficiencies, including specific deficiencies of the interferon-gamma pathway
    - People with malignancies involving bone marrow or lymphoid systems
  - o People with any serious underlying illness (such as malignancy)
    - NB: People with cardiovascular disease, hypertension, diabetes, and/or chronic respiratory disease are <u>eligible</u> if not immunocompromised, and if they meet other eligibility criteria

- Known or suspected HIV infection,[11] even if they are asymptomatic or have normal immune function.
  - This is because of the risk of disseminated BCG infection[12,13]
- People with active skin disease such as eczema, dermatitis or psoriasis at or near the site of vaccination
  - A different adjacent site on the upper arm can be chosen if necessary
- Pregnant
  - Although there is no evidence that BCG vaccination is harmful during pregnancy, it is a contraindication to BCG vaccination. Therefore, we will exclude women who think they could be pregnant or are planning to become pregnant within the next month.
  - UK specific: Although there is no evidence that BCG vaccination is harmful during pregnancy, it is a contraindication to BCG vaccination. Therefore, we will exclude women of childbearing potential (WOCBP) who think they could be pregnant. See section 8.2 for definition of WOCBP and Appendix 3 for UK specific pregnancy test requirements.
  - Spain specific: If the patient is female, and of childbearing potential, she must have a negative pregnancy test (provided by Sponsor) at the time of inclusion and practice a reliable method of birth control for 30 days after receiving the BCG vaccination. See Appendix 6 for Spain specific requirements
- Another live vaccine administered in the month prior to randomisation
- Require another live vaccine to be administered within the month following BCG randomisation
  - If the other live vaccine can be given on the same day, this exclusion criteria does not apply
- Known anaphylactic reaction to any of the ingredients present in the BCG vaccine
- Previous active TB disease

- Currently receiving long term (more than 1 month) treatment with isoniazid, rifampicin or quinolone as these antibiotics have activity against *Mycobacterium bovis*
- Previous adverse reaction to BCG vaccine (significant local reaction (abscess) or suppurative lymphadenitis)
- BCG vaccine given within the last year

- Have previously had a SARS-CoV-2 positive test result (positive PCR on a respiratory sample or a positive SARS-CoV-2 diagnostic RAT test approved by the local jurisdiction's public health policy)
- Already part of this trial, recruited at a different site/hospital.
- Participation in another COVID-19 prevention trial
- Have previously received a COVID-19-specific vaccine

4.4 Lifestyle considerations

Not applicable

4.5 Screen failures

Screen failures are defined as participants who consent to participate in the trial but who are found, during the screening procedures, to be ineligible to continue into the trial. They therefore do not receive the intervention / are not randomised.

4.6 Recruitment and Consent

Potential participants will receive information (via email, healthcare facilities notice board and/or website/social media etc) about the trial. This will include a short blurb about the study and a link to a website where they can read further information contact details for further questions. Potential participants will be able to evaluate their eligibility online via the REDCap public link and having met the eligibility criteria access the site-specific participant information and consent form (PICF) prior to attending clinic.

Interested healthcare workers will be given the opportunity to talk with a member of the research team by phone or video conferencing if they have any questions (social distancing practices will still be applied wherever possible). The process will vary slightly between locations due to contextual adaptations, however, it will be built around the same core essentials:

- Providing accurate information regarding the trial through a combination of publicly available information and additional detailed explanation by trained study staff.
- Eligibility screening for all participants. If participants are ineligible no identifying information will be collected.
- Informed consent secured from all participants through signed (electronic or hard copy) PICFs. Consent will be voluntary and free from coercion.
- Study staff will confirm eligibility and consent with prospective participants.

The webpage text, PICFs (electronic or hard copy) and eligibility questionnaire will have prior approval of HREC before use.

In Australia, influenza vaccination 72 hours prior to randomisation is an inclusion criterion as outlined in 4.3.2. For sites outside Australia, the research team will provide recommendation to all participants not to have the influenza vaccine within the 72 hours prior or post randomisation.

For those who are eligible and provide informed consent, they will be asked to provide their contact details (including date of birth, healthcare card number or equivalent (except for Australia), name and other identifying details) and a baseline questionnaire on participant characteristic (demographics and environmental information) into either REDCap database or hard-copy forms based on national privacy regulations.

Participants will be told when completing eligibility check (before consenting) that pregnancy, or planning to become pregnant within the next month is a contraindication to getting BCG. We will ask that if they are unsure to do a home pregnancy test, and on the day of randomisation we will have pregnancy tests available that they can use to take away to self-test at the site before randomisation. In the UK and Spain completing a pregnancy test will be an eligibility requirement as outlined in Appendix 3 and 6. This will be done in a subtle way to limit the likelihood that other staff will be aware that they have requested a pregnancy test. We have structured it this way to allow people to test in the privacy of their homes rather than have a conversation with the researchers.

Because only 10,078 participants are to be recruited over multiple sites, it is possible that more staff will be interested in participating than can be included in the trial. Given there will likely be interested participants who complete e-consent (where relevant) but are not randomised (become sick, become ineligible, changed their mind) we will continue recruitment until we reach the required number of participants randomised (10,078 participants). Randomisation will cease on the day that 10,078 participants are randomised. On the consent form and other pre-information, interested participants will be informed that due to the limited numbers who can be included in the trial, despite consenting, we cannot guarantee they will be randomised.

Given the importance of finding an intervention that can be used early in future pandemics (before a disease-specific vaccine is available), we expect there will be significant interest from researchers to try and understand how BCG works to boost the immune system. To this end, we will include an optional consent for participants to indicate whether they are interested in being approached for other projects.

No identifying information will be provided to the hospital or recruiting sites regarding any staff who have consented to be part of the trial.

4.7 Pre-randomisation blood sample

To remain eligible for randomisation in BRACE a pre-randomisation blood sample must be provided. This blood sample will be taken at enrolment but can be taken up to 24 hours prior to randomisation. This sample cannot be taken after administration of the intervention or placebo.

4.8 Re-consent

As required, participants will be contacted through REDCap and sent appropriate and relevant information for re-consent. Re-consent materials will contain contact details for the study team so that participants can ask questions. Participants will be asked if they agree to the changes by signing the re-consent in either electronic or hard copy format depending on country specific ethics requirements. All participant information for re-consent will be approved by HREC prior to use.

5 INTERVENTION

5.1 Treatment arms

Intervention group: BCG vaccine
Comparator group: 0.9% Saline

5.2 Trial Intervention(s)

5.2.1 Description of trial investigational products

5.2.1.1 BCG vaccine SSI

Active substance and excipients	Freeze-dried powder:<br>Live attenuated bacteria of the type *Mycobacterium bovis* BCG (Bacillus Calmette-Guerin), Danish strain 1331<br>0.1 ml vaccine contains between 2 to 8 x 10^5 colony forming units.<br><br>Powder Excipient: Sodium glutamate<br><br>Solvent for resuspension:<br>magnesium sulphate heptahydrate, dipotassium phosphate, citric acid monohydrate, l-asparagine monohydrate, ferric ammonium citrate, glycerol 85%, and water for injections.
Trade or Generic name	BCG Vaccine SSI
Dosage form	Powder for Injection with solvent for resuspension
Route of administration	Intradermal

5.2.1.2 Placebo to match BCG vaccine SSI

Active substance and excipients	Sodium Chloride 0.9%.
Trade or Generic name	Sodium Chloride Injection BP or USP
Dosage form	Ampoule (10 mL)
Route of administration	Intradermal

5.2.2 Dosage

A single dose of BCG vaccine SSI or matched placebo will be given to all participants who are randomised. The adult dose is 0.1 mL (of BCG vaccine SSI or 0.9% NaCl) injected intradermally over the distal insertion of the deltoid muscle onto the humerus (approximately one third down the upper arm).

5.2.3 Dose modification

There are no allowable dose modifications

5.2.4 Storage and dispensing of BCG vaccine SSI

- Store between 2ºC - 8ºC
- Store in the original package in order to protect from light
- Do not freeze
- Do not use the vaccine after the expiry date which is stated on the carton as "EXP" and refers to the last day of the month listed
- Any unused vaccine at the end of the study, meaning vaccines unused after the last dosing of the last participant will be disposed of according to local regulations

Placebo – sodium chloride 0.9%

- Store less than 25ºC
- Do not use after the expiry date which is stated on the carton and ampoule as "EXP" and refers to the last day of the month listed
- Any unused sodium chloride 0.9% at the end of the study, unused ampoules after the last dosing of the last participant will be disposed of according to local regulations

5.2.5 Preparation

BCG Vaccine SSI

BCG Vaccine SSI consists of a powder and solvent for suspension for injection (2-8 x 10^5 CFU/0.1 mL dose).

Prior to reconstitution, the storage temperature of the BCG will be checked to ensure that the appropriate temperature has been maintained during storage, and transport (if applicable) unless the storage and transport has occurred in validated containers or conditions where the temperature is stable and the data readily available.

The rubber stopper must not be wiped with any antiseptic or detergent. In the eventuality of alcohol being used to swab the rubber stopper, it must be allowed to evaporate before the stopper is penetrated with the syringe needle. The BCG is re-suspended using the solvent provided according to the product directions then carefully inverted a few times to produce uniform resuspension of the lyophilised BCG. Study staff must not shake the vial. The study staff member who re-suspends the BCG will label the vial with the date, time of reconstitution and their initials.

To ensure a uniform suspension, and therefore dose, the vial will be gently swirled before drawing up each dose. When drawn up into the syringe the reconstituted vaccine should appear homogeneous, slightly opaque and colourless.

Each vial of BCG contains up to 10 adult doses. Study staff must NEVER administer the whole vial. Each vial can be kept for up to 4 hours after resuspension. During this time the vial is kept between 2-8°C. Each vial is discarded after 4 hours, or when the vial is empty, whichever occurs first.

Sodium Chloride 0.9% placebo

During each recruitment session sodium chloride 0.9% will be decanted using aseptic technique into an empty sterile amber glass vial or prepared in 0.1 mL dosing syringes as per local vaccination practices. The study staff member who prepares the sodium chloride for injection will record the date, time of the preparation and their initials.

The prepared sodium chloride for placebo can be kept for up to 24 hours. During this time the placebo is kept between 2-25°C. All prepared syringes or vials unused at the end of a vaccination session will be discarded.

5.2.6 Administration of trial drug

The vaccine or placebo will only be administered by clinician members of the study team trained in the intradermal vaccination technique.

The vaccinator will follow the vaccination SOP and relevant site safety requirements.

Administration of the BCG vaccine or placebo will take place in locations set-up by the study team prioritising participant safety for example ensuring appropriate facilities for management of any potential adverse event are available (e.g anaphylactic reaction, extremely rare). There will be space to allow for privacy for the participant if required (e.g. upper left/right arm not accessible due to clothing).

As per standard practice, participants will be required to remain at the site for 20 minutes after vaccination, in case an allergic reaction should occur, wearing a sticker “I have received the BCG vaccine at [time of vaccination]” for both BCG and placebo recipients.

The time and date of resuspension of the vaccine vial, or placebo preparation, batch identifier, immunisation date/time, any issues with immunisation will be entered in the participants’ study record in REDCap Vaccinators database.

Route/method of administration

The injection site should be clean and dry using non-alcohol based antiseptic. Alcohol antiseptics should not be used prior to administration. If alcohol is used to swab the skin, it must be allowed to evaporate before the vaccine or placebo is injected. The vaccine or placebo must be given strictly intradermally, approximately one third down the upper arm corresponding to the area of the distal insertion of the deltoid muscle, as follows:

- The skin is stretched between thumb and forefinger
- The needle should be almost parallel with the skin surface and slowly inserted (bevel upwards), approximately 2 mm into the superficial layers of the dermis. The needle should be visible through the epidermis during insertion

- The vaccine or placebo should be given slowly

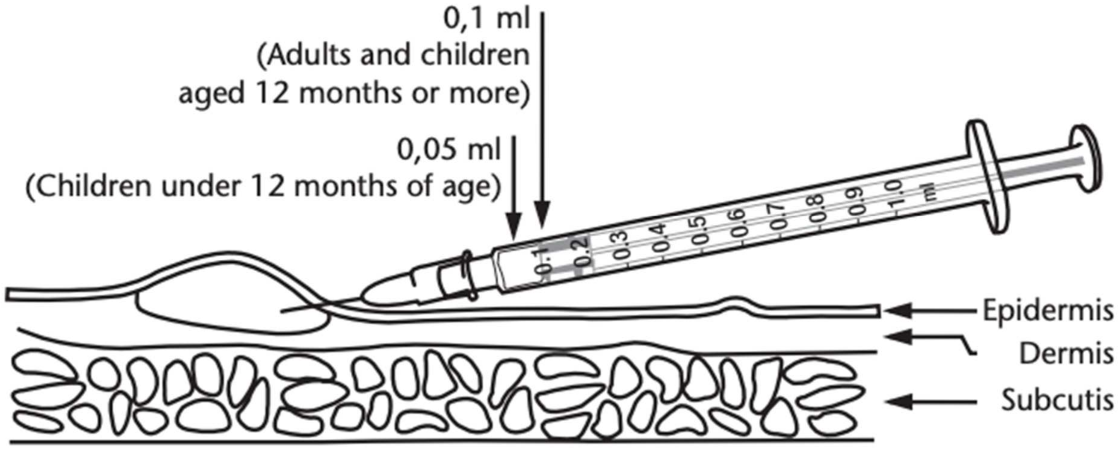

- The mixed vaccine should be administered with a syringe of 1 ml graduated into hundredths of millilitre (1/100) fitted with a short bevel syringe needle (Preference to use 25G or 26G, accepted up to 30G).
- You should feel considerable resistance as you give the injection. If there is no resistance, the needle may be in the subcutaneous tissues.
- If the injection is not intradermal, withdraw the needle and repeat at a new site.
- A raised, blanched papule/bleb of about 7 mm diameter (looks like orange peel) at the needle point is a sign of correct injection
- The injection site is best left uncovered to facilitate healing
- Jet injectors or multiple puncture devices should not be used to administer the vaccine.
- A photo of the bleb (with measuring tape or 10 cent coin used as scale (or equivalent in local currency)) should be uploaded in REDCap form.

Over/under dosage or incorrect administration

Overdose increases the risk of suppurative lymphadenitis and may lead to excessive scar formation. Gross over dosage increases the risk of undesirable BCG complications. Deep injections increase the risk of lymphadenitis and abscess formation.

The clinician members of the research team who administers BCG or placebo as part of this trial will be required to document whether the vaccination was given ‘perfectly’ with appropriate bleb. Any variations will be documented, and standard procedures followed regarding the need for re-administration, notification to RPI (or delegate).

Complications

All BCG-related complications will be referred to the SPI for advice regarding management. In the very unlikely event a participant has a systemic infection of *Mycobacterium bovis* or persistent local infection following vaccination the SPI will provide advice to the local treating team regarding management, including antibiotic treatment choice. Any serious adverse event or adverse event of interest occurring during the administration of the IP or the 20 minutes post administration will be documented appropriately according to the safety monitoring and reporting section of this protocol.

5.2.7 Product accountability

A pharmacy in each region will act as the study central pharmacy and coordinate the storage, distribution and maintain accountability records of the BCG vaccine and placebo supply in that region as appropriate. The RCH pharmacy will act as the study central pharmacy for Australia. The UMC Utrecht pharmacy will be the study central pharmacy in mainland Europe. A UK based pharmacy will act as the central study pharmacy should any UK sites be included in the trial. The LAC/UFMS will act as the central pharmacy in Mato Grosso do Sul, Brazil and a central pharmacy in Rio De Janeiro and Manus will be managed through the collaborating institution. Trial accountability of IP including documentation of storage, dispensation and destruction (if required) will be maintained in the pharmacy files at each region/site as appropriate. A pharmacy summary/manual will outline the specific processes for each region in line with local processes and regulations.

Any reason for departure from the expected dispensing regimen will be recorded. At the end of the trial, there will be final reconciliation of trial drug received, dispensed, used and returned. Any discrepancies will be investigated, resolved and documented by the study team.

5.2.8 Excluded medications and treatments

BCG vaccination may be given on the same day of any inactivated or live vaccines. If not given on the same day a period of not less than 4 weeks must pass before giving another live vaccine (although there is no real data supporting this precaution). There must be an interval of at least 3 months before a vaccination in the same arm can take place. Inactivated vaccine (such as the diphtheria-tetanus-pertussis vaccine) can be given in the other arm at any time before, during, or after BCG vaccination if needed.

Participants should not take part in any other COVID-19 preventative intervention clinical trials during the 6 month follow-up period.

5.2.9 Discontinuation from trial intervention

The trial intervention is a once-off vaccination. Due to this there is no possibility to ‘discontinue the trial intervention’. If a participant changes their mind between randomisation and vaccination, deciding that they do not want to have the vaccination (but are happy to continue in the study for the follow-up period) they will be included in the analysis as intention to treat.

6 RANDOMISATION AND BLINDING

Once consent has been obtained, and following baseline assessment, eligible participants will be recruited and randomised on the day of the enrolment via REDCap. Randomisation will be to intervention or placebo group with an allocation ratio of 1:1, using a web-based randomisation procedure. The randomisation schedule and web-based service will be provided by an independent statistician from the Clinical Epidemiology and Biostatistics Unit (CEBU) at the Murdoch Children’s Research Institute. Randomisation will be in randomly permuted blocks of variable length (2, 4, or 6). Randomisation will be stratified by stage of the study (prior to or post the addition of the placebo vaccination), study site, by age (<40 years; 40 to 59 years; >=60 years) and by presence of comorbidity (any of diabetes, chronic respiratory disease, cardiac condition, hypertension). Stratification by age is necessary for data analysis because

older ages are associated with a greater likelihood of developing severe COVID-19. Likewise, presence of comorbidity is associated with a greater risk of developing severe COVID-19. Each study site will have their own randomisation list stratified by study stage (where relevant), age and presence of comorbidity.

6.1 Concealment mechanism

The control group will receive a placebo of 0.9% NaCl. Most people vaccinated with the BCG vaccine develop a papule/blister at the injection site around two-weeks after vaccination. Due to this, even using a placebo, it is not completely possible to blind participants to their treatment group allocation. The outcomes (symptomatic and severe COVID-19 or admission to hospital for COVID-19) are objective measures, it is however still plausible that participant's suspicion of their group allocation might bias the study results. This risk will be mitigated by using a placebo where an element of doubt over treatment allocation may persist even in the absence of scar formation. Members of the study team, except immunisers, will be blinded to the group allocation (by the removal of this variable and all other variables related to BCG from the dataset) until the formal detailed statistical analysis plan is confirmed and signed by all investigators and all data cleaning/preparation is complete.

7 TRIAL VISITS AND PROCEDURES

7.1 TRIAL TIMELINE

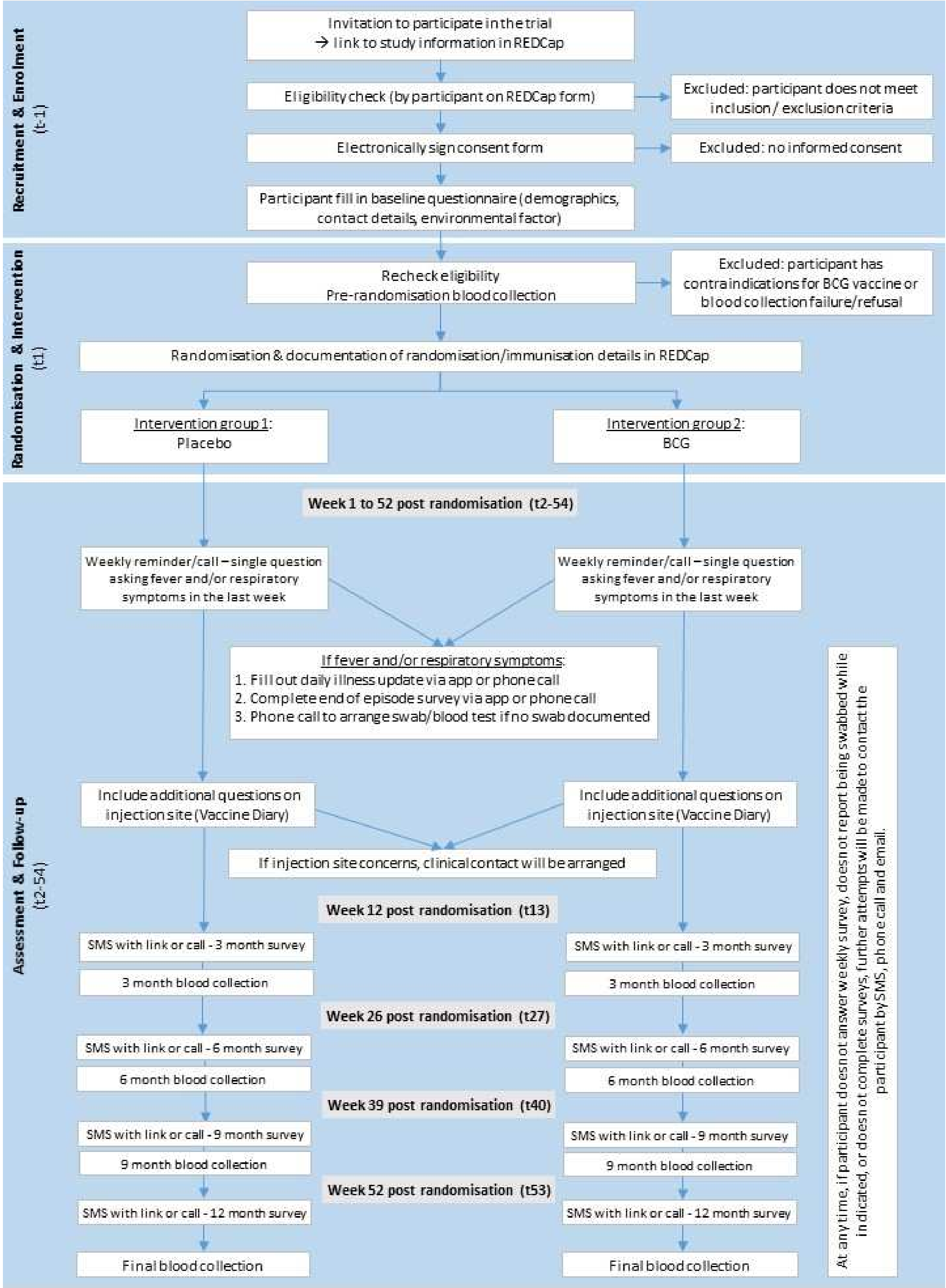

7.2 Schedule of assessments

	TRIAL PERIOD									
	Pre-study	Inclusion & randomisation	Post-randomisation							
TIME POINT	t_{-1}	t_0	t_{1-12}	t_{13}	t_{14-25}	t_{26}	t_{27-38}	t_{39}	t_{40-51}	t_{52}
RECRUITMENT:										
Eligibility screen	X									
Informed consent	X									
Contact details	X									
Allocation to intervention		X								
INTERVENTIONS:										
BCG vaccine		X (BCG group)								
Saline injection		X (Placebo group)								
ASSESSMENTS:										
Baseline questionnaire	X	X								
Weekly survey			X	X	X	X	X	X	X	X
Instruction for swab testing *(if indicated by weekly survey)*			(X)	(X)	(X)	(X)	(X)	(X)	(X)	(X)
3-month survey				X						
6-month survey						X				
9-month survey								X		
12-month survey										X
*Clinical advice on injection site **			X	X						
*Blood collection***		X		X		X		X#		X#
*Baseline SARS-CoV-2 Test ****		X								

T=week (e.g. t1=first week). A 42day window period is accepted for the periodic survey and the blood collection timepoints
* In indicated Infectious Diseases clinician, or state-based organisation, as appropriate
** Optional consent for additional biological sample including blood sample when illness reported
*** Brazil only as outlined in Appendix 4
# Sub-set of participants

7.3 Description of procedures

The procedures related to recruitment, consent, eligibility confirmation, randomisation and intervention are described in sections 4 and 5 of this protocol. The procedure for blood collection is described in the relevant SOP. Capture of applicable adverse events is described in section 8.

In Brazil only, a baseline respiratory swab will be collected as outlined in appendix 4.

After randomisation there are two key aspects of the 1-year follow-up period; questionnaires and sample collections for SARS-CoV-2 identification (respiratory swabs or blood samples). Participants will be asked to complete a questionnaire using the smartphone application (app) designed for the trial, electronic messages or via phone calls to report symptoms, access SARS-CoV-2 testing through the public health system and if needed self-collect a respiratory swab each time they have a febrile illness or a respiratory symptom. Where app is utilised, participants will be trained on how to use the app on day of enrolment.

<u>Questionnaires</u>

Baseline

- Comorbidities: diabetes, cardiovascular disease, chronic respiratory disease, hypertension
- Risk factors: smoking, body mass index (calculated with weight and height)
- BCG/TB history: Prior BCG vaccination, ever positive TST
- Influenza immunisation: date of last influenza vaccine (if within influenza season)
- Exposure (presence of COVID-19 cases at workplace, average days working in hospital or other healthcare settings as appropriate)
- Other: Recurrent herpes infection (such as cold sores)

Regular (generally weekly) questionnaires on smartphone app, via phone call or via electronic messages

- Any symptoms of COVID-19: fever or at least one sign or symptom of respiratory disease such as sore throat, cough, shortness of breath, respiratory distress/failure (y/n)

For each episode of illness (via smartphone app, via phone call or via electronic messages)

- Which symptoms of COVID-19: fever, cough, shortness of breath and/or difficulty breathing, runny/blocked nose, sore throat, fatigue, muscle and/or joint ache, headache, nausea, vomiting and/or diarrhoea, loss of taste and smell
- Has a COVID-19 swab been taken? (if so what was the result)
- Date of/days since onset and cessation of symptoms
- Days absent from work (total number and number due to illness)
- ED presentations
- Hospital admission (oxygen, ICU admission, mechanical ventilation)
- Known test results
- If a swab has been taken for clinical purposes, who ordered it
- Impact on daily activities
- Days in bed
- Chest x-ray results

For local reaction to injection: the Vaccine Diary (daily diary for the two weeks following injection)

- Questionnaire collecting common reactions to the injection, including photograph of injection site

Periodic questionnaires (once every 3 months)

- Exposure (presence of COVID-19 cases at workplace, average days working in hospital or other healthcare settings as appropriate)
- Cold sore recurrence
- Request for participants to confirm the main episodes of illness experienced in the prior 3 months.
- Screening, exposure or treatment of TB

- Detail on other vaccinations
- Hospitalisation (any)
- Information regarding participation in any other COVID-19 preventative intervention clinical trials
- Injection site evolution and side effects (photo of injection site)
- Treatment that could influence COVID-19 outcome

Additional questions for 3rd month questionnaire only:

- Record any non-serious adverse event of interest, including Injection site evolution and side effects (photo of injection site), with onset between randomisation and 3 months post randomisation
- If relevant: Influenza vaccine side effects
- Record any serious adverse event with onset between randomisation and 3 months post randomisation

<u>Swabs</u>

- Where a participant has had a swab sample assessed outside the indication of the study (e.g. with non-respiratory symptoms or asymptomatic) results will be collected via self-report. All test results will also be obtained, where possible, from centralised SARS-CoV-2 testing government database.

Where a participant has symptoms of febrile or respiratory illness (cough, sore throat, shortness of breath) and a swab sample for SARS-CoV-2 is not collected through standard pathways (for example due to swab shortage, or government decision to restrict screening to high-risk patients), a sample collection study visit may be done. A respiratory swab/s may be collected from the participant at home and linked with the relevant public health testing and reporting systems. If respiratory swab/s are done by participant self-collection (e.g. nasal/throat swabs) they will receive full instructions on how to take the samples, when to take them and how to correctly store them until a member of the research team collects them.

<u>Blood samples</u>

- At randomisation, a blood sample will be taken for later assessment of seroconversion (production of specific anti-SARS-CoV-2 antibodies). This will identify participants who had SARS-CoV-2 exposure and immunity prior to commencement of the study.
    - The baseline blood samples will be analysed in batch months after randomisation, so there will be no clinically actionable results. We will provide individualised results to participants via email after completion of the trial. This email will be sent to the applicable HRECs to review before being sent out to any participant.
    - In Brazil, IGRA testing will be completed on pre-randomisation and 12-month blood samples as outlined in appendix 4.
- At 3 months and 6 months (+ 42 days) post randomisation, the study team will coordinate to collect study blood samples from participants and in a subset of participants at 9 and 12 months (+ 42 days) post randomisation. This will identify

participants who had an immune response to SARS-CoV-2 (surrogate marker of infection) during the study. This is needed to determine asymptomatic SARS-CoV-2 infections.

- Blood samples will also be taken for assessment of the immune system. This will be used to meet the planned exploratory analyses related to vaccine induced changes in the immune system. These blood samples will be taken at the same time as blood collection for serum or plasma samples (i.e. at randomisation and post randomisation)
- In the eventuality that it is unfeasible to collect swab samples to confirm SARS-CoV-2 infection at the time of febrile or respiratory illness episodes (or conduct a validated RAT test), seroconversion may be used to associate episodes of febrile or respiratory illness with SARS-CoV-2 infection. Therefore, for episodes of febrile or respiratory illness where a swab sample cannot be taken, 1 month after the onset of symptoms (expected peak post-infection antibody production), participants may be asked to come to the hospital to provide a blood sample. If rapid point-of care testing is available, these tests may be distributed to participants to self-test. Should these alternative methods of testing become required, an amendment will be submitted to HRECs to outline the process and submit any information for participants. This testing will not be conducted without further consultation with and approval from the HRECs, including providing the HRECs with details of the test and its efficacy.

For blood sample collection for serum/plasma plus analysis of the immune system, a venous blood sample (up to 10ml or up to 35ml depending on the study site) will be taken by a trained member of the study team and labelled with participant ID, date/time collected, study timepoint, year of birth (no identifying information). Samples will be transported to the site's designated laboratory for the trial. Samples will be processed for serum/plasma separation and analysis of the immune system and stored at -80˚C or in liquid nitrogen for later assessment.

A self-collected dried blood spot may be requested from participants instead of a venous blood sample collection. These may be stored in a locked cabinet prior to elution and storage at -80˚C. If blood samples are done by participant self-collection of dried blood spot, participants will receive full instructions on how to take the samples, when to take them and how to correctly store them until they are returned to the study site.

<u>Data Retrieval</u>
Data retrieval is further described in Section 9 of this Protocol.

The present study expects that it will acquire some research data from existing administrative and service data sources. In Victoria, for example, this would include obtaining details from the Victorian Department of Health and Human Services (VDHHS) who collects information about presentations to hospitals and emergency departments for medical care in Victoria. Similar processes will be followed in other Australian states. In mainland Europe and Brazil, participants will be required to consent to provide access to their medical records by study staff. In the UK self –reports from participants may be supplemented by tracking of participants using their NHS number or other relevant unique identifiers (provided by

participant), drawing on Hospital Episode Statistics and Office of National Statistics data to track health service use (admissions) and deaths.

7.4 Notes on specific trial visits

7.4.1 Unscheduled visit

If participants have any concerns related to side effects or the injection site evolution or scarring, they can call or email the study team for advice and if necessary, they will be seen by a clinician member of the study team or delegate. Reassurance, appropriate management or referral for medical care will be done according to best practice. Documentation of adverse event will be done as indicated in section 8.

7.5 Procedure discontinuation, participant withdrawals and losses to follow up

7.5.1 Discontinuation of blood collection - participant remains in trial for follow up

Participants that decline further blood collection may still continue in all other aspects of the study.

7.5.2 Withdrawal of consent - participant withdraws from all trial participation

Participants are free to withdraw from the trial at any time upon their request. Withdrawing from the trial will not affect their access to standard treatment or their employment as their participation will not be shared with their employer.

For the safety of all participants withdrawing from the trial, reasonable efforts should be made to undertake protocol-specified safety evaluations.

A dedicated Case Report Form (CRF) page will be used to capture the date of participant withdrawal of consent, and the reason if offered.

7.5.3 Losses to follow-up

Due to the study taking place with healthcare workers during a pandemic, we expect that there may be periods that participants will ignore the smartphone app prompts, phone calls or electronic messages. This includes the eventuality that a participant has been admitted to hospital. The weekly smartphone app prompts will only ask whether the participant has had a fever or respiratory symptom since the last time they answered in the app (date provided). Alternatively, where appropriate, phone follow-up will be used (i.e., Brazil). We deem this very unlikely to annoy participants excessively as they can ignore the notification or call if they are too busy (or withdraw). This will give the project the best chance of having a complete dataset to analyse as they can answer 'Yes' when they get the opportunity and fill in the associated questionnaire. Therefore, we will continue to send out weekly notifications or calls for the entire study regardless of whether participants respond.

In Australia and Europe, if a participant does not answer 2 regular smartphone app prompts (2 consecutive weeks), further attempts will be made to contact them by electronic messages (maximal 3 attempts), phone call (maximal 3 attempts) and email (maximal 3 attempts). If there is still no response, and the participant is not found to have died on medical records, we will try to contact them later (when the workload is expected to have decreased).

In Brazil, if a participant does not answer 3 follow-ups phone contacts (phone call or electronic messages), a home visit may be carried out by study staff. If there is still no response, and the participant is not found to have died on medical records, we will try to contact them later (when the workload is expected to have decreased).

Where secondary contact provided, the study team will follow-up if unable to contact participants.

7.5.4 Replacements

Participants who have been randomised may NOT be replaced.

7.5.5 Trial Completion

A participant is considered to have completed the trial if he or she has completed all processes of the trial including the last visit or the last scheduled procedure shown in the Schedule of Assessments.

The end of the trial is defined as completion of the last visit or procedure shown in the Schedule of Assessments in the trial at all sites. At the end of the trial, the Sponsor-Investigator will ensure that all HRECs as well as all regulatory and funding bodies have been notified, if required.

This trial may be temporarily suspended or prematurely terminated if there is sufficient reasonable cause. If the trial is prematurely terminated or suspended, the Sponsor and Investigators will promptly inform trial participants, HRECs, the funding (where applicable) and regulatory bodies, providing the reason(s) for the termination or suspension.

Circumstances that may warrant termination or suspension include, but are not limited to:

- Determination of an unexpected, significant, or unacceptable risk to participants that meets the definition of a Significant Safety Issue (for the definition refer to Section 8.1).
- Insufficient compliance to protocol requirements
- Data that are not sufficiently complete and/or evaluable
- Demonstration of efficacy that would warrant stopping
- Determination that the primary endpoint has been met
- Determination of futility

In the case of concerns about safety, protocol compliance or data quality, the trial may resume once the concerns have been addressed to the satisfaction of the sponsor, HRECs, funding and/or regulatory bodies.

7.5.6 Continuation of therapy

As the treatment is ‘once-off’ there is no provision for continuation of therapy.

8 SAFETY MONITORING AND REPORTING

8.1 Definitions

Adverse Event (AE):

An AE is any untoward medical occurrence in a participant administered an investigational product and does not necessarily have a causal relationship with the study treatment. For this study, only certain adverse events are recorded, specifically serious adverse events as defined below, and non-serious adverse events of interest specified in section 8.2

Serious Adverse Event (SAE) :

Any serious adverse event (SAE) is an untoward medical occurrence that:

- Results in death; or
- Is life-threatening; or
- Requires hospitalisation or prolongation of existing hospitalisation;
    - Hospitalisation is to be considered an SAE only in the event of an overnight admission. Any elective hospitalisation does not constitute an SAE
- Results in persistent or significant disability/incapacity; or
- Is a congenital anomaly/birth defect

Note: Life-threatening refers to an event in which the participant was at risk of death at the time of the event. It does not refer to an event that hypothetically might have caused death if it were more severe.

Medical and scientific judgement should be exercised in deciding whether an adverse event should be classified as serious in other situations. Important medical events that are not immediately life-threatening or do not result in death or hospitalisation but may jeopardise the participant or may require intervention to prevent one of the other outcomes listed in this definition should also be considered serious.

For this study, all SAE will be collected for the period from randomisation to 3 months post randomisation.

Suspected Unexpected Serious Adverse Reaction (SUSAR):

A SUSAR is an AE that meets all of the following criteria:

- The AE is serious (as defined above; an SAE); and
- The SAE is suspected adverse reaction to the investigational product, meaning it is judged by either the reporting investigator or the Sponsor as having a reasonable possibility of a causal relationship to a study vaccine (possibly, probably or definitely related), and
- The SAE is also unexpected: An unexpected serious adverse reaction is one for which the nature or severity of the reaction is not consistent with reference safety information (Which is comprised of the BCG vaccine Product Information and the *WHO information sheet: Observed rate of vaccine reactions Bacille Calmette Guerin Vaccine April 2012*).

Note that an event is instead considered 'expected' if it is listed in the Reference Safety Information and therefore cannot meet the definition of SUSAR.

Significant Safety Issue:
A significant safety issue is an issue that could adversely affect the safety of participants or materially impact on the continued ethical acceptability or conduct of the trial.
Comment: A significant safety issue is a new safety issue or validated signal considered by the Sponsor in relation to the study vaccines that requires urgent attention of stakeholders. This may be because of the seriousness and potential impact on the benefit-risk balance of the study vaccines, which could prompt regulatory action and/or changes to the overall conduct of the clinical trial, including the monitoring of safety and/or the administration of the study vaccines.

Urgent Safety Measure (USM):
A measure required to be taken in order to eliminate an immediate hazard to a participant's health or safety. Note: This is a type of significant safety issue that can be instigated by either the investigator or Sponsor and can be implemented before seeking approval from HRECs or institutions.

8.2 Capturing and eliciting adverse event information

For the period of randomisation to 3 months post randomisation only, the following are non-serious adverse events of interest for this study:

- At injection site:
    - Reaction (pain, tenderness, redness, swelling) of grade 3 (severe) or 4 (potentially life threatening)
    - Abscess
    - Large ulcer (>1.5 cm diameter)
    - Keloid scar
    - Unusual local reaction
- Lymphadenopathy (in region of injection site)
- BCG osteitis/osteomyelitis
- Disseminated BCG infection (BCG-osis)
- Allergic reaction due to IP
- Fainting episode, seizures and convulsions following IP administration (recorded on the day of IP administration only)

Only these non-serious AE and all SAE, occurring between randomisation and 3 months post randomisation, will be recorded for this study. If applicable, for the remainder of the follow-up period, sites may additionally document participants' AE as required to meet reporting requirements of the applicable HREC/s and/or regulatory authority.

8.2.1 SAE capture

SAE are captured on the day of IP administration, as recorded by the site personnel. Information on any SAE since randomisation will be solicited from participants at the 3-month questionnaire. SAE may also be captured via participant notification, in the period

between randomisation and the 3-month questionnaire, such as through spontaneous contact by the participant via call or email, data entered in the Vaccine Diary or the study smartphone app (or equivalent, e.g. weekly phone calls). In cases where a participant does not respond to multiple attempts at contact, over several weeks, the participant's secondary contact will be contacted to confirm their status and record fatal SAE if applicable.

For this study, all SAE will be collected for the period from randomisation to 3 months post randomisation.

8.2.2 Non-Serious AE Capture

Non-serious AE of interest are captured:

- On the day of IP administration, recorded by the site personnel
- Within the Vaccine diary (which triggers an alert to the site personnel to contact the participant)
- Through the 3-month questionnaire (questions on injection site evolution)
- Through spontaneous contact (e.g. phone call, electronic message or email) from the participant to the site team.

8.3 Documentation of AEs

For the purposes of this study the investigator or delegate is responsible for recording the applicable Adverse Events, regardless of their relationship to study vaccines.

The documentation of each applicable AE on the REDCap CRF will include:

- A description of the AE
- The onset date, duration, date of resolution
- Severity
- Seriousness (SAE or not)
- Any action taken (e.g. treatment, follow-up tests)
- The outcome (recovery, death, continuing)
- The likelihood of the relationship of the AE to the trial treatment

All AEs will be followed to resolution or stabilisation, where possible.

8.4 Assessing the relatedness (causality) of a participant's AE

All non-serious AE of interest and SAE must have their relationship to the trial intervention assessed by the SPI (or delegate) who evaluates the AE based on temporal relationship and their clinical judgment. The degree of certainty about causality will be graded using the categories below.

The relationship of the event to the trial intervention will be assessed as follows:

Code	Causal Relationship	Description
1	Unrelated	The AE is clearly NOT related to the intervention
2	Unlikely	The AE is doubtfully related to the intervention
3	Possible	The AE may be related to the intervention
4	Probable	The AE is likely related to the intervention
5	Definite	The AE is clearly related to the intervention

8.5 Assessing the severity of a participant's AE

The SPI (or delegate) will be responsible for assessing the severity of an AE. The determination of severity for all AE should be made by the investigator based upon medical judgment and the severity categories of Grade 1 to 5 as defined in the first table below, with the following exceptions: injection site pain, redness, tenderness and swelling/induration are assigned severity grades using the specific toxicity grade specified in the second table below.

Grade	Severity	Description
Grade 1	Mild	Asymptomatic or mild symptoms; clinical or diagnostic observations only; intervention not indicated
Grade 2	Moderate	Moderate; minimal, local or non-invasive intervention indicated; limiting age-appropriate instrumental activities of daily living (ADL)
Grade 3	Severe	Severe or medically significant but not immediately life-threatening; hospitalisation or prolongation of hospitalisation indicated; disabling; limiting self-care ADL
Grade 4	Life Threatening	Life-threatening consequences; urgent intervention indicated
Grade 5	Fatal	Death related to AE

Toxicity grading scale
Local reaction to vaccination are monitored using Vaccine diary completed by the participant up to 14 days after vaccination. A toxicity grading scale is used to categorise the reports (Food and Drug Administration 2007):

**Local reaction**	**Grade 0** None	**Grade 1** Mild	**Grade 2** Moderate	**Grade 3** Severe	**Grade 4** Potentially life threatening
**Pain**	None	Does not interfere with activity	Repeated use of nonnarcotic pain reliever > 24 hours or interferes with activity	Any use of narcotic pain reliever or prevents daily activity	Emergency room visit or hospitalization
**Redness**	None	2.5 - 5 cm	5.1 - 10 cm	>10 cm	Necrosis or exfoliative dermatitis
**Tenderness**	None	Mild discomfort to touch	Discomfort with movement	Significant discomfort at rest	Emergency room visit or hospitalization
**Swelling / induration**	None	2.5 - 5 cm and does not interfere with activity	5.1 - 10 cm or interferes with activity	>10 cm or prevents daily activity	Necrosis
**Itch**	None	Itching localised to injection site that is relieved spontaneously or in <48 hours of treatment	Itching beyond the injection site that is not generalised OR Itching localised to injection site requiring ≥48 hours of treatment	Generalised itching causing inability to perform usual social & functional activities	Not applicable

Food and Drug Administration. (2007). "Guidance for Industry: toxicity grading scale for healthy adult and adolescent volunteers enrolled in preventive vaccine clinical"
Retrieved 08.04.2020, from https://www.fda.gov/downloads/BiologicsBloodVaccines/GuidanceComplianceRegulatoryInformation/Guidances/Vaccines/ucm091977.pdf.

8.6 Reporting of safety events

Site Principal Investigator Reporting Procedures:
The SPI (or delegate) is responsible for expedited reporting (within 24 hours of becoming aware of the event) to the Sponsor the following:

- USMs
- All SAEs (including SUSAR)

SAE reports should be submitted using the REDCap SAE form, or by alternative means specified in the Safety Reporting Plan.

At MCRI, the CPI (or delegate) will determine whether or not each SAE meets the definition of SUSAR and will notify all RPI in a timely manner.

The RPI and SPI will be notified of USM and other significant safety issues in a timely manner following MCRI first knowledge of the event/s.

In each country, USM, other significant safety issues, SUSARs and other SAE, will be reported to the applicable regulatory authorities and HRECs in accordance with the requirements.

Further details of event reporting responsibilities and processes are documented in the Safety Reporting Plan.
For safety reporting requirements specific to Brazil, refer to Appendix 4.

9 DATA AND INFORMATION MANAGEMENT

9.1 Overview

The Site Principal Investigator is responsible for storing essential trial documents relevant to data management and maintaining a site-specific record of the location(s) of the site's data management-related Essential Documents.

The Site Principal Investigator is responsible for maintaining adequate and accurate files of any relevant source documents that include observations or other data relating to participants at their site. Source data will be attributable, legible (including any changes or corrections), contemporaneous, original, accurate, complete, consistent, enduring and available. Changes to source data (hardcopy and electronic) must be traceable, must not obscure the original entry, and must be explained where this is necessary.

The Site Principal Investigator will also maintain accurate case report forms (CRFs) (i.e. the data collection forms) where applicable and be responsible for ensuring that the collected and reported data is accurate, legible, complete, entered in a timely manner and enduring. To maintain the integrity of the data, any changes to data (hardcopy and electronic) must be traceable, must not obscure the original entry, and must be explained where this is necessary.

Any person delegated to collect data, perform data entry or sign for data completeness will be recorded on the delegation log and will be trained to perform these trial-related duties and functions.

9.2 Data management

9.2.1 Data generation (source data)

In this study, the following types of data will be collected:

- personal identifying information (names, dates of birth, contact details; NHS number in UK, SUS CARD and CPF in Brazil)
- sensitive information including health data (medical history, participant eligibility, adverse reactions and other notes as appropriate)
- participant completed electronic questionnaires
- de-identified data from laboratory assays

Source document plan

Much of the data for this trial will be collected electronically directly from participants. There will be a limited number of source documents for this study; recorded data from automated instruments, laboratory reports and the signed information and consent forms (in REDCap or hard copy). Each site participating in the trial will maintain a site-specific Source Document Plan that will document the source, i.e. original recording, for each data discrete item/ category of items collected for the study. This Source Document Plan, signed and dated by

the Site Principal Investigator, will be prepared prior to recruitment of the first participant and will be filed in the site's Investigator Site File.

9.2.2 Data capture methods and data use, storage, access and disclosure during the trial

Data collection methods

Data for this trial will be collected and entered using electronic database REDCap and a smartphone application developed for this trial. REDCap is a secure, web-based application for building and managing online surveys and databases. The trial smartphone application stores participant information directly in the REDCap database. In line with local privacy regulations, identifying or personal data may be maintained in complementary site level information management systems as required.

Use of the data

The data will be used for the analyses specified in the protocol and Statistical Analysis Plan.

Following the completion and analysis of the trial, the data will be retained long-term following the mandatory archive period for use in future research projects.

Storage and access

Hard copy data will be stored by collaborators in a locked cabinet in a secure location, accessible to the research team only.

Electronic data maintained on REDCap database will be securely stored in MCRI's ’network file servers, which are backed up nightly. Electronic or hard copy files containing private or confidential data will be stored only in locations accessible only by appropriate designated members of the research team.

REDCap is hosted on MCRI infrastructure and is subject to the same security and backup regimen as other systems (e.g. the network file servers). Data is backed up nightly to a local backup server, with a monthly backup taken to tape and stored offsite. REDCap maintains an audit trail of data create/update/delete events that is accessible to project users who are granted permission to view it. Access to REDCap will be provided via an MCRI user account or (for external collaborators) via a REDCap user account created by the MCRI system administrator. The permissions granted to each user within each REDCap project will be controlled by, and will be the responsibility of, the study team delegated this task by the Principal Investigator. REDCap has functionality that makes adding and removing users and managing user permissions straightforward. All data transmissions between users and the REDCap server are encrypted. The instructions for data entry to REDCap must be read and the training log signed prior to personnel commencing data entry on REDCap.

Authorised representatives of the sponsoring institution as well as representatives from the HREC, Research Governance Office and regulatory agencies may inspect all documents and records required to be maintained by the CPI for the participants in this trial. The trial site will permit access to such records.

Disclosure

The trial protocol, documentation, data and all other information generated will be held in

strict confidence. No information concerning the study or the data will be released to any unauthorised third party, without prior written approval of MCRI. Clinical information will not be released without written permission of the participant, except as necessary for monitoring by the HREC, Research Governance Office or regulatory agencies.

9.2.3 Data confidentiality

Data confidentiality

Participant confidentiality is strictly held in trust by the CPI, participating investigators, research staff, and the MCRI and their agents. This confidentiality is extended to cover testing of biological samples in addition to the clinical information relating to participating participants.

To preserve confidentiality and reduce the risk of identification during collection, analysis and storage of data and information, the following will be undertaken:

(1) The number of private/confidential variables collected for each individual has been minimised. The data collected will be limited to that required to address the primary and secondary objectives.

(2) Participant data and samples will be identified through use of a unique participant study number assigned to the study participant ("re-identifiable").

The CPI is responsible for the storage in REDCap of a master-file of identifiable data with the participant ID; access is managed by restricting user permissions to members of the research team and authorised persons.

(3) Separation of the roles responsible for management of identifiers and those responsible for analysing content. The data will be analysed by members of the research team who will be provided with anonymised data identified only by the unique participant study ID.

9.2.4 Quality assurance

A REDCap data dictionary with range checks will be used to minimise data entry errors, such as out-of-range values. Data quality control checks (e.g. checking for invalid characters, invalid dates, data that are not consistent with data in other data fields) and data cleaning will be done by trained members of the research team on a regular basis. Any discrepancies will be reported to the CPI or delegate and addressed in a timely manner.

Quality control checks will be run by the data team, on a regular basis, who will highlight any queries to the CPI, RPI and SPI.

9.2.5 Archiving - Data and document retention

Upon completion of the study, data will be stored securely on MCRI server (restricted access) and/or locked in secure cabinet in MCRI laboratories (for hardcopy data) for at least 15 years after study completion, in accordance with the requirements of the Therapeutic Goods Administration and Health Privacy Principles and any other relevant regulatory authorities.

Prof Nigel Curtis (CPI) will be the custodian during the archive period, and members of the research team will have access to the stored data. If the CPI becomes unable to perform this

task all responsibilities of the custodian will fall to the sponsor (MCRI). At the end of the archival period, long-term retention of the data may occur.

Records should not be destroyed without the written consent of the Sponsor. The Sponsor will inform Site Principal Investigators when these documents no longer need to be retained.

9.2.6 Data sharing

Data sharing- Bill and Melinda Gates foundation requirements

Under the terms of the funding agreement with the Bill and Melinda Gates foundation, the BRACE trial has a data sharing agreement in place. Further information about the requirement can be found here: https://openaccess.gatesfoundation.org/how-to-comply/data-sharing-requirements/. The key actions undertaken are:

- Registration of the BRACE trial with clinicaltrials.gov and provided all required information (https://clinicaltrials.gov/ct2/show/NCT04327206)
- Executed an agreement and created an account with Vivli, a Clinical Trials Data Sharing Repository (https://search.vivli.org/studyDetails/5090da81-0953-464f-9e46-3260697d4f22)

An anonymised Individual Participant Data (IPD) dataset and a data dictionary will be provided to Vivli (https://vivli.org/) under the terms of the agreements with the Bill and Melinda Gates foundation grant and Vivli.

Participant consent to the data sharing requirements is a mandatory requirement in updated Master PICF v8. Participants consented under earlier versions (prior to v7) of the PICF will be advised about the updated data sharing requirements and given an opportunity to opt-out via email of the data sharing arrangement. Data will not be shared, where participants have specifically requested their data not be shared.

After database lock, a 12-month embargo period will be in place, to allow adequate time for analyses and publication outputs. Data transfer to Vivli should occur during the embargo period.

After database lock, the following may be made available long-term for use by future researchers from a recognised research institution whose proposed use of the data has been ethically reviewed and approved by an independent committee and who accept MCRI's conditions, under a collaborator agreement, for accessing:

- Individual participant data that underlie the results reported in our articles after de-identification (text, tables, figures and appendices)
- Study protocol, Statistical Analysis Plan, PICF

9.2.7 Long-term custodianship (after archive period finished)

Prof Nigel Curtis will be the long-term custodian following the archive period. If he is unable to perform this task the responsibility of custodianship will fall to the sponsor (MCRI).

9.2.8 Data retrieval

The present study expects that it will acquire some research data from existing administrative and service. Participant consent may allow retrieval of datasets as has usually been the case of other MCRI studies.

In Brazil, this data can be retrieved, if necessary, from the national government information systems, such as E-SUS, SIVEP-GRIPE, GAL and electronic medical records from SESAU / CG / MS (Municipal Health Secretariat of Campo Grande / MS, through unique identifiers of the participant (registration in the Individual Taxpayer Register - CPF and SUS CARD).

We anticipate data access will occur after the study recruitment period is complete, but the exact timing is yet to be determined.

9.2.9 Sample management: Additional data management considerations

Data and information for biospecimens will be managed as above with the additional considerations.
Data collection: de-identified sample data may also be stored in OpenSpecimen (restricted access stored on secured servers at each study laboratory site), other site-specific electronic laboratory information management systems (LIMS, restricted access) or hard copy . Where biospecimen data are stored in OpenSpecimen or site-specific LIMS, data will be transferred to the MCRI servers on a regular basis. Where biospecimen data is collected on hardcopy, data will be transcribed to REDCap on a regular basis.

9.2.10 Sample management: Specimen collection & storage.

Biospecimens will be processed, stored and data will be recorded at laboratory study sites. Samples will be identified using barcoded tubes or with the unique participant study ID, and year of birth. No identifying information will be stored on biospecimen labels. Biosamples will be stored securely at laboratory study sites in temperature-controlled freezers and liquid nitrogen tanks as appropriate for the sample type. Access to biosamples will be restricted to the study team. The samples will be used for the analyses specified in the protocol. Samples will be shipped from study sites to MCRI for long term storage. For tests that require equipment or technical expertise not available in Melbourne, select specimens may be sent to collaborating laboratories outside of Melbourne (interstate and/or overseas) for further testing. These samples may be shipped from MCRI or directly from study sites if they have not yet been shipped to MCRI. Shipment of samples to MCRI or collaborating laboratories doing testing will be done by International Air Transport Association (IATA) accredited staff with temperature control (e.g. ice pack, dry ice) as appropriate for the sample type.

The biosamples will be retained long-term according to the banking management detailed below. As per data, Prof Nigel Curtis (CPI) will be the custodian of the biosamples during the archive period.

9.2.11 Sample management: Specimen & Biobanking

All samples that are not used immediately for the laboratory assessments described in previous sections, may be cryopreserved for an indefinite period of time to enhance the possible benefit from this study, by providing a sample biobank that may be used for research related to immunology or infectious diseases, in the future. The biobank will be at MCRI laboratories

(Infectious Diseases Group) in Melbourne, (please see Appendix 1 for Biobank Registration Form). The biobank will be registered with the Melbourne Children's Bioresource Centre (MCBC). Written informed permission (extended consent) for banking of specimens and future use for study objectives without further consent will be obtained from the participant. These samples may be used for additional research studies related to immunology or infectious diseases. For tests that require equipment or technical expertise not available in Melbourne, select specimens may be sent to collaborating laboratories outside of Melbourne (interstate and/or overseas) for further testing.

Databank is defined as: "A systematic collection of data, whether individually identifiable, re-identifiable or non-identifiable" (NHMRC National Statement on Ethical Conduct in Human Research)

Biobank is defined as: ".... collections of human biological materials (biospecimens) linked to relevant personal and health information (which may include health records, family history, lifestyle and genetic information) and held specifically for use in health and medical research." (NHMRC Biobanks Information Paper 2010)

10 TRIAL OVERSIGHT

10.1 Governance structure

10.1.1 Trial Steering Committee (TSC)

The trial steering committee will be made up of representatives from the key stakeholders and the chief principal investigator along with independent content expert(s).

10.1.2 Independent Data and Safety Monitoring Board (DSMB)

An independent Data and Safety Monitoring Board (DSMB) will be convened three times during the study: at 3 and 9 months post initial recruitment, and once there have been 100 severe case of COVID-19.

The DSMB at 3 and 9 months will monitor safety (including number of deaths and number of ICU admissions), data completeness, and the general study conduct.
A third DSMB is planned once there have been 100 cases of severe COVID-19. This interim analysis will primarily be on a comparison of the number of cases of severe COVID-19 (primary outcome (2)) between the BCG group and the control group for participants recruited post the introduction of the placebo (second stage of the trial). The DSMB will be given a stopping rule, but since the pandemic is rapidly evolving, the global situation should be considered with the context of any apparent differences. More information of this efficacy interim analysis is explained in section 11.4 of this current Protocol and on the Statistical Analysis Plan for the interim analysis.

All the details of the DSMB analyses will be outlined in the DSMB charter.

The DSMB will be composed of individuals with the appropriate expertise, including at least three independent clinicians and/or biostatisticians who, collectively, have experience in the management of biostatistics and the conduct and monitoring of randomised controlled trials.

Members of the DSMB will be independent of trial conduct. The DSMB will review data from each intervention group of the trial in a semi-blinded fashion. The DSMB will provide its input to the CPI.

10.1.3 Independent Safety Monitor

During the start of the recruitment period until August 2020, an independent safety monitor will review a report of sae and specified non-serious adverse events of interest on a weekly basis and report any concerns to the sponsor-investigator. For the remainder of the recruitment period, the monitor will review such reports monthly until June 2021, after which the reports will be review every 3 monthly.

10.1.4 Quality control and quality assurance.

Both the Chief Principal Investigator and Site Investigators have responsibilities in relation to quality management.

The Chief Principal Investigator will ensure the development of procedures that identify, evaluate and control risk for all aspects of the study, e.g. study design, source data management, training, eligibility, informed consent and adverse event reporting. The Chief Principal Investigator will ensure the implementation of quality control (QC) procedures, which will include the data entry system and data QC checks. Any missing data or data anomalies will be communicated to the site(s) for clarification/resolution.

The Site Principal Investigator will be responsible to ensure the verification that the clinical trial is conducted and data are generated and biological specimens are collected, documented (recorded), and reported in compliance with the protocol, good clinical practice and applicable regulatory requirements. In some regions a subcontracted monitor may be engaged by MCRI as needed.

In the event of non-compliance that significantly affects human participant protection or reliability of results, the Chief Principal Investigator (or delegate) and/or Site Principal Investigator (or delegate) will perform a root cause analysis and corrective and preventative action plan (CAPA).

In addition, each clinical site will perform internal quality management of study conduct, data and biological specimen collection, documentation and completion. An individualised quality management plan will be developed to describe a site’s quality management.

11 STATISTICAL METHODS

11.1 Sample Size Estimation

7244 healthcare workers will be enrolled in the trial outlined in this protocol, although data from this trial will be combined with the data from the 2834 participants enrolled into the first stage of this study which followed an identical protocol but where participants were randomised between BCG and no BCG which was given concurrently with the influenza vaccination, resulting in a total sample size of 10078 participants.

Participants will be randomly allocated in a 1:1 ratio to BCG vaccine group (n=3622, plus 1417 from the first stage who received influenza vaccine at time of randomisation), and to control (n=3622, plus 1417 from the first stage who received influenza vaccine at time of randomisation and no 0.9% NaCl placebo). This sample size was calculated based on the two primary outcomes of (1) symptomatic COVID-19 and (2) severe COVID-19. Since the study aims to assess two primary outcomes, an adjustment for multiplicity will be applied to maintain a global Type I error rate of 5% by splitting of this alpha.

For the primary outcome (1), symptomatic COVID-19 at 6 months: it is conservatively estimated that a proportion of 55% of subjects will be infected by symptomatic COVID-19 in the placebo group; applying a 1:1 ratio for randomisation, a total sample size of n=2016 (1008 group) will provide 95% power with 2-tailed 0.005 significance level (10% of the global significance level) for the Pearson chi-square test (with continuity correction) to detect an absolute difference of 10% between an incidence of symptomatic COVID-19 of 45% in the BCG vaccine group and 55% in the placebo group.

For the primary outcome (2), severe COVID-19 at 6 months, we powered the study to identify a risk ratio of 0.67 in the BCG compared with the placebo group for severe COVID-19 at 6 months (which is much more realistic than a risk ratio of 0.5 as per the original sample size). Assuming that 4% of subjects will be infected by severe COVID-19 by 6 months in the control group, a total sample size of n= 6076 (3038 per group) will provide 80% power with 2-tailed 0.04 significance level (80% of the global significance level) for the Pearson chi-square test to detect a risk ratio of 0.667, equivalent to an absolute difference of 1.3%. Note this calculation was conducted using an alpha of 0.04 to allow the remaining 0.01 to be spent on primary outcome (1) (alpha=0.005) and the interim analysis as originally planned (alpha=0.005, see section 11.4 for details of the interim analysis, total alpha for this outcome=0.045). Allowing for a 16% loss to follow up, it is planned that the study will recruit 7244 healthcare workers.

In the pre-planned meta-analysis, we will have a sample size of 10,078 participants (7244+2834), or 8062 participants allowing for an overall 20% loss to follow up. For the combined analysis it is expected that the drop-out will be slightly higher (20% instead of 16%) because it also includes participants recruited prior to the introduction of the placebo, i.e. not placebo controlled. Again assuming that 4% of subjects will be infected by severe COVID-19 by 6 months in the control group, a total sample size of n=8062 (4031 per group) will provide 90% power with 2-tailed 0.04 significance level for the Pearson chi-square test to detect a risk ratio of 0.667, equivalent to an absolute difference of 1.3%. This will be a secondary analysis of the final study report.

The original trial plan included a meta-analysis on all the outcomes (both COVID-19 and non-COVID-19 related outcomes) combining data from Stage 1 and Stage 2 participants. Stage 1 healthcare workers were recruited only in Victoria and Western Australia, both of which Australian states had almost negligible COVID-19 exposure risk during that trial period (30th Mar 2020 to 13th May 2020). In light of this, the overwhelming majority of Stage 1 blood samples are likely to be seronegative. Moreover, with a low prevalence of COVID-19, there is a high probability that positive SARS-CoV-2 serology results are false positive. For these reasons, in December 2021 the BRACE team has agreed that it is not justifiable to devote extra resources (time and costs) to the data cleaning of potential COVID-19 episodes and SARS-CoV-2 serology for all participants in Stage 1. As a consequence, the meta-analysis will

only be run on non-COVID-19 related outcomes and its main objective will be to determine if BCG vaccination compared with control reduces the rate and severity of febrile or respiratory illness over 12 months following randomisation in Stage 1 and Stage 2 healthcare workers.

11.2 Population to be analysed

The primary analysis of all outcome data will be an intention-to-treat (ITT) analysis including all randomised participants, regardless of whether they received trial drug.

11.2.1 Handling of missing data

For the primary analysis the imputation of missing data will only be considered if 10-20% of the primary outcome is missing and will be undertaken using multiple imputation (MI) models. Multiple imputation analysis will be performed on the ITT population. The frequency and patterns of missing data will be examined. Multiple imputation models will be conducted separately in the two treatment groups using chained equations applied to all outcomes, including baseline measures, as auxiliary variables. Fifty imputed datasets will be generated including all randomised subjects.

11.3 Methods of analysis

Data analysis for the study will be performed by CEBU at MCRI. Ms Francesca Orsini has been appointed for the trial.

Statistical analysis will follow standard methods for randomised trials and the primary analysis will be by intention to treat (ITT), including all randomised participants.

Categorical variables will be presented as the number and proportion in each category. Continuous variables will be presented as means and standard deviations (SDs), or medians and interquartile ranges for skewed data, and the range.

PRIMARY ANALYSIS. Comparison between the BCG and placebo groups in the proportions of participants with symptomatic COVID-19 (primary outcome 1), as well as in the proportions of participants with severe COVID-19 (primary outcome 2), will be presented as the absolute risk difference (RD) as well as the risk ratio (RR) at 6 months and their 95% confidence interval (CI), obtained using a generalised linear model, with adjustment for the strata (defined by site, age and presence of comorbidity) used in the randomisation. The same analysis will be repeated on the same outcomes at 12 months. As secondary analyses the same models will be run to include also the following covariates: gender, number and type of comorbidities, whether already vaccinated for BCG in the past, and any other factor that may show imbalance between the groups at baseline.

A secondary analysis will be performed as above on the total 10078 participants, comparing all the participants who were randomised to BCG (irrespective of whether they received influenza vaccine at randomisation) and those randomised to control (irrespective of whether they received influenza vaccine or placebo at randomisation). Analysis will be as described above, but also adjusted for being in the initial stage of the study. As part of this analysis we will conduct an exploratory analysis of whether the treatment effect varies between the two stages of the study (prior to and post the introduction of the placebo) by including an

interaction between treatment and study stage. Results will be interpreted with caution given that the study is underpowered for this comparison.

SECONDARY OUTOMES. According to the nature of the secondary outcomes to be analysed (binary, continuous or categorical) the appropriate generalised linear model (GLM) will be used to estimate the effect of the BCG vaccine on the outcome of interest compared to the control group. All analyses will be adjusted for the stratification factors used in the randomisation (site, age and presence of comorbidity). As secondary analyses the same models will be run to include the following covariates: sex, number and type of comorbidities, whether already vaccinated for BCG in the past, and any other factor that may show imbalance between the groups at baseline.

Survival analysis techniques will be adopted to analyse time to event data.

A secondary analysis will be conducted on the total 10078 participants using the same methodology but also adjusted for being in the initial stage of the study. We will conduct an exploratory analysis of whether the treatment effect varies between the two stages of the study by including an interaction between treatment and study stage.

Sub-group analyses will be undertaken on outcomes of those who:

- Had previous BCG vaccine before enrolling into the trial
- Had a positive serology to SARS-CoV-2 when enrolling into the trial

The full details for each variable will be included in the Statistical Analysis Plan (SAP).

11.4 Interim Analyses

As part of the interim monitoring there will be a single formal interim analysis of the efficacy data. This interim analysis will be on a comparison of the number of cases of severe COVID-19 (primary outcome (2)) between the BGG group and the control group for those recruited post the introduction of the placebo (second stage of the study). The timing of the interim analysis will be event driven, and will be conducted using a time-to-event analysis, censoring participants who have not had the event at the time of their last follow-up. This data will be used to provide Kaplan-Meier estimates of the survival curve in the BCG and control groups, which will be used to estimate the proportion with severe COVID-19 at 6 months. These proportions will be used to compare the two groups.

The timing of the interim analysis will be determined by the original sample size calculation. Under the original sample size calculation, with 1668 per group and an incidence of 4% in severe COVID-19 at 6 months in the control group and 2% in the intervention group, this would equate to 67 + 33 = 100 cases in total. We therefore planned to conduct a formal interim analysis of severe COVID-19 once there had been 100 cases of severe COVID-19.

The stopping rule to be used in the interim analysis will be based on an alpha spending function, where the threshold to identify efficacy is based on the amount of data available at the time of the interim analysis relative to the data available at the end of the trial. The threshold for the interim analysis and the remaining alpha for the final analysis if the study is not stopped at the interim will be calculated using the Group Sequential Test (GST) of Two Proportions in NQuery (PTT12-1) with an alpha-spending function based on the Pocock stopping rule. This calculation will be based on an overall alpha of 0.045 for the primary

outcome (2), and the amount of available information at the time of the interim analysis (calculated as the percentage of participants with outcome data on severe COVID by 6 months relative to the final sample size).

This interim analysis of severe COVID-19 will be performed on all of the participants randomised into Stage 2 of the trial up to the interim analysis time point, comparing all the participants who are randomised to BCG and those randomised to placebo.

A statistical analysis plan for the interim analysis will be written, shared with the DSMB and made publicly available prior to undertaking the interim analysis. This analysis plan will provide all the details of the interim analysis, including the threshold to be adopted for the interim analysis.

Given the dynamic nature of research in this field, the DSMB will be advised that this rule be used as a guideline rather than a formal rule, and should be interpreted in the context of external information and information on the efficacy of BCG vaccination on the incidence of symptomatic COVID-19.

12 ETHICS AND DISSEMINATION

12.1 Research Ethics Approval & Local Governance Authorisation

This protocol and the informed consent document and any subsequent amendments will be reviewed and approved by the applicable human research ethics committee (HREC) prior to commencing the research at each site. A letter of protocol approval by HREC will be obtained prior to the commencement of the trial, as well as approval for other trial documents requiring HREC review.

12.2 Amendments to the protocol

This trial will be conducted in compliance with the current version of the protocol. Any change to the protocol document or Informed Consent Form that affects the scientific intent, trial design, participant safety, or may affect a participants willingness to continue participation in the trial is considered an amendment, and therefore will be written and filed as an amendment to this protocol and/or informed consent form. All such amendments will be submitted to the HREC, for approval prior to being implemented.

12.3 Protocol Deviations and Serious Breaches

All protocol deviations will be recorded in the participant record (source document) and on the CRF and must be reported to the CPI or delegate, who will assess for seriousness.

Those deviations deemed to affect to a significant degree rights of a trial participant or the reliability and robustness of the data generated in the clinical trial will be reported as serious breaches. Reporting will be done in a timely manner (the CPI or delegate to review and submit to the approving HRECs within 7 days, or as required).

Where non-compliance significantly affects human participant protection or reliability of results, a root cause analysis will be undertaken and a corrective and preventative action plan prepared.

Where protocol deviations or serious breaches identify protocol-related issues, the protocol will be reviewed and, where indicated, amended.

13 CONFIDENTIALITY

Participant confidentiality is strictly held in trust by the participating investigators, research staff, and the sponsoring institution and their agents. This confidentiality is extended to cover testing of biological samples in addition to the clinical information relating to participating participants.

The trial data and all other information generated will be held in strict confidence. No information concerning the trial or the data will be released to any unauthorised third party, without prior written approval of the sponsoring institution. Authorised representatives of the sponsoring institution may inspect all documents and records required to be maintained by the Investigator. The clinical trial sites will permit access to such records.

All laboratory specimens, evaluation forms, reports and other records that leave the site will be identified only by the Participant Identification Number (SID) to maintain participant confidentiality.

Clinical information will not be released without written permission of the participant, except as necessary for monitoring by HREC or regulatory agencies.

14 PARTICIPANT REIMBURSEMENT

In Australia and Europe, participants will not be reimbursed for their involvement.

As outlined in appendix 4 in Brazil in line with federal legislation, expenses resulting from participation in the study, such as transportation to the place where the vaccination will be carried out will be reimbursed. The amount will not be considered substantial and reimbursement system will be designed to reduce risk of reimbursement being considered compensation or inducement to participant in the trial.

15 FINANCIAL DISCLOSURE AND CONFLICTS OF INTEREST

This is an investigator-initiated study, and the funders will have no role in the study design, data collection and analysis, decision to publish, or preparation of the manuscript. MCRI holds no commercial interest in the manufacture and trade of BCG.

16 DISSEMINATION AND TRANSLATION PLAN

The results of the trial will be reported to the participants after analysis is complete. The results of this trial will be submitted to peer reviewed journals, presented at conferences and may form part of student theses.

The Chief Principal Investigator holds primary responsibility for publication of the results of the trial.

17 REFERENCES

1. Novakovic B, Messina N, Curtis N. Chapter 6 - The Heterologous Effects of Bacillus Calmette-Guérin (BCG) Vaccine and Trained Innate Immunity. In: Faustman DL, editor. The Value of BCG and TNF in Autoimmunity (Second Edition). Second edition. ed: Academic Press; 2018. p. 71-90.
2. World Health Organisation. Situation report - 197: World Health Organisation; 2020. Report No: 197.
3. Anderson RM, Heesterbeek H, Klinkenberg D, Hollingsworthd T. How will country-based mitigation measures influence the course of the COVID-19 epidemic? The Lancet. 2020.
4. SARS (Severe Acute Respiratory Syndrome). World Health Organisation, 2020. [https://www.who.int/ith/diseases/sars/en/, accessed 11/03/2020]
5. Middle East respiratory syndrome coronavirus (MERS-CoV). World Health Organisation, 2020. [https://www.who.int/en/news-room/fact-sheets/detail/middle-east-respiratory-syndrome-coronavirus-(mers-cov), accessed 11/03/2020]
6. World Health Organisation. Report of the WHO-China Joint Mission on Coronavirus Disease 2019 (COVID-19) World Health Organisation; 2020.
7. Elguero E, Simondon KB, Vaugelade J, Marra A, Simondon F. Non-specific effects of vaccination on child survival? A prospective study in Senegal. Tropical medicine & international health : TM & IH. 2005;**10**:956-60.
8. Higgins JP, Soares-Weiser K, Lopez-Lopez JA, et al. Association of BCG, DTP, and measles containing vaccines with childhood mortality: systematic review. BMJ. 2016;**355**:i5170.
9. Kristensen I, Aaby P, Jensen H. Routine vaccinations and child survival: follow up study in Guinea-Bissau, West Africa. BMJ (Clinical research ed). 2000;**321**:1435-8.
10. Nankabirwa V, Tumwine JK, Mugaba PM, Tylleskar T, Sommerfelt H. Child survival and BCG vaccination: a community based prospective cohort study in Uganda. BMC public health. 2015;**15**:175.
11. Vaugelade J, Pinchinat S, Guiella G, Elguero E, Simondon F. Non-specific effects of vaccination on child survival: prospective cohort study in Burkina Faso. BMJ (Clinical research ed). 2004;**329**:1309.
12. Hollm-Delgado MG, Stuart EA, Black RE. Acute lower respiratory infection among Bacille Calmette-Guerin (BCG)-vaccinated children. Pediatrics. 2014;**133**:e73-81.
13. de Castro MJ, Pardo-Seco J, Martinon-Torres F. Nonspecific (Heterologous) Protection of Neonatal BCG Vaccination Against Hospitalization Due to Respiratory Infection and Sepsis. Clin Infect Dis. 2015;**60**:1611-9.
14. Arts RJW, Moorlag S, Novakovic B, et al. BCG Vaccination Protects against Experimental Viral Infection in Humans through the Induction of Cytokines Associated with Trained Immunity. Cell Host Microbe. 2018;**23**:89-100 e5.
15. Nemes E, Geldenhuys H, Rozot V, et al. Prevention of M. tuberculosis Infection with H4:IC31 Vaccine or BCG Revaccination. N Engl J Med. 2018;**379**:138-49.
16. Wardhana, Datau EA, Sultana A, Mandang VV, Jim E. The efficacy of Bacillus Calmette-Guerin vaccinations for the prevention of acute upper respiratory tract infection in the elderly. Acta Med Indones. 2011;**43**:185-90.
17. Moorlag S, Arts RJW, van Crevel R, Netea MG. Non-specific effects of BCG vaccine on viral infections. Clin Microbiol Infect. 2019;**25**:1473-8.
18. Freyne B, Marchant A, Curtis N. BCG-associated heterologous immunity, a historical perspective: intervention studies in animal models of infectious diseases. Trans R Soc Trop Med Hyg. 2015;**109**:287.
19. Kleinnijenhuis J, Quintin J, Preijers F, et al. Bacille Calmette-Guerin induces NOD2-dependent nonspecific protection from reinfection via epigenetic reprogramming of monocytes. Proc Natl Acad Sci U S A. 2012;**109**:17537-42.

20. Kleinnijenhuis J, Quintin J, Preijers F, et al. BCG-induced trained immunity in NK cells: Role for non-specific protection to infection. Clin Immunol. 2014;**155**:213-9.
21. Kleinnijenhuis J, Quintin J, Preijers F, et al. Long-lasting effects of BCG vaccination on both heterologous Th1/Th17 responses and innate trained immunity. J Innate Immun. 2014;**6**:152-8.
22. Messina NL, Zimmermann P, Curtis N. The impact of vaccines on heterologous adaptive immunity. Clin Microbiol Infect. 2019;**25**:1484-93.
23. Zimmermann P, Donath S, Perrett KP, et al. The influence of neonatal Bacille Calmette-Guerin (BCG) immunisation on heterologous vaccine responses in infants. Vaccine. 2019;**37**:3735-44.
24. Zykov MP, Subbotina TI. Modulation of humoral immune response to influenza vaccines by BCG. Acta Virol. 1985;**29**:403-9.
25. Australian Technical Advisory Group on Immunisation (ATAGI). Australian Immunisation Handbook. Canberra: Australian Government Department of Health,; 2018.
26. Australian Government Department of Health. The BCG vaccine: information and recommendations for use in Australia March 2013.
27. Lotte A, Wasz-Hockert O, Poisson N, Dumitrescu N, Verron M, Couvet E. BCG complications. Estimates of the risks among vaccinated subjects and statistical analysis of their main characteristics. Adv Tuberc Res. 1984;**21**:107-93.
28. Bothamley GH, Cooper E, Shingadia D, Mellanby A. Tuberculin testing before BCG vaccination. BMJ (Clinical research ed). 2003;**327**:243-4.
29. Hendry AJ, Dey A, Beard FH, Khandaker G, Hill R, Macartney KK. Adverse events following immunisation with bacille Calmette-Guerin vaccination: baseline data to inform monitoring in Australia following introduction of new unregistered BCG vaccine. Communicable diseases intelligence quarterly report. 2016;**40**:E470-e4.
30. BCG Vaccine SSI data sheet 14 March 2018. Ltd SN, [https://www.medsafe.govt.nz/profs/datasheet/b/BCGCSLinj.pdf, accessed 24 Dec 2019]
31. Pilgrim S. Administration of the Bacillus Calmette Guerin (BCG) Vaccination in neonates. 2019.
32. Vaccine schedules in all countries of the European Union. European Centre for Disease Prevention and Control, 2020. [https://vaccine-schedule.ecdc.europa.eu/, accessed Apr 2020]
33. de Vries G, Riesmeijer R. National Tuberculosis Control Plan 2016-2020. Bilthoven BA, National Institute for Public Health and the Environment, 2016. [Available at https://www.rivm.nl/documenten/national-tb-control-plan-0, accessed March 2020]
34. World Health Organization. Bulletin of the World Health Organization 1995: World Health Organization; 1995 1995.
35. Fine P, Carneiro I, Milstien J, Clements JC. Issues relating to the use of BCG in immunization programmes: a discussion document. Geneva: Department of Vaccines and Biologicals, World Health Organization; 1999.
36. Paul Fine IC, Julie Milstien, C. John Clements. Issues relating to the use of BCG in immunization programmes: a discussion document. Geneva: Department of Vaccines and Biologicals, World Health Organization; 1999.
37. Immunisation schedules in the WHO European Region: World Health Organization; 1995.
38. Coulter C. Tuberculosis testing. Aust Fam Physician. 2012;**41**:489-92.
39. Randomised controlled trial of single BCG, repeated BCG, or combined BCG and killed Mycobacterium leprae vaccine for prevention of leprosy and tuberculosis in Malawi. Karonga Prevention Trial Group. Lancet. 1996;**348**:17-24.
40. Roth AE, Benn CS, Ravn H, et al. Effect of revaccination with BCG in early childhood on mortality: randomised trial in Guinea-Bissau. BMJ (Clinical research ed). 2010;**340**:c671.
41. Rakshit S, Ahmed A, Adiga V, et al. BCG revaccination boosts adaptive polyfunctional Th1/Th17 and innate effectors in IGRA+ and IGRA- Indian adults. JCI Insight. 2019;**4**.

42. Hatherill M, Geldenhuys H, Pienaar B, et al. Safety and reactogenicity of BCG revaccination with isoniazid pretreatment in TST positive adults. Vaccine. 2014;**32**:3982-8.
43. Rodrigues LC, Pereira SM, Cunha SS, et al. Effect of BCG revaccination on incidence of tuberculosis in school-aged children in Brazil: the BCG-REVAC cluster-randomised trial. Lancet. 2005;**366**:1290-5.
44. Cunha AJ, Sant'Anna CC, Mannarino R, Labanca TC, Ferreira S, March MF. Adverse effects of BCG revaccination: a report on 13 cases from Rio de Janeiro, Brazil. Int J Tuberc Lung Dis. 2002;**6**:1110-3.
45. Faustman DL. Type 1 Diabetes Reversal Trials at Massachusetts General Hospital. In: Hospital MG, editor. Massachusetts General Hospital: Massachusetts General Hospital; 2018.

17.1 Appendix 1: Specimens for biobanking - completed biobank registration form

Document version & date	Version 1.1 24th Aug 2020
Name of the bank	BCG vaccine to prevent severe COVID-19 in healthcare workers (BRACE)
Custodian of the bank	Name: Prof Nigel Curtis
Purpose of the bank	To store data and samples collected in the ':BCG vaccination to Reduce the impact of COVID-19 in healthcare workers (BRACE)' trial so they can be used in future research related to infectious diseases and immunity.
Sample/data type(s) and where these will be accessed from and over what time period	<u>Data will be collected from</u><br>Questionnaires and test results obtained as part of the research project 'BCG vaccination to Reduce the impact of COVID-19 in healthcare workers (BRACE)', by members of the research team.<br>Blood and/or swab samples will be obtained via this research project and stored for an indefinite period of time.<br>The samples/data may be sent overseas for future research related to infectious diseases, immunology, or vaccines.<br><u>Data stored includes:</u><br>- Demographics (e.g. age, gender, date)<br>- Environment (e.g. household members, exposure to SARS-CoV-2 positive people, role in the hospital, TB exposure, previous vaccinations)<br>- Study outcome related data (e.g. SARS-CoV-2 test results, BCG and influenza vaccine reactions, illnesses during study period, data generated from the laboratory analysis of samples collected)<br><u>Sample types stored:</u><br>- Swabs<br>- Plasma<br>- Serum<br>- Peripheral blood samples<br>- Granulocytes and whole blood<br>- Nucleic acid<br>After data ceases to be collected directly from participants, data may be obtained/generated via access to their medical records, government data sets or as samples are analysed and the data are added back into the data/biobank.

Sample/data identifiability	Clinical data in 'BCG vaccination to Reduce the impact of COVID-19 in healthcare workers (BRACE)' will be collected and stored in a REDCap database; a secure password-encrypted online database, or similar electronic database hosted by MCRI.<br><br>Data will be stored in re-identifiable format with the key held by the custodian or delegate. The REDCap database or comparable database will be hosted on the secure Murdoch Children's Research Institute (MCRI) server and backed up regularly by MCRI Information Technology.<br><br>Only members of the research team involved in data collection or data management will have access to the project's REDCap database or similar electronic database.<br><br>Samples will be stored (frozen) in re-identifiable format by using study ID number or tube barcodes.<br><br>All data associated with sample storage location and tracking will be stored in a separate REDCap database or similar electronic database. Access to this database is limited to members of the research team working in data/sample management or sample processing.<br><br>Laboratory generated data, any data collected outside of REDCap and data exported from REDCap or similar electronic database, will be stored in re-identifiable format by study ID. The data will be stored on the MCRI server in restricted folders on the Infectious Diseases group drive, as per MCRI policy.<br><br>Samples/data stored in re-identifiable format can be linked by the custodian or delegate to participants' identifiable information if it is ethically appropriate and required.
Criteria for Bank participants	Consenting to the project includes allowing the participants' data and samples to be used as defined in the protocol.<br><br>In addition there is an optional consent in the PICF for the storage of participants' biospecimens and participants' re-identifiable data for use in future research related to infectious diseases and immunity.<br><br>Inclusion criteria for Bank participants<br><br>- Recruited participant in the research project 'BCG vaccination to Reduce the impact of COVID-19 in healthcare workers (BRACE))'<br><br>- Provided informed consent for their data and samples to be stored for future ethically approved research (extended consent) related to infectious diseases and immunity.

Access process for obtaining the samples/data	Researchers must discuss their research plan with a member of the research team of the project 'BCG vaccination to Reduce the impact of COVID-19 in healthcare workers: (BRACE))'. The following will be taking into consideration:<br>- Scientifically justifiable hypothesis and aims<br>- Study design is appropriate to achieve study aims<br>- Inclusion/exclusion criteria for participants appropriate to answer question<br>- If the research proposal is deemed to have merit, the researcher will complete a REDCap (or similar electronic database) access form detailing the proposed design, participants, data +/- samples that they would like access to.<br>This will be reviewed by the custodian (or delegate) of the data who will need to take into account the following, before approval is granted:<br>- Does the research plan involve research in the area of immunology or infectious diseases? If not, it is outside the scope of the data/biobank. To use the data one of the following will be required:<br>o a new project approved by the RCH HREC and participants contacted for their consent<br>o a new project approved by the RCH HREC and a waiver of consent granted<br>- Is the planned analysis feasible with the data/samples available in the data/biobank?<br>- Are there competing interests for the sample/data type in question?<br>- Is another researcher already analysing the data in a similar way and would collaboration on the existing project be more appropriate?<br>The access form for access to the data/biobank will be kept on the REDCap database or similar electronic database.
Sample and data input	Members of the research team working in data/sample management will input the data and samples to the data/biobank.
Location of the Bank	Samples will be stored in the MCRI freezer farm or in the Infectious Disease Group's freezers, and may be distributed to other collaborating laboratories where they may also be stored.

	Data will be stored in a REDCap online database or similar electronic database, hosted on the secure Murdoch Children's Research Institute (MCRI) server, as well as in restricted electronic folders on the MCRI Infection and Immunity group drive.
Confidentiality/security of samples/data	Members of the research team of the project 'BCG vaccination to Reduce the impact of COVID-19 in healthcare workers (BRACE)' involved in data/sample collection or management will have open access to the bank data/samples.<br>No identifying data will be provided to researchers using data/samples from the biobank. To re-identify data/samples, the custodian (or delegate) will have access to the key, but will not pass this information onto researchers unless approved by ethics, or as required by law.<br>Data stored on REDCap database or similar electronic database will be password protected, and hosted on the secure MCRI server. This is backed up regularly by MCRI Information Technology.<br>The Bank will be secure against unauthorised access and passwords will be changed at regular intervals (as per MCRI policy).<br>The custodian (or delegate) will ensure removal of access to data once a project is finished or a researcher leaves the project.
Destruction of samples/data	Destruction of samples/data will occur upon participant request. This will be managed by the custodian (or delegate).
Modifications to Bank Protocol	If a change of purpose/data type/type of samples is to be considered, the custodian (or delegate) is required to submit to the HREC for approval and either contact the participants to obtain consent, or a waiver must have been granted.

17.2 Appendix 2. Collection of stool samples from a subset of BRACE participants

Background and Rationale:

For reasons that are poorly understood, B and T cell responses to vaccination (including BCG vaccination) are highly variable between individuals and between different populations. While many host factors, such as genetics, can influence inter-individual variation in these responses, increasing evidence shows that the gut microbiota, a large and diverse group of microorganisms that colonise gastrointestinal tract (GIT), plays a key role in shaping immune responses to vaccination (reviewed Lynn & Pulendran, 2017). For instance, in human infants, the relative abundance of several bacterial species in the stool microbiota has been associated with vaccine-specific IgG and T cell proliferation responses (Huda et al., 2014). Similarly, the composition of the stool microbiota in infants from rural Ghana was correlated with responses to the oral rotavirus vaccine (Harris *et al.,* 2017). Interestingly, germ-free mice have also been found to have impaired antibody responses to immunization with the model antigen ovalbumin (Lamousé-Smith *et al.,* 2011) and to the non-adjuvanted influenza vaccine (Oh *et al.,* 2014). Moreover, one of the principal investigators involved in this trial has recently found that, in mice, dysregulation of the microbiota leads to significantly impaired B and T cells responses to five different adjuvanted and live vaccines (including BCG) that are routinely administered to infants worldwide (Lynn *et al.,* 2018). Restoring the commensal microbiota rescued impaired responses (Lynn *et al.,* 2018). These data strongly suggest that the composition of the gut microbiota plays an important role in specific immune responses to vaccination. Whether the gut microbiota also influences non-specific effects of vaccines is currently unknown.

Primary objective of exploratory sub-study:

In a subset of BRACE trial participants consenting for an optional stool sample collection at baseline, determine whether the composition or metagenome-encoded function of the stool microbiota is correlated with either specific or non-specific immune responses to the BCG vaccine.

Secondary objectives of exploratory sub-study:

- Assess whether the composition of the stool microbiota is associated with any of the other primary, secondary, or exploratory outcomes described in the study protocol.
- Characterise the composition of the stool microbiota in participants in the trial and investigate whether the composition of the microbiota is altered at 3 or 12 months later.
- Assess whether immunisation with BCG leads to an altered microbiota at 3 or 12 months compared to participants receiving the placebo.

Outcomes:
Microbiota composition, including identities and the relative abundance of the bacteria present and their encoded microbial genes.

Population:
BRACE trial participants consenting for an optional stool sample collection at baseline, 3 months and 12 months.

Study Duration:
As per the BRACE trial protocol – 2 years.

Participant Duration:
12 months from randomisation.

Sub-study Locations:
Optional inclusion for Australian sites.

Sub-study Principal Investigator:
Prof. David J. Lynn BA MSc PhD
EMBL Australia Group Leader, Precision Medicine Theme, South Australian Health & Medical Research Institute, Adelaide, SA 5001.
Professor, College of Medicine & Public Health, Flinders University, Bedford Park, South Australia.
Email: david.lynn@sahmri.com

Potential risks and benefits:
Known potential risks:
This sub-study involves minimal risk to participants. Appropriate collection containers will be provided to participants to facilitate stool sample collection, storage and transport. A small stool sample will be collected by the participants at home. The tube contains a reagent that stabilises DNA at room temperature for up to 14 days. Participants will return the sample via a pre-paid addressed envelope. There will be no financial cost to the participant.

Known potential benefits:
This sub-study is exploratory in nature and is not expected to provide any direct additional benefit to the participants. The findings of the study, however, may be of significant value for understanding the role of the microbiota in influencing responses to vaccination.

Sub-study design:
Consent:
An additional option has been added to the BRACE online consent form to allow participants to optionally consent for a stool sample collection at baseline, 3 months and 12 months. The BRACE participant information and consent form (PICF) has been also modified to explain to participants the process for collecting stool samples and why they are being collected. If a participant declines to consent for stool sample collection this will not affect their participation in the BRACE trial (assuming all other inclusion and exclusion criteria are met).
Sample collection process:
Participants consenting for a stool sample collection will be provided with a collection pack at existing study visits at baseline, 3 months, and 12 months. The provided pack will contain: Instruction sheet, gloves, pathology stool pot, stool specimen collector tube and spoon set, protective plastic carrying tube, specimen bag, labels for identification of samples and pre-paid addressed envelope (for return postage). Participants will take the collection pack home with them and follow the following instructions to collect and return the stool sample.

Collection instructions:

1. Wash hands thorough and apply gloves.

2. Collect stool sample into the pathology stool pot within 1-3 days of study appointment.
   Note: Method of collecting the stool sample must prevent stool from falling into toilet water to avoid sample contamination.

3. Unscrew the stool specimen collector tube cap and use the spoon to scoop two spoonsful of stool (approximately 2 gram or 2mL in volume) from the sample.

4. Place the sample in the stool specimen collector tube.

5. Tighten the cap and shake to mix the contents thoroughly (invert 10 times) to create a suspension. Note: Some stool material may be difficult to re-suspend. As long as the material is suspended, the sample is stabilized. Foaming/ frothing during shaking is normal.

6. Dispose of gloves, unused stool material and the pathology stool pot and wash hands thoroughly.

7. Place stool specimen collector tube into the protective plastic carrying tube.

8. Place carrying tube into specimen carrier bag.

9. Place the sealed specimen bag containing the sample into the provided postage-paid reply envelop and post within 7 days of sample collection.

10. Samples will be returned to the nearest BRACE site laboratory for storage at -80C.

What we will do with the sample:
Briefly, samples will be collected at home by the study participants into Zymo fecal collection tubes which contain a reagent to stabilise DNA at ambient temperature. Samples were returned by mail within 2 weeks and stored at -80°C until processed. DNA will be extracted from pelleted samples using the appropriate DNA Isolation kit. We will perform 16S rRNA sequencing and/or metagenomic sequencing to profile the composition of the microbiota in the sample and the metagenome encoded by the microbiota. qPCR will be utilised to quantify bacterial load and quantify specific bacterial populations. We will then determine whether the composition or metagenome-encoded function of the stool microbiota is correlated with either specific or non-specific immune responses to the BCG vaccine. We will also assess whether the composition of the stool microbiota is associated with any of the other primary, secondary, or exploratory outcomes described in the study protocol. Furthermore, we will characterise the composition of the stool microbiota in participants in the trial and investigate whether the composition of the microbiota is altered at 3 or 12 months later. We will assess whether immunisation with BCG leads to an altered microbiota at 3 or 12 months compared to participants receiving the placebo.

References:
Lynn, D.J. and B. Pulendran, *The potential of the microbiota to influence vaccine responses.* J Leukoc Biol, 2017. **103**(2): p. 225-23

Huda, M.N., et al., *Stool microbiota and vaccine responses of infants.* Pediatrics, 2014. **134**: p. e362-72.

Harris, V.C., et al., *The infant gut microbiome correlates significantly with rotavirus vaccine response in rural Ghana.* J Infect Dis, 2017. **215**(1): p. 34-41.

Lynn, M.A., et al., *Early-Life Antibiotic-Driven Dysbiosis Leads to Dysregulated Vaccine Immune Responses in Mice.* Cell Host Microbe, 2018. **23**(5): p. 653-660 e5.

Oh, J.Z., *et al., TLR5- Mediated Sensing of Gut Microbiota Is Necessary for Antibody Responses to Seasonal Influenza Vaccination*. Immunity, 2014. ***41***: 478-492.

17.3 Appendix 3 UK Specific Requirements

In the UK, the Competent Authority (MHRA) required the following two UK specific requirements:

1. In the UK, a negative pregnancy test is required for all WOCBP to confirm eligibility for the trial.

2. In the UK, the responsibility to break the treatment code in emergency situations resides solely with the UK Principle Investigator and will not be delayed by requiring other study staff in Australia such as the Chief Investigator or medical monitor to be involved in the decision to un-blind. The study code will only be broken for valid medical or safety reasons e.g. in the case of a severe adverse event where it is necessary for a treating physician (Requester) to know which intervention the participant has received, in order to manage the participant's condition appropriately.

The Requester contacts the local Principal investigator (PI), or delegate, to discuss the pros and cons of breaking the code. If the consensus is to break the code, the Requester contacts the holder of the code break list. In the UK, this has been delegated to the UK based Data Manager who will provide the Requester with the information on allocated group on direction from the PI. On receipt of the allocation details the Requester deals with the participant's medical emergency as appropriate. Should this code-breaking protocol be activated, the Chief Investigator will be alerted at the earliest opportunity, and within 2 working days at the latest.

<u>Woman of Child Bearing Potential:</u>
For the purpose of this document, a woman is considered of childbearing potential (WOCBP), i.e. fertile, following menarche and until becoming post-menopausal unless permanently sterile. Permanent sterilisation methods include hysterectomy, bilateral salpingectomy and bilateral oophorectomy. A postmenopausal state is defined as no menses for 12 months without an alternative medical cause. A high follicle stimulating hormone (FSH) level in the postmenopausal range may be used to confirm a post-menopausal state in women not using hormonal contraception or hormonal replacement therapy. However in the absence of 12 months of amenorrhea, a single FSH measurement is insufficient.

17.4 Appendix 4 Brazil Specific Requirements

SARS-CoV-2 Screening test

Due to public interest in determining the extent of asymptomatic SARS-CoV-2 infection in healthcare workers in Brazil, the Brazilian investigators will use the BRACE participants to estimate this prevalence rate. Therefore after enrolment a baseline respiratory swab will be collected by the study nurse. The swab samples will be analysed by PCR for detection of SARS-CoV-2 and participants advised when results are confirmed. Participants who return a positive SARS-CoV-2 result on the baseline swab will remain in the trial. In Mato Grosso do Sul, the samples will be analysed in batch months after randomisation, so there will be no clinically actionable results. Results will be shared with participants approximately 3 months after randomisation, for participant who return a positive SARS-CoV-2 result, the site will be required to report the participant's positive SARS-CoV-2 results to the applicable health agencies. They will be told that they will not be informed of their result before then. In Rio de Janeiro, due to high transmission rates, samples will be tested immediately and reported to participants. PCR tests will be conducted by the study lab team and the results reported to health agencies by a system called e-SUS VS, which constitutes a database of several diseases, including COVID-19, which is mandatory.

IGRA

At randomisation, blood for anti-SARS-CoV-2 antibodies and IGRA will be taken for later assessment of seroconversion and TB infection. Therefore the initial blood sample in Brazil will be 35ml. This will identify participants who had TB exposure prior to commencement of the study. IGRA results will not exclude participants at consent & randomisation stage. Results will be shared with participants approximately 3 months after randomisation. A study doctor will follow-up with participants with positive IGRA to offer further assessment and treatment through government service provision.

At 12 months, blood for IGRA will be taken for later assessment to answer exploratory outcomes. Therefore the 12-month blood sample in Brazil will be 35 ml. This will identify participants who acquired TB during the study. Results will be shared with participants approximately 3 months after 12-month blood collection. A study doctor will follow-up with participants with positive IGRA to offer further assessment and treatment through government service provision.

Participant reimbursement

In Brazil, Resolution No. 466 of December 12, 2012 outlines the guidelines and regulatory standards for research involving humans in Brazil. This resolution outlines the requirement to provide reimbursement to participants and their companions, when necessary, such as transportation. In line with this requirement, participants in Brazil will receive reimbursement for relevant transportation costs for participation in the BRACE trial.

Safety Reporting

In Brazil, the RPI/s and SPI/s must comply with the safety reporting requirements of CEP/CONEP (defined in Circular Letter number 13). The HREC/s must be notified of all SAEs through the Brazil Platform (Notification), after the end of the event. The following timelines will be met for this study:

1. 30 days in case of fatal SAE occurring in a participant of the site in the jurisdiction of the HREC
2. 7 days in case of an SAE with a causal relationship with the investigational product, in a participant of the site in the jurisdiction of the HREC (Casual relationship means that the SAE is judged by either the reporting investigator or the sponsor as having a reasonable possibility of a causal relationship to a study vaccine).
3. 6 months for other SAE.

The RPI (or delegate) will notify SUSAR in Brazil to all investigators in their region, as appropriate. The RPI (or delegate) will report significant safety issues (including USM) to SPI in their region, the regulatory authority and applicable HREC/s in accordance with the requirements. The RPI (or delegate) will provide periodic reports of SAE (from Brazil trial sites) to the applicable regulatory authorities and/or HREC/s, as appropriate.

17.5 Appendix 5 The Netherlands Specific Requirements

The following changes will apply for the performance of the protocol in the Netherlands:

Statement of Compliance

This clinical trial will be conducted in compliance with all stipulations of this protocol, the conditions of the ethics committee approval, the NHMRC National Statement on ethical Conduct in Human Research (2007 and all updates), the Integrated Addendum to ICH E6 (R1): Guideline for Good Clinical Practice E6 (R2), dated 9 November 2016 annotated with TGA comments and the NHMRC guidance Safety monitoring and reporting in clinical trials involving therapeutic goods (EH59, 2016), and General Data Protection Regulation (GDPR) , as well as local laws and regulations, such as the Wet medisch-wetenschappelijk onderzoek met mensen (WMO).

2. Recruitment and consent

In Europe, due to ethics regulations, an electronic PICF will not be used and no information on eligibility nor any contact information will be collected prior to the informed consent process being finalized. When participants are interested in the study, they can verify their eligibility with the criteria listed on the website. Then, they will be shown a list of participating centers and advised to contact one of the centers directly to make an appointment for the first study visit. At this visit, the informed consent procedure will be completed in a face-to-face setting where the PICF will be read and signed by both participant and investigator. Exact date of birth will not be collected in the eCRF for the study due to GDPR constraints; instead, year of birth (or 01-01-yyyy) will be used in the eCRF.

3. Data capture methods and data use, storage, access and disclosure during the trial

Archiving will be in compliance with NFU requirements: study data, source documents and the Study File will be kept for 25 years.
EU protocol addendum page_V2.0_20200629

4. COVID-19 testing will be performed via the national testing policy and therefore, the General Practitioner will be notified of the results by the organisation that performs the testing: GGD or the hospital that performs the test.

5. Sharing of contact information

In order to send out the 3, 6, 9, and 12 month questionnaires, the participant's email address will be collected in the REDCap database. No other identifying information will be stored in the database for EU participants.

6. BCG vaccination is not expected to cause an exacerbation of the immune response with adverse consequences, because of 3 main arguments: • By activating anti-viral mechanisms, BCG decreases virus load and systemic inflammation (Arts et al, Cell Host Microbe 2018). Influenza pathophysiology is the same so if BCG had adverse effects, this would have been known for a long time. • Information is available on individuals vaccinated with BCG last year and no COVID19 complications were observed in this group.

17.6 Appendix 6 Spain Specific Requirements

The following changes will apply for the performance of the protocol in Spain:

1. Statement of Compliance

This clinical trial will be conducted in compliance with all stipulations of this protocol, the conditions of the ethics committee approval, the Integrated Addendum to ICH E6 (R1): Guideline for Good Clinical Practice E6 (R2), dated 9 November 2016 and General Data Protection Regulation (GDPR) , as well as local laws and regulations

2. Inclusion criteria

According to recommendations of the competent authority AEMPS (Agencia Española del Medicamento y Productos Sanitarios) If the patient is female, and of childbearing potential, she must have a negative pregnancy test (provided by Sponsor) at the time of inclusion and practice a reliable method of birth control for 30 days after receiving the BCG vaccination. - Woman of Childbearing Potential is defined as a premenopausal female who is capable of becoming pregnant.

3. Recruitment and consent

In Europe, due to ethics regulations, an electronic PICF will not be used and no information on eligibility nor any contact information will be collected prior to the informed consent process being finalized. When participants are interested in the study, they can verify their eligibility with the criteria listed on the website. Then, they will be shown a list of participating centers and advised to contact one of the centers directly to make an appointment for the first study visit. At this visit, the informed consent procedure will be completed in a face-to-face setting where the PICF will be read and signed by both participant and investigator.

Exact date of birth will not be collected in the eCRF for the study due to GDPR constraints; instead, year of birth (or 01-01-yyyy) will be used in the eCRF.

4. Data capture methods and data use, storage, access and disclosure during the trial

Archiving will be in compliance with NFU requirements: study data, source documents and the Study File will be kept for 25 years.

5. Sharing of contact information

In order to send out the 3, 6, 9, and 12 month questionnaires, the participant’s email address will be collected in the REDCap database. No other identifying information will be stored in the database for EU participants.

17.7 Appendix 7 Optional Biological sample collection during episodes of illness

Assessment of immune responses during episodes of illness will provide crucial insights into the mechanisms by which BCG may protect against COVID-19. BCG is proposed to protect against unrelated infections by boosting the innate immune response[1] which can directly protect against infections and also shape the adaptive immune response[2-3]. Biological samples collected after infection provide meaningful insight into the long-lasting effects of the infection and immune memory. However, they do not provide information about the early immune response to infection that can promote early clearance, may impact disease severity and may define the long-lasting memory response. It is this part of the immune response where BCG vaccination may play a crucial role in protection against COVID-19 as well as non-COVID-19 respiratory infections.

Objectives of exploratory sub-study

The additional collection of biological samples from BRACE participants during episodes of febrile or respiratory illness will contribute to the planned subgroup exploratory analyses of BRACE:

11. To determine the impact of BCG vaccination on the immune system that are associated with protection of adult healthcare workers from non-tuberculous infectious diseases including COVID-19.
13. To identify factors (e.g. age, sex, chronic conditions such as diabetes and cardiovascular disease, smoking, asthma, prior BCG vaccination, genetics, influenza vaccination, immunological/molecular factors) that influence adult immune responses and COVID-19 responses.

It will also contribute to the following additional exploratory objectives:

In a sub-set of BRACE trial participants who consent for an optional biological sample to be collected during episodes of fever or respiratory illness:

- To characterise the immune response to SARS-CoV-2 infection
- To compare immune responses during an episode of respiratory illness (COVID-19 or non-COVID-19 illness) in BCG-vaccinated and non-BCG vaccinated participants

Outcomes:

Immune system characterisation and molecular markers of disease in episodes of COVID-19 or non-COVID-19 respiratory or febrile illness from BCG-vaccinated and non-vaccinated participants.

Population: A sub-group of the BRACE trial participants who consent to an optional biological sample to be collected during episodes of fever or respiratory illness.

Study Duration:

As per the BRACE trial protocol – 2 years.

Participant Duration:

12 months from randomisation.

Sub-study Locations:
Optional inclusion for Australian sites.

Sub-study Principal Investigator:
Dr Nicole Messina
Senior Research Officer, Infectious Diseases Group, Murdoch Children's Research Institute, The Royal Children's Hospital, 50 Flemington Road Parkville, 3052 Victoria, Australia
Honorary fellow, Department of Paediatrics at Melbourne Children's Melbourne Medical School, Faculty of Medicine, Dentistry and Health Sciences, The University of Melbourne
Email: nicole.messina@mcri.edu.au

Potential risks and benefits:
Known potential risks:
This sub-study involves minimal risk to participants. Having a blood test can sometimes cause some pain from the needle or be uncomfortable. Occasionally a small amount of bruising can occur on the skin where the blood was taken. Trained members of the study team will collect the blood samples from participants. Having a respiratory swab can sometimes be uncomfortable. Trained members of the study team will collect the respiratory swabs from participants. Self-testing swab kits may be provided as required, with clear instructions to participants on safe self-swabbing technique.

Known potential benefits:
This sub-study is exploratory in nature and is not expected to provide any direct additional benefit to the participants. The findings of the study, however, may be of significant value for understanding the immune response to COVID-19, the off-target effects of BCG vaccination on responses to COVID-19 and other respiratory infections and determinants of disease severity.

Sub-study design:
Consent:
An additional option has been added to the BRACE online consent form to allow participants to optionally consent additional biological sample collection during an episode of illness. The BRACE participant information and consent form (PICF) has been also modified to explain to participants the process for collecting these additional blood samples and why they are being collected. If a participant declines to consent for additional biological sample collection during an episode of illness this will not affect their participation in the BRACE trial (assuming all other inclusion and exclusion criteria are met).

Sample collection process:
Participants consenting for additional biological sample collection during an episode of illness may be contracted by the study team during any episode of respiratory or febrile illness that occurs during their involvement in the BRACE trial (i.e. up to 12 months from randomisation). Sample collection would occur during and up to one month after resolution of an episode of illness with fever or respiratory symptoms. The collection of samples will be done at a study

site (e.g. if they are inpatients or obtaining SARS-CoV-2 testing at a study site) or at the participant's home, depending on the location of the participant.

Samples to be collected are:

-a blood sample

and/or

- saliva/respiratory swab/s

All samples will be collected, processed and stored in accordance with the BRACE trial protocol section 7.3. We will aim to take these samples at the same time as any other clinical or research samples where possible to minimise the number of sample collections for each participant, minimise contact of research staff with infectious patients and to reduce the need for research staff to use vital personal protective equipment (PPE).

What we will do with the sample:

Samples will be processed for analysis of the immune system as detailed in BRACE trial protocol section 3.2. Where indicated, saliva/respiratory swab/s collected will be linked with the relevant public health testing and reporting systems as BRACE trial protocol section 7.3. In addition, samples will be included in the BRACE biobank if participants have also consented for their samples being placed in the BRACE biobank.

References

1. Novakovic B, Messina N, Curtis N. Chapter 6 - The Heterologous Effects of Bacillus Calmette-Guérin (BCG) Vaccine and Trained Innate Immunity. In: Faustman DL, editor. The Value of BCG and TNF in Autoimmunity (Second Edition). Second edition. ed: Academic Press; 2018. p. 71-90.
2. Arts RJW, Moorlag S, Novakovic B, et al. BCG Vaccination Protects against Experimental Viral Infection in Humans through the Induction of Cytokines Associated with Trained Immunity. Cell Host Microbe. 2018;23:89-100 e5.
3. Kleinnijenhuis J, Quintin J, Preijers F, et al. Long-lasting effects of BCG vaccination on both heterologous Th1/Th17 responses and innate trained immunity. J Innate Immun. 2014;6:152-8.

17.8 Appendix 8 Optional Sub-study: collection of blood samples to measure immune responses to COVID-19 specific vaccines.

Sub study locations:
Australia
Brazil

Overview:
COVID-19-specific vaccines are becoming increasingly available and healthcare workers, being at high risk of SARS-CoV-2 exposure, are prioritised for receipt of these vaccines. BCG vaccination alters immune responses to subsequence vaccinations[1,2] and therefore it is plausible that it may boost the immune response to COVID-19-specific vaccines. As healthcare workers, participants in the BRACE trial will be prioritised for receipt of COVID-19-specific vaccines in most regions and as a result will likely receive these vaccines during their involvement of the BRACE trial.

The type of COVID-19-specific vaccine given to BRACE trial participants will vary between sites and it is likely that more than one type of vaccine will be used in a given region. The number of doses given (one or two) and the recommended interval between the two doses are likely to vary as well but are likely to be consistent within a given region.

The BRACE trial exploratory outcomes already include assessment of the effects of vaccines on the immune system (including the effects of BCG-vaccination on immune response to COVID-19-specific vaccines).

To ensure we obtain samples at the optimal times before and after COVID-19-specific vaccination, in a subset of participants, we propose collecting blood samples at up to three additional time-points:

- (Visit 1, site specific) prior to receipt of the first dose of a COVID-19-specific vaccine.
- (Visit 2, site specific) after the first dose of a COVID-19-specific vaccine.
- (Visit 3) 28 days after the second dose of a COVID-19-specific vaccine.

These additional blood samples enable us to:

- screen for prior SARS-CoV-2 exposure (accounted for at analysis), and provide a baseline measure of the immune system prior to receipt of COVID-19-specific vaccines.
- measure the immune response (e.g. antibodies) to the first and second dose of COVID-19-specific vaccines, and other changes in the immune system induced by the COVID-19-specific vaccine.
- compare the vaccine responses to COVID-19-specific vaccines between the BCG and the control group to each COVID-19 specific vaccine.
- compare our findings to other studies on COVID-19-specific vaccines[3].

Determining if BCG vaccination can improve the immune response to COVID-19-specific vaccines have important implications for the potential of BCG vaccination to increase efficacy of COVID-19-specific vaccines and may also impact our interpretation of the outcomes of the BRACE trial. This is particularly important for the COVID-19-specific vaccines that have a lower efficacy.

Objectives of exploratory sub-study

The additional collection of blood samples from BRACE trial participants immediately prior to, and after each COVID-19-specific vaccination will contribute to the existing planned subgroup exploratory analyses of BRACE:

1. *To determine the impact of BCG vaccination on the immune system that are associated with protection of adult healthcare workers from non-tuberculous infectious diseases including COVID-19.*
2. *To determine and compare changes in the immune system induced by vaccination of adult healthcare workers.*
3. *To identify factors (e.g. age, sex, chronic conditions such as diabetes and cardiovascular disease, smoking, asthma, prior BCG vaccination, genetics, other vaccinations including COVID-19-specific vaccines, latent TB, immunological/molecular factors) that influence adult immune responses, infection and COVID-19 risk.*

Population: A sub-group of the BRACE trial participants who receive COVID-19-specific vaccines in regions taking part in the sub-study.

Outcomes: Immune system characterisation and molecular markers of immunity (including seroconversion to SARS-CoV-2) in response to COVID-19-specific vaccines in BCG-vaccinated and non-BCG-vaccinated participants.

Study Duration: As per the BRACE trial protocol – 2 years.

Participant Duration: Up to 4 months from sub-study inclusion

Sub-study Principal Investigator: Prof Nigel Curtis

Potential risks and benefits

<u>Known potential risks</u>

This sub-study involves minimal risk to participants. Having a blood test can sometimes cause some pain from the needle or be uncomfortable. Occasionally a small amount of bruising can occur on the skin where the blood was taken. Trained members of the study team will collect the blood samples from participants.

The amount of blood collected is too small to have any impact on the participants' health. This sub-study will not impact the setting up of COVID-19-specific vaccination clinic at the participating sites. It is not expected to have any negative interactions between the BCG and the COVID-19-specific vaccine.

Known potential benefits
This sub-study is exploratory in nature and is not expected to provide any direct additional benefit to the participants. The findings of the study, however, may be of significant value for understanding the immune response to COVID-19-specific vaccines and the off-target effects of BCG vaccination on responses to COVID-19-specific vaccines.

Sub-study design
Eligibility:

Inclusion Criteria

- Participant in the BRACE trial who has previously consented to be contacted for future ethically approved projects.
- Participant recruited to the BRACE trial at a site taking part in this sub-study.

Exclusion Criteria

- A previous positive SARS-CoV-2 test at any time (not applicable in Brazil).
- Expected inability to provide a blood sample in the indicated time window after: the first dose (visit 2) and/or the second dose (visit 3) of a COVID-19-specific vaccine.
- [site specific]: Inability to provide a blood sample in the indicated time window prior the first dose (visit 1) of a COVID-19-specific vaccine.

Recruitment
Potential BRACE participants will be informed of this sub-study and invited to participate as per their recruitment sites' existing communication approach. BRACE participants will evaluate their eligibility for the sub-study and will have access to the site-specific participant information and consent form (PICF) prior to enrolment in the sub-study.

Consent
An additional participant information and consent form (PICF) will be provided to participants to allow them to optionally consent to this sub-study. If a participant declines to consent for this sub-study it will not affect their participation in the BRACE trial.

Data collection
Participants interested in this sub-study will be contacted by the study team to arrange blood collection if:

- The BRACE trial study site from which they were recruited begins COVID-19-specific vaccinations of staff

Or

- if the participants inform the BRACE trial team that they will receive a COVID-19 specific vaccine.

At these additional sub-study visits, participants will be asked about:

- prior positive COVID-19 tests,
- any other vaccines received since randomisation in BRACE (type, dose, route, date)

- expected date of vaccination with COVID-19-specific vaccine and which vaccine
- episodes of febrile or respiratory illness since last visit (if not already collected as part of the BRACE trial)
- (after vaccination only) adverse reaction to the COVID-19-specific vaccine

After the expected COVID-19-specific vaccine administration date, participants will be contacted as per their recruitment sites' existing communication approach, to confirm which vaccine they have received, where and when they received it, as well as when is the second dose planned.

Sample collection process

Sample collection will occur:

- (Visit 1, site specific) On the day of (or in the 5 to 14 days preceding) the first dose of a COVID-19-specific vaccine.

  [site specific] *Note that for a participant who has already received their first dose of a COVID-19 specific vaccine, the participant's blood sample for the first timepoint will not need to be collected. However, blood samples for the remaining time points below will need to be collected.*
  It is planned to collect blood samples on the same day of vaccination, however we will accept bloods that are taken up to 5 days before the first dose of COVID-19 specific vaccine in all regions, or even up to 14 days before the first dose of COVID-19 specific vaccine in regions where the COVID-19 prevalence is low, are acceptable.

- (Visit 2, site specific) 1 to 28 days (±2) days after the first dose of a COVID-19-specific vaccine

  Note that where the second dose of the COVID-19 specific vaccine is given within 28 days in a given region, this sample will be taken at an earlier time point. Efforts will be made to standardise the interval between the first dose of COVID-19-specific vaccine and the blood sample for each type of COVID-19-specific vaccine within each given region, e.g. within 14 (±2) days after the first dose of COVID-19-specific vaccine if the two doses of COVID-19-specific vaccine are given 2 weeks apart, or within 21 (±2) days after the first dose of COVID-19-specific vaccine if the two doses of COVID-19-specific vaccine are given 3 weeks apart.

  In specific sites, an earlier time-point (<7 days) will enable the exploration of the initial gene expression responses to vaccination.

- (Visit 3) 28 (±2) days after the second dose of a COVID-19-specific vaccine

  Note that efforts will be made to standardise the interval between the COVID-19-specific vaccine doses and the blood collection for both blood collections, for each type of COVID-19-specific vaccine and within a given region.

Blood samples will be collected, processed and stored in accordance with the BRACE trial protocol section 7.3 with the exception that up to 40 mL of blood will be taken at each time point. Also, if this blood collection is done at the same time as a BRACE trial 3-monthly blood collection, an additional 10 mL of blood may be required for a total of 50 mL. The collection of blood samples will be done at a study site or at the participant's home, depending on the region. We will aim to collect these samples at the same time as the existing BRACE Trial 3-monthly blood samples where possible, to minimise the number of sample collections for each participant.

What we will do with the sample:

Samples will be processed for analysis of the immune system as detailed in BRACE trial protocol section 3.2. The immune system will be assessed by several methods, including:

a) measurement of antibodies to SARS-CoV-2 (to assess prior exposure/infection with SARS-COV-2) and their neutralisation ability
b) measurement of antibodies to COVID-19 specific vaccines (to determine seroconversion and antibody titres) and their neutralisation ability
c) characterisation of immune cell subpopulations
d) measurement of immune cell activation and differentiation
e) measurement of immune cell function (e.g. cytokine production and cell division) following *in vitro* stimulation with SARS-CoV-2, COVID-19-specific vaccines, or their components)

Sample size estimation:

As COVID-19-specific vaccines are novel, immune responses following vaccination have yet to be extensively characterised and there is currently no agreed correlate of protection. As such, formal sample size calculations are not possible.

In Australia, based on our previous experience assessing immune responses to other vaccines we estimate that for each region in which this sub-study will take place a sample size of 150 participants per randomisation group and per COVID-19-specific vaccine type (aiming to have 100 participants with blood samples for all three timepoints) will be sufficient to detect a meaningful effect of BCG vaccination on the vaccine responses to COVID-19-specific vaccines. With the expectation that within a region the majority of participants will receive one of two vaccines we will recruit an estimated 600 participants: 150 participants x 2 randomisation groups (BCG or No BCG vaccination) with 2x COVID-19-specific vaccine types.

In Brazil, all BRACE participants will be invited to join the sub-study. This subset of participants provides a unique opportunity to study the influence of natural infection and COVID-19-specific vaccination on both infection and reinfection with SARS-CoV-2, and critically, the impact of variant strains, particularly the P.1 variant. Samples from a large proportion of participants in Brazil will be collected, to optimise capture of participants who may become infected with SARS-CoV-2 different variants.

References

1. Messina NL, Zimmermann P, Curtis N. The impact of vaccines on heterologous adaptive immunity. *Clin Microbiol Infect* 2019; **25**(12): 1484-93.
2. Arts RJW, Moorlag S, Novakovic B, et al. BCG Vaccination Protects against Experimental Viral Infection in Humans through the Induction of Cytokines Associated with Trained Immunity. *Cell Host Microbe* 2018; **23**(1): 89-100 e5.
3. Ramasamy MN, Minassian AM, Ewer KJ, et al. Safety and immunogenicity of ChAdOx1 nCoV-19 vaccine administered in a prime-boost regimen in young and old adults (COV002): a single-blind, randomised, controlled, phase 2/3 trial. *Lancet* 2021; **396**(10267): 1979-93.

17.9 Appendix 9 Optional Sub-study: analysis of swab samples to determine the impact of SARS-COV-2 variants

Sub study locations:
Brazil

Overview:

Eligibility:

Inclusion Criteria

- Participant in the BRACE trial who has previously consented to be contacted for future ethically approved projects.
- Positive SARS-CoV-2 respiratory swab
- Participant recruited to the BRACE trial at a site taking part in this sub-study.

Exclusion Criteria

N/A

Recruitment
BRACE study staff will identify eligible participants for the sub-study. Potential participants will be informed of this sub-study and invited to participate as per their recruitment sites' existing communication approach. Participants will be provided access to the site-specific participant information and consent form (PICF) prior to enrolment in the sub-study.

Consent
An additional PICF will be provided to participants to allow them to optionally consent to this sub-study. If a participant declines to consent for this sub-study it will not affect their participation in the BRACE trial.

Data collection
Participants will be contacted as per their recruitment sites' existing communication approach.

Sample collection process

- Respiratory swabs

  During episodes of illness participants agreed to provide a respiratory swab as part of the main BRACE trial. The swabs collected by the BRACE study team are stored at BRACE sites laboratories, following testing in line with government health guidelines and will be accessed for variant testing. If possible, swabs collected through alternative channels will be accessed for further testing or variant results.

What we will do with the sample:

SARS-CoV-2 variant testing/sequencing of respiratory swabs collected as part of the main BRACE trial.

Sample size estimation:

In Brazil, all BRACE participants who have reported a positive COVID-19 test and the respiratory swab is accessible by the BRACE team will be invited to join the sub-study. This subset of participants provides a unique opportunity to study the impact of variant strains.

Objectives of exploratory sub-study

The analysis of available swabs for SARS-CoV-2 variants will contribute to the existing planned subgroup exploratory analyses of BRACE:

BRACE Protocol exploratory analyses 12. To determine and compare changes in the immune system induced by vaccination of adult healthcare workers.

- ⇨ (Example) to measure the impact of COVID-19-specific vaccines +/- BCG vaccine on the different SARS-CoV-2 variants

BRACE Protocol exploratory analyses 13. To identify factors (e.g. age, sex, chronic conditions such as diabetes and cardiovascular disease, smoking, asthma, prior BCG vaccination, genetics, other vaccinations including COVID-19-specific vaccines, latent TB, immunological/molecular factors) that influence adult immune responses, infection and COVID-19 risk.

- ⇨ (Example) to measure and compare the long-term effects of the different SARS-CoV-2 variants on the immune system
- ⇨ (Example) to identify individual and immunological/molecular factors associated with the risk of COVID-19 caused by the different SARS-CoV-2 variants

Population: A sub-group of the BRACE trial participants who have reported a positive COVID-19 test and the respiratory swab is accessible by the BRACE team or variant data available.

Outcomes:

(i) Influence of COVID-19-specific vaccines +/- BCG vaccine on immunity to and protection against SARS-CoV-2 variants

(ii) Association of immunological factors (e.g. immune responses to prior SARS-CoV-2 infection and vaccination) with infection and COVID-19 caused by the different SARS-CoV-2 variants.

(iii) Changes in immune system caused by infection with the different SARS-CoV-2 variants.

Study Duration: As per the BRACE trial protocol – 2 years.

Participant Duration: As per the BRACE trial protocol – 2 years.

Sub-study Principal Investigator: Prof Nigel Curtis

Potential risks and benefits

Known potential risks

This sub-study involves minimal risk to participants as there are no additional samples required.

Known potential benefits

This sub-study is exploratory in nature and is not expected to provide any direct additional benefit to the participants. The findings of the study, may be of significant value for understanding the impact of SARS-CoV-2 variants on individuals, and the efficacy of the vaccines on the different SARS-CoV-2 variants.

BRACE trial Protocol version tracker			
**Authors:**	Kaya Gardiner, Tenaya Jamieson, Jia Wei Teo		
**Reviewers:**	Prof Nigel Curtis, Dr Laure Pittet (MCRI), Dr Nicole Messina, Ms Susan Perlen, Francesca Orsini		

**Version**	**Date**	**Modifications**	**Signed Protocol Approval Form (PAF)**
1.0	15Mar2020	Original	PAF was not implemented until protocol v9.0.
2.0	16Mar2020	Modifications following peer review	
3.0	26Mar2020	Modifications following Australian HREC review	
4.0	27Mar2020	Modifications following further HREC comments	
5.0	30Mar2020	Amendment 1<br>Additional sub-study (immune system analysis)	
5.1	03Apr2020	Modifications following HREC review of amendment	
6.0	20Apr2020	Amendment 2	
6.1	27Apr2020	Modifications following HREC review of amendment<br>• Addition of details around clinicaltrials.gov registration<br>• Increase of planned number of participants to 10,078, including updated information on trial statistics<br>• Addition of sites to enable recruitment of 10,078 participants<br>• Change to timing of flu vaccine: can be given concurrently with BCG OR can have been given a minimum of 72 hours prior to randomisation<br>• Change to Primary Outcome 2:<br>- definition of severe COVID-19 disease: “COVID-19 positive test, AND hospitalised OR non-hospitalised severe disease”<br>• Change to follow-up blood collection from 6m, to 3m & 12m<br>• Change to follow-up surveys: increase from 6m to 12m<br>• Change to outcome measures: “febrile respiratory illness” revised to “fever OR respiratory illness”<br>• Minor changes for clarity and correction of typographical errors<br><br>Updates to inclusion/exclusion criteria: flu vaccine timeframe changed in IC, BCG adverse reactions added as EC, influenza contraindications added to EC, breastfeeding removed from EC as not required, participation in other COVID-12 prevention trials added as EC	
7	30April2020	Amendment 3	

		• Addition of placebo for those randomised to non-BCG arm<br>• Removal of option of receiving influenza vaccine at same time as BCG vaccine. Participants will have obtained their flu vaccine prior to enrolment and randomisation.<br><br>Addition of further NSW sites (Westmead Hospital & Prince of Wales Hospital)	
7.1	05May2020	Modifications following initial review of amendment	
7.2	08May2020	Modifications following HREC review of amendment	
8.0	29May2020	Amendment 4<br>• Change to definition of healthcare worker for Australian sites – expand to match definition for European sites<br>• Addition of information about data retention and Gates Foundation requirements<br>• Correction of error in product stability information in protocol, to match product information<br>• Addition of appendix describing stool sample collection, to take place at selected sites only<br>• Updates for clarity<br><br>Correction of typographical errors	
8.1	05Jun2020	Addition of information about data retention and Gates Foundation requirements – to accurately reflect the requirements set out in the agreement with the Gates Foundation	
9.0	25Aug2020	• Trial name updated by removing '*following Coronavirus exposure*'<br>• Refining of objective language and definitions, as well as removal of secondary objective<br>• Addition of additional blood samples at 6- and 9-month time points<br>• Recruitment window for the BRACE trial extended to 2.5 years<br>• In line with the window for blood samples extended to 42 days, the timeline for participant follow-up has been updated to 13.5 months from randomisation<br>• Clarification of roles of Chief PI, Regional PI and Site PI, as well as addition of Brazil and UK collaborators<br>• Update to the influenza vaccination eligibility criteria<br>• Review of the Recruitment and Consent section to ensure practical application across all BRACE sites<br>• Inclusion of detail pre-randomisation blood sample<br>• Expansion of needle gauge size under Administration of trial drug<br>• Update to the trial timeline and schedule of assessments<br>• Update to the descriptions of procedures	☒

		• Consolidated procedure discontinuation, withdrawals and losses to follow-up for clarity and include additional detail on processes in Brazil<br>• Safety definitions and information have been consolidated for clarity and a toxicity grading scale has been included<br>• Adjustments made to the data and information management section<br>• Revisions to the description of the BRACE trial Governance structure<br>• Removal of Appendix which outlines division of sponsor responsibilities between Chief PI<br>• Site specific Appendices included	
9.1	22Sep2020	Modifications following HREC review of amendment and the following additional adjustments;<br>• Update of Regional Principal Investigator for Rio de Janerio, Brazil,<br>• A negative PCR test is not proposed as an inclusion criteria for participants in Brazil. Such a screening test was mooted as possible but has since been found to be logistically impossible.<br>• A respiratory swab will be collected at baseline, following informed consent and randomisation. These swabs are being collected as the Brazilian investigators are keen to conduct a COVID prevalence sub-study. These samples cannot be rapidly analysed due to logistic limitations but will be batch analysed at a later date. Brazilian health authorities will be informed of the results after analysis. Results will be shared with participants approximately 3 months after randomisation. Participants will be specifically told that they won't be informed of the results for approximately 3 months.<br>• Expansion of needle gauge size under Administration of trial drug to include Preference to use 25G or 26G accepted up to 30G to incorporate BCG administration practice in Europe,<br>• Refinement of SUSAR definition of expectedness to align with WHO information sheet,<br>• Update to Appendix 4 Brazil Specific Requirements;<br>○ To clarify safety reporting roles<br>○ Adjustment of approach to collection of respiratory swab at baseline with additional detail included outlining the strategy.	☒
10.0	26Nov2020	Initiated to address a number of significant contextual changes impacting the BRACE trial.	☒

**AMENDMENT TO OUTCOMES**	(1) Administrative correction to the Objective 2 and 4 to ensure minor adjustment to correct language and ensure consistency of wording throughout the protocol.<br><br>(2) Secondary outcomes 5, 6 and 7 will analyze data over the 6 and 12 months following randomization.<br><br>(3) Inclusion in exploratory outcome 13 specific reference factors including COVID-19 vaccines that influence adult immune responses, infection and COVID-19 risk.
**1.3 EXPECTED DURATION OF STUDY**	In light of the operational requirements to operate the trial across 5 countries and contextual changes, to reach recruitment target a longer recruitment period will be required. Recruitment will be extended until the end in March 2021.
**1.4 PROTOCOL REVISION PREPARATION**	Administrative adjustment to remove this section.
**4.1 OVERALL DESIGN**	• Inclusion of some specific site details ie. site names in Brazil.<br>• Inclusion of electronic messaging as a form of follow-up as preferred by study teams and participants in Brazil.
**4.3.2 INCLUSION CRITERIA**	Collaborators in Europe have reported a number of operational challenges in the access and availability of the influenza vaccine for healthcare workers in 2020. In addition, influenza vaccination levels in Spain are significantly lower than other sites. Therefore after discussion and review of the impact on recruitment and the trial, proposing the adjustment of the inclusion criteria related to influenza vaccination to the below. Participant's influenza vaccination status is recorded during randomisation and at 3 month periodic surveys.<br>• *Australian sites only: Has received the influenza vaccine at least 72 hours prior to randomisation*
**4.3.3 EXCLUSION CRITERIA**	Additional exclusion criteria to ensure exclusion of people receiving antibiotics as a preventative treatment against TB, additional detail on the definition of previous SARS-CoV-2 to include positive PCR and approved antigen testing and clarification on alternative site.

	• *Currently receiving long term (more than 1 month) treatment with isoniazid, rifampicin or quinolone as these antibiotics have activity against Mycobacterium bovis*<br>• *Have previously had a SARS-CoV-2 positive test result (positive PCR on a respiratory sample or a positive SARS-CoV-2 diagnostic antigen test approved by the local jurisdiction's public health policy)*<br>• *People with active skin disease such as eczema, dermatitis or psoriasis at or near the site of vaccination*<br>○ *A different adjacent site on the upper arm can be chosen if necessary*
**5.8 EXCLUDED MEDICATIONS AND TREATMENTS**	In last protocol amendment (8.1 to 9.1), the timeline for participants not taking part in any other COVID-19 preventative intervention clinical trial was revised from 6 months to 13.5 months, which reflects the full period of recruitment. However, with the trial measuring primary and secondary outcomes at 6m timepoint and quickly evolving COVID-19-specific vaccine environment, adjusting the timeline to 6 months.
**7.3 DESCRIPTION OF PROCEDURES**	Inclusion of electronic messaging as a form of follow-up as preferred by study teams and participants in Brazil.<br><br>Additional detail included on the periodic questionnaires sent to participants.<br><br>Under the revised protocol, blood samples at 9 and 12 months will be collected in a sub-set of participants. The specific sub-set of participants will be agreed with Regional Principal Investigators based on contextual, operational and financial considerations. All participants will still be followed up with app and survey however blood samples may not be collected from all participants.<br><br>Adjustment to timepoints defined for dried blood spots to enable operational flexibility to collect across a number of timepoints.

		**8.5 ASSESSING THE SEVERITY OF A PARTICIPANT'S AE**: Itch added to the toxicity grading scale in line with updated BRACE SAE/AE SOP.<br><br>**11.3 METHODS OF ANALYSIS**: Additional sub-group analysis included.<br>- *Had a positive serology to SARS-CoV-2 when enrolling into the trial*	
10.1	10Dec2020	Protocol amendment v10.0 to v 10.1 proposes some additional adjustments and the submission package will include responses to HREC queries received 9th Dec 2020.<br><br>**4.3.3 EXCLUSION CRITERIA**: Due to the rapid roll-out of COVID-19-specific vaccines in the United Kingdom, additional exclusion criteria proposed;<br>○ *Have previously received a COVID-19-specific vaccine*<br><br>**7.3 DESCRIPTION OF PROCEDURES**: Proposal to retain ad hoc blood sample collection due to the changeable context and enable additional sample collection if required.<br><br>**APPENDIX 4. BRAZIL SPECIFIC**: Adjustment to language around COVID-19 testing in Campo Grande and Rio.	☒
10.2	08Feb2021	Addition of Appendix 8 Optional Sub-study: collection of blood samples to measure immune responses to COVID-19 specific vaccines<br><br>**APPENDIX 8. Optional Sub-study: collection of blood samples to measure immune responses to COVID-19**: With the availability of COVID-19-specific vaccines, healthcare workers are being prioritized to receive COVID-19-specific vaccines due to their high risk of SARS-CoV-2 exposure. As healthcare workers, participants in the BRACE trial will be prioritized to receive a COVID-19-specific vaccine.<br><br>There is evidence that BCG can improve the immune responses to other vaccines, so it is possible that this will also apply to COVID-19-specific vaccines as well.<br><br>The inclusion on Appendix 8 Optional sub-study: a collection of blood samples to measure	☒

<table>
<tr>
<td></td>
<td></td>
<td>
<table>
<tr>
<td>specific vaccines</td>
<td>immune responses to COVID-19-specific vaccines will enable us to determine if BCG vaccination can improve immunity to COVID-19- specific vaccines have important implications for the potential of BCG-vaccination to increase efficacy of COVID-19- specific vaccines, and may also impact our interpretation of the outcomes of the BRACE Trial. This is particularly important for the COVID-19-specific vaccines that have a lower efficacy (e.g. less than 90% efficacy).

The changes in protocol v10.2 will only apply to sites that agree to Appendix 8. Site will need to submit protocol v10.2 and the site-specific PICF to their HREC/Governance for approval. Local COVID-19 safe plans will be utilized to ensure researcher/participant safety in relation to COVID-19

Protocol have been updated in response to HREC queries on the 04Feb2021
- Sub study locations have been amended to Australia and Brazil.
- For Visit 1 blood sample collection (pre vaccination blood) the following information have been included
o [site-specific] Note that for a participant who hasd already received their first dose of a COVID-19- specific vaccine the participant's blood sample for the first timepoint will not need to be collected. However, blood samples for the remaining time points below will need to be collected
- Sample size estimation for the sub study have been increased as follow:
o for each region in which this sub-study will take place (e.g. Australia and Brazil) a sample size of 150 participants per randomisation group and per COVID-19-specific vaccine type (aiming to have 100 participants with blood samples for all three timepoints) will be sufficient to detect a meaningful effect of BCG vaccination on the vaccine responses to COVID-19-specific vaccines.
o Sub study will recruit up to a total of 1200 participants.</td>
</tr>
</table>
</td>
<td></td>
</tr>
<tr>
<td>10.3</td>
<td>11Feb 2021</td>
<td>Update of Appendix 4 Optional Sub-study: collection of blood samples to measure immune responses to COVID-19 specific vaccines

<table>
<tr>
<td>APPENDIX 4. Optional Sub-study: collection of blood samples to</td>
<td>For Visit 1 – Blood can be collected up to 14 days before participant receive the first dose of a COVID-19 specific vaccine.

In specific sites, an earlier time-point (<7 days) will enable the exploration of the initial gene</td>
</tr>
</table>
</td>
<td>☒</td>
</tr>
</table>

		**measure immune responses to COVID-19 specific vaccines**	expression responses to vaccination.<br><br>For Visit 2 – Blood can be collected up to 28 days instead of 28 days only after the participant have received the first dose of COVID-19 specific vaccine.<br><br>The changes implemented is to allow flexibility in collecting blood sample. For visit 2 with the increase of the number of days it has provided the study with an opportunity to explore the initial gene expression to vaccination but this will be site specific only.	
11.0	04Jun2021	Update information on interim analysis and to align Appendix 8 information with the ethically approved protocol in Brazil.		☒
		**Protocol Synopsis**	Included Manaus, Amazonas, Brazil as the third city that will be running the study.	
		**INVESTIGATOR AGREEMENT**	Added Prof Marcus Vinicius Guimaraes de Lacerda who is the region Principal Investigator for for Manaus, Amazonas, Brazil.	
		**Section 4.1**	Added Fundação de Medicina Tropical and Health State Office as the principal site for Manaus, Amazonas, Brazil.	
		**Section 10.1.2**	Updated information on interim analysis	
		**Section 11.1**	Update information on interim analysis	
		**Section 11.4**	Updated the interim analysis to make the following key changes:<br>The interim analysis will consider severe episodes of COVID-19 by 6 months now only in Stage 2 of the trial.<br>• Stage 1 includes only Australia, which has had negligible COVID-19.<br><br>For the primary outcome of severe COVID-19, the strategy has been changed to split the alpha.<br>• The DSMB had recommended unbinding and dissemination of the interim analysis results if they determined they were of clinical or public health importance<br>• A larger alpha spend on the interim analysis will provide the best chance of the interim analysis providing results that the DSMB can recommend be unblinded.	

		**APPENDIX 8. Optional Sub-study: collection of blood samples to measure immune responses to COVID-19 specific vaccines** — The Appendix have been updated to align information with the currently ethically approved protocol in Brazil. The changes are:<br>• A larger number of participants will be recruited to the BCOS sub-study in Brazil to enable the identification of biomarkers predictive of vaccine efficacy against variants.<br>○ This analysis is in line with exploratory outcome No 13.<br>• Manaus will provide more participants in a region with a high prevalence of the P.1 variant.	
11.1	14Sep2021	Update the protocol to include Planned Exploratory Analyses 14 which is only relevant to Brazil.<br><br>**Section 3.1.3 Planned exploratory analyses** — Addition of Planned Exploratory Analyses 14 (Brazil Specific) to identify biomarkers for diagnosing TB infection.<br><br>**APPENDIX 4 Brazil Specific Requirements** — Updates to include the additional volume of blood to be collected for Planned Exploratory Analyses.	☒
11.2	11Nov2021	Update the protocol to include Appendix 9 Optional Sub-study: analysis of swab sample to determine the impact of SARS-CoV-2 variants, which is only relevant to Brazil.<br><br>**APPENDIX 9 Optional Sub-study** — Addition of Appendix 9 (Brazil Specific) to examine the interplay between SARS-CoV-2 variants and the immune system (for example the immune responses to SARS-CoV-2 and vaccines). This is for testing the respiratory swabs previously collected for further analysis, or access variant results. No additional clinic visits or samples required from participants.<br>An additional PICF will be provided to participants to optionally consent to this sub-study, to test the respirator	☒
12.0	17May2022	Protocol updates to match the wording in the SAP analysis.<br>**Overall** — All revisions made to the protocol are minor/administrative in nature and do not impact the safety or scientific value of the clinical study. Revision have also been done to align with the Statistical Analysis Plan.<br>**Section** | **Detail** | **Summary / Rationale**	☒

**1.4 Stakeholder involvement**	Update table to include Murdoch Children's Research Institute (MCRI) as a stakeholder.	MCRI is the sponsor of the study and will need to be included as a stakeholder
**3.1 Objectives**	Replace "COVID-19 disease" with "COVID-19"	Administrative update
	Replace "SARS-CoV-2-proven respiratory illness" with "COVID-19 episode	
	Remove "adult" from healthcare work	
	Removed "exposed to SARS-COV-2"	
	Objective 7: deleted reference to timepoint "6 months"	Administrative updates to match Objective 7 information in 3.2 Outcomes table
	Objective 8: include the timepoint "over 6 and 12months following randomisation"	To clarify at which timepoint will objective be assessed
	Replace "COVID-19 disease" with "Symptomatic COVID-19	"Symptomatic" word has been added to distinguish COVID 19 from Severe COVID19
**3.2 Outcomes**	Replace "COVID-19 disease" with "Symptomatic COVID-19	"Symptomatic" word has been added to distinguish COVID-19 from Severe COVID-19
	Replace "antigen" with "RAT"	To specific RAT is the antigen test
	Objective 2: Update Case definition of Symptomatic and Severe COVID-19	Clarify the case definitions for Symptomatic and Severe COVID-19 (e.g. ensure matching format for the co-primary outcomes 1)
	Remove "Number of participants"	"Number of participants with" is removed as the outcome is what Sponsor will be collecting from participant. For instance, "symptomatic COVID19 over 6 months" is collected from participants, whereas "Number of participants with symptomatic COVID-19" is what Sponsor's will analyse/compare between the groups.

			Removed "exposed to SARS-COV-2"	Administrative update	
			Objective 5: Include 6 month timepoint	To match with information in objective 5 outcome	
			Update definition of asymptomatic SARS-CoV-2 infection	Clarify the case definitions for asymptomatic COVID-19 is (e.g. ensure matching format for the outcomes)	
			Removal of "abnormal chest X-ray"	Participant medical record which will be assessed as stated in the outcomes will contain record participant chest X-ray	
			Replace "associated with a positive SARS-CoV-2 test" with "due to COVID-19"	Administrative update	
			Removal of accessing death registry	Assessing of death registry will not be performed	
			Objective 7: Include definition for Severe fever or respiratory illness	Clarify the definition of what is severe fever or respiratory illness	
			Objective 7: Inclusion of "for", "within", or "as a consequence of" for "febrile or respiratory illness" for outcomes related to objective 7	Clarification of secondary outcomes related to febrile or respiratory illness	
			Objective 8: Include 6 and 12 month timepoint	To clarify at which timepoints will objective be assessed and to match with information in objective 8 outcomes	
			Objective 8: Include "an acute illness or hospitalisation"	To clarify that BRACE will look at the number of days of unplanned absenteeism due to acute illness or hospitalisation instead of any reason which can include carer' leave or annual leave.	
			Objective 9 outcomes	Administrative update to AE	
			Objective 11	Administrative update to match with objective	

		11 information in section 3.1.3
	Objective 13 outcomes	Administrative update to align with information in objective 13
**4.1 Overall design**	Replace “Flu vaccination” with “Influenza vaccination”	Administrative update
**4.1 Overall design**	Include participating site names in the Netherlands, Spain and UK	To list all participating sites name
**4.6 Recruitment and Consent**	Replace “healthcare card number (or equivalent)” with “healthcare card number (or equivalent) except for Australia”	For Australians healthcare card number will not be collected
**5.2.7 Product accountability**	Update to state In Rio De Janeiro and Manus product accountability will be managed through the collaborating institution	To provide updated information
**6.1 Concealment mechanism**	Replace “incidence of symptomatic and severe COVID-19 disease or admission to hospital for COVID-19 disease” with “symptomatic and severe COVID-19 disease or admission to hospital for COVID-19”	To align information with outcome
**7.2 Schedule of assessments**	Correction made to Time point	Provide corrected information (e.g randomisation visit timepoint is t_0 not t_1
**7.3 Description of procedures**	Replace “daily diary for the two weeks following randomisation” with<br><br>“daily diary for the two weeks following injection”	To clarify that the daily diary will only be completed after participant had received injection
	Replace swab “results will be collected via self-report in the 3 monthly questionnaires” with “results will be collected via self-report”	To clarify that participant reports their swab results at any time via the app or 3 monthly questionnaires.

	Data Linkage is removed	Data linkage will not be conducted for the study
**9.2.1 Data generation (source data)**	Removal of collection of Medicare number in Australia	Update information to state that Medicare number will not be collected
**9.2.6 Data Sharing**	Included Bill and Melinda Gates foundation requirements on data sharing	To provide further detail of the data sharing requirements including identification of the clinical trials data sharing repository (Vivli)
**9.2.8 Data retrieval and linkage**	Update to sub heading title and Removal of data linkage information	Data linkage will not be conducted for the study.
**10.1.3 Independent Safety Monitor**	Update to state that after June 2021 the independent monitor will review report of sae and specified non-serious adverse events of interest reports once every 3 months instead of stopping review after the recruitment period.	This to ensure that participants safety is monitored till end of the study,
**11.1 Sample Size Estimation**	Include information on excluding Stage 1 data for meta-analysis of COVID-19-related outcomes and aligning information with outcomes	As Stage 1 recruitment is done in Australia for Victoria and Western Australia sites, where there is negligible COVID-19 exposure risk, there is a high probability that positive SARS-CoV-2 serology results are false positive. As a result Stage 1 data will not be included in Meta-Analysis. This update have been done to also align with the Statical Analysis Plan.

murdoch children's research institute

<table>
<tr><td>RCH HREC 62586</td><td rowspan="3">Statistical Analysis Plan</td></tr>
<tr><td>Protocol v12.0 27Apr2022</td></tr>
<tr><td></td></tr>
</table>

RCH HREC/protocol no: 62586

NCT04327206

BCG vaccination to Reduce the impact of COVID-19 in healthcare workers (BRACE) Trial

Statistical Analysis Plan

Document Version History

Version Date	Version	Author	Signature	Change Description	Reason/Comment
18-May-2022	1	Francesca Orsini		Initial release.	Not applicable.

TABLE OF CONTENTS

LIST OF ABBREVIATIONS 4

1. STUDY OBJECTIVES 5
   1.1. PRIMARY OBJECTIVE 5
   1.2. SECONDARY OBJECTIVES 5
   1.3. PLANNED EXPLORATORY OBJECTIVES 6

2. BACKGROUND/INTRODUCTION 6
   2.1. STUDY DESIGN 6
   2.2. INTERVENTION GROUPS 6
   2.3. STUDY POPULATION 7
   2.4. SAMPLE SIZE 8
   2.5. STUDY PROCEDURE 10

3. POPULATIONS OF ANALYSIS 11

4. OUTCOME VARIABLES 12
   4.1. DATA COLLECTION 12
   4.2. PRIMARY OUTCOMES 13
   4.3. SECONDARY OUTCOMES 15
   4.4. OTHER VARIABLES 24

5. STATISTICAL METHODOLOGY 25
   5.1. GENERAL PRINCIPLES 25
   5.2. DEFINITION OF BASELINE 26
   5.3. DEFINITION OF THE 6- AND 12-MONTH CUT-OFFS 26
   5.4. DESCRIPTIVE STATISTICS 27
   5.5. THE ESTIMAND FRAMEWORK 27
   5.6. ANALYSIS SOFTWARE 27

6. PRIMARY OUTCOMES 28
   6.1. ESTIMANDS 28
   6.2. PRIMARY ANALYSIS 29
   6.3. SUPPLEMENTARY ANALYSES 30
   6.4. SUBGROUP ANALYSES 33
   6.5. SENSITIVITY ANALYSES 34

7. SECONDARY COVID-19 RELATED OUTCOMES 35
   7.1. SYMPTOMATIC COVID-19 AND SEVERE COVID-19 BY 12MONTHS (#3 AND #4) 36
   7.2. TIME TO FIRST SYMPTOM OF COVID-19 (#5A AND #5B) 38
   7.3. NUMBER OF EPISODES OF COVID-19 (#6A AND #6B) 41
   7.4. ASYMPTOMATIC SARS-COV-2 INFECTION (#7) 43
   7.5. NUMBER OF DAYS UNABLE TO WORK DUE TO COVID-19 (#8A AND #8B) 45
   7.6. NUMBER OF DAYS CONFINED TO BED DUE TO COVID-19 (#9A AND #9B) 46
   7.7. NUMBER OF DAYS WITH SYMPTOMS DUE TO COVID-19 (#10A AND #10B) 48
   7.8. PNEUMONIA DUE TO COVID-19 (#11A AND #11B) 49
   7.9. NEED OF OXYGEN DUE TO COVID-19 (#12A AND #12B) 51
   7.10. ADMISSION TO CRITICAL CARE DUE TO COVID-19 (#13A AND #13B) 52
   7.11. NEED OF MECHANICAL VENTILATION DUE TO COVID-19 (#14A AND #14B) 53
   7.12. HOSPITALISATION DUE TO COVID-19 (#15A AND #15B) 55
   7.13. DEATH DUE TO COVID-19 (#16A AND #16B) 56

8. NON-COVID19 RELATED SECONDARY OUTCOMES 57
   8.1. FEVER OR RESPIRATORY ILLNESS (#17) 57
   8.2. SEVERE FEVER OR RESPIRATORY ILLNESS (#18) 58
   8.3. NUMBER OF EPISODES OF FEVER OR RESPIRATORY ILLNESS (#19) 59
   8.4. NUMBER OF DAYS UNABLE TO WORK DUE TO FEVER OR RESPIRATORY ILLNESS (#20) 60
   8.5. NUMBER OF DAYS CONFINED TO BED DUE TO FEVER OR RESPIRATORY ILLNESS (#21) 60

8.6. NUMBER OF DAYS WITH SYMPTOMS DUE TO FEVER OR RESPIRATORY ILLNESS (#22) 61
8.7. PNEUMONIA (#23) 61
8.8. NEED OF OXYGEN (#23) 62
8.9. ADMISSION TO CRITICAL CARE FOLLOWING A FEBRILE OR RESPIRATORY ILLNESS (#25) 63
8.10. NEED OF MV FOR A FEBRILE OR RESPIRATORY ILLNESS (#26) 63
8.11. DEATH AS A CONSEQUENCE OF AN EPISODE OF FEVER OR RESPIRATORY ILLNESS (#27) 64
8.12. HOSPITALISATION FOR AN EPISODE OF FEVER OR RESPIRATORY ILLNESS (#28) 64
8.13. NUMBER OF DAYS OF UNPLANNED ABSENTEEISM FOR AN ACUTE ILLNESS OR HOSPITALISATION (#29A AND #29B) 65
8.14. ADVERSE EVENTS AND SERIOUS ADVERSE EVENTS 66

9. META-ANALYSIS **66**

10. PLANNED ANALYSES **67**

11. REFERENCES **68**

12. SIGNATURES PAGE 69

LIST OF ABBREVIATIONS

AE	Adverse Event
App	Smartphone Application
AR	Adverse Reaction
BCG	Bacille Calmette-Guérin
BMI	Body Mass Index
BRACE	BCG vaccination to Reduce the impAct of COVID-19 in hEalthcare workers
CI	Confidence Interval
COVID-19	Coronavirus Disease of 2019
DSMB	Data Safety Monitoring Board
GST	Group Sequential Test
ICU	Intensive Care Unit
ITT	Intent-To-Treat
LOCF	Last Observation Carry Forward
PCR	Polymerase Chain Reaction
RAT	Rapid Antigen Test
SAE	Serious Adverse Event
SARS-CoV-2	Severe Acute Respiratory Syndrome Coronavirus 2
SD	Standard Deviation
SE	Standard Error
TSC	Trial Steering Committee
MedDRA	Medical Dictionary for Regulatory Activities
WHO DD	World Health Organization Drug Dictionary

1. STUDY OBJECTIVES

1.1. PRIMARY OBJECTIVE

1. To determine if BCG vaccination (Intervention) compared with placebo (Comparator) reduces the incidence of 'symptomatic COVID-19' (Outcome) measured over the 6 months following randomisation (Time) in healthcare workers (Participants).
2. To determine if BCG vaccination (Intervention) compared with placebo (Comparator) reduces the incidence of 'severe COVID-19' (COVID-19-related death, hospitalisation, or non-hospitalised severe disease, defined as 'non-ambulant'[1] for ≥ 3 consecutive days OR Unable to work[2] for ≥ 3 consecutive days) (Outcome) measured over the 6 months following randomisation (Time) in healthcare workers (Participants).

[1] "pretty much confined to bed (meaning finding it very difficult to do any normal daily activities)"

[2] "I do not feel physically well enough to go to work"

Two primary outcomes have been chosen for this study: occurrence of COVID-19 and occurrence of severe COVID-19. Considering the number of unknown factors and the little knowledge of this new virus, we deemed it of clinical importance to have sufficient power to detect the potential effect of BCG vaccine compared to control for both outcomes. Our hypothesis is that, compared to control, the BCG vaccine will reduce both the number of cases of COVID-19 (increase the number of asymptomatic SARS-CoV-2 infections) and the number of cases of severe COVID-19. In other words, we hypothesise that BCG vaccine will shift the "severity of COVID-19" curve down, i.e., generally reduce the severity of the symptoms. The method used to control type I error is explained in the sample size section (11.1).

1.2. SECONDARY OBJECTIVES

3. To determine if BCG vaccination (Intervention) compared with placebo (Comparator) reduces the incidence of symptomatic COVID-19 (Outcome) measured over the 12 months following randomisation (Time) in healthcare workers (Participants).
4. To determine if BCG vaccination (Intervention) compared with placebo (Comparator) reduces the incidence of severe COVID-19 (non-hospitalised severe disease, hospitalisation or death) (Outcome) measured over the 12 months following randomisation (Time) in healthcare workers (Participants).
5. To determine if BCG vaccination (Intervention) compared with placebo (Comparator) prolongs the time to first COVID-19 episode (Outcome) measured over 6 and 12 months following randomisation (Time) in healthcare (Participants).
6. To determine if BCG vaccination (Intervention) compared with placebo (Comparator) reduces the severity of COVID-19 (Outcome) measured over 6 and 12 months following randomisation (Time) in healthcare workers (Participants).
7. To determine if BCG vaccination (Intervention) compared with placebo (Comparator) reduces the rate and severity of illness (fever or at least one sign or symptom of respiratory disease) measured over 12 months following randomisation (Time) in healthcare workers (Participants).
8. To determine if BCG vaccination (Intervention) compared with placebo (Comparator) reduces absenteeism (days off work) measured over 6 and 12 months following randomisation (Time) in healthcare workers (Participants).
9. To evaluate the safety of BCG vaccination in healthcare workers.

1.3. PLANNED EXPLORATORY OBJECTIVES

10. To determine in a subgroup of adults with recurrent cold sores whether BCG vaccination compared with placebo reduces herpes simplex recurrences (such as cold sores).
11. To determine the BCG vaccination induces changes in the immune system that are associated with protection against non-tuberculous infectious diseases including COVID-19.
12. To determine and compare changes in the immune system induced by vaccination.
13. To identify factors (e.g. age, sex, chronic conditions such as diabetes and cardiovascular disease, smoking, asthma, prior BCG vaccination, genetics, other vaccinations including COVID-19-specific vaccines, latent TB, immunological/molecular factors) that influence immune responses, infection and COVID-19 risk.
14. (Brazil specific) To identify biomarkers for diagnosing TB infection.

2. BACKGROUND/INTRODUCTION

2.1. STUDY DESIGN

BRACE is a phase III, two arms, multicentre, randomised placebo-controlled trial in healthcare workers to determine if BCG vaccine reduces the incidence and the severity of COVID-19 during the SARS-CoV-2 pandemic. At trial design, we planned to randomise 7,244 healthcare workers 1:1 to receive BCG or placebo.

Initially the trial was designed to compare primary and secondary outcomes between BCG and no BCG which was given concurrently with the influenza vaccination (Stage 1). The trial was then expanded to international sites and the design was revised to compare primary and secondary outcomes between BCG and a placebo (Stage 2). The comparison of BCG vs placebo in Stage 2 is the primary analysis of interest, however it is planned to combine the data from the two stages of the trial in a meta-analysis for secondary analyses of the non-COVID-19 outcomes. The analysis plan for this pre-planned meta-analysis is specified in section 9.

The two stages of the study are detailed below:

Dates	Stage	Planned Sample Size	Intervention	Control	Blinding
30th Mar 2020 to 13th May 2020	Stage 1	2,834	BCG+ Influenza	Influenza	Unblinded
14th May 2020 to 1st April 2021	Stage 2	7,244	BCG	Placebo	Blinded

As part of the monitoring, there was a formal interim analysis of the efficacy data. The details of this interim analysis are in a separate SAP [https://doi.org/10.25374/MCRI.14721309.v1]. This analysis compared the number of cases of severe COVID-19 (primary outcome 2) between the BGG group and the control group for those recruited after the introduction of the placebo (Stage 2 of the study).

2.2. INTERVENTION GROUPS

Participants were randomly allocated in a 1:1 ratio to the BCG vaccine group or to the control group in both Stage 1 and 2 of the study. Randomisation was stratified by:

- stage of the study (prior to or post the addition of the placebo vaccination);
- study site;
- age (<40 years; 40 to 59 years; ≥60 years); and
- presence of comorbidity (any of diabetes, chronic respiratory disease, cardiac condition, hypertension).

The BCG vaccine group received an adult dose of 0.1 mL of BCG vaccine SSI injected intradermally over the distal insertion of the deltoid muscle onto the humerus (approximately one third down the upper arm).
The control group in Stage 2 of the trial received 0.1 mL of 0.9% NaCl (placebo) injected intradermal over the distal insertion of the deltoid muscle onto the humerus. The control group in Stage 1 of the trial received the influenza vaccine on the day of randomisation. In Stage 2 of the trial, the control group received a placebo in an effort to blind participants to their treatment group allocation (although the subsequent local reaction at the injection site with BCG vaccination prevents total blinding).
Members of the trial team, except immunisers, are also blinded to the group allocation in Stage 2, achieved by hiding or removing the treatment group variable and all other variables related to BCG from the dataset, and will remain blinded until the database is locked for analysis.

2.3. STUDY POPULATION

Participants are adult (≥18 years) healthcare workers from Europe (the Netherlands, Spain and the United Kingdom), South America (Brazil) and Australia.

INCLUSION CRITERIA
- ≥ 18 years of age
- Healthcare worker
    - defined as anyone who works in a healthcare setting or has face-to-face contact with patients.
- Provide a signed and dated informed consent form
- Pre-randomisation blood collected
- Australian sites only: If annual influenza vaccination is available, receiving the influenza vaccine is an eligibility requirement. The influenza vaccine will be required a minimum of 3 days in advance of randomisation in the BRACE trial.

EXCLUSION CRITERIA
- Has any contraindication to BCG vaccine:
    - Fever or generalised skin infection (where feasible, randomisation can be delayed until cleared)
    - Weakened resistance toward infections due to a disease in/of the immune system
    - Receiving medical treatment that affects the immune response or other immunosuppressive therapy in the last year.
        - These therapies include systemic corticosteroids (≥20 mg for ≥2 weeks), non-biological immunosuppressant (also known as 'DMARDS'), biological agents (such as monoclonal antibodies against tumour necrosis factor (TNF)-alpha).
    - Congenital cellular immunodeficiencies, including specific deficiencies of the interferon-gamma pathway
    - Malignancies involving bone marrow or lymphoid systems
    - Any serious underlying illness (such as malignancy)
        - NB: People with cardiovascular disease, hypertension, diabetes, and/or chronic respiratory disease are eligible if not immunocompromised, and if they meet other eligibility criteria
    - Known or suspected HIV infection, even if they are asymptomatic or have normal immune function.
        - This is because of the risk of disseminated BCG infection
    - Active skin disease such as eczema, dermatitis or psoriasis at or near the site of vaccination
        - A different adjacent site on the upper arm can be chosen if necessary
    - Pregnant
        - Although there is no evidence that BCG vaccination is harmful during pregnancy, it is a contra-indication to BCG vaccination. Therefore, we will exclude women who think they could be pregnant or are planning to become pregnant within the next month.

        - UK specific: Although there is no evidence that BCG vaccination is harmful during pregnancy, it is a contra-indication to BCG vaccination. Therefore, we will exclude women of childbearing potential (WOCBP) who think they could be pregnant.
        - Spain specific: If the patient is female, and of childbearing potential, she must have a negative pregnancy test (provided by Sponsor) at the time of inclusion and practice a reliable method of birth control for 30 days after receiving the BCG vaccination.
    - Another live vaccine administered in the month prior to randomisation
    - Require another live vaccine to be administered within the month following BCG randomisation
        - If the other live vaccine can be given on the same day, this exclusion criteria does not apply
    - Known anaphylactic reaction to any of the ingredients present in the BCG vaccine
    - Previous active TB disease
- Currently receiving long term (more than 1 month) treatment with isoniazid, rifampicin or quinolone as these antibiotics have activity against *Mycobacterium bovis*
- Previous adverse reaction to BCG vaccine (significant local reaction (abscess) or suppurative lymphadenitis)
- BCG vaccine given within the last year
- Previous positive SARS-CoV-2 test result (PCR on a respiratory sample or SARS-CoV-2 antigen test approved by the local jurisdiction's public health policy)
- Already part of this trial, recruited at a different site/hospital.
- Participation in another COVID-19 prevention trial
- Previously received a COVID-19-specific vaccine

2.4. SAMPLE SIZE

ORIGINAL SAMPLE SIZE

The original sample size was calculated based on the two primary outcomes of: (1) the proportion of participants with COVID-19; and (2) the proportion of participants with severe COVID-19, by 6 months following randomisation. Since the trial aims to assess two primary outcomes, an adjustment for multiplicity was applied to maintain a global Type I error rate of 5% by splitting of this alpha.

The original sample size was based on the following:

i) 7244 healthcare workers recruited in Stage 2 would provide 80% power to detect a risk ratio of 0.67 (equivalent to a 1.3% absolute difference) in the BCG group compared to the control group for severe COVID-19 at 6 months (primary outcome 2), assuming 4% of subjects will have severe COVID-19 by 6 months in the control group and allowing for 16% lost to follow-up by 6 months (2-sided alpha =0.04).

ii) 2016 healthcare workers would provide 95% power to detect an absolute difference of 10% in incidence of COVID-19 (primary outcome 1), assuming 55% of subjects will have COVID-19 in the control group (2-sided alpha = 0.005).

iii) In the pre-planned meta-analysis, 10,078 healthcare workers recruited in Stages 1 and 2 would provide 90% power to detect a risk ratio of 0.67 (equivalent to an absolute risk difference of 1.3%) in the BCG group compared to the control group for severe COVID-19 at 6 months (primary outcome 2), assuming 4% of subjects will have severe COVID-19 by 6 months in the control group and allowing for 20% lost to follow-up by 6 months (2-sided alpha = 0.04).

iv) We originally allocated alpha=0.005 to an efficacy interim analysis using the conservative approach of splitting the alpha allocated to primary outcome (2) between the interim and final analysis. Under the original sample size calculation in Stage 1 of the trial we planned to recruit 1,668 participants per group which gave us 72% power to identify a reduction from an incidence of 4% in severe COVID-19 at 6 months

in the control group to 2% in the intervention group. If the assumptions were correct, this would equate to 100 cases in total. We therefore planned a formal interim analysis of severe COVID-19 once there had been 100 cases of severe COVID-19. For full details, refer to section 11.4 of the trial protocol.

For full details refer to section 11.1 of the trial protocol. The analysis plan for the pre-planned meta-analysis is detailed in section 9.

CEASATION OF ENROLMENT AND SUBSEQUENT EFFICACY INTERIM ANALYSIS

Recruitment into the BRACE trial was stopped prematurely on 1st April 2021, after 3,988 participants had been recruited into Stage 2, 6,285 overall (including 2,840 recruited in Stage 1). The main reason for stopping before reaching the calculated sample of 10,078 (2,834 planned for Stage 1 + 7,244 planned for Stage 2) was the rollout of COVID-19-specific vaccines in healthcare workers around the world, which started in December 2020. The availability of COVID-19-specific vaccines decreased the interest for potential participants to be recruited into the study, and the receipt of COVID-19 specific vaccines by participants affects the ability of the trial to determine the effectiveness of BCG vaccination in protecting against COVID-19. With 3,988 participants randomised into Stage 2 of the trial, there would be 63.3% power to identify an absolute reduction of 1.30% (relative reduction of 1/3, the effect size used to power Stage 2 of the trial) in the incidence of severe COVID-19 at the end of the trial, from an incidence of 4% at 6 months in the control group to 2.67% in the intervention group (the assumptions used in the sample size calculation for Stage 2) based on a two-sided test with alpha = 0.045, if no interim analysis were planned.

At the time recruitment was stopped, we were monitoring the occurrence of severe COVID-19 cases in preparation for the interim analysis which was scheduled to occur once 100 severe cases was reached (refer to section 11.4 of the protocol). For reasons described in detail in the Interim analysis SAP (https://doi.org/10.25374/MCRI.14721309.v1), we decided to revise the stopping rule to be used in the interim analysis of severe COVID-19 to an alpha spending function, where the threshold to identify efficacy is based on the amount of data available at the time of the interim analysis.

The interim analysis included only exposure time before any dose of any COVID-19-specific vaccine. Given that it is unknown what effect a COVID-19-specific vaccine will have on the effectiveness of the BCG vaccination (or vice versa), participants were censored at the time of their first COVID-19-specific vaccine.

The database lock for the interim analysis happened on the 30th April 2021 (30 day after recruitment was ceased). Up to the this date, 82.3% of participants had received a COVID-19-specific vaccine, or had reported an episode of severe COVID-19, and/or had been followed for at least 6 months from randomisation. Using an alpha-spending function based on the Pocock stopping rule[1], an interim analysis conducted on 82.3% of the available information on 3988 participants, and an overall alpha of 0.045 for this outcome, results in a nominal alpha of 0.04 at the interim analysis, and 0.021 at the end of the study (calculated using a Group Sequential Test (GST) of Two Proportions in NQuery (PTT12-1)). Thus, the threshold of 0.04 was used as the stopping rule for the interim analysis. At the interim time point we had 52.9% power to detect a risk ratio of 0.67 in the incidence of severe COVID-19 at 6 months. Under this spending function we will have 0.021 alpha left for the final analysis given that the treatment comparison did not reach the threshold at the interim analysis.

The details of how the interim analysis was conducted and presented are provided in the Interim analysis SAP (https://doi.org/10.25374/MCRI.14721309.v1).

2.5. STUDY PROCEDURE

Figure 1 and Table 1 provide a summary of the study procedures for the BRACE trial.

Figure 1. Trial timeline

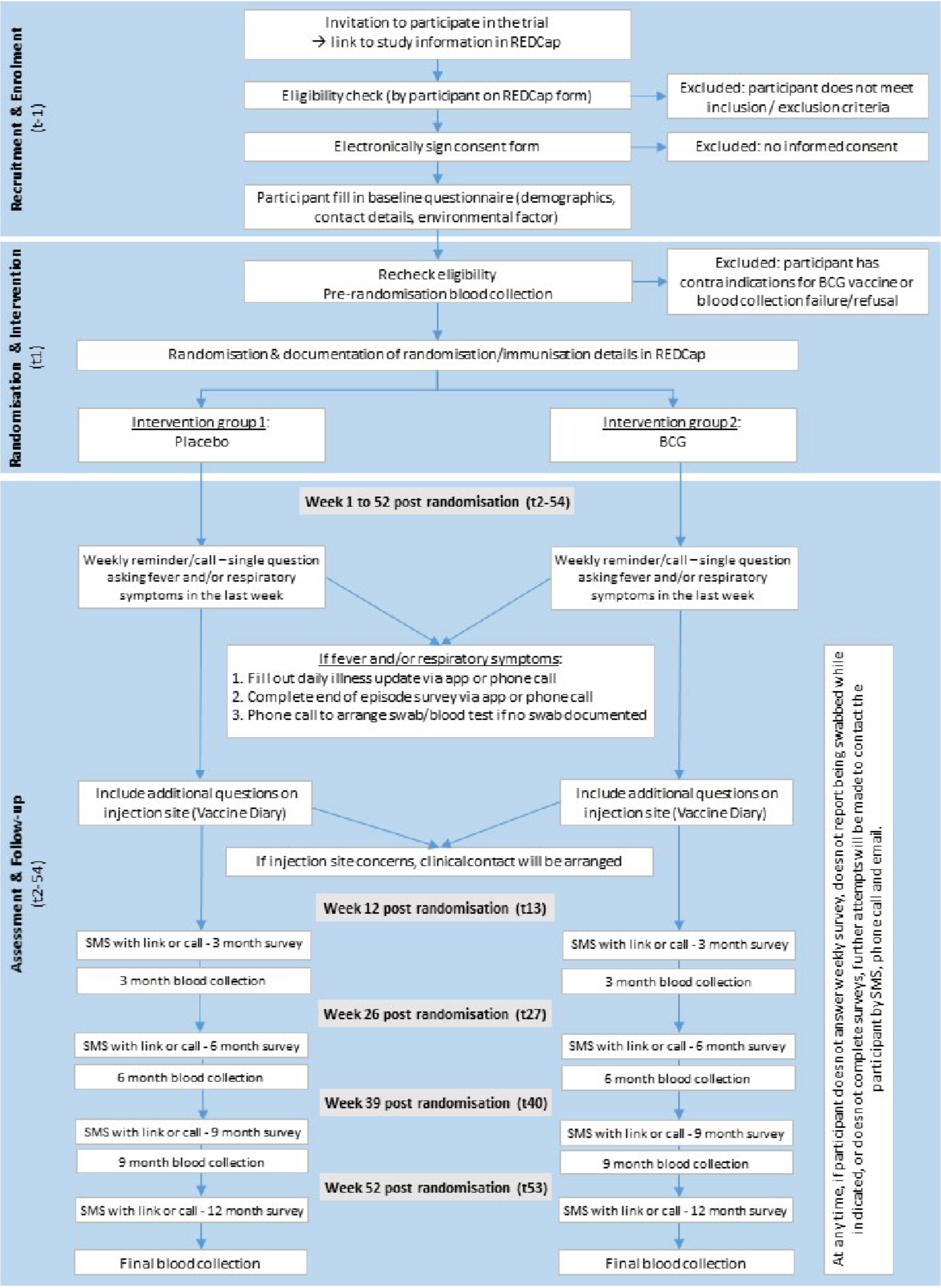

Table 1. Trial timeline

	TRIAL PERIOD									
	Pre-study	Inclusion & randomisation	Post-randomisation							
TIME POINT	t_{-1}	t_0	t_{1-12}	t_{13}	t_{14-25}	t_{26}	t_{27-38}	t_{39}	t_{40-51}	t_{52}
RECRUITMENT:										
Eligibility screen	X									
Informed consent	X									
Contact details	X									
Allocation to intervention		X								
INTERVENTIONS:										
BCG vaccine		X (BCG group)								
Saline injection		X (Placebo group)								
ASSESSMENTS:										
Baseline questionnaire	X	X								
Weekly survey			X	X	X	X	X	X	X	X
Instruction for swab testing (if indicated by weekly survey)			(X)	(X)	(X)	(X)	(X)	(X)	(X)	(X)
3-month survey				X						
6-month survey						X				
9-month survey								X		
12-month survey										X
*Clinical advice on injection site **			X	X						
*Blood collection***		X		X		X		X#		X#
*Baseline SARS-CoV-2 Test ****		X								

T=week (e.g. t1=first week). A 42day window period is accepted for the periodic survey and the blood collection timepoints
* In indicated Infectious Diseases clinician, or state-based organisation, as appropriate
** Optional consent for additional biological sample including blood sample when illness reported
*** Brazil only as outlined in Appendix 4
# Sub-set of participants

3. POPULATIONS OF ANALYSIS

Intention-To-Treat Population

The intention-to-treat (ITT) population will be used for the secondary analysis of efficacy outcomes, with all participants analysed according to the study group to which they were randomly allocated, regardless of the intervention they received. The only participants excluded from this population will be participants who were randomised in error, i.e., assigned by mistake to one of the two allocations even though they were not eligible for the study.

Modified Intention-To-Treat Population (mITT)

The primary population for all efficacy analyses will be the modified intention-to-treat population (mITT) which will only include participants who had a negative SARS-CoV-2 test result at time of randomisation. The mITT population is the same as the ITT population but will also EXCLUDE:

- participants with positive or missing or indeterminant serology at the time of randomisation
- (Applicable only to participants at the Brazilian sites) participant with positive/missing/indeterminant PCR on a respiratory sample or a positive/missing/indeterminant SARS-CoV-2 diagnostic antigen test approved by the local jurisdiction's public health policy at the time of randomisation

Safety Population
The primary population for all safety analyses will be the safety population, which will include all randomised participants who received the vaccine (either BCG or placebo), with all participants analysed according to the intervention they received, irrespective of which group they were randomised to.

4. OUTCOME VARIABLES

4.1. DATA COLLECTION

Participants are asked weekly to report whether they have had any of the following:

1. “Trigger symptoms”, namely:
   - fever (self-reported, defined as temperature > 38 degrees centigrade)
   - intermittent cough
   - persistence cough
   - shortness of breath or difficulty breathing
   - sore throat

2. “Other symptoms”, namely:
   - runny/blocked nose
   - headache
   - muscle and/or joint ache
   - fatigue
   - nausea, vomiting and/or diarrhea
   - loss of taste and/or smell

These weekly reports are collected via a smartphone app (or in the case of Brazil via a weekly phone call) and are collected daily when they are ill (or in the case of Brazil via a phone call every 3 days) (see below). The information collected via the app/phone calls is subsequently confirmed through quarterly questionnaires, at 3, 6, 9 and 12 months post randomisation.

As soon as a participant reports:
- a “trigger symptom”

or
- being non-ambulant for ≥ 3 consecutive days or unable to work for ≥ 3 consecutive days irrespective of symptoms (any episode of illness characterised by ≥ 3 consecutive days or unable to work for ≥ 3 consecutive days is defined a severe episode of illness)

the participant is prompted to have a SARS-CoV-2 test and continues reporting their symptoms on a daily basis until resolution.

Once recovered from the illness, i.e., on the first day with no symptoms, the participant is required to complete an illness resolution form, documenting whether the SARS-CoV-2 test(s) was positive or negative. In circumstances where the participant does not recover from the illness this is entered by the study site coordinator or MCRI data team.

SARS-CoV-2 tests (date, type and results), as well as COVID-19-specific vaccination (date and brand), are also collected via the smartphone app, phone calls, and the quarterly questionnaires.

Quarterly questionnaires at 3, 6, 9 and 12 months from randomisation collect data on hospitalisations and absenteeism. When a participant is hospitalised due to a respiratory or febrile illness, the site coordinator or safety medical doctor complete a hospitalisation form collecting data on diagnosis, treatment and outcome of the hospitalisation. Information on the need for oxygen, mechanical ventilation, admission to critical care/ICU are also collected. Medical records are used by the local teams to complete the hospitalisation forms. Quarterly questionnaires also summarise all the episodes of illness that the participant has entered in the smartphone app

in the prior quarter and ask the participant to confirm those listed, edit them if needed, and retrospectively add episodes of illness that participant may have forgotten to enter into the smartphone app.

Blood samples are collected at baseline, 3-months, 6-months, 9-months and 12 months from randomisation, to assess SARS-CoV-2 serology.

4.2. PRIMARY OUTCOMES

1	Symptomatic COVID-19 by 6 months following randomisation defined as:<br>- positive SARS-CoV-2 test (PCR, antigen or serology), PLUS<br>- [fever (using self-reported questionnaire), OR<br>- at least one 'trigger' symptom of respiratory disease including cough, sore throat, shortness of breath, respiratory distress/failure (using self-reported questionnaire)]
2	Severe COVID-19 by 6 months following randomisation defined as:<br>- positive SARS-CoV-2 test (PCR, antigen or serology), PLUS<br>- [Death as a consequence of COVID-19, OR<br>- Hospitalised as a consequence of COVID-19, OR<br>- Non-hospitalised severe disease as a consequence of COVID-19, defined as non-ambulant[1] for ≥ 3 consecutive days or unable to work[2] for ≥ 3 consecutive days]<br><br>[1] *"pretty much confined to bed (meaning finding it very difficult to do any normal daily activities"*<br><br>[2] *"I do not feel physically well enough to go to work"*

The date of occurrence for either a symptomatic COVID-19 episode (primary outcome 1) or a severe COVID-19 episode (primary outcome 2) will be defined as the first date of any symptom onset for the episode ('trigger' or 'other' symptom, as listed in section 4.1). This is to account for any potential difference between groups in time to SARS-CoV-2 testing from symptom onset.

Excluded episodes

An <u>episode of illness with trigger symptom</u>(s) for which a SARS-CoV-2 testing was not medically indicated (e.g. sore throat due to a known allergy), will not be considered as an episode of illness in the interest of the calculation of primary outcome 1. A <u>severe episode of illness</u> for which a SARS-CoV-2 testing was not medically indicated (e.g. unable to work for ≥ 3 consecutive days due to sprained ankle), will not be considered as an episode of illness in the interest of the calculation of primary outcome 2.

DEFINITION OF SARS-COV-2 TEST

SARS-CoV-2 tests comprise:

- A PCR test, and/or
- A rapid antigen test (RAT), and/or
- Seroconversion determined using the serology results prior to and after the onset of symptoms for an episode of illness (see Figure 3).

DEFINITION OF COVID-19 EPISODES

An episode of illness (either with trigger symptoms or severe episode) will be considered to have an associated COVID-19 test if the episode has a RAT or PCR test or serology data as follows:

PCR/RAT tests – The results of PCR and/or RAT tests will be used for the determination of the COVID-19 episode (either symptomatic or severe) as detailed in Figure 2 using the following testing windows:

PCR: ≤ 3 days prior to onset of symptoms to ≤ 21 days after the onset of symptoms, *or*
≤ 7 days from the last day of symptoms

RAT: ≤ 3 days prior to onset of symptoms to ≤ 10 days after the onset of symptoms

Figure 2 details how results of the RAT/PCT tests will be interpreted.

Figure 2. RAT/PCR testing interpretation

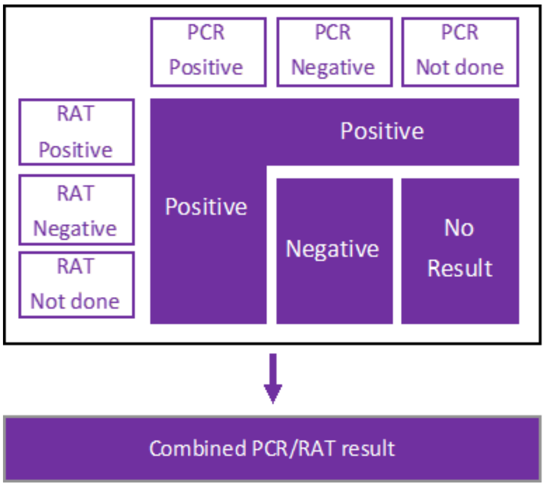

PCR/RAT taken within their testing window

Serology - The results of a serology will also be used for the determination of the COVID-19 episode if both pre-episode and post-episode blood samples results are available. The algorithm in Figure 3 details how serology results will be interpreted.

Figure 3. SARS-CoV-2 Serology Interpretation

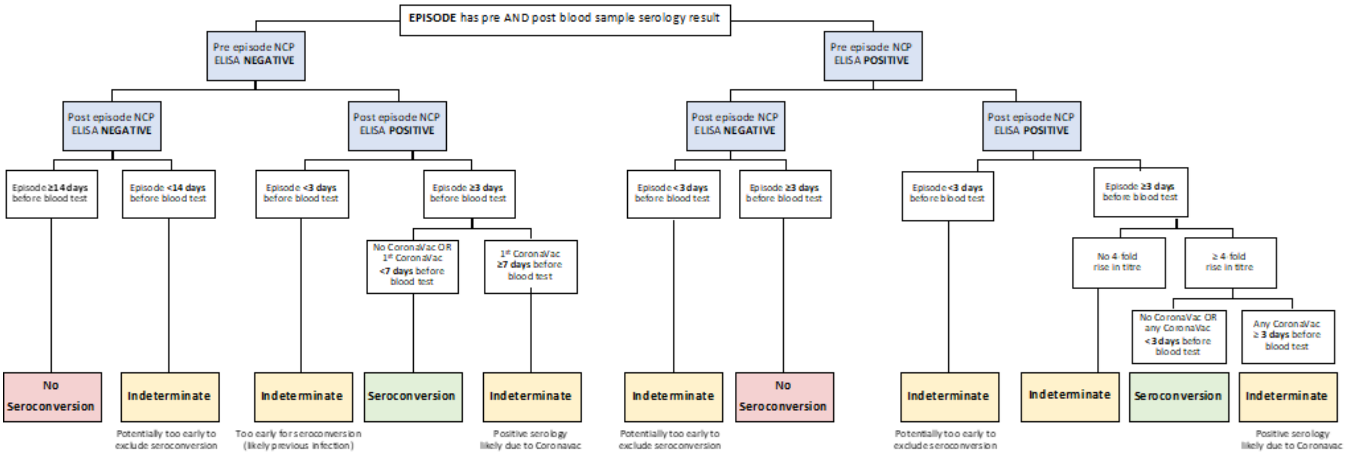

Source: SARS-CoV-2 serology interpretation, version 20-Apr-2022
NCP=nucleocapsid protein

The combination of the results from RAT/PCR tests and/or serology will be interpreted using the algorithm in Figure 4 to categorise episode with trigger symptoms/severe episodes to: COVID-19 episodes (either symptomatic or severe), non-COVID-19 episodes, or episodes with missing information.

Figure 4. COVID-19 episode interpretation

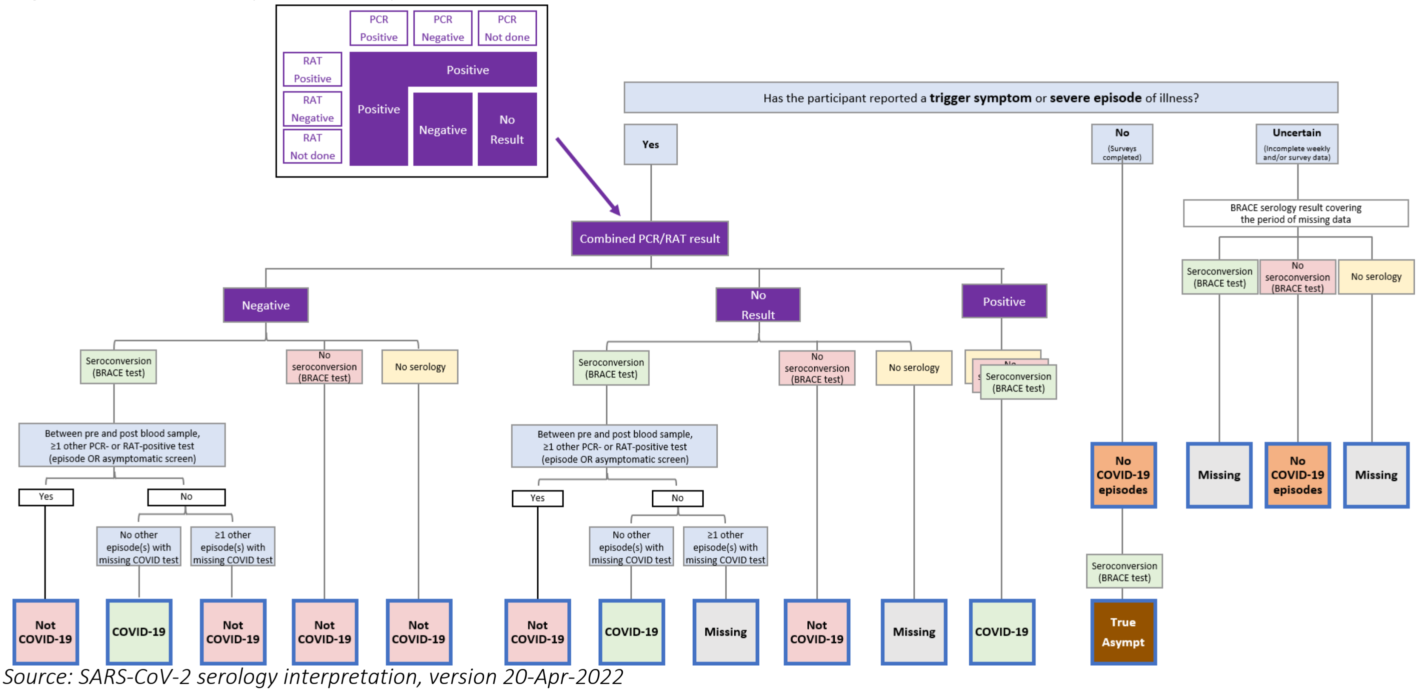

Source: SARS-CoV-2 serology interpretation, version 20-Apr-2022
Asympt COVID-19 = Asymptomatic SARS-CoV-2 infection, as per definition provided in section 4.3, outcome 7a and 7b.

Additionally, each episode of illness (either with trigger symptoms or severe episode) will be categorised as COVID-19 episodes (either symptomatic or severe), non-COVID-19 episodes, or episodes with missing information ignoring serology, i.e. only using data from RAT and PCR tests (Figure 2). This will be used as part of a sensitivity analysis (see session “Sensitivity Analysis 2”).

4.3. SECONDARY OUTCOMES

#	COVID-19 Related Outcomes
3	Symptomatic COVID-19 by 12 months following randomisation as defined for primary outcome 1.<br><br>CALCULATION<br>As per primary outcome 1 but over 12 months.
4	Severe COVID-19 by 12 months following randomisation as defined for primary outcome 2.<br><br>CALCULATION<br>As per primary outcome 1 but over 12 months.
5a	Time to first symptom of COVID-19 over the 6 months following randomisation.<br><br>CALCULATION<br>Participants who had either a symptomatic or severe COVID-19 episode will have time to first symptom of COVID-19 calculated as:<br><br>*date of any symptom onset for the first symptomatic or severe COVID-19 episode – date of randomisation*<br><br>Participants who have not had a symptomatic or severe COVID-19 episode will have time calculated as:<br>*Earliest censoring date*– date of randomisation*

#	COVID-19 Related Outcomes
	For participants who experience more than one COVID-19 episode, the time to the first of these episodes will be used for the analysis.<br><br>**as defined in section 6.2.3*
5b	Time to first symptom of COVID-19 over the 12 months following randomisation.<br><br>CALCULATION<br>As per 5a but over 12 months.
6a	Number of episodes of COVID-19 by 6 months.<br><br>CALCULATION<br>The total number of symptomatic or severe COVID-19 episodes (refer to section 4.2 for their definition) by 6 months will be calculated for each participant.<br>Participants who have had neither a symptomatic nor a severe COVID-19 episode by 6 months will be considered to have had 0 (zero) episodes of COVID-19.<br>Two COVID-19 episodes of illness are considered distinct if they are >10 days apart.
6b	Number of episodes of COVID-19 by 12 months.<br><br>CALCULATION<br>As 6a but over 12 months.
7a	Asymptomatic SARS-CoV-2 infection by 6 months<br><br>Asymptomatic SARS-CoV-2 infection will be defined as<br>- Evidence of SARS-CoV-2 infection (by seroconversion) and<br>- Absence of any episodes of illness (defined by trigger or non-trigger symptoms) (using self-reported questionnaire) and<br>- No evidence of exposure prior to randomisation<br><br>CALCULATION<br>Participants will be considered to have met this outcome if by 6 months:<br>- they seroconverted to SARS-CoV-2, AND<br>- they did not receive CoronaVac* during the seroconversion period (i.e., first CoronaVac* ≥ 7 days before blood test) that could explain the seroconversion, AND<br>- they have complete diary data or survey data up to the earlier of 6 months or the date of the blood draw at which they became seropositive which confirm they did not have any episodes of illness<br><br>Participants will be considered to NOT have met this outcome if they:<br>- have complete diary data or survey data up to 6 months AND<br>- do not show evidence of seroconversion to SARS-CoV-2 at 6 months (i.e., serology result at 3 months is not positive and at 6 months is negative) AND<br>- reported no episodes of illness over the 6 months following randomisation that tested positive to PCR or RAT<br>OR<br>- have complete diary data or survey data up to 6 months AND<br>- show evidence of seroconversion to SARS-CoV-2 at 6 months (i.e., serology result at 3 or 6 months is positive) AND<br>- reported any episode of illness that could have accounted for the seroconversion (experienced either an episode which tested positive to PCR/RAT tests or an episode that was not tested for COVID-19)<br><br>* administration of CoronaVac will be handled in the analysis in line with the Principal Stratum Strategy (refer to section 5.5 and 7.4).

#	COVID-19 Related Outcomes
7b	Asymptomatic SARS-CoV-2 infection over 12 months.<br><br>CALCULATION<br>As per 7a but over 12 months.
8a	Number of days unable to work due to COVID-19 within 6 months following randomisation (excludes quarantine/workplace restrictions)<br><br>CALCULATION<br>This is a count of the days marked “unable to work” across all COVID-19 episodes (either symptomatic or severe, as defined in section 4.2) by 6 months post randomisation. Participants who have not had a symptomatic or severe COVID-19 episode during this period will be regarded as having had 0 (zero) days unable to work due to COVID-19.
8b	Number of days unable to work due to COVID-19 within 12 months following randomisation (excludes quarantine/workplace restrictions)<br><br>CALCULATION<br>As for outcome 8a but over 12 months.
9a	Number of days confined to bed due to COVID-19 within 6 months following randomisation.<br><br>CALCULATION<br>This is a count of the days marked “confined to bed” across all COVID-19 episodes (either symptomatic or severe, as defined in section 4.2) by 6 months post randomisation. Participants who have not had a symptomatic or severe COVID-19 episode during this period will be regarded as having had 0 (zero) days confined to bed due to COVID-19.
9b	Number of days confined to bed due to COVID-19 within 12 months following randomisation.<br><br>CALCULATION<br>As for outcome 9a but over 12 months.
10a	Number of days with symptoms due to COVID-19 within 6 months following randomisation.<br><br>CALCULATION<br>The number of days with symptoms for a given COVID-19 episode is the number of days from the start to the end of the COVID-19 episode (either symptomatic or severe, as defined in section 4.2) using this formula:<br>*Episode duration = Episode end date* – episode start date*<br>** defined as first day with no symptoms*<br><br>For participants who die as a consequence of COVID-19, the end date will be the date of death.<br>For participants who had multiple COVID-19 episodes within 6 months, the number of days with symptoms due to COVID-19 will be the sum of durations across all the COVID-19 episodes within the 6 months following randomisation.<br>Participants who have not had a symptomatic or severe COVID-19 episode within 6 months of randomisation will be regarded as having had 0 (zero) days with symptoms due to COVID-19.
10b	Number of days with symptoms due to COVID-19 within 12 months following randomisation.<br><br>CALCULATION<br>As for outcome 10a but over 12 months.
11a	Pneumonia due to COVID-19 by 6 months.<br><br>CALCULATION

#	COVID-19 Related Outcomes
	The derivation of this outcome will use data from the self-reported questionnaire and/or hospitalisation forms.<br>Participants will be coded as having had the outcome if:<br>- They developed pneumonia during a symptomatic or a severe COVID-19 episode within 6 months of randomisation<br>Participants will be coded as not having had this outcome if:<br>- They did not develop pneumonia during a symptomatic or a severe COVID-19 episode or<br>- They did not have a symptomatic or a severe COVID-19 episode within 6 months of randomisation.
11b	Pneumonia due to COVID-19 by 12 months.<br><br>CALCULATION<br>As for outcome 11a but over 12 months.
12a	Need for oxygen therapy due to COVID-19 by 6 months.<br><br>CALCULATION<br>The derivation of this outcome will use data from the self-reported questionnaire and/or hospitalisation forms.<br>Participants will be coded having had the outcome if:<br>- They needed oxygen therapy during a severe COVID-19 episode within 6 months of randomisation<br>Participants will be coded not having had the outcome if:<br>- They did not need oxygen therapy during a severe COVID-19 episode or<br>- They did not have a severe COVID-19 episode within 6 months of randomisation
12b	Need for oxygen therapy due to COVID-19 by 12 months.<br><br>CALCULATION<br>As for outcome 12a but over a 12 month period.

13a	Admission to critical care and duration of stay due to COVID-19 by 6 months.<br><br>CALCULATION<br>The derivation of both outcomes will use data from the self-reported questionnaire and/or hospitalisation forms.<br>– Admission to critical care (including ICU)<br>Participants will be coded as having had at least one admission to critical care/ICU due to COVID-19 if:<br>- They were admitted to critical care/ICU during a severe COVID-19 episode within 6 months of randomisation<br>Participants will be coded as not having had this outcome if:<br>- They were NOT hospitalised during a severe COVID-19 episode or<br>- They were NOT admitted to critical care/ICU during a hospitalisation for a severe COVID-19 episode or<br>- They did not have a severe COVID-19 episode within 6 months of randomisation<br><br>– Duration of stay<br>For participants who were admitted to critical care/ICU during a severe COVID-19 episode within 6 months of randomisation, we will also calculate the duration of stay as the difference between the date of admission and date of discharge. For participants who had multiple COVID-19 episodes with admission to critical care within 6 months of randomisation, this number will be the sum of durations across all critical care stays.
13b	Admission to critical care and duration of stay due to COVID-19 by 12 months.<br><br>CALCULATION<br>As for outcome 13a but over 12 months.
14a	Need of mechanical ventilation (MV) and duration of MV due to COVID-19 by 6 months.<br><br>CALCULATION<br>The derivation of both outcomes will use data from the self-reported questionnaire and/or hospitalisation forms.<br>– Need of MV<br>Participants will be coded as having been in need of MV due to SARS-CoV-2 if:<br>- They needed MV during a severe COVID-19 episode within 6 months of randomisation<br>Participants will be coded as not having had this outcome if:<br>- They were NOT hospitalised during a severe COVID-19 episode or<br>- They did NOT need MV during a hospitalisation for a severe COVID-19 episode or<br>- They did not have a severe COVID-19 episode within 6 months of randomisation<br><br>– Duration of MV<br>For participants who needed mechanical ventilation within 6 months of randomisation, we will also calculate the duration of MV as the difference between the date/time MV was started and the date/time MV was stopped. For participants who had multiple COVID-19 episodes requiring MV within 6 months of randomisation V, this number will be the sum of durations across all COVID-19 episodes requiring MV.
14b	Need of mechanical ventilation and duration of MV due to COVID-19 by 12 months.<br><br>CALCULATION<br>As for outcome 14a but over 12 months.
15a	Hospitalisation due to COVID-19 (using self-reported questionnaire and/or medical/hospital records) and duration of hospitalisation by 6 months.<br><br>CALCULATION<br>– Hospitalisation due to COVID-19<br>Participants will be coded as having been hospitalised due to COVID-19 if:<br>- They had severe COVID-19 (primary outcome 2) and were hospitalised as a consequence of COVID-19 within 6 months of randomisation

	Participants will be coded as not having had the outcome if:<br>- They had severe COVID-19 (primary outcome 2) and were NOT hospitalised as a consequence of COVID-19<br>- They did not have severe COVID-19 (did not meet the definition of either the primary outcome 2) within 6 months of randomisation<br><br>– Duration of hospitalisation<br>For participants who were hospitalised due to COVID-19 within 6 months of randomisation, the duration of their hospital stay due to COVID-19 will be calculated as the difference between date of admission and date of discharge. For participants who had multiple severe COVID-19 episodes resulting in hospitalisation within 6 months of randomisation, this number will be the sum of durations across all hospitalisations due to COVID-19.
15b	Hospitalisation due to COVID-19 (using self-reported questionnaire and/or medical/hospital records) and duration of hospitalisation by 12 months.<br><br>CALCULATION<br>As for outcome 15a but over 12 months.
16a	Death due to COVID-19 by 6 months.<br><br>CALCULATION<br>Participants will be coded as having had the outcome if:<br>- They had severe COVID-19 (primary outcome 2) and died as a consequence of COVID-19 within 6 months of randomisation<br>Participants will be coded as not having had the outcome if:<br>- They had severe COVID-19 (primary outcome 2) and did NOT die or<br>- They did not have severe COVID-19 or<br>- They died for other reasons (not as a consequence of COVID-19) within 6 months of randomisation
16b	Death due to COVID-19 by 12 months.<br><br>CALCULATION<br>As for outcome 16a but over 12 months.

#	Other Outcomes – NON-COVID-19 Related Outcomes
17	Fever or respiratory illness*(using self-reported questionnaire), over the 12 months following randomisation.<br><br>* Respiratory illness will be defined as:<br>at least one sign or symptom of respiratory disease including cough, sore throat, shortness of breath, respiratory distress/failure, or runny/blocked nose (in combination with another respiratory symptom or fever).<br><br>Fever due to reactions to any vaccine will not be considered for this outcome.<br><br>CALCULATION<br>Participants will be coded as having had the outcome if:<br>- They developed an episode of illness with fever (self-reported, defined as temperature > 38 degrees) or respiratory illness (including due to COVID-19) within 12 months of randomisation<br>Participants will be coded as not having had the outcome if:<br>- They did not have any episodes of fever or respiratory illness within 12 months of randomisation

#	Other Outcomes – NON-COVID-19 Related Outcomes
18	Severe fever or respiratory illness*(using self-reported questionnaire), over the 12 months following randomisation, defined as:<br>- Death, OR<br>- Hospitalised, OR<br>- Non-hospitalised severe disease, defined as non-ambulant1 for ≥ 3 consecutive days orunable to work 2 for ≥ 3 consecutive days<br>as a consequence of fever or respiratory illness, as defined above (outcome 17)<br><br>[1] "pretty much confined to bed (meaning finding it very difficult to do any normal daily activities"<br>[2] "I do not feel physically well enough to go to work" (excludes stay at home exclusively for quarantine/workplace restrictions)<br>Fever due to reactions to any vaccine will not be considered for this outcome.<br><br>CALCULATION<br>Participants will be coded as having had the outcome if:<br>- They developed a severe episode of illness with fever (self-reported, defined as temperature > 38 degrees) or respiratory illness (including due to COVID-19) within 12 months of randomisation<br>Participants will be coded as not having had the outcome if:<br>- They did not have any severe episodes of fever or respiratory illness within 12 months of randomisation
19	Number of episodes of fever or respiratory illness (as defined in outcome 17), over the 12 months following randomisation.<br>CALCULATION<br>This will reflect the number of distinct episodes* of fever or respiratory illness within 12 months of randomisation. Participants who have not experienced any episodes of fever or respiratory illness within 12 months of randomisation will be considered to have had 0 (zero) episodes.
20	Number of days unable to work (using self-reported questionnaire) due to fever or respiratory illness (as defined in outcome 17), over the 12 months following randomisation (excludes quarantine/workplace restrictions)<br>CALCULATION<br>This will be calculated as the sum of the days marked as unable to work due to fever or respiratory illness within 12 months of randomisation. Participants who have not experienced any episodes of fever or respiratory illness within 12 months of randomisation will be considered to have had 0 (zero) episodes.
21	Number of days confined to bed (using self-reported questionnaire) due to fever or respiratory illness, illness (as defined in outcome 17), over the 12 months following randomisation<br>CALCULATION<br>This will be calculated as the sum of the days marked as confined to bed to fever or respiratory illness within 12 months of randomisation. Participants who have not experienced any episodes of fever or respiratory illness will be considered to have had 0 (zero) episodes within 12 months of randomisation.
22	Number of days with symptoms due to fever or respiratory illness (as defined in outcome 17), over the 12 months following randomisation<br>CALCULATION<br>For each episode of fever or respiratory illness the number of days from the start to the end dates of the episode of fever or respiratory illness will be calculated using this formula:<br>*Episode duration = Episode end date* – episode start date*<br>** defined as first day with no symptoms*

#	Other Outcomes – NON-COVID-19 Related Outcomes
	For participants who are hospitalised due to fever or respiratory illness, the end date will be the date of discharge from the hospital.<br>For participants who die as a consequence of fever or respiratory illness, the end date will be the date of death.<br>For participants who had multiple episodes of fever or respiratory illness within 12 months of randomisation, this number will be the sum of durations across all the episodes.<br>Participants who have not had an episode fever or respiratory illness within 12 months of randomisation will be regarded as having had 0 (zero) days with symptoms due to fever or respiratory illness.
23	Pneumonia within a febrile or respiratory illness over the 12 months following randomisation<br><br>CALCULATION<br>Data from the self-reported questionnaire and/or hospitalisation forms will be used to derive this outcome.<br>Participants will be coded as having had the outcome if:<br>- They developed pneumonia during an episode of fever or respiratory illness within 12 months of randomisation<br>Participants will be coded as not having had the outcome if:<br>- They did not developed pneumonia during a episode of fever or respiratory illness or<br>- They did not have any episodes of fever or respiratory illness<br>- within 12 months of randomisation
24	Need for oxygen therapy for a febrile or respiratory illness over the 12 months following randomisation<br><br>CALCULATION<br>The derivation of this outcome will use data from the self-reported questionnaire and/or hospitalisation forms.<br>Participants will be coded having had the outcome if:<br>- They needed oxygen therapy during an episode of fever or respiratory illness within 12 months of randomisation<br>Participants will be coded not having had the outcome if:<br>- They did not need oxygen therapy during an episode of fever or respiratory illness<br>- They did not have an episode of fever or respiratory illness within 12 months of randomisation
25	Admission to critical care for a febrile or respiratory illness (using self-reported questionnaire and/or medical/hospital records), over the 12 months following randomisation<br><br>Admission to critical care following elective intervention will not be counted as part of this outcome.<br><br>CALCULATION<br>The derivation of this outcome will use data from the self-reported questionnaire and/or hospitalisation forms.<br>Participants will be coded as having had the outcome if:<br>- They were admitted to critical care following a febrile or respiratory illness within 12 months of randomisation<br>Participants will be coded as not having had the outcome if:<br>- They were NOT hospitalised during any episode of fever or respiratory illness<br>- They were NOT admitted to critical care during any hospitalised episode of fever or respiratory illness<br>- They did not have an episode of fever or respiratory illness within 12 months ofrandomisation
26	Need for mechanical ventilation (MV) for a febrile or respiratory illness (using self-reported questionnaire and/or medical/hospital records), over the 12 months following randomisation<br><br>MV required as part of an elective intervention will not be counted as part of this outcome.<br><br>CALCULATION

#	Other Outcomes – NON-COVID-19 Related Outcomes
	The derivation of this outcome will use data from the self-reported questionnaire and/or hospitalisation forms.<br>Participants will be coded as having had the outcome if:<br>- They needed MV during an episode of fever or respiratory illness within 12 months of randomisation<br>Participants will be coded as not having had the outcome if:<br>- They were NOT hospitalised during any episode of fever or respiratory illness<br>- They did NOT need MV during a hospitalisation for an episode of fever or respiratory illness<br>- They did not have an episode of fever or respiratory illness<br>- within 12 months of randomisation
27	Deaths as a consequence of an episode of fever or respiratory illness over the 12 months following randomisation<br><br>CALCULATION<br>Participants will be coded as having had the outcome if:<br>- They died as a consequence of an episode of fever or respiratory illness within 12 months of randomisation<br>Participants will be coded as not having had the outcome if:<br>- They had an episode of fever or respiratory illness and did NOT die<br>- They did not have an episode of fever or respiratory illness<br>- They died for other reasons (not as a consequence of an episode of fever or respiratory illness)<br>- within 12 months of randomisation
28	Hospitalisation for a febrile or respiratory illness and duration of hospitalisation over the 12 months following randomisation.<br><br>CALCULATION<br>The derivation of this outcome will use data from the self-reported questionnaire and/or hospitalisation forms.<br>On participants who were hospitalised due to an episode of fever or respiratory illness, the duration of their hospital stay will be calculated as the difference between date of admission and date of discharge. If a participant experienced >1 episodes of fever or respiratory illness which resulted in hospitalisation within 12 months of randomisation, all the episodes will be included in this analysis, and the overall duration of hospitalisation will be the sum of the duration of each hospitalisation.<br>Participants were hospitalised as a consequence of an episode of fever or respiratory illness within 12 months of randomisation will have their duration of hospitalisation equal to zero
29a	Number of days of unplanned absenteeism for an acute illness or hospitalisation over the 6 months following randomisation.<br><br>CALCULATION<br>The number of days the participant reported unplanned absenteeism (using self-reported questionnaire) for an acute illness or hospitalisation within 6 months of randomisation will be calculated. This will exclude absenteeism for other reason such as elective hospitalisation, issues with vaccination site, mandatory quarantine while not ill, carer leave, annual leave/holidays/planned absence, or pregnancy-related absence.<br>The number of days of unplanned absenteeism for an acute illness or hospitalisation within 6 months of randomisation will be set to missing for participants who don't complete the 6-month self-reported questionnaire.
29b	Number of days of unplanned absenteeism for any reason (using self-reported questionnaire) over the 12 months following randomisation.<br><br>CALCULATION<br>As 28a.

#	Other Outcomes – NON-COVID-19 Related Outcomes
30	Adverse events (AEs) experienced by the participant over the 3 months following randomisation, by type, severity (graded using toxicity grading scale), relationship to intervention of adverse events (AEs) of interest*.<br><br>* AEs of interest are defined as:<br>- Reaction at injection site (pain, tenderness, redness, swelling) of grade 3 (severe) or 4 (potentially life threatening)<br>- Abscess at injection site<br>- Large ulcer (>1.5 cm diameter) at injection site<br>- Keloid scar at injection site<br>- Lymphadenopathy (in region of injection site)<br>- BCG osteitis/osteomyelitis<br>- Disseminated BCG infection (BCG-osis)<br>- Allergic reaction due to IP<br>- Fainting episode, seizures and convulsions following IP administration (recorded on the day of IP administration only)
31	Serious Adverse Events (SAEs) experienced by the participant over the 3 months following randomisation.

4.4. OTHER VARIABLES

DEMOGRAPHY AND BASELINE

Baseline characteristics that will be presented include:

- Sex – Male/Female/Other/Declined/ Missing
- Age*, years
- Body Mass Index (BMI), kg/m^2 – < 18.5 / 18.5 to 24.9/ 25 to 29.9 / >30 / Missing
- Department – Emergency / Intensive Care Unit or High Dependency Unit / Operating Theatre / General Ward / Pharmacy / Practice outside of hospital setting/ Other / Missing
- Role – Administrative-clerical staff / Allied Health / Dentist-dental therapy / Doctor / Nurse-Midwife / Patient Services Assistant-hospital maintenance / Scientist (medical research) / Other / Missing
- Contact with patients, hours - <10 / 10-20 / >20 / Missing
- Confirmed cases of COVID-19 within department – Yes / No / Missing
- Smoking – Yes / No / Missing
- Previous BCG vaccination -No / last BCG dose <1 year ago / 1-5 years ago / >5 years ago / Missing
- Evidence of BCG scar at randomisation – Yes / No / Unsure / Missing
- Positive (>5mm) Tuberculin Skin Test or positive Mantoux test in the past? Yes / No / Unsure
- Previous tuberculosis (TB) exposure – Yes / No / Missing
- Positive PCR or SARS-CoV-2 diagnostic antigen test or serology at randomisation– Yes / No / Missing
- Comorbidities
  - Diabetes – Yes / No / Missing
    - Type 1 diabetes
    - Type 2 diabetes
    - Type 1 and type 2 diabetes
    - Other diabetes
    - Missing
  - Cardiovascular disease– Yes / No / Missing
    - Ischaemic heart disease
    - Congestive heart disease
    - Other cardiovascular disease
    - Hypertension– Yes / No / Missing

    - Missing
- Chronic respiratory disease– Yes / No / Missing
- Number of co-morbidities
    - 1
    - 2
    - 3 or more
    - Missing

* Since year of birth rather than DOB is collected for EU participants, age will be calculated using "1-July" in their year of birth as their DOB.

COVID-19-SPECIFIC VACCINES

- Time between randomisation and first COVID-19-specific vaccine dose
- Brand of the first dose of COVID-19-specific vaccine received
    - First and second doses of the same brand
- Number of doses received

OTHER VACCINES

- Time between randomisation and first other vaccine dose
- Type of the first dose of vaccine received
- Number of doses received
- Number of types of vaccine received

PARTICIPANT FOLLOW-UP

- Withdrawal after randomisation
- Reasons for withdrawal.

PROTOCOL DEVIATIONS

- Whether there was a protocol deviation
- Reasons for protocol deviation - Received the opposite intervention/ Did not receive any intervention / Participant randomised twice/ Did not receive the questionnaire in time/ Not able to use the app/ Did not have a swab while indicated / Did not have the blood sample taken / Blood/vaccination not performed on day of randomisation, but later/ Randomised in the wrong strata / Blood sample (3m) taken and re-consent form incomplete / Blood sample taken without correct consent / Blood taken outside window / Blood collection: did not collect the right tube/ Improper preparation of BCG/ Participant received triple the dose of BCG / Problem during blood processing / Delay in delivery of bloods / BCG within 12 months of randomisation / Pregnancy at randomisation / Other
- Whether there was a protocol violation
- Reasons for protocol violations - Participant received twice the dose of BCG / Intravenous injection of BCG / Other

5. STATISTICAL METHODOLOGY

5.1. GENERAL PRINCIPLES

The details of the randomisation groups will be unblinded only once the database has been locked and the SAP has been finalised, approved by the TSC and made publicly available.

Multiple outcomes will be considered in evaluating the effectiveness of the trial intervention. The magnitude of the treatment effect, with 95% confidence interval and p-value, will be estimated for each outcome. Findings will

be interpreted based on the magnitude of the treatment effect and in context of one another rather than in isolation considering the patterns and consistency in the findings across outcomes.

The comparison of BCG vs placebo in Stage 2 participants is the primary analysis of interest. It is planned to combine data from the two stages of the trial (Stage 1 + Stage 2) in a meta-analysis for the secondary analysis of the non-COVID19 outcomes only (see Section 9 for details).

5.2. DEFINITION OF BASELINE

Baseline is defined as time of enrolment, captured by date of randomisation (day 0).

5.3. DEFINITION OF THE 6- and 12-MONTH CUT-OFFS

The 6-month period from randomisation will be defined as the date of randomisation date plus 182 days following randomisation.
The 12-month period from randomisation will be defined as the date of randomisation date plus 365 days following randomisation

5.4. DESCRIPTIVE STATISTICS

5.4.1. PARTICIPANT DISPOSITION

All participants who were invited to participate in the BRACE trial will be accounted for as part of the CONSORT flow diagram. The number of participants that were screened but not randomised will be presented and the reasons for their non-participation will be listed. The number of participants who fulfilled eligibility criteria and were recruited will be presented overall and by study centre. The number and proportion of participants who discontinue the study prematurely and/or withdraw during the study will be presented, and the reasons for early withdrawal will be presented by intervention group (BCG or Placebo).
The number of participants with at least one protocol deviation, the number of protocol deviations per participant and the reasons for protocol deviation will be summarised by intervention group (BCG or Placebo).

5.4.2. PARTICIPANT CHARACTERISTICS

The demographic characteristics at randomisation of the participants in the mITT as well in the ITT and safety populations will be presented for each intervention group (BCG or Placebo) using the mean and standard deviation (SD) or median and interquartile range (IQR) for continuous data and using numbers and proportions for categorical data.

5.5. THE ESTIMAND FRAMEWORK

The estimand is the precise description of the intervention effect of interest for a given objective. The estimand is described by the following attributes:

- Population
- Outcome
- Interventions
- Handling of intercurrent events
- Summary measure

An intercurrent event is one that occurs after randomisation and prior to observation of the trial endpoint (primary or secondary). There are two important intercurrent events within this trial:

- the administration of COVID-19-specific vaccine
- the administration of any vaccine (including influenza vaccine and COVID-19-specific vaccine)

Within the analysis of this trial, we will adopt three strategies to handle the intercurrent events:

- Hypothetical Strategy: the aim of this strategy is to estimate the intervention effect of being offered the intervention in the absence of intercurrent events. This strategy involves considering what would have happened if the participant had not had the intercurrent event.
- Treatment Policy Strategy: the aim of this strategy is to assess the effect of being offered the intervention irrespective of any intercurrent events. In this strategy intercurrent events are ignored and all outcome data are used regardless of occurrence of the intercurrent event.
- Principal stratum strategy: this strategy considers measurements in a subgroup of participants where the intercurrent event(s) is not likely or less likely to occur. This strategy classifies participants according to their potential occurrence of an intercurrent event in both study groups.

The estimands of interest in this trial are outlined in section 6.

5.6. ANALYSIS SOFTWARE

All analyses will be performed using Stata Release 16.1 or later.

6. PRIMARY OUTCOMES

6.1. ESTIMANDS

Objective	Estimand
To determine if BCG vaccination compared with placebo reduces the incidence of symptomatic COVID-19 in the absence of a COVID-19 specific vaccine, over the 6 months following randomisation, in healthcare workers who did not have a previous SARS-CoV-2 positive test result when assessed at time of randomisation.	Estimand 1.1 [Primary analysis]<br><br>Population: mITT population<br>Outcome: symptomatic COVID-19 by 6 months<br>Interventions: BCG vs Placebo<br>Handling of Intercurrent events:<br>- COVID-19 specific vaccine (Hypothetical Strategy)<br>- any other vaccine (Treatment Policy strategy)<br>Summary Measure: Adjusted* difference in proportion of participants
To determine if BCG vaccination compared with placebo reduces the incidence of severe COVID-19 in the absence of a COVID-19 specific vaccine, over the 6 months following randomisation, in healthcare workers who did not have a previous SARS-CoV-2 positive test result when assessed at time of randomisation.	Estimand 2.1 [Primary analysis]<br><br>Population: mITT population<br>Outcome: severe COVID-19 by 6 months<br>Interventions: BCG vs Placebo<br>Handling of Intercurrent events:<br>- COVID-19 specific vaccine (Hypothetical Strategy)<br>- any other vaccine (Treatment Policy strategy)<br>Summary Measure: Adjusted* difference in proportion of participants
** 1) adjusted for stratification factors used at randomisation (age group, presence of comorbidity, and geographical location); 2) adjusted for stratification factors used at randomisation + sex, BMI at baseline, BCG vaccine before randomisation;*	

Analytical approach for the primary estimands

For the primary analysis of each of the primary outcomes, receiving a COVID-19 vaccine will be handled using a hypothetical strategy; participants who receive a COVID-19-specific vaccine will have their data used up to the date of their first dose of COVID-19-specific vaccine (data collected after the COVID-19-specific vaccine will be ignored). These primary analyses will be conducted on the mITT population including only Stage 2 participants.

6.2. PRIMARY ANALYSIS

6.2.1. SUMMARY STATISTICS

The outcomes of symptomatic COVID-19 and severe COVID-19 prior to 6 months will be described by intervention group as the absolute number of participants with the event. The primary outcome of severe COVID-19 by 6 months will also be presented as the number of participants within 5 categories, according to the most severe event they encountered over the 6-month period, by intervention group:

- Severe COVID-19 which resulted in death
- Severe COVID-19 which resulted in hospitalisation
- Non-hospitalised severe COVID-19
  This category will be further broken down as:
    - Non-hospitalised severe COVID-19 who were confined to bed for 3 consecutive days or more
    - Non-hospitalised severe COVID-19 who were too sick to go to work for 3 consecutive days or more

The numbers of participants whose follow-up data is censored due to:

- missing PCR, RAT or serology test result,
- incomplete data entry,
- drop-out from the study, and
- intercurrent event (COVID-19 specific vaccine / Any other vaccine)

will also be reported separately by intervention group.

6.2.2. ANALYSIS

The outcomes of symptomatic COVID-19 and severe COVID-19 prior to 6 months will be compared between the BCG group and the placebo group recruited in Stage 2 using a difference in proportions. This will be estimated using a time-to-event analysis. The first analysis will be adjusted for stratification factors used in randomisation, namely age group (<40 years; 40 to 59 years; >=60 years), presence of comorbidity (any of diabetes, chronic respiratory disease, cardiovascular disease, hypertension), and geographical location (Europe/Australia/South America). Although randomisation was stratification was by participating hospitals and clinics, for the analysis it was decided to group the sites into the 3 regions due to the high number of randomising sites which could lead to computational problems. For participants who were randomised in the incorrect stratum, the correct stratum will be used as covariate in the model. To do this analysis, the survival curve for each combination of strata and randomised group will be calculated using a flexible parametric survival model (Royston-Parmar model[2]). This will be done using the *stpm2* command in Stata, with the *meansurv* and *timevar* options specified. The average survival curve for each randomised group will be estimated as a weighted average of the corresponding stratum-specific survival curves, with weights proportional to the number of individuals in each stratum in the randomised group at baseline. The parameter of interest will be the (adjusted) point estimate for the difference in proportion with the event at 6 months between BCG and control group. A two-sided bias-corrected 95% CI for the difference in proportion (BCG – Control) will be calculated with bootstrap standard errors using the Stata bootstrap command. A bootstrap p-value will also be calculated. The bootstrapping will sample 1000 times (with replacement) and be stratified by the stratification factors. Note because modelling will be used to estimate the difference in proportion, the results from this analysis will not correspond directly to the raw summaries that will be presented.

A Kaplan-Meier survival curve will also be presented by treatment arm.

The proportional hazards assumption will be checked when running these analyses.

The analysis of both primary outcomes (for all estimands 1.1-6 and 2.1-6) will then be repeated including adjustment for the following baseline covariates which are expected to be associated with COVID-19:

1. Sex (Female / Male)

2. BMI at baseline (< 30 / ≥ 30 kg/m2)
3. BCG vaccination before enrolling into the trial (Yes / No)

Should the fully adjusted models not run when all the all the covariates listed above are included in the model, the covariates will be removed starting from the bottom of the list to the top, until the adjusted model runs.

Handling of missing data
Protocol version 12 stated that, for the primary analysis, multiple imputation (MI) would be used to handle missing data if >10% of the primary outcome data were missing. During the trial it became apparent that a number of participants would have partial data on the primary outcomes due to the expansion of COVID-19 vaccination programs. Therefore, survival analysis will be used to estimate the proportion with symptomatic COVID-19 or severe COVID-19, which enables this partial follow-up data to be included in the analysis. Strategies to handle missingness due to non-testing within trigger and severe episodes is also described in section 6.2.3. Given the survival analysis strategy enables all participants with at least some follow-up data to be included in the analysis, the analysis will be conducted using the available data with no MI.

6.2.3. DATES AND CENSORING FOR 6-MONTH FOLLOW-UP

Censoring dates used for the analysis of the primary outcomes are described in table 2 below.

Table 2. Dates and censoring algorithm

MAIN ANALYSIS	SENSITIVITY ANALYSIS*
Censored at the earlier of:<br>[A] their first COVID-19 specific vaccine dose or<br>[B] day 182 of their participation in the trial or<br>[C] their last entered date prior to which there are more than 3 consecutive days of missing data which aren't ruled out by negative serology or<br>[D] their first day with symptoms for their first episode of illness with trigger/severe symptoms, which the algorithm in Figure 4 cannot ascertain be a COVID-19 episode (categorised as missing in Fig.4)<br><br><u>unless</u> the definition of the outcome is met (as per Fig. 4) and first day with symptoms for their first symptomatic/severe COVID-19 episode <u>precedes all the events above, [A] – [D].</u>	Censored at the earlier of:<br>[A] or<br>[B] or<br>[E] date of withdrawal/last contact<br><br>unless the definition of the outcome is met (as per Fig. 4) and first day with symptoms for their first symptomatic/severe COVID-19 episode <u>precedes [A], [B] and [E]</u>.<br><br>[Episodes of illness with trigger/severe symptoms which the algorithm in Figure 4 cannot ascertain be a COVID-19 episode will be ignored from the censoring algorithm.]

*See section sensitivity analysis 3

6.3. SUPPLEMENTARY ANALYSES

The following supplementary analyses will be conducted on primary outcomes 1 and 2 with the intent to provide additional insights into the treatment effect:

i. Including follow-up after first dose of any COVID-19-specific vaccine (ie the intercurrent event of COVID-19-specific vaccine handled using a Treatment Policy Strategy. This analysis is summarised in estimands 1.2 and 2.2 below.

ii. Excluding COVID-19 episodes (either trigger or severe) starting ≤14 days from date of randomisation (ie the intercurrent event of symptomatic/severe COVID-19 in the first 14 days post randomisation, handled using a Principal Stratum Strategy). This analysis is summarised in estimands 1.3 and 2.3

below). For this analysis, time at risk of COVID-19 will start on the 15th day post randomisation date (as opposed to date of randomisation as for the primary analysis). In line with the definition of mITT that excludes participants who were exposed to COVID-19 prior to being randomised into the study, participants who:

- had a COVID-19 episodes (either symptomatic or severe), or
- had a trigger symptoms/severe episode reported, which the algorithm in Figure 4 cannot ascertain be a COVID-19 episode

starting ≤14 days from date of randomisation, will be excluded from this analysis.

iii. Censoring participants at the time of any subsequent vaccine (ie the intercurrent event of any vaccine, including influenza vaccination and COVID-19-specific vaccine, handled by the Hypothetical Strategy, summarised in estimands 1.4 and 2.4 below).

iv. on the ITT population (as summarised in estimands 1.5 and 2.5 below)

v. Treatment Policy Strategy on the ITT population (summarised in estimands 1.6 and 2.6 below).

The following table summarises the estimands of secondary interest around primary outcomes 1 and 2.

Objective	Estimand
To determine if BCG vaccination compared with placebo reduces the incidence of symptomatic COVID-19 irrespective of receiving a COVID-19-specific vaccine or any other vaccine, over the 6 months following randomisation, in healthcare workers who did not have a previous SARS-CoV-2 positive test result when assessed at time of randomisation.	Estimand 1.2 [Supplementary analysis i.]<br><br>Population: as for estimand 1.1<br>Outcome: as for estimand 1.1<br>Interventions: as for estimand 1.1<br>Handling of Intercurrent events:<br>- COVID-19 specific vaccine (Treatment Policy strategy)<br>- any other vaccine (Treatment Policy strategy)<br>Summary Measure: as for estimand 1.1
To determine if BCG vaccination compared with placebo reduces the incidence of symptomatic COVID-19 following the 14 days after randomisation in the absence of any COVID-19-specific vaccine, over the 6 months, in healthcare workers who did not have a previous SARS-CoV-2 positive test result when assessed at time of randomisation.	Estimand 1.3 [Supplementary analysis ii.]<br><br>Population: as for estimand 1.1<br>Outcome: as for estimand 1.1<br>Interventions: as for estimand 1.1<br>Handling of Intercurrent events: as for estimand 1.1<br>- COVID-19 specific vaccine (Hypothetical strategy)<br>- any other vaccine (Treatment Policy strategy)<br>- COVID-19 in the 14 days post randomisation (Principal Stratum Strategy)<br>Summary Measure: as for estimand 1.1
To determine if BCG vaccination compared with placebo reduces the incidence of symptomatic COVID-19 in the absence of any vaccine (including COVID-19-specific vaccine), over the 6 months following randomisation, in healthcare workers who did not have a previous SARS-CoV-2 positive test result when assessed at time of randomisation.	Estimand 1.4 [Supplementary analysis iii.]<br><br>Population: as for estimand 1.1<br>Outcome: as for estimand 1.1<br>Interventions: as for estimand 1.1<br>Handling of Intercurrent events:<br>- COVID-19 specific vaccine (Hypothetical Strategy)<br>- any other vaccine (Hypothetical strategy)<br>Summary Measure: as for estimand 1.1
To determine if BCG vaccination compared with placebo reduces the incidence of symptomatic COVID-19 in the absence of a COVID-19-specific vaccine, over the 6	Estimand 1.5 [Supplementary analysis iv.]<br><br>Population: ITT population

Objective	Estimand
months following randomisation, in healthcare workers exposed to SARS-CoV-2.	Outcome: as for estimand 1.1<br>Interventions: as for estimand 1.1<br>Handling of Intercurrent events: as for estimand 1.1<br>Summary Measure: as for estimand 1.1
To determine if BCG vaccination compared with placebo reduces the incidence of symptomatic COVID-19 irrespective of receiving a COVID-19-specific vaccine or any other vaccine, over the 6 months following randomisation, in healthcare workers.	Estimand 1.6 [Supplementary analysis v.]<br><br>Population: ITT population<br>Outcome: as for estimand 1.1<br>Interventions: as for estimand 1.1<br>Handling of Intercurrent events: as for estimand 1.2<br>Summary Measure: as for estimand 1.1
To determine if BCG vaccination compared with placebo reduces the incidence of severe COVID-19 irrespective of receiving a COVID-19 specific vaccine or any other vaccine, over the 6 months following randomisation, in healthcare workers who did not have a previous SARS-CoV-2 positive test result when assessed at time of randomisation.	Estimand 2.2 [Supplementary analysis i.]<br><br>Population: as for estimand 2.1<br>Outcome: as for estimand 2.1<br>Interventions: as for estimand 2.1<br>Handling of Intercurrent events:<br>- COVID-19 specific vaccine (Treatment Policy strategy)<br>- any other vaccine (Treatment Policy strategy)<br>Summary Measure: as for estimand 2.1
To determine if BCG vaccination compared with placebo reduces the incidence of severe COVID-19 following the 14 days after randomisation in the absence of any COVID-19 specific vaccine, over the 6 months, in healthcare workers who did not have a previous SARS-CoV-2 positive test result when assessed at time of randomisation.	Estimand 2.3 [Supplementary analysis ii.]<br><br>Population: as for estimand 2.1<br>Outcome: as for estimand 2.1<br>Interventions: as for estimand 2.1<br>Handling of Intercurrent events: as for estimand 2.1<br>- COVID-19 specific vaccine (Hypothetical strategy)<br>- any other vaccine (Treatment Policy strategy)<br>- COVID-19 in the 14 days post randomisation (Principal Stratum Strategy)<br>Summary Measure: as for estimand 2.1
To determine if BCG vaccination compared with placebo reduces the incidence of severe COVID-19 in the absence of any vaccine (including a COVID-19 specific vaccine), over the 6 months following randomisation, in healthcare workers who did not have a previous SARS-CoV-2 positive test result when assessed at time of randomisation.	Estimand 2.4 [Supplementary analysis iii.]<br><br>Population: as for estimand 2.1<br>Outcome: as for estimand 2.1<br>Interventions: as for estimand 2.1<br>Handling of Intercurrent events:<br>- COVID-19 specific vaccine (Hypothetical Strategy)<br>- any other vaccine (Hypothetical strategy)<br>Summary Measure: as for estimand 2.1
To determine if BCG vaccination compared with placebo reduces the incidence of severe COVID-19 in the absence of a COVID-19 specific vaccine, over the 6 months following randomisation, in healthcare workers exposed to SARS-CoV-2.	Estimand 2.5 [Supplementary analysis iii.]<br><br>Population: ITT population<br>Outcome: as for estimand 2.1<br>Interventions: as for estimand 2.1<br>Handling of Intercurrent events: as for estimand 2.1<br>Summary Measure: as for estimand 2.1
To determine if BCG vaccination compared with placebo reduces the incidence of severe COVID-19 irrespective of receiving a COVID-19-specific vaccine or any other	Estimand 2.6 [Supplementary analysis v.]<br><br>Population: ITT population<br>Outcome: as for estimand 2.1

Objective	Estimand
vaccine, over the 6 months following randomisation, in healthcare workers.	Interventions: as for estimand 2.1<br>Handling of Intercurrent events: as for estimand 2.2<br>Summary Measure: as for estimand 2.1
** 1) adjusted for stratification factors used at randomisation (age group, presence of comorbidity, and geographical location); 2) adjusted for stratification factors used at randomisation + sex, BMI at baseline, BCG vaccine before randomisation;*	

The analyses for estimands 1.2-1.6 and 2.2-2.6 will be conducted using the same methodology as for the primary analysis (estimand 1.1 and 2.1, as specified in section 6.2.2).

6.4. SUBGROUP ANALYSES

The following sub-group analyses will be performed (only for estimands 1.1 and 2.1, unless otherwise indicated), but since we have not powered the trial to consider sub-groups, the results will be considered exploratory only. These sub-group analyses will examine the evidence for differences in the effect of the intervention between the sub-groups. The intervention effect in each sub-group and their 95% confidence intervals will be presented, together with the p-value for the intervention-by-subgroup interaction, as a guide to the strength of the evidence for an interaction.

1 – By age group (stratification factor at randomisation)

Subgroups will be defined by age at randomisation, as follows:

- <40 years vs.
- 40 to 59 years vs.
- >=60 years

2 – By presence of comorbidities

Subgroups will be:

- Presence of comorbidity at randomisation (any of diabetes, chronic respiratory disease, cardiovascular disease -including hypertension/high blood pressure, and obesity defined as BMI >= 30 kg/m^2) vs.
- Absence of comorbidity

2a – By presence of diabetes

Subgroups will be:

- Presence of diabetes at randomisation vs.
- Absence of diabetes at randomisation

2b – By presence of chronic respiratory disease

Subgroups will be:

- Presence of chronic respiratory disease at randomisation vs.
- Absence of chronic respiratory disease at randomisation

2c – By presence of ANY cardiovascular disease

Subgroups will be:

- Presence of cardiovascular disease at randomisation vs.
- Absence of cardiovascular disease at randomisation

2d – By presence of hypertension/high blood pressure

Subgroups will be:

- Presence of hypertension at randomisation vs.
- Absence of hypertension at randomisation

2e – By presence of obesity (BMI>=30 kg/m2)

Subgroups will be:
- Presence of obesity at randomisation vs
- Absence of obesity at randomisation

3 – By geographical Location
Sub-groups will be:
- Australia vs
- Europe vs
- South America

4 – By sex
Sub-groups will be:
- Females vs
- Males

5 – By BCG in the past or not
Sub-groups will be:
- Participants who received BCG vaccine before participating in the trial vs
- Participants who never received BCG vaccine before participating in the trial

Prior BCG vaccination status will be ascertained by self-reported answer to the question "Have you been vaccinated with BCG in the past?".

6 – By baseline serology results to SARS-CoV-2 (negative or non-negative) [ITT population only]
Sub-groups will be:
- Participants with negative serology to SARS-CoV-2 when enrolling into the trial (participants at the Brazilian sites will also need to show negative PCR on a respiratory sample or a negative SARS-CoV-2 diagnostic antigen test approved by the local jurisdiction's public health policy at the time of randomisation) vs
- Participants with non-negative (ie positive/missing/indeterminant) serology to SARS-CoV-2 when enrolling into the trial (Applicable only to participants at the Brazilian sites: positive/missing/indeterminant PCR on a respiratory sample or a positive/missing/indeterminant SARS-CoV-2 diagnostic antigen test approved by the local jurisdiction's public health policy at the time of randomisation)

This subgroup analysis will only be run on the ITT population (estimand 1.5 and 2.5).

6.5. SENSITIVITY ANALYSES

Sensitivity analysis 1 – BCG/Placebo vaccination date (as opposed to their randomisation date)
The primary analysis includes follow up data from the randomisation date (day 0), which is also when most participants received their trial BCG/placebo injection. However, a small number of participants received the intervention several days or weeks following randomisation. A sensitivity analysis on estimands 1.1 and 2.1 will be run, to have these participants follow up data start on the actual vaccination date (as opposed to their randomisation date).

Objective	Estimand
To determine if BCG vaccination compared with placebo reduces the incidence of symptomatic COVID-19 in the absence of a COVID-19 specific vaccine, over the 6 months following vaccination, in healthcare workers who did not have a previous SARS-CoV-2 positive test result when assessed at time of randomisation.	Estimand 1.1_s1 [Sensitivity analysis 1]<br><br>Population: as for estimand 1.1<br>Outcome: symptomatic COVID-19 by 6 months following vaccination<br>Interventions: as for estimand 1.1<br>Handling of Intercurrent events: as for estimand 1.1<br>Summary Measure: as for estimand 1.1

Objective	Estimand
To determine if BCG vaccination compared with placebo reduces the incidence of severe COVID-19 in the absence of a COVID-19 specific vaccine, over the 6 months following vaccination, in healthcare workers who did not have a previous SARS-CoV-2 positive test result when assessed at time of randomisation.	Estimand 2.1_s1 [Sensitivity analysis 1]<br><br>Population: as for estimand 2.1<br>Outcome: severe COVID-19 by 6 months following vaccination<br>Interventions: as for estimand 2.1<br>Handling of Intercurrent events: as for estimand 2.1<br>Summary Measure: as for estimand 2.1
** 1) adjusted for stratification factors used at randomisation (age group, presence of comorbidity, and geographical location); 2) adjusted for stratification factors used at randomisation + sex, BMI at baseline, BCG vaccine before randomisation;*	

Sensitivity analysis 2 – Clinical algorithm based only on combined PCR/RAT result (Fig. 2) using the ITT population (i.e. disregarding all serology testing and baseline PCR in Brazil)

As a second sensitivity analysis on estimands 1.1 and 2.1, for the derivation of the primary outcomes, episodes of illness will be re-categorised as COVID-19 episodes (either symptomatic or severe), non-COVID-19 episodes, episodes with missing information using only the combination of the results from RAT and PCR tests (as in Figure 2).

Objective	Estimand
To determine if BCG vaccination compared with placebo reduces the incidence of symptomatic COVID-19 (determined using PCR/RAT tests only) in the absence of a COVID-19 specific vaccine, over the 6 months following randomisation, in healthcare workers.	Estimand 1.1_s2 [Sensitivity analysis 2]<br><br>Population: ITT population<br>Outcome: symptomatic COVID-19 by 6 months determined using PCR/RAT tests only<br>Interventions: as for estimand 1.1<br>Handling of Intercurrent events: as for estimand 1.1<br>Summary Measure: as for estimand 1.1
To determine if BCG vaccination compared with placebo reduces the incidence of severe COVID-19 (determined using PCR/RAT tests only) in the absence of a COVID-19 specific vaccine, over the 6 months following randomisation, in healthcare workers.	Estimand 2.1_s2 [Sensitivity analysis 2]<br><br>Population: ITT population<br>Outcome: severe COVID-19 by 6 months determined using PCR/RAT tests only<br>Interventions: as for estimand 2.1<br>Handling of Intercurrent events: as for estimand 2.1<br>Summary Measure: as for estimand 2.1
** 1) adjusted for stratification factors used at randomisation (age group, presence of comorbidity, and geographical location); 2) adjusted for stratification factors used at randomisation + sex, BMI at baseline, BCG vaccine before randomisation;*	

Sensitivity analysis 3

A third sensitivity analysis on estimands 1.1-1.6 and 2.1-2.6 will adopt a modified version of the censoring rule section as presented in table 2, in the column "SENSITIVITY ANALYSIS" (section 6.2.3).

7. SECONDARY COVID-19 RELATED OUTCOMES

Similarly to the analyses of the primary outcomes, the analyses of the COVID-19 related secondary outcomes will be conducted following three different approaches, specifically:

1. Censoring participants at the time of their COVID-19 specific vaccine (ie the intercurrent event of COVID-19-specific vaccine, will be handled using a Hypothetical Strategy)
2. Including follow-up after first dose of any COVID-19-specific vaccine (ie the intercurrent event of COVID-19-specific vaccine, will be handled using a Treatment Policy Strategy)

3. Censoring participants at the time of any vaccine (ie the intercurrent event of any vaccine, including influenza vaccination and COVID-19-specific vaccine, will be handled using a Hypothetical Strategy)

The analyses will be conducted on the mITT population, unless otherwise indicated, and including only Stage 2 participants.

7.1. SYMPTOMATIC COVID-19 AND SEVERE COVID-19 BY 12MONTHS (#3 and #4)

7.1.1. ESTIMANDS

Objective	Estimand
To determine if BCG vaccination compared with placebo reduces the incidence of symptomatic COVID-19 in the absence of a COVID-19 specific vaccine, over the 12 months following randomisation, in healthcare workers who did not have a previous SARS-CoV-2 positive test result when assessed at time of randomisation.	Estimand 3.1<br><br>Population: mITT population<br>Outcome: symptomatic COVID-19 by 12 months<br>Interventions: BCG vs Placebo<br>Handling of Intercurrent events:<br>- COVID-19 specific vaccine (Hypothetical Strategy)<br>- any other vaccine (Treatment Policy strategy)<br>Summary Measure: Adjusted* difference in proportion of participants
To determine if BCG vaccination compared with placebo reduces the incidence of symptomatic COVID-19 irrespective of receiving a COVID-19 specific vaccine or any other vaccine, over the 12 months following randomisation, in healthcare workers who did not have a previous SARS-CoV-2 positive test result when assessed at time of randomisation.	Estimand 3.2<br><br>Population: as for estimand 3.1<br>Outcome: as for estimand 3.1<br>Interventions: as for estimand 3.1<br>Handling of Intercurrent events:<br>- COVID-19 specific vaccine (Treatment Policy strategy)<br>- any other vaccine (Treatment Policy strategy)<br>Summary Measure: as for estimand 3.1
To determine if BCG vaccination compared with placebo reduces the incidence of symptomatic COVID-19 in the absence of a COVID-19 specific vaccine, over the 12 months following the 14 days after randomisation, in healthcare workers who did not have a previous SARS-CoV-2 positive test result when assessed at time of randomisation.	Estimand 3.3<br><br>Population: as for estimand 3.1<br>Outcome: symptomatic COVID-19, but excluding COVID-19 episodes that started in the 14 days window after randomisation<br>Interventions: as for estimand 3.1<br>Handling of Intercurrent events: as for estimand 3.1<br>Summary Measure: as for estimand 3.1
To determine if BCG vaccination compared with placebo reduces the incidence of symptomatic COVID-19 in the absence of any other vaccine (including COVID-19 specific vaccine), over the 12 months following randomisation, in healthcare workers who did not have a previous SARS-CoV-2 positive test result when assessed at time of randomisation.	Estimand 3.4<br><br>Population: as for estimand 3.1<br>Outcome: as for estimand 3.1<br>Interventions: as for estimand 3.1<br>Handling of Intercurrent events:<br>- COVID-19 specific vaccine (Hypothetical Strategy)<br>- any other vaccine (Hypothetical strategy)<br>Summary Measure: as for estimand 3.1
To determine if BCG vaccination compared with placebo reduces the incidence of symptomatic COVID-19 in the absence of a COVID-19 specific vaccine, over the 12 months following randomisation, in healthcare workers exposed to SARS-CoV-2.	Estimand 3.5<br><br>Population: ITT population<br>Outcome: as for estimand 3.1<br>Interventions: as for estimand 3.1<br>Handling of Intercurrent events: as for estimand 3.1<br>Summary Measure: as for estimand 3.1

Objective	Estimand
To determine if BCG vaccination compared with placebo reduces the incidence of symptomatic COVID-19 irrespective of receiving a COVID-19 specific vaccine or any other vaccine, over the 12 months following randomisation, in healthcare workers.	Estimand 3.6<br><br>Population: ITT population<br>Outcome: as for estimand 3.1<br>Interventions: as for estimand 3.1<br>Handling of Intercurrent events: as for estimand 3.2<br>Summary Measure: as for estimand 3.1
To determine if BCG vaccination compared with placebo reduces the incidence of severe COVID-19 in the absence of a COVID-19 specific vaccine, over the 12 months following randomisation, in healthcare workers who did not have a previous SARS-CoV-2 positive test result when assessed at time of randomisation.	Estimand 4.1<br><br>Population: mITT population<br>Outcome: severe COVID-19 by 12 months<br>Interventions: BCG vs Placebo<br>Handling of Intercurrent events:<br>- COVID-19 specific vaccine (Hypothetical Strategy)<br>- any other vaccine (Treatment Policy strategy)<br>Summary Measure: Adjusted* difference in proportion of participants
To determine if BCG vaccination compared with placebo reduces the incidence of severe COVID-19 irrespective of receiving a COVID-19 specific vaccine or any other vaccine, over the 12 months following randomisation, in healthcare workers who did not have a previous SARS-CoV-2 positive test result when assessed at time of randomisation.	Estimand 4.2<br><br>Population: as for estimand 4.1<br>Outcome: as for estimand 4.1<br>Interventions: as for estimand 4.1<br>Handling of Intercurrent events:<br>- COVID-19 specific vaccine (Treatment Policy strategy)<br>- any other vaccine (Treatment Policy strategy)<br>Summary Measure: as for estimand 4.1
To determine if BCG vaccination compared with placebo reduces the incidence of severe COVID-19 in the absence of a COVID-19 specific vaccine, over the 12 months following the 14 days after randomisation, in healthcare workers who did not have a previous SARS-CoV-2 positive test result when assessed at time of randomisation.	Estimand 4.3<br><br>Population: as for estimand 4.1<br>Outcome: severe COVID-19, but excluding severe COVID-19 episodes that started in the 14 days window after randomisation<br>Interventions: as for estimand 4.1<br>Handling of Intercurrent events: as for estimand 4.1<br>Summary Measure: as for estimand 4.1
To determine if BCG vaccination compared with placebo reduces the incidence of severe COVID-19 in the absence of any vaccine (including COVID-19 specific vaccine), over the 12 months following randomisation, in healthcare workers who did not have a previous SARS-CoV-2 positive test result when assessed at time of randomisation.	Estimand 4.4<br><br>Population: as for estimand 4.1<br>Outcome: as for estimand 4.1<br>Interventions: as for estimand 4.1<br>Handling of Intercurrent events:<br>- COVID-19 specific vaccine (Hypothetical Strategy)<br>- any other vaccine (Hypothetical strategy)<br>Summary Measure: as for estimand 4.1
To determine if BCG vaccination compared with placebo reduces the incidence of severe COVID-19 in the absence of a COVID-19 specific vaccine, over the 12 months following randomisation, in healthcare workers.	Estimand 4.5<br><br>Population: ITT population<br>Outcome: as for estimand 4.1<br>Interventions: as for estimand 4.1<br>Handling of Intercurrent events: as for estimand 4.1<br>Summary Measure: as for estimand 4.1
To determine if BCG vaccination compared with placebo reduces the incidence of severe COVID-19 irrespective of receiving a COVID-19 specific vaccine	Estimand 4.6<br><br>Population: ITT population

Objective	Estimand
or any other vaccine, over the 12 months following randomisation, in healthcare workers.	Outcome: as for estimand 4.1<br>Interventions: as for estimand 4.1<br>Handling of Intercurrent events: as for estimand 4.2<br>Summary Measure: as for estimand 4.1
** 1) adjusted for stratification factors used at randomisation (age group, presence of comorbidity, and geographical location); 2) adjusted for stratification factors used at randomisation + sex, BMI at baseline, BCG vaccine before randomisation;*	

7.1.2. ANALYSIS

Since outcomes 3 and 4 are the equivalent of the primary outcomes 1 and 2 over 12 months post randomisation, the same analyses that are presented in section 6.2 will be applied on these outcomes (summarised in estimands 3.1 to 3.6 for symptomatic COVID-19 by 12 months and in estimands 4.1 to 4.6 for severe COVID -19 outcome)

7.1.3. ADDITIONAL ANALYSIS

Additional outcomes 3 and 4 will be compared between the following groups:

- Participants who received BCG vaccine at recruitment AND who show evidence of the scar left by this vaccine 12 months post randomisation
- Participants who received placebo AND participants who received BCG vaccine at recruitment but don't show evidence of the scar left by this vaccine 12 months post randomisation

The same analyses that are presented in section 6.2 will be run.

7.1.4. SUBGROUP ANALYSIS

The following subgroup analyses (described in detail in section 6.4) will be conducted for estimands 3.1 and 4.1:

- Sub-Group analysis 1 – Age group (stratification factor at randomisation)
- Sub-Group analyses 2, 2a, 2b, 2c, 2d, 2e – Presence of comorbidities
- Sub-Group analysis 3 – Geographical Location
- Sub-Group analysis 4 – Sex
- Sub-Group analysis 5 – BCG in the past or not
- Sub-Group analysis 6 – Serology results to SARS-CoV-2 at enrolment (ITT pop only, estimand 3.5 and 4.5)

Additionally, a second categorisation of subgroup analysis 5 will be done to include evidence of a scar due to BCG vaccine in the past, as assessed by the immuniser and the photo of the scar review by clinical BRACE team unblinded to the treatment allocation. The will be conducted as follows:

- Sub-Group analysis 5a – By BCG in the past or not (as confirmed by scar evidence)
  - Participants who received BCG vaccine before participating in the trial and show evidence of a scar vs
  - Participants who never received BCG vaccine before participating in the trial and show no evidence of a scar

This subgroup analysis will exclude those participants who reported prior BCG vaccine but have no evidence of a scar, and those who did not report prior BCG vaccine but have evidence of a scar.

7.2. TIME TO FIRST SYMPTOM OF COVID-19 (#5a and #5b)

7.2.1.ESTIMANDS

Objective	Estimand
To determine if BCG vaccination compared with placebo prolongs the time to first SARS-CoV-2-proven respiratory illness in the absence of a COVID-19 specific vaccine, measured over 6 months following randomisation in healthcare workers who did not have a previous SARS-CoV-2 positive test result when assessed at time of randomisation.	Estimand 5a.1<br><br>Population: mITT population<br>Outcome: time to COVID-19 by 6 months<br>Interventions: BCG vs Placebo<br>Handling of Intercurrent events:<br>- COVID-19 specific vaccine (Hypothetical Strategy)<br>- any other vaccine (Treatment Policy strategy)<br>Summary Measure: Adjusted* hazard ratios
To determine if BCG vaccination compared with placebo prolongs the time to first SARS-CoV-2-proven respiratory illness in the absence of a COVID-19 specific vaccine, measured over 12 months following randomisation in healthcare workers who did not have a previous SARS-CoV-2 positive test result when assessed at time of randomisation.	Estimand 5b.1<br><br>Population: mITT population<br>Outcome: time to COVID-19 by 12 months<br>Interventions: BCG vs Placebo<br>Handling of Intercurrent events:<br>- COVID-19 specific vaccine (Hypothetical Strategy)<br>- any other vaccine (Treatment Policy strategy)<br>Summary Measure: Adjusted* hazard ratios
To determine if BCG vaccination compared with placebo prolongs the time to first SARS-CoV-2-proven respiratory illness irrespective of receiving a COVID-19 specific vaccine or any other vaccine, measured over 6 months following randomisation in healthcare workers who did not have a previous SARS-CoV-2 positive test result when assessed at time of randomisation.	Estimand 5a.2<br><br>Population: as 5a.1<br>Outcome: as 5a.1<br>Interventions: as 5a.1<br>Handling of Intercurrent events:<br>- COVID-19 specific vaccine (Treatment Policy strategy)<br>- any other vaccine (Treatment Policy strategy)<br>Summary Measure: as 5a.1
To determine if BCG vaccination compared with placebo prolongs the time to first SARS-CoV-2-proven respiratory illness irrespective of receiving a COVID-19 specific vaccine or any other vaccine, measured over 12 months following randomisation in healthcare workers who did not have a previous SARS-CoV-2 positive test result when assessed at time of randomisation.	Estimand 5b.2<br><br>Population: as 5b.1<br>Outcome: as 5b.1<br>Interventions: as 5b.1<br>Handling of Intercurrent events:<br>- COVID-19 specific vaccine (Treatment Policy strategy)<br>- any other vaccine (Treatment Policy strategy)<br>Summary Measure: as 5b.1
To determine if BCG vaccination compared with placebo prolongs the time to first SARS-CoV-2-proven respiratory illness following the 14 days after randomisation irrespective of receiving a COVID-19 specific vaccine or any other vaccine, measured over the 6 months in healthcare workers who did not have a previous SARS-CoV-2 positive test result when assessed at time of randomisation.	Estimand 5a.3<br><br>Population: as 5a.1<br>Outcome: time to COVID-19 by 6 months, but excluding COVID-19 episodes that started in the 14 days window after randomisation<br>Interventions: as 5a.1<br>Handling of Intercurrent events: as 5a.1<br>Summary Measure: as 5a.1
To determine if BCG vaccination compared with placebo prolongs the time to first SARS-CoV-2-proven respiratory illness following the 14 days after randomisation irrespective of receiving a COVID-19 specific vaccine or any other vaccine, measured over the 12 months in healthcare workers who did not have a previous SARS-CoV-2 positive test result when assessed at time of randomisation.	Estimand 5b.3<br><br>Population: as 5b.1<br>Outcome: time to COVID-19 by 12 months, but excluding COVID-19 episodes that started in the 14 days window after randomisation<br>Interventions: as 5b.1<br>Handling of Intercurrent events: as 5a.1<br>Summary Measure: as 5b.1

Objective	Estimand
To determine if BCG vaccination compared with placebo prolongs the time to first SARS-CoV-2-proven respiratory illness in the absence of any vaccine, measured over 6 months following randomisation in healthcare workers who did not have a previous SARS-CoV-2 positive test result when assessed at time of randomisation.	Estimand 5a.4<br><br>Population: as 5a.1<br>Outcome: as 5a.1<br>Interventions: as 5a.1<br>Handling of Intercurrent events:<br>- COVID-19 specific vaccine (Hypothetical Strategy)<br>- any other vaccine (Hypothetical Strategy)<br>Summary Measure: as 5a.1
To determine if BCG vaccination compared with placebo prolongs the time to first SARS-CoV-2-proven respiratory illness in the absence of any vaccine, measured over 12 months following randomisation in healthcare workers who did not have a previous SARS-CoV-2 positive test result when assessed at time of randomisation.	Estimand 5b.4<br><br>Population: as for estimand 5b.1<br>Outcome: as for estimand 5b.1<br>Interventions: as for estimand 5b.1<br>Handling of Intercurrent events:<br>- COVID-19 specific vaccine (Hypothetical Strategy)<br>- any other vaccine (Hypothetical Strategy)<br>Summary Measure: as for estimand 5b.1
To determine if BCG vaccination compared with placebo prolongs the time to first SARS-CoV-2-proven respiratory illness in the absence of a COVID-19 specific vaccine, measured over 6 months following randomisation in healthcare.	Estimand 5a.5<br><br>Population: ITT population<br>Outcome: as for estimand 5a.1<br>Interventions: as for estimand 5a.1<br>Handling of Intercurrent events: as for estimand 5a.1<br>Summary Measure: as for estimand 5a.1
To determine if BCG vaccination compared with placebo prolongs the time to first SARS-CoV-2-proven respiratory illness in the absence of a COVID-19 specific vaccine, measured over 12 months following randomisation in healthcare.	Estimand 5b.5<br><br>Population: ITT population<br>Outcome: as for estimand 5b.1<br>Interventions: as for estimand 5b.1<br>Handling of Intercurrent events: as for estimand 5b.1<br>Summary Measure: as for estimand 5b.1
To determine if BCG vaccination compared with placebo prolongs the time to first SARS-CoV-2-proven respiratory illness irrespective of receiving a COVID-19 specific vaccine or any other vaccine, measured over 6 months following randomisation in healthcare.	Estimand 5a.6<br><br>Population: ITT population<br>Outcome: as for estimand 5a.1<br>Interventions: as for estimand 5a.1<br>Handling of Intercurrent events: as for estimand 5a.2<br>Summary Measure: as for estimand 5a.1
To determine if BCG vaccination compared with placebo prolongs the time to first SARS-CoV-2-proven respiratory illness rrespective of receiving a COVID-19 specific vaccine or any other vaccine, measured over 12 months following r40andomization in healthcare.	Estimand 5b.6<br><br>Population: ITT population<br>Outcome: as for estimand 5b.1<br>Interventions: as for estimand 5b.1<br>Handling of Intercurrent events: as for estimand 5b.2<br>Summary Measure: as for estimand 5b.1
** 1) adjusted for stratification factors used at randomisation (age group, presence of comorbidity, and geographical location); 2) adjusted for stratification factors used at randomisation + sex, BMI at baseline, BCG vaccine before randomisation;*	

7.2.1. ANALYSIS

The time to COVID-19 (either symptomatic or severe COVID-19) will be calculated and presented in the two intervention groups.
Survival curves for the time to COVID-19 episode will be constructed for each intervention group using the Kaplan-Meier product limit method.

Adjusted analyses will be used to compare the time to event distributions between groups by means of Cox's proportional hazards model. Initially the model will be adjusted for the stratification factors used during randomisation (age group, presence of comorbidity, geographical location -Europe/Australia/South America). Additional analyses will be conducted using the same Cox's proportional hazards but also adjusted for the following baseline covariates (as described in section 6.2.2): sex, BMI, BCG prior to study enrolment. The proportional hazards assumption will be checked when running these analyses. Assuming the proportional hazards assumption is found to be reasonable, results from the proportional hazards regression will be presented as the hazard ratios and their corresponding 95% confidence intervals.

7.2.2. SUBGROUP ANALYSIS

The following subgroup analyses (described in details in section 6.4) will be conducted for estimands 5a.1 and 5b.1:

- Sub-Group analysis 1 – Age group (stratification factor at randomisation)
- Sub-Group analyses 2, 2a, 2b, 2c, 2d, 2e – Presence of comorbidities
- Sub-Group analysis 3 – Geographical Location
- Sub-Group analysis 4 – Sex
- Sub-Group analysis 5 – BCG in the past or not
- Sub-Group analysis 6 – serology results to SARS-CoV-2 at enrolment (ITT pop only, estimands 5a.5 and 5b.5)

7.3. NUMBER OF EPISODES OF COVID-19 (#6a and #6b)

7.3.1. ESTIMANDS

Objective	Estimand
To determine if BCG vaccination compared with placebo reduces the number of COVID-19 episodes in the absence of a COVID-19 specific vaccine, measured over 6 months following randomisation in healthcare workers who did not have a previous SARS-CoV-2 positive test result when assessed at time of randomisation.	Estimand 6a.1<br><br>Population: mITT population<br>Outcome: number of COVID-19 episodes by 6 months<br>Interventions: BCG vs Placebo<br>Handling of Intercurrent events:<br>- COVID-19 specific vaccine (Hypothetical Strategy)<br>- any other vaccine (Treatment Policy strategy)<br>Summary Measure: Adjusted* difference in the expected counts
To determine if BCG vaccination compared with placebo reduces the number of COVID-19 episodes in the absence of a COVID-19 specific vaccine, measured over 12 months following randomisation in healthcare workers who did not have a previous SARS-CoV-2 positive test result when assessed at time of randomisation.	Estimand 6b.1<br><br>Population: mITT population<br>Outcome: number of COVID-19 episodes by 12 months<br>Interventions: BCG vs Placebo<br>Handling of Intercurrent events:<br>- COVID-19 specific vaccine (Hypothetical Strategy)<br>- any other vaccine (Treatment Policy strategy)<br>Summary Measure: Adjusted* difference in the expected counts
To determine if BCG vaccination compared with placebo reduces the number of COVID-19 episodes irrespective of receiving a COVID-19 specific vaccine or any other vaccine, measured over 6 months	Estimand 6a.2<br><br>Population: as 6a.1<br>Outcome: as 6a.1

Objective	Estimand
following randomisation in healthcare workers who did not have a previous SARS-CoV-2 positive test result when assessed at time of randomisation.	Interventions: as 6a.1<br>Handling of Intercurrent events:<br>- COVID-19 specific vaccine (Treatment Policy strategy)<br>- any other vaccine (Treatment Policy strategy)<br>Summary Measure: as 6a.1
To determine if BCG vaccination compared with placebo reduces the number of COVID-19 episodes irrespective of receiving a COVID-19 specific vaccine or any other vaccine, measured over 12 months following randomisation in healthcare workers who did not have a previous SARS-CoV-2 positive test result when assessed at time of randomisation.	Estimand 6b.2<br><br>Population: as 6b.1<br>Outcome: as 6b.1<br>Interventions: as 6b.1<br>Handling of Intercurrent events:<br>- COVID-19 specific vaccine (Treatment Policy strategy)<br>- any other vaccine (Treatment Policy strategy)<br>Summary Measure: as 6b.1
To determine if BCG vaccination compared with placebo reduces the number of COVID-19 episodes illness in the absence of any vaccine, measured over 6 months following randomisation in healthcare workers who did not have a previous SARS-CoV-2 positive test result when assessed at time of randomisation.	Estimand 6a.3<br><br>Population: as 6a.1<br>Outcome: as 6a.1<br>Interventions: as 6a.1<br>Handling of Intercurrent events:<br>- COVID-19 specific vaccine (Hypothetical Strategy)<br>- any other vaccine (Hypothetical Strategy)<br>Summary Measure: as 6a.1
To determine if BCG vaccination compared with placebo reduces the number of COVID-19 episodes in the absence of any vaccine, measured over 12 months following randomisation in healthcare workers who did not have a previous SARS-CoV-2 positive test result when assessed at time of randomisation.	Estimand 6b.3<br><br>Population: as 6b.1<br>Outcome: as 6b.1<br>Interventions: as 6b.1<br>Handling of Intercurrent events:<br>- COVID-19 specific vaccine (Hypothetical Strategy)<br>- any other vaccine (Hypothetical Strategy)<br>Summary Measure: as 6b.1
** 1) adjusted for stratification factors used at randomisation (age group, presence of comorbidity, and geographical location)*	

7.3.2. ANALYSIS

The median and IQR for the number of episodes will be presented by intervention group.
The difference between BCG and placebo groups will be summarised as difference in the logs of expected number of episodes and its 95%CI estimated using a Zero-Inflated Negative Binomial (ZINB) model. Since this secondary outcome will be analysed mainly for descriptive purposes, the model will only be adjusted for the stratification factors used at randomisation (Geographical Location (Australia/Europe/South America, age group and presence of comorbidity).
The analysis will be done using the *zinb* command in Stata, with the *inflate()* and *exposure()* options. The *inflate()* option will be used to indicate whether the participant had the COVID-19 event, while the *exposure()* option will be used to indicate the amount of exposure over which the number of episodes of COVID-19 were observed for each participant (refer to section 6.2.3).

7.3.3. ADDITIONAL ANALYSIS

Additionally, the median and IQR of the number of episodes will be calculated and presented by intervention group in the subgroups of participants who:

- had COVID-19 (either symptomatic or severe)
- had symptomatic COVID-19
- had severe COVID-19

7.3.4. SUBGROUP ANALYSIS

None

7.4. ASYMPTOMATIC SARS-COV-2 INFECTION (#7)

7.4.1. ESTIMANDS

Objective	Estimand
To determine if BCG vaccination compared with placebo reduces the incidence of asymptomatic COVID-19 irrespective of receiving a COVID-19 specific vaccine or any other vaccine, over the 6 months following randomisation, in healthcare workers who did not have a previous SARS-CoV-2 positive test result when assessed at time of randomisation.	Estimand 7a.1<br><br>Population: mITT population<br>Outcome: asymptomatic SARS-COV-2 infection by 6 months<br>Interventions: BCG vs Placebo<br>Handling of Intercurrent events:<br>- COVID-19 specific vaccine (Treatment Policy strategy)<br>- CoronaVac vaccine (Principal Stratum strategy)<br>- any other other vaccine (Treatment Policy strategy)<br>Summary Measure: Adjusted* difference in proportion of participants
To determine if BCG vaccination compared with placebo reduces the incidence of asymptomatic COVID-19 irrespective of receiving a COVID-19 specific vaccine or any other vaccine, over the 12 months following randomisation, in healthcare workers who did not have a previous SARS-CoV-2 positive test result when assessed at time of randomisation.	Estimand 7b.1<br><br>Population: mITT population<br>Outcome: asymptomatic SARS-COV-2 infection by 12 months<br>Interventions: BCG vs Placebo<br>Handling of Intercurrent events:<br>- COVID-19 specific vaccine (Treatment Policy strategy)<br>- any other other vaccine (Treatment Policy strategy)<br>Summary Measure: Adjusted* difference in proportion of participants
** 1) adjusted for stratification factors used at randomisation (age group, presence of comorbidity, and geographical location); 2) adjusted for stratification factors used at randomisation + sex, BMI at baseline, BCG vaccine before randomisation*	

7.4.2. ANALYSIS

The outcome of asymptomatic COVID-19, determined by seroconversion at 3 or 6 months not associated with any episode of illness will be described by intervention group as the absolute number of participants with the outcome.

The treatment effect for this outcome will be the difference in proportion (BCG - Control) estimated using a binomial regression model, adjusted for stratification factors used at randomisation (age group, presence of comorbidity, and geographical location). An additional analysis will be conducted using the same binomial regression model but also adjusted for the following baseline covariates (as described in section 6.2.2): sex, BMI, and BCG prior to study enrolment. Should the binomial regression model have convergence difficulties (due to low prevalence outcome), generalised liner model (GLM) approach with Gaussian error distribution and identity link function will be adopted.

Since the administration of CoronaVac (which is a post randomisation intercurrent event) more than 7 days prior to the blood collection makes the results of serology indeterminant, CoronaVac will be handled the Principal Stratum strategy i.e. estimating the treatment effect in participants who would not have received CoronaVac in either treatment arm. For the main analysis we will assume that the occurrence of this intercurrent event is not related to the study intervention (BCG/Placebo), and we will restrict the analysis to participants who did not receive CoronaVac (all participants who received CoronaVac will be excluded from the analysis).

As a sensitivity analysis, we will repeat the analysis under the assumption of conditional independence of the treatment and the intercurrent event (known as principal ignorability). For this analysis, separate models will be specified for the outcome and the intercurrent event itself, assuming that conditional on baseline covariates the outcome in the control group and the occurrence of the intercurrent event in the intervention group are independent. In order to conduct this analysis, we will first model the probability of the occurrence of the intercurrent event on the intervention group as well study site, sex, presence of comorbidities, and age (variables that could potentially confound the outcome and the intercurrent event), using logistic regression. Then, we will use the predicted probabilities as weights for participants on the control group in the analysis model for the outcome.

As a second, and very conservative, sensitivity analysis, participants in study sites where CoronaVac was available (Mato Grosso do Sul, Rio de Janeiro, and Amazonas -BRA), will be excluded from the analysis of this outcome so that this intercurrent event is no longer relevant.

As an additional approach we will use the principal stratum strategy, which classifies subjects according to their potential occurrence of an intercurrent event on both study groups.

Participants with unavailable/indeterminant serology data, and participants on whom the absence of episodes of illness over 6 months cannot be ascertained (due to incomplete data entry) will be coded as missing the outcome.

7.4.3. ADDITIONAL ANALYSIS

Since a SARS-CoV-2 infection could also be detected by a positive PCR/RAT not associated with a severe episode of illness or an episode with “trigger” symptoms” (e.g., SARS-CoV-2 infection detected via a screening test), and since seroconversion could also be explained by not severe SARS-CoV-2 infection not characterised by trigger symptoms, we will calculate and report by intervention group the number of participants who:

- Show evidence of COVID-19 determined by a positive PCR or positive RAT (and not serology) not related any episode of illness OR
- Show evidence of COVID-19 determined by a positive PCR or positive RAT (and not serology) related to a non-trigger non-severe episode of illness
- Show evidence of COVID-19 determined by seroconversion at 3 or 6 months associated with an episode of illness which is not trigger AND not severe (as defined in section 4.1)

Moreover, in those participants who meet the asymptomatic outcome the following information will be described and reported by intervention group as mean, standard deviation (or median and IQR if distribution is skewed) or absolute and relative frequencies, according to the nature of the variable:

- Sex – Male/Female/Other/Declined/Missing
- Age, years
- Body Mass Index (BMI), kg/m^2 – < 18.5 / 18.5 to 24.9/ 25 to 29.9 / >30 / Missing

- Role – Administrative-clerical staff / Allied Health / Dentist-dental therapy / Doctor / Nurse-Midwife / Patient Services Assistant-hospital maintenance / Scientist (medical research) / Other / Missing
- Contact with patients, hours - <10 / 10-20 / >20 / Missing
- Confirmed cases of COVID-19 within department – Yes / No / Missing
- Smoking – Yes / No / Missing
- Previous BCG vaccination -No / last BCG dose <1 year ago / 1-5 years ago / >5 years ago / Missing

7.4.4. SUBGROUP ANALYSIS

The following subgroup analyses (described in detail in section 6.4) will be conducted for estimands 7a.1 and 7b.1:

- Sub-Group analysis 1 – Age group (stratification factor at randomisation)
- Sub-Group analyses 2, 2a, 2b, 2c, 2d, 2e – Presence of comorbidities
- Sub-Group analysis 3 – Geographical Location
- Sub-Group analysis 4 – Sex
- Sub-Group analysis 5 – BCG in the past or not

7.5. NUMBER OF DAYS UNABLE TO WORK DUE TO COVID-19 (#8a and #8b)

7.5.1. ESTIMANDS

Objective	Estimand
To determine if BCG vaccination compared with placebo reduces the number of days unable to work due to COVID-19 in the absence of a COVID-19 specific vaccine, measured over 6 months following randomisation in healthcare workers who did not have a previous SARS-CoV-2 positive test result when assessed at time of randomisation.	Estimand 8a.1<br><br>Population: mITT population<br>Outcome: number of days unable to work due to COVID-19 by 6 months<br>Interventions: BCG vs Placebo<br>Handling of Intercurrent events:<br>- COVID-19 specific vaccine (Hypothetical Strategy)<br>- any other vaccine (Treatment Policy strategy)<br>Summary Measure: adjusted* difference in the expected counts
To determine if BCG vaccination compared with placebo reduces the number of days unable to work due to COVID-19 in the absence of a COVID-19 specific vaccine, measured over 12 months following randomisation in healthcare workers who did not have a previous SARS-CoV-2 positive test result when assessed at time of randomisation.	Estimand 8b.1<br><br>Population: mITT population<br>Outcome: number of days unable to work due to COVID-19 by 12 months<br>Interventions: BCG vs Placebo<br>Handling of Intercurrent events:<br>- COVID-19 specific vaccine (Hypothetical Strategy)<br>- any other vaccine (Treatment Policy strategy)<br>Summary Measure: adjusted* difference in the expected counts
To determine if BCG vaccination compared with placebo reduces the number of days unable to work due to COVID-19 irrespective of receiving a COVID-19 specific vaccine or any other vaccine, measured over 6 months following randomisation in healthcare workers who did not have a previous SARS-CoV-2 positive test result when assessed at time of randomisation.	Estimand 8a.2<br><br>Population: as 8a.1<br>Outcome: as 8a.1<br>Interventions: as 8a.1<br>Handling of Intercurrent events:<br>- COVID-19 specific vaccine (Treatment Policy strategy)<br>- any other vaccine (Treatment Policy strategy)<br>Summary Measure: as 8a.1
To determine if BCG vaccination compared with placebo reduces the number of days unable to work due to COVID-19 irrespective of receiving a COVID-19 specific vaccine or any other vaccine, measured	Estimand 8b.2<br><br>Population: as 8b.1<br>Outcome: as 8b.1

Objective	Estimand
over 12 months following randomisation in healthcare workers who did not have a previous SARS-CoV-2 positive test result when assessed at time of randomisation.	Interventions: as 8b.1<br>Handling of Intercurrent events:<br>- COVID-19 specific vaccine (Treatment Policy strategy)<br>- any other vaccine (Treatment Policy strategy)<br>Summary Measure: as 8b.1
To determine if BCG vaccination compared with placebo reduces the number of days unable to work due to COVID-19 illness in the absence of any vaccine, measured over 6 months following randomisation in healthcare workers who did not have a previous SARS-CoV-2 positive test result when assessed at time of randomisation.	Estimand 8a.3<br><br>Population: as 8a.1<br>Outcome: as 8a.1<br>Interventions: as 8a.1<br>Handling of Intercurrent events:<br>- COVID-19 specific vaccine (Hypothetical Strategy)<br>- any other vaccine (Hypothetical Strategy)<br>Summary Measure: as 8a.1
To determine if BCG vaccination compared with placebo reduces the number of days unable to work due to COVID-19 in the absence of any vaccine, measured over 12 months following randomisation in healthcare workers who did not have a previous SARS-CoV-2 positive test result when assessed at time of randomisation.	Estimand 8b.3<br><br>Population: as 8b.1<br>Outcome: as 8b.1<br>Interventions: as 8b.1<br>Handling of Intercurrent events:<br>- COVID-19 specific vaccine (Hypothetical Strategy)<br>- any other vaccine (Hypothetical Strategy)<br>Summary Measure: as 8b.1
** 1) adjusted for stratification factors used at randomisation (age group, presence of comorbidity, and geographical location)*	

7.5.2. ANALYSIS

As per section 7.3.2.

7.5.3. ADDITIONAL ANALYSIS

As per section 7.3.3.

7.5.4. SUBGROUP ANALYSIS

None.

7.6. NUMBER OF DAYS CONFINED TO BED DUE TO COVID-19 (#9a and #9b)

7.6.1.ESTIMANDS

Objective	Estimand
To determine if BCG vaccination compared with placebo reduces the number of days confined to bed due to COVID-19 in the absence of a COVID-19 specific vaccine, measured over 6 months following randomisation in healthcare workers who did not have a previous SARS-CoV-2 positive test result when assessed at time of randomisation.	Estimand 9a.1<br><br>Population: mITT population<br>Outcome: number of days confined to bed due to COVID-19 by 6 months<br>Interventions: BCG vs Placebo<br>Handling of Intercurrent events:<br>- COVID-19 specific vaccine (Hypothetical Strategy)<br>- any other vaccine (Treatment Policy strategy)<br>Summary Measure: adjusted* difference in the expected counts
To determine if BCG vaccination compared with placebo reduces the number of days confined to bed due to COVID-19 in the absence of a COVID-19 specific vaccine, measured over 12 months	Estimand 9b.1<br><br>Population: mITT population<br>Outcome: number of days confined to bed due to COVID-19 by 12 months

Objective	Estimand
following randomisation in healthcare workers who did not have a previous SARS-CoV-2 positive test result when assessed at time of randomisation.	Interventions: BCG vs Placebo<br>Handling of Intercurrent events:<br>- COVID-19 specific vaccine (Hypothetical Strategy)<br>- any other vaccine (Treatment Policy strategy)<br>Summary Measure: adjusted* difference in the expected counts
To determine if BCG vaccination compared with placebo reduces the number of days confined to bed due to COVID-19 irrespective of receiving a COVID-19 specific vaccine or any other vaccine, measured over 6 months following randomisation in healthcare workers who did not have a previous SARS-CoV-2 positive test result when assessed at time of randomisation.	Estimand 9a.2<br><br>Population: as 9a.1<br>Outcome: as 9a.1<br>Interventions: as 9a.1<br>Handling of Intercurrent events:<br>- COVID-19 specific vaccine (Treatment Policy strategy)<br>- any other vaccine (Treatment Policy strategy)<br>Summary Measure: as 9a.1
To determine if BCG vaccination compared with placebo reduces the number of days confined to bed due to COVID-19 irrespective of receiving a COVID-19 specific vaccine or any other vaccine, measured over 12 months following randomisation in healthcare workers who did not have a previous SARS-CoV-2 positive test result when assessed at time of randomisation.	Estimand 9b.2<br><br>Population: as 9b.1<br>Outcome: as 9b.1<br>Interventions: as 9b.1<br>Handling of Intercurrent events:<br>- COVID-19 specific vaccine (Treatment Policy strategy)<br>- any other vaccine (Treatment Policy strategy)<br>Summary Measure: as 9b.1
To determine if BCG vaccination compared with placebo reduces the number of days confined to bed due to COVID-19 illness in the absence of any vaccine, measured over 6 months following randomisation in healthcare workers who did not have a previous SARS-CoV-2 positive test result when assessed at time of randomisation.	Estimand 9a.3<br><br>Population: as 9a.1<br>Outcome: as 9a.1<br>Interventions: as 9a.1<br>Handling of Intercurrent events:<br>- COVID-19 specific vaccine (Hypothetical Strategy)<br>- any other vaccine (Hypothetical Strategy)<br>Summary Measure: as 9a.1
To determine if BCG vaccination compared with placebo reduces the number of days confined to bed due to COVID-19 in the absence of any vaccine, measured over 12 months following randomisation in healthcare workers who did not have a previous SARS-CoV-2 positive test result when assessed at time of randomisation.	Estimand 9b.3<br><br>Population: as 9b.1<br>Outcome: as 9b.1<br>Interventions: as 9b.1<br>Handling of Intercurrent events:<br>- COVID-19 specific vaccine (Hypothetical Strategy)<br>- any other vaccine (Hypothetical Strategy)<br>Summary Measure: as 9b.1
** 1) adjusted for stratification factors used at randomisation (age group, presence of comorbidity, and geographical location)*	

7.6.1. ANALYSIS

As per section 7.3.2.

7.6.2. ADDITIONAL ANALYSIS

As per section 7.3.3.

7.6.3. SUBGROUP ANALYSIS

None.

7.7. NUMBER OF DAYS WITH SYMPTOMS DUE TO COVID-19 (#10a and #10b)

7.7.1. ESTIMANDS

Objective	Estimand
To determine if BCG vaccination compared with placebo reduces the number of days with symptoms due to COVID-19 in the absence of a COVID-19 specific vaccine, measured over 6 months following randomisation in healthcare workers who did not have a previous SARS-CoV-2 positive test result when assessed at time of randomisation.	Estimand 10a.1<br><br>Population: mITT population<br>Outcome: number of days with symptoms due to COVID-19 by 6 months<br>Interventions: BCG vs Placebo<br>Handling of Intercurrent events:<br>- COVID-19 specific vaccine (Hypothetical Strategy)<br>- any other vaccine (Treatment Policy strategy)<br>Summary Measure: adjusted* difference in the expected counts
To determine if BCG vaccination compared with placebo reduces the number of days with symptoms due to COVID-19 in the absence of a COVID-19 specific vaccine, measured over 12 months following randomisation in healthcare workers who did not have a previous SARS-CoV-2 positive test result when assessed at time of randomisation.	Estimand 10b.1<br><br>Population: mITT population<br>Outcome: number of days with symptoms due to COVID-19 by 12 months<br>Interventions: BCG vs Placebo<br>Handling of Intercurrent events:<br>- COVID-19 specific vaccine (Hypothetical Strategy)<br>- any other vaccine (Treatment Policy strategy)<br>Summary Measure: adjusted difference in the expected counts
To determine if BCG vaccination compared with placebo reduces the number of days with symptoms due to COVID-19 irrespective of receiving a COVID-19 specific vaccine or any other vaccine, measured over 6 months following randomisation in healthcare workers who did not have a previous SARS-CoV-2 positive test result when assessed at time of randomisation.	Estimand 10a.2<br><br>Population: as 10a.1<br>Outcome: as 10a.1<br>Interventions: as 10a.1<br>Handling of Intercurrent events:<br>- COVID-19 specific vaccine (Treatment Policy strategy)<br>- any other vaccine (Treatment Policy strategy)<br>Summary Measure: as 10a.1
To determine if BCG vaccination compared with placebo reduces the number of days with symptoms due to COVID-19 irrespective of receiving a COVID-19 specific vaccine or any other vaccine, measured over 12 months following randomisation in healthcare workers who did not have a previous SARS-CoV-2 positive test result when assessed at time of randomisation.	Estimand 10b.2<br><br>Population: as 10b.1<br>Outcome: as 10b.1<br>Interventions: as 10b.1<br>Handling of Intercurrent events:<br>- COVID-19 specific vaccine (Treatment Policy strategy)<br>- any other vaccine (Treatment Policy strategy)<br>Summary Measure: as 10b.1
To determine if BCG vaccination compared with placebo reduces the number of days with symptoms due to COVID-19 illness in the absence of any vaccine, measured over 6 months following randomisation in healthcare workers who did not have a previous SARS-CoV-2 positive test result when assessed at time of randomisation.	Estimand 10a.3<br><br>Population: as 10a.1<br>Outcome: as 10a.1<br>Interventions: as 10a.1<br>Handling of Intercurrent events:<br>- COVID-19 specific vaccine (Hypothetical Strategy)<br>- any other vaccine (Hypothetical Strategy)<br>Summary Measure: as 10a.1
To determine if BCG vaccination compared with placebo reduces the number of days with symptoms due to COVID-19 in the absence of any vaccine, measured over 12 months following randomisation in healthcare workers who did not have a previous	Estimand 10b.3<br><br>Population: as 10b.1<br>Outcome: as 10b.1<br>Interventions: as 10b.1

Objective	Estimand
SARS-CoV-2 positive test result when assessed at time of randomisation.	Handling of Intercurrent events:<br>- COVID-19 specific vaccine (Hypothetical Strategy)<br>- any other vaccine (Hypothetical Strategy)<br>Summary Measure: as 10b.1
** 1) adjusted for stratification factors used at randomisation (age group, presence of comorbidity, and geographical location)*	

7.7.2. ANALYSIS

As per section 7.3.2.

7.7.3. ADDITIONAL ANALYSIS

As per section 7.3.3.

7.7.4. SUBGROUP ANALYSIS

None.

7.8. PNEUMONIA DUE TO COVID-19 (#11a and #11b)

7.8.1. ESTIMANDS

Objective	Estimand
To determine if BCG vaccination compared with placebo reduces the incidence of pneumonia due to COVID-19 in the absence of a COVID vaccine, measured over 6 months following randomisation in healthcare workers who did not have a previous SARS-CoV-2 positive test result when assessed at time of randomisation.	Estimand 11a.1<br><br>Population: mITT population<br>Outcome: pneumonia due to COVID-19 by 6 months<br>Interventions: BCG vs Placebo<br>Handling of Intercurrent events:<br>- COVID-19 specific vaccine (Hypothetical Strategy)<br>- any other vaccine (Treatment Policy strategy)<br>Summary Measure: Adjusted* difference in proportion of participants
To determine if BCG vaccination compared with placebo reduces the incidence of pneumonia due to COVID-19 in the absence of a COVID vaccine, measured over 12 months following randomisation in healthcare workers who did not have a previous SARS-CoV-2 positive test result when assessed at time of randomisation.	Estimand 11b.1<br><br>Population: mITT population<br>Outcome: pneumonia due to COVID-19 by 6 months by 12 months<br>Interventions: BCG vs Placebo<br>Handling of Intercurrent events:<br>- COVID-19 specific vaccine (Hypothetical Strategy)<br>- any other vaccine (Treatment Policy strategy)<br>Summary Measure: Adjusted* difference in proportion of participants
To determine if BCG vaccination compared with placebo reduces the incidence of pneumonia due to COVID-19 irrespective of receiving a COVID-19 specific vaccine or any other vaccine, measured over 6 months following randomisation in healthcare workers who did not have a previous SARS-CoV-2 positive test result when assessed at time of randomisation.	Estimand 11a.2<br><br>Population: as 11a.1<br>Outcome: as 11a.1<br>Interventions: as 11a.1<br>Handling of Intercurrent events:<br>- COVID-19 specific vaccine (Treatment Policy strategy)<br>- any other vaccine (Treatment Policy strategy)<br>Summary Measure: as 11a.1
To determine if BCG vaccination compared with placebo reduces the incidence of pneumonia due to COVID-19 irrespective of receiving a COVID-19 specific vaccine or any other vaccine, measured over 12 months following randomisation in healthcare	Estimand 11b.2<br><br>Population: as 11b.1<br>Outcome: as 11b.1<br>Interventions: as 11b.1

Objective	Estimand
workers who did not have a previous SARS-CoV-2 positive test result when assessed at time of randomisation.	Handling of Intercurrent events:<br>- COVID-19 specific vaccine (Treatment Policy strategy)<br>- any other vaccine (Treatment Policy strategy)<br>Summary Measure: as 11b.1
To determine if BCG vaccination compared with placebo reduces the incidence of pneumonia due to COVID-19 illness in the absence of any vaccine, measured over 6 months following randomisation in healthcare workers who did not have a previous SARS-CoV-2 positive test result when assessed at time of randomisation.	Estimand 11a.3<br><br>Population: as 11a.1<br>Outcome: as 11a.1<br>Interventions: as 11a.1<br>Handling of Intercurrent events:<br>- COVID-19 specific vaccine (Hypothetical Strategy)<br>- any other vaccine (Hypothetical Strategy)<br>Summary Measure: as 11a.1
To determine if BCG vaccination compared with placebo reduces the incidence of pneumonia due to COVID-19 in the absence of any vaccine, measured over 12 months following randomisation in healthcare workers who did not have a previous SARS-CoV-2 positive test result when assessed at time of randomisation.	Estimand 11b.3<br><br>Population: as 11b.1<br>Outcome: as 11b.1<br>Interventions: as 11b.1<br>Handling of Intercurrent events:<br>- COVID-19 specific vaccine (Hypothetical Strategy)<br>- any other vaccine (Hypothetical Strategy)<br>Summary Measure: as 11b.1
** 1) adjusted for stratification factors used at randomisation (age group, presence of comorbidity, and geographical location)*	

7.8.2. ANALYSIS

Absolute and relative frequencies of pneumonia prior to 6/12 months will be presented by intervention group. The outcome will be compared between the BCG group and the placebo group using a difference in proportions estimated using the same time-to-event analysis approach adopted for primary outcomes 1 and 2 (flexible parametric survival model), adjusted by the stratification factors used at randomisation. A two-sided bias-corrected 95% CI for the difference in proportion (BCG – Control) and a bootstrap p-value will be calculated with bootstrap standard errors. A Kaplan-Meier survival curve will also be presented by treatment arm for descriptive purposes.

For participants that meet the outcome the date of the first day with symptoms associated to a symptomatic/severe COVID-19 event will be taken as the outcome. Participants who did not have COVID-19 within the first 6/12 months on the study will be censored according to the same rules used for the censoring of primary outcomes 1 and 2 (section 6.2.3).

Should the overall number of pneumonia events be so small that the analysis model described has computational difficulties, the outcome will be analysed using the Cox's proportional hazards model, adjusted for the stratification factors used during randomisation (age group, presence of comorbidity, geographical location -Europe/Australia/South America), and presented as the hazard ratio rather than the difference in proportion.

The analysis will be adjusted for the stratification factors only if a minimum number of events is observed, namely 1 event per strata per intervention group.

7.8.3. ADDITIONAL ANALYSIS

None.

7.8.4. SUBGROUP ANALYSIS

None.

7.9. NEED OF OXYGEN DUE TO COVID-19 (#12a and #12b)

7.9.1. ESTIMANDS

Objective	Estimand
To determine if BCG vaccination compared with placebo reduces the need for oxygen therapy due to COVID-19 in the absence of a COVID vaccine, measured over 6 months following randomisation in healthcare workers who did not have a previous SARS-CoV-2 positive test result when assessed at time of randomisation.	Estimand 12a.1<br><br>Population: mITT population<br>Outcome: need of oxygen therapy due to COVID-19 by 6 months<br>Interventions: BCG vs Placebo<br>Handling of Intercurrent events:<br>- COVID-19 specific vaccine (Hypothetical Strategy)<br>- any other vaccine (Treatment Policy strategy)<br>Summary Measure: Adjusted* difference in proportion of participants
To determine if BCG vaccination compared with placebo reduces the need for oxygen therapy due to COVID-19 in the absence of a COVID vaccine, measured over 12 months following randomisation in healthcare workers who did not have a previous SARS-CoV-2 positive test result when assessed at time of randomisation.	Estimand 12b.1<br><br>Population: mITT population<br>Outcome: need of oxygen therapy due to COVID-19 by 12 months<br>Interventions: BCG vs Placebo<br>Handling of Intercurrent events:<br>- COVID-19 specific vaccine (Hypothetical Strategy)<br>- any other vaccine (Treatment Policy strategy)<br>Summary Measure: Adjusted* difference in proportion of participants
To determine if BCG vaccination compared with placebo reduces the need for oxygen therapy due to COVID-19 irrespective of receiving a COVID-19 specific vaccine or any other vaccine, measured over 6 months following randomisation in healthcare workers who did not have a previous SARS-CoV-2 positive test result when assessed at time of randomisation.	Estimand 12a.2<br><br>Population: as 12a.1<br>Outcome: as 12a.1<br>Interventions: as 12a.1<br>Handling of Intercurrent events:<br>- COVID-19 specific vaccine (Treatment Policy strategy)<br>- any other vaccine (Treatment Policy strategy)<br>Summary Measure: as 12a.1
To determine if BCG vaccination compared with placebo reduces the need for oxygen therapy due to COVID-19 irrespective of receiving a COVID-19 specific vaccine or any other vaccine, measured over 12 months following randomisation in healthcare workers who did not have a previous SARS-CoV-2 positive test result when assessed at time of randomisation.	Estimand 12b.2<br><br>Population: as 12b.1<br>Outcome: as 12b.1<br>Interventions: as 12b.1<br>Handling of Intercurrent events:<br>- COVID-19 specific vaccine (Treatment Policy strategy)<br>- any other vaccine (Treatment Policy strategy)<br>Summary Measure: as 12b.1
To determine if BCG vaccination compared with placebo reduces the need for oxygen therapy due to COVID-19 illness in the absence of any vaccine, measured over 6 months following randomisation in healthcare workers who did not have a previous SARS-CoV-2 positive test result when assessed at time of randomisation.	Estimand 12a.3<br><br>Population: as 12a.1<br>Outcome: as 12a.1<br>Interventions: as 12a.1<br>Handling of Intercurrent events:<br>- COVID-19 specific vaccine (Hypothetical Strategy)<br>- any other vaccine (Hypothetical Strategy)<br>Summary Measure: as 12a.1
To determine if BCG vaccination compared with placebo reduces the need for oxygen therapy due to COVID-19 in the absence of any vaccine, measured over 12 months following randomisation in healthcare workers who did not have a previous	Estimand 12b.3<br><br>Population: as 12b.1<br>Outcome: as 12b.1<br>Interventions: as 12b.1

Objective	Estimand
SARS-CoV-2 positive test result when assessed at time of randomisation.	Handling of Intercurrent events:<br>- COVID-19 specific vaccine (Hypothetical Strategy)<br>- any other vaccine (Hypothetical Strategy)<br>Summary Measure: as 12b.1
** 1) adjust by stratification factors used at randomisation;*	

7.9.2. ANALYSIS

As per section 7.8.2.

7.9.3. ADDITIONAL ANALYSIS

On the subset of participants who needed oxygen due to severe COVID-19, the mean and standard deviation (or median and IQR if not normally distributed) of the duration of oxygen therapy will be calculated and presented by intervention group.

7.9.4. SUBGROUP ANALYSIS

None.

7.10. ADMISSION TO CRITICAL CARE DUE TO COVID-19 (#13a and #13b)

7.10.1. ESTIMANDS

Objective	Estimand
To determine if BCG vaccination compared with placebo reduces admission to critical care DUE TO COVID-19 in the absence of a COVID vaccine, measured over 6 months following randomisation in healthcare workers who did not have a previous SARS-CoV-2 positive test result when assessed at time of randomisation.	Estimand 13a.1<br><br>Population: mITT population<br>Outcome: admission to critical care due to COVID-19 by 6 months<br>Interventions: BCG vs Placebo<br>Handling of Intercurrent events:<br>- COVID-19 specific vaccine (Hypothetical Strategy)<br>- any other vaccine (Treatment Policy strategy)<br>Summary Measure: Adjusted* difference in proportion of participants
To determine if BCG vaccination compared with placebo reduces admission to critical care DUE TO COVID-19 in the absence of a COVID vaccine, measured over 12 months following randomisation in healthcare workers who did not have a previous SARS-CoV-2 positive test result when assessed at time of randomisation.	Estimand 13b.1<br><br>Population: mITT population<br>Outcome: admission to critical care due to COVID-19 by 12 months<br>Interventions: BCG vs Placebo<br>Handling of Intercurrent events:<br>- COVID-19 specific vaccine (Hypothetical Strategy)<br>- any other vaccine (Treatment Policy strategy)<br>Summary Measure: Adjusted* difference in proportion of participants
To determine if BCG vaccination compared with placebo reduces admission to critical care due to COVID-19 irrespective of receiving a COVID-19 specific vaccine or any other vaccine, measured over 6 months following randomisation in healthcare workers who did not have a previous SARS-CoV-2 positive test result when assessed at time of randomisation.	Estimand 13a.2<br><br>Population: as 13a.1<br>Outcome: as 13a.1<br>Interventions: as 13a.1<br>Handling of Intercurrent events:<br>- COVID-19 specific vaccine (Treatment Policy strategy)<br>- any other vaccine (Treatment Policy strategy)<br>Summary Measure: as 13a.1
To determine if BCG vaccination compared with placebo reduces admission to critical care due to COVID-19 irrespective of receiving a COVID-19 specific vaccine or any other vaccine, measured over 12 months following randomisation in healthcare	Estimand 13b.2<br><br>Population: as 13b.1<br>Outcome: as 13b.1<br>Interventions: as 13b.1

Objective	Estimand
workers who did not have a previous SARS-CoV-2 positive test result when assessed at time of randomisation.	Handling of Intercurrent events:<br>- COVID-19 specific vaccine (Treatment Policy strategy)<br>- any other vaccine (Treatment Policy strategy)<br>Summary Measure: as 13b.1
To determine if BCG vaccination compared with placebo reduces admission to critical care due to COVID-19 illness in the absence of any vaccine, measured over 6 months following randomisation in healthcare workers who did not have a previous SARS-CoV-2 positive test result when assessed at time of randomisation.	Estimand 13a.3<br><br>Population: as 13a.1<br>Outcome: as 13a.1<br>Interventions: as 13a.1<br>Handling of Intercurrent events:<br>- COVID-19 specific vaccine (Hypothetical Strategy)<br>- any other vaccine (Hypothetical Strategy)<br>Summary Measure: as 13a.1
To determine if BCG vaccination compared with placebo reduces admission to critical care due to COVID-19 in the absence of any vaccine, measured over 12 months following randomisation in healthcare workers who did not have a previous SARS-CoV-2 positive test result when assessed at time of randomisation.	Estimand 13b.3<br><br>Population: as 13b.1<br>Outcome: as 13b.1<br>Interventions: as 13b.1<br>Handling of Intercurrent events:<br>- COVID-19 specific vaccine (Hypothetical Strategy)<br>- any other vaccine (Hypothetical Strategy)<br>Summary Measure: as 13b.1
** 1) adjust by stratification factors used at randomisation;*	

7.10.2. PRIMARY ANALYSIS

As per section 7.8.2.

7.10.3. ADDITIONAL ANALYSIS

In the those of participants who were admitted to critical care due to severe COVID-19, the mean and standard deviation (or median and IQR if distribution is skewed) of the duration of critical care will be calculated and presented by intervention group.

7.10.4. SUBGROUP ANALYSIS

None.

7.11. NEED OF MECHANICAL VENTILATION DUE TO COVID-19 (#14a and #14b)

7.11.1. ESTIMANDS

Objective	Estimand
To determine if BCG vaccination compared with placebo reduces the need of mechanical ventilation due to COVID-19 in the absence of a COVID vaccine, measured over 6 months following randomisation in healthcare workers who did not have a previous SARS-CoV-2 positive test result when assessed at time of randomisation.	Estimand 14a.1<br><br>Population: mITT population<br>Outcome: need of mechanical ventilation due to COVID-19 by 6 months<br>Interventions: BCG vs Placebo<br>Handling of Intercurrent events:<br>- COVID-19 specific vaccine (Hypothetical Strategy)<br>- any other vaccine (Treatment Policy strategy)<br>Summary Measure: Adjusted* difference in proportion of participants
To determine if BCG vaccination compared with placebo reduces the need of mechanical ventilation DUE TO COVID-19 in the absence of a COVID vaccine, measured over 12 months following	Estimand 14b.1<br><br>Population: mITT population<br>Outcome: need of mechanical ventilation due to COVID-19 by 12 months

Objective	Estimand
randomisation in healthcare workers who did not have a previous SARS-CoV-2 positive test result when assessed at time of randomisation.	Interventions: BCG vs Placebo<br>Handling of Intercurrent events:<br>- COVID-19 specific vaccine (Hypothetical Strategy)<br>- any other vaccine (Treatment Policy strategy)<br>Summary Measure: Adjusted* difference in proportion of participants
To determine if BCG vaccination compared with placebo reduces the need of mechanical ventilation due to COVID-19 irrespective of receiving a COVID-19 specific vaccine or any other vaccine, measured over 6 months following randomisation in healthcare workers who did not have a previous SARS-CoV-2 positive test result when assessed at time of randomisation.	Estimand 14a.2<br><br>Population: as 14a.1<br>Outcome: as 14a.1<br>Interventions: as 14a.1<br>Handling of Intercurrent events:<br>- COVID-19 specific vaccine (Treatment Policy strategy)<br>- any other vaccine (Treatment Policy strategy)<br>Summary Measure: as 14a.1
To determine if BCG vaccination compared with placebo reduces the need of mechanical ventilation due to COVID-19 irrespective of receiving a COVID-19 specific vaccine or any other vaccine, measured over 12 months following randomisation in healthcare workers who did not have a previous SARS-CoV-2 positive test result when assessed at time of randomisation.	Estimand 14b.2<br><br>Population: as 14b.1<br>Outcome: as 14b.1<br>Interventions: as 14b.1<br>Handling of Intercurrent events:<br>- COVID-19 specific vaccine (Treatment Policy strategy)<br>- any other vaccine (Treatment Policy strategy)<br>Summary Measure: as 14b.1
To determine if BCG vaccination compared with placebo reduces the need of mechanical ventilation due to COVID-19 illness in the absence of any vaccine, measured over 6 months following randomisation in healthcare workers who did not have a previous SARS-CoV-2 positive test result when assessed at time of randomisation.	Estimand 14a.3<br><br>Population: as 14a.1<br>Outcome: as 14a.1<br>Interventions: as 14a.1<br>Handling of Intercurrent events:<br>- COVID-19 specific vaccine (Hypothetical Strategy)<br>- any other vaccine (Hypothetical Strategy)<br>Summary Measure: as 14a.1
To determine if BCG vaccination compared with placebo reduces the need of mechanical ventilation due to COVID-19 in the absence of any vaccine, measured over 12 months following randomisation in healthcare workers who did not have a previous SARS-CoV-2 positive test result when assessed at time of randomisation.	Estimand 14b.3<br><br>Population: as 14b.1<br>Outcome: as 14b.1<br>Interventions: as 14b.1<br>Handling of Intercurrent events:<br>- COVID-19 specific vaccine (Hypothetical Strategy)<br>- any other vaccine (Hypothetical Strategy)<br>Summary Measure: as 14b.1
** 1) adjust by stratification factors used at randomisation;*	

7.11.2. ANALYSIS

As per section 7.8.2.

7.11.3. ADDITIONAL ANALYSIS

In participants who needed MV due to severe COVID-19, the mean and standard deviation (or median and IQR if distribution is skewed) of the duration of critical care will be calculated and presented by intervention group.

7.11.4. SUBGROUP ANALYSIS

None.

7.12. HOSPITALISATION DUE TO COVID-19 (#15a and #15b)

7.12.1. ESTIMANDS

Objective	Estimand
To determine if BCG vaccination compared with placebo reduces the incidence of hospitalisation due to COVID-19 in the absence of a COVID vaccine, measured over 6 months following randomisation in healthcare workers who did not have a previous SARS-CoV-2 positive test result when assessed at time of randomisation.	Estimand 15a.1<br><br>Population: mITT population<br>Outcome: hospitalisation due to COVID-19 by 6 months<br>Interventions: BCG vs Placebo<br>Handling of Intercurrent events:<br>- COVID-19 specific vaccine (Hypothetical Strategy)<br>- any other vaccine (Treatment Policy strategy)<br>Summary Measure: Adjusted* difference in proportion of participants
To determine if BCG vaccination compared with placebo reduces the incidence of hospitalisation due to COVID-19 in the absence of a COVID vaccine, measured over 12 months following randomisation in healthcare workers who did not have a previous SARS-CoV-2 positive test result when assessed at time of randomisation.	Estimand 15b.1<br><br>Population: mITT population<br>Outcome: hospitalisation due to COVID-19 by 12 months<br>Interventions: BCG vs Placebo<br>Handling of Intercurrent events:<br>- COVID-19 specific vaccine (Hypothetical Strategy)<br>- any other vaccine (Treatment Policy strategy)<br>Summary Measure: Adjusted* difference in proportion of participants
To determine if BCG vaccination compared with placebo reduces the incidence of hospitalisation due to COVID-19irrespective of receiving a COVID-19 specific vaccine or any other vaccine, measured over 6 months following randomisation in healthcare workers who did not have a previous SARS-CoV-2 positive test result when assessed at time of randomisation.	Estimand 15a.2<br><br>Population: as 15a.1<br>Outcome: as 15a.1<br>Interventions: as 15a.1<br>Handling of Intercurrent events:<br>- COVID-19 specific vaccine (Treatment Policy strategy)<br>- any other vaccine (Treatment Policy strategy)<br>Summary Measure: as 15a.1
To determine if BCG vaccination compared with placebo reduces the incidence of hospitalisation due to COVID-19 irrespective of receiving a COVID-19 specific vaccine or any other vaccine, measured over 12 months following randomisation in healthcare workers who did not have a previous SARS-CoV-2 positive test result when assessed at time of randomisation.	Estimand 15b.2<br><br>Population: as 15b.1<br>Outcome: as 15b.1<br>Interventions: as 15b.1<br>Handling of Intercurrent events:<br>- COVID-19 specific vaccine (Treatment Policy strategy)<br>- any other vaccine (Treatment Policy strategy)<br>Summary Measure: as 15b.1
To determine if BCG vaccination compared with placebo reduces the incidence of hospitalisation due to COVID-19 illness in the absence of any vaccine, measured over 6 months following randomisation in healthcare workers who did not have a previous SARS-CoV-2 positive test result when assessed at time of randomisation.	Estimand 15a.3<br><br>Population: as 15a.1<br>Outcome: as 15a.1<br>Interventions: as 15a.1<br>Handling of Intercurrent events:<br>- COVID-19 specific vaccine (Hypothetical Strategy)<br>- any other vaccine (Hypothetical Strategy)<br>Summary Measure: as 15a.1
To determine if BCG vaccination compared with placebo reduces the incidence of hospitalisation due	Estimand 15b.3

Objective	Estimand
to COVID-19 in the absence of any vaccine, measured over 12 months following randomisation in healthcare workers who did not have a previous SARS-CoV-2 positive test result when assessed at time of randomisation.	Population: as 15b.1<br>Outcome: as 15b.1<br>Interventions: as 15b.1<br>Handling of Intercurrent events:<br>- COVID-19 specific vaccine (Hypothetical Strategy)<br>- any other vaccine (Hypothetical Strategy)<br>Summary Measure: as 15b.1
** 1) adjust by stratification factors used at randomisation;*	

7.12.2. ANALYSIS

As per section 7.8.2.

7.12.3. ADDITIONAL ANALYSIS

In participants who were admitted to hospital due to COVID-19, the mean and standard deviation (or median and IQR if not normally distributed) of the hospital stay will be calculated and presented by intervention group.

7.12.4. SUBGROUP ANALYSIS

None.

7.13. DEATH DUE TO COVID-19 (#16a and #16b)

7.13.1. ESTIMANDS

Objective	Estimand
To determine if BCG vaccination compared with placebo reduces the incidence of death due to COVID-19 in the absence of a COVID vaccine, measured over 6 months following randomisation in healthcare workers who did not have a previous SARS-CoV-2 positive test result when assessed at time of randomisation.	Estimand 16a.1<br><br>Population: mITT population<br>Outcome: death due to COVID-19 by 6 months<br>Interventions: BCG vs Placebo<br>Handling of Intercurrent events:<br>- COVID-19 specific vaccine (Hypothetical Strategy)<br>- any other vaccine (Treatment Policy strategy)<br>Summary Measure: Adjusted* difference in proportion of participants
To determine if BCG vaccination compared with placebo reduces the incidence of death due to COVID-19 in the absence of a COVID vaccine, measured over 12 months following randomisation in healthcare workers who did not have a previous SARS-CoV-2 positive test result when assessed at time of randomisation.	Estimand 16b.1<br><br>Population: mITT population<br>Outcome: death due to COVID-19 by 12 months<br>Interventions: BCG vs Placebo<br>Handling of Intercurrent events:<br>- COVID-19 specific vaccine (Hypothetical Strategy)<br>- any other vaccine (Treatment Policy strategy)<br>Summary Measure: Adjusted* difference in proportion of participants
To determine if BCG vaccination compared with placebo reduces the incidence of death due to COVID-19irrespective of receiving a COVID-19 specific vaccine or any other vaccine, measured over 6 months following randomisation in healthcare workers who did not have a previous SARS-CoV-2 positive test result when assessed at time of randomisation.	Estimand 16a.2<br><br>Population: as 16a.1<br>Outcome: as 16a.1<br>Interventions: as 16a.1<br>Handling of Intercurrent events:<br>- COVID-19 specific vaccine (Treatment Policy strategy)<br>- any other vaccine (Treatment Policy strategy)<br>Summary Measure: as 16a.1

Objective	Estimand
To determine if BCG vaccination compared with placebo reduces the incidence of death due to COVID-19 irrespective of receiving a COVID-19 specific vaccine or any other vaccine, measured over 12 months following randomisation in healthcare workers who did not have a previous SARS-CoV-2 positive test result when assessed at time of randomisation.	Estimand 16b.2<br><br>Population: as 16b.1<br>Outcome: as 16b.1<br>Interventions: as 16b.1<br>Handling of Intercurrent events:<br>- COVID-19 specific vaccine (Treatment Policy strategy)<br>- any other vaccine (Treatment Policy strategy)<br>Summary Measure: as 16b.1
To determine if BCG vaccination compared with placebo reduces the incidence of death due to COVID-19 illness in the absence of any vaccine, measured over 6 months following randomisation in healthcare workers who did not have a previous SARS-CoV-2 positive test result when assessed at time of randomisation.	Estimand 16a.3<br><br>Population: as 16a.1<br>Outcome: as 16a.1<br>Interventions: as 16a.1<br>Handling of Intercurrent events:<br>- COVID-19 specific vaccine (Hypothetical Strategy)<br>- any other vaccine (Hypothetical Strategy)<br>Summary Measure: as 16a.1
To determine if BCG vaccination compared with placebo reduces the incidence of death due to COVID-19 in the absence of any vaccine, measured over 12 months following randomisation in healthcare workers who did not have a previous SARS-CoV-2 positive test result when assessed at time of randomisation.	Estimand 16b.3<br><br>Population: as 16b.1<br>Outcome: as 16b.1<br>Interventions: as 16b.1<br>Handling of Intercurrent events:<br>- COVID-19 specific vaccine (Hypothetical Strategy)<br>- any other vaccine (Hypothetical Strategy)<br>Summary Measure: as 16b.1
** 1) adjust by stratification factors used at randomisation;*	

7.13.2. ANALYSIS
Same as per 7.8.2.

7.13.3. ADDITIONAL ANALYSIS
None.

7.13.4. SUBGROUP ANALYSIS
None.

8. NON-COVID19 RELATED SECONDARY OUTCOMES
For these secondary outcomes, the intercurrent events of receiving any vaccine (including a COVID-19-specific vaccine) will be handled using a Hypothetical Strategy; participants who receive any vaccine (including a COVID-19-specific vaccine) will have their data used up to the date of their first dose of vaccine (data collected after the first dose of the vaccine will be ignored). In a secondary analysis, these intercurrent events will be handled using a treatment policy strategy, and all outcome data will be used regardless of occurrence of the intercurrent event. The analyses of these secondary outcomes will be conducted on the ITT population including only Stage 2 participants. No subgroup analyses for any of these outcomes will be carried out.

8.1. FEVER OR RESPIRATORY ILLNESS (#17)

8.1.1. ESTIMANDS

Objective	Estimand
To determine if BCG vaccination compared with placebo reduces the incidence of fever or respiratory illness in the absence of any vaccine (including COVID-19 specific vaccine), over the 12 months following randomisation, in healthcare workers exposed to SARS-CoV-2.	Estimand 17.1<br><br>Population: ITT population<br>Outcome: fever or respiratory illness by 12 months<br>Interventions: BCG vs Placebo<br>Handling of Intercurrent events:<br>- any vaccine, including COVID-19 specific vaccine (Hypothetical strategy)<br>Summary Measure: Adjusted difference in proportion of participants
To determine if BCG vaccination compared with placebo reduces the incidence of fever or respiratory illness irrespective of receiving any vaccine (including COVID-19 specific vaccine), over the 12 months following randomisation, in healthcare workers exposed to SARS-CoV-2.	Estimand 17.2<br><br>Population: as for estimand 17.1<br>Outcome: as for estimand 17.1<br>Interventions: as for estimand 17.1<br>Handling of Intercurrent events:<br>- any vaccine, including COVID-19 specific vaccine (Treatment Policy strategy)<br>Summary Measure: as for estimand 17.1

8.1.2. ANALYSIS

The outcome of respiratory or febrile illness prior to 12 months will be described by intervention group as the absolute number of participants with the event.
The numbers of participants whose follow-up data is censored due to:

- incomplete data entry,
- drop-out from the study, and
- intercurrent event (any vaccine, including COVID-19 specific vaccine)

will also be reported separately by intervention group.

Participants with complete data entry (i.e. complete APP weekly data and/or survey data) will be censored:

- (for estimand 17.1) at the earlier of their first dose of any vaccine given during the 12 months follow-up or 12 months unless the definition of the outcome is met and precedes both of these dates.
- (for estimand 17.2) at the 12 months follow-up or 12 months unless the definition of the outcome is met and precedes this date.

Participants with incomplete data entry (i.e. incomplete APP weekly data and incomplete survey data) will be censored at the earlier of:

- their first dose of any vaccine given during the 12 months follow-up (for estimand 17.1 only) or
- their last entered date prior to which there are more than 3 consecutive days of missing data following the last

unless the definition of the outcome is met and precedes both of these dates.

When the definition of the outcome is met, the first day with symptoms for the first episode of fever or respiratory illness will be used in the analysis.

The analysis of this outcome will be conducted as for the primary analysis of the primary outcome 1 and 2 (as presented in section 6.1.1). This outcome will be compared between the BCG group and the placebo group using a difference in proportions. This will be estimated using a time-to-event analysis adjusted for stratification factors used in randomisation, with the survival curve for each combination of strata and randomised group calculated using a flexible parametric survival model (Royston-Parmar model[2]).
A Kaplan-Meier survival curve will also be presented by treatment arm.

8.2. SEVERE FEVER OR RESPIRATORY ILLNESS (#18)

8.2.1. ESTIMANDS

Objective	Estimand
To determine if BCG vaccination compared with placebo reduces the incidence of severe fever or respiratory illness in the absence of any vaccine (including COVID-19 specific vaccine), over the 12 months following randomisation, in healthcare workers exposed to SARS-CoV-2.	Estimand 18.1<br><br>Population: ITT population<br>Outcome: severe fever or respiratory illness by 12 months<br>Interventions: BCG vs Placebo<br>Handling of Intercurrent events:<br>- any vaccine, including COVID-19 specific vaccine (Hypothetical strategy)<br>Summary Measure: Adjusted difference in proportion of participants
To determine if BCG vaccination compared with placebo reduces the incidence of severe fever or respiratory illness irrespective of receiving any vaccine (including COVID-19 specific vaccine), over the 12 months following randomisation, in healthcare workers exposed to SARS-CoV-2.	Estimand 18.2<br><br>Population: as for estimand 18.1<br>Outcome: as for estimand 18.1<br>Interventions: as for estimand 18.1<br>Handling of Intercurrent events:<br>- any vaccine, including COVID-19 specific vaccine (Treatment Policy strategy)<br>Summary Measure: as for estimand 18.1

8.2.2. ANALYSIS

As per section 8.2.1.

8.3. NUMBER OF EPISODES OF FEVER OR RESPIRATORY ILLNESS (#19)

8.3.1. ESTIMANDS

Objective	Estimand
To determine if BCG vaccination compared with placebo reduces the number of fever or respiratory illness in the absence of any vaccine (including COVID-19 specific vaccine), over the 12 months following randomisation, in healthcare workers exposed to SARS-CoV-2.	Estimand 19.1<br><br>Population: ITT population<br>Outcome: number of fever or respiratory illness by 12 months<br>Interventions: BCG vs Placebo<br>Handling of Intercurrent events:<br>- any vaccine, including COVID-19 specific vaccine (Hypothetical strategy)<br>Summary Measure: difference in the expected counts
To determine if BCG vaccination compared with placebo reduces the number of fever or respiratory illness irrespective of receiving any vaccine (including COVID-19 specific vaccine), over the 12 months following randomisation, in healthcare workers exposed to SARS-CoV-2.	Estimand 19.2<br><br>Population: as for estimand 19.1<br>Outcome: as for estimand 19.1<br>Interventions: as for estimand 19.1<br>Handling of Intercurrent events:<br>- any vaccine, including COVID-19 specific vaccine (Treatment Policy strategy)<br>Summary Measure: as for estimand 19.1

8.3.1. ANALYSIS

Median and IQR of the number of episodes will be calculated and presented by intervention group.
The difference between the BCG and Placebo groups in the number of episodes and its 95%CI will be estimated using a ZIP model, adjusted by the stratification factors used at randomisation (Geographical Location (Australia/Europe/South America, age group and presence of comorbidity). For further details on the ZIP model refer to section 7.3.2. Particularly the *exposure()* option will be used to indicate the number of days elapsed from the date of randomisation to:

- the date of the 12 months follow-up or the date of first dose of any vaccine (for estimand 18.1), whichever is earlier, for those participants with complete data entry
- the date of the last contact with the participant or the date of first dose of any vaccine (for estimand 18.1), whichever is earliest, for those participants with incomplete data entry

8.4. NUMBER OF DAYS UNABLE TO WORK DUE TO FEVER OR RESPIRATORY ILLNESS (#20)

8.4.1. ESTIMANDS

Objective	Estimand
To determine if BCG vaccination compared with placebo reduces the number of days unable to work due to fever or respiratory illness in the absence of any vaccine (including COVID-19 specific vaccine), over the 12 months following randomisation, in healthcare workers exposed to SARS-CoV-2.	Estimand 20.1<br><br>Population: ITT population<br>Outcome: number days unable to work due to fever or respiratory illness by 12 months<br>Interventions: BCG vs Placebo<br>Handling of Intercurrent events:<br>- any vaccine, including COVID-19 specific vaccine (Hypothetical strategy)<br>Summary Measure: difference in the expected counts
To determine if BCG vaccination compared with placebo reduces the number of days unable to work due to fever or respiratory illness irrespective of receiving any vaccine (including COVID-19 specific vaccine), over the 12 months following randomisation, in healthcare workers exposed to SARS-CoV-2.	Estimand 19.2<br><br>Population: as for estimand 20.1<br>Outcome: as for estimand 20.1<br>Interventions: as for estimand 20.1<br>Handling of Intercurrent events:<br>- any vaccine, including COVID-19 specific vaccine (Treatment Policy strategy)<br>Summary Measure: as for estimand 20.1

8.4.2. ANALYSIS

As per section 8.2.1.

8.5. NUMBER OF DAYS CONFINED TO BED DUE TO FEVER OR RESPIRATORY ILLNESS (#21)

8.5.1. ESTIMANDS

Objective	Estimand
To determine if BCG vaccination compared with placebo reduces the number of days confined to bed due to fever or respiratory illness in the absence of any vaccine (including COVID-19 specific vaccine), over the 12 months following randomisation, in healthcare workers exposed to SARS-CoV-2.	Estimand 21.1<br><br>Population: ITT population<br>Outcome: number days confined to bed due fever or respiratory illness by 12 months<br>Interventions: BCG vs Placebo<br>Handling of Intercurrent events:<br>- any vaccine, including COVID-19 specific vaccine (Hypothetical strategy)<br>Summary Measure: difference in the expected counts
To determine if BCG vaccination compared with placebo reduces the number of days confined to bed due to fever or respiratory illness irrespective of receiving any vaccine (including COVID-19 specific vaccine), over the 12 months following randomisation, in healthcare workers exposed to SARS-CoV-2.	Estimand 21.2<br><br>Population: as for estimand 21.1<br>Outcome: as for estimand 21.1<br>Interventions: as for estimand 21.1<br>Handling of Intercurrent events:<br>- any vaccine, including COVID-19 specific vaccine (Treatment Policy strategy)

Objective	Estimand
	Summary Measure: as for estimand 21.1

8.5.2. ANALYSIS

As per section 8.2.1.

8.6. NUMBER OF DAYS WITH SYMPTOMS DUE TO FEVER OR RESPIRATORY ILLNESS (#22)

8.6.1. ESTIMANDS

Objective	Estimand
To determine if BCG vaccination compared with placebo reduces the number of days with symptoms due to fever or respiratory illness in the absence of any vaccine (including COVID-19 specific vaccine), over the 12 months following randomisation, in healthcare workers exposed to SARS-CoV-2.	Estimand 22.1<br><br>Population: ITT population<br>Outcome: number days with symptoms due fever or respiratory illness by 12 months<br>Interventions: BCG vs Placebo<br>Handling of Intercurrent events:<br>- any vaccine, including COVID-19 specific vaccine (Hypothetical strategy)<br>Summary Measure: difference in the expected counts
To determine if BCG vaccination compared with placebo reduces the number of days with symptoms due to fever or respiratory illness irrespective of receiving any vaccine (including COVID-19 specific vaccine), over the 12 months following randomisation, in healthcare workers exposed to SARS-CoV-2.	Estimand 22.2<br><br>Population: as for estimand 22.1<br>Outcome: as for estimand 22.1<br>Interventions: as for estimand 22.1<br>Handling of Intercurrent events:<br>- any vaccine, including COVID-19 specific vaccine (Treatment Policy strategy)<br>Summary Measure: as for estimand 22.1

8.6.2. ANALYSIS

As per section 8.2.1.

8.7. PNEUMONIA (#23)

8.7.1. ESTIMANDS

Objective	Estimand
To determine if BCG vaccination compared with placebo reduces the incidence of pneumonia in the absence of any vaccine, including COVID-19 specific vaccine , measured over 12 months following randomisation in healthcare exposed to SARS-CoV-2.	Estimand 23.1<br><br>Population: ITT population<br>Outcome: pneumonia by 12 months<br>Interventions: BCG vs Placebo<br>Handling of Intercurrent events:<br>- any vaccine, including COVID-19 specific vaccine (Hypothetical strategy)<br>Summary Measure: Adjusted difference in proportion of participants
To determine if BCG vaccination compared with placebo reduces the incidence of pneumonia irrespective of receiving any other vaccine (including COVID-19 specific vaccine), over the 12 months following randomisation, in healthcare workers exposed to SARS-CoV-2.	Estimand 23.2<br><br>Population: as for estimand 23.1<br>Outcome: as for estimand 23.1<br>Interventions: as for estimand 23.1<br>Handling of Intercurrent events:<br>- any vaccine, including COVID-19 specific vaccine (Treatment policy)<br>Summary Measure: as for estimand 23.1

8.7.2. ANALYSIS

The absolute and relative frequencies of participants with the outcome prior to 12 months will be presented by intervention group. The outcome will be compared between the BCG group and the placebo group using a difference in proportions estimated using the same time-to-event analysis approach adopted for primary outcomes 1 and 2 (flexible parametric survival model). A two-sided bias-corrected 95% CI for the difference in proportion (BCG – Control) and a bootstrap p-value will be calculated with bootstrap standard errors. A Kaplan-Meier survival curve will also be presented by treatment arm for descriptive purposes.
For participants that meet the outcome the date of the first day with symptoms associated to a febrile or respiratory illness will be taken as the outcome. Participants who did not have pneumonia within 12 months will be censored according to the same rules used for the censoring of outcomes 17 (section 8.1.2).

Should the overall number of pneumonia events be so small that the analysis model described has computational difficulties, the outcome will be analysed using the Cox's proportional hazards model, adjusted for the stratification factors used during randomisation (age group, presence of comorbidity, geographical location -Europe/Australia/South America), and presented as the hazard ratio rather than the difference in proportion.

The analysis will be adjusted for the stratification factors only if a minimum number of events is observed, namely 1 event per strata per intervention group.

8.8. NEED OF OXYGEN (#23)

8.8.1. ESTIMANDS

Objective	Estimand
To determine if BCG vaccination compared with placebo reduces the need for oxygen therapy in the absence of any vaccine, measured over 12 months following randomisation in healthcare exposed to SARS-CoV-2.	Estimand 24.1<br><br>Population: ITT population<br>Outcome: need of oxygen therapy by 12 months<br>Interventions: BCG vs Placebo<br>Handling of Intercurrent events:<br>- any vaccine, including COVID-19 specific vaccine (Hypothetical strategy)<br>Summary Measure: Adjusted difference in proportion of participants
To determine if BCG vaccination compared with placebo reduces the need for oxygen therapy	Estimand 24.2

Objective	Estimand
irrespective of receiving any other vaccine (including COVID-19 specific vaccine), over the 12 months following randomisation, in healthcare workers exposed to SARS-CoV-2.	Population: as for estimand 24.1<br>Outcome: as for estimand 24.1<br>Interventions: as for estimand 24.1<br>Handling of Intercurrent events:<br>- any vaccine, including COVID-19 specific vaccine (Treatment Policy strategy)<br>Summary Measure: as for estimand 24.1

8.8.2. ANALYSIS

As per section 8.6.2.

8.9. ADMISSION TO CRITICAL CARE FOLLOWING A FEBRILE OR RESPIRATORY ILLNESS (#25)

8.9.1. ESTIMANDS

Objective	Estimand
To determine if BCG vaccination compared with placebo reduces admission to critical care following a febrile or respiratory illness in the absence of any vaccine, measured over 12 months following randomisation in healthcare exposed to SARS-CoV-2.	Estimand 25.1<br><br>Population: ITT population<br>Outcome: admission to critical care following a febrile or respiratory illness by 12 months<br>Interventions: BCG vs Placebo<br>Handling of Intercurrent events:<br>- any vaccine, including COVID-19 specific vaccine (Hypothetical strategy)<br>Summary Measure: Adjusted difference in proportion of participants
To determine if BCG vaccination compared with placebo reduces admission to critical care following a febrile or respiratory illness irrespective of receiving any vaccine, including COVID-19 specific vaccine (including COVID-19 specific vaccine), over the 12 months following randomisation, in healthcare workers exposed to SARS-CoV-2.	Estimand 25.2<br><br>Population: as for estimand 25.1<br>Outcome: as for estimand 25.1<br>Interventions: as for estimand 25.1<br>Handling of Intercurrent events:<br>- any vaccine, including COVID-19 specific vaccine (Treatment Policy strategy)<br>Summary Measure: as for estimand 25.1

8.9.2. ANALYSIS

As per section 8.6.2.
Additionally, in the subgroups of participants who were admitted to critical care following a febrile or respiratory illness, the mean and standard deviation (or median and IQR if distribution is skewed) of the duration of critical care will be calculated and presented by intervention group.

8.10. NEED OF MV FOR A FEBRILE OR RESPIRATORY ILLNESS (#26)

8.10.1. ESTIMANDS

Objective	Estimand
To determine if BCG vaccination compared with placebo reduces the need of MV for a febrile or respiratory illness in the absence of any vaccine, measured over 12 months following randomisation in healthcare exposed to SARS-CoV-2.	Estimand 26.1<br><br>Population: ITT population<br>Outcome: Need of MV for a febrile or respiratory illness by 12 months<br>Interventions: BCG vs Placebo<br>Handling of Intercurrent events:

Objective	Estimand
	- any vaccine, including COVID-19 specific vaccine (Hypothetical strategy)<br>Summary Measure: Adjusted difference in proportion of participants
To determine if BCG vaccination compared with placebo reduces Need of MV for a febrile or respiratory illness irrespective of receiving any vaccine, including COVID-19 specific vaccine , over the 12 months following randomisation, in healthcare workers exposed to SARS-CoV-2.	Estimand 26.2<br><br>Population: as for estimand 26.1<br>Outcome: as for estimand 26.1<br>Interventions: as for estimand 26.1<br>Handling of Intercurrent events:<br>- any vaccine, including COVID-19 specific vaccine (Treatment Policy strategy)<br>Summary Measure: as for estimand 26.1

8.10.2. ANALYSIS

As per section 8.6.2.

Additionally, in the subgroup of participants who needed MV for a febrile or respiratory illness, the mean and standard deviation (or median and IQR if distribution is skewed) of the duration of MV will be calculated and presented by intervention group.

8.11. DEATH AS A CONSEQUENCE OF AN EPISODE OF FEVER OR RESPIRATORY ILLNESS (#27)

8.11.1. ESTIMANDS

Objective	Estimand
To determine if BCG vaccination compared with placebo reduces the incidence of death as a consequence of an episode of fever or respiratory illness irrespective of receiving any vaccine, including COVID-19 specific vaccine , measured over 12 months following randomisation in healthcare exposed to SARS-CoV-2.	Estimand 27.1<br><br>Population: ITT population<br>Outcome: death as a consequence of an episode of fever or respiratory illness by 12 months<br>Interventions: BCG vs Placebo<br>Handling of Intercurrent events:<br>- any vaccine, including COVID-19 specific vaccine (Hypothetical strategy)<br>Summary Measure: Adjusted difference in proportion of participants
To determine if BCG vaccination compared with placebo reduces the incidence of death as a consequence of an episode of fever or respiratory illness in the absence of any vaccine, including COVID-19 specific vaccine , measured over 12 months following randomisation in healthcare exposed to SARS-CoV-2.	Estimand 27.2<br><br>Population: as for estimand 27.1<br>Outcome: as for estimand 27.1<br>Interventions: as for estimand 27.1<br>Handling of Intercurrent events:<br>- any vaccine, including COVID-19 specific vaccine (Treatment Policy strategy)<br>Summary Measure: as for estimand 27.1

8.11.2. ANALYSIS

As per section 8.6.2.

8.12. HOSPITALISATION FOR AN EPISODE OF FEVER OR RESPIRATORY ILLNESS (#28)

8.12.1. ESTIMANDS

Objective	Estimand
To determine if BCG vaccination compared with placebo reduces the incidence of hospitalisation for an episode of fever or respiratory illness irrespective of receiving any vaccine, including COVID-19 specific vaccine , measured over 12 months following randomisation in healthcare exposed to SARS-CoV-2.	Estimand 28.1<br><br>Population: ITT population<br>Outcome: hospitalisation for an episode of fever or respiratory illness by 12 months<br>Interventions: BCG vs Placebo<br>Handling of Intercurrent events:<br>- any vaccine, including COVID-19 specific vaccine (Hypothetical strategy)<br>Summary Measure: Adjusted difference in proportion of participants
To determine if BCG vaccination compared with placebo reduces the incidence of hospitalisation for an episode of fever or respiratory illness in the absence of any vaccine, including COVID-19 specific vaccine , measured over 12 months following randomisation in healthcare exposed to SARS-CoV-2.	Estimand 28.2<br><br>Population: as for estimand 28.1<br>Outcome: as for estimand 28.1<br>Interventions: as for estimand 28.1<br>Handling of Intercurrent events:<br>- any vaccine, including COVID-19 specific vaccine (Treatment Policy strategy)<br>Summary Measure: as for estimand 28.1

8.12.2. ANALYSIS

Same as per 7.8.2.

In the subset of participants who were admitted to hospital due to COVID-19, the mean and standard deviation (or median and IQR if not normally distributed) of the hospital stay will be calculated and presented by intervention group.

8.13. NUMBER OF DAYS OF UNPLANNED ABSENTEEISM FOR AN ACUTE ILLNESS OR HOSPITALISATION (#29a and #29b)

8.13.1. ESTIMANDS

Objective	Estimand
To determine if BCG vaccination compared with placebo reduces absenteeism, measured over 6 months following randomisation in healthcare workers who did not have a previous SARS-CoV-2 positive test result when assessed at time of randomisation.	Estimand 29a.1<br><br>Population: mITT population<br>Outcome: Number of days of unplanned absenteeism for an acute illness or hospitalisation by 6 months<br>Interventions: BCG vs Placebo<br>Handling of Intercurrent events:<br>- any vaccine, including COVID-19 specific vaccine (Treatment Policy strategy)<br>Summary Measure: Mean difference
To determine if BCG vaccination compared with placebo reduces absenteeism, measured over 6 months following randomisation in healthcare exposed to SARS-CoV-2.	Estimand 29a.2<br><br>Population: ITT population<br>Outcome: as for estimand 29a.1<br>Interventions: as for estimand 29a.1<br>Handling of Intercurrent events:<br>- as for estimand 29a.1<br>Summary Measure: as for estimand 29a.1

Objective	Estimand
To determine if BCG vaccination compared with placebo reduces absenteeism, measured over 12 months following randomisation in healthcare.	Estimand 29b.1<br><br>Population: ITT population<br>Outcome: Number of days of unplanned absenteeism for an acute illness or hospitalisation by 12 months<br>Interventions: BCG vs Placebo<br>Handling of Intercurrent events:<br>- any vaccine, including COVID-19 specific vaccine (Treatment Policy strategy)<br>Summary Measure: Mean difference

8.13.2. ANALYSIS

Mean and SD will be calculated and presented by intervention group. The difference in the means between the two treatment groups will be estimated using a GLM approach which will employ a Gaussian family and an identity link with robust standard errors, adjusted for randomisation strata used during randomisation as a covariate, and presented as mean differences and 95% CIs.
Should the outcome show a severely skewed distribution, then median, interquartile range (IQR) and range will be calculated and presented by intervention group. The differences between the medians with the 95% CI will be estimated using quantile regression models with robust standard errors, adjusted only for randomisation strata used during randomisation as a covariate

8.14. ADVERSE EVENTS and SERIOUS ADVERSE EVENTs

The number and proportion of participants with 1 or more adverse events over the 3 months following randomisation will be described overall as well as by type, severity (grade 0-4) and relationship to intervention. All results summaries will be presented by intervention group in the Safety population.

9. META-ANALYSIS

The data from the participants in the two stages of the trial will be combined in a meta-analysis for secondary analyses of all the non-COVID-19 outcomes.

The original trial plan included a meta-analysis of all the outcomes (both COVID-19- and non-COVID-19-related outcomes) combining data from Stage 1 and Stage 2 participants. However, in Stage 1 healthcare workers were recruited only in Victoria and Western Australia, both of which states had almost negligible COVID-19 exposure risk during the trial period (30th Mar 2020 to 13th May 2020). In light of this, the overwhelming majority of Stage 1 blood samples are likely to be seronegative. Moreover, with a low prevalence of COVID-19, there is a high probability that positive SARS-CoV-2 serology results are false positive. For these reasons, in December 2021 the BRACE team decided it was not justifiable to devote extra resources (time and costs) to the data cleaning of potential COVID-19 episodes and to testing SARS-CoV-2 serology for all participants in Stage 1. As a consequence, the meta-analysis will only be run on non-COVID-19 related outcomes and its main objective will be to determine if BCG vaccination compared with control reduces the rate and severity of febrile or other non-COVID-19 illness.

The analyses of these secondary non-COVID-19 outcomes will be the same as described in section 8 conducted on all Stage 1 and Stage 2 participants.

10. PLANNED ANALYSES

ANALYSIS OF THE 6 MONTHS COVID-19 OUTCOMES, Stage 2 participants
The first manuscript will include results from the analysis of the COVID-19-related outcomes collected in the first 6 months of each participant's participation in the trial and one non-COVID-19 related outcome at 6 months (29a). This analysis will include only participants recruited in Stage 2 of the trial.

ANALYSIS OF THE 12 MONTHS COVID-19 OUTCOMES, Stage 2 participants
A future manuscript will present results of the analyses of the COVID-19 related outcomes collected at 12 months. This analysis will include only participants recruited in Stage 2 of the trial.

ANALYSIS OF THE 12 MONTHS NON COVID-19 RELATED OUTCOMES (META ANALYSIS), Stages 1 and 2.
A future manuscript will present results of the analyses of all the non-COVID-19 related outcomes collected at 12 months. As addressed in section 9, this analysis will combine participants recruited in Stages 1 and 2 of the trial.

ANALYSIS OF THE EXPLORATORY OUTCOMES
A future manuscript will present results of the analyses of all the exploratory outcomes at 12 months combining participants recruited in Stages 1 and 2 of the trial.

11. REFERENCES

1. Pocock SJ. Group sequential methods in the design and analysis of clinical trials. Biometrika. 1977;64:191–199. [Google Scholar]

2. Royston P. Flexible Parametric Alternatives to the Cox Model: Update. The Stata Journal 2004; 4: 98-101. DOI:10.1177/1536867x0100400112.

3. Pittet LF, Messina NL, Gardiner K, Orsini F, Abruzzo V, Bannister S, Bonten M, Campbell JL, Croda J, Dalcolmo M, Elia S, Germano S, Goodall C, Gwee A, Jamieson T, Jardim B, Kollmann TR, Guimarães Lacerda MV, Lee KJ, Legge D, Lucas M, Lynn DJ, McDonald E, Manning L, Munns CF, Perrett KP, Prat Aymerich C, Richmond P, Shann F, Sudbury E, Villanueva P, Wood NJ, Lieschke K, Subbarao K, Davidson A, Curtis N; BRACE trial Consortium Group. BCG vaccination to reduce the impact of COVID-19 in healthcare workers: Protocol for a randomised controlled trial (BRACE trial). BMJ Open. 2021 Oct 28;11(10):e052101. doi:10.1136/bmjopen-2021-052101. PMID: 34711598; PMCID: PMC8557250.

12. SIGNATURES PAGE

Signature of Chief Principal Investigator:	*Nigel Curtis*<br>Nigel Curtis (May 23, 2022 08:33 GMT+2)	Date	May 23, 2022
Print Name	Prof. Nigel Curtis		

Signature of Trial Statistician:	*Francesca Orsini*<br>Francesca Orsini (May 18, 2022 11:47 GMT+10)	Date	May 18, 2022
Print Name	Francesca Orsini		

Signature of Trial Statistician:	[signature]<br>KJLee (May 18, 2022 11:49 GMT+10)	Date	May 18, 2022
Print Name	Katherine Lee		

BRACE_SAP_V1_18052022

Final Audit Report 2022-05-23

Created:	2022-05-18
By:	Thilanka Morawakage (thilanka.morawakage@mcri.edu.au)
Status:	Signed
Transaction ID:	CBJCHBCAABAAmcrVPECkTOvB5HcdM7Ec2mh5vu_dViN4

"BRACE_SAP_V1_18052022" History

Document created by Thilanka Morawakage (thilanka.morawakage@mcri.edu.au)
2022-05-18 - 1:34:12 AM GMT- IP address: 203.16.41.5

Document emailed to Nigel Curtis (nigel.curtis@rch.org.au) for signature
2022-05-18 - 1:38:07 AM GMT

Document emailed to Francesca Orsini (francesca.orsini@mcri.edu.au) for signature
2022-05-18 - 1:38:07 AM GMT

Document emailed to KJLee (katherine.lee@mcri.edu.au) for signature
2022-05-18 - 1:38:07 AM GMT

Email viewed by Francesca Orsini (francesca.orsini@mcri.edu.au)
2022-05-18 - 1:46:11 AM GMT- IP address: 203.16.41.5

Document e-signed by Francesca Orsini (francesca.orsini@mcri.edu.au)
Signature Date: 2022-05-18 - 1:47:02 AM GMT - Time Source: server- IP address: 203.16.41.5

Email viewed by KJLee (katherine.lee@mcri.edu.au)
2022-05-18 - 1:48:12 AM GMT- IP address: 185.243.108.184

Document e-signed by KJLee (katherine.lee@mcri.edu.au)
Signature Date: 2022-05-18 - 1:49:21 AM GMT - Time Source: server- IP address: 203.16.41.66

Email viewed by Nigel Curtis (nigel.curtis@rch.org.au)
2022-05-18 - 7:08:18 AM GMT- IP address: 172.225.189.101

Email viewed by Nigel Curtis (nigel.curtis@rch.org.au)
2022-05-19 - 9:05:13 AM GMT- IP address: 104.28.60.90

Email viewed by Nigel Curtis (nigel.curtis@rch.org.au)
2022-05-20 - 3:45:01 PM GMT- IP address: 172.225.94.86

Document e-signed by Nigel Curtis (nigel.curtis@rch.org.au)

Signature Date: 2022-05-23 - 6:33:09 AM GMT - Time Source: server- IP address: 185.37.119.232

Agreement completed.

2022-05-23 - 6:33:09 AM GMT

Adobe Acrobat Sign

RCH HREC 62586	Statistical Analysis Plan
Protocol Version 12.0 17May2022	

RCH HREC/protocol no: 62586
NCT04327206

BCG vaccination to Reduce the impact of COVID-19 in healthcare workers (BRACE) Trial

Statistical Analysis Plan

Document Version History

Version Date	Version	Author	Signature	Change Description	Reason/Comment
18-May-2022	1	Francesca Orsini	[signature]	Initial release.	Not applicable.
10-Aug-2022	2	Cecilia Moore	[signature]	Second release	a) Error in protocol version number and date corrected<br>b) Errors in section 4.3 and 7.4 corrected. The description of outcome derivation and estimands of secondary outcome 7a.1/7b.1 are now aligned with intended analysis of outcomes detailed in 7.4.1<br>c) revisions to analysis of non-COVID-19 related outcomes – i) treatment policy approach for intercurrent event of COVID-19 vaccination revised to be primary analysis, ii) model for outcome 19 (episodes of fever or respiratory illness) revised to ZNIB model and censoring rules corrected to align with analysis description of outcome 17 (section 8.1), iii) revisions made to analyses of outcome 29a and 29b (unplanned absenteeism) due to the anticipated nature of the data being zero-inflated and skewed count data

TABLE OF CONTENTS

LIST OF ABBREVIATIONS 4

1. STUDY OBJECTIVES 5
   - 1.1. PRIMARY OBJECTIVE 5
   - 1.2. SECONDARY OBJECTIVES 5
   - 1.3. PLANNED EXPLORATORY OBJECTIVES 6
2. BACKGROUND/INTRODUCTION 6
   - 2.1. STUDY DESIGN 6
   - 2.2. INTERVENTION GROUPS 6
   - 2.3. STUDY POPULATION 7
   - 2.4. SAMPLE SIZE 8
   - 2.5. STUDY PROCEDURE 10
3. POPULATIONS OF ANALYSIS 11
4. OUTCOME VARIABLES 12
   - 4.1. DATA COLLECTION 12
   - 4.2. PRIMARY OUTCOMES 13
   - 4.3. SECONDARY OUTCOMES 15
   - 4.4. OTHER VARIABLES 23
5. STATISTICAL METHODOLOGY 25
   - 5.1. GENERAL PRINCIPLES 25
   - 5.2. DEFINITION OF BASELINE 25
   - 5.3. DEFINITION OF THE 6- AND 12-MONTH CUT-OFFS 25
   - 5.4. DESCRIPTIVE STATISTICS 25
   - 5.5. THE ESTIMAND FRAMEWORK 26
   - 5.6. ANALYSIS SOFTWARE 26
6. PRIMARY OUTCOMES 27
   - 6.1. ESTIMANDS 27
   - 6.2. PRIMARY ANALYSIS 27
   - 6.3. SUPPLEMENTARY ANALYSES 29
   - 6.4. SUBGROUP ANALYSES 31
   - 6.5. SENSITIVITY ANALYSES 33
7. SECONDARY COVID-19 RELATED OUTCOMES 34
   - 7.1. SYMPTOMATIC COVID-19 AND SEVERE COVID-19 BY 12MONTHS (#3 AND #4) 34
   - 7.2. TIME TO FIRST SYMPTOM OF COVID-19 (#5A AND #5B) 37
   - 7.3. NUMBER OF EPISODES OF COVID-19 (#6A AND #6B) 40
   - 7.4. ASYMPTOMATIC SARS-COV-2 INFECTION (#7) 42
   - 7.5. NUMBER OF DAYS UNABLE TO WORK DUE TO COVID-19 (#8A AND #8B) 44
   - 7.6. NUMBER OF DAYS CONFINED TO BED DUE TO COVID-19 (#9A AND #9B) 45
   - 7.7. NUMBER OF DAYS WITH SYMPTOMS DUE TO COVID-19 (#10A AND #10B) 46
   - 7.8. PNEUMONIA DUE TO COVID-19 (#11A AND #11B) 48
   - 7.9. NEED OF OXYGEN DUE TO COVID-19 (#12A AND #12B) 49
   - 7.10. ADMISSION TO CRITICAL CARE DUE TO COVID-19 (#13A AND #13B) 51
   - 7.11. NEED OF MECHANICAL VENTILATION DUE TO COVID-19 (#14A AND #14B) 52
   - 7.12. HOSPITALISATION DUE TO COVID-19 (#15A AND #15B) 53
   - 7.13. DEATH DUE TO COVID-19 (#16A AND #16B) 55
8. NON-COVID19 RELATED SECONDARY OUTCOMES 56
   - 8.1. FEVER OR RESPIRATORY ILLNESS (#17) 56
   - 8.2. SEVERE FEVER OR RESPIRATORY ILLNESS (#18) 57
   - 8.3. NUMBER OF EPISODES OF FEVER OR RESPIRATORY ILLNESS (#19) 58
   - 8.4. NUMBER OF DAYS UNABLE TO WORK DUE TO FEVER OR RESPIRATORY ILLNESS (#20) 59
   - 8.5. NUMBER OF DAYS CONFINED TO BED DUE TO FEVER OR RESPIRATORY ILLNESS (#21) 59

8.6. NUMBER OF DAYS WITH SYMPTOMS DUE TO FEVER OR RESPIRATORY ILLNESS (#22) 60
8.7. PNEUMONIA (#23) 60
8.8. NEED OF OXYGEN (#23) 61
8.9. ADMISSION TO CRITICAL CARE FOLLOWING A FEBRILE OR RESPIRATORY ILLNESS (#25) 62
8.10. NEED OF MV FOR A FEBRILE OR RESPIRATORY ILLNESS (#26) 62
8.11. DEATH AS A CONSEQUENCE OF AN EPISODE OF FEVER OR RESPIRATORY ILLNESS (#27) 63
8.12. HOSPITALISATION FOR AN EPISODE OF FEVER OR RESPIRATORY ILLNESS (#28) 63
8.13. NUMBER OF DAYS OF UNPLANNED ABSENTEEISM FOR AN ACUTE ILLNESS OR HOSPITALISATION (#29A AND #29B) 64
8.14. ADVERSE EVENTS AND SERIOUS ADVERSE EVENTS 65

9. META-ANALYSIS **65**

10. PLANNED ANALYSES **65**

11. REFERENCES **66**

12. SIGNATURES PAGE 67

LIST OF ABBREVIATIONS

AE	Adverse Event
App	Smartphone Application
AR	Adverse Reaction
BCG	Bacille Calmette-Guérin
BMI	Body Mass Index
BRACE	BCG vaccination to Reduce the impAct of COVID-19 in hEalthcare workers
CI	Confidence Interval
COVID-19	Coronavirus Disease of 2019
DSMB	Data Safety Monitoring Board
GST	Group Sequential Test
ICU	Intensive Care Unit
ITT	Intent-To-Treat
LOCF	Last Observation Carry Forward
PCR	Polymerase Chain Reaction
RAT	Rapid Antigen Test
SAE	Serious Adverse Event
SARS-CoV-2	Severe Acute Respiratory Syndrome Coronavirus 2
SD	Standard Deviation
SE	Standard Error
TSC	Trial Steering Committee
MedDRA	Medical Dictionary for Regulatory Activities
WHO DD	World Health Organization Drug Dictionary

1. STUDY OBJECTIVES

1.1. PRIMARY OBJECTIVE

1. To determine if BCG vaccination (Intervention) compared with placebo (Comparator) reduces the incidence of 'symptomatic COVID-19' (Outcome) measured over the 6 months following randomisation (Time) in healthcare workers (Participants).
2. To determine if BCG vaccination (Intervention) compared with placebo (Comparator) reduces the incidence of 'severe COVID-19' (COVID-19-related death, hospitalisation, or non-hospitalised severe disease, defined as 'non-ambulant'[1] for ≥ 3 consecutive days OR Unable to work[2] for ≥ 3 consecutive days) (Outcome) measured over the 6 months following randomisation (Time) in healthcare workers (Participants).

[1] "pretty much confined to bed (meaning finding it very difficult to do any normal daily activities)"

[2] "I do not feel physically well enough to go to work"

Two primary outcomes have been chosen for this study: occurrence of COVID-19 and occurrence of severe COVID-19. Considering the number of unknown factors and the little knowledge of this new virus, we deemed it of clinical importance to have sufficient power to detect the potential effect of BCG vaccine compared to control for both outcomes. Our hypothesis is that, compared to control, the BCG vaccine will reduce both the number of cases of COVID-19 (increase the number of asymptomatic SARS-CoV-2 infections) and the number of cases of severe COVID-19. In other words, we hypothesise that BCG vaccine will shift the "severity of COVID-19" curve down, i.e., generally reduce the severity of the symptoms. The method used to control type I error is explained in the sample size section (11.1).

1.2. SECONDARY OBJECTIVES

3. To determine if BCG vaccination (Intervention) compared with placebo (Comparator) reduces the incidence of symptomatic COVID-19 (Outcome) measured over the 12 months following randomisation (Time) in healthcare workers (Participants).
4. To determine if BCG vaccination (Intervention) compared with placebo (Comparator) reduces the incidence of severe COVID-19 (non-hospitalised severe disease, hospitalisation or death) (Outcome) measured over the 12 months following randomisation (Time) in healthcare workers (Participants).
5. To determine if BCG vaccination (Intervention) compared with placebo (Comparator) prolongs the time to first COVID-19 episode (Outcome) measured over 6 and 12 months following randomisation (Time) in healthcare (Participants).
6. To determine if BCG vaccination (Intervention) compared with placebo (Comparator) reduces the severity of COVID-19 (Outcome) measured over 6 and 12 months following randomisation (Time) in healthcare workers (Participants).
7. To determine if BCG vaccination (Intervention) compared with placebo (Comparator) reduces the rate and severity of illness (fever or at least one sign or symptom of respiratory disease) measured over 12 months following randomisation (Time) in healthcare workers (Participants).
8. To determine if BCG vaccination (Intervention) compared with placebo (Comparator) reduces absenteeism (days off work) measured over 6 and 12 months following randomisation (Time) in healthcare workers (Participants).
9. To evaluate the safety of BCG vaccination in healthcare workers.

1.3. PLANNED EXPLORATORY OBJECTIVES

10. To determine in a subgroup of adults with recurrent cold sores whether BCG vaccination compared with placebo reduces herpes simplex recurrences (such as cold sores).
11. To determine the BCG vaccination induces changes in the immune system that are associated with protection against non-tuberculous infectious diseases including COVID-19.
12. To determine and compare changes in the immune system induced by vaccination.
13. To identify factors (e.g. age, sex, chronic conditions such as diabetes and cardiovascular disease, smoking, asthma, prior BCG vaccination, genetics, other vaccinations including COVID-19-specific vaccines, latent TB, immunological/molecular factors) that influence immune responses, infection and COVID-19 risk.
14. (Brazil specific) To identify biomarkers for diagnosing TB infection.

2. BACKGROUND/INTRODUCTION

2.1. STUDY DESIGN

BRACE is a phase III, two arms, multicentre, randomised placebo-controlled trial in healthcare workers to determine if BCG vaccine reduces the incidence and the severity of COVID-19 during the SARS-CoV-2 pandemic. At trial design, we planned to randomise 7,244 healthcare workers 1:1 to receive BCG or placebo.

Initially the trial was designed to compare primary and secondary outcomes between BCG and no BCG which was given concurrently with the influenza vaccination (Stage 1). The trial was then expanded to international sites and the design was revised to compare primary and secondary outcomes between BCG and a placebo (Stage 2). The comparison of BCG vs placebo in Stage 2 is the primary analysis of interest, however it is planned to combine the data from the two stages of the trial in a meta-analysis for secondary analyses of the non-COVID-19 outcomes. The analysis plan for this pre-planned meta-analysis is specified in section 9.

The two stages of the study are detailed below:

Dates	Stage	Planned Sample Size	Intervention	Control	Blinding
30th Mar 2020 to 13th May 2020	Stage 1	2,834	BCG+ Influenza	Influenza	Unblinded
14th May 2020 to 1st April 2021	Stage 2	7,244	BCG	Placebo	Blinded

As part of the monitoring, there was a formal interim analysis of the efficacy data. The details of this interim analysis are in a separate SAP [https://doi.org/10.25374/MCRI.14721309.v1]. This analysis compared the number of cases of severe COVID-19 (primary outcome 2) between the BGG group and the control group for those recruited after the introduction of the placebo (Stage 2 of the study).

2.2. INTERVENTION GROUPS

Participants were randomly allocated in a 1:1 ratio to the BCG vaccine group or to the control group in both Stage 1 and 2 of the study. Randomisation was stratified by:

- stage of the study (prior to or post the addition of the placebo vaccination);
- study site;
- age (<40 years; 40 to 59 years; ≥60 years); and
- presence of comorbidity (any of diabetes, chronic respiratory disease, cardiac condition, hypertension).

The BCG vaccine group received an adult dose of 0.1 mL of BCG vaccine SSI injected intradermally over the distal insertion of the deltoid muscle onto the humerus (approximately one third down the upper arm).

The control group in Stage 2 of the trial received 0.1 mL of 0.9% NaCl (placebo) injected intradermal over the distal insertion of the deltoid muscle onto the humerus. The control group in Stage 1 of the trial received the influenza vaccine on the day of randomisation. In Stage 2 of the trial, the control group received a placebo in an effort to blind participants to their treatment group allocation (although the subsequent local reaction at the injection site with BCG vaccination prevents total blinding).

Members of the trial team, except immunisers, are also blinded to the group allocation in Stage 2, achieved by hiding or removing the treatment group variable and all other variables related to BCG from the dataset, and will remain blinded until the database is locked for analysis.

2.3. STUDY POPULATION

Participants are adult (≥18 years) healthcare workers from Europe (the Netherlands, Spain and the United Kingdom), South America (Brazil) and Australia.

INCLUSION CRITERIA

- ≥ 18 years of age
- Healthcare worker
    - defined as anyone who works in a healthcare setting or has face-to-face contact with patients.
- Provide a signed and dated informed consent form
- Pre-randomisation blood collected
- Australian sites only: If annual influenza vaccination is available, receiving the influenza vaccine is an eligibility requirement. The influenza vaccine will be required a minimum of 3 days in advance of randomisation in the BRACE trial.

EXCLUSION CRITERIA

- Has any contraindication to BCG vaccine:
    - Fever or generalised skin infection (where feasible, randomisation can be delayed until cleared)
    - Weakened resistance toward infections due to a disease in/of the immune system
    - Receiving medical treatment that affects the immune response or other immunosuppressive therapy in the last year.
        - These therapies include systemic corticosteroids (≥20 mg for ≥2 weeks), non-biological immunosuppressant (also known as 'DMARDS'), biological agents (such as monoclonal antibodies against tumour necrosis factor (TNF)-alpha).
    - Congenital cellular immunodeficiencies, including specific deficiencies of the interferon-gamma pathway
    - Malignancies involving bone marrow or lymphoid systems
    - Any serious underlying illness (such as malignancy)
        - NB: People with cardiovascular disease, hypertension, diabetes, and/or chronic respiratory disease are eligible if not immunocompromised, and if they meet other eligibility criteria
    - Known or suspected HIV infection, even if they are asymptomatic or have normal immune function.
        - This is because of the risk of disseminated BCG infection
    - Active skin disease such as eczema, dermatitis or psoriasis at or near the site of vaccination
        - A different adjacent site on the upper arm can be chosen if necessary
    - Pregnant
        - Although there is no evidence that BCG vaccination is harmful during pregnancy, it is a contra-indication to BCG vaccination. Therefore, we will exclude women who think they could be pregnant or are planning to become pregnant within the next month.

        - UK specific: Although there is no evidence that BCG vaccination is harmful during pregnancy, it is a contra-indication to BCG vaccination. Therefore, we will exclude women of childbearing potential (WOCBP) who think they could be pregnant.
        - Spain specific: If the patient is female, and of childbearing potential, she must have a negative pregnancy test (provided by Sponsor) at the time of inclusion and practice a reliable method of birth control for 30 days after receiving the BCG vaccination.
    - Another live vaccine administered in the month prior to randomisation
    - Require another live vaccine to be administered within the month following BCG randomisation
        - If the other live vaccine can be given on the same day, this exclusion criteria does not apply
    - Known anaphylactic reaction to any of the ingredients present in the BCG vaccine
    - Previous active TB disease
- Currently receiving long term (more than 1 month) treatment with isoniazid, rifampicin or quinolone as these antibiotics have activity against *Mycobacterium bovis*
- Previous adverse reaction to BCG vaccine (significant local reaction (abscess) or suppurative lymphadenitis)
- BCG vaccine given within the last year
- Previous positive SARS-CoV-2 test result (PCR on a respiratory sample or SARS-CoV-2 antigen test approved by the local jurisdiction's public health policy)
- Already part of this trial, recruited at a different site/hospital.
- Participation in another COVID-19 prevention trial
- Previously received a COVID-19-specific vaccine

2.4. SAMPLE SIZE

ORIGINAL SAMPLE SIZE

The original sample size was calculated based on the two primary outcomes of: (1) the proportion of participants with COVID-19; and (2) the proportion of participants with severe COVID-19, by 6 months following randomisation. Since the trial aims to assess two primary outcomes, an adjustment for multiplicity was applied to maintain a global Type I error rate of 5% by splitting of this alpha.

The original sample size was based on the following:

i) 7244 healthcare workers recruited in Stage 2 would provide 80% power to detect a risk ratio of 0.67 (equivalent to a 1.3% absolute difference) in the BCG group compared to the control group for severe COVID-19 at 6 months (primary outcome 2), assuming 4% of subjects will have severe COVID-19 by 6 months in the control group and allowing for 16% lost to follow-up by 6 months (2-sided alpha =0.04).

ii) 2016 healthcare workers would provide 95% power to detect an absolute difference of 10% in incidence of COVID-19 (primary outcome 1), assuming 55% of subjects will have COVID-19 in the control group (2-sided alpha = 0.005).

iii) In the pre-planned meta-analysis, 10,078 healthcare workers recruited in Stages 1 and 2 would provide 90% power to detect a risk ratio of 0.67 (equivalent to an absolute risk difference of 1.3%) in the BCG group compared to the control group for severe COVID-19 at 6 months (primary outcome 2), assuming 4% of subjects will have severe COVID-19 by 6 months in the control group and allowing for 20% lost to follow-up by 6 months (2-sided alpha = 0.04).

iv) We originally allocated alpha=0.005 to an efficacy interim analysis using the conservative approach of splitting the alpha allocated to primary outcome (2) between the interim and final analysis. Under the original sample size calculation in Stage 1 of the trial we planned to recruit 1,668 participants per group which gave us 72% power to identify a reduction from an incidence of 4% in severe COVID-19 at 6 months

in the control group to 2% in the intervention group. If the assumptions were correct, this would equate to 100 cases in total. We therefore planned a formal interim analysis of severe COVID-19 once there had been 100 cases of severe COVID-19. For full details, refer to section 11.4 of the trial protocol.

For full details refer to section 11.1 of the trial protocol. The analysis plan for the pre-planned meta-analysis is detailed in section 9.

CEASATION OF ENROLMENT AND SUBSEQUENT EFFICACY INTERIM ANALYSIS

Recruitment into the BRACE trial was stopped prematurely on 1st April 2021, after 3,988 participants had been recruited into Stage 2, 6,285 overall (including 2,840 recruited in Stage 1). The main reason for stopping before reaching the calculated sample of 10,078 (2,834 planned for Stage 1 + 7,244 planned for Stage 2) was the rollout of COVID-19-specific vaccines in healthcare workers around the world, which started in December 2020. The availability of COVID-19-specific vaccines decreased the interest for potential participants to be recruited into the study, and the receipt of COVID-19 specific vaccines by participants affects the ability of the trial to determine the effectiveness of BCG vaccination in protecting against COVID-19. With 3,988 participants randomised into Stage 2 of the trial, there would be 63.3% power to identify an absolute reduction of 1.30% (relative reduction of 1/3, the effect size used to power Stage 2 of the trial) in the incidence of severe COVID-19 at the end of the trial, from an incidence of 4% at 6 months in the control group to 2.67% in the intervention group (the assumptions used in the sample size calculation for Stage 2) based on a two-sided test with alpha = 0.045, if no interim analysis were planned.

At the time recruitment was stopped, we were monitoring the occurrence of severe COVID-19 cases in preparation for the interim analysis which was scheduled to occur once 100 severe cases was reached (refer to section 11.4 of the protocol). For reasons described in detail in the Interim analysis SAP (https://doi.org/10.25374/MCRI.14721309.v1), we decided to revise the stopping rule to be used in the interim analysis of severe COVID-19 to an alpha spending function, where the threshold to identify efficacy is based on the amount of data available at the time of the interim analysis.

The interim analysis included only exposure time before any dose of any COVID-19-specific vaccine. Given that it is unknown what effect a COVID-19-specific vaccine will have on the effectiveness of the BCG vaccination (or vice versa), participants were censored at the time of their first COVID-19-specific vaccine.

The database lock for the interim analysis happened on the 30th April 2021 (30 days after recruitment was ceased). Up to the this date, 82.3% of participants had received a COVID-19-specific vaccine, or had reported an episode of severe COVID-19, and/or had been followed for at least 6 months from randomisation. Using an alpha-spending function based on the Pocock stopping rule[1], an interim analysis conducted on 82.3% of the available information on 3988 participants, and an overall alpha of 0.045 for this outcome, results in a nominal alpha of 0.04 at the interim analysis, and 0.021 at the end of the study (calculated using a Group Sequential Test (GST) of Two Proportions in NQuery (PTT12-1)). Thus, the threshold of 0.04 was used as the stopping rule for the interim analysis. At the interim time point we had 52.9% power to detect a risk ratio of 0.67 in the incidence of severe COVID-19 at 6 months. Under this spending function we will have 0.021 alpha left for the final analysis given that the treatment comparison did not reach the threshold at the interim analysis.

The details of how the interim analysis was conducted and presented are provided in the Interim analysis SAP (https://doi.org/10.25374/MCRI.14721309.v1).

2.5. STUDY PROCEDURE

Figure 1 and Table 1 provide a summary of the study procedures for the BRACE trial.

Figure 1. Trial timeline

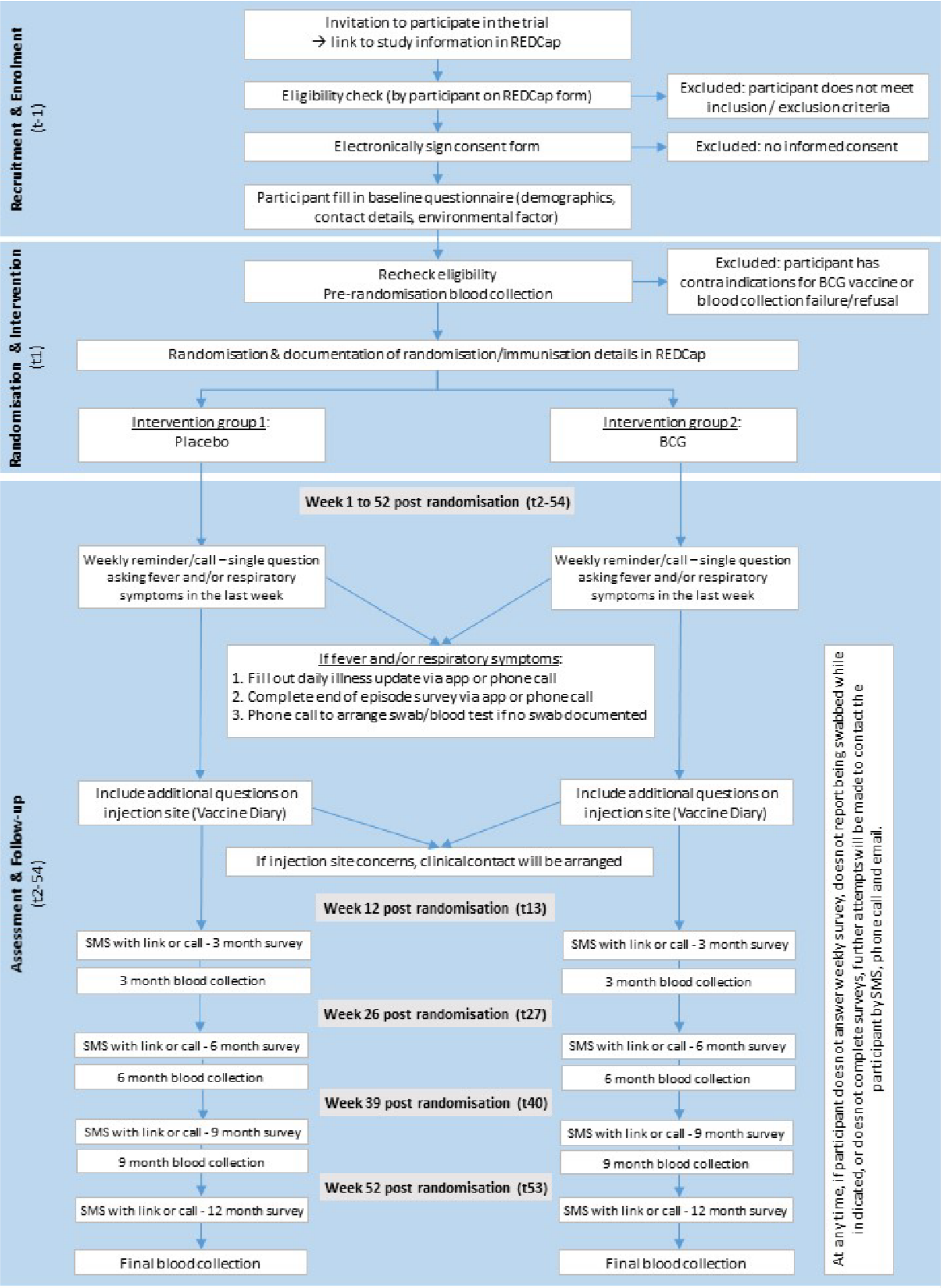

Table 1. Trial timeline

	TRIAL PERIOD									
	Pre-study	Inclusion & randomisation	Post-randomisation							
TIME POINT	t_{-1}	t_0	t_{1-12}	t_{13}	t_{14-25}	t_{26}	t_{27-38}	t_{39}	t_{40-51}	t_{52}
RECRUITMENT:										
Eligibility screen	X									
Informed consent	X									
Contact details	X									
Allocation to intervention		X								
INTERVENTIONS:										
BCG vaccine		X (BCG group)								
Saline injection		X (Placebo group)								
ASSESSMENTS:										
Baseline questionnaire	X	X								
Weekly survey			X	X	X	X	X	X	X	X
Instruction for swab testing (if indicated by weekly survey)			(X)	(X)	(X)	(X)	(X)	(X)	(X)	(X)
3-month survey				X						
6-month survey						X				
9-month survey								X		
12-month survey										X
*Clinical advice on injection site **			X	X						
*Blood collection***		X		X		X		X#		X#
*Baseline SARS-CoV-2 Test ****		X								

T=week (e.g. t1=first week). A 42day window period is accepted for the periodic survey and the blood collection timepoints
* In indicated Infectious Diseases clinician, or state-based organisation, as appropriate
** Optional consent for additional biological sample including blood sample when illness reported
*** Brazil only as outlined in Appendix 4
# Sub-set of participants

3. POPULATIONS OF ANALYSIS

Intention-To-Treat Population

The intention-to-treat (ITT) population will be used for the secondary analysis of efficacy outcomes, with all participants analysed according to the study group to which they were randomly allocated, regardless of the intervention they received. The only participants excluded from this population will be participants who were randomised in error, i.e., assigned by mistake to one of the two allocations even though they were not eligible for the study.

Modified Intention-To-Treat Population (mITT)

The primary population for all efficacy analyses will be the modified intention-to-treat population (mITT) which will only include participants who had a negative SARS-CoV-2 test result at time of randomisation. The mITT population is the same as the ITT population but will also EXCLUDE:

- participants with positive or missing or indeterminant serology at the time of randomisation
- (Applicable only to participants at the Brazilian sites) participant with positive/missing/indeterminant PCR on a respiratory sample or a positive/missing/indeterminant SARS-CoV-2 diagnostic antigen test approved by the local jurisdiction's public health policy at the time of randomisation

Safety Population

The primary population for all safety analyses will be the safety population, which will include all randomised participants who received the vaccine (either BCG or placebo), with all participants analysed according to the intervention they received, irrespective of which group they were randomised to.

4. OUTCOME VARIABLES

4.1. DATA COLLECTION

Participants are asked weekly to report whether they have had any of the following:

1. “Trigger symptoms”, namely:
    - fever (self-reported, defined as temperature > 38 degrees centigrade)
    - intermittent cough
    - persistence cough
    - shortness of breath or difficulty breathing
    - sore throat

2. “Other symptoms”, namely:
    - runny/blocked nose
    - headache
    - muscle and/or joint ache
    - fatigue
    - nausea, vomiting and/or diarrhea
    - loss of taste and/or smell

These weekly reports are collected via a smartphone app (or in the case of Brazil via a weekly phone call) and are collected daily when they are ill (or in the case of Brazil via a phone call every 3 days) (see below). The information collected via the app/phone calls is subsequently confirmed through quarterly questionnaires, at 3, 6, 9 and 12 months post randomisation.

As soon as a participant reports:

- a “trigger symptom”

or

- being non-ambulant for ≥ 3 consecutive days or unable to work for ≥ 3 consecutive days irrespective of symptoms (any episode of illness characterised by ≥ 3 consecutive days or unable to work for ≥ 3 consecutive days is defined a severe episode of illness)

the participant is prompted to have a SARS-CoV-2 test and continues reporting their symptoms on a daily basis until resolution.

Once recovered from the illness, i.e., on the first day with no symptoms, the participant is required to complete an illness resolution form, documenting whether the SARS-CoV-2 test(s) was positive or negative. In circumstances where the participant does not recover from the illness this is entered by the study site coordinator or MCRI data team.

SARS-CoV-2 tests (date, type and results), as well as COVID-19-specific vaccination (date and brand), are also collected via the smartphone app, phone calls, and the quarterly questionnaires.

Quarterly questionnaires at 3, 6, 9 and 12 months from randomisation collect data on hospitalisations and absenteeism. When a participant is hospitalised due to a respiratory or febrile illness, the site coordinator or safety medical doctor complete a hospitalisation form collecting data on diagnosis, treatment and outcome of the hospitalisation. Information on the need for oxygen, mechanical ventilation, admission to critical care/ICU are also collected. Medical records are used by the local teams to complete the hospitalisation forms. Quarterly questionnaires also summarise all the episodes of illness that the participant has entered in the smartphone app

in the prior quarter and ask the participant to confirm those listed, edit them if needed, and retrospectively add episodes of illness that participant may have forgotten to enter into the smartphone app.

Blood samples are collected at baseline, 3-months, 6-months, 9-months and 12 months from randomisation, to assess SARS-CoV-2 serology.

4.2. PRIMARY OUTCOMES

1	Symptomatic COVID-19 by 6 months following randomisation defined as:<br>- positive SARS-CoV-2 test (PCR, antigen or serology), PLUS<br>- [fever (using self-reported questionnaire), OR<br>- at least one 'trigger' symptom of respiratory disease including cough, sore throat, shortness of breath, respiratory distress/failure (using self-reported questionnaire)]
2	Severe COVID-19 by 6 months following randomisation defined as:<br>- positive SARS-CoV-2 test (PCR, antigen or serology), PLUS<br>- [Death as a consequence of COVID-19, OR<br>- Hospitalised as a consequence of COVID-19, OR<br>- Non-hospitalised severe disease as a consequence of COVID-19, defined as non-ambulant[1] for ≥ 3 consecutive days or unable to work[2] for ≥ 3 consecutive days]<br><br>[1] *"pretty much confined to bed (meaning finding it very difficult to do any normal daily activities"*<br><br>[2] *"I do not feel physically well enough to go to work"*

The date of occurrence for either a symptomatic COVID-19 episode (primary outcome 1) or a severe COVID-19 episode (primary outcome 2) will be defined as the first date of any symptom onset for the episode ('trigger' or 'other' symptom, as listed in section 4.1). This is to account for any potential difference between groups in time to SARS-CoV-2 testing from symptom onset.

Excluded episodes
An episode of illness with trigger symptom(s) for which a SARS-CoV-2 testing was not medically indicated (e.g. sore throat due to a known allergy), will not be considered as an episode of illness in the interest of the calculation of primary outcome 1. A severe episode of illness for which a SARS-CoV-2 testing was not medically indicated (e.g. unable to work for ≥ 3 consecutive days due to sprained ankle), will not be considered as an episode of illness in the interest of the calculation of primary outcome 2.

DEFINITION OF SARS-COV-2 TEST
SARS-CoV-2 tests comprise:
- A PCR test, and/or
- A rapid antigen test (RAT), and/or
- Seroconversion determined using the serology results prior to and after the onset of symptoms for an episode of illness (see Figure 3).

DEFINITION OF COVID-19 EPISODES
An episode of illness (either with trigger symptoms or severe episode) will be considered to have an associated COVID-19 test if the episode has a RAT or PCR test or serology data as follows:

PCR/RAT tests – The results of PCR and/or RAT tests will be used for the determination of the COVID-19 episode (either symptomatic or severe) as detailed in Figure 2 using the following testing windows:

PCR: ≤ 3 days prior to onset of symptoms to ≤ 21 days after the onset of symptoms, *or*
≤ 7 days from the last day of symptoms

RAT: ≤ 3 days prior to onset of symptoms to ≤ 10 days after the onset of symptoms

Figure 2 details how results of the RAT/PCT tests will be interpreted.

Figure 2. RAT/PCR testing interpretation

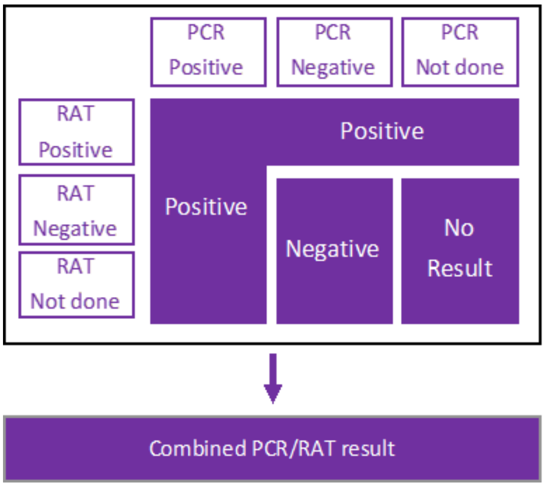

PCR/RAT taken within their testing window

Serology - The results of a serology will also be used for the determination of the COVID-19 episode if both pre-episode and post-episode blood samples results are available. The algorithm in Figure 3 details how serology results will be interpreted.

Figure 3. SARS-CoV-2 Serology Interpretation

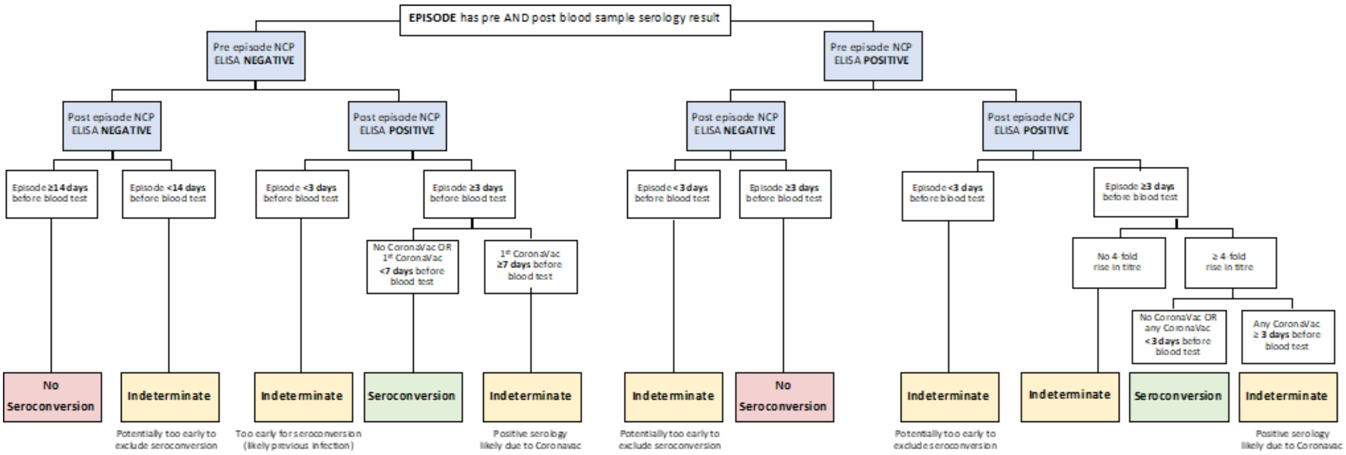

Source: SARS-CoV-2 serology interpretation, version 20-Apr-2022
NCP=nucleocapsid protein

The combination of the results from RAT/PCR tests and/or serology will be interpreted using the algorithm in Figure 4 to categorise episode with trigger symptoms/severe episodes to: COVID-19 episodes (either symptomatic or severe), non-COVID-19 episodes, or episodes with missing information.

Figure 4. COVID-19 episode interpretation

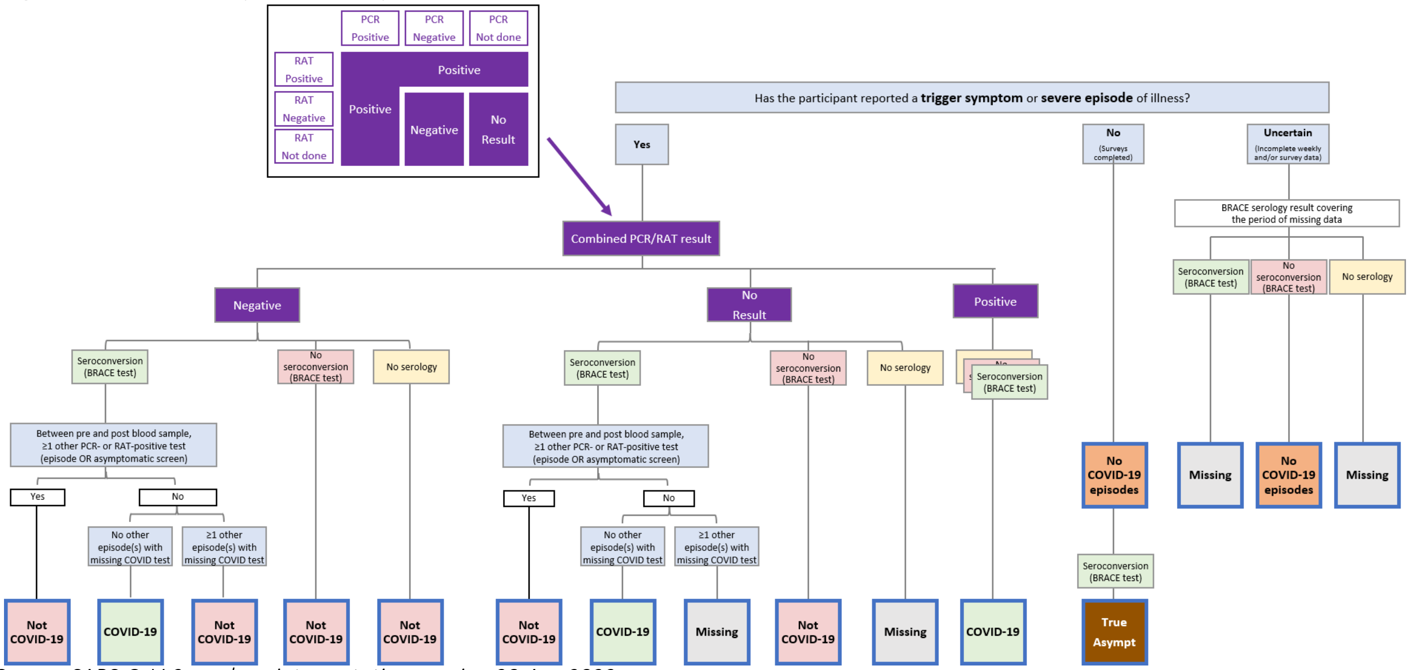

Source: SARS-CoV-2 serology interpretation, version 20-Apr-2022
Asympt COVID-19 = Asymptomatic SARS-CoV-2 infection, as per definition provided in section 4.3, outcome 7a and 7b.

Additionally, each episode of illness (either with trigger symptoms or severe episode) will be categorised as COVID-19 episodes (either symptomatic or severe), non-COVID-19 episodes, or episodes with missing information ignoring serology, i.e. only using data from RAT and PCR tests (Figure 2). This will be used as part of a sensitivity analysis (see session "Sensitivity Analysis 2").

4.3. SECONDARY OUTCOMES

#	COVID-19 Related Outcomes
3	Symptomatic COVID-19 by 12 months following randomisation as defined for primary outcome 1.<br><br>CALCULATION<br>As per primary outcome 1 but over 12 months.
4	Severe COVID-19 by 12 months following randomisation as defined for primary outcome 2.<br><br>CALCULATION<br>As per primary outcome 1 but over 12 months.
5a	Time to first symptom of COVID-19 over the 6 months following randomisation.<br><br>CALCULATION<br>Participants who had either a symptomatic or severe COVID-19 episode will have time to first symptom of COVID-19 calculated as:<br><br>*date of any symptom onset for the first symptomatic or severe COVID-19 episode – date of randomisation*<br><br>Participants who have not had a symptomatic or severe COVID-19 episode will have time calculated as:<br>*Earliest censoring date*– date of randomisation*

#	COVID-19 Related Outcomes
	For participants who experience more than one COVID-19 episode, the time to the first of these episodes will be used for the analysis.<br><br>**as defined in section 6.2.3*
5b	Time to first symptom of COVID-19 over the 12 months following randomisation.<br><br>CALCULATION<br>As per 5a but over 12 months.
6a	Number of episodes of COVID-19 by 6 months.<br><br>CALCULATION<br>The total number of symptomatic or severe COVID-19 episodes (refer to section 4.2 for their definition) by 6 months will be calculated for each participant.<br>Participants who have had neither a symptomatic nor a severe COVID-19 episode by 6 months will be considered to have had 0 (zero) episodes of COVID-19.<br>Two COVID-19 episodes of illness are considered distinct if they are >10 days apart.
6b	Number of episodes of COVID-19 by 12 months.<br><br>CALCULATION<br>As 6a but over 12 months.
7a	Asymptomatic SARS-CoV-2 infection by 6 months<br><br>Asymptomatic SARS-CoV-2 infection will be defined as<br>- Evidence of SARS-CoV-2 infection (by seroconversion) and<br>- Absence of any episodes of illness (defined by trigger or non-trigger symptoms) (using self-reported questionnaire) and<br>- No evidence of exposure prior to randomisation<br><br>CALCULATION<br>Participants will be considered to have met this outcome if by 6 months:<br>- they seroconverted to SARS-CoV-2, AND<br>- they have complete diary data or survey data up to the earlier of 6 months or the date of the blood draw at which they became seropositive which confirm they did not have any episodes of illness<br><br>Participants will be considered to NOT have met this outcome if they:<br>- have complete diary data or survey data up to 6 months AND<br>do not show evidence of seroconversion to SARS-CoV-2 at 6 months (i.e., serology result at 3 months is not positive and at 6 months is negative) OR<br>- have complete diary data or survey data up to 6 months AND<br>- show evidence of seroconversion to SARS-CoV-2 at 6 months (i.e., serology result at 3 or 6 months is positive) AND<br>- reported any episode of illness that could have accounted for the seroconversion (experienced either an episode which tested positive to PCR/RAT tests or an episode that was not tested for COVID-19)
7b	Asymptomatic SARS-CoV-2 infection over 12 months.<br><br>CALCULATION<br>As per 7a but over 12 months.
8a	Number of days unable to work due to COVID-19 within 6 months following randomisation (excludes quarantine/workplace restrictions)

#	COVID-19 Related Outcomes
	CALCULATION<br>This is a count of the days marked "unable to work" across all COVID-19 episodes (either symptomatic or severe, as defined in section 4.2) by 6 months post randomisation. Participants who have not had a symptomatic or severe COVID-19 episode during this period will be regarded as having had 0 (zero) days unable to work due to COVID-19.
8b	Number of days unable to work due to COVID-19 within 12 months following randomisation (excludes quarantine/workplace restrictions)<br><br>CALCULATION<br>As for outcome 8a but over 12 months.
9a	Number of days confined to bed due to COVID-19 within 6 months following randomisation.<br><br>CALCULATION<br>This is a count of the days marked "confined to bed" across all COVID-19 episodes (either symptomatic or severe, as defined in section 4.2) by 6 months post randomisation. Participants who have not had a symptomatic or severe COVID-19 episode during this period will be regarded as having had 0 (zero) days confined to bed due to COVID-19.
9b	Number of days confined to bed due to COVID-19 within 12 months following randomisation.<br><br>CALCULATION<br>As for outcome 9a but over 12 months.
10a	Number of days with symptoms due to COVID-19 within 6 months following randomisation.<br><br>CALCULATION<br>The number of days with symptoms for a given COVID-19 episode is the number of days from the start to the end of the COVID-19 episode (either symptomatic or severe, as defined in section 4.2) using this formula:<br>*Episode duration = Episode end date* – episode start date*<br>** defined as first day with no symptoms*<br><br>For participants who die as a consequence of COVID-19, the end date will be the date of death.<br>For participants who had multiple COVID-19 episodes within 6 months, the number of days with symptoms due to COVID-19 will be the sum of durations across all the COVID-19 episodes within the 6 months following randomisation.<br>Participants who have not had a symptomatic or severe COVID-19 episode within 6 months of randomisation will be regarded as having had 0 (zero) days with symptoms due to COVID-19.
10b	Number of days with symptoms due to COVID-19 within 12 months following randomisation.<br><br>CALCULATION<br>As for outcome 10a but over 12 months.
11a	Pneumonia due to COVID-19 by 6 months.<br><br>CALCULATION<br>The derivation of this outcome will use data from the self-reported questionnaire and/or hospitalisation forms.<br>Participants will be coded as having had the outcome if:<br>- They developed pneumonia during a symptomatic or a severe COVID-19 episode within 6 months of randomisation<br>Participants will be coded as not having had this outcome if:<br>- They did not develop pneumonia during a symptomatic or a severe COVID-19 episode or

#	COVID-19 Related Outcomes
	- They did not have a symptomatic or a severe COVID-19 episode within 6 months of randomisation.
11b	Pneumonia due to COVID-19 by 12 months.<br><br>CALCULATION<br>As for outcome 11a but over 12 months.
12a	Need for oxygen therapy due to COVID-19 by 6 months.<br><br>CALCULATION<br>The derivation of this outcome will use data from the self-reported questionnaire and/or hospitalisation forms.<br>Participants will be coded having had the outcome if:<br>- They needed oxygen therapy during a severe COVID-19 episode within 6 months of randomisation<br>Participants will be coded not having had the outcome if:<br>- They did not need oxygen therapy during a severe COVID-19 episode or<br>- They did not have a severe COVID-19 episode within 6 months of randomisation
12b	Need for oxygen therapy due to COVID-19 by 12 months.<br><br>CALCULATION<br>As for outcome 12a but over a 12 month period.
13a	Admission to critical care and duration of stay due to COVID-19 by 6 months.<br><br>CALCULATION<br>The derivation of both outcomes will use data from the self-reported questionnaire and/or hospitalisation forms.<br>– Admission to critical care (including ICU)<br>Participants will be coded as having had at least one admission to critical care/ICU due to COVID-19 if:<br>- They were admitted to critical care/ICU during a severe COVID-19 episode within 6 months of randomisation<br>Participants will be coded as not having had this outcome if:<br>- They were NOT hospitalised during a severe COVID-19 episode or<br>- They were NOT admitted to critical care/ICU during a hospitalisation for a severe COVID-19 episode or<br>- They did not have a severe COVID-19 episode within 6 months of randomisation<br><br>– Duration of stay<br>For participants who were admitted to critical care/ICU during a severe COVID-19 episode within 6 months of randomisation, we will also calculate the duration of stay as the difference between the date of admission and date of discharge. For participants who had multiple COVID-19 episodes with admission to critical care within 6 months of randomisation, this number will be the sum of durations across all critical care stays.
13b	Admission to critical care and duration of stay due to COVID-19 by 12 months.<br><br>CALCULATION<br>As for outcome 13a but over 12 months.
14a	Need of mechanical ventilation (MV) and duration of MV due to COVID-19 by 6 months.<br><br>CALCULATION<br>The derivation of both outcomes will use data from the self-reported questionnaire and/or hospitalisation forms.<br>– Need of MV

#	COVID-19 Related Outcomes
	Participants will be coded as having been in need of MV due to SARS-CoV-2 if:<br>- They needed MV during a severe COVID-19 episode within 6 months of randomisation<br>Participants will be coded as not having had this outcome if:<br>- They were NOT hospitalised during a severe COVID-19 episode or<br>- They did NOT need MV during a hospitalisation for a severe COVID-19 episode or<br>- They did not have a severe COVID-19 episode within 6 months of randomisation<br><br>– Duration of MV<br>For participants who needed mechanical ventilation within 6 months of randomisation, we will also calculate the duration of MV as the difference between the date/time MV was started and the date/time MV was stopped. For participants who had multiple COVID-19 episodes requiring MV within 6 months of randomisation V, this number will be the sum of durations across all COVID-19 episodes requiring MV.
14b	Need of mechanical ventilation and duration of MV due to COVID-19 by 12 months.<br><br>CALCULATION<br>As for outcome 14a but over 12 months.
15a	Hospitalisation due to COVID-19 (using self-reported questionnaire and/or medical/hospital records) and duration of hospitalisation by 6 months.<br><br>CALCULATION<br>– Hospitalisation due to COVID-19<br>Participants will be coded as having been hospitalised due to COVID-19 if:<br>- They had severe COVID-19 (primary outcome 2) and were hospitalised as a consequence of COVID-19 within 6 months of randomisation<br>Participants will be coded as not having had the outcome if:<br>- They had severe COVID-19 (primary outcome 2) and were NOT hospitalised as a consequence of COVID-19<br>- They did not have severe COVID-19 (did not meet the definition of either the primary outcome 2) within 6 months of randomisation<br><br>– Duration of hospitalisation<br>For participants who were hospitalised due to COVID-19 within 6 months of randomisation, the duration of their hospital stay due to COVID-19 will be calculated as the difference between date of admission and date of discharge. For participants who had multiple severe COVID-19 episodes resulting in hospitalisation within 6 months of randomisation, this number will be the sum of durations across all hospitalisations due to COVID-19.
15b	Hospitalisation due to COVID-19 (using self-reported questionnaire and/or medical/hospital records) and duration of hospitalisation by 12 months.<br><br>CALCULATION<br>As for outcome 15a but over 12 months.
16a	Death due to COVID-19 by 6 months.<br><br>CALCULATION<br>Participants will be coded as having had the outcome if:<br>- They had severe COVID-19 (primary outcome 2) and died as a consequence of COVID-19 within 6 months of randomisation<br>Participants will be coded as not having had the outcome if:<br>- They had severe COVID-19 (primary outcome 2) and did NOT die or<br>- They did not have severe COVID-19 or<br>- They died for other reasons (not as a consequence of COVID-19) within 6 months of randomisation

#	COVID-19 Related Outcomes
16b	Death due to COVID-19 by 12 months.<br><br>CALCULATION<br>As for outcome 16a but over 12 months.

#	Other Outcomes – NON-COVID-19 Related Outcomes
17	Fever or respiratory illness*(using self-reported questionnaire), over the 12 months following randomisation.<br><br>* Respiratory illness will be defined as:<br>at least one sign or symptom of respiratory disease including cough, sore throat, shortness of breath, respiratory distress/failure, or runny/blocked nose (in combination with another respiratory symptom or fever).<br><br>Fever due to reactions to any vaccine will not be considered for this outcome.<br><br>CALCULATION<br>Participants will be coded as having had the outcome if:<br>- They developed an episode of illness with fever (self-reported, defined as temperature > 38 degrees) or respiratory illness (including due to COVID-19) within 12 months of randomisation<br>Participants will be coded as not having had the outcome if:<br>- They did not have any episodes of fever or respiratory illness within 12 months of randomisation
18	Severe fever or respiratory illness*(using self-reported questionnaire), over the 12 months following randomisation, defined as:<br><br>- Death, OR<br>- Hospitalised, OR<br>- Non-hospitalised severe disease, defined as non-ambulant1 for ≥ 3 consecutive days or unable to work 2 for ≥ 3 consecutive days<br>as a consequence of fever or respiratory illness, as defined above (outcome 17)<br><br>[1] "pretty much confined to bed (meaning finding it very difficult to do any normal daily activities"<br><br>[2] "I do not feel physically well enough to go to work" (excludes stay at home exclusively for quarantine/workplace restrictions)<br><br>Fever due to reactions to any vaccine will not be considered for this outcome.<br><br>CALCULATION<br>Participants will be coded as having had the outcome if:<br>- They developed a severe episode of illness with fever (self-reported, defined as temperature > 38 degrees) or respiratory illness (including due to COVID-19) within 12 months of randomisation<br>Participants will be coded as not having had the outcome if:<br>- They did not have any severe episodes of fever or respiratory illness within 12 months of randomisation
19	Number of episodes of fever or respiratory illness (as defined in outcome 17), over the 12 months following randomisation.<br><br>CALCULATION

#	Other Outcomes – NON-COVID-19 Related Outcomes
	This will reflect the number of distinct episodes of fever or respiratory illness within 12 months of randomisation. Participants who have not experienced any episodes of fever or respiratory illness within 12 months of randomisation will be considered to have had 0 (zero) episodes.
20	Number of days unable to work (using self-reported questionnaire) due to fever or respiratory illness (as defined in outcome 17), over the 12 months following randomisation (excludes quarantine/workplace restrictions)<br><br>CALCULATION<br>This will be calculated as the sum of the days marked as unable to work due to fever or respiratory illness within 12 months of randomisation. Participants who have not experienced any episodes of fever or respiratory illness within 12 months of randomisation will be considered to have had 0 (zero) episodes.
21	Number of days confined to bed (using self-reported questionnaire) due to fever or respiratory illness, illness (as defined in outcome 17), over the 12 months following randomisation<br><br>CALCULATION<br>This will be calculated as the sum of the days marked as confined to bed to fever or respiratory illness within 12 months of randomisation. Participants who have not experienced any episodes of fever or respiratory illness will be considered to have had 0 (zero) episodes within 12 months of randomisation.
22	Number of days with symptoms due to fever or respiratory illness (as defined in outcome 17), over the 12 months following randomisation<br><br>CALCULATION<br>For each episode of fever or respiratory illness the number of days from the start to the end dates of the episode of fever or respiratory illness will be calculated using this formula:<br>*Episode duration = Episode end date* – episode start date*<br>** defined as first day with no symptoms*<br><br>For participants who are hospitalised due to fever or respiratory illness, the end date will be the date of discharge from the hospital.<br>For participants who die as a consequence of fever or respiratory illness, the end date will be the date of death.<br>For participants who had multiple episodes of fever or respiratory illness within 12 months of randomisation, this number will be the sum of durations across all the episodes.<br>Participants who have not had an episode fever or respiratory illness within 12 months of randomisation will be regarded as having had 0 (zero) days with symptoms due to fever or respiratory illness.
23	Pneumonia within a febrile or respiratory illness over the 12 months following randomisation<br><br>CALCULATION<br>Data from the self-reported questionnaire and/or hospitalisation forms will be used to derive this outcome.<br>Participants will be coded as having had the outcome if:<br>- They developed pneumonia during an episode of fever or respiratory illness within 12 months of randomisation<br>Participants will be coded as not having had the outcome if:<br>- They did not developed pneumonia during a episode of fever or respiratory illness or<br>- They did not have any episodes of fever or respiratory illness<br>- within 12 months of randomisation
24	Need for oxygen therapy for a febrile or respiratory illness over the 12 months following randomisation<br><br>CALCULATION<br>The derivation of this outcome will use data from the self-reported questionnaire and/or hospitalisation forms.<br>Participants will be coded having had the outcome if:

#	Other Outcomes – NON-COVID-19 Related Outcomes
	- They needed oxygen therapy during an episode of fever or respiratory illness within 12 months of randomisation<br>Participants will be coded not having had the outcome if:<br>- They did not need oxygen therapy during an episode of fever or respiratory illness<br>- They did not have an episode of fever or respiratory illness within 12 months of randomisation
25	Admission to critical care for a febrile or respiratory illness (using self-reported questionnaire and/or medical/hospital records), over the 12 months following randomisation<br><br>Admission to critical care following elective intervention will not be counted as part of this outcome.<br><br>CALCULATION<br>The derivation of this outcome will use data from the self-reported questionnaire and/or hospitalisation forms.<br>Participants will be coded as having had the outcome if:<br>- They were admitted to critical care following a febrile or respiratory illness within 12 months of randomisation<br>Participants will be coded as not having had the outcome if:<br>- They were NOT hospitalised during any episode of fever or respiratory illness<br>- They were NOT admitted to critical care during any hospitalised episode of fever or respiratory illness<br>- They did not have an episode of fever or respiratory illness within 12 months of randomisation
26	Need for mechanical ventilation (MV) for a febrile or respiratory illness (using self-reported questionnaire and/or medical/hospital records), over the 12 months following randomisation<br><br>MV required as part of an elective intervention will not be counted as part of this outcome.<br><br>CALCULATION<br>The derivation of this outcome will use data from the self-reported questionnaire and/or hospitalisation forms.<br>Participants will be coded as having had the outcome if:<br>- They needed MV during an episode of fever or respiratory illness within 12 months of randomisation<br>Participants will be coded as not having had the outcome if:<br>- They were NOT hospitalised during any episode of fever or respiratory illness<br>- They did NOT need MV during a hospitalisation for an episode of fever or respiratory illness<br>- They did not have an episode of fever or respiratory illness<br>- within 12 months of randomisation
27	Deaths as a consequence of an episode of fever or respiratory illness over the 12 months following randomisation<br><br>CALCULATION<br>Participants will be coded as having had the outcome if:<br>- They died as a consequence of an episode of fever or respiratory illness within 12 months of randomisation<br>Participants will be coded as not having had the outcome if:<br>- They had an episode of fever or respiratory illness and did NOT die<br>- They did not have an episode of fever or respiratory illness<br>- They died for other reasons (not as a consequence of an episode of fever or respiratory illness)<br>- within 12 months of randomisation
28	Hospitalisation for a febrile or respiratory illness and duration of hospitalisation over the 12 months following randomisation.<br><br>CALCULATION

#	Other Outcomes – NON-COVID-19 Related Outcomes
	The derivation of this outcome will use data from the self-reported questionnaire and/or hospitalisation forms.<br>On participants who were hospitalised due to an episode of fever or respiratory illness, the duration of their hospital stay will be calculated as the difference between date of admission and date of discharge. If a participant experienced >1 episodes of fever or respiratory illness which resulted in hospitalisation within 12 months of randomisation, all the episodes will be included in this analysis, and the overall duration of hospitalisation will be the sum of the duration of each hospitalisation.<br>Participants were hospitalised as a consequence of an episode of fever or respiratory illness within 12 months of randomisation will have their duration of hospitalisation equal to zero
29a	Number of days of unplanned absenteeism for an acute illness or hospitalisation over the 6 months following randomisation.<br><br>CALCULATION<br>The number of days the participant reported unplanned absenteeism (using self-reported questionnaire) for an acute illness or hospitalisation within 6 months of randomisation will be calculated. This will exclude absenteeism for other reason such as elective hospitalisation, issues with vaccination site, mandatory quarantine while not ill, carer leave, annual leave/holidays/planned absence, or pregnancy-related absence. The number of days of unplanned absenteeism for an acute illness or hospitalisation within 6 months of randomisation will be set to missing for participants who don't complete the 6-month self-reported questionnaire.
29b	Number of days of unplanned absenteeism for any reason (using self-reported questionnaire) over the 12 months following randomisation.<br><br>CALCULATION<br>As 28a.
30	Adverse events (AEs) experienced by the participant over the 3 months following randomisation, by type, severity (graded using toxicity grading scale), relationship to intervention of adverse events (AEs) of interest*.<br><br>* AEs of interest are defined as:<br>- Reaction at injection site (pain, tenderness, redness, swelling) of grade 3 (severe) or 4 (potentially life threatening)<br>- Abscess at injection site<br>- Large ulcer (>1.5 cm diameter) at injection site<br>- Keloid scar at injection site<br>- Lymphadenopathy (in region of injection site)<br>- BCG osteitis/osteomyelitis<br>- Disseminated BCG infection (BCG-osis)<br>- Allergic reaction due to IP<br>- Fainting episode, seizures and convulsions following IP administration (recorded on the day of IP administration only)
31	Serious Adverse Events (SAEs) experienced by the participant over the 3 months following randomisation.

4.4. OTHER VARIABLES

DEMOGRAPHY AND BASELINE

Baseline characteristics that will be presented include:

- Sex – Male/Female/Other/Declined/ Missing
- Age*, years
- Body Mass Index (BMI), kg/m^2 – < 18.5 / 18.5 to 24.9/ 25 to 29.9 / >30 / Missing

- Department – Emergency / Intensive Care Unit or High Dependency Unit / Operating Theatre / General Ward / Pharmacy / Practice outside of hospital setting/ Other / Missing
- Role – Administrative-clerical staff / Allied Health / Dentist-dental therapy / Doctor / Nurse-Midwife / Patient Services Assistant-hospital maintenance / Scientist (medical research) / Other / Missing
- Contact with patients, hours - <10 / 10-20 / >20 / Missing
- Confirmed cases of COVID-19 within department – Yes / No / Missing
- Smoking – Yes / No / Missing
- Previous BCG vaccination -No / last BCG dose <1 year ago / 1-5 years ago / >5 years ago / Missing
- Evidence of BCG scar at randomisation – Yes / No / Unsure / Missing
- Positive (>5mm) Tuberculin Skin Test or positive Mantoux test in the past? Yes / No / Unsure
- Previous tuberculosis (TB) exposure – Yes / No / Missing
- Positive PCR or SARS-CoV-2 diagnostic antigen test or serology at randomisation– Yes / No / Missing
- Comorbidities
    - Diabetes – Yes / No / Missing
        - Type 1 diabetes
        - Type 2 diabetes
        - Type 1 and type 2 diabetes
        - Other diabetes
        - Missing
    - Cardiovascular disease– Yes / No / Missing
        - Ischaemic heart disease
        - Congestive heart disease
        - Other cardiovascular disease
        - Hypertension– Yes / No / Missing
        - Missing
    - Chronic respiratory disease– Yes / No / Missing
    - Number of co-morbidities
        - 1
        - 2
        - 3 or more
        - Missing

* Since year of birth rather than DOB is collected for EU participants, age will be calculated using “1-July” in their year of birth as their DOB.

COVID-19-SPECIFIC VACCINES

- Time between randomisation and first COVID-19-specific vaccine dose
- Brand of the first dose of COVID-19-specific vaccine received
    - First and second doses of the same brand
- Number of doses received

OTHER VACCINES

- Time between randomisation and first other vaccine dose
- Type of the first dose of vaccine received
- Number of doses received
- Number of types of vaccine received

PARTICIPANT FOLLOW-UP

- Withdrawal after randomisation
- Reasons for withdrawal.

PROTOCOL DEVIATIONS

- Whether there was a protocol deviation
- Reasons for protocol deviation - Received the opposite intervention/ Did not receive any intervention / Participant randomised twice/ Did not receive the questionnaire in time/ Not able to use the app/ Did not have a swab while indicated / Did not have the blood sample taken / Blood/vaccination not performed on day of randomisation, but later/ Randomised in the wrong strata / Blood sample (3m) taken and re-consent form incomplete / Blood sample taken without correct consent / Blood taken outside window / Blood collection: did not collect the right tube/ Improper preparation of BCG/ Participant received triple the dose of BCG / Problem during blood processing / Delay in delivery of bloods / BCG within 12 months of randomisation / Pregnancy at randomisation / Other
- Whether there was a protocol violation
- Reasons for protocol violations - Participant received twice the dose of BCG / Intravenous injection of BCG / Other

5. STATISTICAL METHODOLOGY

5.1. GENERAL PRINCIPLES

The details of the randomisation groups will be unblinded only once the database has been locked and the SAP has been finalised, approved by the TSC and made publicly available.

Multiple outcomes will be considered in evaluating the effectiveness of the trial intervention. The magnitude of the treatment effect, with 95% confidence interval and p-value, will be estimated for each outcome. Findings will be interpreted based on the magnitude of the treatment effect and in context of one another rather than in isolation considering the patterns and consistency in the findings across outcomes.

The comparison of BCG vs placebo <u>in Stage 2 participants is the primary analysis of interest.</u> It is planned to combine data from the two stages of the trial (Stage 1 + Stage 2) in a meta-analysis for the secondary analysis of the non-COVID19 outcomes only (see Section 9 for details).

5.2. DEFINITION OF BASELINE

Baseline is defined as time of enrolment, captured by date of randomisation (day 0).

5.3. DEFINITION OF THE 6- and 12-MONTH CUT-OFFS

The 6-month period from randomisation will be defined as the date of randomisation date plus 182 days following randomisation.
The 12-month period from randomisation will be defined as the date of randomisation date plus 365 days following randomisation

5.4. DESCRIPTIVE STATISTICS

5.4.1. PARTICIPANT DISPOSITION

All participants who were invited to participate in the BRACE trial will be accounted for as part of the CONSORT flow diagram. The number of participants that were screened but not randomised will be presented and the reasons for their non-participation will be listed. The number of participants who fulfilled eligibility criteria and were recruited will be presented overall and by study centre. The number and proportion of participants who discontinue the study prematurely and/or withdraw during the study will be presented, and the reasons for early withdrawal will be presented by intervention group (BCG or Placebo).
The number of participants with at least one protocol deviation, the number of protocol deviations per participant and the reasons for protocol deviation will be summarised by intervention group (BCG or Placebo).

5.4.2. PARTICIPANT CHARACTERISTICS

The demographic characteristics at randomisation of the participants in the mITT as well in the ITT and safety populations will be presented for each intervention group (BCG or Placebo) using the mean and standard deviation (SD) or median and interquartile range (IQR) for continuous data and using numbers and proportions for categorical data.

5.5. THE ESTIMAND FRAMEWORK

The estimand is the precise description of the intervention effect of interest for a given objective. The estimand is described by the following attributes:

- Population
- Outcome
- Interventions
- Handling of intercurrent events
- Summary measure

An intercurrent event is one that occurs after randomisation and prior to observation of the trial endpoint (primary or secondary). There are two important intercurrent events within this trial:

- the administration of COVID-19-specific vaccine
- the administration of any vaccine (including influenza vaccine and COVID-19-specific vaccine)

Within the analysis of this trial, we will adopt three strategies to handle the intercurrent events:

- Hypothetical Strategy: the aim of this strategy is to estimate the intervention effect of being offered the intervention in the absence of intercurrent events. This strategy involves considering what would have happened if the participant had not had the intercurrent event.
- Treatment Policy Strategy: the aim of this strategy is to assess the effect of being offered the intervention irrespective of any intercurrent events. In this strategy intercurrent events are ignored and all outcome data are used regardless of occurrence of the intercurrent event.
- Principal stratum strategy: this strategy considers measurements in a subgroup of participants where the intercurrent event(s) is not likely or less likely to occur. This strategy classifies participants according to their potential occurrence of an intercurrent event in both study groups.

The estimands of interest in this trial are outlined in section 6.

5.6. ANALYSIS SOFTWARE

All analyses will be performed using Stata Release 16.1 or later.

6. PRIMARY OUTCOMES

6.1. ESTIMANDS

Objective	Estimand
To determine if BCG vaccination compared with placebo reduces the incidence of symptomatic COVID-19 in the absence of a COVID-19 specific vaccine, over the 6 months following randomisation, in healthcare workers who did not have a previous SARS-CoV-2 positive test result when assessed at time of randomisation.	Estimand 1.1 [Primary analysis]<br><br>Population: mITT population<br>Outcome: symptomatic COVID-19 by 6 months<br>Interventions: BCG vs Placebo<br>Handling of Intercurrent events:<br>- COVID-19 specific vaccine (Hypothetical Strategy)<br>- any other vaccine (Treatment Policy strategy)<br>Summary Measure: Adjusted* difference in proportion of participants
To determine if BCG vaccination compared with placebo reduces the incidence of severe COVID-19 in the absence of a COVID-19 specific vaccine, over the 6 months following randomisation, in healthcare workers who did not have a previous SARS-CoV-2 positive test result when assessed at time of randomisation.	Estimand 2.1 [Primary analysis]<br><br>Population: mITT population<br>Outcome: severe COVID-19 by 6 months<br>Interventions: BCG vs Placebo<br>Handling of Intercurrent events:<br>- COVID-19 specific vaccine (Hypothetical Strategy)<br>- any other vaccine (Treatment Policy strategy)<br>Summary Measure: Adjusted* difference in proportion of participants
** 1) adjusted for stratification factors used at randomisation (age group, presence of comorbidity, and geographical location); 2) adjusted for stratification factors used at randomisation + sex, BMI at baseline, BCG vaccine before randomisation;*	

Analytical approach for the primary estimands

For the primary analysis of each of the primary outcomes, receiving a COVID-19 vaccine will be handled using a hypothetical strategy; participants who receive a COVID-19-specific vaccine will have their data used up to the date of their first dose of COVID-19-specific vaccine (data collected after the COVID-19-specific vaccine will be ignored). These primary analyses will be conducted on the mITT population including only Stage 2 participants.

6.2. PRIMARY ANALYSIS

6.2.1. SUMMARY STATISTICS

The outcomes of symptomatic COVID-19 and severe COVID-19 prior to 6 months will be described by intervention group as the absolute number of participants with the event. The primary outcome of severe COVID-19 by 6 months will also be presented as the number of participants within 5 categories, according to the most severe event they encountered over the 6-month period, by intervention group:

- Severe COVID-19 which resulted in death
- Severe COVID-19 which resulted in hospitalisation
- Non-hospitalised severe COVID-19

  This category will be further broken down as:
  - Non-hospitalised severe COVID-19 who were confined to bed for 3 consecutive days or more
  - Non-hospitalised severe COVID-19 who were too sick to go to work for 3 consecutive days or more

The numbers of participants whose follow-up data is censored due to:

- missing PCR, RAT or serology test result,
- incomplete data entry,
- drop-out from the study, and
- intercurrent event (COVID-19 specific vaccine / Any other vaccine)

will also be reported separately by intervention group.

6.2.2. ANALYSIS

The outcomes of symptomatic COVID-19 and severe COVID-19 prior to 6 months will be compared between the BCG group and the placebo group recruited in Stage 2 using a difference in proportions. This will be estimated using a time-to-event analysis. The first analysis will be adjusted for stratification factors used in randomisation, namely age group (<40 years; 40 to 59 years; >=60 years), presence of comorbidity (any of diabetes, chronic respiratory disease, cardiovascular disease, hypertension), and geographical location (Europe/Australia/South America). Although randomisation was stratification was by participating hospitals and clinics, for the analysis it was decided to group the sites into the 3 regions due to the high number of randomising sites which could lead to computational problems. For participants who were randomised in the incorrect stratum, the correct stratum will be used as covariate in the model. To do this analysis, the survival curve for each combination of strata and randomised group will be calculated using a flexible parametric survival model (Royston-Parmar model[2]). This will be done using the *stpm2* command in Stata, with the *meansurv* and *timevar* options specified. The average survival curve for each randomised group will be estimated as a weighted average of the corresponding stratum-specific survival curves, with weights proportional to the number of individuals in each stratum in the randomised group at baseline. The parameter of interest will be the (adjusted) point estimate for the difference in proportion with the event at 6 months between BCG and control group. A two-sided bias-corrected 95% CI for the difference in proportion (BCG – Control) will be calculated with bootstrap standard errors using the Stata bootstrap command. A bootstrap p-value will also be calculated. The bootstrapping will sample 1000 times (with replacement) and be stratified by the stratification factors. Note because modelling will be used to estimate the difference in proportion, the results from this analysis will not correspond directly to the raw summaries that will be presented.

A Kaplan-Meier survival curve will also be presented by treatment arm.

The proportional hazards assumption will be checked when running these analyses.

The analysis of both primary outcomes (for all estimands 1.1-6 and 2.1-6) will then be repeated including adjustment for the following baseline covariates which are expected to be associated with COVID-19:

1. Sex (Female / Male)
2. BMI at baseline (< 30 / ≥ 30 kg/m2)
3. BCG vaccination before enrolling into the trial (Yes / No)

Should the fully adjusted models not run when all the all the covariates listed above are included in the model, the covariates will be removed starting from the bottom of the list to the top, until the adjusted model runs.

Handling of missing data

Protocol version 12 stated that, for the primary analysis, multiple imputation (MI) would be used to handle missing data if >10% of the primary outcome data were missing. During the trial it became apparent that a number of participants would have partial data on the primary outcomes due to the expansion of COVID-19 vaccination programs. Therefore, survival analysis will be used to estimate the proportion with symptomatic COVID-19 or severe COVID-19, which enables this partial follow-up data to be included in the analysis. Strategies to handle missingness due to non-testing within trigger and severe episodes is also described in section 6.2.3. Given the survival analysis strategy enables all participants with at least some follow-up data to be included in the analysis, the analysis will be conducted using the available data with no MI.

6.2.3. DATES AND CENSORING FOR 6-MONTH FOLLOW-UP

Censoring dates used for the analysis of the primary outcomes are described in table 2 below.

Table 2. Dates and censoring algorithm

MAIN ANALYSIS	SENSITIVITY ANALYSIS*
Censored at the earlier of:<br>[A] their first COVID-19 specific vaccine dose or<br>[B] day 182 of their participation in the trial or<br>[C] their last entered date prior to which there are more than 3 consecutive days of missing data which aren't ruled out by negative serology or<br>[D] their first day with symptoms for their first episode of illness with trigger/severe symptoms, which the algorithm in Figure 4 cannot ascertain be a COVID-19 episode (categorised as missing in Fig.4)<br><br><u>unless</u> the definition of the outcome is met (as per Fig. 4) and first day with symptoms for their first symptomatic/severe COVID-19 episode <u>precedes all the events above, [A] – [D].</u>	Censored at the earlier of:<br>[A] or<br>[B] or<br>[E] date of withdrawal/last contact<br><br>unless the definition of the outcome is met (as per Fig. 4) and first day with symptoms for their first symptomatic/severe COVID-19 episode <u>precedes [A], [B] and [E]</u>.<br><br>[Episodes of illness with trigger/severe symptoms which the algorithm in Figure 4 cannot ascertain be a COVID-19 episode will be ignored from the censoring algorithm.]

*See section sensitivity analysis 3

6.3. SUPPLEMENTARY ANALYSES

The following supplementary analyses will be conducted on primary outcomes 1 and 2 with the intent to provide additional insights into the treatment effect:

i. Including follow-up after first dose of any COVID-19-specific vaccine (ie the intercurrent event of COVID-19-specific vaccine handled using a Treatment Policy Strategy. This analysis is summarised in estimands 1.2 and 2.2 below.

ii. Excluding COVID-19 episodes (either trigger or severe) starting ≤14 days from date of randomisation (ie the intercurrent event of symptomatic/severe COVID-19 in the first 14 days post randomisation, handled using a Principal Stratum Strategy). This analysis is summarised in estimands 1.3 and 2.3 below). For this analysis, time at risk of COVID-19 will start on the 15th day post randomisation date (as opposed to date of randomisation as for the primary analysis). In line with the definition of mITT that excludes participants who were exposed to COVID-19 prior to being randomised into the study, participants who:
   - had a COVID-19 episodes (either symptomatic or severe), or
   - had a trigger symptoms/severe episode reported, which the algorithm in Figure 4 cannot ascertain be a COVID-19 episode

   starting ≤14 days from date of randomisation, will be excluded from this analysis.

iii. Censoring participants at the time of any subsequent vaccine (ie the intercurrent event of any vaccine, including influenza vaccination and COVID-19-specific vaccine, handled by the Hypothetical Strategy, summarised in estimands 1.4 and 2.4 below).

iv. on the ITT population (as summarised in estimands 1.5 and 2.5 below)

v. Treatment Policy Strategy on the ITT population (summarised in estimands 1.6 and 2.6 below).

The following table summarises the estimands of secondary interest around primary outcomes 1 and 2.

Objective	Estimand
To determine if BCG vaccination compared with placebo reduces the incidence of symptomatic COVID-19 irrespective of receiving a COVID-19-specific vaccine or any other vaccine, over the 6 months following randomisation, in healthcare workers who did not have a previous SARS-CoV-2 positive test result when assessed at time of randomisation.	Estimand 1.2 [Supplementary analysis i.]<br><br>Population: as for estimand 1.1<br>Outcome: as for estimand 1.1<br>Interventions: as for estimand 1.1<br>Handling of Intercurrent events:<br>- COVID-19 specific vaccine (Treatment Policy strategy)<br>- any other vaccine (Treatment Policy strategy)<br>Summary Measure: as for estimand 1.1
To determine if BCG vaccination compared with placebo reduces the incidence of symptomatic COVID-19 following the 14 days after randomisation in the absence of any COVID-19-specific vaccine, over the 6 months, in healthcare workers who did not have a previous SARS-CoV-2 positive test result when assessed at time of randomisation.	Estimand 1.3 [Supplementary analysis ii.]<br><br>Population: as for estimand 1.1<br>Outcome: as for estimand 1.1<br>Interventions: as for estimand 1.1<br>Handling of Intercurrent events: as for estimand 1.1<br>- COVID-19 specific vaccine (Hypothetical strategy)<br>- any other vaccine (Treatment Policy strategy)<br>- COVID-19 in the 14 days post randomisation (Principal Stratum Strategy)<br>Summary Measure: as for estimand 1.1
To determine if BCG vaccination compared with placebo reduces the incidence of symptomatic COVID-19 in the absence of any vaccine (including COVID-19-specific vaccine), over the 6 months following randomisation, in healthcare workers who did not have a previous SARS-CoV-2 positive test result when assessed at time of randomisation.	Estimand 1.4 [Supplementary analysis iii.]<br><br>Population: as for estimand 1.1<br>Outcome: as for estimand 1.1<br>Interventions: as for estimand 1.1<br>Handling of Intercurrent events:<br>- COVID-19 specific vaccine (Hypothetical Strategy)<br>- any other vaccine (Hypothetical strategy)<br>Summary Measure: as for estimand 1.1
To determine if BCG vaccination compared with placebo reduces the incidence of symptomatic COVID-19 in the absence of a COVID-19-specific vaccine, over the 6 months following randomisation, in healthcare workers exposed to SARS-CoV-2.	Estimand 1.5 [Supplementary analysis iv.]<br><br>Population: ITT population<br>Outcome: as for estimand 1.1<br>Interventions: as for estimand 1.1<br>Handling of Intercurrent events: as for estimand 1.1<br>Summary Measure: as for estimand 1.1
To determine if BCG vaccination compared with placebo reduces the incidence of symptomatic COVID-19 irrespective of receiving a COVID-19-specific vaccine or any other vaccine, over the 6 months following randomisation, in healthcare workers.	Estimand 1.6 [Supplementary analysis v.]<br><br>Population: ITT population<br>Outcome: as for estimand 1.1<br>Interventions: as for estimand 1.1<br>Handling of Intercurrent events: as for estimand 1.2<br>Summary Measure: as for estimand 1.1
To determine if BCG vaccination compared with placebo reduces the incidence of severe COVID-19 irrespective of receiving a COVID-19 specific vaccine or any other vaccine, over the 6 months following randomisation, in healthcare workers who did not have a previous SARS-	Estimand 2.2 [Supplementary analysis i.]<br><br>Population: as for estimand 2.1<br>Outcome: as for estimand 2.1<br>Interventions: as for estimand 2.1<br>Handling of Intercurrent events:

Objective	Estimand
CoV-2 positive test result when assessed at time of randomisation.	- COVID-19 specific vaccine (Treatment Policy strategy)<br>- any other vaccine (Treatment Policy strategy)<br>Summary Measure: as for estimand 2.1
To determine if BCG vaccination compared with placebo reduces the incidence of severe COVID-19 following the 14 days after randomisation in the absence of any COVID-19 specific vaccine, over the 6 months, in healthcare workers who did not have a previous SARS-CoV-2 positive test result when assessed at time of randomisation.	Estimand 2.3 [Supplementary analysis ii.]<br><br>Population: as for estimand 2.1<br>Outcome: as for estimand 2.1<br>Interventions: as for estimand 2.1<br>Handling of Intercurrent events: as for estimand 2.1<br>- COVID-19 specific vaccine (Hypothetical strategy)<br>- any other vaccine (Treatment Policy strategy)<br>- COVID-19 in the 14 days post randomisation (Principal Stratum Strategy)<br>Summary Measure: as for estimand 2.1
To determine if BCG vaccination compared with placebo reduces the incidence of severe COVID-19 in the absence of any vaccine (including a COVID-19 specific vaccine), over the 6 months following randomisation, in healthcare workers who did not have a previous SARS-CoV-2 positive test result when assessed at time of randomisation.	Estimand 2.4 [Supplementary analysis iii.]<br><br>Population: as for estimand 2.1<br>Outcome: as for estimand 2.1<br>Interventions: as for estimand 2.1<br>Handling of Intercurrent events:<br>- COVID-19 specific vaccine (Hypothetical Strategy)<br>- any other vaccine (Hypothetical strategy)<br>Summary Measure: as for estimand 2.1
To determine if BCG vaccination compared with placebo reduces the incidence of severe COVID-19 in the absence of a COVID-19 specific vaccine, over the 6 months following randomisation, in healthcare workers exposed to SARS-CoV-2.	Estimand 2.5 [Supplementary analysis iii.]<br><br>Population: ITT population<br>Outcome: as for estimand 2.1<br>Interventions: as for estimand 2.1<br>Handling of Intercurrent events: as for estimand 2.1<br>Summary Measure: as for estimand 2.1
To determine if BCG vaccination compared with placebo reduces the incidence of severe COVID-19 irrespective of receiving a COVID-19-specific vaccine or any other vaccine, over the 6 months following randomisation, in healthcare workers.	Estimand 2.6 [Supplementary analysis v.]<br><br>Population: ITT population<br>Outcome: as for estimand 2.1<br>Interventions: as for estimand 2.1<br>Handling of Intercurrent events: as for estimand 2.2<br>Summary Measure: as for estimand 2.1
** 1) adjusted for stratification factors used at randomisation (age group, presence of comorbidity, and geographical location); 2) adjusted for stratification factors used at randomisation + sex, BMI at baseline, BCG vaccine before randomisation;*	

The analyses for estimands 1.2-1.6 and 2.2-2.6 will be conducted using the same methodology as for the primary analysis (estimand 1.1 and 2.1, as specified in section 6.2.2).

6.4. SUBGROUP ANALYSES

The following sub-group analyses will be performed (only for estimands 1.1 and 2.1, unless otherwise indicated), but since we have not powered the trial to consider sub-groups, the results will be considered exploratory only. These sub-group analyses will examine the evidence for differences in the effect of the intervention between the sub-groups. The intervention effect in each sub-group and their 95% confidence intervals will be presented, together with the p-value for the intervention-by-subgroup interaction, as a guide to the strength of the evidence for an interaction.

1 – By age group (stratification factor at randomisation)
Subgroups will be defined by age at randomisation, as follows:
- <40 years vs.
- 40 to 59 years vs.
- >=60 years

2 – By presence of comorbidities
Subgroups will be:
- Presence of comorbidity at randomisation (any of diabetes, chronic respiratory disease, cardiovascular disease -including hypertension/high blood pressure, and obesity defined as BMI >= 30 kg/m^2) vs.
- Absence of comorbidity

2a – By presence of diabetes
Subgroups will be:
- Presence of diabetes at randomisation vs.
- Absence of diabetes at randomisation

2b – By presence of chronic respiratory disease
Subgroups will be:
- Presence of chronic respiratory disease at randomisation vs.
- Absence of chronic respiratory disease at randomisation

2c – By presence of ANY cardiovascular disease
Subgroups will be:
- Presence of cardiovascular disease at randomisation vs.
- Absence of cardiovascular disease at randomisation

2d – By presence of hypertension/high blood pressure
Subgroups will be:
- Presence of hypertension at randomisation vs.
- Absence of hypertension at randomisation

2e – By presence of obesity (BMI>=30 kg/m2)
Subgroups will be:
- Presence of obesity at randomisation vs
- Absence of obesity at randomisation

3 – By geographical Location
Sub-groups will be:
- Australia vs
- Europe vs
- South America

4 – By sex
Sub-groups will be:
- Females vs
- Males

5 – By BCG in the past or not
Sub-groups will be:
- Participants who received BCG vaccine before participating in the trial vs

- Participants who never received BCG vaccine before participating in the trial

Prior BCG vaccination status will be ascertained by self-reported answer to the question “Have you been vaccinated with BCG in the past?”.

6 – By baseline serology results to SARS-CoV-2 (negative or non-negative) [ITT population only]
Sub-groups will be:

- Participants with negative serology to SARS-CoV-2 when enrolling into the trial (participants at the Brazilian sites will also need to show negative PCR on a respiratory sample or a negative SARS-CoV-2 diagnostic antigen test approved by the local jurisdiction's public health policy at the time of randomisation) vs
- Participants with non-negative (ie positive/missing/indeterminant) serology to SARS-CoV-2 when enrolling into the trial (Applicable only to participants at the Brazilian sites: positive/missing/indeterminant PCR on a respiratory sample or a positive/missing/indeterminant SARS-CoV-2 diagnostic antigen test approved by the local jurisdiction's public health policy at the time of randomisation)

This subgroup analysis will only be run on the ITT population (estimand 1.5 and 2.5).

6.5. SENSITIVITY ANALYSES

Sensitivity analysis 1 – BCG/Placebo vaccination date (as opposed to their randomisation date)
The primary analysis includes follow up data from the randomisation date (day 0), which is also when most participants received their trial BCG/placebo injection. However, a small number of participants received the intervention several days or weeks following randomisation. A sensitivity analysis on estimands 1.1 and 2.1 will be run, to have these participants follow up data start on the actual vaccination date (as opposed to their randomisation date).

Objective	Estimand
To determine if BCG vaccination compared with placebo reduces the incidence of symptomatic COVID-19 in the absence of a COVID-19 specific vaccine, over the 6 months following vaccination, in healthcare workers who did not have a previous SARS-CoV-2 positive test result when assessed at time of randomisation.	Estimand 1.1_s1 [Sensitivity analysis 1]<br><br>Population: as for estimand 1.1<br>Outcome: symptomatic COVID-19 by 6 months following vaccination<br>Interventions: as for estimand 1.1<br>Handling of Intercurrent events: as for estimand 1.1<br>Summary Measure: as for estimand 1.1
To determine if BCG vaccination compared with placebo reduces the incidence of severe COVID-19 in the absence of a COVID-19 specific vaccine, over the 6 months following vaccination, in healthcare workers who did not have a previous SARS-CoV-2 positive test result when assessed at time of randomisation.	Estimand 2.1_s1 [Sensitivity analysis 1]<br><br>Population: as for estimand 2.1<br>Outcome: severe COVID-19 by 6 months following vaccination<br>Interventions: as for estimand 2.1<br>Handling of Intercurrent events: as for estimand 2.1<br>Summary Measure: as for estimand 2.1
** 1) adjusted for stratification factors used at randomisation (age group, presence of comorbidity, and geographical location); 2) adjusted for stratification factors used at randomisation + sex, BMI at baseline, BCG vaccine before randomisation;*	

Sensitivity analysis 2 – Clinical algorithm based only on combined PCR/RAT result (Fig. 2) using the ITT population (i.e. disregarding all serology testing and baseline PCR in Brazil)
As a second sensitivity analysis on estimands 1.1 and 2.1, for the derivation of the primary outcomes, episodes of illness will be re-categorised as COVID-19 episodes (either symptomatic or severe), non-COVID-19 episodes, episodes with missing information using only the combination of the results from RAT and PCR tests (as in Figure 2).

Objective	Estimand
To determine if BCG vaccination compared with placebo reduces the incidence of symptomatic COVID-19 (determined using PCR/RAT tests only) in the absence of a COVID-19 specific vaccine, over the 6 months following randomisation, in healthcare workers.	Estimand 1.1_s2 [Sensitivity analysis 2]<br><br>Population: ITT population<br>Outcome: symptomatic COVID-19 by 6 months determined using PCR/RAT tests only<br>Interventions: as for estimand 1.1<br>Handling of Intercurrent events: as for estimand 1.1<br>Summary Measure: as for estimand 1.1
To determine if BCG vaccination compared with placebo reduces the incidence of severe COVID-19 (determined using PCR/RAT tests only) in the absence of a COVID-19 specific vaccine, over the 6 months following randomisation, in healthcare workers.	Estimand 2.1_s2 [Sensitivity analysis 2]<br><br>Population: ITT population<br>Outcome: severe COVID-19 by 6 months determined using PCR/RAT tests only<br>Interventions: as for estimand 2.1<br>Handling of Intercurrent events: as for estimand 2.1<br>Summary Measure: as for estimand 2.1
** 1) adjusted for stratification factors used at randomisation (age group, presence of comorbidity, and geographical location); 2) adjusted for stratification factors used at randomisation + sex, BMI at baseline, BCG vaccine before randomisation;*	

Sensitivity analysis 3

A third sensitivity analysis on estimands 1.1-1.6 and 2.1-2.6 will adopt a modified version of the censoring rule section as presented in table 2, in the column "SENSITIVITY ANALYSIS" (section 6.2.3).

7. SECONDARY COVID-19 RELATED OUTCOMES

Similarly to the analyses of the primary outcomes, the analyses of the COVID-19 related secondary outcomes will be conducted following three different approaches, specifically:

1. Censoring participants at the time of their COVID-19 specific vaccine (ie the intercurrent event of COVID-19-specific vaccine, will be handled using a Hypothetical Strategy)
2. Including follow-up after first dose of any COVID-19-specific vaccine (ie the intercurrent event of COVID-19-specific vaccine, will be handled using a Treatment Policy Strategy)
3. Censoring participants at the time of any vaccine (ie the intercurrent event of any vaccine, including influenza vaccination and COVID-19-specific vaccine, will be handled using a Hypothetical Strategy)

The analyses will be conducted on the mITT population, unless otherwise indicated, and including only Stage 2 participants.

7.1. SYMPTOMATIC COVID-19 AND SEVERE COVID-19 BY 12MONTHS (#3 and #4)

7.1.1. ESTIMANDS

Objective	Estimand
To determine if BCG vaccination compared with placebo reduces the incidence of symptomatic COVID-19 in the absence of a COVID-19 specific vaccine, over the 12 months following randomisation, in healthcare workers who did not have a previous SARS-CoV-2 positive test result when assessed at time of randomisation.	Estimand 3.1<br><br>Population: mITT population<br>Outcome: symptomatic COVID-19 by 12 months<br>Interventions: BCG vs Placebo<br>Handling of Intercurrent events:<br>- COVID-19 specific vaccine (Hypothetical Strategy)<br>- any other vaccine (Treatment Policy strategy)<br>Summary Measure: Adjusted* difference in proportion of participants

Objective	Estimand
To determine if BCG vaccination compared with placebo reduces the incidence of symptomatic COVID-19 irrespective of receiving a COVID-19 specific vaccine or any other vaccine, over the 12 months following randomisation, in healthcare workers who did not have a previous SARS-CoV-2 positive test result when assessed at time of randomisation.	Estimand 3.2<br><br>Population: as for estimand 3.1<br>Outcome: as for estimand 3.1<br>Interventions: as for estimand 3.1<br>Handling of Intercurrent events:<br>- COVID-19 specific vaccine (Treatment Policy strategy)<br>- any other vaccine (Treatment Policy strategy)<br>Summary Measure: as for estimand 3.1
To determine if BCG vaccination compared with placebo reduces the incidence of symptomatic COVID-19 in the absence of a COVID-19 specific vaccine, over the 12 months following the 14 days after randomisation, in healthcare workers who did not have a previous SARS-CoV-2 positive test result when assessed at time of randomisation.	Estimand 3.3<br><br>Population: as for estimand 3.1<br>Outcome: symptomatic COVID-19, but excluding COVID-19 episodes that started in the 14 days window after randomisation<br>Interventions: as for estimand 3.1<br>Handling of Intercurrent events: as for estimand 3.1<br>Summary Measure: as for estimand 3.1
To determine if BCG vaccination compared with placebo reduces the incidence of symptomatic COVID-19 in the absence of any other vaccine (including COVID-19 specific vaccine), over the 12 months following randomisation, in healthcare workers who did not have a previous SARS-CoV-2 positive test result when assessed at time of randomisation.	Estimand 3.4<br><br>Population: as for estimand 3.1<br>Outcome: as for estimand 3.1<br>Interventions: as for estimand 3.1<br>Handling of Intercurrent events:<br>- COVID-19 specific vaccine (Hypothetical Strategy)<br>- any other vaccine (Hypothetical strategy)<br>Summary Measure: as for estimand 3.1
To determine if BCG vaccination compared with placebo reduces the incidence of symptomatic COVID-19 in the absence of a COVID-19 specific vaccine, over the 12 months following randomisation, in healthcare workers exposed to SARS-CoV-2.	Estimand 3.5<br><br>Population: ITT population<br>Outcome: as for estimand 3.1<br>Interventions: as for estimand 3.1<br>Handling of Intercurrent events: as for estimand 3.1<br>Summary Measure: as for estimand 3.1
To determine if BCG vaccination compared with placebo reduces the incidence of symptomatic COVID-19 irrespective of receiving a COVID-19 specific vaccine or any other vaccine, over the 12 months following randomisation, in healthcare workers.	Estimand 3.6<br><br>Population: ITT population<br>Outcome: as for estimand 3.1<br>Interventions: as for estimand 3.1<br>Handling of Intercurrent events: as for estimand 3.2<br>Summary Measure: as for estimand 3.1
To determine if BCG vaccination compared with placebo reduces the incidence of severe COVID-19 in the absence of a COVID-19 specific vaccine, over the 12 months following randomisation, in healthcare workers who did not have a previous SARS-CoV-2 positive test result when assessed at time of randomisation.	Estimand 4.1<br><br>Population: mITT population<br>Outcome: severe COVID-19 by 12 months<br>Interventions: BCG vs Placebo<br>Handling of Intercurrent events:<br>- COVID-19 specific vaccine (Hypothetical Strategy)<br>- any other vaccine (Treatment Policy strategy)<br>Summary Measure: Adjusted* difference in proportion of participants
To determine if BCG vaccination compared with placebo reduces the incidence of severe COVID-19 irrespective of receiving a COVID-19 specific vaccine or any other vaccine, over the 12 months following	Estimand 4.2<br><br>Population: as for estimand 4.1<br>Outcome: as for estimand 4.1

Objective	Estimand
randomisation, in healthcare workers who did not have a previous SARS-CoV-2 positive test result when assessed at time of randomisation.	Interventions: as for estimand 4.1<br>Handling of Intercurrent events:<br>- COVID-19 specific vaccine (Treatment Policy strategy)<br>- any other vaccine (Treatment Policy strategy)<br>Summary Measure: as for estimand 4.1
To determine if BCG vaccination compared with placebo reduces the incidence of severe COVID-19 in the absence of a COVID-19 specific vaccine, over the 12 months following the 14 days after randomisation, in healthcare workers who did not have a previous SARS-CoV-2 positive test result when assessed at time of randomisation.	Estimand 4.3<br><br>Population: as for estimand 4.1<br>Outcome: severe COVID-19, but excluding severe COVID-19 episodes that started in the 14 days window after randomisation<br>Interventions: as for estimand 4.1<br>Handling of Intercurrent events: as for estimand 4.1<br>Summary Measure: as for estimand 4.1
To determine if BCG vaccination compared with placebo reduces the incidence of severe COVID-19 in the absence of any vaccine (including COVID-19 specific vaccine), over the 12 months following randomisation, in healthcare workers who did not have a previous SARS-CoV-2 positive test result when assessed at time of randomisation.	Estimand 4.4<br><br>Population: as for estimand 4.1<br>Outcome: as for estimand 4.1<br>Interventions: as for estimand 4.1<br>Handling of Intercurrent events:<br>- COVID-19 specific vaccine (Hypothetical Strategy)<br>- any other vaccine (Hypothetical strategy)<br>Summary Measure: as for estimand 4.1
To determine if BCG vaccination compared with placebo reduces the incidence of severe COVID-19 in the absence of a COVID-19 specific vaccine, over the 12 months following randomisation, in healthcare workers.	Estimand 4.5<br><br>Population: ITT population<br>Outcome: as for estimand 4.1<br>Interventions: as for estimand 4.1<br>Handling of Intercurrent events: as for estimand 4.1<br>Summary Measure: as for estimand 4.1
To determine if BCG vaccination compared with placebo reduces the incidence of severe COVID-19 irrespective of receiving a COVID-19 specific vaccine or any other vaccine, over the 12 months following randomisation, in healthcare workers.	Estimand 4.6<br><br>Population: ITT population<br>Outcome: as for estimand 4.1<br>Interventions: as for estimand 4.1<br>Handling of Intercurrent events: as for estimand 4.2<br>Summary Measure: as for estimand 4.1
** 1) adjusted for stratification factors used at randomisation (age group, presence of comorbidity, and geographical location); 2) adjusted for stratification factors used at randomisation + sex, BMI at baseline, BCG vaccine before randomisation;*	

7.1.2. ANALYSIS

Since outcomes 3 and 4 are the equivalent of the primary outcomes 1 and 2 over 12 months post randomisation, the same analyses that are presented in section 6.2 will be applied on these outcomes (summarised in estimands 3.1 to 3.6 for symptomatic COVID-19 by 12 months and in estimands 4.1 to 4.6 for severe COVID -19 outcome)

7.1.3. ADDITIONAL ANALYSIS

Additional outcomes 3 and 4 will be compared between the following groups:

- Participants who received BCG vaccine at recruitment AND who show evidence of the scar left by this vaccine 12 months post randomisation

- Participants who received placebo AND participants who received BCG vaccine at recruitment but don't show evidence of the scar left by this vaccine 12 months post randomisation

The same analyses that are presented in section 6.2 will be run.

7.1.4. SUBGROUP ANALYSIS

The following subgroup analyses (described in detail in section 6.4) will be conducted for estimands 3.1 and 4.1:

- Sub-Group analysis 1 – Age group (stratification factor at randomisation)
- Sub-Group analyses 2, 2a, 2b, 2c, 2d, 2e – Presence of comorbidities
- Sub-Group analysis 3 – Geographical Location
- Sub-Group analysis 4 – Sex
- Sub-Group analysis 5 – BCG in the past or not
- Sub-Group analysis 6 – Serology results to SARS-CoV-2 at enrolment (ITT pop only, estimand 3.5 and 4.5)

Additionally, a second categorisation of subgroup analysis 5 will be done to include evidence of a scar due to BCG vaccine in the past, as assessed by the immuniser and the photo of the scar review by clinical BRACE team unblinded to the treatment allocation. The will be conducted as follows:

- Sub-Group analysis 5a – By BCG in the past or not (as confirmed by scar evidence)
    - Participants who received BCG vaccine before participating in the trial and show evidence of a scar vs
    - Participants who never received BCG vaccine before participating in the trial and show no evidence of a scar

This subgroup analysis will exclude those participants who reported prior BCG vaccine but have no evidence of a scar, and those who did not report prior BCG vaccine but have evidence of a scar.

7.2. TIME TO FIRST SYMPTOM OF COVID-19 (#5a and #5b)

7.2.1.ESTIMANDS

Objective	Estimand
To determine if BCG vaccination compared with placebo prolongs the time to first SARS-CoV-2-proven respiratory illness in the absence of a COVID-19 specific vaccine, measured over 6 months following randomisation in healthcare workers who did not have a previous SARS-CoV-2 positive test result when assessed at time of randomisation.	Estimand 5a.1<br><br>Population: mITT population<br>Outcome: time to COVID-19 by 6 months<br>Interventions: BCG vs Placebo<br>Handling of Intercurrent events:<br>- COVID-19 specific vaccine (Hypothetical Strategy)<br>- any other vaccine (Treatment Policy strategy)<br>Summary Measure: Adjusted* hazard ratios
To determine if BCG vaccination compared with placebo prolongs the time to first SARS-CoV-2-proven respiratory illness in the absence of a COVID-19 specific vaccine, measured over 12 months following randomisation in healthcare workers who did not have a previous SARS-CoV-2 positive test result when assessed at time of randomisation.	Estimand 5b.1<br><br>Population: mITT population<br>Outcome: time to COVID-19 by 12 months<br>Interventions: BCG vs Placebo<br>Handling of Intercurrent events:<br>- COVID-19 specific vaccine (Hypothetical Strategy)<br>- any other vaccine (Treatment Policy strategy)<br>Summary Measure: Adjusted* hazard ratios
To determine if BCG vaccination compared with placebo prolongs the time to first SARS-CoV-2-	Estimand 5a.2

Objective	Estimand
proven respiratory illness irrespective of receiving a COVID-19 specific vaccine or any other vaccine, measured over 6 months following randomisation in healthcare workers who did not have a previous SARS-CoV-2 positive test result when assessed at time of randomisation.	Population: as 5a.1<br>Outcome: as 5a.1<br>Interventions: as 5a.1<br>Handling of Intercurrent events:<br>- COVID-19 specific vaccine (Treatment Policy strategy)<br>- any other vaccine (Treatment Policy strategy)<br>Summary Measure: as 5a.1
To determine if BCG vaccination compared with placebo prolongs the time to first SARS-CoV-2-proven respiratory illness irrespective of receiving a COVID-19 specific vaccine or any other vaccine, measured over 12 months following randomisation in healthcare workers who did not have a previous SARS-CoV-2 positive test result when assessed at time of randomisation.	Estimand 5b.2<br><br>Population: as 5b.1<br>Outcome: as 5b.1<br>Interventions: as 5b.1<br>Handling of Intercurrent events:<br>- COVID-19 specific vaccine (Treatment Policy strategy)<br>- any other vaccine (Treatment Policy strategy)<br>Summary Measure: as 5b.1
To determine if BCG vaccination compared with placebo prolongs the time to first SARS-CoV-2-proven respiratory illness following the 14 days after randomisation irrespective of receiving a COVID-19 specific vaccine or any other vaccine, measured over the 6 months in healthcare workers who did not have a previous SARS-CoV-2 positive test result when assessed at time of randomisation.	Estimand 5a.3<br><br>Population: as 5a.1<br>Outcome: time to COVID-19 by 6 months, but excluding COVID-19 episodes that started in the 14 days window after randomisation<br>Interventions: as 5a.1<br>Handling of Intercurrent events: as 5a.1<br>Summary Measure: as 5a.1
To determine if BCG vaccination compared with placebo prolongs the time to first SARS-CoV-2-proven respiratory illness following the 14 days after randomisation irrespective of receiving a COVID-19 specific vaccine or any other vaccine, measured over the 12 months in healthcare workers who did not have a previous SARS-CoV-2 positive test result when assessed at time of randomisation.	Estimand 5b.3<br><br>Population: as 5b.1<br>Outcome: time to COVID-19 by 12 months, but excluding COVID-19 episodes that started in the 14 days window after randomisation<br>Interventions: as 5b.1<br>Handling of Intercurrent events: as 5a.1<br>Summary Measure: as 5b.1
To determine if BCG vaccination compared with placebo prolongs the time to first SARS-CoV-2-proven respiratory illness in the absence of any vaccine, measured over 6 months following randomisation in healthcare workers who did not have a previous SARS-CoV-2 positive test result when assessed at time of randomisation.	Estimand 5a.4<br><br>Population: as 5a.1<br>Outcome: as 5a.1<br>Interventions: as 5a.1<br>Handling of Intercurrent events:<br>- COVID-19 specific vaccine (Hypothetical Strategy)<br>- any other vaccine (Hypothetical Strategy)<br>Summary Measure: as 5a.1
To determine if BCG vaccination compared with placebo prolongs the time to first SARS-CoV-2-proven respiratory illness in the absence of any vaccine, measured over 12 months following randomisation in healthcare workers who did not have a previous SARS-CoV-2 positive test result when assessed at time of randomisation.	Estimand 5b.4<br><br>Population: as for estimand 5b.1<br>Outcome: as for estimand 5b.1<br>Interventions: as for estimand 5b.1<br>Handling of Intercurrent events:<br>- COVID-19 specific vaccine (Hypothetical Strategy)<br>- any other vaccine (Hypothetical Strategy)<br>Summary Measure: as for estimand 5b.1

Objective	Estimand
To determine if BCG vaccination compared with placebo prolongs the time to first SARS-CoV-2-proven respiratory illness in the absence of a COVID-19 specific vaccine, measured over 6 months following randomisation in healthcare.	Estimand 5a.5<br><br>Population: ITT population<br>Outcome: as for estimand 5a.1<br>Interventions: as for estimand 5a.1<br>Handling of Intercurrent events: as for estimand 5a.1<br>Summary Measure: as for estimand 5a.1
To determine if BCG vaccination compared with placebo prolongs the time to first SARS-CoV-2-proven respiratory illness in the absence of a COVID-19 specific vaccine, measured over 12 months following randomisation in healthcare.	Estimand 5b.5<br><br>Population: ITT population<br>Outcome: as for estimand 5b.1<br>Interventions: as for estimand 5b.1<br>Handling of Intercurrent events: as for estimand 5b.1<br>Summary Measure: as for estimand 5b.1
To determine if BCG vaccination compared with placebo prolongs the time to first SARS-CoV-2-proven respiratory illness irrespective of receiving a COVID-19 specific vaccine or any other vaccine, measured over 6 months following randomisation in healthcare.	Estimand 5a.6<br><br>Population: ITT population<br>Outcome: as for estimand 5a.1<br>Interventions: as for estimand 5a.1<br>Handling of Intercurrent events: as for estimand 5a.2<br>Summary Measure: as for estimand 5a.1
To determine if BCG vaccination compared with placebo prolongs the time to first SARS-CoV-2-proven respiratory illness rrespective of receiving a COVID-19 specific vaccine or any other vaccine, measured over 12 months following r39andomization in healthcare.	Estimand 5b.6<br><br>Population: ITT population<br>Outcome: as for estimand 5b.1<br>Interventions: as for estimand 5b.1<br>Handling of Intercurrent events: as for estimand 5b.2<br>Summary Measure: as for estimand 5b.1
** 1) adjusted for stratification factors used at randomisation (age group, presence of comorbidity, and geographical location); 2) adjusted for stratification factors used at randomisation + sex, BMI at baseline, BCG vaccine before randomisation;*	

7.2.1. ANALYSIS

The time to COVID-19 (either symptomatic or severe COVID-19) will be calculated and presented in the two intervention groups.
Survival curves for the time to COVID-19 episode will be constructed for each intervention group using the Kaplan-Meier product limit method.

Adjusted analyses will be used to compare the time to event distributions between groups by means of Cox's proportional hazards model. Initially the model will be adjusted for the stratification factors used during randomisation (age group, presence of comorbidity, geographical location -Europe/Australia/South America). Additional analyses will be conducted using the same Cox's proportional hazards but also adjusted for the following baseline covariates (as described in section 6.2.2): sex, BMI, BCG prior to study enrolment. The proportional hazards assumption will be checked when running these analyses. Assuming the proportional hazards assumption is found to be reasonable, results from the proportional hazards regression will be presented as the hazard ratios and their corresponding 95% confidence intervals.

7.2.2. SUBGROUP ANALYSIS

The following subgroup analyses (described in details in section 6.4) will be conducted for estimands 5a.1 and 5b.1:

- Sub-Group analysis 1 – Age group (stratification factor at randomisation)
- Sub-Group analyses 2, 2a, 2b, 2c, 2d, 2e – Presence of comorbidities
- Sub-Group analysis 3 – Geographical Location
- Sub-Group analysis 4 – Sex
- Sub-Group analysis 5 – BCG in the past or not
- Sub-Group analysis 6 – serology results to SARS-CoV-2 at enrolment (ITT pop only, estimands 5a.5 and 5b.5)

7.3. NUMBER OF EPISODES OF COVID-19 (#6a and #6b)

7.3.1. ESTIMANDS

Objective	Estimand
To determine if BCG vaccination compared with placebo reduces the number of COVID-19 episodes in the absence of a COVID-19 specific vaccine, measured over 6 months following randomisation in healthcare workers who did not have a previous SARS-CoV-2 positive test result when assessed at time of randomisation.	Estimand 6a.1<br><br>Population: mITT population<br>Outcome: number of COVID-19 episodes by 6 months<br>Interventions: BCG vs Placebo<br>Handling of Intercurrent events:<br>- COVID-19 specific vaccine (Hypothetical Strategy)<br>- any other vaccine (Treatment Policy strategy)<br>Summary Measure: Adjusted* difference in the expected counts
To determine if BCG vaccination compared with placebo reduces the number of COVID-19 episodes in the absence of a COVID-19 specific vaccine, measured over 12 months following randomisation in healthcare workers who did not have a previous SARS-CoV-2 positive test result when assessed at time of randomisation.	Estimand 6b.1<br><br>Population: mITT population<br>Outcome: number of COVID-19 episodes by 12 months<br>Interventions: BCG vs Placebo<br>Handling of Intercurrent events:<br>- COVID-19 specific vaccine (Hypothetical Strategy)<br>- any other vaccine (Treatment Policy strategy)<br>Summary Measure: Adjusted* difference in the expected counts
To determine if BCG vaccination compared with placebo reduces the number of COVID-19 episodes irrespective of receiving a COVID-19 specific vaccine or any other vaccine, measured over 6 months following randomisation in healthcare workers who did not have a previous SARS-CoV-2 positive test result when assessed at time of randomisation.	Estimand 6a.2<br><br>Population: as 6a.1<br>Outcome: as 6a.1<br>Interventions: as 6a.1<br>Handling of Intercurrent events:<br>- COVID-19 specific vaccine (Treatment Policy strategy)<br>- any other vaccine (Treatment Policy strategy)<br>Summary Measure: as 6a.1
To determine if BCG vaccination compared with placebo reduces the number of COVID-19 episodes irrespective of receiving a COVID-19 specific vaccine or any other vaccine, measured over 12 months following randomisation in healthcare workers who did not have a previous SARS-CoV-2 positive test result when assessed at time of randomisation.	Estimand 6b.2<br><br>Population: as 6b.1<br>Outcome: as 6b.1<br>Interventions: as 6b.1<br>Handling of Intercurrent events:<br>- COVID-19 specific vaccine (Treatment Policy strategy)<br>- any other vaccine (Treatment Policy strategy)<br>Summary Measure: as 6b.1
To determine if BCG vaccination compared with placebo reduces the number of COVID-19 episodes illness in the absence of any vaccine, measured over 6 months following randomisation in healthcare	Estimand 6a.3<br><br>Population: as 6a.1<br>Outcome: as 6a.1

Objective	Estimand
workers who did not have a previous SARS-CoV-2 positive test result when assessed at time of randomisation.	Interventions: as 6a.1<br>Handling of Intercurrent events:<br>- COVID-19 specific vaccine (Hypothetical Strategy)<br>- any other vaccine (Hypothetical Strategy)<br>Summary Measure: as 6a.1
To determine if BCG vaccination compared with placebo reduces the number of COVID-19 episodes in the absence of any vaccine, measured over 12 months following randomisation in healthcare workers who did not have a previous SARS-CoV-2 positive test result when assessed at time of randomisation.	Estimand 6b.3<br><br>Population: as 6b.1<br>Outcome: as 6b.1<br>Interventions: as 6b.1<br>Handling of Intercurrent events:<br>- COVID-19 specific vaccine (Hypothetical Strategy)<br>- any other vaccine (Hypothetical Strategy)<br>Summary Measure: as 6b.1
** 1) adjusted for stratification factors used at randomisation (age group, presence of comorbidity, and geographical location)*	

7.3.2. ANALYSIS

The median and IQR for the number of episodes will be presented by intervention group.
The difference between BCG and placebo groups will be summarised as difference in the logs of expected number of episodes and its 95%CI estimated using a Zero-Inflated Negative Binomial (ZINB) model. Since this secondary outcome will be analysed mainly for descriptive purposes, the model will only be adjusted for the stratification factors used at randomisation (Geographical Location (Australia/Europe/South America, age group and presence of comorbidity).
The analysis will be done using the *zinb* command in Stata, with the *inflate()* and *exposure()* options. The *inflate()* option will be used to indicate whether the participant had the COVID-19 event, while the *exposure()* option will be used to indicate the amount of exposure over which the number of episodes of COVID-19 were observed for each participant (refer to section 6.2.3).

7.3.3. ADDITIONAL ANALYSIS

Additionally, the median and IQR of the number of episodes will be calculated and presented by intervention group in the subgroups of participants who:

- had COVID-19 (either symptomatic or severe)
- had symptomatic COVID-19
- had severe COVID-19

7.3.4. SUBGROUP ANALYSIS

None

7.4. ASYMPTOMATIC SARS-COV-2 INFECTION (#7)

7.4.1. ESTIMANDS

Objective	Estimand
To determine if BCG vaccination compared with placebo reduces the incidence of asymptomatic COVID-19 irrespective of receiving a non-CoronaVac COVID-19 specific vaccine or any other vaccine, over the 6 months following randomisation, in healthcare workers who did not receive CoronaVac during the study or have a previous SARS-CoV-2 positive test result when assessed at time of randomisation.	Estimand 7a.1<br><br>Population: mITT population<br>Outcome: asymptomatic SARS-COV-2 infection by 6 months<br>Interventions: BCG vs Placebo<br>Handling of Intercurrent events:<br>- non-CoronaVac COVID-19 specific vaccine (Treatment Policy strategy)<br>- CoronaVac vaccine (Principal Stratum strategy)<br>- any other other vaccine (Treatment Policy strategy)<br>Summary Measure: Adjusted* difference in proportion of participants
To determine if BCG vaccination compared with placebo reduces the incidence of asymptomatic COVID-19 irrespective of receiving a non-CoronaVac COVID-19 specific vaccine or any other vaccine, over the 12 months following randomisation, in healthcare workers who did not receive CoronaVac during the study or have a previous SARS-CoV-2 positive test result when assessed at time of randomisation.	Estimand 7b.1<br><br>Population: mITT population<br>Outcome: asymptomatic SARS-COV-2 infection by 12 months<br>Interventions: BCG vs Placebo<br>Handling of Intercurrent events:<br>- non-CoronaVac COVID-19 specific vaccine (Treatment Policy strategy)<br>- CoronaVac vaccine (Principal Stratum strategy)<br>- any other other vaccine (Treatment Policy strategy)<br>Summary Measure: Adjusted* difference in proportion of participants
** 1) adjusted for stratification factors used at randomisation (age group, presence of comorbidity, and geographical location); 2) adjusted for stratification factors used at randomisation + sex, BMI at baseline, BCG vaccine before randomisation*	

7.4.2. ANALYSIS

The outcome of asymptomatic COVID-19, determined by seroconversion at 3 or 6 months not associated with any episode of illness will be described by intervention group as the absolute number of participants with the outcome.

The treatment effect for this outcome will be the difference in proportion (BCG - Control) estimated using a binomial regression model, adjusted for stratification factors used at randomisation (age group, presence of comorbidity, and geographical location). An additional analysis will be conducted using the same binomial regression model but also adjusted for the following baseline covariates (as described in section 6.2.2): sex, BMI, and BCG prior to study enrolment. Should the binomial regression model have convergence difficulties (due to low prevalence outcome), generalised liner model (GLM) approach with Gaussian error distribution and identity link function will be adopted.
Since the administration of CoronaVac (which is a post randomisation intercurrent event) more than 7 days prior to the blood collection makes the results of serology indeterminant, CoronaVac will be handled the Principal Stratum strategy i.e. estimating the treatment effect in participants who would not have received CoronaVac in either treatment arm. For the main analysis we will assume that the occurrence of this intercurrent event is not related to the study intervention (BCG/Placebo), and we will restrict the analysis to participants who did not receive CoronaVac (all participants who received CoronaVac will be excluded from the analysis).
As a sensitivity analysis, we will repeat the analysis under the assumption of conditional independence of the treatment and the intercurrent event (known as principal ignorability). For this analysis, separate models will be specified for the outcome and the intercurrent event itself, assuming that conditional on baseline covariates the outcome in the control group and the occurrence of the intercurrent event in the intervention group are independent. In order to conduct this analysis, we will first model the probability of the occurrence of the intercurrent event on the intervention group as well study site, sex, presence of comorbidities, and age (variables that could potentially confound the outcome and the intercurrent event),

using logistic regression. Then, we will use the predicted probabilities as weights for participants on the control group in the analysis model for the outcome.

As a second, and very conservative, sensitivity analysis, participants in study sites where CoronaVac was available (Mato Grosso do Sul, Rio de Janeiro, and Amazonas -BRA), will be excluded from the analysis of this outcome so that this intercurrent event is no longer relevant.

Participants with unavailable/indeterminant serology data, and participants on whom the absence of episodes of illness over 6 months cannot be ascertained (due to incomplete data entry) will be coded as missing the outcome.

7.4.3. ADDITIONAL ANALYSIS

Since a SARS-CoV-2 infection could also be detected by a positive PCR/RAT not associated with a severe episode of illness or an episode with "trigger" symptoms" (e.g., SARS-CoV-2 infection detected via a screening test), and since seroconversion could also be explained by not severe SARS-CoV-2 infection not characterised by trigger symptoms, we will calculate and report by intervention group the number of participants who:

- Show evidence of COVID-19 determined by a positive PCR or positive RAT (and not serology) not related any episode of illness OR
- Show evidence of COVID-19 determined by a positive PCR or positive RAT (and not serology) related to a non-trigger non-severe episode of illness
- Show evidence of COVID-19 determined by seroconversion at 3 or 6 months associated with an episode of illness which is not trigger AND not severe (as defined in section 4.1)

Moreover, in those participants who meet the asymptomatic outcome the following information will be described and reported by intervention group as mean, standard deviation (or median and IQR if distribution is skewed) or absolute and relative frequencies, according to the nature of the variable:

- Sex – Male/Female/Other/Declined/Missing
- Age, years
- Body Mass Index (BMI), kg/m^2 – < 18.5 / 18.5 to 24.9/ 25 to 29.9 / >30 / Missing
- Role – Administrative-clerical staff / Allied Health / Dentist-dental therapy / Doctor / Nurse-Midwife / Patient Services Assistant-hospital maintenance / Scientist (medical research) / Other / Missing
- Contact with patients, hours - <10 / 10-20 / >20 / Missing
- Confirmed cases of COVID-19 within department – Yes / No / Missing
- Smoking – Yes / No / Missing
- Previous BCG vaccination -No / last BCG dose <1 year ago / 1-5 years ago / >5 years ago / Missing

7.4.4. SUBGROUP ANALYSIS

The following subgroup analyses (described in detail in section 6.4) will be conducted for estimands 7a.1 and 7b.1:

- Sub-Group analysis 1 – Age group (stratification factor at randomisation)
- Sub-Group analyses 2, 2a, 2b, 2c, 2d, 2e – Presence of comorbidities
- Sub-Group analysis 3 – Geographical Location
- Sub-Group analysis 4 – Sex
- Sub-Group analysis 5 – BCG in the past or not

7.5. NUMBER OF DAYS UNABLE TO WORK DUE TO COVID-19 (#8a and #8b)

7.5.1. ESTIMANDS

Objective	Estimand
To determine if BCG vaccination compared with placebo reduces the number of days unable to work due to COVID-19 in the absence of a COVID-19 specific vaccine, measured over 6 months following randomisation in healthcare workers who did not have a previous SARS-CoV-2 positive test result when assessed at time of randomisation.	Estimand 8a.1<br><br><u>Population</u>: mITT population<br><u>Outcome</u>: number of days unable to work due to COVID-19 by 6 months<br><u>Interventions</u>: BCG vs Placebo<br><u>Handling of Intercurrent events</u>:<br>- COVID-19 specific vaccine (Hypothetical Strategy)<br>- any other vaccine (Treatment Policy strategy)<br><u>Summary Measure</u>: adjusted* difference in the expected counts
To determine if BCG vaccination compared with placebo reduces the number of days unable to work due to COVID-19 in the absence of a COVID-19 specific vaccine, measured over 12 months following randomisation in healthcare workers who did not have a previous SARS-CoV-2 positive test result when assessed at time of randomisation.	Estimand 8b.1<br><br><u>Population</u>: mITT population<br><u>Outcome</u>: number of days unable to work due to COVID-19 by 12 months<br><u>Interventions</u>: BCG vs Placebo<br><u>Handling of Intercurrent events</u>:<br>- COVID-19 specific vaccine (Hypothetical Strategy)<br>- any other vaccine (Treatment Policy strategy)<br><u>Summary Measure</u>: adjusted* difference in the expected counts
To determine if BCG vaccination compared with placebo reduces the number of days unable to work due to COVID-19 irrespective of receiving a COVID-19 specific vaccine or any other vaccine, measured over 6 months following randomisation in healthcare workers who did not have a previous SARS-CoV-2 positive test result when assessed at time of randomisation.	Estimand 8a.2<br><br><u>Population</u>: as 8a.1<br><u>Outcome</u>: as 8a.1<br><u>Interventions</u>: as 8a.1<br><u>Handling of Intercurrent events</u>:<br>- COVID-19 specific vaccine (Treatment Policy strategy)<br>- any other vaccine (Treatment Policy strategy)<br><u>Summary Measure</u>: as 8a.1
To determine if BCG vaccination compared with placebo reduces the number of days unable to work due to COVID-19 irrespective of receiving a COVID-19 specific vaccine or any other vaccine, measured over 12 months following randomisation in healthcare workers who did not have a previous SARS-CoV-2 positive test result when assessed at time of randomisation.	Estimand 8b.2<br><br><u>Population</u>: as 8b.1<br><u>Outcome</u>: as 8b.1<br><u>Interventions</u>: as 8b.1<br><u>Handling of Intercurrent events</u>:<br>- COVID-19 specific vaccine (Treatment Policy strategy)<br>- any other vaccine (Treatment Policy strategy)<br><u>Summary Measure</u>: as 8b.1
To determine if BCG vaccination compared with placebo reduces the number of days unable to work due to COVID-19 illness in the absence of any vaccine, measured over 6 months following randomisation in healthcare workers who did not have a previous SARS-CoV-2 positive test result when assessed at time of randomisation.	Estimand 8a.3<br><br><u>Population</u>: as 8a.1<br><u>Outcome</u>: as 8a.1<br><u>Interventions</u>: as 8a.1<br><u>Handling of Intercurrent events</u>:<br>- COVID-19 specific vaccine (Hypothetical Strategy)<br>- any other vaccine (Hypothetical Strategy)<br><u>Summary Measure</u>: as 8a.1
To determine if BCG vaccination compared with placebo reduces the number of days unable to work due to COVID-19 in the absence of any vaccine, measured over 12 months following randomisation	Estimand 8b.3<br><br><u>Population</u>: as 8b.1<br><u>Outcome</u>: as 8b.1

Objective	Estimand
in healthcare workers who did not have a previous SARS-CoV-2 positive test result when assessed at time of randomisation.	Interventions: as 8b.1<br>Handling of Intercurrent events:<br>- COVID-19 specific vaccine (Hypothetical Strategy)<br>- any other vaccine (Hypothetical Strategy)<br>Summary Measure: as 8b.1
** 1) adjusted for stratification factors used at randomisation (age group, presence of comorbidity, and geographical location)*	

7.5.2. ANALYSIS

As per section 7.3.2.

7.5.3. ADDITIONAL ANALYSIS

As per section 7.3.3.

7.5.4. SUBGROUP ANALYSIS

None.

7.6. NUMBER OF DAYS CONFINED TO BED DUE TO COVID-19 (#9a and #9b)

7.6.1. ESTIMANDS

Objective	Estimand
To determine if BCG vaccination compared with placebo reduces the number of days confined to bed due to COVID-19 in the absence of a COVID-19 specific vaccine, measured over 6 months following randomisation in healthcare workers who did not have a previous SARS-CoV-2 positive test result when assessed at time of randomisation.	Estimand 9a.1<br><br>Population: mITT population<br>Outcome: number of days confined to bed due to COVID-19 by 6 months<br>Interventions: BCG vs Placebo<br>Handling of Intercurrent events:<br>- COVID-19 specific vaccine (Hypothetical Strategy)<br>- any other vaccine (Treatment Policy strategy)<br>Summary Measure: adjusted* difference in the expected counts
To determine if BCG vaccination compared with placebo reduces the number of days confined to bed due to COVID-19 in the absence of a COVID-19 specific vaccine, measured over 12 months following randomisation in healthcare workers who did not have a previous SARS-CoV-2 positive test result when assessed at time of randomisation.	Estimand 9b.1<br><br>Population: mITT population<br>Outcome: number of days confined to bed due to COVID-19 by 12 months<br>Interventions: BCG vs Placebo<br>Handling of Intercurrent events:<br>- COVID-19 specific vaccine (Hypothetical Strategy)<br>- any other vaccine (Treatment Policy strategy)<br>Summary Measure: adjusted* difference in the expected counts
To determine if BCG vaccination compared with placebo reduces the number of days confined to bed due to COVID-19 irrespective of receiving a COVID-19 specific vaccine or any other vaccine, measured over 6 months following randomisation in healthcare workers who did not have a previous SARS-CoV-2 positive test result when assessed at time of randomisation.	Estimand 9a.2<br><br>Population: as 9a.1<br>Outcome: as 9a.1<br>Interventions: as 9a.1<br>Handling of Intercurrent events:<br>- COVID-19 specific vaccine (Treatment Policy strategy)<br>- any other vaccine (Treatment Policy strategy)<br>Summary Measure: as 9a.1
To determine if BCG vaccination compared with placebo reduces the number of days confined to bed due to COVID-19 irrespective of receiving a COVID-19 specific vaccine or any other vaccine,	Estimand 9b.2<br><br>Population: as 9b.1<br>Outcome: as 9b.1

Objective	Estimand
measured over 12 months following randomisation in healthcare workers who did not have a previous SARS-CoV-2 positive test result when assessed at time of randomisation.	Interventions: as 9b.1<br>Handling of Intercurrent events:<br>- COVID-19 specific vaccine (Treatment Policy strategy)<br>- any other vaccine (Treatment Policy strategy)<br>Summary Measure: as 9b.1
To determine if BCG vaccination compared with placebo reduces the number of days confined to bed due to COVID-19 illness in the absence of any vaccine, measured over 6 months following randomisation in healthcare workers who did not have a previous SARS-CoV-2 positive test result when assessed at time of randomisation.	Estimand 9a.3<br><br>Population: as 9a.1<br>Outcome: as 9a.1<br>Interventions: as 9a.1<br>Handling of Intercurrent events:<br>- COVID-19 specific vaccine (Hypothetical Strategy)<br>- any other vaccine (Hypothetical Strategy)<br>Summary Measure: as 9a.1
To determine if BCG vaccination compared with placebo reduces the number of days confined to bed due to COVID-19 in the absence of any vaccine, measured over 12 months following randomisation in healthcare workers who did not have a previous SARS-CoV-2 positive test result when assessed at time of randomisation.	Estimand 9b.3<br><br>Population: as 9b.1<br>Outcome: as 9b.1<br>Interventions: as 9b.1<br>Handling of Intercurrent events:<br>- COVID-19 specific vaccine (Hypothetical Strategy)<br>- any other vaccine (Hypothetical Strategy)<br>Summary Measure: as 9b.1
** 1) adjusted for stratification factors used at randomisation (age group, presence of comorbidity, and geographical location)*	

7.6.1. ANALYSIS

As per section 7.3.2.

7.6.2. ADDITIONAL ANALYSIS

As per section 7.3.3.

7.6.3. SUBGROUP ANALYSIS

None.

7.7. NUMBER OF DAYS WITH SYMPTOMS DUE TO COVID-19 (#10a and #10b)

7.7.1. ESTIMANDS

Objective	Estimand
To determine if BCG vaccination compared with placebo reduces the number of days with symptoms due to COVID-19 in the absence of a COVID-19 specific vaccine, measured over 6 months following randomisation in healthcare workers who did not have a previous SARS-CoV-2 positive test result when assessed at time of randomisation.	Estimand 10a.1<br><br>Population: mITT population<br>Outcome: number of days with symptoms due to COVID-19 by 6 months<br>Interventions: BCG vs Placebo<br>Handling of Intercurrent events:<br>- COVID-19 specific vaccine (Hypothetical Strategy)<br>- any other vaccine (Treatment Policy strategy)<br>Summary Measure: adjusted* difference in the expected counts

Objective	Estimand
To determine if BCG vaccination compared with placebo reduces the number of days with symptoms due to COVID-19 in the absence of a COVID-19 specific vaccine, measured over 12 months following randomisation in healthcare workers who did not have a previous SARS-CoV-2 positive test result when assessed at time of randomisation.	Estimand 10b.1<br><br>Population: mITT population<br>Outcome: number of days with symptoms due to COVID-19 by 12 months<br>Interventions: BCG vs Placebo<br>Handling of Intercurrent events:<br>- COVID-19 specific vaccine (Hypothetical Strategy)<br>- any other vaccine (Treatment Policy strategy)<br>Summary Measure: adjusted difference in the expected counts
To determine if BCG vaccination compared with placebo reduces the number of days with symptoms due to COVID-19 irrespective of receiving a COVID-19 specific vaccine or any other vaccine, measured over 6 months following randomisation in healthcare workers who did not have a previous SARS-CoV-2 positive test result when assessed at time of randomisation.	Estimand 10a.2<br><br>Population: as 10a.1<br>Outcome: as 10a.1<br>Interventions: as 10a.1<br>Handling of Intercurrent events:<br>- COVID-19 specific vaccine (Treatment Policy strategy)<br>- any other vaccine (Treatment Policy strategy)<br>Summary Measure: as 10a.1
To determine if BCG vaccination compared with placebo reduces the number of days with symptoms due to COVID-19 irrespective of receiving a COVID-19 specific vaccine or any other vaccine, measured over 12 months following randomisation in healthcare workers who did not have a previous SARS-CoV-2 positive test result when assessed at time of randomisation.	Estimand 10b.2<br><br>Population: as 10b.1<br>Outcome: as 10b.1<br>Interventions: as 10b.1<br>Handling of Intercurrent events:<br>- COVID-19 specific vaccine (Treatment Policy strategy)<br>- any other vaccine (Treatment Policy strategy)<br>Summary Measure: as 10b.1
To determine if BCG vaccination compared with placebo reduces the number of days with symptoms due to COVID-19 illness in the absence of any vaccine, measured over 6 months following randomisation in healthcare workers who did not have a previous SARS-CoV-2 positive test result when assessed at time of randomisation.	Estimand 10a.3<br><br>Population: as 10a.1<br>Outcome: as 10a.1<br>Interventions: as 10a.1<br>Handling of Intercurrent events:<br>- COVID-19 specific vaccine (Hypothetical Strategy)<br>- any other vaccine (Hypothetical Strategy)<br>Summary Measure: as 10a.1
To determine if BCG vaccination compared with placebo reduces the number of days with symptoms due to COVID-19 in the absence of any vaccine, measured over 12 months following randomisation in healthcare workers who did not have a previous SARS-CoV-2 positive test result when assessed at time of randomisation.	Estimand 10b.3<br><br>Population: as 10b.1<br>Outcome: as 10b.1<br>Interventions: as 10b.1<br>Handling of Intercurrent events:<br>- COVID-19 specific vaccine (Hypothetical Strategy)<br>- any other vaccine (Hypothetical Strategy)<br>Summary Measure: as 10b.1
** 1) adjusted for stratification factors used at randomisation (age group, presence of comorbidity, and geographical location)*	

7.7.2. ANALYSIS

As per section 7.3.2.

7.7.3. ADDITIONAL ANALYSIS

As per section 7.3.3.

7.7.4. SUBGROUP ANALYSIS

None.

7.8. PNEUMONIA DUE TO COVID-19 (#11a and #11b)

7.8.1. ESTIMANDS

Objective	Estimand
To determine if BCG vaccination compared with placebo reduces the incidence of pneumonia due to COVID-19 in the absence of a COVID vaccine, measured over 6 months following randomisation in healthcare workers who did not have a previous SARS-CoV-2 positive test result when assessed at time of randomisation.	Estimand 11a.1<br><br>Population: mITT population<br>Outcome: pneumonia due to COVID-19 by 6 months<br>Interventions: BCG vs Placebo<br>Handling of Intercurrent events:<br>- COVID-19 specific vaccine (Hypothetical Strategy)<br>- any other vaccine (Treatment Policy strategy)<br>Summary Measure: Adjusted* difference in proportion of participants
To determine if BCG vaccination compared with placebo reduces the incidence of pneumonia due to COVID-19 in the absence of a COVID vaccine, measured over 12 months following randomisation in healthcare workers who did not have a previous SARS-CoV-2 positive test result when assessed at time of randomisation.	Estimand 11b.1<br><br>Population: mITT population<br>Outcome: pneumonia due to COVID-19 by 6 months by 12 months<br>Interventions: BCG vs Placebo<br>Handling of Intercurrent events:<br>- COVID-19 specific vaccine (Hypothetical Strategy)<br>- any other vaccine (Treatment Policy strategy)<br>Summary Measure: Adjusted* difference in proportion of participants
To determine if BCG vaccination compared with placebo reduces the incidence of pneumonia due to COVID-19 irrespective of receiving a COVID-19 specific vaccine or any other vaccine, measured over 6 months following randomisation in healthcare workers who did not have a previous SARS-CoV-2 positive test result when assessed at time of randomisation.	Estimand 11a.2<br><br>Population: as 11a.1<br>Outcome: as 11a.1<br>Interventions: as 11a.1<br>Handling of Intercurrent events:<br>- COVID-19 specific vaccine (Treatment Policy strategy)<br>- any other vaccine (Treatment Policy strategy)<br>Summary Measure: as 11a.1
To determine if BCG vaccination compared with placebo reduces the incidence of pneumonia due to COVID-19 irrespective of receiving a COVID-19 specific vaccine or any other vaccine, measured over 12 months following randomisation in healthcare workers who did not have a previous SARS-CoV-2 positive test result when assessed at time of randomisation.	Estimand 11b.2<br><br>Population: as 11b.1<br>Outcome: as 11b.1<br>Interventions: as 11b.1<br>Handling of Intercurrent events:<br>- COVID-19 specific vaccine (Treatment Policy strategy)<br>- any other vaccine (Treatment Policy strategy)<br>Summary Measure: as 11b.1
To determine if BCG vaccination compared with placebo reduces the incidence of pneumonia due to COVID-19 illness in the absence of any vaccine, measured over 6 months following randomisation in healthcare workers who did not have a previous SARS-CoV-2 positive test result when assessed at time of randomisation.	Estimand 11a.3<br><br>Population: as 11a.1<br>Outcome: as 11a.1<br>Interventions: as 11a.1<br>Handling of Intercurrent events:<br>- COVID-19 specific vaccine (Hypothetical Strategy)<br>- any other vaccine (Hypothetical Strategy)<br>Summary Measure: as 11a.1

Objective	Estimand
To determine if BCG vaccination compared with placebo reduces the incidence of pneumonia due to COVID-19 in the absence of any vaccine, measured over 12 months following randomisation in healthcare workers who did not have a previous SARS-CoV-2 positive test result when assessed at time of randomisation.	Estimand 11b.3<br><br>Population: as 11b.1<br>Outcome: as 11b.1<br>Interventions: as 11b.1<br>Handling of Intercurrent events:<br>- COVID-19 specific vaccine (Hypothetical Strategy)<br>- any other vaccine (Hypothetical Strategy)<br>Summary Measure: as 11b.1
** 1) adjusted for stratification factors used at randomisation (age group, presence of comorbidity, and geographical location)*	

7.8.2. ANALYSIS

Absolute and relative frequencies of pneumonia prior to 6/12 months will be presented by intervention group. The outcome will be compared between the BCG group and the placebo group using a difference in proportions estimated using the same time-to-event analysis approach adopted for primary outcomes 1 and 2 (flexible parametric survival model), adjusted by the stratification factors used at randomisation. A two-sided bias-corrected 95% CI for the difference in proportion (BCG – Control) and a bootstrap p-value will be calculated with bootstrap standard errors. A Kaplan-Meier survival curve will also be presented by treatment arm for descriptive purposes.
For participants that meet the outcome the date of the first day with symptoms associated to a symptomatic/severe COVID-19 event will be taken as the outcome. Participants who did not have COVID-19 within the first 6/12 months on the study will be censored according to the same rules used for the censoring of primary outcomes 1 and 2 (section 6.2.3).

Should the overall number of pneumonia events be so small that the analysis model described has computational difficulties, the outcome will be analysed using the Cox's proportional hazards model, adjusted for the stratification factors used during randomisation (age group, presence of comorbidity, geographical location -Europe/Australia/South America), and presented as the hazard ratio rather than the difference in proportion.

The analysis will be adjusted for the stratification factors only if a minimum number of events is observed, namely 1 event per strata per intervention group.

7.8.3. ADDITIONAL ANALYSIS

None.

7.8.4. SUBGROUP ANALYSIS

None.

7.9. NEED OF OXYGEN DUE TO COVID-19 (#12a and #12b)

7.9.1. ESTIMANDS

Objective	Estimand
To determine if BCG vaccination compared with placebo reduces the need for oxygen therapy due to COVID-19 in the absence of a COVID vaccine, measured over 6 months following randomisation in healthcare workers who did not have a previous SARS-CoV-2 positive test result when assessed at time of randomisation.	Estimand 12a.1<br><br>Population: mITT population<br>Outcome: need of oxygen therapy due to COVID-19 by 6 months<br>Interventions: BCG vs Placebo<br>Handling of Intercurrent events:<br>- COVID-19 specific vaccine (Hypothetical Strategy)<br>- any other vaccine (Treatment Policy strategy)<br>Summary Measure: Adjusted* difference in proportion of participants

Objective	Estimand
To determine if BCG vaccination compared with placebo reduces the need for oxygen therapy due to COVID-19 in the absence of a COVID vaccine, measured over 12 months following randomisation in healthcare workers who did not have a previous SARS-CoV-2 positive test result when assessed at time of randomisation.	Estimand 12b.1<br><br>Population: mITT population<br>Outcome: need of oxygen therapy due to COVID-19 by 12 months<br>Interventions: BCG vs Placebo<br>Handling of Intercurrent events:<br>- COVID-19 specific vaccine (Hypothetical Strategy)<br>- any other vaccine (Treatment Policy strategy)<br>Summary Measure: Adjusted* difference in proportion of participants
To determine if BCG vaccination compared with placebo reduces the need for oxygen therapy due to COVID-19 irrespective of receiving a COVID-19 specific vaccine or any other vaccine, measured over 6 months following randomisation in healthcare workers who did not have a previous SARS-CoV-2 positive test result when assessed at time of randomisation.	Estimand 12a.2<br><br>Population: as 12a.1<br>Outcome: as 12a.1<br>Interventions: as 12a.1<br>Handling of Intercurrent events:<br>- COVID-19 specific vaccine (Treatment Policy strategy)<br>- any other vaccine (Treatment Policy strategy)<br>Summary Measure: as 12a.1
To determine if BCG vaccination compared with placebo reduces the need for oxygen therapy due to COVID-19 irrespective of receiving a COVID-19 specific vaccine or any other vaccine, measured over 12 months following randomisation in healthcare workers who did not have a previous SARS-CoV-2 positive test result when assessed at time of randomisation.	Estimand 12b.2<br><br>Population: as 12b.1<br>Outcome: as 12b.1<br>Interventions: as 12b.1<br>Handling of Intercurrent events:<br>- COVID-19 specific vaccine (Treatment Policy strategy)<br>- any other vaccine (Treatment Policy strategy)<br>Summary Measure: as 12b.1
To determine if BCG vaccination compared with placebo reduces the need for oxygen therapy due to COVID-19 illness in the absence of any vaccine, measured over 6 months following randomisation in healthcare workers who did not have a previous SARS-CoV-2 positive test result when assessed at time of randomisation.	Estimand 12a.3<br><br>Population: as 12a.1<br>Outcome: as 12a.1<br>Interventions: as 12a.1<br>Handling of Intercurrent events:<br>- COVID-19 specific vaccine (Hypothetical Strategy)<br>- any other vaccine (Hypothetical Strategy)<br>Summary Measure: as 12a.1
To determine if BCG vaccination compared with placebo reduces the need for oxygen therapy due to COVID-19 in the absence of any vaccine, measured over 12 months following randomisation in healthcare workers who did not have a previous SARS-CoV-2 positive test result when assessed at time of randomisation.	Estimand 12b.3<br><br>Population: as 12b.1<br>Outcome: as 12b.1<br>Interventions: as 12b.1<br>Handling of Intercurrent events:<br>- COVID-19 specific vaccine (Hypothetical Strategy)<br>- any other vaccine (Hypothetical Strategy)<br>Summary Measure: as 12b.1
** 1) adjust by stratification factors used at randomisation;*	

7.9.2. ANALYSIS

As per section 7.8.2.

7.9.3.ADDITIONAL ANALYSIS

On the subset of participants who needed oxygen due to severe COVID-19, the mean and standard deviation (or median and IQR if not normally distributed) of the duration of oxygen therapy will be calculated and presented by intervention group.

7.9.4.SUBGROUP ANALYSIS

None.

7.10. ADMISSION TO CRITICAL CARE DUE TO COVID-19 (#13a and #13b)

7.10.1. ESTIMANDS

Objective	Estimand
To determine if BCG vaccination compared with placebo reduces admission to critical care DUE TO COVID-19 in the absence of a COVID vaccine, measured over 6 months following randomisation in healthcare workers who did not have a previous SARS-CoV-2 positive test result when assessed at time of randomisation.	Estimand 13a.1<br><br>Population: mITT population<br>Outcome: admission to critical care due to COVID-19 by 6 months<br>Interventions: BCG vs Placebo<br>Handling of Intercurrent events:<br>- COVID-19 specific vaccine (Hypothetical Strategy)<br>- any other vaccine (Treatment Policy strategy)<br>Summary Measure: Adjusted* difference in proportion of participants
To determine if BCG vaccination compared with placebo reduces admission to critical care DUE TO COVID-19 in the absence of a COVID vaccine, measured over 12 months following randomisation in healthcare workers who did not have a previous SARS-CoV-2 positive test result when assessed at time of randomisation.	Estimand 13b.1<br><br>Population: mITT population<br>Outcome: admission to critical care due to COVID-19 by 12 months<br>Interventions: BCG vs Placebo<br>Handling of Intercurrent events:<br>- COVID-19 specific vaccine (Hypothetical Strategy)<br>- any other vaccine (Treatment Policy strategy)<br>Summary Measure: Adjusted* difference in proportion of participants
To determine if BCG vaccination compared with placebo reduces admission to critical care due to COVID-19 irrespective of receiving a COVID-19 specific vaccine or any other vaccine, measured over 6 months following randomisation in healthcare workers who did not have a previous SARS-CoV-2 positive test result when assessed at time of randomisation.	Estimand 13a.2<br><br>Population: as 13a.1<br>Outcome: as 13a.1<br>Interventions: as 13a.1<br>Handling of Intercurrent events:<br>- COVID-19 specific vaccine (Treatment Policy strategy)<br>- any other vaccine (Treatment Policy strategy)<br>Summary Measure: as 13a.1
To determine if BCG vaccination compared with placebo reduces admission to critical care due to COVID-19 irrespective of receiving a COVID-19 specific vaccine or any other vaccine, measured over 12 months following randomisation in healthcare workers who did not have a previous SARS-CoV-2 positive test result when assessed at time of randomisation.	Estimand 13b.2<br><br>Population: as 13b.1<br>Outcome: as 13b.1<br>Interventions: as 13b.1<br>Handling of Intercurrent events:<br>- COVID-19 specific vaccine (Treatment Policy strategy)<br>- any other vaccine (Treatment Policy strategy)<br>Summary Measure: as 13b.1
To determine if BCG vaccination compared with placebo reduces admission to critical care due to COVID-19 illness in the absence of any vaccine, measured over 6 months following randomisation in healthcare workers who did not have a previous SARS-CoV-2 positive test result when assessed at time of randomisation.	Estimand 13a.3<br><br>Population: as 13a.1<br>Outcome: as 13a.1<br>Interventions: as 13a.1<br>Handling of Intercurrent events:<br>- COVID-19 specific vaccine (Hypothetical Strategy)

Objective	Estimand
	- any other vaccine (Hypothetical Strategy)<br>Summary Measure: as 13a.1
To determine if BCG vaccination compared with placebo reduces admission to critical care due to COVID-19 in the absence of any vaccine, measured over 12 months following randomisation in healthcare workers who did not have a previous SARS-CoV-2 positive test result when assessed at time of randomisation.	Estimand 13b.3<br><br>Population: as 13b.1<br>Outcome: as 13b.1<br>Interventions: as 13b.1<br>Handling of Intercurrent events:<br>- COVID-19 specific vaccine (Hypothetical Strategy)<br>- any other vaccine (Hypothetical Strategy)<br>Summary Measure: as 13b.1
** 1) adjust by stratification factors used at randomisation;*	

7.10.2. PRIMARY ANALYSIS

As per section 7.8.2.

7.10.3. ADDITIONAL ANALYSIS

In the those of participants who were admitted to critical care due to severe COVID-19, the mean and standard deviation (or median and IQR if distribution is skewed) of the duration of critical care will be calculated and presented by intervention group.

7.10.4. SUBGROUP ANALYSIS

None.

7.11. NEED OF MECHANICAL VENTILATION DUE TO COVID-19 (#14a and #14b)

7.11.1. ESTIMANDS

Objective	Estimand
To determine if BCG vaccination compared with placebo reduces the need of mechanical ventilation due to COVID-19 in the absence of a COVID vaccine, measured over 6 months following randomisation in healthcare workers who did not have a previous SARS-CoV-2 positive test result when assessed at time of randomisation.	Estimand 14a.1<br><br>Population: mITT population<br>Outcome: need of mechanical ventilation due to COVID-19 by 6 months<br>Interventions: BCG vs Placebo<br>Handling of Intercurrent events:<br>- COVID-19 specific vaccine (Hypothetical Strategy)<br>- any other vaccine (Treatment Policy strategy)<br>Summary Measure: Adjusted* difference in proportion of participants
To determine if BCG vaccination compared with placebo reduces the need of mechanical ventilation DUE TO COVID-19 in the absence of a COVID vaccine, measured over 12 months following randomisation in healthcare workers who did not have a previous SARS-CoV-2 positive test result when assessed at time of randomisation.	Estimand 14b.1<br><br>Population: mITT population<br>Outcome: need of mechanical ventilation due to COVID-19 by 12 months<br>Interventions: BCG vs Placebo<br>Handling of Intercurrent events:<br>- COVID-19 specific vaccine (Hypothetical Strategy)<br>- any other vaccine (Treatment Policy strategy)<br>Summary Measure: Adjusted* difference in proportion of participants
To determine if BCG vaccination compared with placebo reduces the need of mechanical ventilation due to COVID-19 irrespective of receiving a COVID-19 specific vaccine or any other vaccine, measured over 6 months following randomisation in healthcare workers who did not have a previous	Estimand 14a.2<br><br>Population: as 14a.1<br>Outcome: as 14a.1<br>Interventions: as 14a.1<br>Handling of Intercurrent events:<br>- COVID-19 specific vaccine (Treatment Policy strategy)

Objective	Estimand
SARS-CoV-2 positive test result when assessed at time of randomisation.	- any other vaccine (Treatment Policy strategy)<br>Summary Measure: as 14a.1
To determine if BCG vaccination compared with placebo reduces the need of mechanical ventilation due to COVID-19 irrespective of receiving a COVID-19 specific vaccine or any other vaccine, measured over 12 months following randomisation in healthcare workers who did not have a previous SARS-CoV-2 positive test result when assessed at time of randomisation.	Estimand 14b.2<br><br>Population: as 14b.1<br>Outcome: as 14b.1<br>Interventions: as 14b.1<br>Handling of Intercurrent events:<br>- COVID-19 specific vaccine (Treatment Policy strategy)<br>- any other vaccine (Treatment Policy strategy)<br>Summary Measure: as 14b.1
To determine if BCG vaccination compared with placebo reduces the need of mechanical ventilation due to COVID-19 illness in the absence of any vaccine, measured over 6 months following randomisation in healthcare workers who did not have a previous SARS-CoV-2 positive test result when assessed at time of randomisation.	Estimand 14a.3<br><br>Population: as 14a.1<br>Outcome: as 14a.1<br>Interventions: as 14a.1<br>Handling of Intercurrent events:<br>- COVID-19 specific vaccine (Hypothetical Strategy)<br>- any other vaccine (Hypothetical Strategy)<br>Summary Measure: as 14a.1
To determine if BCG vaccination compared with placebo reduces the need of mechanical ventilation due to COVID-19 in the absence of any vaccine, measured over 12 months following randomisation in healthcare workers who did not have a previous SARS-CoV-2 positive test result when assessed at time of randomisation.	Estimand 14b.3<br><br>Population: as 14b.1<br>Outcome: as 14b.1<br>Interventions: as 14b.1<br>Handling of Intercurrent events:<br>- COVID-19 specific vaccine (Hypothetical Strategy)<br>- any other vaccine (Hypothetical Strategy)<br>Summary Measure: as 14b.1
** 1) adjust by stratification factors used at randomisation;*	

7.11.2. ANALYSIS

As per section 7.8.2.

7.11.3. ADDITIONAL ANALYSIS

In participants who needed MV due to severe COVID-19, the mean and standard deviation (or median and IQR if distribution is skewed) of the duration of critical care will be calculated and presented by intervention group.

7.11.4. SUBGROUP ANALYSIS

None.

7.12. HOSPITALISATION DUE TO COVID-19 (#15a and #15b)

7.12.1. ESTIMANDS

Objective	Estimand
To determine if BCG vaccination compared with placebo reduces the incidence of hospitalisation due to COVID-19 in the absence of a COVID vaccine, measured over 6 months following randomisation in	Estimand 15a.1<br><br>Population: mITT population<br>Outcome: hospitalisation due to COVID-19 by 6 months

Objective	Estimand
healthcare workers who did not have a previous SARS-CoV-2 positive test result when assessed at time of randomisation.	Interventions: BCG vs Placebo<br>Handling of Intercurrent events:<br>- COVID-19 specific vaccine (Hypothetical Strategy)<br>- any other vaccine (Treatment Policy strategy)<br>Summary Measure: Adjusted* difference in proportion of participants
To determine if BCG vaccination compared with placebo reduces the incidence of hospitalisation due to COVID-19 in the absence of a COVID vaccine, measured over 12 months following randomisation in healthcare workers who did not have a previous SARS-CoV-2 positive test result when assessed at time of randomisation.	Estimand 15b.1<br><br>Population: mITT population<br>Outcome: hospitalisation due to COVID-19 by 12 months<br>Interventions: BCG vs Placebo<br>Handling of Intercurrent events:<br>- COVID-19 specific vaccine (Hypothetical Strategy)<br>- any other vaccine (Treatment Policy strategy)<br>Summary Measure: Adjusted* difference in proportion of participants
To determine if BCG vaccination compared with placebo reduces the incidence of hospitalisation due to COVID-19irrespective of receiving a COVID-19 specific vaccine or any other vaccine, measured over 6 months following randomisation in healthcare workers who did not have a previous SARS-CoV-2 positive test result when assessed at time of randomisation.	Estimand 15a.2<br><br>Population: as 15a.1<br>Outcome: as 15a.1<br>Interventions: as 15a.1<br>Handling of Intercurrent events:<br>- COVID-19 specific vaccine (Treatment Policy strategy)<br>- any other vaccine (Treatment Policy strategy)<br>Summary Measure: as 15a.1
To determine if BCG vaccination compared with placebo reduces the incidence of hospitalisation due to COVID-19 irrespective of receiving a COVID-19 specific vaccine or any other vaccine, measured over 12 months following randomisation in healthcare workers who did not have a previous SARS-CoV-2 positive test result when assessed at time of randomisation.	Estimand 15b.2<br><br>Population: as 15b.1<br>Outcome: as 15b.1<br>Interventions: as 15b.1<br>Handling of Intercurrent events:<br>- COVID-19 specific vaccine (Treatment Policy strategy)<br>- any other vaccine (Treatment Policy strategy)<br>Summary Measure: as 15b.1
To determine if BCG vaccination compared with placebo reduces the incidence of hospitalisation due to COVID-19 illness in the absence of any vaccine, measured over 6 months following randomisation in healthcare workers who did not have a previous SARS-CoV-2 positive test result when assessed at time of randomisation.	Estimand 15a.3<br><br>Population: as 15a.1<br>Outcome: as 15a.1<br>Interventions: as 15a.1<br>Handling of Intercurrent events:<br>- COVID-19 specific vaccine (Hypothetical Strategy)<br>- any other vaccine (Hypothetical Strategy)<br>Summary Measure: as 15a.1
To determine if BCG vaccination compared with placebo reduces the incidence of hospitalisation due to COVID-19 in the absence of any vaccine, measured over 12 months following randomisation in healthcare workers who did not have a previous SARS-CoV-2 positive test result when assessed at time of randomisation.	Estimand 15b.3<br><br>Population: as 15b.1<br>Outcome: as 15b.1<br>Interventions: as 15b.1<br>Handling of Intercurrent events:<br>- COVID-19 specific vaccine (Hypothetical Strategy)<br>- any other vaccine (Hypothetical Strategy)<br>Summary Measure: as 15b.1
** 1) adjust by stratification factors used at randomisation;*	

7.12.2. ANALYSIS

As per section 7.8.2.

7.12.3. ADDITIONAL ANALYSIS

In participants who were admitted to hospital due to COVID-19, the mean and standard deviation (or median and IQR if not normally distributed) of the hospital stay will be calculated and presented by intervention group.

7.12.4. SUBGROUP ANALYSIS

None.

7.13. DEATH DUE TO COVID-19 (#16a and #16b)

7.13.1. ESTIMANDS

Objective	Estimand
To determine if BCG vaccination compared with placebo reduces the incidence of death due to COVID-19 in the absence of a COVID vaccine, measured over 6 months following randomisation in healthcare workers who did not have a previous SARS-CoV-2 positive test result when assessed at time of randomisation.	Estimand 16a.1<br><br>Population: mITT population<br>Outcome: death due to COVID-19 by 6 months<br>Interventions: BCG vs Placebo<br>Handling of Intercurrent events:<br>- COVID-19 specific vaccine (Hypothetical Strategy)<br>- any other vaccine (Treatment Policy strategy)<br>Summary Measure: Adjusted* difference in proportion of participants
To determine if BCG vaccination compared with placebo reduces the incidence of death due to COVID-19 in the absence of a COVID vaccine, measured over 12 months following randomisation in healthcare workers who did not have a previous SARS-CoV-2 positive test result when assessed at time of randomisation.	Estimand 16b.1<br><br>Population: mITT population<br>Outcome: death due to COVID-19 by 12 months<br>Interventions: BCG vs Placebo<br>Handling of Intercurrent events:<br>- COVID-19 specific vaccine (Hypothetical Strategy)<br>- any other vaccine (Treatment Policy strategy)<br>Summary Measure: Adjusted* difference in proportion of participants
To determine if BCG vaccination compared with placebo reduces the incidence of death due to COVID-19irrespective of receiving a COVID-19 specific vaccine or any other vaccine, measured over 6 months following randomisation in healthcare workers who did not have a previous SARS-CoV-2 positive test result when assessed at time of randomisation.	Estimand 16a.2<br><br>Population: as 16a.1<br>Outcome: as 16a.1<br>Interventions: as 16a.1<br>Handling of Intercurrent events:<br>- COVID-19 specific vaccine (Treatment Policy strategy)<br>- any other vaccine (Treatment Policy strategy)<br>Summary Measure: as 16a.1
To determine if BCG vaccination compared with placebo reduces the incidence of death due to COVID-19 irrespective of receiving a COVID-19 specific vaccine or any other vaccine, measured over 12 months following randomisation in healthcare workers who did not have a previous SARS-CoV-2 positive test result when assessed at time of randomisation.	Estimand 16b.2<br><br>Population: as 16b.1<br>Outcome: as 16b.1<br>Interventions: as 16b.1<br>Handling of Intercurrent events:<br>- COVID-19 specific vaccine (Treatment Policy strategy)<br>- any other vaccine (Treatment Policy strategy)<br>Summary Measure: as 16b.1

Objective	Estimand
To determine if BCG vaccination compared with placebo reduces the incidence of death due to COVID-19 illness in the absence of any vaccine, measured over 6 months following randomisation in healthcare workers who did not have a previous SARS-CoV-2 positive test result when assessed at time of randomisation.	Estimand 16a.3<br><br>Population: as 16a.1<br>Outcome: as 16a.1<br>Interventions: as 16a.1<br>Handling of Intercurrent events:<br>- COVID-19 specific vaccine (Hypothetical Strategy)<br>- any other vaccine (Hypothetical Strategy)<br>Summary Measure: as 16a.1
To determine if BCG vaccination compared with placebo reduces the incidence of death due to COVID-19 in the absence of any vaccine, measured over 12 months following randomisation in healthcare workers who did not have a previous SARS-CoV-2 positive test result when assessed at time of randomisation.	Estimand 16b.3<br><br>Population: as 16b.1<br>Outcome: as 16b.1<br>Interventions: as 16b.1<br>Handling of Intercurrent events:<br>- COVID-19 specific vaccine (Hypothetical Strategy)<br>- any other vaccine (Hypothetical Strategy)<br>Summary Measure: as 16b.1
** 1) adjust by stratification factors used at randomisation;*	

7.13.2. ANALYSIS

Same as per 7.8.2.

7.13.3. ADDITIONAL ANALYSIS

None.

7.13.4. SUBGROUP ANALYSIS

None.

8. NON-COVID19 RELATED SECONDARY OUTCOMES

For these secondary outcomes, these intercurrent events will be handled using a treatment policy strategy, and all outcome data will be used regardless of occurrence of the intercurrent event. In a secondary analysis, the intercurrent events of receiving any vaccine (including a COVID-19-specific vaccine) will be handled using a Hypothetical Strategy; participants who receive any vaccine (including a COVID-19-specific vaccine) will have their data used up to the date of their first dose of vaccine (data collected after the first dose of the vaccine will be ignored).
The analyses of these secondary outcomes will be conducted on the ITT population including only Stage 2 participants. No subgroup analyses for any of these outcomes are planned.

8.1. FEVER OR RESPIRATORY ILLNESS (#17)

8.1.1. ESTIMANDS

Objective	Estimand
To determine if BCG vaccination compared with placebo reduces the incidence of fever or respiratory illness irrespective of receiving any vaccine (including COVID-19 specific vaccine), over the 12 months following randomisation, in healthcare workers exposed to SARS-CoV-2.	Estimand 17.1<br>Population: ITT population<br>Outcome: fever or respiratory illness by 12 months<br>Interventions: BCG vs Placebo<br>Handling of Intercurrent events:<br>- any vaccine, including COVID-19 specific vaccine (Treatment Policy strategy)<br>Summary Measure: Adjusted difference in proportion of participants

Objective	Estimand
To determine if BCG vaccination compared with placebo reduces the incidence of fever or respiratory illness in the absence of any vaccine (including COVID-19 specific vaccine), over the 12 months following randomisation, in healthcare workers exposed to SARS-CoV-2.	Estimand 17.2<br><br>Population: ITT population<br>Outcome: fever or respiratory illness by 12 months<br>Interventions: BCG vs Placebo<br>Handling of Intercurrent events:<br>- any vaccine, including COVID-19 specific vaccine (Hypothetical strategy)<br>Summary Measure: Adjusted difference in proportion of participants

8.1.2. ANALYSIS

The outcome of respiratory or febrile illness prior to 12 months will be described by intervention group as the absolute number of participants with the event.
The numbers of participants whose follow-up data is censored due to:

- incomplete data entry,
- drop-out from the study, and
- intercurrent event (any vaccine, including COVID-19 specific vaccine) (for estimand 17.2 only)

will also be reported separately by intervention group.

Participants with complete data entry (i.e. complete APP weekly data and/or survey data) will be censored:

- (for estimand 17.1) at the 12 months follow-up or 12 months unless the definition of the outcome is met and precedes this date.
- (for estimand 17.2) at the earlier of their first dose of any vaccine given during the 12 months follow-up or 12 months unless the definition of the outcome is met and precedes both of these dates.

Participants with incomplete data entry (i.e. incomplete APP weekly data and incomplete survey data) will be censored at the earlier of:

- their first dose of any vaccine given during the 12 months follow-up (for estimand 17.2 only) or
- their last entered date prior to which there are more than 3 consecutive days of missing data following the last

unless the definition of the outcome is met and precedes both of these dates.

When the definition of the outcome is met, the first day with symptoms for the first episode of fever or respiratory illness will be used in the analysis.

The analysis of this outcome will be conducted as for the primary analysis of the primary outcome 1 and 2 (as presented in section 6.2.2). This outcome will be compared between the BCG group and the placebo group using a difference in proportions. This will be estimated using a time-to-event analysis adjusted for stratification factors used in randomisation, with the survival curve for each combination of strata and randomised group calculated using a flexible parametric survival model (Royston-Parmar model[2]).
A Kaplan-Meier survival curve will also be presented by treatment arm.

8.2. SEVERE FEVER OR RESPIRATORY ILLNESS (#18)

8.2.1. ESTIMANDS

Objective	Estimand
To determine if BCG vaccination compared with placebo reduces the incidence of severe fever or respiratory illness irrespective of receiving any vaccine (including COVID-19 specific vaccine), over	Estimand 18.1<br><br>Population: ITT population<br>Outcome: severe fever or respiratory illness by 12 months<br>Interventions: BCG vs Placebo

Objective	Estimand
the 12 months following randomisation, in healthcare workers exposed to SARS-CoV-2.	Handling of Intercurrent events:<br>- any vaccine, including COVID-19 specific vaccine (Treatment Policy strategy)<br>Summary Measure: Adjusted difference in proportion of participants
To determine if BCG vaccination compared with placebo reduces the incidence of severe fever or respiratory illness in the absence of any vaccine (including COVID-19 specific vaccine), over the 12 months following randomisation, in healthcare workers exposed to SARS-CoV-2.	Estimand 18.2<br><br>Population: ITT population<br>Outcome: severe fever or respiratory illness by 12 months<br>Interventions: BCG vs Placebo<br>Handling of Intercurrent events:<br>- any vaccine, including COVID-19 specific vaccine (Hypothetical strategy)<br>Summary Measure: Adjusted difference in proportion of participants

8.2.2. ANALYSIS

As per section 8.1.2

8.3. NUMBER OF EPISODES OF FEVER OR RESPIRATORY ILLNESS (#19)

8.3.1. ESTIMANDS

Objective	Estimand
To determine if BCG vaccination compared with placebo reduces the number of fever or respiratory illness irrespective of receiving any vaccine (including COVID-19 specific vaccine), over the 12 months following randomisation, in healthcare workers exposed to SARS-CoV-2.	Estimand 19.1<br><br>Population: ITT population<br>Outcome: number of fever or respiratory illness by 12 months<br>Interventions: BCG vs Placebo<br>Handling of Intercurrent events:<br>- any vaccine, including COVID-19 specific vaccine (Treatment Policy strategy)<br>Summary Measure: difference in the expected counts
To determine if BCG vaccination compared with placebo reduces the number of fever or respiratory illness in the absence of any vaccine (including COVID-19 specific vaccine), over the 12 months following randomisation, in healthcare workers exposed to SARS-CoV-2.	Estimand 19.2<br><br>Population: ITT population<br>Outcome: number of fever or respiratory illness by 12 months<br>Interventions: BCG vs Placebo<br>Handling of Intercurrent events:<br>- any vaccine, including COVID-19 specific vaccine (Hypothetical strategy)<br>Summary Measure: difference in the expected counts

8.3.1. ANALYSIS

Median and IQR of the number of episodes will be calculated and presented by intervention group.
The difference between the BCG and Placebo groups in the number of episodes and its 95%CI will be estimated using a Zero-Inflated Negative Binomial (ZINB) model, adjusted by the stratification factors used at randomisation (Geographical Location (Australia/Europe/South America, age group and presence of comorbidity). For further details on the ZINB model refer to section 7.3.2. Particularly the *exposure()* option will be used to indicate the number of days elapsed from the date of randomisation to:

- the date of the 12 months follow-up (for estimand 19.1), or the date of 12 months follow-up or date of first dose of any vaccine, whichever is earlier, (for estimand 19.2)for those participants with complete data entry
- the date of 3 consecutive days of missing data with the participant (for estimand 19.1), or the first date of 3 consecutive days of missing data or date of first dose of any vaccine, whichever is earliest, (for estimand 19.2) for those participants with incomplete data entry

8.4. NUMBER OF DAYS UNABLE TO WORK DUE TO FEVER OR RESPIRATORY ILLNESS (#20)

8.4.1. ESTIMANDS

Objective	Estimand
To determine if BCG vaccination compared with placebo reduces the number of days unable to work due to fever or respiratory illness irrespective of receiving any vaccine (including COVID-19 specific vaccine), over the 12 months following randomisation, in healthcare workers exposed to SARS-CoV-2.	Estimand 20.1<br><br>Population: ITT population<br>Outcome: number days unable to work due to fever or respiratory illness by 12 months<br>Interventions: BCG vs Placebo<br>Handling of Intercurrent events:<br>- any vaccine, including COVID-19 specific vaccine (Treatment Policy strategy)<br>Summary Measure: as for estimand 20.1
To determine if BCG vaccination compared with placebo reduces the number of days unable to work due to fever or respiratory illness in the absence of any vaccine (including COVID-19 specific vaccine), over the 12 months following randomisation, in healthcare workers exposed to SARS-CoV-2.	Estimand 20.1<br><br>Population: ITT population<br>Outcome: number days unable to work due to fever or respiratory illness by 12 months<br>Interventions: BCG vs Placebo<br>Handling of Intercurrent events:<br>- any vaccine, including COVID-19 specific vaccine (Hypothetical strategy)<br>Summary Measure: difference in the expected counts

8.4.2. ANALYSIS

As per section 8.3.1.

8.5. NUMBER OF DAYS CONFINED TO BED DUE TO FEVER OR RESPIRATORY ILLNESS (#21)

8.5.1. ESTIMANDS

Objective	Estimand
To determine if BCG vaccination compared with placebo reduces the number of days confined to bed due to fever or respiratory illness irrespective of receiving any vaccine (including COVID-19 specific vaccine), over the 12 months following randomisation, in healthcare workers exposed to SARS-CoV-2.	Estimand 21.1<br><br>Population: as for estimand 21.1<br>Outcome: as for estimand 21.1<br>Interventions: as for estimand 21.1<br>Handling of Intercurrent events:<br>- any vaccine, including COVID-19 specific vaccine (Treatment Policy strategy)<br>Summary Measure: as for estimand 21.1
To determine if BCG vaccination compared with placebo reduces the number of days confined to bed due to fever or respiratory illness in the absence of any vaccine (including COVID-19 specific vaccine), over the 12 months following randomisation, in healthcare workers exposed to SARS-CoV-2.	Estimand 21.2<br><br>Population: ITT population<br>Outcome: number days confined to bed due fever or respiratory illness by 12 months<br>Interventions: BCG vs Placebo<br>Handling of Intercurrent events:<br>- any vaccine, including COVID-19 specific vaccine (Hypothetical strategy)<br>Summary Measure: difference in the expected counts

8.5.2. ANALYSIS

As per section 8.3.1.

8.6. NUMBER OF DAYS WITH SYMPTOMS DUE TO FEVER OR RESPIRATORY ILLNESS (#22)

8.6.1. ESTIMANDS

Objective	Estimand
To determine if BCG vaccination compared with placebo reduces the number of days with symptoms due to fever or respiratory illness irrespective of receiving any vaccine (including COVID-19 specific vaccine), over the 12 months following randomisation, in healthcare workers exposed to SARS-CoV-2.	Estimand 22.1<br><br>Population: ITT population<br>Outcome: number days with symptoms due fever or respiratory illness by 12 months<br>Interventions: BCG vs Placebo<br>Handling of Intercurrent events:<br>- any vaccine, including COVID-19 specific vaccine (Treatment Policy strategy)<br>Summary Measure: as for estimand 22.1
To determine if BCG vaccination compared with placebo reduces the number of days with symptoms due to fever or respiratory illness in the absence of any vaccine (including COVID-19 specific vaccine), over the 12 months following randomisation, in healthcare workers exposed to SARS-CoV-2.	Estimand 22.2<br><br>Population: ITT population<br>Outcome: number days with symptoms due fever or respiratory illness by 12 months<br>Interventions: BCG vs Placebo<br>Handling of Intercurrent events:<br>- any vaccine, including COVID-19 specific vaccine (Hypothetical strategy)<br>Summary Measure: difference in the expected counts

8.6.2. ANALYSIS

As per section 8.3.1.

8.7. PNEUMONIA (#23)

8.7.1. ESTIMANDS

Objective	Estimand
To determine if BCG vaccination compared with placebo reduces the incidence of pneumonia irrespective of receiving any other vaccine (including COVID-19 specific vaccine), over the 12 months following randomisation, in healthcare workers exposed to SARS-CoV-2.	Estimand 23.1<br><br>Population: ITT population<br>Outcome: pneumonia by 12 months<br>Interventions: BCG vs Placebo<br>Handling of Intercurrent events:<br>- any vaccine, including COVID-19 specific vaccine (Treatment policy)<br>Summary Measure: as for estimand 23.1
To determine if BCG vaccination compared with placebo reduces the incidence of pneumonia in the absence of any vaccine, including COVID-19 specific vaccine, measured over 12 months following randomisation in healthcare exposed to SARS-CoV-2.	Estimand 23.2<br><br>Population: ITT population<br>Outcome: pneumonia by 12 months<br>Interventions: BCG vs Placebo<br>Handling of Intercurrent events:<br>- any vaccine, including COVID-19 specific vaccine (Hypothetical strategy)<br>Summary Measure: Adjusted difference in proportion of participants

8.7.2. ANALYSIS

The absolute and relative frequencies of participants with the outcome prior to 12 months will be presented by intervention group. The outcome will be compared between the BCG group and the placebo group using a difference in proportions estimated using the same time-to-event analysis approach adopted for primary outcomes 1 and 2 (flexible parametric survival model). A two-sided bias-corrected 95% CI for the difference in proportion (BCG – Control) and a bootstrap p-value will be calculated with bootstrap standard errors. A Kaplan-Meier survival curve will also be presented by treatment arm for descriptive purposes.
For participants that meet the outcome the date of the first day with symptoms associated to a febrile or respiratory illness will be taken as the outcome. Participants who did not have pneumonia within 12 months will be censored according to the same rules used for the censoring of outcomes 17 (section 8.1.2).

Should the overall number of pneumonia events be so small that the analysis model described has computational difficulties, the outcome will be analysed using the Cox's proportional hazards model, adjusted for the stratification factors used during randomisation (age group, presence of comorbidity, geographical location -Europe/Australia/South America), and presented as the hazard ratio rather than the difference in proportion.

The analysis will be adjusted for the stratification factors only if a minimum number of events is observed, namely 1 event per strata per intervention group.

8.8. NEED OF OXYGEN (#23)

8.8.1. ESTIMANDS

Objective	Estimand
To determine if BCG vaccination compared with placebo reduces the need for oxygen therapy irrespective of receiving any other vaccine (including COVID-19 specific vaccine), over the 12 months following randomisation, in healthcare workers exposed to SARS-CoV-2.	Estimand 24.1<br><br>Population: ITT population<br>Outcome: need of oxygen therapy by 12 months<br>Interventions: BCG vs Placebo<br>Handling of Intercurrent events:<br>- any vaccine, including COVID-19 specific vaccine (Treatment Policy strategy)<br>Summary Measure: as for estimand 24.1
To determine if BCG vaccination compared with placebo reduces the need for oxygen therapy in the absence of any vaccine, measured over 12 months following randomisation in healthcare exposed to SARS-CoV-2.	Estimand 24.2<br><br>Population: ITT population<br>Outcome: need of oxygen therapy by 12 months<br>Interventions: BCG vs Placebo<br>Handling of Intercurrent events:<br>- any vaccine, including COVID-19 specific vaccine (Hypothetical strategy)<br>Summary Measure: Adjusted difference in proportion of participants

8.8.2. ANALYSIS

As per section 8.7.2.

8.9. ADMISSION TO CRITICAL CARE FOLLOWING A FEBRILE OR RESPIRATORY ILLNESS (#25)

8.9.1. ESTIMANDS

Objective	Estimand
To determine if BCG vaccination compared with placebo reduces admission to critical care following a febrile or respiratory illness irrespective of receiving any vaccine, including COVID-19 specific vaccine (including COVID-19 specific vaccine), over the 12 months following randomisation, in healthcare workers exposed to SARS-CoV-2.	Estimand 25.1<br><br>Population: ITT population<br>Outcome: admission to critical care following a febrile or respiratory illness by 12 months<br>Interventions: BCG vs Placebo<br>Handling of Intercurrent events:<br>- any vaccine, including COVID-19 specific vaccine (Treatment Policy strategy)<br>Summary Measure: as for estimand 25.1
To determine if BCG vaccination compared with placebo reduces admission to critical care following a febrile or respiratory illness in the absence of any vaccine, measured over 12 months following randomisation in healthcare exposed to SARS-CoV-2.	Estimand 25.2<br><br>Population: ITT population<br>Outcome: admission to critical care following a febrile or respiratory illness by 12 months<br>Interventions: BCG vs Placebo<br>Handling of Intercurrent events:<br>- any vaccine, including COVID-19 specific vaccine (Hypothetical strategy)<br>Summary Measure: Adjusted difference in proportion of participants

8.9.2. ANALYSIS

As per section 8.7.2.

Additionally, in the subgroups of participants who were admitted to critical care following a febrile or respiratory illness, the mean and standard deviation (or median and IQR if distribution is skewed) of the duration of critical care will be calculated and presented by intervention group.

8.10. NEED OF MV FOR A FEBRILE OR RESPIRATORY ILLNESS (#26)

8.10.1. ESTIMANDS

Objective	Estimand
To determine if BCG vaccination compared with placebo reduces Need of MV for a febrile or respiratory illness irrespective of receiving any vaccine, including COVID-19 specific vaccine , over the 12 months following randomisation, in healthcare workers exposed to SARS-CoV-2.	Estimand 26.1<br><br>Population: ITT population<br>Outcome: Need of MV for a febrile or respiratory illness by 12 months<br>Interventions: BCG vs Placebo<br>Handling of Intercurrent events:<br>- any vaccine, including COVID-19 specific vaccine (Treatment Policy strategy)<br>Summary Measure: as for estimand 26.1
To determine if BCG vaccination compared with placebo reduces the need of MV for a febrile or respiratory illness in the absence of any vaccine, measured over 12 months following randomisation in healthcare exposed to SARS-CoV-2.	Estimand 26.2<br><br>Population: ITT population<br>Outcome: Need of MV for a febrile or respiratory illness by 12 months<br>Interventions: BCG vs Placebo<br>Handling of Intercurrent events:<br>- any vaccine, including COVID-19 specific vaccine (Hypothetical strategy)<br>Summary Measure: Adjusted difference in proportion of participants

8.10.2. ANALYSIS

As per section 8.7.2.

Additionally, in the subgroup of participants who needed MV for a febrile or respiratory illness, the mean and standard deviation (or median and IQR if distribution is skewed) of the duration of MV will be calculated and presented by intervention group.

8.11. DEATH AS A CONSEQUENCE OF AN EPISODE OF FEVER OR RESPIRATORY ILLNESS (#27)

8.11.1. ESTIMANDS

Objective	Estimand
To determine if BCG vaccination compared with placebo reduces the incidence of death as a consequence of an episode of fever or respiratory illness irrespective of receiving any vaccine, including COVID-19 specific vaccine, measured over 12 months following randomisation in healthcare exposed to SARS-CoV-2.	Estimand 27.1<br><br>Population: ITT population<br>Outcome: death as a consequence of an episode of fever or respiratory illness by 12 months<br>Interventions: BCG vs Placebo<br>Handling of Intercurrent events:<br>- any vaccine, including COVID-19 specific vaccine (Treatment Policy strategy)<br>Summary Measure: as for estimand 27.1
To determine if BCG vaccination compared with placebo reduces the incidence of death as a consequence of an episode of fever or respiratory illness in the absence of receiving any vaccine, including COVID-19 specific vaccine, measured over 12 months following randomisation in healthcare exposed to SARS-CoV-2.	Estimand 27.2<br><br>Population: ITT population<br>Outcome: death as a consequence of an episode of fever or respiratory illness by 12 months<br>Interventions: BCG vs Placebo<br>Handling of Intercurrent events:<br>- any vaccine, including COVID-19 specific vaccine (Hypothetical strategy)<br>Summary Measure: Adjusted difference in proportion of participants

8.11.2. ANALYSIS

As per section 8.7.2.

8.12. HOSPITALISATION FOR AN EPISODE OF FEVER OR RESPIRATORY ILLNESS (#28)

8.12.1. ESTIMANDS

Objective	Estimand
To determine if BCG vaccination compared with placebo reduces the incidence of hospitalisation for an episode of fever or respiratory illness irrespective of receiving any vaccine, including COVID-19 specific vaccine, measured over 12 months following randomisation in healthcare exposed to SARS-CoV-2.	Estimand 28.1<br><br>Population: ITT population<br>Outcome: hospitalisation for an episode of fever or respiratory illness by 12 months<br>Interventions: BCG vs Placebo<br>Handling of Intercurrent events:<br>- any vaccine, including COVID-19 specific vaccine (Treatment Policy strategy)<br>Summary Measure: as for estimand 28.1
To determine if BCG vaccination compared with placebo reduces the incidence of hospitalisation for an episode of fever or respiratory illness in the absence of receiving any vaccine, including COVID-	Estimand 28.2<br><br>Population: ITT population

Objective	Estimand
19 specific vaccine, measured over 12 months following randomisation in healthcare exposed to SARS-CoV-2.	Outcome: hospitalisation for an episode of fever or respiratory illness by 12 months<br>Interventions: BCG vs Placebo<br>Handling of Intercurrent events:<br>- any vaccine, including COVID-19 specific vaccine (Hypothetical strategy)<br>Summary Measure: Adjusted difference in proportion of participants

8.12.2. ANALYSIS

Same as per 8.7.2.

In the subset of participants who were admitted to hospital due to COVID-19, the mean and standard deviation (or median and IQR if not normally distributed) of the hospital stay will be calculated and presented by intervention group.

8.13. NUMBER OF DAYS OF UNPLANNED ABSENTEEISM FOR AN ACUTE ILLNESS OR HOSPITALISATION (#29a and #29b)

8.13.1. ESTIMANDS

Objective	Estimand
To determine if BCG vaccination compared with placebo reduces absenteeism, measured over 6 months following randomisation in healthcare workers who did not have a previous SARS-CoV-2 positive test result when assessed at time of randomisation.	Estimand 29a.1<br><br>Population: mITT population<br>Outcome: Number of days of unplanned absenteeism for an acute illness or hospitalisation by 6 months<br>Interventions: BCG vs Placebo<br>Handling of Intercurrent events:<br>- any vaccine, including COVID-19 specific vaccine (Treatment Policy strategy)<br>Summary Measure: Mean difference
To determine if BCG vaccination compared with placebo reduces absenteeism, measured over 6 months following randomisation in healthcare exposed to SARS-CoV-2.	Estimand 29a.2<br><br>Population: ITT population<br>Outcome: as for estimand 29a.1<br>Interventions: as for estimand 29a.1<br>Handling of Intercurrent events:<br>- as for estimand 29a.1<br>Summary Measure: as for estimand 29a.1
To determine if BCG vaccination compared with placebo reduces absenteeism, measured over 12 months following randomisation in healthcare.	Estimand 29b.1<br><br>Population: ITT population<br>Outcome: Number of days of unplanned absenteeism for an acute illness or hospitalisation by 12 months<br>Interventions: BCG vs Placebo<br>Handling of Intercurrent events:<br>- any vaccine, including COVID-19 specific vaccine (Treatment Policy strategy)<br>Summary Measure: Mean difference

8.13.2. ANALYSIS

The median and IQR for the number of days of unplanned absenteeism for an acute illness or hospitalisation will be presented by arm.
The difference between BCG and placebo groups will be summarised as difference in the logs of expected number of days and its 95%CI estimated using a Zero-Inflated Negative Binomial (ZINB) model, adjusting for stratification factors. The analysis will be done using the zinb command in Stata, with the inflate() option. The inflate() option will be used to indicate whether the participant had one or more days of unplanned absenteeism for an acute illness or hospitalisation.

8.14. ADVERSE EVENTS and SERIOUS ADVERSE EVENTs

The number and proportion of participants with 1 or more adverse events over the 3 months following randomisation will be described overall as well as by type, severity (grade 0-4) and relationship to intervention. All results summaries will be presented by intervention group in the Safety population.

9. META-ANALYSIS

The data from the participants in the two stages of the trial will be combined in a meta-analysis for secondary analyses of all the non-COVID-19 outcomes.

The original trial plan included a meta-analysis of all the outcomes (both COVID-19- and non-COVID-19-related outcomes) combining data from Stage 1 and Stage 2 participants. However, in Stage 1 healthcare workers were recruited only in Victoria and Western Australia, both of which states had almost negligible COVID-19 exposure risk during the trial period (30th Mar 2020 to 13th May 2020). In light of this, the overwhelming majority of Stage 1 blood samples are likely to be seronegative. Moreover, with a low prevalence of COVID-19, there is a high probability that positive SARS-CoV-2 serology results are false positive. For these reasons, in December 2021 the BRACE team decided it was not justifiable to devote extra resources (time and costs) to the data cleaning of potential COVID-19 episodes and to testing SARS-CoV-2 serology for all participants in Stage 1. As a consequence, the meta-analysis will only be run on non-COVID-19 related outcomes and its main objective will be to determine if BCG vaccination compared with control reduces the rate and severity of febrile or other non-COVID-19 illness.

The analyses of these secondary non-COVID-19 outcomes will be the same as described in section 8 conducted on all Stage 1 and Stage 2 participants.

10. PLANNED ANALYSES

ANALYSIS OF THE 6 MONTHS COVID-19 OUTCOMES, Stage 2 participants
The first manuscript will include results from the analysis of the COVID-19-related outcomes collected in the first 6 months of each participant’s participation in the trial and one non-COVID-19 related outcome at 6 months (29a). This analysis will include only participants recruited in Stage 2 of the trial.

ANALYSIS OF THE 12 MONTHS COVID-19 OUTCOMES, Stage 2 participants
A future manuscript will present results of the analyses of the COVID-19 related outcomes collected at 12 months. This analysis will include only participants recruited in Stage 2 of the trial.

ANALYSIS OF THE 12 MONTHS NON COVID-19 RELATED OUTCOMES (META ANALYSIS), Stages 1 and 2.
A future manuscript will present results of the analyses of all the non-COVID-19 related outcomes collected at 12 months. As addressed in section 9, this analysis will combine participants recruited in Stages 1 and 2 of the trial.

ANALYSIS OF THE EXPLORATORY OUTCOMES
A future manuscript will present results of the analyses of all the exploratory outcomes at 12 months combining participants recruited in Stages 1 and 2 of the trial.

11. REFERENCES

1. Pocock SJ. Group sequential methods in the design and analysis of clinical trials. Biometrika. 1977;64:191–199. [Google Scholar]

2. Royston P. Flexible Parametric Alternatives to the Cox Model: Update. The Stata Journal 2004; 4: 98-101. DOI:10.1177/1536867x0100400112.

3. Pittet LF, Messina NL, Gardiner K, Orsini F, Abruzzo V, Bannister S, Bonten M, Campbell JL, Croda J, Dalcolmo M, Elia S, Germano S, Goodall C, Gwee A, Jamieson T, Jardim B, Kollmann TR, Guimarães Lacerda MV, Lee KJ, Legge D, Lucas M, Lynn DJ, McDonald E, Manning L, Munns CF, Perrett KP, Prat Aymerich C, Richmond P, Shann F, Sudbury E, Villanueva P, Wood NJ, Lieschke K, Subbarao K, Davidson A, Curtis N; BRACE trial Consortium Group. BCG vaccination to reduce the impact of COVID-19 in healthcare workers: Protocol for a randomised controlled trial (BRACE trial). BMJ Open. 2021 Oct 28;11(10):e052101. doi:10.1136/bmjopen-2021-052101. PMID: 34711598; PMCID: PMC8557250.

12. SIGNATURES PAGE

Signature of Chief Principal Investigator:	*Nigel Curtis*<br>Nigel Curtis (Aug 11, 2022 15:09 GMT+10)	Aug 11, 2022
Print Name	Prof. Nigel Curtis	Date
Signature of Trial Statistician:	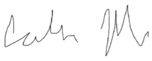	Aug 11, 2022
Print Name	Dr Cecilia Moore	Date
Signature of Trial Statistician:	*KJLee*<br>KJLee (Aug 11, 2022 16:56 GMT+10)	Aug 11, 2022
Print Name	Prof. Katherine Lee	Date

BRACE_SAP_V2.0 Date 10Aug2022 [CL]

Final Audit Report 2022-08-11

Created:	2022-08-11
By:	Thilanka Morawakage (thilanka.morawakage@mcri.edu.au)
Status:	Signed
Transaction ID:	CBJCHBCAABAAQEwaWeDJbYq-LL1p-W7nJCtPSekafM9p

"BRACE_SAP_V2.0 Date 10Aug2022 [CL]" History

Document created by Thilanka Morawakage (thilanka.morawakage@mcri.edu.au)
2022-08-11 - 4:32:47 AM GMT- IP address: 165.225.226.254

Document emailed to nigel.curtis@rch.org.au for signature
2022-08-11 - 4:34:17 AM GMT

Document emailed to cecilia.moore@mcri.edu.au for signature
2022-08-11 - 4:34:17 AM GMT

Document emailed to katherine.lee@mcri.edu.au for signature
2022-08-11 - 4:34:17 AM GMT

Email viewed by cecilia.moore@mcri.edu.au
2022-08-11 - 4:59:22 AM GMT- IP address: 89.184.212.227

Signer cecilia.moore@mcri.edu.au entered name at signing as Cecilia Moore
2022-08-11 - 5:00:49 AM GMT- IP address: 165.225.227.5

Document e-signed by Cecilia Moore (cecilia.moore@mcri.edu.au)
Signature Date: 2022-08-11 - 5:00:51 AM GMT - Time Source: server- IP address: 165.225.227.5

Email viewed by nigel.curtis@rch.org.au
2022-08-11 - 5:08:54 AM GMT- IP address: 203.16.41.5

Signer nigel.curtis@rch.org.au entered name at signing as Nigel Curtis
2022-08-11 - 5:09:08 AM GMT- IP address: 203.16.41.5

Document e-signed by Nigel Curtis (nigel.curtis@rch.org.au)
Signature Date: 2022-08-11 - 5:09:10 AM GMT - Time Source: server- IP address: 203.16.41.5

Email viewed by katherine.lee@mcri.edu.au
2022-08-11 - 6:55:31 AM GMT- IP address: 77.95.117.75

Signer katherine.lee@mcri.edu.au entered name at signing as KJLee

2022-08-11 - 6:55:59 AM GMT- IP address: 165.225.226.222

Document e-signed by KJLee (katherine.lee@mcri.edu.au)

Signature Date: 2022-08-11 - 6:56:01 AM GMT - Time Source: server- IP address: 165.225.226.222

Agreement completed.

2022-08-11 - 6:56:01 AM GMT

RCH HREC/protocol no: 62586
NCT04327206

BCG vaccination to Reduce the impact of febrile and respiratory illness in healthcare workers (BRACE) Trial

Final Statistical Report

Based on Statistical Analysis Plan version 2.0, 10Aug2022
Based on Protocol version 12.0, 17May2022

Document Version History

Version Date	Version	Author	Signature	Change Description	Reason/Comment
13-Jun-2023	1	Francesca Orsini	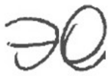	Initial release.	Not applicable.
2-Nov-2023	2	Francesca Orsini	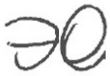	Analysis run on updated dataset.	

Contents

1. BASELINE CHARACTERISTICS 6
Table 1 Baseline Characteristics of ITT Population 6

2. FOLLOW-UP 9
Table 2 Follow-up Characteristics of ITT Population 9

3. OUTCOMES 11
3.1 FEVER OR RESPIRATORY ILLNESS BY 12 MONTHS 11
Table 3.1 Estimand 17.1 – Incidence of fever or respiratory illness (ITT), Treatment policy Strategy 11
Table 3.2 Estimand 17.2 – Incidence of fever or respiratory illness (ITT), Hypothetical Strategy 12
Figure 3.1 Incidence of Fever and respiratory illness (ITT) – 17.1 Treatment policy Strategy (a), 17.2 Hypothetical Strategy (b) 13
3.2 SEVERE FEVER OR RESPIRATORY ILLNESS BY 12 MONTHS 15
Table 3.3 Estimand 18.1 – Incidence of Severe fever or respiratory illness (ITT), Treatment policy Strategy 15
Table 3.4 Estimand 18.2 – Incidence of Severe fever or respiratory illness (ITT), Hypothetical Strategy 16
Figure 3.2 Incidence of Severe Fever and respiratory illness (ITT) – 18.1 Treatment policy Strategy (a), 18.2 Hypothetical Strategy (b) 17
3.3 NUMBER OF EPISODES FEVER OR RESPIRATORY ILLNESS BY 12 MONTHS 19
Table 3.5 Estimand 19.1 – Number of episodes fever or respiratory illness (ITT), Treatment policy Strategy 19
Table 3.6 Estimand 19.2 – Number of episodes fever or respiratory illness (ITT), Hypothetical Strategy 20
3.4 NUMBER DAYS UNABLE TO WORK DUE TO FEVER OR RESPIRATORY ILLNESS BY 12 MONTHS 21
3.4.1 NUMBER DAYS UNABLE TO WORK, TREATMENT POLICY STRATEGY 21
Table 3.7 Estimand 20.1 – Number days unable to work due to fever or respiratory illness (ITT), Treatment policy Strategy 21
3.4.1.1 SUBGROUP ANALYSES BY AGE: NUMBER DAYS UNABLE TO WORK, TREATMENT POLICY STRATEGY 22
Table 3.7.1 Estimand 20.1 by AGE – Number days unable to work due to fever or respiratory illness (ITT), Treatment policy Strategy 22
3.4.1.2 SUBGROUP ANALYSES BY PRESENCE OF COMORBIDITIES: NUMBER DAYS UNABLE TO WORK, TREATMENT POLICY STRATEGY 23
Table 3.7.2 Estimand 20.1 by PRESENCE OF COMORBIDITIES – Number days unable to work due to fever or respiratory illness (ITT), Treatment policy Strategy 23
3.4.2 NUMBER DAYS UNABLE TO WORK, HYPOTHETICAL STRATEGY 24
Table 3.8 Estimand 20.2 – Number days unable to work due to fever or respiratory illness (ITT), Hypothetical Strategy 24
3.4.2.1 SUBGROUP ANALYSES BY AGE: NUMBER DAYS UNABLE TO WORK, HYPOTHETICAL STRATEGY 25
Table 3.8.1 Estimand 20.2 – Number days unable to work due to fever or respiratory illness (ITT), Hypothetical Strategy ... 25
3.5 NUMBER DAYS CONFINED TO BED DUE FEVER OR RESPIRATORY ILLNESS BY 12 MONTHS 26
Table 3.9 Estimand 21.1 – Number days confined to bed due to fever or respiratory illness (ITT), Treatment policy Strategy 26
Table 3.10 Estimand 21.2 – Number days confined to bed due to fever or respiratory illness (ITT), Hypothetical Strategy ... 27
3.6 NUMBER DAYS WITH SYMPTOMS DUE FEVER OR RESPIRATORY ILLNESS BY 12 MONTHS 28
3.6.1 NUMBER OF DAYS WITH SYMPTOMS, TREATMENT POLICY STRATEGY 28
Table 3.11 Estimand 22.1 – Number days with symptoms due to fever or respiratory illness (ITT), Treatment policy Strategy 28

3.6.1.1 SUBGROUP ANALYSES BY PRESENCE OF COMORBIDITIES: NUMBER DAYS WITH SYMPTOMS, TREATMENT POLICY STRATEGY 29
Table 3.11.1 Estimand 22.1 by PRESENCE OF COMORBIDITIES – Number days with symptoms due to fever or respiratory illness (ITT), Treatment policy Strategy ... 29
3.6.1.2 SUBGROUP ANALYSES BY GEOGRAPHICAL AREA: NUMBER DAYS WITH SYMPTOMS, TREATMENT POLICY STRATEGY 30
Table 3.11.2 Estimand 22.1 by GEOGRAPHICAL AREA – Number days with symptoms due to fever or respiratory illness (ITT), Treatment policy Strategy ... 30
3.6.2 NUMBER OF DAYS WITH SYMPTOMS, HYPOTHETICAL STRATEGY ... 32
Table 3.12 Estimand 22.2 – Number days with symptoms due to fever or respiratory illness (ITT), Hypothetical Strategy ... 32
3.6.2.1 SUBGROUP ANALYSES BY PRESENCE OF COMORBIDITIES: NUMBER DAYS WITH SYMPTOMS, HYPOTHETICAL STRATEGY 33
Table 3.12.1 Estimand 22.2 by PRESENCE OF COMORBIDITIES – Number days with symptoms due to fever or respiratory illness (ITT), hypothetical Strategy ... 33
Table 3.12.2 Estimand 22.2 by PRESENCE OF GEOGRAPHICAL AREA – Number days with symptoms due to fever or respiratory illness (ITT), hypothetical Strategy ... 34
3.7 PNEUMONIA WITHIN A FEBRILE OR RESPIRATORY ILLNESS BY 12 MONTHS ... 36
Table 3.13 Estimand 23.1 – Incidence of pneumonia (ITT), Treatment policy Strategy ... 36
Table 3.14 Estimand 23.2 – Incidence of pneumonia (ITT), Hypothetical Strategy ... 37
3.8 NEED FOR OXYGEN THERAPY FOR A FEBRILE OR RESPIRATORY ILLNESS BY 12 MONTHS ... 38
Table 3.15 Estimand 24.1 – Oxygen therapy (ITT), Treatment policy Strategy ... 38
Table 3.16 Estimand 24.2 – Oxygen therapy (ITT), Hypothetical Strategy ... 39
3.9 ADMISSION TO CRITICAL CARE FOR A FEBRILE OR RESPIRATORY ILLNESS BY 12 MONTHS ... 40
Table 3.17 Estimand 25.1 –Admission to critical care (ITT), Treatment policy Strategy ... 40
Table 3.18 Estimand 25.2 –Admission to critical care (ITT), Hypothetical Strategy ... 41
3.10 NEED FOR MECHANICAL VENTILATION (MV) FOR A FEBRILE OR RESPIRATORY ILLNESS BY 12 MONTHS ... 42
Table 3.19 Estimand 26.1 – Need of Mechanical Ventilation (ITT), Treatment policy Strategy ... 42
Listing 3.1 Estimand 26.1 –List of participants who needed Mechanical Ventilation (ITT), Treatment policy Strategy ... 43
Table 3.20 Estimand 26.2 – Need of Mechanical Ventilation (ITT), Hypothetical Strategy ... 44
3.11 DEATHS AS A CONSEQUENCE OF AN EPISODE OF FEVER OR RESPIRATORY ILLNESS BY 12 MONTHS ... 45
Table 3.21 Estimand 27.1 – Death (ITT), Treatment Policy Strategy ... 45
Listing 3.2 Estimand 27.1 –List of participants who died as a consequence of an episode of fever or respiratory illness (ITT), Treatment policy Strategy ... 46
Table 3.22 Estimand 27.2 – Death (ITT), Hypothetical Strategy ... 47
3.12 HOSPITALISATION FOR A FEBRILE OR RESPIRATORY ILLNESS AND DURATION OF HOSPITALISATION BY 12 MONTHS 48
3.12.1 HOSPITALISATION FOR A FEBRILE OR RESPIRATORY ILLNESS, TREATMENT POLICY STRATEGY ... 48
Table 3.23.1 Estimand 28.1 Hospitalisation for a febrile or respiratory illness (ITT), Treatment policy Strategy ... 48
Listing 3.3 Estimand 28.1 –List of participants who were hospitalisation (ITT), Treatment policy Strategy ... 49
3.12.2 HOSPITALISATION FOR A FEBRILE OR RESPIRATORY ILLNESS, HYPOTETICAL STRATEGY ... 51
Table 3.24.1 Estimand 28.2 Hospitalisation for a febrile or respiratory illness (ITT), Hypothetical Strategy ... 51
Listing 3.4 Estimand 28.2 –List of participants who were hospitalisation (ITT), Hypothetical Strategy ... 52
3.13 NUMBER OF DAYS OF UNPLANNED ABSENTEEISM FOR AN ACUTE ILLNESS OR HOSPITALISATION BY 12 MONTHS 53
Table 3.25 Estimand 29.1b Number of days of unplanned absenteeism for an acute illness or hospitalisation by 12 months (ITT), Treatment Policy Strategy ... 53

3.13.1 SUBGROUP ANALYSES BY PRESENCE OF COMORBIDITIES: NUMBER OF DAYS OF UNPLANNED ABSENTEEISM FOR AN ACUTE ILLNESS OR HOSPITALISATION BY 12 MONTHS 54

Table 3.25.1 Estimand 29.1b by PRESENCE OF COMORBIDITIES – Number of days of unplanned absenteeism for an acute illness or hospitalisation by 12 months (ITT), Treatment Policy Strategy 54

3.13.2 SUBGROUP ANALYSES BY GEOGRAPHICAL AREA: NUMBER OF DAYS OF UNPLANNED ABSENTEEISM FOR AN ACUTE ILLNESS OR HOSPITALISATION BY 12 MONTHS 55

Table 3.25.2 Estimand 29.1b by PRESENCE OF COMORBIDITIES – Number of days of unplanned absenteeism for an acute illness or hospitalisation by 12 months (ITT), Treatment Policy Strategy 55

LIST OF ABBREVIATIONS

BCG	Bacille Calmette-Guérin
BMI	Body Mass Index
BRACE	BCG vaccination to Reduce the impAct of COVID-19 in hEalthcare workers
CI	Confidence Interval
COVID-19	Coronavirus Disease of 2019
ICU	Intensive Care Unit
ITT	Intent-To-Treat
mITT	Modified Intent-To-Treat
PCR	Polymerase Chain Reaction
RAT	Rapid Antigen Test
SARS-CoV-2	Severe Acute Respiratory Syndrome Coronavirus 2
SD	Standard Deviation
SE	Standard Error

1. BASELINE CHARACTERISTICS

Table 1 Baseline Characteristics of ITT Population

	**BCG** N=3417	**Control** N=3411
Participants included in itt_pop	3417	3411
**STAGE**		
1	1418/3417 (41.50%)	1422/3411 (41.69%)
2	1999/3417 (58.50%)	1989/3411 (58.31%)
**BASELINE CHARACTERISTICS**		
**SEX Female**	2511/3417 (73.49%)	2593/3411 (76.02%)
**Age at randomisation**	3417, 42.0 (12.1)	3411, 42.0 (12.0)
**COUNTRY**		
Australia-Stage 1	1418/3417 (41.50%)	1422/3411 (41.69%)
Australia-Stage 2	216/3417 (6.32%)	206/3411 (6.04%)
Europe	498/3417 (14.57%)	500/3411 (14.66%)
South America	1285/3417 (37.61%)	1283/3411 (37.61%)
**BMI**		
< 18.5 kg/m2	42/3285 (1.28%)	47/3281 (1.43%)
18.5 to 24.9 kg/m2	1420/3285 (43.23%)	1439/3281 (43.86%)
25 to 29.9 kg/m2	1165/3285 (35.46%)	1161/3281 (35.39%)
>=30 kg/m2	658/3285 (20.03%)	634/3281 (19.32%)
MISSING	132	130
**WORK PLACE**		
Emergency Department	202/3417 (5.91%)	228/3411 (6.68%)
Intensive Care Unit / High Dependency Unit	268/3417 (7.84%)	245/3411 (7.18%)
Operating Theatre	184/3417 (5.38%)	191/3411 (5.60%)
General ward	564/3417 (16.51%)	545/3411 (15.98%)
Pharmacy	93/3417 (2.72%)	100/3411 (2.93%)
Other ward/area	1726/3417 (50.51%)	1733/3411 (50.81%)
Paramedic / Ambulance	24/3417 (0.70%)	24/3411 (0.70%)
Aged care facility	31/3417 (0.91%)	33/3411 (0.97%)
Practice outside of hospital	325/3417 (9.51%)	312/3411 (9.15%)
**WORK ROLE**		
Nurse/Midwife	981/3417 (28.71%)	956/3411 (28.03%)
Doctor	472/3417 (13.81%)	467/3411 (13.69%)
Allied Health	656/3417 (19.20%)	672/3411 (19.70%)
PSA/hospital maintenance	415/3417 (12.15%)	395/3411 (11.58%)
Administrative/clerical staff	500/3417 (14.63%)	494/3411 (14.48%)
Other	91/3417 (2.66%)	91/3411 (2.67%)
Paramedic	29/3417 (0.85%)	30/3411 (0.88%)
Carer	21/3417 (0.61%)	21/3411 (0.62%)
Dentist/dental therapy	35/3417 (1.02%)	43/3411 (1.26%)
Community Health Agent	86/3417 (2.52%)	101/3411 (2.96%)
Scientist (medical/research)	131/3417 (3.83%)	141/3411 (4.13%)
**PATIENT CONTACT WEEKLY**		
No direct patient contact	593/3417 (17.35%)	606/3410 (17.77%)
< 10 hours	554/3417 (16.21%)	575/3410 (16.86%)
10 - 20 hours	559/3417 (16.36%)	516/3410 (15.13%)
>20 hours	1711/3417 (50.07%)	1713/3410 (50.23%)
MISSING	0	1

	BCG<br>N=3417	Control<br>N=3411
**SMOKING**		
No	3130/3417 (91.60%)	3091/3410 (90.65%)
Yes,rarely (1 or 2 cigarettes a month)	60/3417 (1.76%)	78/3410 (2.29%)
Yes,occasionally (1 or 2 cigarettes a week)	67/3417 (1.96%)	75/3410 (2.20%)
Yes,regularly	160/3417 (4.68%)	166/3410 (4.87%)
MISSING	0	1
**BCG VACCINATION IN THE PAST**		
No	1153/3417 (33.74%)	1164/3410 (34.13%)
Yes - 1 to 5 years ago	53/3417 (1.55%)	49/3410 (1.44%)
Yes - Greater than 5 years ago	2211/3417 (64.71%)	2197/3410 (64.43%)
MISSING	0	1
**BCG SCAR AT RANDOMISATION**		
No	1208/3114 (38.79%)	1162/2949 (39.40%)
Yes	1812/3114 (58.19%)	1611/2949 (54.63%)
Unsure	94/3114 (3.02%)	176/2949 (5.97%)
MISSING	303	462
**TB EXPOSURE**		
No	3368/3417 (98.57%)	3365/3410 (98.68%)
Yes	29/3417 (0.85%)	24/3410 (0.70%)
Not sure	20/3417 (0.59%)	21/3410 (0.62%)
MISSING	0	1
**POSITIVE TUBERCULIN SKIN TEST OR MANTOUX TEST**		
No	2851/3417 (83.44%)	2858/3410 (83.81%)
Yes	205/3417 (6.00%)	224/3410 (6.57%)
Not sure	361/3417 (10.56%)	328/3410 (9.62%)
MISSING	0	1
**COMORBIDITIES**		
**Presence of comorbidities (Excluding BMI>=30km/m2)**	613/3417 (17.94%)	625/3411 (18.32%)
Number of co-morbidities (1,2,3)		
1	552/613 (90.05%)	577/625 (92.32%)
2	60/613 (9.79%)	45/625 (7.20%)
3	1/613 (0.16%)	3/625 (0.48%)
**Presence of comorbidities (ANY)**	1060/3313 (32.00%)	1054/3305 (31.89%)
MISSING	104	106
**Diabetes**	89/3417 (2.60%)	104/3411 (3.05%)
What type of Diabetes?		
Type 1 diabetes only	15/89 (16.85%)	14/104 (13.46%)
Type 2 diabetes only	69/89 (77.53%)	79/104 (75.96%)
Type 1 and Type 2 diabetes	1/89 (1.12%)	1/104 (0.96%)
Unsure	3/89 (3.37%)	8/104 (7.69%)
Other	1/89 (1.12%)	2/104 (1.92%)
**Chronic respiratory disease**	230/3417 (6.73%)	208/3411 (6.10%)
**Cardiovascular disease (any)**	354/3417 (10.36%)	364/3411 (10.67%)
Ischaemic heart disease	6/354 (1.69%)	9/364 (2.47%)
Congestive heart disease	2/354 (0.56%)	3/364 (0.82%)
Other	30/354 (8.47%)	19/364 (5.22%)
Unsure	1/354 (0.28%)	1/364 (0.27%)
**Hypertension**	333/3417 (9.75%)	344/3411 (10.09%)
**BMI>=30 kg/m2**	658/3285 (20.03%)	634/3281 (19.32%)
MISSING	132	130

	BCG<br>N=3417	Control<br>N=3411
**OTHER SUBGROUPS**		
**Subgroup 1 – By age group (<40 / 40-59 / 60+)**		
<40 years old	1592/3417 (46.59%)	1594/3411 (46.73%)
40-59 years old	1566/3417 (45.83%)	1557/3411 (45.65%)
60+ years old	259/3417 (7.58%)	260/3411 (7.62%)
**Subgroup 3 – By geographical Location and stage (Australia=Stage1 vs Australia=S**		
Australia-Stage 1	1418/3417 (41.50%)	1422/3411 (41.69%)
Australia-Stage 2	216/3417 (6.32%)	206/3411 (6.04%)
Europe	498/3417 (14.57%)	500/3411 (14.66%)
South America	1285/3417 (37.61%)	1283/3411 (37.61%)
**Subgroup 4 – By sex (F vs M)**		
Female	2511/3412 (73.59%)	2593/3411 (76.02%)
Male	901/3412 (26.41%)	818/3411 (23.98%)
MISSING	5	0
**Subgroup 5 – By BCG in the past or not**		
No BCG in the past	1153/3417 (33.74%)	1164/3410 (34.13%)
BCG in the past	2264/3417 (66.26%)	2246/3410 (65.87%)
MISSING	0	1

2. FOLLOW-UP

Table 2 Follow-up Characteristics of ITT Population

	BCG N=3417	Control N=3411
**Stage**		
1	1418	1422
2	1999	1989
**INTERVENTION RECEIVED - Stage 1**		
(Stage 1)		
BCG NOT received	5/1418 (0.35%)	1420/1422 (99.86%)
BCG received	1413/1418 (99.65%)	2/1422 (0.14%)
Specifics of vaccines receaved at randomisation		
FLU + BCG	1412/1418 (99.58%)	2/1422 (0.14%)
FLU only	2/1418 (0.14%)	1394/1422 (98.03%)
BCG only	1/1418 (0.07%)	0/1422 (0.00%)
no vax received	3/1418 (0.21%)	26/1422 (1.83%)
**INTERVENTION RECEIVED - Stage 2**		
Specifics of vaccines receaved at randomisation		
BCG Received	1988/1999 (99.45%)	8/1989 (0.40%)
BCG NOT Received	4/1999 (0.20%)	1978/1989 (99.45%)
Not sure which Intervention Received	1/1999 (0.05%)	0/1989 (0.00%)
No Intervention received (Stage 2)	6/1999 (0.30%)	3/1989 (0.15%)
Vaccination given on same day of randomisation	1987/1993 (99.70%)	1974/1986 (99.40%)
Vaccination NOT given on same day of randomisation	6/1993 (0.30%)	12/1986 (0.60%)
MISSING	6	3
Received opposite intervention (no reason specified)	1/1999 (0.05%)	8/1989 (0.40%)
Received opposite intervention due to informatic bug	3/1999 (0.15%)	0/1989 (0.00%)
Not possible to determine what the participant received	1/1999 (0.05%)	0/1989 (0.00%)
**FOLLOW-UP**		
Months of Follow-up, Median(IQR)	12.0 (12.0-12.0)	12.0 (12.0-12.0)
Months of Follow-up Prior to First Vaccination, Median(IQR)	5.4 (2.0-11.0)	5.0 (1.9-10.8)
Followed for 12 months	3151/3417 (92.22%)	2971/3411 (87.10%)
**WITHDRAWALS IN THE FIRST 12 MONTHS**		
Withdrawn from the study	88/3417 (2.58%)	153/3411 (4.49%)
Reason for withdrawal		
Disappointed regarding allocation following randomisation	2/88	6/153
Study has become a burden	24/88	41/153
Adverse event	1/88	0/153
Serious concurrent medical condition	5/88	3/153
Relocation	9/88	19/153
No reason given	37/88	72/153
Ppersonal reason / change in personal life	1/88	5/153
Other	9/88	7/153
**LOST TO FOLLOW-UP IN THE FIRST 12 MONTHS**		
Lost to Follow-up	178/3417 (5.21%)	283/3411 (8.30%)

	BCG N=3417	Control N=3411
**VACCINATION IN THE 12 MONTHS FOLLOWING RANDOMISATION**		
Received Vaccines	2842/3417 (83.17%)	2705/3411 (79.30%)
Number of vaccines received during the 12 mo follow-up		
1	672/2842 (23.65%)	568/2705 (21.00%)
2	829/2842 (29.17%)	840/2705 (31.05%)
3	786/2842 (27.66%)	711/2705 (26.28%)
4	519/2842 (18.26%)	559/2705 (20.67%)
5	36/2842 (1.27%)	27/2705 (1.00%)
Days between Randomisation and First dose of any vaccine	2842, 169.1 (127.1)	2705, 162.9 (124.8)
**Type of Vaccine**		
AstraZeneca/Oxford (ChAdOx1, Covishield)	892/2842 (31.39%)	817/2705 (30.20%)
Diphtheria-tetanus vaccine	31/2842 (1.09%)	18/2705 (0.67%)
Diphtheria-tetanus-pertussis vaccine	48/2842 (1.69%)	43/2705 (1.59%)
Diphtheria-tetanus-pertussis-polio vaccine	7/2842 (0.25%)	1/2705 (0.04%)
Hepatitis A vaccine	4/2842 (0.14%)	2/2705 (0.07%)
Hepatitis A-hepatitis B vaccine	0/2842 (0.00%)	1/2705 (0.04%)
Hepatitis B vaccine	12/2842 (0.42%)	12/2705 (0.44%)
Influenza vaccine	189/2842 (6.65%)	169/2705 (6.25%)
Johnson & Johnson (Ad26.COV2.S)	47/2842 (1.65%)	45/2705 (1.66%)
MMR	5/2842 (0.18%)	9/2705 (0.33%)
MMR varicella	2/2842 (0.07%)	1/2705 (0.04%)
Meningococcal vaccine	0/2842 (0.00%)	1/2705 (0.04%)
Moderna (mRNA-1273)	61/2842 (2.15%)	77/2705 (2.85%)
Papillomavirus vaccine	9/2842 (0.32%)	2/2705 (0.07%)
Pfizer/BioNTech (BNT162b2, Comirnaty)	890/2842 (31.32%)	834/2705 (30.83%)
Pneumococcal vaccine	2/2842 (0.07%)	2/2705 (0.07%)
Rabies vaccine	1/2842 (0.04%)	1/2705 (0.04%)
Sinovac (CoronaVac)	640/2842 (22.52%)	667/2705 (24.66%)
Typhoid injected vaccine	0/2842 (0.00%)	1/2705 (0.04%)
Typhoid oral vaccine	0/2842 (0.00%)	1/2705 (0.04%)
Zoster live	1/2842 (0.04%)	0/2705 (0.00%)
Zoster nonlive	1/2842 (0.04%)	1/2705 (0.04%)

3. OUTCOMES

3.1 FEVER OR RESPIRATORY ILLNESS BY 12 MONTHS

Objective	Estimand
To determine if BCG vaccination compared with Control reduces the incidence of fever or respiratory illness irrespective of receiving any vaccine (including COVID-19 specific vaccine), over the 12 months following randomisation, in healthcare workers exposed to SARS-CoV-2.	Estimand 17.1<br>Population: ITT population<br>Outcome: fever or respiratory illness by 12 months<br>Interventions: BCG vs Control<br>Handling of Intercurrent events:<br>- any vaccine, including COVID-19 specific vaccine (**Treatment Policy strategy**)<br>Summary Measure: Adjusted difference in proportion of participants

Table 3.1 Estimand 17.1 – Incidence of fever or respiratory illness (ITT), Treatment policy Strategy

	BCG	Control	Difference (BCG-Control)	P value
	N=3417	N=3411		
Participants in itt_pop	3417	3411		
Fever or Resp Illness	2214/3417 (64.79%)	2029/3411 (59.48%)		
Censoring				
Censored- 12 months w/ no event	1008/3417 (29.50%)	1028/3411 (30.14%)		
Censored- incomplete data entry/drop-out from the study	195/3417 (5.71%)	354/3411 (10.38%)		
.	2214/3417 (64.79%)	2029/3411 (59.48%)		
Person years	1776	1783		
Event rate (per 100 person years)	121.63 95%CI(116.60 ; 126.87)	111.55 95%CI(106.75 ; 116.56)		
Estimated probability of Fever or Resp Illness				
- Unadjusted	0.670 95%CI(0.654 ; 0.686)	0.639 95%CI(0.622 ; 0.655)	0.031 95%CI(0.008 ; 0.055)	0.010
- Adjusted for stratification factors	0.668 95%CI(0.653 ; 0.682)	0.634 95%CI(0.618 ; 0.650)	0.034 95%CI(0.013 ; 0.055)	0.002

BCG: 38 participant(s) censored on randomisation date and 54 participant(s) had event starting on randomisation date. Control: 87 participant(s) censored on randomisation date and 40 participant(s) had event starting on randomisation date.

Model adjusted for : subgroup_1 subgroup_2_rand subgroup_3

Soft DB Lock on . Descriptive Part : generated on 20231101

Table completed on 20231101 - POP = itt_pop - Bootstrap = 1000

Objective	Estimand
To determine if BCG vaccination compared with Control reduces the incidence of fever or respiratory illness in the absence of any vaccine (including COVID-19 specific vaccine), over the 12 months following randomisation, in healthcare workers exposed to SARS-CoV-2.	**Estimand 17.2**<br>Population: ITT population<br>Outcome: fever or respiratory illness by 12 months<br>Interventions: BCG vs Control<br>Handling of Intercurrent events:<br>- any vaccine, including COVID-19 specific vaccine (**Hypothetical strategy**)<br>Summary Measure: Adjusted difference in proportion of participants

Table 3.2 Estimand 17.2 – Incidence of fever or respiratory illness (ITT), Hypothetical Strategy

	BCG	Control		Difference (BCG-Control)	P value
	N=3417	N=3411			
Participants in itt_pop	3417	3411			
Fever or Resp Illness	1447/3417 (42.35%)	1277/3411 (37.44%)			
Censoring					
Censored- Vaccination < 12 months	1654/3417 (48.41%)	1681/3411 (49.28%)			
Censored- 12 months w/ no event	151/3417 (4.42%)	150/3411 (4.40%)			
Censored- incomplete data entry/drop-out from the study	165/3417 (4.83%)	303/3411 (8.88%)			
.	1447/3417 (42.35%)	1277/3411 (37.44%)			
Person years	1107	1089			
Event rate (per 100 person years)	125.84 95%CI(119.40 ; 132.62)	113.62 95%CI(107.46 ; 120.13)			
Estimated probability of Fever or Resp Illness					
- Unadjusted	0.607 95%CI(0.584 ; 0.630)	0.570 95%CI(0.544 ; 0.596)		0.037 95%CI(0.002 ; 0.071)	0.036
- Adjusted for stratification factors	0.655 95%CI(0.632 ; 0.677)	0.613 95%CI(0.589 ; 0.637)		0.041 95%CI(0.014 ; 0.069)	0.003
Ciao					
UNADJUSTED- BCG=.6073641180992126 // Placebo=.5703817009925842 // RD=.0369824096560478					
ADJUSTED- BCG=.6545340418815613 // Placebo=.6132532358169556 // RD=.0412808209657669					

Figure 3.1 Incidence of Fever and respiratory illness (ITT) – 17.1 Treatment policy Strategy (a), 17.2 Hypothetical Strategy (b)

(a)

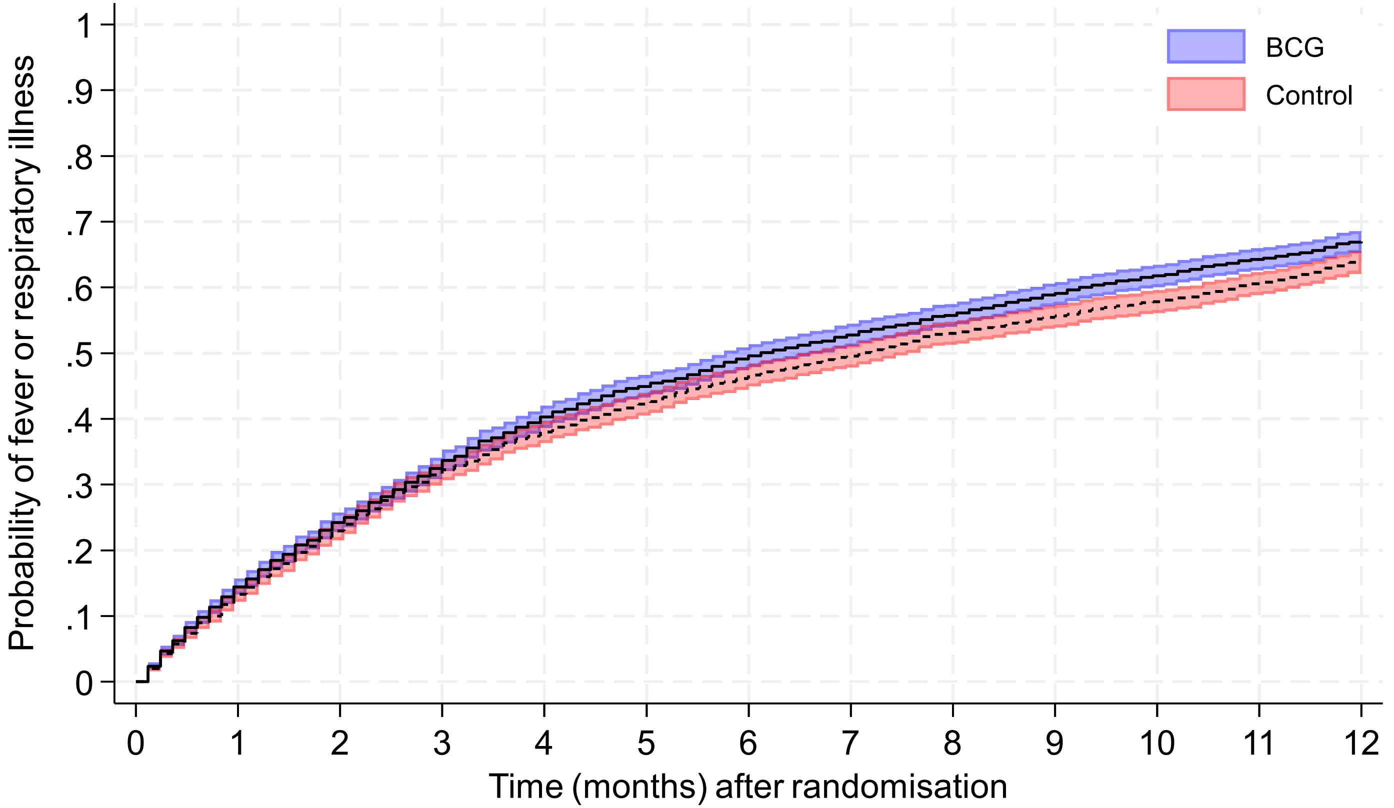

Number at risk

	0	1	2	3	4	5	6	7	8	9	10	11	12
BCG	3325	2815	2485	2212	1918	1759	1621	1480	1379	1281	1170	1091	1011
Control	3284	2785	2459	2186	1927	1781	1657	1497	1385	1308	1207	1126	1030

(b)

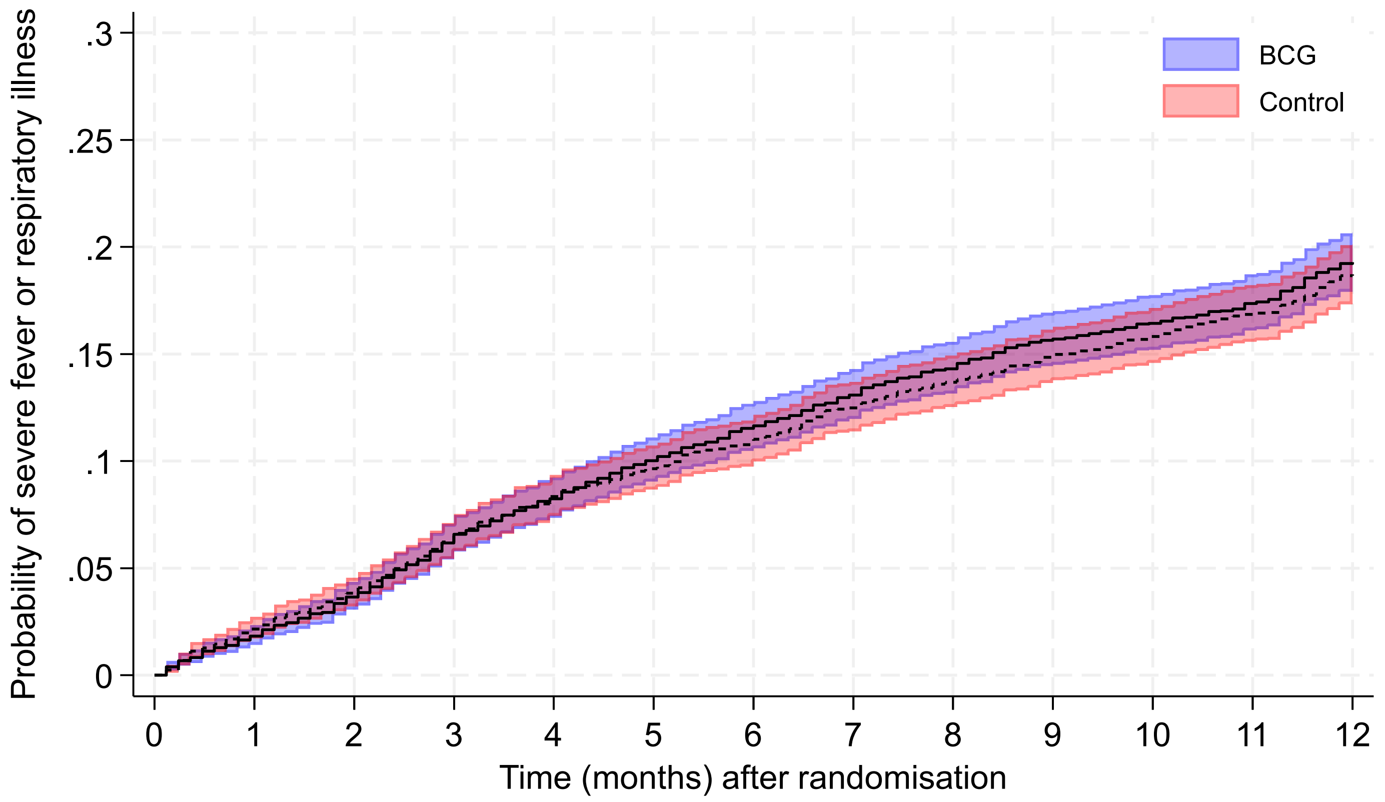

Number at risk

BCG	3368	3273	3202	3114	2996	2925	2872	2767	2719	2675	2607	2572	2511
Control	3320	3177	3105	3024	2889	2825	2785	2653	2604	2560	2477	2440	2375

3.2 SEVERE FEVER OR RESPIRATORY ILLNESS BY 12 MONTHS

Objective	Estimand
To determine if BCG vaccination compared with Control reduces the incidence of severe fever or respiratory illness irrespective of receiving any vaccine (including COVID-19 specific vaccine), over the 12 months following randomisation, in healthcare workers exposed to SARS-CoV-2.	**Estimand 18.1**<br><br>Population: ITT population<br>Outcome: severe fever or respiratory illness by 12 months<br>Interventions: BCG vs Control<br>Handling of Intercurrent events:<br>- any vaccine, including COVID-19 specific vaccine (**Treatment Policy strategy**)<br>Summary Measure: Adjusted difference in proportion of participants

Table 3.3 Estimand 18.1 – Incidence of Severe fever or respiratory illness (ITT), Treatment policy Strategy

Severe Fever or respiratory illness - Estimand 18.1	BCG	Control	Difference (BCG-Control)	P value
	N=3417	N=3411		
Participants in itt_pop	3417	3411		
Severe Fever or Resp Illness	637/3417 (18.64%)	588/3411 (17.24%)		
Censoring				
Censored- 12 months w/ no event	2509/3417 (73.43%)	2373/3411 (69.57%)		
Censored- incomplete data entry/drop-out from the study	271/3417 (7.93%)	450/3411 (13.19%)		
Person years	2885	2778		
Event rate (per 100 person years)	21.70 95%CI(20.06 ; 23.46)	21.02 95%CI(19.39 ; 22.80)		
Estimated probability of Fever or Resp Illness				
- Unadjusted	0.193 95%CI(0.179 ; 0.206)	0.187 95%CI(0.174 ; 0.201)	0.006 95%CI(-0.013 ; 0.025)	0.555
- Adjusted for stratification factors	0.194 95%CI(0.180 ; 0.207)	0.188 95%CI(0.174 ; 0.202)	0.006 95%CI(-0.013 ; 0.025)	0.554

BCG: 38 participant(s) censored on randomisation date and 11 participant(s) had event starting on randomisation date. Control: 87 participant(s) censored on randomisation date and 4 participant(s) had event starting on randomisation date.

Model adjusted for : subgroup_1 subgroup_2_rand subgroup_3

Soft DB Lock on . Descriptive Part : generated on 20231101

Table completed on 20231101 - POP = itt_pop - Bootstrap = 1000

Objective	Estimand
To determine if BCG vaccination compared with Control reduces the incidence of severe fever or respiratory illness in the absence of any vaccine (including COVID-19 specific vaccine), over the 12 months following randomisation, in healthcare workers exposed to SARS-CoV-2.	Estimand 18.2<br>Population: ITT population<br>Outcome: severe fever or respiratory illness by 12 months<br>Interventions: BCG vs Control<br>Handling of Intercurrent events:<br>- any vaccine, including COVID-19 specific vaccine (Hypothetical strategy)<br>Summary Measure: Adjusted difference in proportion of participants

Table 3.4 Estimand 18.2 – Incidence of Severe fever or respiratory illness (ITT), Hypothetical Strategy

	BCG	Control	Difference (BCG-Control)	P value
	N=3417	N=3411		
Participants in itt_pop	3417	3411		
Severe Fever or Resp Illness	376/3417 (11.00%)	352/3411 (10.32%)		
Censoring				
Censored- Vaccination < 12 months	2541/3417 (74.36%)	2436/3411 (71.42%)		
Censored- 12 months w/ no event	285/3417 (8.34%)	260/3411 (7.62%)		
Censored- incomplete data entry/drop-out from the study	215/3417 (6.29%)	363/3411 (10.64%)		
Person years	1591	1489		
Event rate (per 100 person years)	22.94 95%CI(20.71 ; 25.42)	23.38 95%CI(21.04 ; 25.97)		
Estimated probability of Fever or Resp Illness				
- Unadjusted	0.196 95%CI(0.176 ; 0.215)	0.192 95%CI(0.172 ; 0.213)	0.003 95%CI(-0.026 ; 0.032)	0.830
- Adjusted for stratification factors	0.199 95%CI(0.178 ; 0.221)	0.201 95%CI(0.179 ; 0.223)	-0.002 95%CI(-0.027 ; 0.024)	0.909

BCG: 41 participant(s) censored on randomisation date and 11 participant(s) had event starting on randomisation date. Control: 94 participant(s) censored on randomisation date and 4 participant(s) had event starting on randomisation date.

Model adjusted for : subgroup_1 subgroup_2_rand subgroup_3

Soft DB Lock on . Descriptive Part : generated on 20231101. Table completed on 20231101 - POP = itt_pop - Bootstrap = 1000

Figure 3.2 Incidence of Severe Fever and respiratory illness (ITT) – 18.1 Treatment policy Strategy (a), 18.2 Hypothetical Strategy (b)

(a)

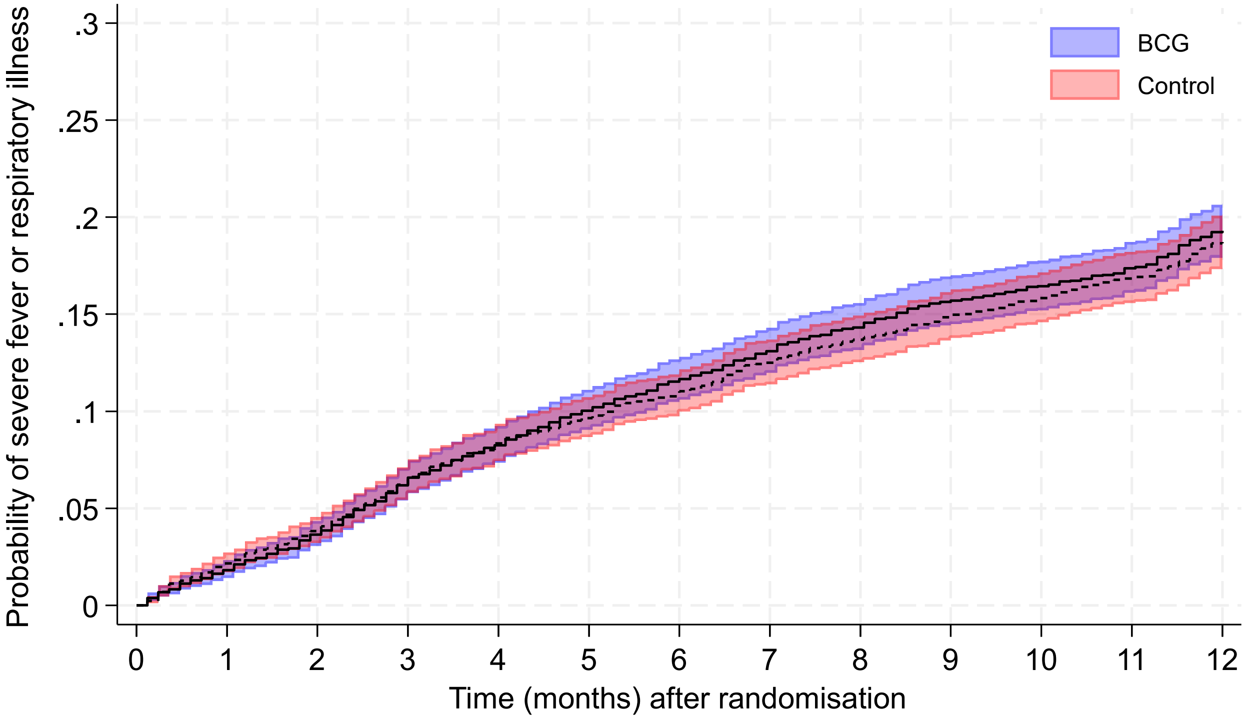

Number at risk

	0	1	2	3	4	5	6	7	8	9	10	11	12
BCG	3368	3273	3202	3114	2996	2925	2872	2767	2719	2675	2607	2572	2511
Control	3320	3177	3105	3024	2889	2825	2785	2653	2604	2560	2477	2440	2375

(b)

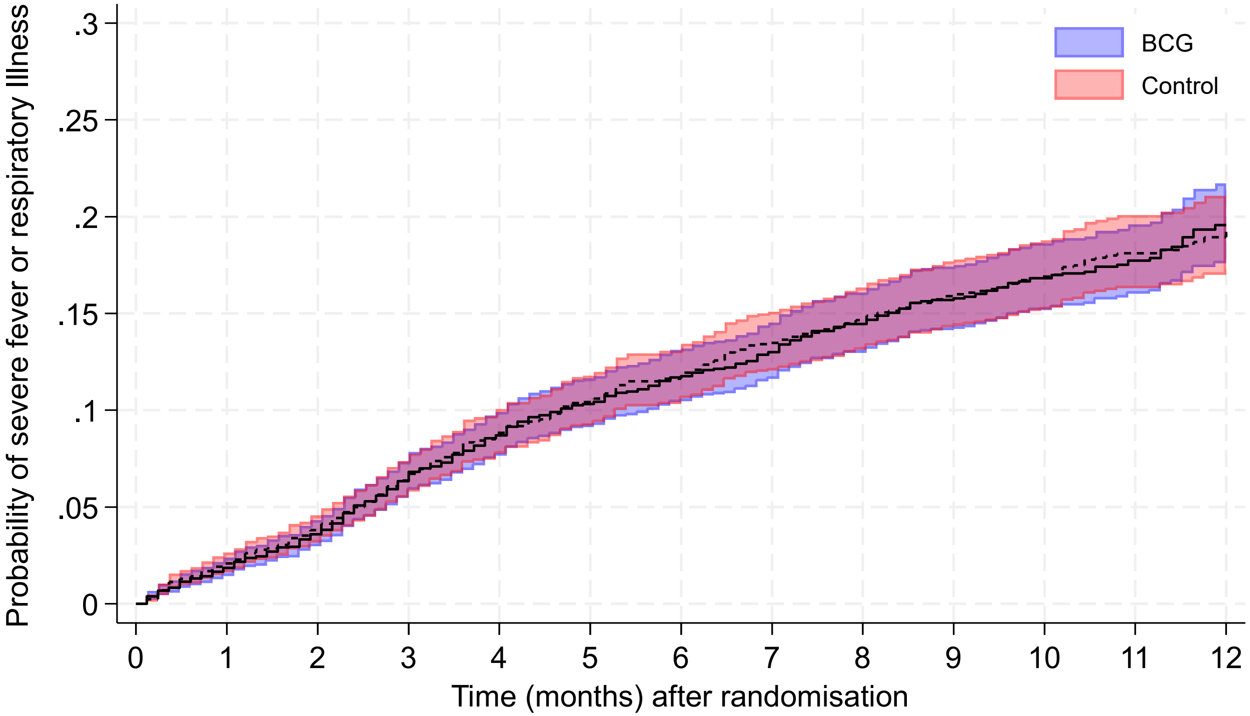

Number at risk

	0	1	2	3	4	5	6	7	8	9	10	11	12
BCG	3365	2986	2433	1978	1682	1537	1429	1285	1178	1101	1009	705	295
Control	3313	2903	2309	1901	1602	1454	1339	1165	1059	963	869	580	269

3.3 NUMBER OF EPISODES FEVER OR RESPIRATORY ILLNESS BY 12 MONTHS

Objective	Estimand
To determine if BCG vaccination compared with Control reduces the number of fever or respiratory illness irrespective of receiving any vaccine (including COVID-19 specific vaccine), over the 12 months following randomisation, in healthcare workers exposed to SARS-CoV-2.	Estimand 19.1<br><br>Population: ITT population<br>Outcome: number of fever or respiratory illness by 12 months<br>Interventions: BCG vs Control<br>Handling of Intercurrent events:<br>- any vaccine, including COVID-19 specific vaccine (**Treatment Policy strategy**)<br>Summary Measure: difference in the expected counts

Table 3.5 Estimand 19.1 – Number of episodes fever or respiratory illness (ITT), Treatment policy Strategy

	BCG	Control	Difference in Logs of Expected Counts (BCG - Control)	Incidence Rate Ratio (BCG v Control)	P value
	N=3417	N=3411			
Participants in itt_pop	3417	3411			
Number of episodes, Median(IQR)	1.0 (0.0-2.0)	1.0 (0.0-2.0)			
Number of episodes, Mean(SD)	1.3 (1.4)	1.2 (1.4)			
Number of episodes in subgroup with Fever or Resp Illness, Median(IQR)	2214, 2.0 (1.0-3.0)	2029, 2.0 (1.0-3.0)			
Number of episodes in subgroup with Fever or Resp Illness, Mean(SD)	2214, 2.0 (1.3)	2029, 2.0 (1.4)			
Comparison of Counts					
- Unadjusted			-0.021 95%CI(-0.063 ; 0.022)	0.980 95%CI(0.939 ; 1.022)	0.342
- Adjusted for stratification factors			-0.018 95%CI(-0.061 ; 0.025)	0.982 95%CI(0.941 ; 1.025)	0.406

Soft DB Lock on . Descriptive Part : generated on 20231101
ZIP Model adjusted for : subgroup_1 (age group), subgroup_2_rand (presence of comorbidity), subgroup_3 (geographical location)
Number of episodes fever or respiratory illness - Estimand 19.1 (Treatment Policy)
pvalue interaction arm##subgroup_1 = 0.5031
pvalue interaction arm##subgroup_2 = 0.0686
pvalue interaction arm##subgroup_3 = 0.3530
BCG: 38 participant(s) censored on randomisation date. Control: 87 participant(s) censored on randomisation date.

Objective	Estimand
To determine if BCG vaccination compared with Control reduces the number of fever or respiratory illness in the absence of any vaccine (including COVID-19 specific vaccine), over the 12 months following randomisation, in healthcare workers exposed to SARS-CoV-2.	Estimand 19.2<br><br>Population: ITT population<br>Outcome: number of fever or respiratory illness by 12 months<br>Interventions: BCG vs Control<br>Handling of Intercurrent events:<br>- any vaccine, including COVID-19 specific vaccine (**Hypothetical strategy**)<br>Summary Measure: difference in the expected counts

Table 3.6 Estimand 19.2 – Number of episodes fever or respiratory illness (ITT), Hypothetical Strategy

	BCG	Control	Difference in Logs of Expected Counts (BCG - Control)	Incidence Rate Ratio (BCG v Control)	P value
	N=3417	N=3411			
Participants in itt_pop	3417	3411			
Number of episodes, Median(IQR)	0.0 (0.0-1.0)	0.0 (0.0-1.0)			
Number of episodes, Mean(SD)	0.7 (1.0)	0.6 (1.0)			
Number of episodes in subgroup with Fever or Resp Illness, Median(IQR)	1447, 1.0 (1.0-2.0)	1277, 1.0 (1.0-2.0)			
Number of episodes in subgroup with Fever or Resp Illness, Mean(SD)	1447, 1.6 (0.9)	1277, 1.6 (1.1)			
Comparison of Counts					
- Unadjusted			-0.058 95%CI(-0.118 ; 0.002)	0.944 95%CI(0.889 ; 1.002)	0.057
- Adjusted for stratification factors			-0.055 95%CI(-0.114 ; 0.005)	0.947 95%CI(0.892 ; 1.005)	0.073

BCG: 41 participant(s) censored on randomisation date. Control: 94 participant(s) censored on randomisation date.

Soft DB Lock on . Descriptive Part : generated on 20231101

ZIP Model adjusted for : subgroup_1 (age group), subgroup_2_rand (presence of comorbidity), subgroup_3 (geographical location)

3.4 NUMBER DAYS UNABLE TO WORK DUE TO FEVER OR RESPIRATORY ILLNESS BY 12 MONTHS

3.4.1 NUMBER DAYS UNABLE TO WORK, TREATMENT POLICY STRATEGY

Objective	Estimand
To determine if BCG vaccination compared with Control reduces the number of days unable to work due to fever or respiratory illness irrespective of receiving any vaccine (including COVID-19 specific vaccine), over the 12 months following randomisation, in healthcare workers exposed to SARS-CoV-2.	**Estimand 20.1**<br><br>Population: ITT population<br>Outcome: number days unable to work due to fever or respiratory illness by 12 months<br>Interventions: BCG vs Control<br>Handling of Intercurrent events:<br>- any vaccine, including COVID-19 specific vaccine (**Treatment Policy strategy**)<br>Summary Measure: as for estimand 20.1

Table 3.7 Estimand 20.1 – Number days unable to work due to fever or respiratory illness (ITT), Treatment policy Strategy

	BCG	Control	Difference in Logs of Expected Counts (BCG - Control)	Incidence Rate Ratio (BCG v Control)	P value
	N=3417	N=3411			
Participants in itt_pop	3417	3411			
Number days unable to work, Median(IQR)	0.0 (0.0-2.0)	0.0 (0.0-1.0)			
Number days unable to work, Mean(SD)	1.8 (6.1)	1.6 (4.5)			
Number days unable to work in subgroup with Fever or Resp Illness, Median(IQR)	2214, 1.0 (0.0-4.0)	2029, 1.0 (0.0-4.0)			
Number days unable to work in subgroup with Fever or Resp Illness, Mean(SD)	2214, 2.7 (7.4)	2029, 2.7 (5.5)			
Comparison of Counts					
- Unadjusted			0.030 95%CI(-0.095 ; 0.155)	1.030 95%CI(0.910 ; 1.167)	0.638
- Adjusted for stratification factors			0.000 95%CI(-0.125 ; 0.125)	1.000 95%CI(0.883 ; 1.133)	0.996

Soft DB Lock on . Descriptive Part : generated on 20231101

Negative Binomial adjusted for : subgroup_1 (age group), subgroup_2_rand (presence of comorbidity), subgroup_3 (geographical location)

Number days unable to work due to fever or respiratory illness - Estimand 20.1 (Treatment Policy)

pvalue interaction arm##subgroup_1 = 0.0481 pvalue interaction arm##subgroup_2 = 0.0465 pvalue interaction arm##subgroup_3 = 0.7011

3.4.1.1 SUBGROUP ANALYSES BY AGE: NUMBER DAYS UNABLE TO WORK, TREATMENT POLICY STRATEGY

Table 3.7.1 Estimand 20.1 by AGE – Number days unable to work due to fever or respiratory illness (ITT), Treatment policy Strategy

	BCG	Control	Difference in Logs of Expected Counts (BCG - Placebo)	Incidence Rate Ratio (BCG v Placebo)	P value
	N=1592	N=1594			
Participants Subgroup 1 = <40 years old	1592	1594			
Number days unable to work, Median(IQR)	0.0 (0.0-2.0)	0.0 (0.0-2.0)			
Number days unable to work, Mean(SD)	1.7 (3.4)	1.6 (3.4)			
Number days unable to work in subgroup with Fever or Resp Illness N, Media	1133, 1.0 (0.0-3.0)	1041, 1.0 (0.0-4.0)			
Number days unable to work in subgroup with Fever or Resp Illness N, Mean(	1133, 2.4 (3.8)	1041, 2.5 (3.9)			
Comparison of Counts					
- Unadjusted			-0.007 95%CI(-0.170 ; 0.157)	0.993 95%CI(0.843 ; 1.170)	0.936
- Adjusted for stratification factors			-0.007 95%CI(-0.170 ; 0.157)	0.993 95%CI(0.843 ; 1.170)	0.936
Participants Subgroup 1 = 40-59 years old	1566	1557			
Number days unable to work, Median(IQR)	0.0 (0.0-1.0)	0.0 (0.0-1.0)			
Number days unable to work, Mean(SD)	2.0 (8.2)	1.6 (5.3)			
Number days unable to work in subgroup with Fever or Resp Illness N, Media	952, 1.0 (0.0-4.0)	867, 0.0 (0.0-4.0)			
Number days unable to work in subgroup with Fever or Resp Illness N Mean(S	952, 3.2 (10.4)	867, 2.9 (6.8)			
Comparison of Counts					
- Unadjusted			0.131 95%CI(-0.070 ; 0.331)	1.140 95%CI(0.933 ; 1.393)	0.201
- Adjusted for stratification factors			0.131 95%CI(-0.070 ; 0.331)	1.140 95%CI(0.933 ; 1.393)	0.201
Participants Subgroup 1 = 60+ years old	259	260			
Number days unable to work, Median(IQR)	0.0 (0.0-0.0)	0.0 (0.0-1.0)			
Number days unable to work, Mean(SD)	1.0 (2.7)	1.6 (5.1)			
Number days unable to work in subgroup with Fever or Resp Illness N, Media	129, 0.0 (0.0-3.0)	121, 1.0 (0.0-3.0)			
Number days unable to work in subgroup with Fever or Resp Illness N, Mean(	129, 2.1 (3.6)	121, 3.4 (7.0)			
Comparison of Counts					
- Unadjusted			-0.528 95%CI(-1.061 ; 0.005)	0.590 95%CI(0.346 ; 1.005)	0.052
- Adjusted for stratification factors			-0.528 95%CI(-1.061 ; 0.005)	0.590 95%CI(0.346 ; 1.005)	0.052

3.4.1.2 SUBGROUP ANALYSES BY PRESENCE OF COMORBIDITIES: NUMBER DAYS UNABLE TO WORK, TREATMENT POLICY STRATEGY

Table 3.7.2 Estimand 20.1 by PRESENCE OF COMORBIDITIES – Number days unable to work due to fever or respiratory illness (ITT), Treatment policy Strategy

	BCG	Control	Difference in Logs of Expected Counts (BCG - Placebo)	Incidence Rate Ratio (BCG v Placebo)	P value
	N=2804	N=2786			
Participants Subgroup 2 = Without Comorbidities	2804	2786			
Number days unable to work due to fever or respiratory illness, Median(IQR)	0.0 (0.0-2.0)	0.0 (0.0-1.0)			
Number days unable to work due to fever or respiratory illness, Mean(SD)	1.6 (4.3)	1.6 (4.5)			
Number days unable to work due to fever or respiratory illness in subgroup with	1807, 1.0 (0.0-4.0)	1662, 1.0 (0.0-4.0)			
Number days unable to work due to fever or respiratory illness in subgroup with	1807, 2.5 (5.1)	1662, 2.7 (5.6)			
Comparison of Counts					
- Unadjusted			-0.054 95%CI(-0.191 ; 0.083)	0.947 95%CI(0.826 ; 1.086)	0.439
- Adjusted for stratification factors			-0.063 95%CI(-0.200 ; 0.074)	0.939 95%CI(0.819 ; 1.077)	0.367
Participants Subgroup 2 = With Comorbidities	613	625			
Number days unable to work due to fever or respiratory illness, Median(IQR)	0.0 (0.0-2.0)	0.0 (0.0-1.0)			
Number days unable to work due to fever or respiratory illness, Mean(SD)	2.4 (11.1)	1.6 (4.1)			
Number days unable to work due to fever or respiratory illness in subgroup with Fev	407, 1.0 (0.0-4.0)	367, 0.0 (0.0-4.0)			
Number days unable to work due to fever or respiratory illness in subgroup with Fev	407, 3.7 (13.4)	367, 2.7 (5.1)			
Comparison of Counts					
- Unadjusted			0.345 95%CI(0.044 ; 0.645)	1.411 95%CI(1.045 ; 1.907)	0.025
- Adjusted for stratification factors			0.226 95%CI(-0.074 ; 0.526)	1.253 95%CI(0.929 ; 1.691)	0.140

3.4.2 NUMBER DAYS UNABLE TO WORK, HYPOTHETICAL STRATEGY

Objective	Estimand
To determine if BCG vaccination compared with Control reduces the number of days unable to work due to fever or respiratory illness in the absence of any vaccine (including COVID-19 specific vaccine), over the 12 months following randomisation, in healthcare workers exposed to SARS-CoV-2.	Estimand 20.1<br><br>Population: ITT population<br>Outcome: number days unable to work due to fever or respiratory illness by 12 months<br>Interventions: BCG vs Control<br>Handling of Intercurrent events:<br>- any vaccine, including COVID-19 specific vaccine (**Hypothetical strategy**)<br>Summary Measure: difference in the expected counts

Table 3.8 Estimand 20.2 – Number days unable to work due to fever or respiratory illness (ITT), Hypothetical Strategy

	BCG	Control	Difference in Logs of Expected Counts (BCG - Control)	Incidence Rate Ratio (BCG v Control)	P value
	N=3417	N=3411			
Participants in itt_pop	3417	3411			
Number days unable to work, Median(IQR)	0.0 (0.0-0.0)	0.0 (0.0-0.0)			
Number days unable to work, Mean(SD)	1.1 (5.5)	0.9 (3.0)			
Number days unable to work in subgroup with Fever or Resp Illness, Median(IQR)	1447, 1.0 (0.0-3.0)	1277, 1.0 (0.0-3.0)			
Number days unable to work in subgroup with Fever or Resp Illness, Mean(SD)	1447, 2.6 (8.3)	1277, 2.5 (4.4)			
Comparison of Counts					
- Unadjusted			0.094<br>95%CI(-0.058 ; 0.246)	1.099<br>95%CI(0.943 ; 1.279)	0.227
- Adjusted for stratification factors			0.054<br>95%CI(-0.096 ; 0.205)	1.056<br>95%CI(0.908 ; 1.228)	0.479

Soft DB Lock on . Descriptive Part : generated on 20231101

Negative Binomial adjusted for : subgroup_1 (age group), subgroup_2_rand (presence of comorbidity), subgroup_3 (geographical location)

Number days unable to work due to fever or respiratory illness - Estimand 20.2 (Hypothetical Strategy)

pvalue interaction arm##subgroup_1 = 0.0017

pvalue interaction arm##subgroup_2 = 0.1847

pvalue interaction arm##subgroup_3 = 0.4262

3.4.2.1 SUBGROUP ANALYSES BY AGE: NUMBER DAYS UNABLE TO WORK, HYPOTHETICAL STRATEGY

Table 3.8.1 Estimand 20.2 – Number days unable to work due to fever or respiratory illness (ITT), Hypothetical Strategy

	BCG	Control	Difference in Logs of Expected Counts (BCG - Placebo)	Incidence Rate Ratio (BCG v Placebo)	P value
	N=1592	N=1594			
Participants Subgroup 1 = <40 years old	1592	1594			
Number days unable to work due to fever or respiratory illness, Median(IQR)	0.0 (0.0-0.0)	0.0 (0.0-0.0)			
Number days unable to work due to fever or respiratory illness, Mean(SD)	1.0 (2.5)	1.0 (2.6)			
Number days unable to work due to fever or respiratory illness in subgroup wit	745, 1.0 (0.0-3.0)	642, 1.0 (0.0-4.0)			
Number days unable to work due to fever or respiratory illness in subgroup wit	745, 2.0 (3.3)	642, 2.4 (3.7)			
Comparison of Counts					
- Unadjusted			-0.040 95%CI(-0.239 ; 0.160)	0.961 95%CI(0.787 ; 1.173)	0.696
- Adjusted for stratification factors			-0.047 95%CI(-0.245 ; 0.151)	0.954 95%CI(0.782 ; 1.163)	0.639
Participants Subgroup 1 = 40-59 years old	1566	1557			
Number days unable to work due to fever or respiratory illness, Median(IQR)	0.0 (0.0-0.0)	0.0 (0.0-0.0)			
Number days unable to work due to fever or respiratory illness, Mean(SD)	1.3 (7.7)	0.9 (3.0)			
Number days unable to work due to fever or respiratory illness in subgroup wit	618, 1.0 (0.0-4.0)	561, 0.0 (0.0-3.0)			
Number days unable to work due to fever or respiratory illness in subgroup wit	618, 3.3 (12.1)	561, 2.5 (4.5)			
			0.351 95%CI(0.109 ; 0.593)	1.420 95%CI(1.115 ; 1.809)	0.004
			0.278 95%CI(0.039 ; 0.517)	1.320 95%CI(1.040 ; 1.676)	0.023
Participants Subgroup 1 = 60+ years old	259	260			
Number days unable to work due to fever or respiratory illness, Median(IQR)	0.0 (0.0-0.0)	0.0 (0.0-0.0)			
Number days unable to work due to fever or respiratory illness, Mean(SD)	0.6 (2.0)	1.1 (4.3)			
Number days unable to work due to fever or respiratory illness in subgroup wit	84, 0.0 (0.0-2.0)	74, 1.0 (0.0-4.0)			
Number days unable to work due to fever or respiratory illness in subgroup wit	84, 1.8 (3.2)	74, 3.9 (7.4)			
			-0.935 95%CI(-1.624 ; -0.246)	0.393 95%CI(0.197 ; 0.782)	0.008
			-0.609 95%CI(-1.310 ; 0.092)	0.544 95%CI(0.270 ; 1.096)	0.088

3.5 NUMBER DAYS CONFINED TO BED DUE FEVER OR RESPIRATORY ILLNESS BY 12 MONTHS

Objective	Estimand
To determine if BCG vaccination compared with Control reduces the number of days confined to bed due to fever or respiratory illness irrespective of receiving any vaccine (including COVID-19 specific vaccine), over the 12 months following randomisation, in healthcare workers exposed to SARS-CoV-2.	Estimand 21.1<br><br>Population: as for estimand 21.1<br>Outcome: as for estimand 21.1<br>Interventions: as for estimand 21.1<br>Handling of Intercurrent events:<br>- any vaccine, including COVID-19 specific vaccine (Treatment Policy strategy)<br>Summary Measure: as for estimand 21.1

Table 3.9 Estimand 21.1 – Number days confined to bed due to fever or respiratory illness (ITT), Treatment policy Strategy

	BCG	Control	Difference in Logs of Expected Counts (BCG - Control)	Incidence Rate Ratio (BCG v Control)	P value
	N=3417	N=3411			
Participants in itt_pop	3417	3411			
Number days confined to bed due fever or respiratory illness, Median(IQR)	0.0 (0.0-0.0)	0.0 (0.0-0.0)			
Number days confined to bed due fever or respiratory illness, Mean(SD)	0.5 (2.6)	0.4 (1.5)			
Number days confined to bed due fever or respiratory illness in subgroup with Fever or	2214, 0.0 (0.0-0.0)	2029, 0.0 (0.0-0.0)			
Number days confined to bed due fever or respiratory illness in subgroup with Fever or	2214, 0.7 (3.2)	2029, 0.7 (1.9)			
Comparison of Counts					
- Unadjusted			-0.049 95%CI(-0.233 ; 0.135)	0.952 95%CI(0.792 ; 1.144)	0.600
- Adjusted for stratification factors			-0.055 95%CI(-0.238 ; 0.128)	0.946 95%CI(0.788 ; 1.136)	0.554

Soft DB Lock on . Descriptive Part : generated on 20231101
Zero-Inflated Negative Binomial Regression adjusted for : subgroup_1 (age group), subgroup_2_rand (presence of comorbidity), subgroup_3 (geographical location)
Number days confined to bed due fever or respiratory illness - Estimand 21.1 (Treatment Policy)
pvalue interaction arm##subgroup_1 = 0.0918
pvalue interaction arm##subgroup_2 = 0.8069
pvalue interaction arm##subgroup_3 = 0.7476
BCG: 38 participant(s) censored on randomisation date. Placebo: 87 participant(s) censored on randomisation date

Objective	Estimand
To determine if BCG vaccination compared with Control reduces the number of days confined to bed due to fever or respiratory illness in the absence of any vaccine (including COVID-19 specific vaccine), over the 12 months following randomisation, in healthcare workers exposed to SARS-CoV-2.	Estimand 21.2<br><br>Population: ITT population<br>Outcome: number days confined to bed due fever or respiratory illness by 12 months<br>Interventions: BCG vs Control<br>Handling of Intercurrent events:<br>- any vaccine, including COVID-19 specific vaccine (Hypothetical strategy)<br>Summary Measure: difference in the expected counts

Table 3.10 Estimand 21.2 – Number days confined to bed due to fever or respiratory illness (ITT), Hypothetical Strategy

	BCG	Control		Difference in Logs of Expected Counts (BCG - Placebo)	Incidence Rate Ratio (BCG v Placebo)	P value
	N=3417	N=3411				
Participants in itt_pop	3417	3411				
Number days confined to bed due fever or respiratory illness, Median(IQR)	0.0 (0.0-0.0)	0.0 (0.0-0.0)				
Number days confined to bed due fever or respiratory illness, Mean(SD)	0.3 (2.3)	0.3 (1.2)				
Number days confined to bed due fever or respiratory illness in subgroup with Fe	1447, 0.0 (0.0-0.0)	1277, 0.0 (0.0-0.0)				
Number days confined to bed due fever or respiratory illness in subgroup with Fe	1447, 0.7 (3.5)	1277, 0.7 (1.9)				
Comparison of Counts						
- Unadjusted				-0.072 95%CI(-0.297 ; 0.153)	0.930 95%CI(0.743 ; 1.165)	0.530
- Adjusted for stratification factors				-0.090 95%CI(-0.307 ; 0.127)	0.914 95%CI(0.736 ; 1.135)	0.416
Soft DB Lock on . Descriptive Part : generated on 20231101						
Zero-Inflated Negative Binomial Regression adjusted for : subgroup_1 (age group), subgroup_2_rand (presence of comorbidity), subgroup_3 (geographical location)						
Number days confined to bed due fever or respiratory illness - Estimand 21.2 (Hypotetical Strategy)						
pvalue interaction arm##subgroup_1 = 0.1632						
pvalue interaction arm##subgroup_2 = 0.8169						
pvalue interaction arm##subgroup_3 = 0.9861						
BCG: 41 participant(s) censored on randomisation date. Placebo: 94 participant(s) censored on randomisation date						

3.6 NUMBER DAYS WITH SYMPTOMS DUE FEVER OR RESPIRATORY ILLNESS BY 12 MONTHS

3.6.1 NUMBER OF DAYS WITH SYMPTOMS, TREATMENT POLICY STRATEGY

Objective	Estimand
To determine if BCG vaccination compared with Control reduces the number of days with symptoms due to fever or respiratory illness irrespective of receiving any vaccine (including COVID-19 specific vaccine), over the 12 months following randomisation, in healthcare workers exposed to SARS-CoV-2.	Estimand 22.1<br><br>Population: ITT population<br>Outcome: number days with symptoms due fever or respiratory illness by 12 months<br>Interventions: BCG vs Control<br>Handling of Intercurrent events:<br>- any vaccine, including COVID-19 specific vaccine (Treatment Policy strategy)<br>Summary Measure: as for estimand 22.1

Table 3.11 Estimand 22.1 – Number days with symptoms due to fever or respiratory illness (ITT), Treatment policy Strategy

Number of days with symptoms - Estimand 22.1 (Treatment Policy)					
	BCG	Control	Difference in Logs of Expected Counts (BCG - Placebo)	Incidence Rate Ratio (BCG v Placebo)	P value
	N=3417	N=3411			
Participants in itt_pop	3417	3411			
Number of days with symptoms, Median(IQR)	5.0 (0.0-15.0)	4.0 (0.0-14.0)			
Number of days with symptoms, Mean(SD)	11.1 (19.6)	10.5 (19.4)			
Number of days with symptoms in subgroup with Fever or Resp Illness, Median(IQR)	2214, 11.0 (5.0-21.0)	2029, 11.0 (6.0-22.0)			
Number of days with symptoms in subgroup with Fever or Resp Illness, Mean(SD)	2214, 17.1 (22.2)	2029, 17.7 (22.6)			
Comparison of Counts					
- Unadjusted			-0.054 95%CI(-0.110 ; 0.001)	0.947 95%CI(0.896 ; 1.001)	0.054
- Adjusted for stratification factors			-0.059 95%CI(-0.112 ; -0.005)	0.943 95%CI(0.894 ; 0.995)	0.032
Soft DB Lock on . Descriptive Part : generated on 20231101					
Zero-Inflated Negative Binomial Regression adjusted for : subgroup_1 (age group), subgroup_2_rand (presence of comorbidity), subgroup_3 (geographical location)					
Number of days with symptoms - Estimand 22.1 (Treatment Policy)					
pvalue interaction arm##subgroup_1 = 0.8752					
pvalue interaction arm##subgroup_2 = 0.0035					
pvalue interaction arm##subgroup_3 = 0.0093					
BCG: 38 participant(s) censored on randomisation date. Placebo: 87 participant(s) censored on randomisation date					

3.6.1.1 SUBGROUP ANALYSES BY PRESENCE OF COMORBIDITIES: NUMBER DAYS WITH SYMPTOMS, TREATMENT POLICY STRATEGY

Table 3.11.1 Estimand 22.1 by PRESENCE OF COMORBIDITIES – Number days with symptoms due to fever or respiratory illness (ITT), Treatment policy Strategy

	BCG	Control	Difference in Logs of Expected Counts (BCG - Placebo)	Incidence Rate Ratio (BCG v Placebo)	P value
	N=2804	N=2786			
Participants Subgroup 2 = Without Comorbidities	2804	2786			
Number of days with symptoms, Median(IQR)	5.0 (0.0-14.0)	4.0 (0.0-14.0)			
Number of days with symptoms , Mean(SD)	10.4 (17.4)	10.3 (18.7)			
Number of days with symptoms in subgroup with Fever or Resp Illness N, Median(IQR)	1807, 11.0 (5.0-20.0)	1662, 11.0 (6.0-22.0)			
Number of days with symptoms in subgroup with Fever or Resp Illness N, Mean(SD)	1807, 16.2 (19.5)	1662, 17.3 (21.5)			
Comparison of Counts					
- Unadjusted			-0.095 95%CI(-0.155 ; -0.035)	0.909 95%CI(0.857 ; 0.966)	0.002
- Adjusted for stratification factors			-0.096 95%CI(-0.154 ; -0.038)	0.908 95%CI(0.857 ; 0.963)	0.001
Participants Subgroup 2 = With Comorbidities	613	625			
Number of days with symptoms, Median(IQR)	6.0 (0.0-17.0)	4.0 (0.0-13.0)			
Number of days with symptoms , Mean(SD)	14.2 (27.4)	11.3 (22.6)			
Number of days with symptoms in subgroup with Fever or Resp Illness N, Median(IQR)	407, 13.0 (6.0-25.0)	367, 11.0 (5.0-22.0)			
Number of days with symptoms in subgroup with Fever or Resp Illness N, Mean(SD)	407, 21.4 (31.3)	367, 19.3 (26.8)			
Comparison of Counts					
- Unadjusted			0.094 95%CI(-0.045 ; 0.233)	1.098 95%CI(0.956 ; 1.262)	0.186
- Adjusted for stratification factors			0.092 95%CI(-0.045 ; 0.229)	1.097 95%CI(0.956 ; 1.258)	0.186

3.6.1.2 SUBGROUP ANALYSES BY GEOGRAPHICAL AREA: NUMBER DAYS WITH SYMPTOMS, TREATMENT POLICY STRATEGY

Table 3.11.2 Estimand 22.1 by GEOGRAPHICAL AREA – Number days with symptoms due to fever or respiratory illness (ITT), Treatment policy Strategy

	BCG	Control	Difference in Logs of Expected Counts (BCG - Placebo)	Incidence Rate Ratio (BCG v Placebo)	P value
	N=1418	N=1422			
Participants Subgroup 3 = Australia-Stage 1	1418	1422			
Number of days with symptoms, Median(IQR)	2.0 (0.0-9.0)	0.0 (0.0-8.0)			
Number of days with symptoms, Mean(SD)	6.4 (10.7)	6.2 (12.2)			
Number of days with symptoms in subgroup with Fever or Resp Illness N, Median(I	776, 8.0 (4.0-15.0)	684, 9.0 (4.0-16.0)			
Number of days with symptoms in subgroup with Fever or Resp Illness N, Mean(SD	776, 11.7 (12.1)	684, 13.0 (14.9)			
Comparison of Counts					
- Unadjusted			-0.157 95%CI(-0.248 ; -0.066)	0.855 95%CI(0.780 ; 0.936)	0.001
- Adjusted for stratification factors			-0.159 95%CI(-0.250 ; -0.068)	0.853 95%CI(0.779 ; 0.934)	0.001
Participants Subgroup 3 = Australia-Stage 2	216	206			
Number of days with symptoms, Median(IQR)	5.0 (0.0-13.0)	2.0 (0.0-9.0)			
Number of days with symptoms, Mean(SD)	8.4 (11.7)	7.4 (13.5)			
Number of days with symptoms in subgroup with Fever or Resp Illness N, Median(IQR	149, 8.0 (4.0-16.0)	116, 7.5 (4.0-18.0)			
Number of days with symptoms in subgroup with Fever or Resp Illness N, Mean(SD)	149, 12.1 (12.4)	116, 13.1 (15.8)			
Comparison of Counts					
- Unadjusted			-0.090 95%CI(-0.306 ; 0.126)	0.914 95%CI(0.736 ; 1.134)	0.414
- Adjusted for stratification factors			-0.083 95%CI(-0.301 ; 0.135)	0.921 95%CI(0.740 ; 1.145)	0.457
Participants Subgroup 3 = Europe	498	500			
Number of days with symptoms, Median(IQR)	4.0 (0.0-14.0)	4.0 (0.0-13.0)			
Number of days with symptoms, Mean(SD)	12.3 (29.1)	10.1 (17.4)			
Number of days with symptoms in subgroup with Fever or Resp Illness N, Median(IQR	317, 11.0 (5.0-21.0)	306, 10.0 (5.0-20.0)			
Number of days with symptoms in subgroup with Fever or Resp Illness N, Mean(SD)	317, 19.3 (34.5)	306, 16.5 (19.7)			
Comparison of Counts					
- Unadjusted			0.144 95%CI(-0.012 ; 0.301)	1.155 95%CI(0.988 ; 1.351)	0.071
- Adjusted for stratification factors			0.102 95%CI(-0.055 ; 0.258)	1.107 95%CI(0.946 ; 1.295)	0.205

Participants Subgroup 3 = South America	1285	1283				
Number of days with symptoms, Median(IQR)	10.0 (2.0-23.0)	9.0 (0.0-22.0)				
Number of days with symptoms, Mean(SD)	16.3 (22.3)	16.0 (25.3)				
Number of days with symptoms in subgroup with Fever or Resp Illness N, Median(IQR	972, 15.0 (8.0-27.0)	923, 14.0 (8.0-28.0)				
Number of days with symptoms in subgroup with Fever or Resp Illness N, Mean(SD)	972, 21.5 (23.3)	923, 22.2 (27.5)				
Comparison of Counts						
- Unadjusted				-0.035 95%CI(-0.113 ; 0.043)	0.965 95%CI(0.893 ; 1.043)	0.375
- Adjusted for stratification factors				-0.039 95%CI(-0.116 ; 0.039)	0.962 95%CI(0.891 ; 1.040)	0.330

3.6.2 NUMBER OF DAYS WITH SYMPTOMS, HYPOTHETICAL STRATEGY

Objective	Estimand
To determine if BCG vaccination compared with Control reduces the number of days with symptoms due to fever or respiratory illness in the absence of any vaccine (including COVID-19 specific vaccine), over the 12 months following randomisation, in healthcare workers exposed to SARS-CoV-2.	Estimand 22.2<br><br>Population: ITT population<br>Outcome: number days with symptoms due fever or respiratory illness by 12 months<br>Interventions: BCG vs Control<br>Handling of Intercurrent events:<br>- any vaccine, including COVID-19 specific vaccine (Hypothetical strategy)<br>Summary Measure: difference in the expected counts

Table 3.12 Estimand 22.2 – Number days with symptoms due to fever or respiratory illness (ITT), Hypothetical Strategy

	BCG	Control		Difference in Logs of Expected Counts (BCG - Placebo)	Incidence Rate Ratio (BCG v Placebo)	P value
	N=3417	N=3411				
Participants in itt_pop	3417	3411				
Number of days with symptoms, Median(IQR)	0.0 (0.0-7.0)	0.0 (0.0-6.0)				
Number of days with symptoms, Mean(SD)	5.3 (13.7)	4.8 (11.9)				
Number of days with symptoms in subgroup with Fever or Resp Illness, Median(	1447, 8.0 (4.0-15.0)	1277, 8.0 (4.0-15.0)				
Number of days with symptoms in subgroup with Fever or Resp Illness, Mean(SI	1447, 12.4 (18.8)	1277, 12.9 (16.5)				
Comparison of Counts						
- Unadjusted				-0.032 95%CI(-0.116 ; 0.051)	0.968 95%CI(0.891 ; 1.053)	0.450
- Adjusted for stratification factors				-0.061 95%CI(-0.131 ; 0.009)	0.941 95%CI(0.878 ; 1.009)	0.088
Soft DB Lock on . Descriptive Part : generated on 20231101						
Zero-Inflated Negative Binomial Regression adjusted for : subgroup_1 (age group), subgroup_2_rand (presence of comorbidity), subgroup_3 (geographical location)						
Number of days with symptoms - Estimand 22.2 (Hypotetical Strategy)						
pvalue interaction arm##subgroup_1 = 0.5588						
pvalue interaction arm##subgroup_2 = 0.0211						
pvalue interaction arm##subgroup_3 = 0.0239						
BCG: 41 participant(s) censored on randomisation date. Placebo: 94 participant(s) censored on randomisation date						

3.6.2.1 SUBGROUP ANALYSES BY PRESENCE OF COMORBIDITIES: NUMBER DAYS WITH SYMPTOMS, HYPOTHETICAL STRATEGY

Table 3.12.1 Estimand 22.2 by PRESENCE OF COMORBIDITIES – Number days with symptoms due to fever or respiratory illness (ITT), hypothetical Strategy

	BCG	Control	Difference in Logs of Expected Counts (BCG - Placebo)	Incidence Rate Ratio (BCG v Placebo)	P value
	N=2804	N=2786			
Participants Subgroup 2 = Without Comorbidities	2804	2786			
Number of days with symptoms due fever or respiratory illness, Median(IQR)	0.0 (0.0-7.0)	0.0 (0.0-6.0)			
Number of days with symptoms due fever or respiratory illness, Mean(SD)	5.1 (13.1)	4.9 (12.0)			
Number of days with symptoms due fever or respiratory illness in subgroup v	1191, 8.0 (4.0-14.0)	1063, 9.0 (5.0-15.0)			
Number of days with symptoms due fever or respiratory illness in subgroup v	1191, 12.1 (17.8)	1063, 12.9 (16.5)			
Comparison of Counts					
- Unadjusted			-0.091 95%CI(-0.181 ; -0.001)	0.913 95%CI(0.835 ; 0.999)	0.048
- Adjusted for stratification factors			-0.098 95%CI(-0.173 ; -0.023)	0.907 95%CI(0.841 ; 0.978)	0.011
Participants Subgroup 2 = With Comorbidities	613	625			
Number of days with symptoms due fever or respiratory illness, Median(IQR)	0.0 (0.0-6.0)	0.0 (0.0-4.0)			
Number of days with symptoms due fever or respiratory illness, Mean(SD)	5.9 (16.1)	4.4 (11.3)			
Number of days with symptoms due fever or respiratory illness in subgroup v	256, 8.0 (4.0-16.0)	214, 8.0 (4.0-16.0)			
Number of days with symptoms due fever or respiratory illness in subgroup v	256, 14.1 (22.6)	214, 12.8 (16.3)			
Comparison of Counts					
- Unadjusted			0.185 95%CI(-0.029 ; 0.400)	1.204 95%CI(0.971 ; 1.492)	0.090
- Adjusted for stratification factors			0.081 95%CI(-0.100 ; 0.262)	1.085 95%CI(0.905 ; 1.300)	0.379

Table 3.12.2 Estimand 22.2 by PRESENCE OF GEOGRAPHICAL AREA – Number days with symptoms due to fever or respiratory illness (ITT), hypothetical Strategy

	BCG	Control	Difference in Logs of Expected Counts (BCG - Control)	Incidence Rate Ratio (BCG v Control)	P value
Participants Subgroup 3 = Australia-Stage 1	1418	1422			
Number of days with symptoms , Median(IQR)	1.0 (0.0-8.0)	0.0 (0.0-8.0)			
Number of days with symptoms , Mean(SD)	6.0 (10.5)	5.9 (12.0)			
Number of days with symptoms in subgroup with Fever or Resp Illness N, Median(IQR)	733, 8.0 (4.0-14.0)	654, 9.0 (4.0-16.0)			
Number of days with symptoms in subgroup with Fever or Resp Illness N, Mean(SD)	733, 11.5 (12.2)	654, 12.8 (15.0)			
Comparison of Counts					
- Unadjusted			-0.148 95%CI(-0.242 ; -0.053)	0.863 95%CI(0.785 ; 0.949)	0.002
- Adjusted for stratification factors			-0.149 95%CI(-0.244 ; -0.055)	0.861 95%CI(0.784 ; 0.947)	0.002
Participants Subgroup 3 = Australia-Stage 2	216	206			
Number of days with symptoms , Median(IQR)	2.0 (0.0-8.0)	0.0 (0.0-5.0)			
Number of days with symptoms , Mean(SD)	5.1 (8.0)	3.9 (7.7)			
Number of days with symptoms in subgroup with Fever or Resp Illness N, Median(IQR)	123, 6.0 (4.0-12.0)	90, 5.0 (3.0-12.0)			
Number of days with symptoms in subgroup with Fever or Resp Illness N, Mean(SD)	123, 8.9 (8.9)	90, 9.0 (9.6)			
Comparison of Counts					
- Unadjusted			0.112 95%CI(-0.147 ; 0.371)	1.119 95%CI(0.863 ; 1.450)	0.397
- Adjusted for stratification factors			0.105 95%CI(-0.154 ; 0.363)	1.110 95%CI(0.857 ; 1.438)	0.428
Participants Subgroup 3 = Europe	498	500			
Number of days with symptoms , Median(IQR)	0.0 (0.0-4.0)	0.0 (0.0-4.0)			
Number of days with symptoms , Mean(SD)	5.8 (22.1)	4.9 (11.6)			
Number of days with symptoms in subgroup with Fever or Resp Illness N, Median(IQR)	193, 6.0 (3.0-16.0)	176, 8.5 (3.5-16.0)			
Number of days with symptoms in subgroup with Fever or Resp Illness N, Mean(SD)	193, 15.0 (33.6)	176, 13.8 (16.2)			
Comparison of Counts					

	BCG	Control	Difference in Logs of Expected Counts (BCG - Control)	Incidence Rate Ratio (BCG v Control)	P value
- Unadjusted			0.184 95%CI(-0.042 ; 0.409)	1.202 95%CI(0.959 ; 1.506)	0.110
- Adjusted for stratification factors			0.093 95%CI(-0.127 ; 0.313)	1.097 95%CI(0.880 ; 1.368)	0.408
Participants Subgroup 3 = South America	1285	1283			
Number of days with symptoms , Median(IQR)	0.0 (0.0-5.0)	0.0 (0.0-4.0)			
Number of days with symptoms , Mean(SD)	4.3 (13.2)	3.8 (12.2)			
Number of days with symptoms in subgroup with Fever or Resp Illness N, Median(IQR)	398, 9.0 (6.0-16.0)	357, 9.0 (5.0-15.0)			
Number of days with symptoms in subgroup with Fever or Resp Illness N, Mean(SD)	398, 13.9 (20.7)	357, 13.7 (20.1)			
Comparison of Counts					
- Unadjusted			-0.036 95%CI(-0.161 ; 0.090)	0.965 95%CI(0.851 ; 1.094)	0.575
- Adjusted for stratification factors			-0.029 95%CI(-0.154 ; 0.096)	0.972 95%CI(0.857 ; 1.101)	0.651

3.7 PNEUMONIA WITHIN A FEBRILE OR RESPIRATORY ILLNESS BY 12 MONTHS

Objective	Estimand
To determine if BCG vaccination compared with Control reduces the incidence of pneumonia irrespective of receiving any other vaccine (including COVID-19 specific vaccine), over the 12 months following randomisation, in healthcare workers exposed to SARS-CoV-2.	Estimand 23.1<br><br>Population: ITT population<br>Outcome: pneumonia by 12 months<br>Interventions: BCG vs Control<br>Handling of Intercurrent events:<br>- any vaccine, including COVID-19 specific vaccine (Treatment policy)<br>Summary Measure: as for estimand 23.1

Table 3.13 Estimand 23.1 – Incidence of pneumonia (ITT), Treatment policy Strategy

	BCG	Control	Difference (BCG-Control)	P value
	N=3417	N=3411		
Participants in itt_pop	3417	3411		
PNEUMONIA	22/3417 (0.64%)	21/3411 (0.62%)		
Censoring				
Censored- 12 months w/ no event	3088/3417 (90.37%)	2888/3411 (84.67%)		
Censored- incomplete data entry/drop-out from the study	307/3417 (8.98%)	502/3411 (14.72%)		
.	22/3417 (0.64%)	21/3411 (0.62%)		
Person years	3219	3074		
Event rate (per 100 person years)	0.68 95%CI(0.45 ; 1.04)	0.68 95%CI(0.45 ; 1.05)		
Estimated probability of Fever or Resp Illness				
- Unadjusted	0.007 95%CI(0.004 ; 0.009)	0.007 95%CI(0.004 ; 0.010)	0.000 95%CI(-0.004 ; 0.004)	1.000
- Adjusted for stratification factors	0.007 95%CI(0.004 ; 0.009)	0.006 95%CI(0.004 ; 0.009)	0.000 95%CI(-0.004 ; 0.004)	0.909
BCG: 38 participant(s) censored on randomisation date and . participant(s) had event starting on randomisation date. Control: 87 participant(s) censored on randomisation date and . participant(s) had event starting on randomisation date.				
Model adjusted for : subgroup_1 subgroup_2_rand subgroup_3				
Soft DB Lock on . Descriptive Part : generated on 20231101				
Table completed on 20231101 - POP = itt_pop - Bootstrap = 1000				

Objective	Estimand
To determine if BCG vaccination compared with Control reduces the incidence of pneumonia in the absence of any vaccine, including COVID-19 specific vaccine, measured over 12 months following randomisation in healthcare exposed to SARS-CoV-2.	**Estimand 23.2**<br><br><u>Population</u>: ITT population<br><u>Outcome</u>: pneumonia by 12 months<br><u>Interventions</u>: BCG vs Control<br><u>Handling of Intercurrent events</u>:<br>- any vaccine, including COVID-19 specific vaccine (**Hypothetical strategy**)<br><u>Summary Measure</u>: Adjusted difference in proportion of participants

Table 3.14 Estimand 23.2 – Incidence of pneumonia (ITT), Hypothetical Strategy

	BCG	Control	Difference (BCG-Control)	P value
	N=3417	N=3411		
Participants in itt_pop	3417	3411		
PNEUMONIA	9/3417 (0.26%)	13/3411 (0.38%)		
Censoring				
Censored- Vaccination < 12 months	2820/3417 (82.53%)	2670/3411 (78.28%)		
Censored- 12 months w/ no event	346/3417 (10.13%)	324/3411 (9.50%)		
Censored- incomplete data entry/drop-out from the study	242/3417 (7.08%)	404/3411 (11.84%)		
.	9/3417 (0.26%)	13/3411 (0.38%)		
Person years	1735	1629		
Event rate (per 100 person years)	0.52 95%CI(0.27 ; 1.00)	0.80 95%CI(0.46 ; 1.37)		
Estimated probability of Fever or Resp Illness				
- Unadjusted	0.004 95%CI(0.001 ; 0.007)	0.007 95%CI(0.002 ; 0.013)	-0.003 95%CI(-0.009 ; 0.003)	0.288
- Adjusted for stratification factors	0.008 95%CI(-0.001 ; 0.016)	0.011 95%CI(0.002 ; 0.020)	-0.004 95%CI(-0.012 ; 0.005)	0.408
BCG: 41 participant(s) censored on randomisation date and . participant(s) had event starting on randomisation date. Control: 94 participant(s) censored on randomisation date and . participant(s) had event starting on randomisation date.				
Model adjusted for : subgroup_1 subgroup_2_rand subgroup_3				
Soft DB Lock on . Descriptive Part : generated on 20231101				
Table completed on 20231101 - POP = itt_pop - Bootstrap = 1000				

3.8 NEED FOR OXYGEN THERAPY FOR A FEBRILE OR RESPIRATORY ILLNESS BY 12 MONTHS

Objective	Estimand
To determine if BCG vaccination compared with Control reduces the need for oxygen therapy irrespective of receiving any other vaccine (including COVID-19 specific vaccine), over the 12 months following randomisation, in healthcare workers exposed to SARS-CoV-2.	**Estimand 24.1**<br><br>Population: ITT population<br>Outcome: need of oxygen therapy by 12 months<br>Interventions: BCG vs Control<br>Handling of Intercurrent events:<br>- any vaccine, including COVID-19 specific vaccine (**Treatment Policy strategy**)<br>Summary Measure: as for estimand 24.1

Table 3.15 Estimand 24.1 – Oxygen therapy (ITT), Treatment policy Strategy

	BCG	Control	Difference (BCG-Control)	P value
	N=3417	N=3411		
Participants in itt_pop	3417	3411		
OXYGEN THERAPY	12/3417 (0.35%)	6/3411 (0.18%)		
Censoring				
Censored- 12 months w/ no event	3097/3417 (90.64%)	2901/3411 (85.05%)		
Censored- incomplete data entry/drop-out from the study	308/3417 (9.01%)	504/3411 (14.78%)		
Person years	3226	3083		
Event rate (per 100 person years)	0.37 95%CI(0.21 ; 0.66)	0.19 95%CI(0.09 ; 0.43)		
Estimated probability of Fever or Resp Illness				
- Unadjusted	0.004 95%CI(0.002 ; 0.006)	0.002 95%CI(0.000 ; 0.003)	0.002 95%CI(-0.001 ; 0.004)	0.176
- Adjusted for stratification factors	0.004 95%CI(0.002 ; 0.005)	0.002 95%CI(0.000 ; 0.003)	0.002 95%CI(-0.001 ; 0.004)	0.206

BCG: 38 participant(s) censored on randomisation date and . participant(s) had event starting on randomisation date. Control: 87 participant(s) censored on randomisation date and . participant(s) had event starting on randomisation date.

Model adjusted for : subgroup_1 subgroup_2_rand subgroup_3

Soft DB Lock on . Descriptive Part : generated on 20231101

Table completed on 20231101 - POP = itt_pop - Bootstrap = 1000

Objective	Estimand
To determine if BCG vaccination compared with Control reduces the need for oxygen therapy in the absence of any vaccine, measured over 12 months following randomisation in healthcare exposed to SARS-CoV-2.	Estimand 24.2<br><br>Population: ITT population<br>Outcome: need of oxygen therapy by 12 months<br>Interventions: BCG vs Control<br>Handling of Intercurrent events:<br>- any vaccine, including COVID-19 specific vaccine (**Hypothetical strategy**)<br>Summary Measure: Adjusted difference in proportion of participants

Table 3.16 Estimand 24.2 – Oxygen therapy (ITT), Hypothetical Strategy

	BCG	Control	Difference (BCG-Control)	P value	Hazard Ratio (BCG/Control)	P value
	N=3417	N=3411				
Participants in itt_pop	3417	3411				
OXYGEN THERAPY	5/3417 (0.15%)	4/3411 (0.12%)				
Censoring						
Censored- Vaccination < 12 months	2823/3417 (82.62%)	2675/3411 (78.42%)				
Censored- 12 months w/ no event	347/3417 (10.16%)	327/3411 (9.59%)				
Censored- incomplete data entry/drop-out from the study	242/3417 (7.08%)	405/3411 (11.87%)				
Person years	1736	1632				
Event rate (per 100 person years)	0.29 95%CI(0.12 ; 0.69)	0.25 95%CI(0.09 ; 0.65)				
Estimated probability of Fever or Resp Illness						
- Unadjusted	0.002 95%CI(0.000 ; 0.005)	0.001 95%CI(-0.000 ; 0.003)	0.001 95%CI(-0.002 ; 0.004)	0.446	1.198 95%CI(0.322 ; 4.461)	0.788
- Adjusted for stratification factors					1.189 95%CI(0.319 ; 4.437)	0.797

BCG: 41 participant(s) censored on randomisation date and . participant(s) had event starting on randomisation date. Control: 94 participant(s) censored on randomisation date and . participant(s) had event starting on randomisation date.
Model adjusted for : subgroup_1 subgroup_2_rand subgroup_3
Soft DB Lock on . Descriptive Part : generated on 20231101
Table completed on 20231101 - POP = itt_pop - Bootstrap = 1000

3.9 ADMISSION TO CRITICAL CARE FOR A FEBRILE OR RESPIRATORY ILLNESS BY 12 MONTHS

Objective	Estimand
To determine if BCG vaccination compared with Control reduces admission to critical care following a febrile or respiratory illness irrespective of receiving any vaccine, including COVID-19 specific vaccine (including COVID-19 specific vaccine), over the 12 months following randomisation, in healthcare workers exposed to SARS-CoV-2.	**Estimand 25.1**<br><br>Population: ITT population<br>Outcome: admission to critical care following a febrile or respiratory illness by 12 months<br>Interventions: BCG vs Control<br>Handling of Intercurrent events:<br>- any vaccine, including COVID-19 specific vaccine (**Treatment Policy strategy**)<br>Summary Measure: as for estimand 25.1

Table 3.17 Estimand 25.1 –Admission to critical care (ITT), Treatment policy Strategy

	BCG	Control	Difference (BCG-Control)	P value	Hazard Ratio (BCG/Control)	P value
	N=3417	N=3411				
Participants in itt_pop	3417	3411				
Admission to critical care	5/3417 (0.15%)	3/3411 (0.09%)				
Censoring						
Censored- 12 months w/ no event	3104/3417 (90.84%)	2904/3411 (85.14%)				
Censored- incomplete data entry/drop-out from the study	308/3417 (9.01%)	504/3411 (14.78%)				
Person years	3230	3085				
Event rate (per 100 person years)	0.15 95%CI(0.06 ; 0.37)	0.10 95%CI(0.03 ; 0.30)				
Estimated probability of Fever or Resp Illness						
- Unadjusted	0.002 95%CI(0.000 ; 0.003)	0.001 95%CI(0.000 ; 0.002)	0.001 95%CI(-0.001 ; 0.002)	0.414	1.608 95%CI(0.384 ; 6.736)	0.516
- Adjusted for stratification factors					1.664 95%CI(0.397 ; 6.977)	0.486

BCG: 38 participant(s) censored on randomisation date and . participant(s) had event starting on randomisation date. Control: 87 participant(s) censored on randomisation date and . participant(s) had event starting on randomisation date.

Model adjusted for : subgroup_1 subgroup_2_rand subgroup_3

Soft DB Lock on . Descriptive Part : generated on 20231101

Table completed on 20231101 - POP = itt_pop - Bootstrap = 1000

Objective	Estimand
To determine if BCG vaccination compared with Control reduces admission to critical care following a febrile or respiratory illness in the absence of any vaccine, measured over 12 months following randomisation in healthcare exposed to SARS-CoV-2.	Estimand 25.2<br><br>Population: ITT population<br>Outcome: admission to critical care following a febrile or respiratory illness by 12 months<br>Interventions: BCG vs Control<br>Handling of Intercurrent events:<br>- any vaccine, including COVID-19 specific vaccine (**Hypothetical strategy**)<br>Summary Measure: Adjusted difference in proportion of participants

Table 3.18 Estimand 25.2 –Admission to critical care (ITT), Hypothetical Strategy

	BCG	Control	Difference (BCG-Control)	P value	Hazard Ratio (BCG/Placebo)	P value
	N=3417	N=3411				
Participants in itt_pop	3417	3411				
Admission to critical care	2/3417 (0.06%)	2/3411 (0.06%)				
Censoring						
Censored- Vaccination < 12 months	2826/3417 (82.70%)	2676/3411 (78.45%)				
Censored- 12 months w/ no event	347/3417 (10.16%)	327/3411 (9.59%)				
Censored- incomplete data entry/drop-out from the study	242/3417 (7.08%)	406/3411 (11.90%)				
Person years	1736	1633				
Event rate (per 100 person years)	0.12 95%CI(0.03 ; 0.46)	0.12 95%CI(0.03 ; 0.49)				
Estimated probability of Fever or Resp Illness						
- Unadjusted	0.001 95%CI(-0.000 ; 0.002)	0.001 95%CI(-0.000 ; 0.001)	0.000 95%CI(-0.001 ; 0.001)	0.923	0.972 95%CI(0.137 ; 6.897)	0.977
- Adjusted for stratification factors					0.980 95%CI(0.138 ; 6.958)	0.984

BCG: 41 participant(s) censored on randomisation date and . participant(s) had event starting on randomisation date. Control: 94 participant(s) censored on randomisation date and . participant(s) had event starting on randomisation date.

Model adjusted for : subgroup_1 subgroup_2_rand subgroup_3

Soft DB Lock on . Descriptive Part : generated on 20231101

Table completed on 20231101 - POP = itt_pop - Bootstrap = 1000

3.10 NEED FOR MECHANICAL VENTILATION (MV) FOR A FEBRILE OR RESPIRATORY ILLNESS BY 12 MONTHS

Objective	Estimand
To determine if BCG vaccination compared with Control reduces Need of MV for a febrile or respiratory illness irrespective of receiving any vaccine, including COVID-19 specific vaccine , over the 12 months following randomisation, in healthcare workers exposed to SARS-CoV-2.	Estimand 26.1<br><br>Population: ITT population<br>Outcome: Need of MV for a febrile or respiratory illness by 12 months<br>Interventions: BCG vs Control<br>Handling of Intercurrent events:<br>- any vaccine, including COVID-19 specific vaccine (**Treatment Policy strategy**)<br>Summary Measure: as for estimand 26.1

Table 3.19 Estimand 26.1 – Need of Mechanical Ventilation (ITT), Treatment policy Strategy

	BCG	Control	Hazard Ratio (BCG/Control)	P value
	N=3417	N=3411		
Participants in itt_pop	3417	3411		
Need of MV	2/3417 (0.06%)	1/3411 (0.03%)		
Censoring				
Censored- 12 months w/ no event	3107/3417 (90.93%)	2906/3411 (85.19%)		
Censored- incomplete data entry/drop-out from the study	308/3417 (9.01%)	504/3411 (14.78%)		
Person years	3232	3086		
Event rate (per 100 person years)	0.06 95%CI(0.02 ; 0.25)	0.03 95%CI(0.00 ; 0.23)		
Estimated probability				
- Unadjusted			1.946 95%CI(0.176 ; 21.463)	0.587
- Adjusted for stratification factors			1.982 95%CI(0.180 ; 21.854)	0.577

BCG: 38 participant(s) censored on randomisation date and . participant(s) had event starting on randomisation date. Control: 87 participant(s) censored on randomisation date and . participant(s) had event starting on randomisation date.

Model adjusted for : subgroup_1 subgroup_2_rand subgroup_3

Soft DB Lock on . Descriptive Part : generated on 20231101

Listing 3.1 Estimand 26.1 –List of participants who needed Mechanical Ventilation (ITT), Treatment policy Strategy

arm	participant_id	ra_rand_date_local_dt	po_fever	po_fever_dt	mr_hosp_dateadm	mr_hosp_datedisch	mr_hosp_respsupp___4	mv_dur
BCG	14994	13/11/2020	Need of MV	11/12/2020	26/12/2020	2/04/2021	Yes	96
BCG	16882	9/12/2020	Need of MV	24/01/2021	24/01/2021	11/02/2021	Yes	5
Control	14609	6/11/2020	Need of MV	28/11/2020	6/12/2020	25/12/2020	Yes	17

Objective	Estimand
To determine if BCG vaccination compared with Control reduces the need of MV for a febrile or respiratory illness in the absence of any vaccine, measured over 12 months following randomisation in healthcare exposed to SARS-CoV-2.	**Estimand 26.2**<br><br>Population: ITT population<br>Outcome: Need of MV for a febrile or respiratory illness by 12 months<br>Interventions: BCG vs Control<br>Handling of Intercurrent events:<br>- any vaccine, including COVID-19 specific vaccine (**Hypothetical strategy**)<br>Summary Measure: Adjusted difference in proportion of participants

Table 3.20 Estimand 26.2 – Need of Mechanical Ventilation (ITT), Hypothetical Strategy

	BCG	Control		Hazard Ratio (BCG/Control)	P value
	N=3417	N=3411			
Participants in itt_pop	3417	3411			
Need of MV	2/3417 (0.06%)	1/3411 (0.03%)			
Censoring					
Censored- Vaccination < 12 months	2826/3417 (82.70%)	2678/3411 (78.51%)			
Censored- 12 months w/ no event	347/3417 (10.16%)	327/3411 (9.59%)			
Censored- incomplete data entry/drop-out from the study	242/3417 (7.08%)	405/3411 (11.87%)			
.	2/3417 (0.06%)	1/3411 (0.03%)			
Person years	1736	1633			
Event rate (per 100 person years)	0.12 95%CI(0.03 ; 0.46)	0.06 95%CI(0.01 ; 0.43)			
Estimated probability of Fever or Resp Illness					
- Unadjusted				1.943 95%CI(0.176 ; 21.428)	0.588
- Adjusted for stratification factors				1.968 95%CI(0.178 ; 21.704)	0.580
BCG: 41 participant(s) censored on randomisation date and . participant(s) had event starting on randomisation date. Control: 94 participant(s) censored on randomi:					
Model adjusted for : subgroup_1 subgroup_2_rand subgroup_3					
Soft DB Lock on . Descriptive Part : generated on 20231101					

3.11 DEATHS AS A CONSEQUENCE OF AN EPISODE OF FEVER OR RESPIRATORY ILLNESS BY 12 MONTHS

Objective	Estimand
To determine if BCG vaccination compared with Control reduces the incidence of death as a consequence of an episode of fever or respiratory illness irrespective of receiving any vaccine, including COVID-19 specific vaccine, measured over 12 months following randomisation in healthcare exposed to SARS-CoV-2.	Estimand 27.1<br><br>Population: ITT population<br>Outcome: death as a consequence of an episode of fever or respiratory illness by 12 months<br>Interventions: BCG vs Control<br>Handling of Intercurrent events:<br>- any vaccine, including COVID-19 specific vaccine (Treatment Policy strategy)<br>Summary Measure: as for estimand 27.1

Table 3.21 Estimand 27.1 – Death (ITT), Treatment Policy Strategy

	BCG	Control	Hazard Ratio (BCG/Control)	P value
	N=3417	N=3411		
Participants in itt_pop	3417	3411		
Death	0/3417 (0.00%)	3/3411 (0.09%)		
Censoring				
Censored- 12 months w/ no event	3151/3417 (92.22%)	2973/3411 (87.16%)		
Censored- incomplete data entry/drop-out from the study	266/3417 (7.78%)	435/3411 (12.75%)		
.	0/3417 (0.00%)	3/3411 (0.09%)		
Person years	3282	3170		
Event rate (per 100 person years)	0.00 95%CI(. ; .)	0.09 95%CI(0.03 ; 0.29)		

BCG: 13 participant(s) censored on randomisation date and . participant(s) had event starting on randomisation date. Control: 23 participant(s) censored on randomisation date and . participant(s) had event starting on randomisation date.

Model adjusted for : subgroup_1 subgroup_2_rand subgroup_3

Soft DB Lock on . Descriptive Part : generated on 20230605

Listing 3.2 Estimand 27.1 –List of participants who died as a consequence of an episode of fever or respiratory illness (ITT), Treatment policy Strategy

arm	participant_id	ra_rand_date_local_dt	po_fever	po_fever_dt	death	date_death_dt	dth_primary	dth_primary_oth
Control	14609	6/11/2020	Death	28/11/2020	Yes	25/12/2020	Covid-19	
Control	16255	1/12/2020	Death	5/05/2021	Yes	9/06/2021	Covid-19	
Control	19391	18/02/2021	Death	17/12/2021	Yes	19/12/2021	Other	Unspecified pneumonia

Objective	Estimand
To determine if BCG vaccination compared with Control reduces the incidence of death as a consequence of an episode of fever or respiratory illness in the absence of receiving any vaccine, including COVID-19 specific vaccine, measured over 12 months following randomisation in healthcare exposed to SARS-CoV-2.	Estimand 27.2<br><br>Population: ITT population<br>Outcome: death as a consequence of an episode of fever or respiratory illness by 12 months<br>Interventions: BCG vs Control<br>Handling of Intercurrent events:<br>- any vaccine, including COVID-19 specific vaccine (Hypothetical strategy)<br>Summary Measure: Adjusted difference in proportion of participants

Table 3.22 Estimand 27.2 – Death (ITT), Hypothetical Strategy

	BCG	Control	Hazard Ratio (BCG/Control)	P value
	N=3417	N=3411		
Participants in itt_pop	3417	3411		
Death	0/3417 (0.00%)	1/3411 (0.03%)		
Censoring				
Censored- Vaccination < 12 months	2830/3417 (82.82%)	2693/3411 (78.95%)		
Censored- 12 months w/ no event	378/3417 (11.06%)	364/3411 (10.67%)		
Censored- incomplete data entry/drop-out from the study	209/3417 (6.12%)	353/3411 (10.35%)		
.	0/3417 (0.00%)	1/3411 (0.03%)		
Event rate (per 100 person years)	0.00 95%CI(. ; .)	0.06 95%CI(0.01 ; 0.42)		

BCG: 16 participant(s) censored on randomisation date and . participant(s) had event starting on randomisation date. Control: 30 participant(s) censored on randomisation date and . participant(s) had event starting on randomisation date.

Model adjusted for : subgroup_1 subgroup_2_rand subgroup_3

Soft DB Lock on . Descriptive Part : generated on 20230605

ID of participant who died is 14609 (see listing 3.2)

3.12 HOSPITALISATION FOR A FEBRILE OR RESPIRATORY ILLNESS AND DURATION OF HOSPITALISATION BY 12 MONTHS

3.12.1 HOSPITALISATION FOR A FEBRILE OR RESPIRATORY ILLNESS, TREATMENT POLICY STRATEGY

Objective	Estimand
To determine if BCG vaccination compared with Control reduces the incidence of hospitalisation for an episode of fever or respiratory illness irrespective of receiving any vaccine, including COVID-19 specific vaccine, measured over 12 months following randomisation in healthcare exposed to SARS-CoV-2.	Estimand 28.1<br><br>Population: ITT population<br>Outcome: hospitalisation for an episode of fever or respiratory illness by 12 months<br>Interventions: BCG vs Control<br>Handling of Intercurrent events:<br>- any vaccine, including COVID-19 specific vaccine (**Treatment Policy strategy**)<br>Summary Measure: as for estimand 28.1

Table 3.23.1 Estimand 28.1 Hospitalisation for a febrile or respiratory illness (ITT), Treatment policy Strategy

	BCG	Control	Difference (BCG-Control)	P value
	N=3417	N=3411		
Participants in itt_pop	3417	3411		
Hospitalisation	27/3417 (0.79%)	20/3411 (0.59%)		
Censoring				
Censored- 12 months w/ no event	3084/3417 (90.25%)	2890/3411 (84.73%)		
Censored- incomplete data entry/drop-out from the study	306/3417 (8.96%)	501/3411 (14.69%)		
Person years	3220	3079		
Event rate (per 100 person years)	0.84 95%CI(0.58 ; 1.22)	0.65 95%CI(0.42 ; 1.01)		
Estimated probability of Fever or Resp Illness				
- Unadjusted	0.008 95%CI(0.005 ; 0.011)	0.007 95%CI(0.004 ; 0.009)	0.002 95%CI(-0.002 ; 0.006)	0.405
- Adjusted for stratification factors	0.008 95%CI(0.005 ; 0.011)	0.006 95%CI(0.004 ; 0.009)	0.002 95%CI(-0.002 ; 0.006)	0.382

BCG: 38 participant(s) censored on randomisation date and . participant(s) had event starting on randomisation date. Control: 87 participant(s) censored on randomisation date and . participant(s) had event starting on randomisation date.

Model adjusted for : subgroup_1 subgroup_2_rand subgroup_3

Soft DB Lock on . Descriptive Part : generated on 20231101

Table completed on 20231101 - POP = itt_pop - Bootstrap = 1000

Listing 3.3 Estimand 28.1 –List of participants who were hospitalisation (ITT), Treatment policy Strategy

arm	participant_id	rand_date	fever_startdt	hosp_date	Date_discharge	COVID19 test results	Reason for hospitalisation
BCG	1033	2/04/2020	30/05/2020	30/05/2020	31/05/2020	Unknown, not tested for COVID-19	Other infection
BCG	11775	4/09/2020	22/11/2020	22/11/2020	26/11/2020	Unknown, not tested for COVID-19	Other infection
BCG	12243	15/09/2020	21/11/2020	21/11/2020	22/11/2020	No, all COVID-19 tests were negative	Other infection
BCG	12893	8/10/2020	26/02/2021	6/03/2021	19/03/2021	Yes, COVID-19 test positive	COVID-19 related (acute infection or post infectious inflammatory disease)
BCG	13696	16/10/2020	9/01/2021	20/01/2021	25/01/2021	Yes, COVID-19 test positive	COVID-19 related (acute infection or post infectious inflammatory disease)
BCG	13959	22/10/2020	14/04/2021	17/05/2021	21/05/2021	Yes, COVID-19 test positive	COVID-19 related (acute infection or post infectious inflammatory disease)
BCG	14265-1	9/04/2020	26/12/2020	28/12/2020	30/12/2020	Unknown, not tested for COVID-19	Other infection
BCG	14282	29/10/2020	24/02/2021	8/03/2021	9/03/2021	Yes, COVID-19 test positive	COVID-19 related (acute infection or post infectious inflammatory disease)
BCG	14680	5/11/2020	30/01/2021	30/01/2021	1/02/2021	Unknown, not tested for COVID-19	Other infection
BCG	14922	12/11/2020	26/08/2021	26/08/2021	27/08/2021	Unknown, not tested for COVID-19	Other infection
BCG	14994	13/11/2020	11/12/2020	26/12/2020	2/04/2021	Yes, COVID-19 test positive	COVID-19 related (acute infection or post infectious inflammatory disease)
BCG	15692	23/11/2020	13/07/2021	13/07/2021	18/07/2021	No, all COVID-19 tests were negative	Respiratory tract infection, not COVID-19 related
BCG	16386	1/12/2020	24/08/2021	8/09/2021	11/09/2021	Yes, COVID-19 test positive	COVID-19 related (acute infection or post infectious inflammatory disease)
BCG	16882	9/12/2020	24/01/2021	24/01/2021	11/02/2021	Yes, COVID-19 test positive	COVID-19 related (acute infection or post infectious inflammatory disease)
BCG	17120	15/12/2020	2/05/2021	10/05/2021	11/05/2021	Unknown, not tested for COVID-19	Other infection
BCG	17290	18/12/2020	15/02/2021	1/03/2021	9/03/2021	Yes, COVID-19 test positive	COVID-19 related (acute infection or post infectious inflammatory disease)
BCG	17331	21/12/2020	25/04/2021	1/05/2021	6/05/2021	Yes, COVID-19 test positive	COVID-19 related (acute infection or post infectious inflammatory disease)
BCG	18122	8/01/2021	18/07/2021	28/07/2021	30/07/2021	Yes, COVID-19 test positive	COVID-19 related (acute infection or post infectious inflammatory disease)
BCG	18484	18/01/2021	6/04/2021	19/04/2021	22/04/2021	No, all COVID-19 tests were negative	Respiratory tract infection, not COVID-19 related
BCG	18760	28/01/2021	20/06/2021	25/06/2021	11/07/2021	Yes, COVID-19 test positive	COVID-19 related (acute infection or post infectious inflammatory disease)
BCG	18863	1/02/2021	13/08/2021	13/08/2021	16/08/2021	Unknown, not tested for COVID-19	Other infection
BCG	19008	1/02/2021	7/10/2021	7/10/2021	10/10/2021	Unknown, not tested for COVID-19	Other infection
BCG	2502	2/04/2020	22/12/2020	26/12/2020	27/12/2020	No, all COVID-19 tests were negative	Other infection
BCG	7900	20/04/2020	5/11/2020	5/11/2020	27/11/2020	No, all COVID-19 tests were negative	Other infection
BCG	8783	1/05/2020	11/04/2021	13/04/2021	15/04/2021	Unknown, not tested for COVID-19	Other infection
BCG	8931	19/05/2020	21/01/2021	21/01/2021	29/01/2021	No, all COVID-19 tests were negative	Other infection
BCG	9230	27/05/2020	21/04/2021	21/04/2021	23/04/2021	Unknown, not tested for COVID-19	Other infection
Control	10104	14/07/2020	26/06/2021	26/06/2021	28/06/2021	Unknown, not tested for COVID-19	Other infection
Control	13518	21/10/2020	5/07/2021	5/07/2021	11/07/2021	Unknown, not tested for COVID-19	Other infection
Control	14163	28/10/2020	22/05/2021	27/05/2021	9/06/2021	Yes, COVID-19 test positive	COVID-19 related (acute infection or post infectious inflammatory disease)
Control	14609	6/11/2020	28/11/2020	6/12/2020	25/12/2020	Yes, COVID-19 test positive	COVID-19 related (acute infection or post infectious inflammatory disease)
Control	15886	25/11/2020	15/12/2020	22/12/2020	25/12/2020	Yes, COVID-19 test positive	COVID-19 related (acute infection or post infectious inflammatory disease)
Control	15960	25/11/2020	5/12/2020	16/12/2020	23/12/2020	Yes, COVID-19 test positive	COVID-19 related (acute infection or post infectious inflammatory disease)
Control	16141	27/11/2020	6/11/2021	6/11/2021	8/11/2021	Unknown, not tested for COVID-19	Respiratory tract infection, not COVID-19 related

arm	participant_id	rand_date	fever_startdt	hosp_date	Date_discharge	COVID19 test results	Reason for hospitalisation
Control	1639	30/03/2020	5/07/2020	14/07/2020	15/07/2020	No, all COVID-19 tests were negative	Respiratory tract infection, not COVID-19 related
Control	17384	19/12/2020	6/03/2021	16/03/2021	21/03/2021	Yes, COVID-19 test positive	COVID-19 related (acute infection or post infectious inflammatory disease)
Control	18008	6/01/2021	15/03/2021	29/03/2021	1/04/2021	Unknown, not tested for COVID-19	Other infection
Control	18312	12/01/2021	19/06/2021	30/06/2021	1/07/2021	No, all COVID-19 tests were negative	Other infection
Control	18329	26/01/2021	16/01/2022	18/01/2022	27/01/2022	No, all COVID-19 tests were negative	Other infection
Control	18395	14/01/2021	22/12/2021	23/12/2021	25/12/2021	No, all COVID-19 tests were negative	Respiratory tract infection, not COVID-19 related
Control	18450	15/01/2021	24/04/2021	4/05/2021	6/05/2021	Yes, COVID-19 test positive	COVID-19 related (acute infection or post infectious inflammatory disease)
Control	18487	18/01/2021	13/01/2022	16/01/2022	18/01/2022	Yes, COVID-19 test positive	COVID-19 related (acute infection or post infectious inflammatory disease)
Control	19021	1/02/2021	11/01/2022	22/01/2022	23/01/2022	Unknown, not tested for COVID-19	Respiratory tract infection, not COVID-19 related
Control	19391	18/02/2021	17/12/2021	19/12/2021	19/12/2021	Unknown, not tested for COVID-19	Respiratory tract infection, not COVID-19 related
Control	2401	1/04/2020	22/12/2020	22/12/2020	23/12/2020	No, all COVID-19 tests were negative	Respiratory tract infection, not COVID-19 related
Control	2714	3/04/2020	30/04/2020	1/05/2020	5/05/2020	No, all COVID-19 tests were negative	Other infection
Control	4097	10/04/2020	31/01/2021	31/01/2021	5/02/2021	No, all COVID-19 tests were negative	Other infection

3.12.2 HOSPITALISATION FOR A FEBRILE OR RESPIRATORY ILLNESS, HYPOTETICAL STRATEGY

Objective	Estimand
To determine if BCG vaccination compared with Control reduces the incidence of hospitalisation for an episode of fever or respiratory illness in the absence of receiving any vaccine, including COVID-19 specific vaccine, measured over 12 months following randomisation in healthcare exposed to SARS-CoV-2.	Estimand 28.2<br><br>Population: ITT population<br>Outcome: hospitalisation for an episode of fever or respiratory illness by 12 months<br>Interventions: BCG vs Control<br>Handling of Intercurrent events:<br>- any vaccine, including COVID-19 specific vaccine (**Hypothetical strategy**)<br>Summary Measure: Adjusted difference in proportion of participants

Table 3.24.1 Estimand 28.2 Hospitalisation for a febrile or respiratory illness (ITT), Hypothetical Strategy

	BCG	Control	Difference (BCG-Control)	P value
	N=3417	N=3411		
Participants in itt_pop	3417	3411		
Hospitalisation	10/3417 (0.29%)	9/3411 (0.26%)		
Censoring				
Censored- Vaccination < 12 months	2819/3417 (82.50%)	2674/3411 (78.39%)		
Censored- 12 months w/ no event	347/3417 (10.16%)	325/3411 (9.53%)		
Censored- incomplete data entry/drop-out from the study	241/3417 (7.05%)	403/3411 (11.81%)		
Person years	1734	1631		
Event rate (per 100 person years)	0.58 95%CI(0.31 ; 1.07)	0.55 95%CI(0.29 ; 1.06)		
Estimated probability of Fever or Resp Illness				
- Unadjusted	0.005 95%CI(0.002 ; 0.008)	0.005 95%CI(0.001 ; 0.008)	0.000 95%CI(-0.004 ; 0.005)	0.832
- Adjusted for stratification factors	0.008 95%CI(0.000 ; 0.016)	0.008 95%CI(-0.000 ; 0.016)	0.000 95%CI(-0.008 ; 0.008)	0.981

BCG: 41 participant(s) censored on randomisation date and . participant(s) had event starting on randomisation date. Control: 94 participant(s) censored on randomisation date and . participant(s) had event starting on randomisation date.

Model adjusted for : subgroup_1 subgroup_2_rand subgroup_3

Soft DB Lock on . Descriptive Part : generated on 20231101

Table completed on 20231101 - POP = itt_pop - Bootstrap = 1000

Listing 3.4 Estimand 28.2 –List of participants who were hospitalisation (ITT), Hypothetical Strategy

arm	participant_id	rand_date	fever_startdt	hosp_date	Date_discharge	COVID19 test results	Reason for hospitalisation
BCG	1033	2/04/2020	30/05/2020	30/05/2020	31/05/2020	Unknown, not tested for COVID-19	Other infection
BCG	11775	4/09/2020	22/11/2020	22/11/2020	26/11/2020	Unknown, not tested for COVID-19	Other infection
BCG	12243	15/09/2020	21/11/2020	21/11/2020	22/11/2020	No, all COVID-19 tests were negative	Other infection
BCG	13696	16/10/2020	9/01/2021	20/01/2021	25/01/2021	Yes, COVID-19 test positive	COVID-19 related (acute infection or post infectious inflammatory disease)
BCG	14265-1	9/04/2020	26/12/2020	28/12/2020	30/12/2020	Unknown, not tested for COVID-19	Other infection
BCG	14994	13/11/2020	11/12/2020	26/12/2020	2/04/2021	Yes, COVID-19 test positive	COVID-19 related (acute infection or post infectious inflammatory disease)
BCG	16882	9/12/2020	24/01/2021	24/01/2021	11/02/2021	Yes, COVID-19 test positive	COVID-19 related (acute infection or post infectious inflammatory disease)
BCG	17331	21/12/2020	25/04/2021	1/05/2021	6/05/2021	Yes, COVID-19 test positive	COVID-19 related (acute infection or post infectious inflammatory disease)
BCG	7900	20/04/2020	5/11/2020	5/11/2020	27/11/2020	No, all COVID-19 tests were negative	Other infection
BCG	8931	19/05/2020	21/01/2021	21/01/2021	29/01/2021	No, all COVID-19 tests were negative	Other infection
Control	14609	6/11/2020	28/11/2020	6/12/2020	25/12/2020	Yes, COVID-19 test positive	COVID-19 related (acute infection or post infectious inflammatory disease)
Control	15886	25/11/2020	15/12/2020	22/12/2020	25/12/2020	Yes, COVID-19 test positive	COVID-19 related (acute infection or post infectious inflammatory disease)
Control	15960	25/11/2020	5/12/2020	16/12/2020	23/12/2020	Yes, COVID-19 test positive	COVID-19 related (acute infection or post infectious inflammatory disease)
Control	1639	30/03/2020	5/07/2020	14/07/2020	15/07/2020	No, all COVID-19 tests were negative	Respiratory tract infection, not COVID-19 related
Control	17384	19/12/2020	6/03/2021	16/03/2021	21/03/2021	Yes, COVID-19 test positive	COVID-19 related (acute infection or post infectious inflammatory disease)
Control	18450	15/01/2021	24/04/2021	4/05/2021	6/05/2021	Yes, COVID-19 test positive	COVID-19 related (acute infection or post infectious inflammatory disease)
Control	2401	1/04/2020	22/12/2020	22/12/2020	23/12/2020	No, all COVID-19 tests were negative	Respiratory tract infection, not COVID-19 related
Control	2714	3/04/2020	30/04/2020	1/05/2020	5/05/2020	No, all COVID-19 tests were negative	Other infection
Control	4097	10/04/2020	31/01/2021	31/01/2021	5/02/2021	No, all COVID-19 tests were negative	Other infection

3.13 NUMBER OF DAYS OF UNPLANNED ABSENTEEISM FOR AN ACUTE ILLNESS OR HOSPITALISATION BY 12 MONTHS

Objective	Estimand
To determine if BCG vaccination compared with Control reduces absenteeism, measured over 12 months following randomisation in healthcare.	Estimand 29b.1<br><br>Population: ITT population<br>Outcome: Number of days of unplanned absenteeism for an acute illness or hospitalisation by 12 months<br>Interventions: BCG vs Control<br>Handling of Intercurrent events:<br>- any vaccine, including COVID-19 specific vaccine (Treatment Policy strategy)<br>Summary Measure: Mean difference

Table 3.25 Estimand 29.1b Number of days of unplanned absenteeism for an acute illness or hospitalisation by 12 months (ITT), Treatment Policy Strategy

	BCG	Control	Difference in Logs of Expected Counts (BCG-Control)	Incidence Rate Ratio (BCG v Control)	P value
	N=3417	N=3411			
Participants in itt_pop	3417	3411			
Participants with data	2961/3417 (86.7%)	2731/3411 (80.1%)			
Number of Days of Unplanned Absenteeism , Median(IQR)	0.0 (0.0-4.0)	0.0 (0.0-4.0)			
Number of Days of Unplanned Absenteeism , Mean(SD)	3.7 (9.9)	3.5 (8.7)			
Number of Days of Unplanned Absenteeism in subgroup with >=1 day of absenteeism, Median(IQR)	5.0 (2.0-10.0)	5.0 (2.0-10.0)			
Number of Days of Unplanned Absenteeism in subgroup with >=1 day of absenteeism, Mean(SD)	8.2 (13.4)	8.4 (11.7)			
Count of events					
Unadjusted			-0.015 95%CI(-0.092 ; 0.061)	0.985 95%CI(0.912 ; 1.063)	0.691
Adjusted			-0.042 95%CI(-0.115 ; 0.031)	0.959 95%CI(0.891 ; 1.032)	0.262

Model adjusted for : subgroup_1 (age group), subgroup_2_rand (presence of comorbidity), subgroup_3 (geographical location)

Soft DB Lock on . Descriptive Part : generated on 20231103

Zero-inflated negative binomial model used as the preferred model.

pvalue interaction arm##subgroup_1 = 0.1999 ; pvalue interaction arm##subgroup_2_rand = 0.0039 ; pvalue interaction arm##subgroup_3 = 0.0124

3.13.1 SUBGROUP ANALYSES BY PRESENCE OF COMORBIDITIES: NUMBER OF DAYS OF UNPLANNED ABSENTEEISM FOR AN ACUTE ILLNESS OR HOSPITALISATION BY 12 MONTHS

Table 3.25.1 Estimand 29.1b by PRESENCE OF COMORBIDITIES – Number of days of unplanned absenteeism for an acute illness or hospitalisation by 12 months (ITT), Treatment Policy Strategy

	BCG	Control	Difference in Logs of Expected Counts (BCG-Control)	Incidence Rate Ratio (BCG v Control)	P value
Participants Subgroup 2 = With Comorbidities	613	625			
Participants with data	554/613 (90.4%)	519/625 (83.0%)			
Number of Days of Unplanned Absenteeism , Median(IQR)	0.0 (0.0-5.0)	0.0 (0.0-5.0)			
Number of Days of Unplanned Absenteeism , Mean(SD)	5.3 (16.2)	3.7 (7.5)			
Number of Days of Unplanned Absenteeism in subgroup with >=1 day of absenteeism N, Median(IQR)	258, 6.0 (2.0-14.0)	216, 6.0 (3.0-11.0)			
Number of Days of Unplanned Absenteeism in subgroup with >=1 day of absenteeism N, Mean(SD)	258, 11.3 (22.2)	216, 8.9 (9.4)			
Comparison of counts					
Unadjusted			0.243 95%CI(0.058 ; 0.428)	1.275 95%CI(1.060 ; 1.535)	0.010
Adjusted			0.141 95%CI(-0.037 ; 0.320)	1.152 95%CI(0.963 ; 1.377)	0.121
Participants Subgroup 2 = Without Comorbidities	2804	2786			
Participants with data	2407/2804 (85.8%)	2212/2786 (79.4%)			
Number of Days of Unplanned Absenteeism , Median(IQR)	0.0 (0.0-4.0)	0.0 (0.0-4.0)			
Number of Days of Unplanned Absenteeism , Mean(SD)	3.3 (7.7)	3.5 (8.9)			
Number of Days of Unplanned Absenteeism in subgroup with >=1 day of absenteeism N, Median(IQR)	1074, 5.0 (2.0-10.0)	937, 5.0 (2.0-10.0)			
Number of Days of Unplanned Absenteeism in subgroup with >=1 day of absenteeism N, Mean(SD)	1074, 7.5 (10.1)	937, 8.2 (12.2)			
Comparison of counts					
Unadjusted			-0.096 95%CI(-0.179 ; -0.013)	0.909 95%CI(0.836 ; 0.987)	0.023
Adjusted			-0.093 95%CI(-0.173 ; -0.013)	0.911 95%CI(0.842 ; 0.987)	0.023

3.13.2 SUBGROUP ANALYSES BY GEOGRAPHICAL AREA: NUMBER OF DAYS OF UNPLANNED ABSENTEEISM FOR AN ACUTE ILLNESS OR HOSPITALISATION BY 12 MONTHS

Table 3.25.2 Estimand 29.1b by PRESENCE OF COMORBIDITIES – Number of days of unplanned absenteeism for an acute illness or hospitalisation by 12 months (ITT), Treatment Policy Strategy

	BCG	Control	Difference in Logs of Expected Counts (BCG-Control)	Incidence Rate Ratio (BCG v Control)	P value
Participants Subgroup 3 = Australia-Stage 1	1418	1422			
Participants with data	1096/1418 (77.3%)	924/1422 (65.0%)			
Number of Days of Unplanned Absenteeism, Median(IQR)	1.0 (0.0-4.0)	1.0 (0.0-4.0)			
Number of Days of Unplanned Absenteeism , Mean(SD)	3.0 (6.3)	3.7 (8.2)			
Number of Days of Unplanned Absenteeism in subgroup with >=1 day of absenteeism N, Median(IQR)	604, 3.0 (2.0-6.0)	516, 4.0 (2.0-7.5)			
Number of Days of Unplanned Absenteeism in subgroup with >=1 day of absenteeism N, Mean(SD)	604, 5.5 (7.7)	516, 6.6 (10.1)			
Comparison of counts			-0.176 95%CI(-0.289 ; -0.063)	0.839 95%CI(0.749 ; 0.939)	0.002
Unadjusted			-0.172 95%CI(-0.286 ; -0.059)	0.842 95%CI(0.752 ; 0.942)	0.003
Adjusted					
Participants Subgroup 3 = Australia-Stage 2	216	206			
Participants with data	188/216 (87.0%)	174/206 (84.5%)			
Number of Days of Unplanned Absenteeism, Median(IQR)	1.0 (0.0-4.0)	0.0 (0.0-3.0)			
Number of Days of Unplanned Absenteeism , Mean(SD)	3.7 (7.7)	2.8 (6.5)			
Number of Days of Unplanned Absenteeism in subgroup with >=1 day of absenteeism N, Median(IQR)	118, 3.0 (2.0-7.0)	86, 3.0 (2.0-6.0)			
Number of Days of Unplanned Absenteeism in subgroup with >=1 day of absenteeism N, Mean(SD)	118, 6.0 (9.1)	86, 5.6 (8.4)			
Comparison of counts			0.060 95%CI(-0.219 ; 0.340)	1.062 95%CI(0.803 ; 1.405)	0.672
Unadjusted			0.029 95%CI(-0.252 ; 0.311)	1.030 95%CI(0.777 ; 1.365)	0.838
Adjusted					
Participants Subgroup 3 = Europe	498	500			
Participants with data	464/498 (93.2%)	437/500 (87.4%)			
Number of Days of Unplanned Absenteeism, Median(IQR)	0.0 (0.0-2.0)	0.0 (0.0-2.0)			
Number of Days of Unplanned Absenteeism , Mean(SD)	4.7 (18.3)	3.4 (14.2)			
Number of Days of Unplanned Absenteeism in subgroup with >=1 day of absenteeism N, Median(IQR)	162, 4.5 (2.0-12.0)	137, 3.0 (2.0-10.0)			
Number of Days of Unplanned Absenteeism in subgroup with >=1 day of absenteeism N, Mean(SD)	162, 13.5 (29.1)	137, 11.0 (23.7)			
Comparison of counts			0.209 95%CI(-0.081 ; 0.498)	1.232 95%CI(0.922 ; 1.646)	0.158

	BCG	Control	Difference in Logs of Expected Counts (BCG-Control)	Incidence Rate Ratio (BCG v Control)	P value
Unadjusted			0.156 95%CI(-0.135 ; 0.447)	1.169 95%CI(0.874 ; 1.563)	0.293
Adjusted					
Participants Subgroup 3 = South America	1285	1283			
Participants with data	1213/1285 (94.4%)	1196/1283 (93.2%)			
Number of Days of Unplanned Absenteeism, Median(IQR)	0.0 (0.0-7.0)	0.0 (0.0-7.0)			
Number of Days of Unplanned Absenteeism , Mean(SD)	3.9 (8.0)	3.6 (6.3)			
Number of Days of Unplanned Absenteeism in subgroup with >=1 day of absenteeism N, Median(IQR)	448, 10.0 (5.0-14.0)	414, 10.0 (5.0-14.0)			
Number of Days of Unplanned Absenteeism in subgroup with >=1 day of absenteeism N, Mean(SD)	448, 10.6 (10.1)	414, 10.3 (6.8)			
Comparison of counts			0.027 95%CI(-0.064 ; 0.119)	1.028 95%CI(0.938 ; 1.126)	0.556
Unadjusted			0.020 95%CI(-0.072 ; 0.111)	1.020 95%CI(0.931 ; 1.117)	0.674
Adjusted					

Supplementary table: Numerators and denominators for subgroup analyses defined by randomisation strata

Subgroup analysis defined by randomisation strata	BCG	Control
**Number of days with symptoms** [a]		
Comorbidity randomisation strata		
Presence of any comorbidity	407/613	367/625
Absence of any comorbidity	1807/2804	1662/2786
Region strata		
Australia stage 1	776/1418	684/1422
Australia stage 2	149/216	116/206
Europe	317/498	306/500
South America	972/1285	923/1283
**Number of days unable to work** [a]		
Age group randomisation strata		
<40 years-old	1133/1592	1041/1594
40 to 59 years-old	952/1566	867/1557
≥60 years-old	129/259	121/260
Comorbidity randomisation strata		
Presence of any comorbidity	407/613	367/625
Absence of any comorbidity	1807/2804	1662/2786
**Number of days of unplanned absenteeism** [b]		
Comorbidity randomisation strata		
Presence of any comorbidity	258/554	216/519
Absence of any comorbidity	1074/2407	937/2212
Region strata		
Australia stage 1	604/1096	516/924
Australia stage 2	118/188	86/174
Europe	162/464	137/437
South America	448/1213	414/1196

[a] Numerators = number of cases of fever or respiratory illness in stratum; Denominators = number of participants in stratum.

[b] Numerators = number of participants with >=1 day of absenteeism in stratum; Denominators = number of participants with available data on absenteeism in stratum.

BRACE trial Consortium Group

Australia (Victoria)

MCRI Central Team: **Prof Nigel Curtis**, Prof Andrew Davidson, Kaya Gardiner, A/Prof Amanda Gwee, Tenaya Jamieson, Dr Nicole Messina, Thilanka Morawakage, Dr Susan Perlen, A/Prof Kirsten Perrett, Dr Laure Pittet, Amber Sastry, Jia Wei Teo;

Biostatisticians: **Francesca Orsini**, Prof Katherine Lee, Dr Cecilia Moore, Suzanna Vidmar;

Data Team: **Dr Laure Pittet**, Rashida Ali, Ross Dunn, Peta Edler, Grace Gell, Casey Goodall, Richard Hall, Ann Krastev, Dr Nathan La, Dr Ellie McDonald, Nick McPhate, Thao Nguyen, Jack Ren, Luke Stevens;

Laboratory Core Team: **Dr Nicole Messina**, Ahmed Alamrousi, Rhian Bonnici, Dr Thanh Dang, Susie Germano, Jenny Hua, Rebecca McElroy, Monica Razmovska, Scott Reddiex, Xiaofang Wang; Laboratory Scientists: Jeremy Anderson, Kristy Azzopardi, Vicki Bennett- Wood, Anna Czajko, Nadia Mazarakis, Conor McCafferty, Frances Oppedisano, Belinda Ortika, Casey Pell, Leena Spry, Ryan Toh, Sunitha Velagapudi, Amanda Vlahos, Ashleigh Wee-Hee; Biobanking: **Pedro Ramos**, Karina De La Cruz, Dinusha Gamage, Anushka Karunanayake, Isabella Mezzetti, Dr Benjamin Ong, Ronita Singh, Enoshini Sooriyarachchi;

Serology testing (VIDRL): **Dr Suellen Nicholson**, Natalie Cain, Rianne Brizuela, Han Huang;

Study Visit and Phone Call Team: **Veronica Abruzzo**, Morgan Bealing, Patricia Bimboese, Kirsty Bowes, Emma Burrell, Dr Joyce Chan, Jac Cushnahan, Hannah Elborough, Olivia Elkington, Kieran Fahey, Monique Fernandez, Catherine Flynn, Sarah Fowler, Marie Gentile Andrit, Bojana Gladanac, Catherine Hammond, Norine Ma, Sam Macalister, Emmah Milojevic, Jesutofunmi Mojeed, Jill Nguyen, Liz O'Donnell, Nadia Olivier, Isabelle Ooi, Stephanie Reynolds, Lisa Shen, Barb Sherry, Judith Spotswood, Jamie Wedderburn, Angela Younes;

Pharmacy Team: **Donna Legge,** Jason Bell, Jo Cheah, Annie Cobbledick, Kee Lim;

Immunisation Team: **Sonja Elia**, Lynne Addlem, Anna Bourke, Clare Brophy, Nadine Henare, Narelle Jenkins, Francesca Machingaifa, Skye Miller, Kirsten Mitchell, Sigrid Pitkin, Kate Wall;

Safety and Quality Monitoring Team: **Dr Paola Villanueva**, A/Prof Nigel Crawford, Dr Laure Pittet, Dr Wendy Norton;

Epworth Healthcare: **Dr Niki Tan**, Thilakavathi Chengodu, Diane Dawson, Victoria Gordon;

Monash Health: **Tony Korman**, Jess O'Bryan, Veronica Abruzzo;

MCRI Start-up Support Team: Sophie Agius, Dr Samantha Bannister, Jess Bucholc, Alison Burns, Beatriz Camesella, Prof John Carlin, Marianna Ciaverella, Maxwell Curtis, Stephanie Firth, Dr Christina Guo, Matthew Hannan, Erin Hill, Sri Joshi, Katherine Lieschke, Megan Mathers, Sasha Odoi, Ashleigh Rak, Dr Chris Richards, Leah Steve, Carolyn Stewart, Dr Eva Sudbury, Helen Thomson, Emma Watts, Fiona Williams, Angela Young;

Legal: **Penny Glenn**, Andrew Kaynes, Amandine Philippart De Floy App Development: Sandy Buchanan, Thijs Sondag, Ivy Xie;

Media and Communications: **Harriet Edmund**, Bridie Byrne, Tom Keeble, Belle Ngien, Fran Noonan, Michelle Wearing-Smith;

Orygen Volunteers: Alison Clarke, Pemma Davies, Oliver Eastwood, Alric Ellinghaus, Rachid Ghieh, Zahra Hilton, Emma Jennings, Athina Kakkos, Iris Liang, Katie Nicol, Sally O'Callaghan, Helen Osman, Gowri Rajaram, Sophia Ratcliffe, Victoria Rayner, Ashleigh Salmon, Angela Scheppokat, Aimee Stevens, Rebekah Street, Nicholas Toogood.

Australia (New South Wales)

Westmead Children's Hospital: **A/Prof Nicholas Wood**, Twinkle Bahaduri, Therese Baulman, Jennifer Byrne, Candace Carter, Mary Corbett, Aiken Dao, Maria Desylva, Dr Andrew Dunn, Evangeline Gardiner, Rosemary Joyce, Dr Rama Kandasamy, Prof Craig Munns, Lisa Pelayo, Dr Ketaki Sharma, Katrina Sterling, Caitlin Uren; Westmead Hospital: Clinton Colaco, A/Prof Mark Douglas, Kate Hamilton; Sydney Children's Hospital: Dr Adam Bartlett, Dr Brendan McMullan, Dr Pamela Palasanthiran, Dr Phoebe Williams; Prince of Wales Hospital: Dr Justin Beardsley, Nikki Bergant, Renier Lagunday, Dr Kristen Overton, Prof Jeffrey Post; St Vincent's Hospital Sydney: Dr Yasmeen Al- Hindawi, Sarah Barney, A/Prof Anthony Byrne, Lee Mead, Marshall Plit.

Australia (South Australia)

SAHMRI: **Prof. David Lynn**, Saoirse Benson, Dr Stephen Blake, Rochelle Botten, Tee Yee Chern, Georgina Eden, Liddy Griffith, Jane James, Dr Miriam Lynn, Angela Markow, Domenic Sacca, Dr Natalie Stevens, Prof. Steve Wesselingh; Royal Adelaide Hospital: Catriona Doran, Dr Simone Barry, Dr Alice Sawka; Women's and Children's Hospital: Dr Sue Evans, Louise Goodchild, Christine Heath, Meredith Krieg, Prof. Helen Marshall, Mark McMillan, Mary Walker.

Australia (Western Australia)

Perth Children's Hospital/Telethon Kids Institute: **Prof Peter Richmond**, Nelly Amenyogbe, Christina Anthony, Annabelle Arnold, Beth Arrowsmith, Rym Ben-Othman, Sharon Clark, Jemma Dunnill, Nat Eiffler, Krist Ewe, Carolyn Finucane, Lorraine Flynn, Camille Gibson, Lucy Hartnell, Elysia Hollams, Heidi Hutton, Lance Jarvis, Jane Jones, Jan Jones, Karen Jones, Jennifer Kent, Prof Tobias Kollmann, Debbie Lalich, Wenna Lee, Rachel Lim, Sonia McAlister, Fiona McDonald, Andrea Meehan, Asma Minhaj, Lisa Montgomery, Melissa O'Donnell, Jaslyn Ong, Joanne Ong, Kimberley Parkin, Glady Perez, Catherine Power, Shadie Rezazadeh, Holly Richmond, Sally Rogers, Nikki Schultz, Margaret Shave, Patrycja Skut, Lisa Stiglmayer, Alexandra Truelove, Dr Ushma Wadia, Rachael Wallace, Justin Waring; Fiona Stanley Hospital: Michelle England, Erin Latkovic, A/Prof Laurens Manning; Sir Charles Gardiner: Dr Susan Herrmann, Prof Michaela Lucas.

Brazil (Manaus)

Manaus: **Dr Marcus Lacerda**, Paulo Henrique Andrade, Fabiane Bianca Barbosa, Dayanne Barros, Larissa Brasil, Ana Greyce Capella, Ramon Castro, Erlane Costa, Dilcimar de Souza, Maianne Dias, José Dias, Klenilson Ferreira, Paula Figueiredo, Thamires Freitas, Ana Carolina Furtado, Larissa Gama, Vanessa Godinho, Cintia Gouy, Daniele Hinojosa, Dr Bruno Jardim, Dr Tyane Jardim, Joel Junior, Augustto Lima, Bernardo Maia, Adriana Marins, Kelry Mazurega, Tercilene Medeiros, Rosangela Melo, Marinete Moraes, Elizandra Nascimento, Juliana Neves, Maria Gabriela Oliveira, Thais Oliveira, Ingrid Oliveira, Arthur Otsuka, Rayssa Paes, Handerson Pereira, Gabrielle Pereira, Christiane Prado, Evelyn Queiroz, Laleyska Rodrigues, Bebeto Rodrigues, Dr Vanderson Sampaio, Anna Gabriela Santos, Daniel Santos, Tilza Santos, Evelyn Santos, Ariandra Sartim, Ana Beatriz Silva, Juliana Silva, Emanuelle Silva, Mariana Simão, Caroline Soares, Antonny Sousa, Alexandre Trindade, Dr Fernando Val, Adria Vasconcelos, Heline Vasconcelos.

Brazil (Mato Grosso do Sul)

Mato Grosso do Sul: **Prof Julio Croda**, Carolinne Abreu, Katya Martinez Almeida, Camila Bitencourt de Andrade, Jhenyfer Thalyta Campos Angelo, Ghislaine Gonçalvez de Araújo Arcanjo, Bianca Maria Silva Menezes Arruda, Wellyngthon Espindola Ayala, Adelita Agripina Refosco Barbosa, Felipe Zampieri Vieira Batista, Fabiani de Morais Batista, Miriam de Jesus Costa, Dr Mariana Garcia Croda, Lais Alves da Cruz, Roberta Carolina Pereira Diogo, Rodrigo Cezar Dutra Escobar, Iara Rodrigues Fernandes, Leticia Ramires Figueiredo, Leandro Galdino Cavalcanti Gonçalves, Sarita Lahdo, Joyce dos Santos Lencina, Guilherme Teodoro de Lima, Larissa Santos Matos, Bruna Tayara Leopoldina Meireles, Debora Quadros Moreira, Lilian Batista Silva Muranaka, Adriely de Oliveira, Karla Regina Warszawski de Oliveira, Matheus Vieira de Oliveira, Prof Roberto Dias de Oliveira, Andrea Antonia Souza de Almeida dos Reis Pereira, Marco Puga, Caroliny Veron Ramos, Thaynara Haynara Souza da Rosa, Karla Lopes dos Santos, Claudinalva Ribeiro dos Santos, Dyenyffer Stéffany Leopoldina dos Santos, Karina Marques Santos, Paulo César Pereira da Silva, Paulo Victor Rocha da Silva, Débora dos Santos Silva, Patricia Vieira da Silva, Bruno Freitas da Rosa Soares, Mariana Gazzoni Sperotto, Mariana Mayumi Tadokoro, Daniel Tsuha, Hugo Miguel Ramos Vieira.

Brazil (Rio de Janeiro)

Rio de Janeiro: **Prof Margareth Maria Pretti Dalcolmo**, Cíntia Maria Lopes Alves da Paixão, Gabriela Corrêa E Castro, Simone Silva Collopy, Renato da Costa Silva, Samyra Almeida da Silveira, Alda Maria Da-Cruz, Alessandra Maria da Silva Passos de Carvalho, Rita de Cássia Batista, Maria Luciana Silva De Freitas, Aline Gerhardt de Oliveira Ferreira, Ana Paula Conceição de Souza, Paola Cerbino Doblas, Ayla Alcoforado da Silva dos Santos, Vanessa Cristine de Moraes dos Santos, Dayane Alves dos Santos Gomes, Anderson Lage Fortunato, Adriano Gomes-Silva, Monique Pinto Gonçalves, Paulo Leandro Garcia Meireles Junior, Estela Martins da Costa Carvalho, Fernando do Couto Motta, Ligia Maria Olivo de Mendonça, Girlene dos Santos Pandine, Rosa Maria Plácido Pereira, Ivan Ramos Maia, Jorge Luiz da Rocha, João Victor Paiva Romano, Glauce dos Santos, Erica Fernandes da Silva, Marilda Agudo Mendonça Teixeira de Siqueira, Ágatha Cristinne Prudêncio Soares.

The Netherlands

UMC Utrecht: **Prof Marc Bonten,** Sandra Franch Arroyo, Henny Ophorst-den Besten, Anna Boon, Karin M Brakke, Axel Janssen, Marijke A.H. Koopmans, Toos Lemmens, Titia Leurink, A/Prof Cristina Prat-Aymerich, Engelien Septer-Bijleveld, Kimberly Stadhouders, Dr Darren Troeman, Marije van der Waal, Marjoleine van Opdorp, Nicolette van Sluis, Beatrijs Wolters; Amphia Hospital: Prof Jan Kluytmans, Jannie Romme, Dr Wouter van den Bijllaardt, Linda van Mook, Dr M.M.L (Miranda) van Rijen; Rijnstate Hospital: P. M. G. Filius, Jet Gisolf, Frances Greven, Danique Huijbens, Dr Robert Jan Hassing, R. C. Pon, Lieke Preijers, J. H. van Leusen, Harald Verheij; Noord West Ziekenhuis: Dr Wim Boersma, Evelien Brans, Paul Kloeg, Kitty Molenaar-Groot, Nhat Khanh Nguyen, Dr Nienke Paternotte, Anke Rol, Lida Stooper; Radboud UMC: Helga Dijkstra, Esther Eggenhuizen, Lucas Huijs, Dr Simone Moorlag, Prof Mihai Netea, Eva Pranger, Dr Esther Taks, Dr Jaap ten Oever, Rob ter Heine; St Antonius Hospital: Kitty Blauwendraat, Dr Bob Meek, Isil Erkaya, Houda Harbech, Dr Nienke Roescher, Rifka Peeters, Menno te Riele, Carmen Zhou.

Spain

Mutua Terrassa University Hospital: Dr Esther Calbo, Cristina Badia Marti, Emma Triviño Palomares, Tomás Perez Porcuna; University Hospital Germans Trias I Pujol: Anabel Barriocanal, Ana Maria Barriocanal, Irma Casas, Jose Dominguez, Maria Esteve, Alicia Lacoma, Irene Latorre, Gemma Molina, Barbara Molina, Dr Antoni Rosell, Sandra Vidal; Hospital Virgen Macarena: Lydia Barrera, Natalia Bustos, Ines Portillo Calderón, David Gutierrez Campos, Jose Manuel Carretero, Angel Dominguez Castellano, Renato Compagnone, Encarnacion Ramirez de Arellano, Almudena de la Serna, Maria Dolores del Toro Lopez, Marie-Alix Clement Espindola, Ana Belen Martin Gutierrez, Alvaro Pascual Hernandez, Virginia Palomo Jiménez, Elisa Moreno, Nicolas Navarrete, Teresa Rodriguez Paño, Prof Jesús Rodríguez-Baño, Enriqueta Tristán, Maria Jose Rios Villegas; University Hospital Cruces: Atsegiñe Canga Garces, Erika Castro Amo, Raquel Coya Guerrero, Dr.Josune Goikoetxea, Leticia Jorge, Cristina Perez; Marqués de Valdecilla University Hospital: Dr María Carmen Fariñas Álvarez, Manuel Gutierrez Cuadra, Dr Francisco Arnaiz de las Revillas Almajano, Pilar Bohedo Garcia, Dr Teresa Giménez Poderos, Claudia González Rico, Blanca Sanchez, Olga Valero, Noelia Vega.

United Kingdom

University of Exeter/Exeter Clinical Trials Unit: **Prof John Campbell,** Anna Barnes, Dr Helen Catterick, Tim Cranston, Phoebe Dawe, Emily Fletcher, Liam Fouracre, Dr Alison Gifford, Prof Neil Gow, John Kirkwood, Dr Christopher Martin, Dr Amy McAndrew, Marcus Mitchell, Georgina Newman, Dr Abby O'Connell, Jakob Onysk, Lynne Quinn, Dr Shelley Rhodes, Samuel Stone, Dr Lorrie Symons, Harry Tripp, Prof Adilia Warris, Darcy Watkins, Bethany Whale; St Leonard's Practice: Dr Alex Harding, Gemma Lockhart, Dr Kate Sidaway-Lee; Ide Lane Surgery: Dr John Campbell, Dr Sam Hilton, Sarah Manton, Dr Daniel Webber-Rookes, Rachel Winder; Travel Clinic: James Moore; Royal Devon and Exeter NHS Foundation Trust: Freya Bateman, Dr Michael Gibbons, Dr Bridget Knight, Julie Moss, Dr Sarah Statton, Josephine Studham; Teign Estuary Medical Group/Glendevon Medical Practice: Lydia Hall, Will Moyle, Dr Tamsin Venton.
